# Highly selective palladium-catalyzed one-pot, five-fold B–H/C–H cross coupling of monocarboranes with alkenes†
†Electronic supplementary information (ESI) available: Experimental details, compound characterization and X-ray crystallographic data in CIF format. CCDC 1886699–1886701. For ESI and crystallographic data in CIF or other electronic format see DOI: 10.1039/c9sc00078j


**DOI:** 10.1039/c9sc00078j

**Published:** 2019-03-04

**Authors:** Yunjun Shen, Kang Zhang, Xuewei Liang, Rakesh Dontha, Simon Duttwyler

**Affiliations:** a Department of Chemistry , Zhejiang University , 310027 Hangzhou , Zhejiang , P. R. China . Email: duttwyler@zju.edu.cn

## Abstract

Palladium-catalyzed dehydrogenative B–H/C–H cross coupling of monocarborane anions with alkenes is reported, allowing for the first time the isolation of selectively penta-alkenylated boron clusters. The reaction cascade is regioselective for the cage positions, leading directly to B2–6 functionalization. Under mild and convenient conditions, styrenes, benzylic alkenes and aliphatic alkenes are demonstrated to be viable coupling partners with exclusive vinyl-type B–C bond formation. Multiple subsequent transformations provide access to directing group-free products, chiral derivatives and penta-alkylated cages. The five-fold coupling, combined with the latter reactions, represents a powerful methodology for the straightforward synthesis of new classes of boron clusters.

## Abstract 2

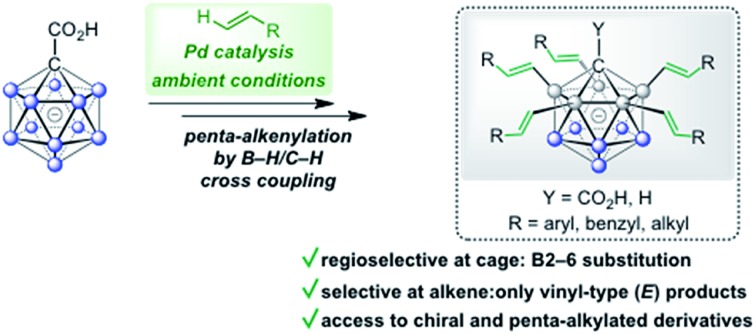
Palladium-catalyzed five-fold B–H/C–H cross coupling of monocarboranes with alkenes enables the synthesis of selectively penta-functionalized boron clusters.

## Introduction

Icosahedral monocarboranes based on the [CB_11_H_12_]^–^ anion and dicarbaboranes with the parent skeleton C_2_B_10_H_12_ are polyhedral 12-vertex boranes with one or two BH vertices replaced by CH. They possess unique steric and electronic properties that set them apart from traditional organic or inorganic building blocks.^1^,^2^ Their sphere-like distribution of electron density gives rise to remarkable chemical and thermal stability, including low toxicity. Monocarboranes are well known as weakly coordinating anions,^3^ and various applications of monocarboranes and dicarbaboranes have been reported in areas such as coordination,^4^ supramolecular^5^ and medicinal chemistry,^6^ as well as luminescence^7^ and materials science.^8^ In order to exploit the potential of carboranes in these fields, improved methodologies which enable the coupling of cluster vertices to organic substituents are essential.

**Fig. 1:**
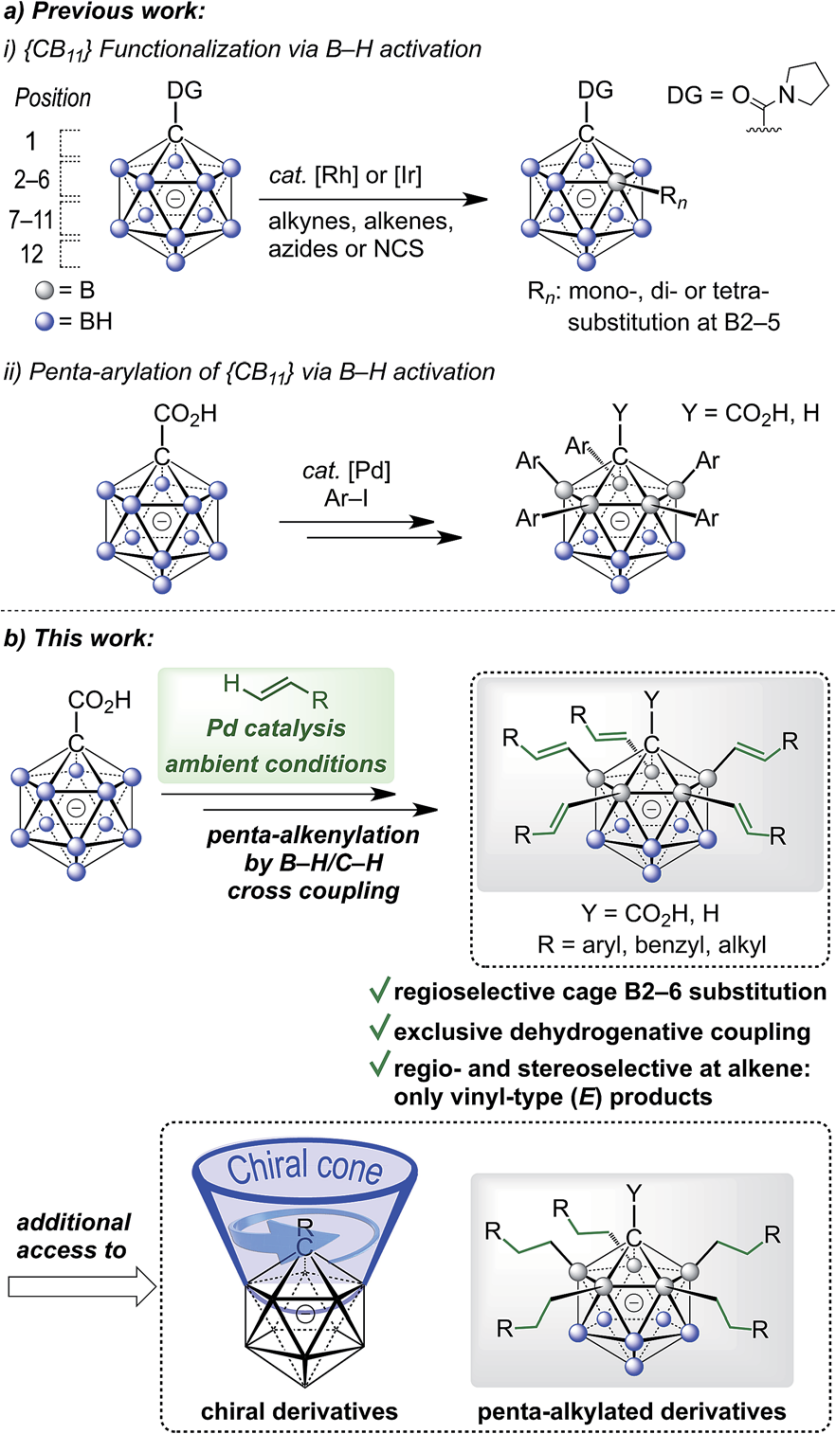
(a) Previously reported metal-catalyzed functionalization of {CB_11_} cages and (b) regioselective dehydrogenative penta-alkenylation by direct B–H/C–H cross coupling.

C–C bond forming reactions are at the heart of classical organic synthesis. Likewise, when it comes to the preparation of inorganic–organic hybrid molecules, the construction of E–C bonds (E = main group element other than C) is equally important. For icosahedral carboranes, the modification of boron vertices traditionally relies on direct electrophilic substitution or electrophilic halogenation followed by transformation to a B–X (X = C, N, O, S, P) bond.^9^,^10^ Drawbacks of these approaches are harsh conditions, limited control over selectivity of the substituted positions and moderate overall yields. The functionalization of boron vertices by directing group-mediated, metal-catalyzed B–H activation is a highly attractive alternative strategy. Over the past years, the utility of this concept to provide access to selectively modified carborane derivatives has been demonstrated impressively.^11^,^12^ Among others, the groups of Xie, Yan, Bregadze, Spokoyny and Peryshkov have established effective methodologies involving neutral dicarbaboranes.^13^,^14^ Our group, on the other hand, has focused on catalytic B–H activation of mono- and dianionic boron clusters. These procedures have made use of amide or ureido directing groups for Rh- and Ir-catalyzed reactions with acrylates, styrenes, sulfones, alkynes, azides and *N*-chlorosuccinimide.^15^ These transformations allow mono- to tetra-substitution of boron vertices. Reactions with acrylates or styrenes resulted either in one- or two-fold oxidative (vinyl-type) coupling or higher mixed oxidative/reductive coupling.15b,d,f Recently, we have also achieved penta-arylation of the monocarborane-1-carboxylic acid with aryl halides under Pd catalysis (Fig. 1a).15*e* Herein, we report the first Pd-catalyzed penta-functionalization of [CB_11_H_12_]^–^-based anions by selective B–H/C–H cross coupling with a wide range of alkenes at room temperature under air (Fig. 1b). The substitution is highly regioselective with respect to the cage at the B2–6 positions, and the coupling to the alkene occurs regio- and stereoselectively with regard to the alkene, giving vinyl-type (*E*) products exclusively. This mild and ligand-free catalytic protocol using a removable directing group represents a powerful method for the construction of highly substituted monocarborane clusters.

## Results and discussion

In order to establish conditions for this intermolecular coupling, we studied the model reaction between **1a** and 4-fluorostyrene (**2a**, 5.4 equivalents) to give functionalized product **3a** with potential multiple substitution (Table 1). No reaction occurred in the presence of only catalyst Pd(OAc)_2_ or AgOAc in acetonitrile (entries 1 and 2). On the other hand, a combination of Pd(ii) (10 mol%) and Ag(i) (10 equivalents) cleanly afforded **3a** in 86% isolated yield, as evidenced by reaction monitoring using ESI-mass spectrometry and NMR spectroscopy of the purified product (entry 3). The penta-substitution occurred at 25 °C within 24 hours; increasing the temperature to 60 °C was associated with a faster completion (*ca.* 12 hours) and slightly reduced yield (entry 4). The use of other solvents, such as tetrahydrofuran, 1,2-dichloroethane or dimethylacetamide, led to reduced yields (entries 5 and 7). Oxidants such as Cu(OAc)_2_ or 1,4-benzoquinone were ineffective (entries 8 and 9); interestingly, for Cu(OAc)_2_ the reaction stopped at the mono- and di-substitution stage (inseparable mixture), and no higher degree of substitution was observed. We also tested the use of smaller amounts of Pd(ii) and Ag(i); lowering the catalyst or oxidant loading afforded the desired **3a**, but as a mixture with di-, tri- and tetra-substituted products even after 48 hours. All of the above screening reactions were set up in a fumehood under air atmosphere. Applying the conditions from entry 3 under nitrogen in a glovebox gave **3a** in 84% yield, indicating that the presence of oxygen does not play a significant role in this transformation.

**Table 1:** Optimization of reaction conditions

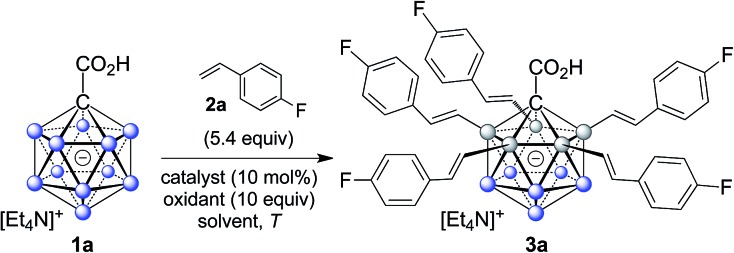					
Entry^*a*^	Catalyst	Oxidant	Solv.^*b*^	*T* [°C]	Result^*c*^
1	Pd(OAc)_2_	None	ACN	25	N.R.
2	None	AgOAc	ACN	25	N.R.
**3**	**Pd(OAc)**_**2**_	**AgOAc**	**ACN**	**25**	**86%**
4	Pd(OAc)_2_	AgOAc	ACN	60	83%
5	Pd(OAc)_2_	AgOAc	THF	25	32%
6	Pd(OAc)_2_	AgOAc	DCE	25	10%
7	Pd(OAc)_2_	AgOAc	DMA	25	72%
8	Pd(OAc)_2_	Cu(OAc)_2_	ACN	25	0
9	Pd(OAc)_2_	BQ	ACN	25	30%

^*a*^ **1a** (0.15 mmol), Pd(OAc)_2_ (0.015 mmol), oxidant (1.5 mmol), in 4 mL of solvent.

^*b*^ACN = acetonitrile, DMA = dimethylacetamide, DCE = 1,2-dichloroethane, THF = tetrahydrofuran.

^*c*^Isolated yields after purification by silica gel chromatography.

**Fig. 2:**
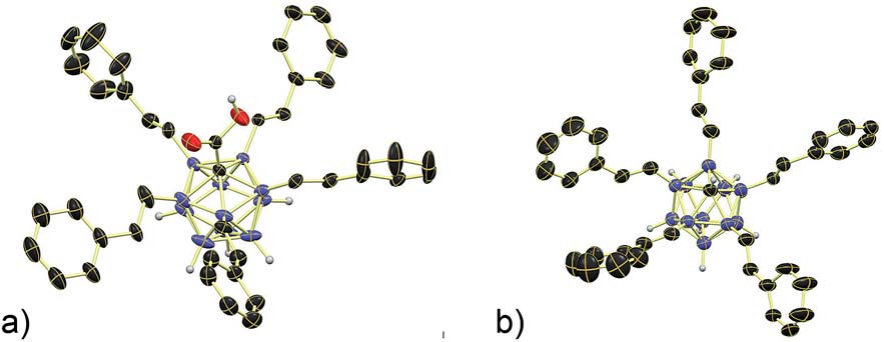
X-ray crystal structures of (a) **3b** and (b) **4b** (cations, solvent molecules and styryl H atoms omitted for clarity, 30% displacement ellipsoids).

Subsequently, we studied the substrate scope of the penta-alkenylation under the aforementioned conditions of Table 1, entry 3. All reactions were conveniently set up and run under air. The *ortho* B–H alkenylation of acid carborane **1** with an array of terminal alkenes proceeded smoothly to give the desired products in moderate to high yields (Table 2). Coupling with electron-neutral and electron-deficient styrenes bearing 4-fluoro, 4-hydrogen, 4-trifluoromethyl, and 4-cyano groups gave the desired products **3a–e** in yields of 53–86%. 4-Methoxystyrene and 4-methylstyrene as an electron-rich alkene were also tested; however, these substrates afforded only small amounts of product, and attempted chromatographic separation of the reaction mixtures showed that compounds with a lower degree of substitution were also present. Benzylic alkenes with CH_2_-phenyl and CH_2_-pentafluorophenyl substituents gave **3f** and **3g** in yields of 72% and 59%. The reaction was equally successful with aliphatic substrates of different steric requirements, such as hex-1-ene, 4-methylpent-1-ene, 4-methyl-hex-1-ene, 4,4-dimethylpent-1-ene and 4-phenylbut-1-ene, with consistently good yields of 79–84% (**3h–3l**). Notably, functionalized oxygen-containing alkenes were found to be compatible applying this protocol to give ester **3m** and ether **3n** in yields of 66% and 74%, respectively. Even though for alkenes **2** with R = benzyl or alkyl, isomeric allyl-type products could form, only vinyl-type coupling was observed (*vide infra*, discussion of the mechanism). We then chose styrene and 4-fluorostyrene as coupling partners to explore the penta-alkenylation of multiple acid carboranes **1** bearing different substituents at the B12 position. The five-fold selective *ortho* B–H alkenylation occurred with B12–R (R = Cl, Br, Me, Ph, CN) in yields of 62–82% (**3o–t**). Furthermore, the method is also reproducible on a gram scale; starting from 1.0 g of **1a**, product **3a** was obtained in 80% isolated yield (2.3 g). Clusters **3** were fully characterized by ^1^H, ^1^H{^11^B}, ^13^C{^1^H}, ^11^B and ^11^B{^1^H} NMR spectroscopy as well as high-resolution mass spectrometry. Upon substitution of the B2–6 positions, a characteristic change in the ^11^B NMR spectra was observed. Comparing the starting material **1a** and **3a** as a representative product, the B2–6 resonances appeared at –14.1 and –6.1 ppm, while almost no change was observed for positions B7–11 (–13.3/–12.9 ppm) and B12 (–6.5/–6.1 ppm). Deshielding of the substituted positions by *ca.* 8 ppm occurred in all cases, while the chemical shift differences for the other vertices were very small. For **3b**, the molecular structures was in addition elucidated by X-ray crystallography; Fig. 2a shows an ORTEP representation of this product.^16^

**Table 2:** Dehydrogenative penta-alkenylation of **1**^*a*^ ^,^^*b*^

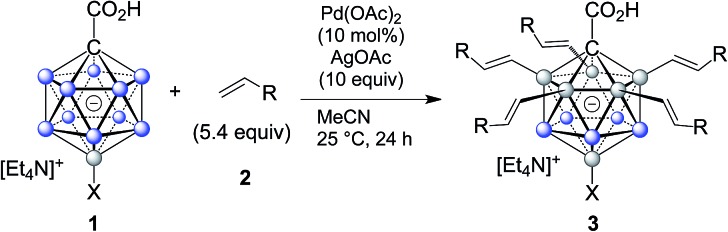				
Carborane		Alkene		Product
**1a**	X = H	R = aryl	R = C_6_H_4_-4-F	**3a** 86%
	X = H		R = C_6_H_5_	**3b** 75%
	X = H		R = C_6_H_4_-4-CF_3_	**3c** 70%
	X = H		R = C_6_H_4_-4-CN	**3d** 63%
	X = H		R = C_6_F_5_	**3e** 53%
	X = H	R = benzyl	R = CH_2_-C_6_H_5_	**3f** 72%
	X = H		R = CH_2_-C_6_F_5_	**3g** 59%
	X = H	R = alkyl	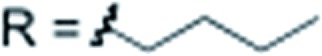	**3h** 81%
	X = H		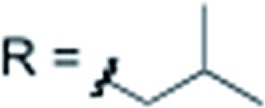	**3i** 84%
	X = H		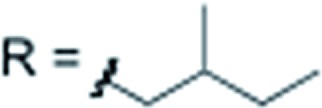	**3j** 82%
	X = H		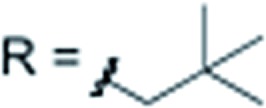	**3k** 79%
	X = H		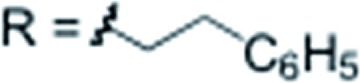	**3l** 76%
	X = H		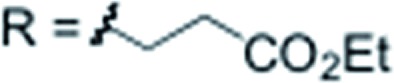	**3m** 66%
	X = H		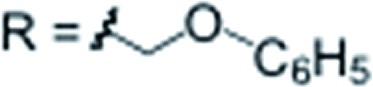	**3n** 74%
**1b**	X = Cl	R = aryl	R = C_6_H_5_	**3o** 71%
**1c**	X = Br		R = C_6_H_4_-4-F	**3p** 78%
**1d**	X = Me		R = C_6_H_4_-4-F	**3q** 82%
**1e**	X = Ph		R = C_6_H_5_	**3r** 62%
**1e**	X = Ph		R = C_6_H_4_-4-F	**3s** 69%
**1f**	X = CN		R = C_6_H_4_-4-F	**3t** 72%

^*a*^ **1** (0.15 mmol), **2** (0.81 mmol), Pd(OAc)_2_ (0.015 mmol), AgOAc (1.5 mmol), in acetonitrile (4 mL); for further details, see the ESI.

^*b*^Yields = isolated yields after purification by silica gel chromatography.

The carboxylic acid moiety at the C1 cluster position is a highly versatile directing group. On the one hand, it can be transformed to the acid chloride, which in turn is a very useful functional group handle for the preparation of, *e.g.*, esters and amides.^17^ Product **3c** was chosen as a representative example, and details about its clean conversion to the acid chloride **3c–Cl** are provided in the ESI (p. S12).† Moreover, we recently demonstrated that penta-arylated monocarborane C1-carboxylic acids can be decarboxylated in dimethylformamide at 100 °C to give the C1–H product.15*e* For products **3**, these conditions did not lead to complete directing group removal even after prolonged periods of heating. However, we found that decarboxylation takes place cleanly under microwave irradiation under slightly basic conditions. Specifically, at 150 °C in dimethylacetamide solvent and with the addition of 10 equivalents of NaOAc, formation of the C1–H products **4** occurred within 8 hours (Table 3). This transformation was applied to **3a**, **3b**, **3q**, **3r**, and **3t**, affording **4a**, **4b**, **4q**, **4r** and **4t** in isolated yields of 76–86% after purification by silica gel chromatography. This method is particularly attractive because it is transition metal-free and can be set up and run under air. Notably, under these microwave conditions, starting material **1a** remains mostly unchanged (*ca.* 10% decarboxylation), which indicated that substitution at the B2–6 positions facilitates the process, likely by stabilizing the intermediate formal C1 *exo*-carbanion.15*e* The molecular structure of **4b** was determined by X-ray crystallography, and an ORTEP representation is displayed in Fig. 2b.

**Table 3:** Decarboxylation of **3**^*a*^ ^,^^*b*^

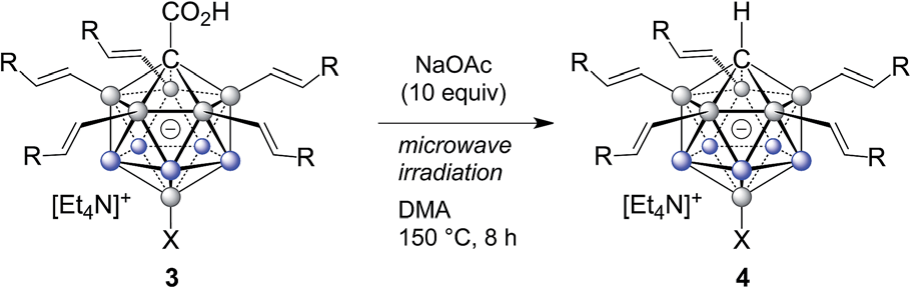			
**3a**	X = H	R = C_6_H_4_-4-F	**4a** 86%
**3b**	X = H	R = C_6_H_5_	**4b** 79%
**3q**	X = Me	R = C_6_H_4_-4-F	**4q** 87%
**3r**	X = Ph	R = C_6_H_5_	**4r** 76%
**3t**	X = CN	R = C_6_H_4_-4-F	**4t** 80%

^*a*^ **3** (0.1 mmol), NaOAc (1 mmol), in dimethylacetamide (4 mL) in a sealed microwave vial; for further details, see the ESI.

^*b*^Yields = isolated yields after purification by silica gel chromatography.

**Scheme 1:**
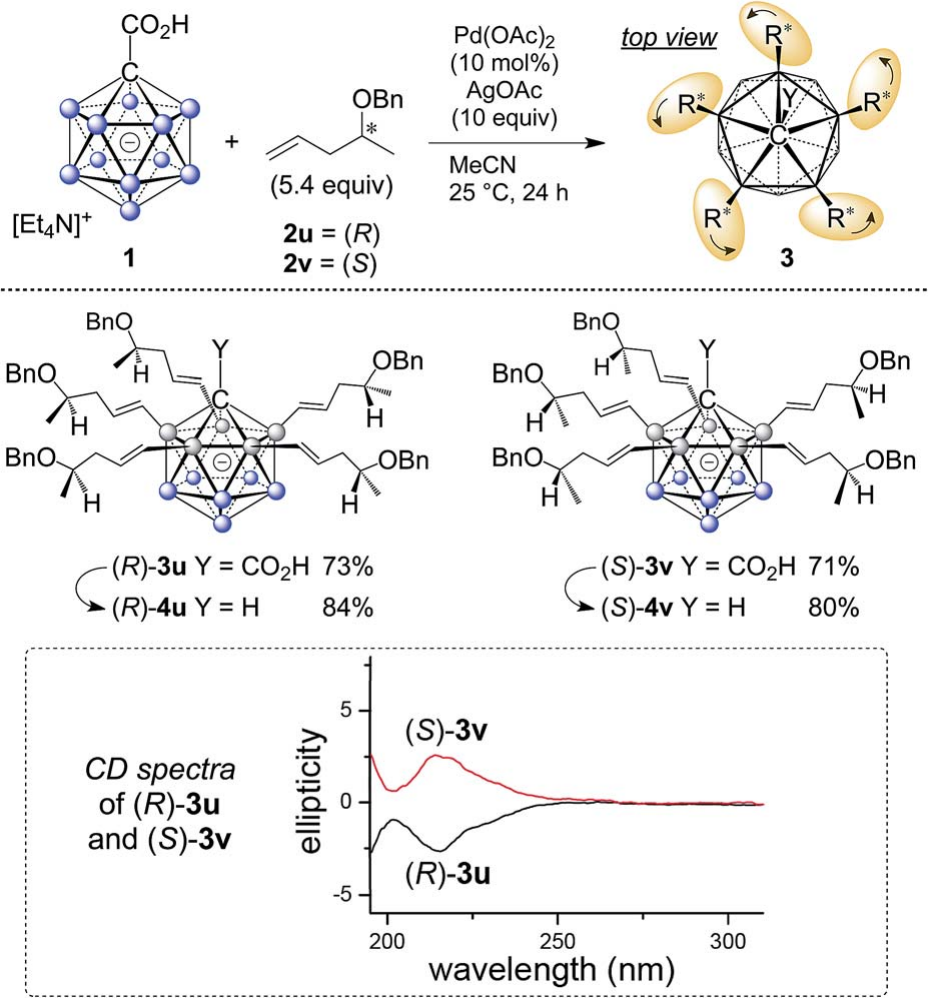
Synthesis of chiral **3u**/**v** and **4u**/**v**; curved arrows around R* indicate identical sense of absolute configuration.

The five groups at B2–6 are oriented in the direction of the B12–C1 axis and thus define a large *C*_5_-symmetrical cone. If five chiral substituents with identical absolute stereochemistry are attached, a chiral “unidirectional” cone results (Fig. 1). Such compounds have the potential to serve as ligands for enantiodiscriminative analysis, the build-up of chiral supramolecular structures and enantioselective transformations within the chiral space. To demonstrate the feasibility of the formation of optically active products, an optically pure chiral ether was chosen as the coupling partner, namely, the benzyl ether of pent-4-en-2-ol (Scheme 1). The enantiopure ethers **2u** and **2v** were prepared starting from commercially available (*R*)- and (*S*)-pent-4-en-2-ol. By applying our methodology for penta-alkenylation, desired products **3u** and **3v** were obtained in yields of 73% and 71%. Subsequent removal of the directing group afforded decarboxylated **4u** and **4v** in 84% and 80% isolated yields. The two enantiomers **3u** and **3v** were characterized by circular dichroism (CD) spectroscopy. The mirror-imaged CD spectra featured maxima at 215 nm, and no strong bands were observed above 225 nm. Given the negative charge of **3** and **4** and the ability for hydrogen bonding of the carboxylic acid unit of **3**, these novel chiral clusters are promising candidates for the above-mentioned applications, especially for enantioselective recognition and reactions involving cationic guests.

**Scheme 2:**
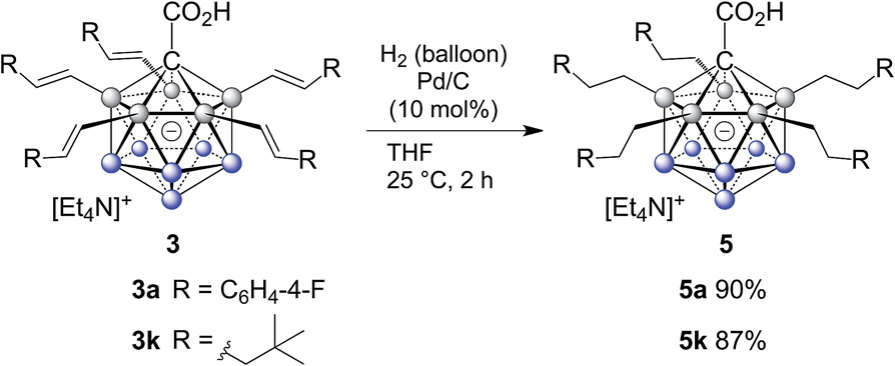
Reduction of the double bond.

The efficient and selective introduction of C(sp^3^) substituents to {CB_11_} clusters has been an long-standing synthetic challenge.^9^ To further demonstrate the synthetic utility of our B–H functionalization methodology, we examined the hydrogenation of alkenylated products. Treatment of **3a** and **3k** under hydrogen atmosphere with Pd/C as the catalyst afforded the corresponding products **5a** and **5k**, which were conveniently purified by column chromatography and isolated in 90% and 87% yields. Remarkably, complete reduction of all five double bonds occured within two hours at room temperature without the need for high pressure (hydrogen balloon). On the one hand, access to carboranes bearing multiple fully reduced substituents is important from a fundamental perspective because it rounds off the synthetic palette; on the other hand, it becomes relevant in the context of modulating physical properties, such as solubility in solvents of low polarity, redox behavior and melting point as well as applications associated with them (Scheme 2).

**Fig. 3:**
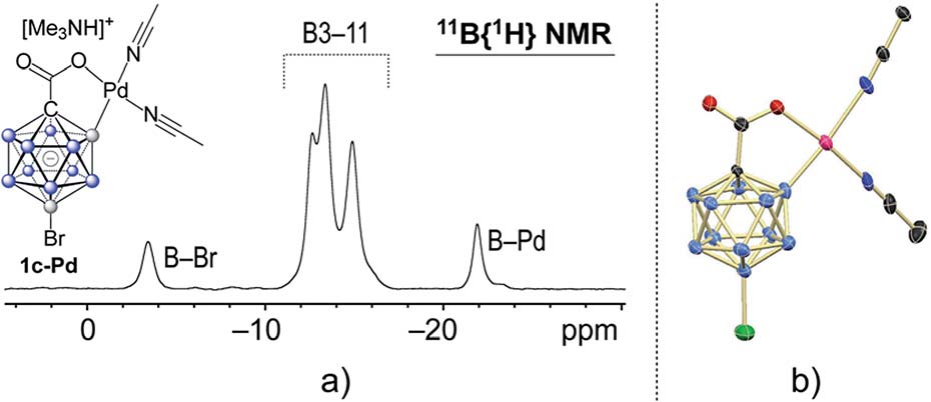
(a) ^11^B{^1^H} NMR spectrum and (b) X-ray crystal structure of palladium complex **1c–Pd** (cation and H atoms omitted for clarity; 30% displacement ellipsoids).

In a stoichiometric experiment between **1c** and Pd(OAc)_2_ in acetonitrile, cyclometaled complex **1c–Pd** formed cleanly and was isolated in 82% yield (see the ESI for details†). Its ^11^B NMR spectrum contained diagnostic signals at –21.9 and –3.5 ppm for the B2–Pd and B12–Br positions, respectively (Fig. 2a).4h,15e,^18^ All other boron vertices overlapped and resonated in the range of –10.5 to –17.5 ppm. Furthermore, the solid-state structure of **1c–Pd** was elucidated by X-ray crystallography (Fig. 3b). The Pd–B and Pd–O distances are 2.010(14) and 2.059(8) Å, and the structural features are comparable to those of recently reported monocarborane–palladium complexes.4h,15e,^18^ Treatment of **1c–Pd** with 6.0 equivalents of 4-fluorostyrene and 10 equivalents of AgOAc in acetonitrile cleanly afforded penta-substituted **3p**. Furthermore, a control experiment without the addition of AgOAc was carried out. The reaction of **1c–Pd** with 6.0 equivalents of 4-fluorostyrene in acetonitrile-*d*_3_ cleanly afforded mono-substituted product **3p-mono** within 1 hour at 25 °C, as evidenced by NMR spectroscopy and mass spectrometry (see the ESI for details†). The spectra remained unchanged after 3, 5 and 12 hours of total reaction time, *i.e.*, no products with higher degree of substitution were formed. These results suggest that the palladium complex **1c–Pd** is an intermediate relevant to the catalytic cycle and that AgOAc is necessary for catalytic turnover.

**Scheme 3:**
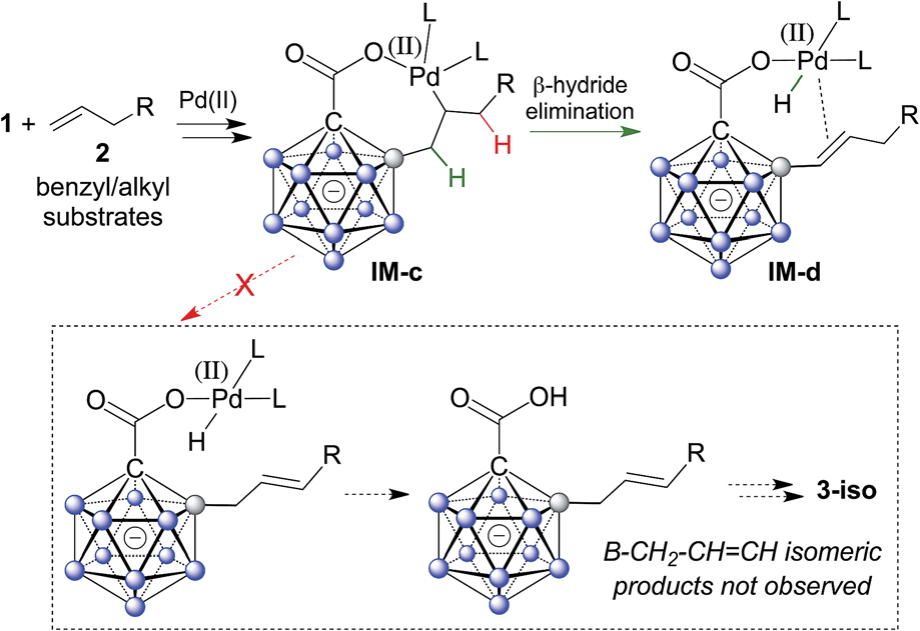
Putative selective β-hydride elimination to explain the regioselectivity of double bond formation with benzyl and alkyl substrates.

A plausible mechanistic cycle involving five sequential B–H bond activation/B–C coupling events is displayed in Scheme S1.† Carborane acid **1** can bind to Pd(ii) *via* its carboxylate group, affording the initial intermediate **IM-a**. Cyclometalation–deprotonation then gives palladacycle **1–Pd** with a direct B–Pd bond. Alkene coordination/insertion (intermediates **IM-b**/**IM-c**) is followed by β-hydride elimination, furnishing Pd–H complex **IM-d**. Subsequent reaction with AgOAc leads to **IM-a′**, which contains one alkenyl substituent. From this point on, further B–H activation/alkenylation steps occur in a similar manner until the final product **3** and Pd(ii) are liberated. The use of benzyl and alkyl substrates **2** in principle allows for the occurrence of isomeric products with a B–CH_2_–CH=CH bonding pattern (Scheme 3). It is remarkable that there is no indication of the formation of such isomers (analysis of crude NMR spectra). The regioselective β-hydride elimination may be explained by stabilization of **IM-d***via* π_alkene_ → Pd coordination, which we believe would be weaker in the case of the alternative pathway.

## Conclusions

In conclusion, we have developed a mild and versatile methodology for the regioselective B2–6 cage alkenylation of monocarborane anions through a palladium-catalyzed B–H/C–H cross coupling cascade. Styrenes and benzylic as well as aliphatic alkenes serve as efficient coupling partners, providing access to a variety of penta-substituted products in moderate to high yields. The carboxylic acid directing group can be readily removed without the need for transition metals. Furthermore, the introduction of homochiral substituents and the possibility to reduce the double bond allows for the preparation of novel chiral and selectively alkylated monocarboranes. Therefore, we believe this methodology can be of broad utility for the construction of new classes of carborane-based compounds, which will be beneficial for synthetic chemists and also researchers in multiple areas of applications.

## Conflicts of interest

There are no conflicts to declare.

## Supplementary Material

Supplementary information

Crystal structure data
