# Supplementary material for: Highly selective palladium-catalyzed one-pot, five-fold B–H/C–H cross coupling of monocarboranes with alkenes
Source: Chem Sci. 2019 Mar 4;10(15):4177–84. doi: 10.1039/c9sc00078j (PMC6471670; doi:10.1039/c9sc00078j)
Supplement: Supplementary file 1 [file SC-010-C9SC00078J-s001.pdf]

Electronic Supplementary Material (ESI) for Chemical Science.
This journal is © The Royal Society of Chemistry 2019

Supplementary Information for

Palladium-Catalyzed Penta-Functionalization of Monocarboranes with Alkenes *via* Regioselective B−H/C–H Bond Coupling

Yunjun Shen,[a] Kang Zhang,[a] Xuewei Liang,[a] Rakesh Dontha,[a] and Simon Duttwyler*[a]

[a]Department of Chemistry, Zhejiang University,
38 Zheda Road, 310027 Hangzhou, PR China

*E-mail address: duttwyler@zju.edu.cn

Table of Contents

**I**	**General Information**	**p. S2–S3**
**II**	**Experimental Section**	**p. S4–S48**
**III**	**X-ray Crystallography**	**p. S49–S54**
**IV**	**References**	**p. S55**
**V**	**NMR spectra**	**p. S56–S201**
**VI**	**Mass spectra**	**p. S202–S236**

I General Information

Chemicals

If not otherwise specified, reagents and organic solvents were commercially available and used without further purification. Acetone-d_6 and acetonitrile-d_3 were purchased from Cambridge Isotope Laboratories and filtered through Al_2O_3 prior to use. Anhydrous solvents were prepared by passage through activated Al_2O_3 and stored over 3 Å molecular sieves. Previously reported carborane anions $[CB_{11}H_{12}]^-$, $[1\text{-}(CO_2H)\text{-}CB_{11}H_{11}]^-$, $[1\text{-}(CO_2H)\text{-}CB_{11}H_{10}\text{-}12\text{-}Br]^-$, $[CB_{11}H_{11}\text{-}12\text{-}Cl]^-$, $[CB_{11}H_{11}\text{-}12\text{-}CH_3]^-$, $[CB_{11}H_{11}\text{-}12\text{-}Ph]^-$, $[1\text{-}(CO_2H)\text{-}CB_{11}H_{10}\text{-}12\text{-}CN]^-$ and **2u/v** were prepared according to the literature.[1-8]

Reaction Conditions

Glassware for air-sensitive reactions was dried at 150 °C for 12 h and allowed to cool in a vacuum.

Characterization

Thin-layer chromatography (TLC) was carried out using silica gel 60, F254 with a thickness of 0.25 mm. Column chromatography was accomplished on silica gel 60 (200-300 mesh).

NMR spectra were recorded on a Bruker AVANCE III 500 spectrometer (^{1}H NMR 500.13 MHz, ^{13}C NMR 125.77 MHz, ^{11}B NMR 160.46 MHz) or a Bruker AVANCE III 400 spectrometer (^{1}H NMR 400.13 MHz, ^{13}C NMR 100.62 MHz, ^{11}B NMR 128.38 MHz) at 23 °C. Chemical shifts are given in ppm. ^{1}H NMR and ^{13}C NMR spectra were referenced using the solvent signals (^{1}H: residual $C\mathit{H}D_2C(O)CD_3$ = 2.05 ppm, residual CHD_2CN = 1.94 ppm, $^{13}C\{^1H\}$: $\mathit{C}D_3C(O)\mathit{C}D_3$ = 29.84 ppm, $\mathit{C}D_3CN$ = 1.32 ppm). ^{11}B and $^{11}B\{^1H\}$ NMR spectra were calibrated against external $BF_3{*}Et_2O$ = 0 ppm ($BF_3{*}Et_2O$ capillary in C_6D_6). Data are reported as follows: Chemical shift in ppm, multiplicity (s = singlet, d = doublet, t = triplet, q = quartet, m

= multiplet, dd = doublet of doublets, etc.), coupling constant *J* in Hz, integration, and (where applicable) interpretation.

General notes:

- The CH_3 group of the $[Et_4N]^+$ cation showed $^{3}J_{1H,14N}$ coupling to the central nitrogen atom, and therefore the signal appeared as a triplet of triplets ($^{3}J_{1H,1H}$ and $^{3}J_{1H,14N}$). Generally speaking, coupling to ^{14}N is sometimes observed in highly symmetrically coordinated nitrogen compounds; the coupling constant is not uniformly related to the distance to the nitrogen atom.
- In certain ^{1}H and $^{1}H\{^{11}B\}$ NMR spectra measured in acetone-d_6, double water peaks were observed. This is a result of different resonances from H_2O and HOD and has been described in the literature.[9]

Low-resolution ESI-MS data were recorded on Advion Expression CMS instrument. High-resolution MS data were recorded using IT-TOF detection (Shimadzu, Japan) equipped with an electrospray ionization source (ESI). Accurate mass determination was corrected by calibration using sodium trifluoroacetate clusters as a reference.

Single-crystal X-ray diffraction studies were performed on an Oxford Diffraction Gemini A Ultra diffractometer equipped with 135 mm Atlas CCD detector and using Mo or Cu X-ray sources or on a Bruker D8 Venture instrument with Ga wavelength.

Elemental analysis was carried out by the analytical facilities of the Department of Chemistry at Zhejiang University. Three representative samples were measured in order to confirm that bulk purity by spectroscopic methods corresponded to bulk purity by elemental analysis:

3o: Analysis calculated for $[Et_4N][C_{42}H_{41}B_{11}ClO_2]$: C, 69.64; H, 7.13; N, 1.62; found: C, 69.44; H, 7.19; N, 1.53.

3r: Analysis calculated for $[Et_4N][C_{48}H_{46}B_{11}O_2]$: C, 74.40; H, 7.36; N, 1.55; found: C, 74.10; H, 7.46; N, 1.47.

4r: Analysis calculated for $[Et_4N][C_{47}H_{46}B_{11}]$: C, 76.81; H, 7.73; N, 1.63; found: C, 76.22; H, 7.92; N, 1.55.

II Experimental Section

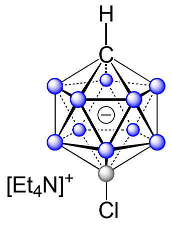

[Et_4N][$CB_{11}H_{11}$-12-Cl]: This carborane anion has been reported before.[5] A modified procedure is given below. The spectroscopic data match with those reported.

To a stirred solution of [Cs][$CB_{11}H_{12}$] (200 mg, 0.72 mmol) in CH_3OH (12 mL) was added *N*-chlorosuccinimide (105 mg, 0.79 mmol). The reaction mixture was then stirred at 40 °C for 24 h while being monitored by (–)-ESI mass spectrometry. Na_2SO_3 (19 mg, 0.15 mmol) was added to the mixture, followed by 1 M HCl (30 mL). Methanol was removed under reduced pressure. The aqueous solution was extracted with Et_2O (3 x 35 mL). Water (15 ml) was added to the combined extracts, and Et_2O was removed under reduced pressure. The aqueous solution was filtered, and [Et_4N]Br (227 mg, 1.08 mmol) was added to the filtrate. The precipitate that formed was collected by vacuum filtration, washed with water and dried under vacuum to give a colorless solid (157 mg, 71%).

$^{1}H\{^{11}B\}$ NMR (400 MHz, acetone-d_6, 23 °C): δ 3.48 (q, J = 7.3 Hz, 8H, CH_2 of cation), 2.19-2.07 (m, 1H, CH), 2.02-1.70 (broad signal, 5H, BH), 1.69-1.47 (broad signal, 5H, BH), 1.39 (tt, J = 7.3Hz, 1.8 Hz, 12H, CH_3 of cation).
$^{11}B\{^{1}H\}$ NMR (128 MHz, acetone-d_6, 23 °C): δ 3.78 (1B), -12.75 (5B). -17.55 (5B).
$^{13}C\{^{1}H\}$ NMR (101 MHz, acetone-d_6, 23 °C): δ 52.99 (CH_2 of cation), 42.63 (cage C), 7.66 (CH_3 of cation).

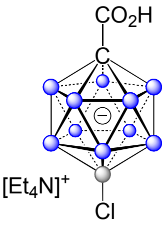

$[Et_4N]^+$

1b: A dry 100 mL round bottom flask equipped with magnetic stir bar was charged with $[Et_4N][CB_{11}H_{11}\text{-12-Cl}]$ (600 mg, 1.95 mmol) and capped with a rubber septum. Anhydrous THF (25 mL) was then added to the flask, and the resulting solution was cooled to 0 °C in an ice bath. A solution of *n*-BuLi (2.5 M in hexane, 2.4 mL, 6 mmol) was slowly added. After 1 h of stirring at 0 °C, a slightly turbid, yellowish solution was obtained. Dry CO_2 gas was then bubbled through the mixture at 0 °C (2-3 bubbles/s) for 5 h. Water (2 mL) was slowly added, and the solution was concentrated on a rotary evaporator. The residue was dissolved in water and washed with diethyl ether (under these basic conditions the dianionic product was in the water layer). The aqueous phase was acidified with 1 M HCl (pH = 2) and extracted with diethyl ether (3 x 40 mL). The combined organic extracts were evaporated under reduced pressure, and the crude product was dissolved in water (10 mL) and filtered through a glass frit. $[Et_4N]Br$ (630 mg, 3 mmol) was added to the filtrate, and the resulting white precipitate was collected in a glass frit and dried in a vacuum to give **1b** (556 mg, 81% yield).

$^1H\{^{11}B\}$ NMR (500 MHz, acetone-d_6, 23 °C): δ 3.48 (q, J = 7.3 Hz, 8H, CH_2 of cation), 1.94 (broad signal, 5H, BH), 1.86 (broad signal, 5H, BH), 1.40 (tt, J = 7.3Hz, 1.9 Hz, 12H, CH_3 of cation).

$^{11}B\{^1H\}$ NMR (160 MHz, acetone-d_6, 23 °C): δ 5.12 (1B), -12.63 (5B), -15.11 (5B).

$^{13}C\{^1H\}$ NMR (125 MHz, acetone-d_6, 23 °C): δ 168.57 (CO), 60.77 (cage C), 53.02 (CH_2 of cation), 7.66 (CH_3 of cation).

HRMS (ESI): *m/z* Calcd. for $[C_2H_{11}B_{11}ClO_2]^-$, 221.1544; found, 221.1542.

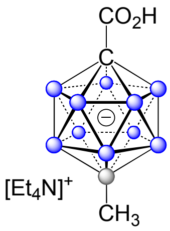

1d: Following a similar procedure as for the preparation of **1b,** using $[Et_4N][CB_{11}H_{11}\text{-}12\text{-}Me]$ (500 mg, 1.74 mmol) as starting material, **1d** was obtained as a colorless solid (438 mg, 76%).

$^1H\{^{11}B\}$ NMR (500 MHz, acetone-d_6, 23 °C): δ 3.48 (q, J = 7.3 Hz, 8H, CH_2 of cation), 1.94 (broad signal, 5H, BH), 1.62 (broad signal, 5H, BH), 1.40 (tt, J = 7.3Hz, 1.9 Hz, 12H, CH_3 of cation), 0.00 (s, 3H, CH_3).

$^{11}B\{^1H\}$ NMR (160 MHz, acetone-d_6, 23 °C): δ 2.45 (s, 1B), -11.83 (s, 5B), -14.26 (s, 5B).

$^{13}C\{^1H\}$ NMR (125 MHz, acetone-d_6, 23 °C): δ 168.95 (CO), 62.32 (cage C), 53.00 (CH_2 of cation), 7.67 (CH_3 of cation). The CH_3 group appeared as a broad and weak signal at 5.5 ppm.

HRMS (ESI): *m/z* Calcd. for $[C_3H_{14}B_{11}O_2]^-$, 201.2090; found, 201.2093.

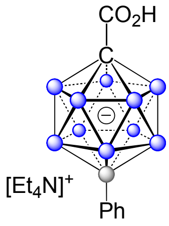

1e: Following a similar procedure as for the preparation of **1b,** using $[Et_4N][CB_{11}H_{11}\text{-}12\text{-}Ph]$ (500 mg, 1.43 mmol) as starting material, **1e** was obtained as a colorless solid (388 mg, 69%).

$^1H\{^{11}B\}$ NMR (400 MHz, acetone-d_6, 23 °C): δ 7.42-7.32 (m, 2H, ArH), 7.05-6.90 (overlapping m, 3H, ArH), 3.43 (q, J = 7.3 Hz, 8H, CH_2 of cation), 2.04 (broad signal overlapping with solvent residual signal, 5H, BH), 1.85 (broad signal, 5H, BH), 1.35 (tt, J = 7.3Hz, 1.9 Hz, 12H, CH_3 of cation).

$^{11}B\{^1H\}$ NMR (128 MHz, acetone-d_6, 23 °C): δ 3.20 (s, 1B), -12.39 (s, , 5B), -14.20 (s, 5B).

$^{13}C\{^1H\}$ NMR (101 MHz, acetone-d_6, 23 °C): δ 168.75 (CO), 133.56, 126.89, 125.41 (aryl C), 64.64 (cage C), 52.96 (CH_2 of cation), 7.64 (CH_3 of cation). The B–C(*ipso*) signal could not be detected unambiguously.

HRMS (ESI): *m/z* Calcd. for $[C_8H_{16}B_{11}O_2]^-$, 263.2247; found, 263.2244.

General Procedure A for the palladium-catalyzed penta-alkenylation

To a 10 mL glass vial equipped with a magnetic stir bar, carborane carboxylic acid **1** (0.150 mmol), $Pd(OAc)_2$ (0.015 mmol), AgOAc (1.5 mmol) and dry acetonitrile (4 mL) were added. To the stirring mixture the required alkene **2** (0.81 mmol) was added, and the resulting mixture was stirred for 24 h at 25 °C. The mixture was filtered through celite, and the filtrate was treated with $[Et_4N]Br$ (5 equiv). The white precipitate that formed was removed by filtration, and the filtrate was then evaporated to dryness under reduced pressure. Purification was done by chromatography on silica gel by eluting with CH_2Cl_2 to afford the desired product **3**.

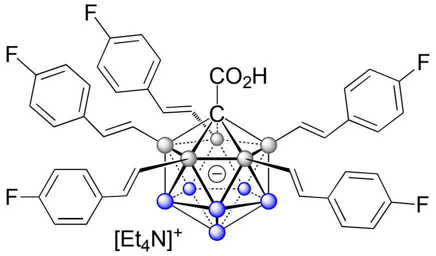

3a: Prepared following the general procedure A, using **1a** and 4-fluorostyrene, **3a** was obtained as a colorless solid (118 mg, 86%).

$^1H\{^{11}B\}$ NMR (400 MHz, acetone-d_6, 23 °C): δ 7.46-7.31 (m, 10H, ArH), 7.12-6.98 (m, 10H, ArH), 6.94 (d, J = 18.0 Hz, 5H, alkenyl CH), 6.66 (d, J = 18.0 Hz, 5H, alkenyl CH), 3.32 (q, J = 7.3 Hz, 8H, CH_2 of cation), 2.48-1.56 (overlapping broad signals, 6H, BH), 1.29 (tt, J = 7.3 Hz, 1.6 Hz, 12H, CH_3 of cation).
$^{11}B\{^1H\}$ NMR (128 MHz, acetone-d_6, 23 °C): δ ca. -2.02 to -9.83 (overlapping signals, 6B), -12.90 (overlapping signals, 5B).
$^{13}C\{^1H\}$ NMR (101 MHz, acetone-d_6, 23 °C): δ 165.75 (CO), 162.40 (d, J = 243 Hz, C-F), 138.27 (BCH*C*H), 137.55 (d, J = 3.0 Hz, C(*ipso*)), 131.66 (broad signal, BCH), 128.03 (d, J = 7.7 Hz, C(*ortho*)), 115.75 (d, J = 22.0 Hz, C(*meta*)), 68.38 (cage C), 52.88 (CH_2 of cation), 7.56 (CH_3 of cation).
HRMS (ESI): *m/z* Calcd. for $[C_{42}H_{37}B_{11}F_5O_2]^-$, 787.3810; found, 787.3833.

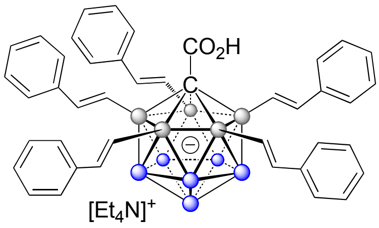

3b: Prepared following the general procedure A, using **1a** and styrene, **3b** was obtained as a colorless solid (93 mg, 75%).

^{1}H{^{11}B} NMR (500 MHz, acetone-d_6, 23 °C): δ 7.41-7.28 (m, 10H, ArH), 7.27-7.16 (m, 10H, ArH), 7.15-7.05 (m, 5H, ArH), 6.96 (d, J= 18.0 Hz, 5H, alkenyl CH), 6.73 (d, J = 18.0 Hz, 5H, alkenyl CH), 3.37 (q, J = 7.3 Hz, 8H, CH_2 of cation), 2.15-1.35 (m, 6H, BH), 1.31 (tt, J = 7.3 Hz, 1.6 Hz, 12H, CH_3 of cation).

^{11}B{^{1}H} NMR (160 MHz, acetone-d_6, 23 °C): δ ca. -3.51 to -10.11 (overlapping signals, 6B), ca. -12.95 (5B).

^{13}C{^{1}H} NMR (126 MHz, acetone-d_6, 23 °C): δ 165.83 (CO), 141.29 (aryl C), 139.58 (BCH*C*H), 132.26 (broad signal, BCH), 129.10 (aryl CH), 127.08 (aryl CH), 126.57 (aryl CH), 68.53 (cage C), 52.96 (CH_2 of cation), 7.63 (CH_3 of cation).

HRMS (ESI): m/z Calcd. for $[C_{42}H_{42}B_{11}O_2]^-$, 697.4281; found, 697.4326.

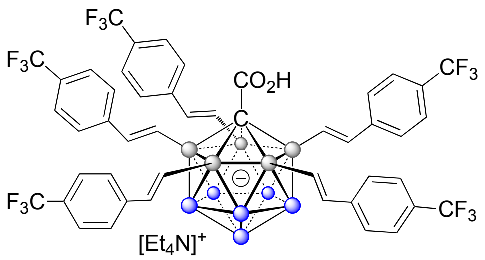

3c: Prepared following the general procedure A, using **1a** and 4-trifluoromethylstyrene, **3c** was obtained as a colorless solid (122 mg, 70%).

$^{1}H\{^{11}B\}$ NMR (500 MHz, acetone-d_6, 23 °C): δ 7.69-7.47 (overlapping m, 20H, ArH), 7.04 (d, *J*= 18.0 Hz, 5H, alkenyl CH), 6.90 (d, *J* = 18.0 Hz, 5H, alkenyl CH), 3.41 (q, *J* = 7.3 Hz, 8H, CH_2 of cation), 2.47-1.76 (overlapping broad signals, 6H, BH), 1.34 (tt, *J* = 7.3 Hz, 1.6 Hz, 12H, CH_3 of cation).

$^{11}B\{^{1}H\}$ NMR (160 MHz, acetone-d_6, 23 °C): δ ca. -2.46 to -9.26 (overlapping signals, 6B), -12.52 (5B).

$^{13}C\{^{1}H\}$ NMR (125 MHz, acetone-d_6, 23 °C): δ 165.67 (CO), 144.67 (C(*ipso*)), 138.57 (BCH*C*H), 135.13 (broad signal, B*C*HCH), 128.50 (q, *J* = 32 Hz, C(*para*)), 126.97 (C(*ortho*)), 126.19 (C(*meta*)), 125.55 (q, *J* = 271 Hz, CF_3), 68.51 (cage C), 52.98 (CH_2 of cation), 7.61 (CH_3 of cation).

HRMS (ESI): *m/z* Calcd. for $[C_{47}H_{37}B_{11}F_{15}O_2]^-$, 1037.3650; found, 1037.3671.

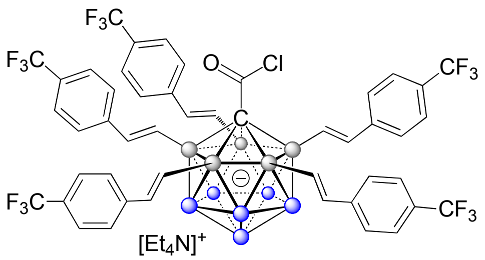

3c-Cl: To a stirred solution of **3c** (105 mg, 0.090 mmol) in dry CH_2Cl_2 (5 mL) were added dimethylformamide (4 drops) and oxalyl chloride (0.015 ml, 0.18 mmol) under nitrogen atmosphere. The reaction mixture was allowed to stir for 15 min at room temperature. All the volatiles were removed carefully under vacuum with nitrogen-cooled solvent traps. The residue was then stirred with hexane (5 mL) at 25 °C for 10 min and collected by filtration through a glass frit. After drying in a vacuum, a colorless solid was obtained and identified as acid chloride **3c-Cl** in >95% purity as evidenced by NMR spectroscopy and mass spectrometry (99 mg, 93% yield).

$^{1}H\{^{11}B\}$ NMR (500 MHz, acetonitrile-d_3, 23 °C): δ 7.65-7.48 (overlapping m, 20H, ArH), 7.04 (d, J = 18.0 Hz, 5H, alkenyl CH), 6.69 (d, J = 18.0 Hz, 5H, alkenyl CH), 3.12 (q, J = 7.3 Hz, 8H, CH_2 of cation), 2.58-1.58 (overlapping broad signals, 6H, BH), 1.18 (tt, J = 7.3 Hz, 1.6 Hz, 12H, CH_3 of cation).

$^{11}B\{^{1}H\}$ NMR (160 MHz, acetonitrile-d_3, 23 °C): δ ca. -0.80 to -9.40 (overlapping signals, 6B), -12.52 (5B).

$^{13}C\{^{1}H\}$ NMR (126 MHz, acetonitrile-d_3, 23 °C): δ 166.26 (CO), 143.96 (C(*ipso*)), 140.06 (BCH*C*H), 132.47 (broad signal, B*C*HCH), 128.98 (q, J = 32 Hz, C(*para*)), 127.20 (C(*ortho*)), 126.38 (C(*meta*)), 125.55 (q, J = 270 Hz, CF_3), 74.90 (cage C), 52.99 (CH_2 of cation), 7.60 (CH_3 of cation).

HRMS (ESI): *m/z* Calcd. for $[C_{47}H_{36}B_{11}ClF_{15}O]^-$, 1055.3311; found, 1055.3348.

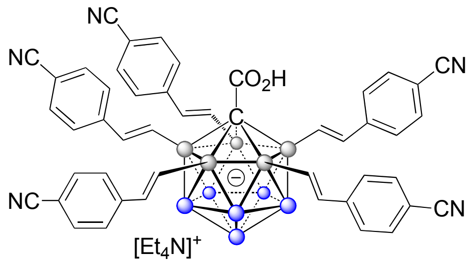

3d: Prepared following the general procedure A, using **1a** and 4-cyanostyrene, **3d** was obtained as a colorless solid (90 mg, 63%).

$^{1}H\{^{11}B\}$ NMR (400 MHz, acetone-d_6, 23 °C): δ 7.70-7.57 (m, 10H, ArH), 7.56-7.45(m, 10H, ArH), 6.97 (d, J = 18.0 Hz, 5H, alkenyl CH), 6.87 (d, J = 18.0 Hz, 5H, alkenyl CH), 3.48 (q, J = 7.3 Hz, 8H, CH_2 of cation), 2.29-1.84 (overlapping broad signals, 6H, BH), 1.39 (tt, J = 7.3 Hz, 1.6 Hz, 12H, CH_3 of cation).

$^{11}B\{^{1}H\}$ NMR (128 MHz, acetone-d_6, 23 °C): δ ca. -3.11 to -9.13 (overlapping signals, 6B), -12.50 (5B).

$^{13}C\{^{1}H\}$ NMR (100 MHz, acetone-d_6, 23 °C): δ 165.55 (CO), 145.07 (aryl C), 138.59 (BCH*C*H), 136.13 (broad signal, B*C*HCH), 133.17, 127.23 (aryl C), 119.67 (CN), 110.37 (aryl C), 68.45 (cage C), 52.96 (CH_2 of cation), 7.63 (CH_3 of cation).

HRMS (ESI): *m/z* Calcd. for $[C_{47}H_{37}B_{11}N_5O_2]^-$, 822.4043; found, 822.4064.

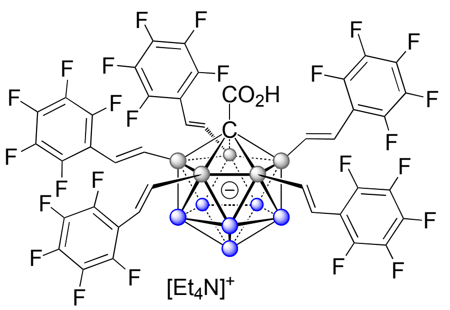

3e: Prepared following the general procedure A, using **1a** and pentafluorostyrene, **3e** was obtained as a colorless solid (102 mg, 53%).

$^{1}H\{^{11}B\}$ NMR (500 MHz, acetone-d_6, 23 °C): δ 6.98 (d, J = 18.6 Hz, 5H, alkenyl CH), 6.83 (d, J = 18.6 Hz, 5H, alkenyl CH), 3.51 (q, J = 7.3 Hz, 8H, CH_2 of cation), 2.49-2.05 (overlapping broad signals, 6H, BH), 1.41 (tt, J = 7.3 Hz, 1.6 Hz, 12H, CH_3 of cation).

$^{11}B\{^{1}H\}$ NMR (160 MHz, acetone-d_6, 23 °C): δ ca. -3.90 to -9.30 (overlapping signals, 6B), -12.41 (5B).

$^{13}C\{^{1}H\}$ NMR (126 MHz, acetone-d_6, 23 °C): δ 164.97 (CO), 145.51 (m with $^{1}J_{C\text{-}F}$ = 248 Hz, aryl C), 142.42 (broad signal, B*C*HCH), 140.08 (m with $^{1}J_{C\text{-}F}$ = 250 Hz, C(*para*)), 138.57 (m with $^{1}J_{C\text{-}F}$ = 246 Hz, aryl C), 123.55 (BCH*C*H), 115.40 (t, J = 13 Hz, C(*ipso*)), 68.36 (cage C), 53.05 (CH_2 of cation), 7.66 (CH_3 of cation).

HRMS (ESI): *m/z* Calcd. for $[C_{42}H_{17}B_{11}F_{25}O_2]^-$, 1147.1926; found, 1147.1946.

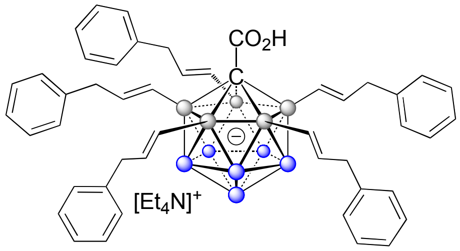

3f: Prepared following the general procedure A, using **1a** and allylbenzene, **3f** was obtained as a colorless solid (97 mg, 72%).

$^{1}H\{^{11}B\}$ NMR (400 MHz, acetone-d_6, 23 °C): δ 7.35-7.17 (overlapping m, 20H, ArH), 7.16-7.07 (m, 5H, ArH), 6.18-5.97 (m, 5H, alkenyl CH), 5.96-5.78 (m, 5H, alkenyl CH), 3.57-3.17 (overlapping signals, 18H, CH_2 of cation and $PhCH_2$), 2.00-1.61 (broad overlapping signals, 6H, BH), 1.30 (tt, J = 7.3 Hz, 1.6 Hz, 12H, CH_3 of cation).
$^{11}B\{^{1}H\}$ NMR (160 MHz, acetone-d_6, 23 °C): δ ca. -1.41 to -10.05 (overlapping signals, 6B), -13.51 (5B).
$^{13}C\{^{1}H\}$ NMR (126 MHz, acetone-d_6, 23 °C): δ 165.46 (CO), 142.74 (aryl C), 138.97 (BCH*C*H), 133.33 (broad signal, BCH), 129.43 (aryl CH), 128.86 (aryl CH), 126.22 (aryl CH), 68.30 (cage C), 52.96 (CH_2 of cation), 43.36 ($PhCH_2$), 7.64 (CH_3 of cation).
HRMS (ESI): m/z Calcd. for $[C_{47}H_{52}B_{11}O_2]^-$, 767.5064; found, 767.5081.

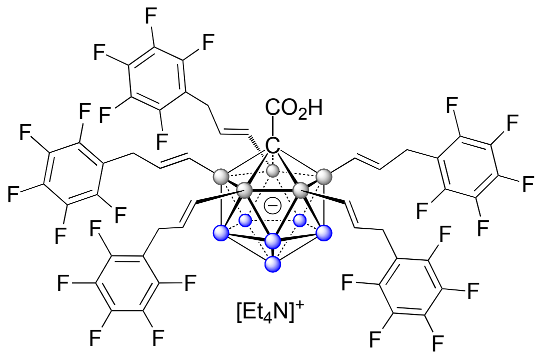

3g: Prepared following the general procedure A, using **1a** and allylpentafluorobenzene, **3g** was obtained as a colorless solid (119 mg, 59%).

$^{1}H\{^{11}B\}$ NMR (400 MHz, acetone-d_6, 23 °C): δ 5.88-5.64 (m, 5H, alkenyl CH), 5.48 (d, J = 17.3 Hz, 5H, alkenyl CH), 3.49 (q, J = 7.3 Hz, 8H, CH_2 of cation), 3.31 (d, J = 5.4 Hz, 10H, CH_2), 2.02-1.45 (overlapping broad signals, 6H, BH), 1.39 (tt, J = 7.3 Hz, 1.6 Hz, 12H, CH_3 of cation).
$^{11}B\{^{1}H\}$ NMR (128 MHz, acetone-d_6, 23 °C): δ ca. -3.82 to -10.32 (overlapping signals, 6B), ca. -13.71 (5B).
$^{13}C\{^{1}H\}$ NMR (101 MHz, acetone-d_6, 23 °C): δ 164.92 (CO), 145.79 (m with $^{1}J_{C\text{-}F}$ = 244 Hz, aryl C), 140.18 (m with $^{1}J_{C\text{-}F}$ = 248 Hz, C(*para*)), 138.18 (m with $^{1}J_{C\text{-}F}$ = 248 Hz, aryl C), 133.81 (one sharp and one broad signal, overlapping, B*C*HCH and BCH*C*H), 115.71 (t, J = 19 Hz, C(*ipso*)), 67.93 (cage C), 53.01 (CH_2 of cation), 28.63 (CH_2), 7.64 (CH_3 of cation).
HRMS (ESI): *m/z* Calcd. for $[C_{47}H_{27}B_{11}F_{25}O_2]^-$, 1217.2708; found, 1217.2728.

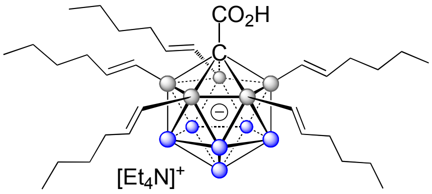

3h: Prepared following the general procedure A, using **1a** and 1-hexene, **3h** was obtained as a colorless solid (88 mg, 81%).

$^1H\{^{11}B\}$ NMR (500 MHz, acetone-d_6, 23 °C): δ 6.00-5.79 (m, 5H, alkenyl CH), 5.60 (d, J = 17.40 Hz, 5H, alkenyl CH), 3.47 (q, J = 7.3 Hz, 8H, CH_2 of cation), 2.05-1.89 (m, 10H, $CH_3CH_2CH_2C\mathit{H_2}$) 1.84-1.53 (overlapping broad signals, 6H, BH), 1.39 (tt, J = 7.3 Hz, 1.6 Hz, 12H, CH_3 of cation), 1.36-1.22 (overlapping m, 20H, $CH_3C\mathit{H_2}C\mathit{H_2}$), 1.00-0.75 (m, 15H, $C\mathit{H_3}CH_2$).

$^{11}B\{^1H\}$ NMR (160 MHz, acetone-d_6, 23 °C): δ ca. -3.63 to -10.17 (overlapping signals, 6B), -13.68 (5B).

$^{13}C\{^1H\}$ NMR (126 MHz, acetone-d_6, 23 °C): δ 164.42 (CO), 141.48 (BCH*C*H), 130.94 (broad signal, B*C*HCH), 68.77 (cage C), 53.02 (CH_2 of cation), 36.49 ($CH_3CH_2CH_2\mathit{C}H_2$), 32.57 ($CH_3CH_2\mathit{C}H_2$), 22.76 ($CH_3\mathit{C}H_2$), 14.34 ($\mathit{C}H_3CH_2$), 7.70 ($CH_3$ of cation).

HRMS (ESI): *m/z* Calcd. for $[C_{32}H_{62}B_{11}O_2]^-$, 597.5846; found, 597.5843.

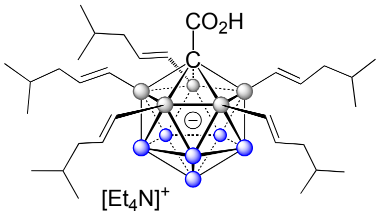

3i: Prepared following the general procedure A, using **1a** and 4-methyl-1-pentene, **3i** was obtained as a colorless solid (92 mg, 84%).

$^{1}H\{^{11}B\}$ NMR (500 MHz, acetone-d_6, 23 °C): δ 5.95-5.84 (m, 5H, alkenyl CH), 5.62 (d, J = 17.40 Hz, 5H, alkenyl CH), 3.48 (q, J = 7.3 Hz, 8H, CH_2 of cation), 1.95-1.85 (m, 10H, CH_2), 1.84-1.64 (overlapping broad signals, 6H, BH), 1.63-1.52 (m, 5H, $CH_3)_2CH$), 1.39 (tt, J = 7.3 Hz, 1.6 Hz, 12H, CH_3 of cation), 0.94-0.81 (m, 30H, $(CH_3)_2CH$).

$^{11}B\{^{1}H\}$ NMR (160 MHz, acetone-d_6, 23 °C): δ ca. -3.64 to -10.39 (overlapping signals, 6B), -13.56 (5B).

$^{13}C\{^{1}H\}$ NMR (101 MHz, acetone-d_6, 23 °C): δ 164.57 (CO), 140.09 (BCHCH), 132.74 (broad signal, BCHCH), 68.53 (cage C), 53.00 (CH_2 of cation), 46.52 (CH_2), 29.34 $(CH_3)_2CH$), 22.80 $(CH_3)_2CH$), 7.68 (CH_3 of cation).

HRMS (ESI): *m/z* Calcd. for $[C_{32}H_{62}B_{11}O_2]^-$, 597.5846; found, 597.5849.

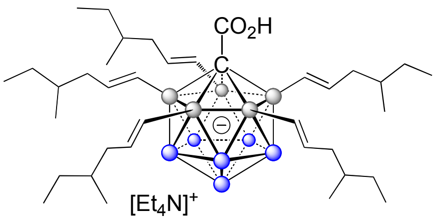

3j: Prepared following the general procedure A, using **1a** and (±)-4-methyl-1-hexene, **3j** was obtained as a colorless solid (98 mg, 82%).

$^{1}H\{^{11}B\}$ NMR (500 MHz, acetone-d_6, 23 °C): δ 6.00-5.79 (m, 5H, alkenyl CH), 5.63 (d, J = 17.40 Hz, 5H, alkenyl CH), 3.48 (q, J = 7.3 Hz, 8H, CH_2 of cation), 2.05-1.96 (m, 5H, BCHCHCH_2), 1.92-1.80 (m, 5H, BCHCHCH_2), 1.79-1.53 (broad overlapping signals, 6H, BH), 1.48-1.33 (overlapping m, 22H, CH_3CH_2 and CH_3 of cation), 1.20-1.05 (m, 5H, CH_3CH), 0.92-0.78 (overlapping m, 30H, CH_3).
$^{11}B\{^{1}H\}$ NMR (160 MHz, acetone-d_6, 23 °C): δ ca. -3.66 to -10.58 (overlapping signals, 6B), -13.18 (5B).
$^{13}C\{^{1}H\}$ NMR (126 MHz, acetone-d_6, 23 °C): δ 164.52 (CO), 139.96 (BCH*C*H), 132.62 (broad signal, B*C*HCH), 68.60 (cage C), 53.03 (CH_2 of cation), 44.21 (BCHCH*C*H_2), 35.87 (CH_3*C*H), 19.62 (*C*H_3CH), 11.88 (*C*H_3CH_2), 7.69 (CH_3 of cation). One CH_2 signal could not be detected unambiguously because of overlap with the solvent signal.
HRMS (ESI): *m/z* Calcd. for $[C_{37}H_{72}B_{11}O_2]^-$, 667.6629; found, 667.6634.

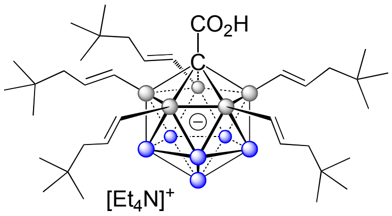

3k: Prepared following the general procedure A, using **1a** and 4,4-dimethyl-1-pentene, was obtained as a colorless solid **3k** (94 mg, 79%).

$^{1}H\{^{11}B\}$ NMR (400 MHz, acetone-d_6, 23 °C): δ 6.13-5.83 (m, 5H, alkenyl CH), 5.67 (d, J = 17.40 Hz, 5H, alkenyl CH), 3.48 (q, J = 7.3 Hz, 8H, CH_2 of cation), 1.98-1.84 (m, 10H, CH_2), 1.83-1.52 (overlapping broad signals, 6H, BH), 1.39 (tt, J = 7.3 Hz, 1.6 Hz, 12H, CH_3 of cation), 0.94-0.82 (s, 45H, $(CH_3)_3C$).

$^{11}B\{^{1}H\}$ NMR (128 MHz, acetone-d_6, 23 °C): δ ca. -3.02 to -10.49 (overlapping signals, 6B), -13.57 (5B).

$^{13}C\{^{1}H\}$ NMR (126 MHz, acetone-d_6, 23 °C): δ 164.64 (CO), 138.29 (BCH*C*H), 134.09 (broad signal, B*C*HCH), 68.35 (cage C), 53.00 (CH_2 of cation), 51.65 (CH_2), 31.73 ($(CH_3)_3C$), 7.67 (CH_3 of cation). The carborane CH_3 signal could not be detected unambiguously because of overlap with the solvent signal.

HRMS (ESI): *m/z* Calcd. for $[C_{37}H_{72}B_{11}O_2]^-$, 667.6629; found, 667.6638.

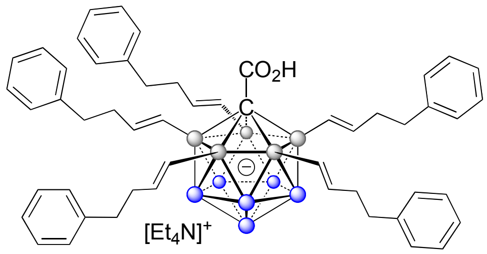

3l: Prepared following the general procedure A, using **1a** and 4-phenyl-1-butene, **3l** was obtained as a colorless solid (110 mg, 76%).

$^{1}H\{^{11}B\}$ NMR (400 MHz, acetone-d_6, 23 °C): δ 7.38-7.18 (overlapping m, 20H, ArH), 7.17-7.06 (m, 5H, ArH), 6.20-5.88 (m, 5H, alkenyl CH), 5.75 (d, J = 17.5 Hz, 5H, alkenyl CH), 3.38 (q, J = 7.3 Hz, 8H, CH_2 of cation), 2.79-2.55 (m, 10H, CH_2), 2.46-2.20 (m, 10H, CH_2), 2.01-1.53 (overlapping broad signals, 6H, BH), 1.32 (tt, J = 7.3 Hz, 1.6 Hz, 12H, CH_3 of cation).

$^{11}B\{^{1}H\}$ NMR (128 MHz, acetone-d_6, 23 °C): δ ca. -0.80 to -10.66 (overlapping signals, 6B), -13.47 (5B).

$^{13}C\{^{1}H\}$ NMR (101 MHz, acetone-d_6, 23 °C): δ 164.82 (CO), 143.45 (BCH*C*H), 140.67 (aryl C), 131.41 (broad signal, BCH), 129.20 (aryl CH), 128.91 (aryl CH), 126.24 (aryl CH), 68.42 (cage C), 52.91 (CH_2 of cation), 38.98 (CH_2), 37.00 (CH_2) 7.63 (CH_3 of cation).

HRMS (ESI): *m/z* Calcd. for $[C_{52}H_{62}B_{11}O_2]^-$, 837.5846; found, 837.5870.

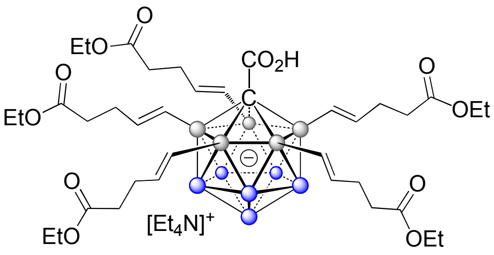

3m: Prepared following the general procedure A, using **1a** and ethyl 4-petenoate, **3m** was obtained as a colorless solid (94 mg, 66%).

$^{1}H\{^{11}B\}$ NMR (500 MHz, acetone-d_6, 23 °C): δ 5.99-5.74 (m, 5H, BCHC*H*), 5.64 (d, *J* = 17.5 Hz, 5H, BC*H*CH), 4.07 (q, *J* = 7.1 Hz, 10H, CH_3CH_2), 3.50 (q, *J* = 7.3 Hz, 8H, CH_2 of cation), 2.51-2.16 (m, 20H, $(CH_2)_2CO_2Et$), 1.90-1.51 (broad overlapping signals, 6H, BH), 1.40 (tt, *J* = 7.3 Hz, 1.6 Hz, 12H, CH_3 of cation), 1.21 (t, *J* = 7.1 Hz, 15H, CH_3CH_2).

$^{11}B\{^{1}H\}$ NMR (160 MHz, acetone-d_6, 23 °C): δ ca. -4.55 to -9.71 (overlapping signals, 6B), -13.56 (5B).

$^{13}C\{^{1}H\}$ NMR (126 MHz, acetone-d_6, 23 °C): δ 173.42 (CO_2Et), 164.82 (COOH), 139.02 (BCH*C*H), 131.98 (broad signal, BCH), 66.28 (cage C), 60.36 (CH_3CH_2), 53.04 (CH_2 of cation), 35.03 ($CH_2CH_2CO_2Et$), 32.08 ($CH_2CH_2CO_2Et$), 14.62 (CH_3CH_2), 7.69 (CH_3 of cation).

HRMS (ESI): *m/z* Calcd. for $[C_{37}H_{62}B_{11}O_{12}]^-$, 817.5338; found, 817.5357.

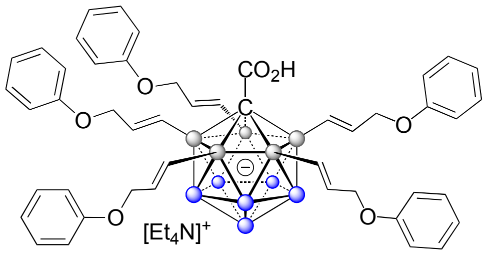

3n: Prepared following the general procedure A, using **1a** and allyl phenyl ether, **3n** was obtained as a colorless solid (108 mg, 74%).

$^{1}H\{^{11}B\}$ NMR (500 MHz, acetone-d_6, 23 °C): δ 7.30-7.12 (m, 10H, ArH), 7.00-6.87 (m, 10H, ArH), 6.86-6.80 (m, 5H, ArH), 6.19-5.87 (m, 10H, alkenyl CH), 4.38 (d, J = 4.7 Hz, 10H), 3.42 (q, J = 7.3 Hz, 8H, CH_2 of cation), 2.04-1.44 (broad overlapping signals, 6H, BH), 1.35 (tt, J = 7.3 Hz, 1.6 Hz, 12H, CH_3 of cation).
$^{11}B\{^{1}H\}$ NMR (160 MHz, acetone-d_6, 23 °C): δ ca. -4.49 to -11.40 (overlapping signals, 6B), -13.36 (5B).
$^{13}C\{^{1}H\}$ NMR (126 MHz, acetone-d_6, 23 °C): δ 165.48 (CO), 160.03 (aryl C), 135.98 (BCH*C*H), 135.05 (broad signal, BCH), 130.01 (aryl CH), 120.79 (aryl CH), 115.77 (aryl CH), 71.99 (CH_2), 67.73 (cage C), 52.99 (CH_2 of cation), 7.66 (CH_3 of cation).
HRMS (ESI): m/z Calcd. for $[C_{47}H_{52}B_{11}O_7]^-$, 847.4809; found, 847.4828.

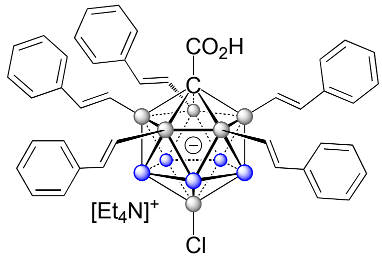

3o: Prepared following the general procedure A, using **1b** and styrene, **3o** was obtained as a colorless solid (92 mg, 71%).

$^1H\{^{11}B\}$ NMR (500 MHz, acetone-d_6, 23 °C): δ 7.45-7.31 (m, 10H, ArH), 7.29-7.18 (m, 10H, ArH), 7.17-7.08 (m, 5H, ArH), 6.99 (d, J= 18.0 Hz, 5H, alkenyl CH), 6.68 (d, J = 18.0 Hz, 5H, alkenyl CH), 3.28 (q, J = 7.3 Hz, 8H, CH_2 of cation), 2.61-2.05 (broad signal, 5H, BH), 1.26 (tt, J = 7.3 Hz, 1.6 Hz, 12H, CH_3 of cation).
$^{11}B\{^1H\}$ NMR (160 MHz, acetone-d_6, 23 °C): δ 3.72 (s, 1B), -7.07 (5B), -12.73 (5B).
$^{13}C\{^1H\}$ NMR (126 MHz, acetone-d_6, 23 °C): δ 166.52 (CO), 140.93 (aryl C), 140.19 (BCH*C*H), 130.71 (broad signal, BCH), 129.15 (aryl CH), 127.33 (aryl CH), 126.59 (aryl CH), 60.55 (cage C), 52.92 (CH_2 of cation), 7.62 (CH_3 of cation).
HRMS (ESI): m/z Calcd. for $[C_{42}H_{41}B_{11}ClO_2]^-$, 731.3891; found, 731.3910.

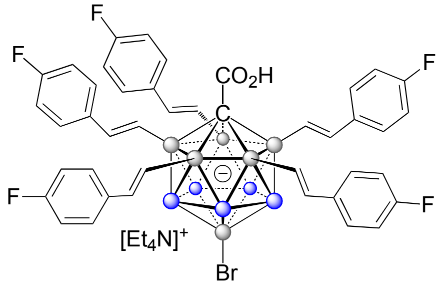

3p: Prepared following the general procedure A, using **1c** and 4-fluorostyrene, **3p** was obtained as a colorless solid (116 mg, 78%).

$^{1}H\{^{11}B\}$ NMR (500 MHz, acetone-d_6, 23 °C): δ 7.48-7.30 (m, 10H, ArH), 7.09-6.97 (m, 10H, ArH), 6.94 (d, J = 18.0 Hz, 5H, alkenyl CH), 6.56 (d, J = 18.0 Hz, 5H, alkenyl CH), 3.41 (q, J = 7.3 Hz, 8H, CH_2 of cation), 2.65-2.05 (broad signal, 5H, BH), 1.34 (tt, J = 7.3 Hz, 1.6 Hz, 12H, CH_3 of cation).
$^{11}B\{^{1}H\}$ NMR (128 MHz, acetone-d_6, 23 °C): δ -3.01 (s, 1B), -6.72 (5B), -12.66 (5B).
$^{13}C\{^{1}H\}$ NMR (100 MHz, acetone-d_6, 23 °C): δ 166.53 (CO), 162.57 (d, J = 243 Hz, C-F), 138.97 (BCH*C*H), 137.36 (aryl C, C(*ipso*)), 130.36 (broad signal, BCH), 128.19 (d, J = 6.1 Hz, C(*ortho*)), 115.82 (d, J = 21.5 Hz, C(*meta*)), 62.44 (cage C), 52.97 (CH_2 of cation), 7.64 (CH_3 of cation).
HRMS (ESI): *m/z* Calcd. for $[C_{42}H_{36}B_{11}BrF_5O_2]^-$, 865.2915; found, 865.2956.

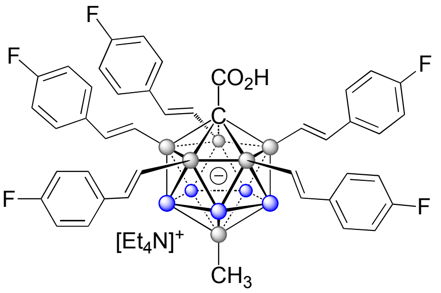

3q: Prepared following the general procedure A, using **1d** and 4-fluorostyrene, **3q** was obtained as a colorless solid (114 mg, 82%).

^{1}H{^{11}B} NMR (400 MHz, acetone-d_6, 23 °C): δ 7.45-7.30 (m, 10H, ArH), 7.09-6.96 (m, 10H, ArH), 6.92 (d, J = 18.0 Hz, 5H, alkenyl CH), 6.63 (d, J = 18.0 Hz, 5H, alkenyl CH), 3.35 (q, J = 7.3 Hz, 8H, CH_2 of cation), 2.65-2.02 (broad signal, 5H, BH), 1.30 (tt, J = 7.3 Hz, 1.6 Hz, 12H, CH_3 of cation), 0.23 (s, 3H, CH_3).
^{11}B{^{1}H} NMR (128 MHz, acetone-d_6, 23 °C): δ 1.39 (s, 1B), -6.15 (5B), -11.6 (5B).
^{13}C{^{1}H} NMR (100 MHz, acetone-d_6, 23 °C): δ 166.47 (CO), 163.58 (aryl C), 162.38 (d, J = 243 Hz, C-F), 138.11 (BCH*C*H), 137.68 (aryl C, C(*ipso*)), 131.95 (broad signal, BCH), 128.01 (d, J = 7.6 Hz, C(*ortho*)), 115.73 (d, J = 21.4 Hz, C(*meta*)), 62.27 (cage C), 52.89 (CH_2 of cation), 7.57 (CH_3 of cation), 4.83 (broad signal, CH_3).
HRMS (ESI): *m/z* Calcd. for $[C_{43}H_{39}B_{11}F_5O_2]^-$, 801.3966; found, 801.3983.

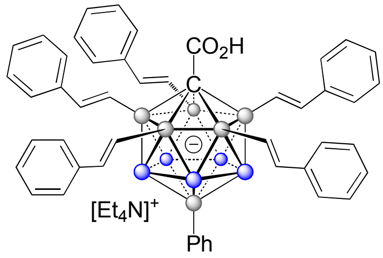

3r: Prepared following the general procedure A, using **1e** and styrene, **3r** was obtained as a colorless solid (84 mg, 62%).

$^{1}H\{^{11}B\}$ NMR (400 MHz, acetone-d_6, 23 °C): δ 7.65-7.52 (m, 2H, ArH), 7.44-7.31 (m, 10H, ArH), 7.29-7.17 (m, 10H, ArH), 7.16-7.08 (overlapping m, 7H, ArH), 7.07-6.95 (overlapping m, 6H, ArH, alkenyl CH), 6.77 (d, J = 18.0 Hz, 5H, alkenyl CH), 3.20 (q, J = 7.3 Hz, 8H, CH_2 of cation), 2.61-2.07 (broad signal, 5H, BH), 1.20 (tt, J = 7.3 Hz, 1.6 Hz, 12H, CH_3 of cation).

$^{11}B\{^{1}H\}$ NMR (128 MHz, acetone-d_6, 23 °C): δ 1.87 (s, 1B), -6.18 (5B), -12.35 (5B).

$^{13}C\{^{1}H\}$ NMR (100 MHz, acetone-d_6, 23 °C): δ 166.48 (CO), 141.17 (aryl C), 139.72 (BCH*C*H), 133.77(aryl C), 131.74 (broad signal, BCH), 129.12 (aryl CH), 127.15 (aryl CH), 127.04 (aryl C), 126.56 (aryl C), 125.60 (aryl C), 64.48 (cage C), 52.83 (CH_2 of cation), 7.57 (CH_3 of cation). The B12-C signal could not be detected unambiguously.

HRMS (ESI): *m/z* Calcd. for $[C_{48}H_{46}B_{11}O_2]^-$, 773.4594; found, 773.4615.

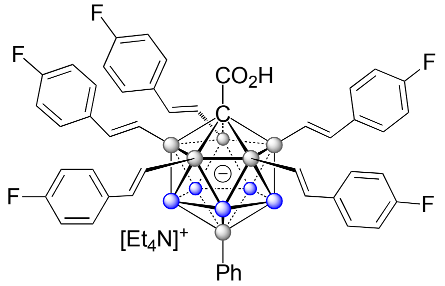

3s: Prepared following the general procedure A, using **1e** and 4-fluorostyrene, **3s** was obtained as a colorless solid (103 mg, 69%).

^{1}H{^{11}B} NMR (400 MHz, acetone-d_6, 23 °C): δ 7.64-7.54 (m, 2H, ArH), 7.47-7.32 (m, 10H, ArH), 7.17-7.09 (m, 2H, ArH), 7.08-6.90 (overlapping m, 16H, ArH, alkenyl CH), 6.67 (d, J = 18.0 Hz, 5H, alkenyl CH), 3.28 (q, J = 7.3 Hz, 8H, CH_2 of cation), 2.60-2.05 (broad signal, 5H, BH), 1.26 (tt, J = 7.3 Hz, 1.6 Hz, 12H, CH_3 of cation).
^{11}B{^{1}H} NMR (128 MHz, acetone-d_6, 23 °C): δ 1.95 (s, 1B), -6.16 (5B), -12.31 (5B).
^{13}C{^{1}H} NMR (101 MHz, acetone-d_6, 23 °C): δ 166.44 (CO), 162.46 (d, J = 243 Hz, C-F), 138.43 (BCH*C*H), 137.59 (aryl C, C(*ipso*)), 133.74 (aryl C) 131.58 (broad signal, BCH), 128.10 (d, J = 7.6 Hz, C(*ortho*)), 127.07 (aryl C), 125.68 (aryl C), 115.78 (d, J = 21.4 Hz, C(*meta*)), 64.37 (cage C), 52.85 (CH_2 of cation), 7.56 (CH_3 of cation). The B12-C signal could not be detected unambiguously.
HRMS (ESI): *m/z* Calcd. for $[C_{48}H_{41}B_{11}F_5O_2]^-$, 863.4123; found, 863.4145.

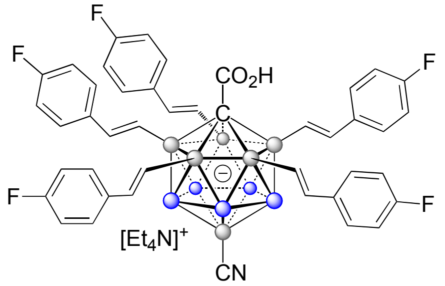

3t: Prepared following the general procedure A, using **1f** and 4-fluorostyrene, **3t** was obtained as a colorless solid (102 mg, 72%).

$^{1}H\{^{11}B\}$ NMR (400 MHz, acetone-d_6, 23 °C): δ 7.49-7.28 (m, 10H, ArH), 7.12-6.97 (m, 10H, ArH), 6.93 (d, J = 18.0 Hz, 5H, alkenyl CH), 6.55 (d, J = 18.0 Hz, 5H, alkenyl CH), 3.43 (q, J = 7.3 Hz, 8H, CH_2 of cation), 2.58-2.05 (broad signal, 5H, BH), 1.35 (tt, J = 7.3 Hz, 1.6 Hz, 12H, CH_3 of cation).
$^{11}B\{^{1}H\}$ NMR (160 MHz, acetone-d_6, 23 °C): δ -6.31 (5B), ca. -10.55 to -16.60 (overlapping signals, 6B).
$^{13}C\{^{1}H\}$ NMR (101 MHz, acetone-d_6, 23 °C): δ 165.44 (CO), 162.44 (d, J = 244 Hz, C-F), 139.28 (BCH*C*H), 137.22 (aryl C, C(*ipso*)), 129.82 (broad signal, BCH), 128.26 (d, J = 7.9 Hz, C(*ortho*)), 115.86 (d, J = 21.6 Hz, C(*meta*)), 69.05 (cage C), 52.94 (CH_2 of cation), 7.63 (CH_3 of cation). The B-CN signal could not be detected unambiguously.
HRMS (ESI): *m/z* Calcd. for $[C_{43}H_{36}B_{11}F_5NO_2]^-$, 812.3762; found, 812.3785.

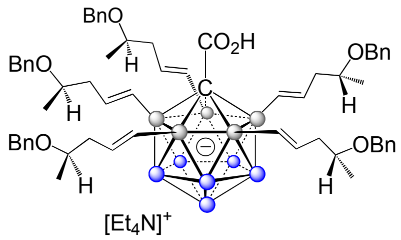

3u: Prepared following the general procedure, using **1a** and {[(2*R*)-pent-4-en-2-yloxy]methyl}benzene, **3u** was obtained as a colorless solid (130 mg, 73%). The eluent for column chromatography was EtOAc.

$^1H\{^{11}B\}$ NMR (400 MHz, acetone-d_6, 23 °C): δ 7.43-7.26 (overlapping m, 20H, ArH), 7.25-7.17 (m, 5H, ArH), 6.02-5.87 (m, 5H, alkenyl CH), 5.76 (d, J = 17.4 Hz, 5H, alkenyl CH), 4.57-4.42 (m, 10H, OCH_2), 3.60-3.44 (m, 5H, OCH), 3.36 (q, J = 7.3 Hz, 8H, CH_2 of cation), 2.50-2.32 (m, 5H, CHCH_2), 2.18-2.04 (m, 5H, CHCH_2) 1.96-1.54 (overlapping broad signals, 6H, BH), 1.32 (tt, J = 7.3 Hz, 1.6 Hz, 12H, CH_3 of cation), 1.14 (d, J = 6.0 Hz, 15H, CH_3CH).

$^{11}B\{^1H\}$ NMR (128 MHz, acetone-d_6, 23 °C): δ ca. -1.60 to -10.54 (overlapping signals, 6B), -13.52 (5B).

$^{13}C\{^1H\}$ NMR (101 MHz, acetone-d_6, 23 °C): δ 165.05 (CO), 140.73 (BCH*C*H), 137.22 (aryl C), 134.13 (broad signal, BCH), 128.87 (aryl CH), 128.14 (aryl CH), 127.73 (aryl CH), 76.19 (OCH), 70.66 (OCH_2), 68.12 (cage C), 52.91 (CH_2 of cation), 44.18 (CH*C*H_2), 20.07 (*C*H_3CH), 7.64 (CH_3 of cation).

HRMS (ESI): *m/z* Calcd. for $[C_{62}H_{82}B_{11}O_7]^-$, 1057.7157; found, 1057.7194.

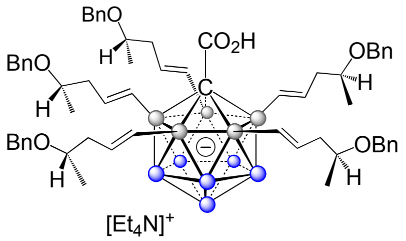

3v: Prepared following the general procedure, using **1a** and {[(2*S*)-pent-4-en-2-yloxy]methyl}benzene, **3v** was obtained as a colorless solid (126 mg, 71%). The eluent for column chromatography was EtOAc.

^{1}H{^{11}B} NMR (500 MHz, acetone-d_6, 23 °C): δ 7.43-7.26 (overlapping m, 20H, ArH), 7.25-7.17 (m, 5H, ArH), 6.02-5.87 (m, 5H, alkenyl CH), 5.76 (d, J = 17.4 Hz, 5H, alkenyl CH), 4.57-4.42 (m, 10H, OCH_2), 3.56-3.48 (m, 5H, OCH), 3.45 (q, J = 7.3 Hz, 8H, CH_2 of cation), 2.49-2.35 (m, 5H, CHCH_2), 2.12-2.06 (m, 5H, CHCH_2) 1.92-1.60 (broad overlapping signals, 6H, BH), 1.37 (tt, J = 7.3 Hz, 1.6 Hz, 12H, CH_3 of cation), 1.14 (d, J = 6.0 Hz, 15H, CH_3CH).

^{11}B{^{1}H} NMR (128 MHz, acetone-d_6, 23 °C): δ ca. -1.60 to -10.54 (overlapping signals, 6B), -13.54 (5B).

^{13}C{^{1}H} NMR (126 MHz, acetone-d_6, 23 °C): δ 165.03 (CO), 140.84 (aryl C), 137.33 (BCH*C*H), 134.07 (broad signal, BCH), 128.90 (aryl CH), 128.19 (aryl CH), 127.74 (aryl CH), 76.28 (OCH), 70.72 (OCH_2), 68.23 (cage C), 53.01 (CH_2 of cation), 44.26 (CH*C*H_2), 20.13 (*C*H_3CH), 7.68 (CH_3 of cation).

HRMS (ESI): *m/z* Calcd. for $[C_{62}H_{82}B_{11}O_7]^-$, 1057.7157; found, 1057.7190.

General Procedure B for the decarboxylation of the penta-alkenylation products

A 10 mL microwave vial was charged with carborane acid **1** (0.060 mmol), NaOAc (0.60 mmol) and capped under nitrogen. DMA (4 mL) was added via a syringe, and the resulting mixture was heated to 150 °C in a microwave reactor. The progress of the reaction was monitored by (–)-ESI-MS analysis. After the completion of the reaction, all the volatiles were removed in vacuo, and the residue was dissolved in EtOAc and washed with an aqueous solution of $[Et_4N]Br$ (3 x 10 mL). The organic layer was dried over $MgSO_4$ and concentrated under reduced pressure. The crude product was purified by column chromatography (silica gel, eluent: dichloromethane) to afford **4** as a colorless solid.

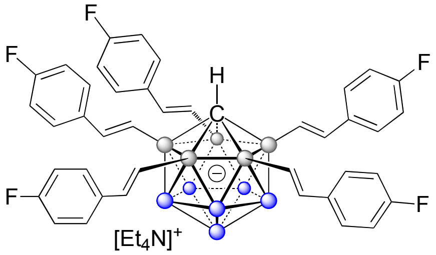

4a: Prepared following the general procedure, starting from **3a** , **4a** was obtained as a colorless solid (45 mg, 86%).

^{1}H{^{11}B} NMR (400 MHz, acetone-d_6, 23 °C): δ 7.41-7.27 (m, 10H, ArH), 7.06-6.86 (m, 10H, ArH), 6.78 (d, J = 18.0 Hz, 5H, alkenyl CH), 6.43 (d, J = 18.0 Hz, 5H, alkenyl CH), 3.45 (q, J = 7.3 Hz, 8H, CH_2 of cation), 2.81 (s, 1H, cage CH), 2.05-1.75 (broad overlapping signals, 6H, BH), 1.37 (tt, J = 7.3 Hz, 1.6 Hz, 12H, CH_3 of cation).
^{11}B{^{1}H} NMR (128 MHz, acetone-d_6, 23 °C): δ ca. -4.70 to -11.20 (overlapping signals with peaks at -7.70 and -9.67, 6B), -13.03 (5B).
^{13}C{^{1}H} NMR (100 MHz, Acetone-d_6, 23 °C): δ 162.43 (d, J = 243 Hz, C-F), 137.97 (BCH*C*H), 137.47 (d, J = 2.8 Hz, C(*ipso*)), 132.40 (broad signal, BCH), 128.00 (d, J = 8.0 Hz, C(*ortho*)), 115.74 (d, J = 21.5 Hz, C(*meta*)), 57.27 (cage C), 52.97 (CH_2 of cation), 7.64 (CH_3 of cation).
HRMS (ESI): *m/z* Calcd. for $[C_{41}H_{37}B_{11}F_5]^-$, 743.3912; found, 743.3928.

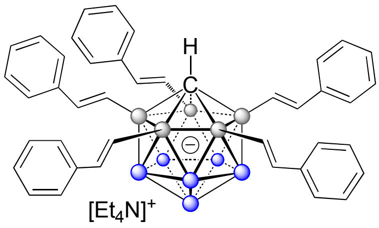

4b: Prepared following the general procedure, starting from **3b**, **4b** was obtained as a colorless solid (37 mg, 79%).

$^1H\{^{11}B\}$ NMR (400 MHz, acetone-d_6, 23 °C): δ 7.40-7.28 (m, 10H, ArH), 7.27-7.14 (m, 10H, ArH), 7.13-6.98 (m, 5H, ArH), 6.84 (d, *J* = 18.0 Hz, 5H, alkenyl CH), 6.54 (d, *J* = 18.0 Hz, 5H, alkenyl CH), 3.36 (q, *J* = 7.3 Hz, 8H, CH_2 of cation), 2.06-1.74 (broad overlapping signals, 6H, BH), 1.31 (tt, *J* = 7.3 Hz, 1.6 Hz, 12H, CH_3 of cation). The $^1H/^{13}C$ HSQC spectrum shows that the cage CH signal is overlapping with the double water peak.

$^{11}B\{^1H\}$ NMR (128 MHz, acetone-d_6, 23 °C): δ ca. -4.98 to -11.30 (overlapping signals with peaks at -7.63 and -9.71, 6B), -13.02 (5B).

$^{13}C\{^1H\}$ NMR (101 MHz, acetone-d_6, 23 °C): δ 141.00 (aryl C), 139.26 (BCH*C*H), 132.78 (broad signal, BCH), 129.08 (aryl CH), 127.07 (aryl CH), 126.46 (aryl CH), 57.42 (cage C), 52.93 (CH_2 of cation), 7.61 (CH_3 of cation).

HRMS (ESI): *m/z* Calcd. for $[C_{41}H_{42}B_{11}]^-$, 653.4383; found, 653.4398.

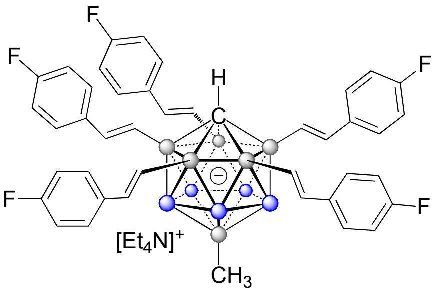

4q: Prepared following the general procedure, starting from **3q**, **4q** was obtained as a colorless solid (46 mg, 87%).

^{1}H{^{11}B} NMR (400 MHz, acetone-d_6, 23 °C): δ 7.42-7.26 (m, 10H, ArH), 7.05-6.89 (m, 10H, ArH), 6.77 (d, *J*= 18.0 Hz, 5H, alkenyl CH), 6.43 (d, *J* = 18.0 Hz, 5H, alkenyl CH), 3.45 (q, *J* = 7.3 Hz, 8H, CH_2 of cation), 2.57 (s, 1H, cage CH), 2.05-1.76 (broad signal, 5H, BH), 1.36 (tt, *J* = 7.3 Hz, 1.6 Hz, 12H, CH_3 of cation), 0.16 (s, 3H, CH_3).
^{11}B{^{1}H} NMR (128 MHz, acetone-d_6, 23 °C): δ -0.98 (s, 1B), -8.03 (5B), -11.68 (5B).
^{13}C{^{1}H} NMR (101 MHz, acetone-d_6, 23 °C): δ 162.38 (d, *J* = 243 Hz, C-F), 137.82 (BCH*C*H), 137.53 (aryl C, C(*ipso*)), 132.55 (broad signal, BCH), 127.97 (d, *J* = 7.7 Hz, C(*ortho*)), 115.73 (d, *J* = 21.5 Hz, C(*meta*)), 52.96 (CH_2 of cation), 51.00 (cage C), 7.63 (CH_3 of cation). The B–CH_3 signal could not be detected unambiguously.
HRMS (ESI): *m/z* Calcd. for $[C_{42}H_{39}B_{11}F_5]^-$, 757.4068; found, 757.4084.

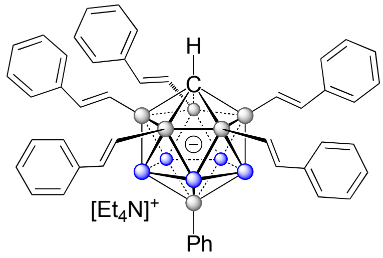

4r: Prepared following the general procedure, starting from **3r**, **4r** was obtained as a colorless solid (39 mg, 76%).

$^{1}H\{^{11}B\}$ NMR (500 MHz, acetone-d_6, 23 °C): δ 7.63-7.52 (m, 2H, ArH), 7.39-7.29 (m, 10H, ArH), 7.25-7.15 (m, 10H, ArH), 7.13-7.04 (overlapping m, 7H, ArH), 7.03-6.97 (m, 1H, ArH), 6.89 (d, J = 18.0 Hz, 5H, alkenyl CH), 6.58 (d, J = 18.0 Hz, 5H, alkenyl CH), 3.34 (q, J = 7.3 Hz, 8H, CH_2 of cation), 2.79 (s, 1H, cage CH), 2.54-2.06 (broad signal, 5H, BH), 1.29 (tt, J = 7.3 Hz, 1.6 Hz, 12H, CH_3 of cation).
The cage CH signal is overlapping with the double water peak.
$^{11}B\{^{1}H\}$ NMR (160 MHz, acetone-d_6, 23 °C): δ -0.23 (s, 1B), -7.85 (5B), -12.36 (5B).
$^{13}C\{^{1}H\}$ NMR (126 MHz, acetone-d_6, 23 °C): δ 141.03 (aryl C), 139.45 (BCH*C*H), 133.94 (aryl C), 132.67 (broad signal, BCH), 129.10 (aryl CH), 127.10 (aryl CH), 126.93 (aryl CH), 126.51 (aryl CH), 125.32 (aryl CH), 53.38 (cage C), 52.94 (CH_2 of cation), 7.62 (CH_3 of cation). The B–C(Ph) signal could not be detected unambiguously.
HRMS (ESI): *m/z* Calcd. for $[C_{47}H_{46}B_{11}]^-$, 729.4696; found, 729.4721.

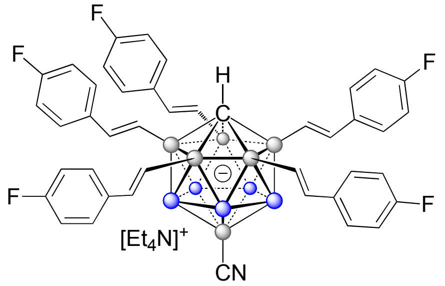

4t: Prepared following the general procedure, starting from **3t**, **4t** was obtained as a colorless solid (43 mg, 80%).

$^{1}H\{^{11}B\}$ NMR (400 MHz, acetone-d_6, 23 °C): δ 7.43-7.30 (m, 10H, ArH), 7.05-6.93 (m, 10H, ArH), 6.81 (d, J = 18.0 Hz, 5H, alkenyl CH), 6.38 (d, J = 18.0 Hz, 5H, alkenyl CH), 3.46 (q, J = 7.3 Hz, 8H, CH_2 of cation), 2.96 (s, 1H, cage CH), 2.44-2.05 (broad signal, 5H, BH), 1.38 (tt, J = 7.3 Hz, 1.6 Hz, 12H, CH_3 of cation).
$^{11}B\{^{1}H\}$ NMR (128 MHz, acetone-d_6, 23 °C): δ -7.75 (5B), -13.38 (5B), -15.53 (1B).
$^{13}C\{^{1}H\}$ NMR (100 MHz, acetone-d_6, 23 °C): δ 163.82 (d, J = 243 Hz, C-F), 138.98 (BCH*C*H), 137.05 (d, J = 2.2 Hz, C(*ipso*)), 130.59 (broad signal, BCH), 128.19 (d, J = 7.8 Hz, C(*ortho*)), 115.83 (d, J = 21.6 Hz, C(*meta*)), 57.87 (cage C), 52.96 (CH_2 of cation), 7.64 (CH_3 of cation). The B–CN signal could not be detected unambiguously.
HRMS (ESI): *m/z* Calcd. for $[C_{42}H_{36}B_{11}F_5N]^-$, 768.3864; found, 768.3879.

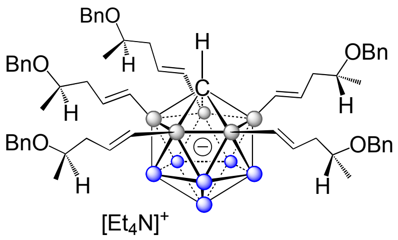

4u: Prepared following the general procedure, starting from **3u**, **4u** was obtained as a colorless solid (58 mg, 84%).

$^{1}H\{^{11}B\}$ NMR (500 MHz, acetone-d_6, 23 °C): δ 7.40-7.33 (m, 10H, ArH), 7.33-7.27 (m, 10H, ArH), 7.25-7.18 (m, 5H, ArH), 5.79-5.63 (m, 5H, alkenyl CH), 5.55 (d, J = 17.4 Hz, 5H, alkenyl CH), 4.62-4.39 (m, 10H, OCH_2), 3.58-3.40 (overlapping signals, 13H, OCH and CH_2 of cation), 2.50-2.28 (m, 5H, $CHCH_2$), 2.14-2.02 (m, 5H, $CHCH_2$) 1.89-1.47 (broad overlapping signals, 6H, BH), 1.38 (tt, J = 7.3 Hz, 1.6 Hz, 12H, CH_3 of cation), 1.14 (d, J = 6.0 Hz, 15H, CH_3CH).
The $^{1}H/^{13}C$ HSQC spectrum shows that the cage CH signal is overlapping with the solvent residual signal and the multiplet at 2.14-2.02 ppm.
$^{11}B\{^{1}H\}$ NMR (160 MHz, acetone-d_6, 23 °C): δ ca. -5.86 to -12.58 (overlapping signals with peaks at -8.53 and -10.36, 6B), -13.89 (5B).
$^{13}C\{^{1}H\}$ NMR (126 MHz, acetone-d_6, 23 °C): δ 140.81 (BCH*C*H), 135.74 (aryl C), 135.20 (broad signal, BCH), 128.90 (aryl CH), 128.16 (aryl CH), 127.77 (aryl CH), 76.27(OCH), 70.66 (OCH_2), 56.96 (cage C), 53.00 (CH_2 of cation), 44.13 ($CHCH_2$), 20.09 (CH_3CH), 7.67 (CH_3 of cation).
HRMS (ESI): *m/z* Calcd. for $[C_{61}H_{82}B_{11}O_5]^-$, 1013.7258; found, 1013.7244.

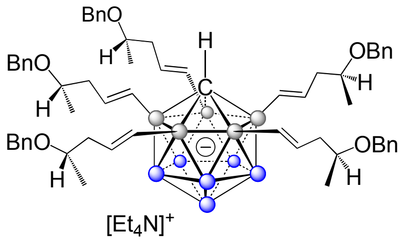

4v: Prepared following the general procedure, starting from **3v**, **4v** was obtained as a colorless solid (55 mg, 80%).

$^{1}H\{^{11}B\}$ NMR (500 MHz, acetone-d_6, 23 °C): δ 7.41-7.33 (m, 10H, ArH), 7.33-7.27 (m, 10H, ArH), 7.26-7.18 (m, 5H, ArH), 5.78-5.64 (m, 5H, alkenyl CH), 5.55 (d, J = 17.4 Hz, 5H, alkenyl CH), 4.60-4.40 (m, 10H, OCH_2), 3.56-3.38 (overlapping signals, 13H, OCH and CH_2 of cation), 2.47-2.30 (m, 5H, CHCH_2), 2.11-2.04 (m, 5H, CHCH_2), 1.80-1.50 (broad overlapping signals, 6H, BH), 1.38 (tt, J = 7.3 Hz, 1.6 Hz, 12H, CH_3 of cation), 1.14 (d, J = 6.0 Hz, 15H, CH_3CH).

The cage CH signal is overlapping with the solvent residual signal and the multiplet at 2.11-2.04 ppm.

$^{11}B\{^{1}H\}$ NMR (160 MHz, acetone-d_6, 23 °C): δ ca. -5.46 to -12.18 (overlapping signals with peaks at -8.47 and -10.39, 6B), -13.86 (5B).

$^{13}C\{^{1}H\}$ NMR (126 MHz, acetone-d_6, 23 °C): δ 140.82 (BCHCH), 135.34 (aryl C, BCH), 128.90 (aryl CH), 128.16 (aryl CH), 127.77 (aryl CH), 76.28 (OCH), 70.67 (OCH_2), 56.97 (cage C), 53.01 (CH_2 of cation), 44.13 (CHCH_2), 20.09 (CH$_3$CH), 7.67 (CH_3 of cation).

HRMS (ESI): m/z Calcd. for $[C_{61}H_{82}B_{11}O_5]^-$, 1013.7258; found, 1013.7291.

General Procedure C for the reduction of the penta-alkenylation products

To a solution of **3** (0.10 mmol) in THF (8 mL) in a 25 mL round-bottom flask was added 10% Pd on carbon (20 mg). The mixture was stirred under H_2 (balloon presure) for 2 h. The catalyst was filtered off, and the solvent was removed under reduced pressure. The crude product was purified by column chromatography (silica gel, eluent: dichloromethane) to afford **5** as a colorless solid.

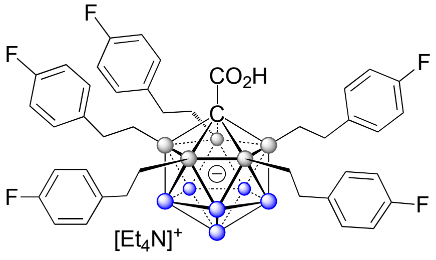

5a: Prepared following the general procedure, starting from **3a**, **5a** was obtained as a colorless solid (83 mg, 90%).

$^1H\{^{11}B\}$ NMR (400 MHz, acetone-d_6, 23 °C): δ 7.32-7.17 (m, 10H, ArH), 7.10-6.90 (m, 10H, ArH), 3.45 (q, J = 7.3 Hz, 8H, CH_2 of cation), 2.92-2.78 (m, 10H, BCH_2CH_2), 2.06-1.56 (broad overlapping signals, 6H, BH), 1.37 (tt, J = 7.3 Hz, 1.6 Hz, 12H, CH_3 of cation), 1.22-1.07 (m, 10H, BCH_2CH_2).
$^{11}B\{^1H\}$ NMR (128 MHz, acetone-d_6, 23 °C): δ ca. 0.52 to -9.42 (overlapping signals with peaks at -4.52 and -5.81, 6B), -15.59 (5B).
$^{13}C\{^1H\}$ NMR (101 MHz, acetone-d_6, 23 °C): δ 166.26 (CO), 161.60 (d, J = 240 Hz, C-F), 144.10 (aryl C, C(*ipso*)), 130.17 (d, J = 7.6 Hz, C(*ortho*)), 115.40 (d, J = 20.8 Hz, C(*meta*)), 70.98 (cage C), 52.97 (CH_2 of cation), 36.12 (BCH_2CH_2), 19.98 (broad signal, BCH_2CH_2), 7.64 (CH_3 of cation).
HRMS (ESI): *m/z* Calcd. for $[C_{42}H_{47}B_{11}F_5O_2]^-$, 797.4592; found, 797.4633.

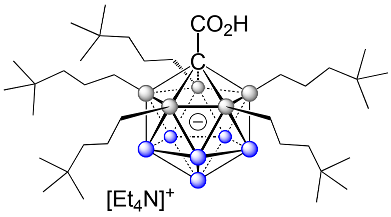

5k: Prepared following the general procedure, starting from **3k**, **5k** was obtained as a colorless solid (70 mg, 87%).

$^{1}H\{^{11}B\}$ NMR (400 MHz, acetone-d_6, 23 °C): δ 3.50 (q, J = 7.3 Hz, 8H, CH_2 of cation), 2.00-1.50 (broad overlapping signals, 6H, BH), 1.49-1.33 (m, 22H, CH_2 of cation and BCH_2CH_2), 1.30-1.18 (m, 10H, $(CH_3)_3CCH_2$), 0.87 (s, 45H, $(CH_3)_3C$), 0.75-0.62 (m, 10H, BCH_2CH_2).

$^{11}B\{^{1}H\}$ NMR (128 MHz, acetone-d_6, 23 °C): δ ca. -0.05 to -9.03 (overlapping signals with peaks at -4.49 and -6.59, 6B), -15.78 (5B).

$^{13}C\{^{1}H\}$ NMR (101 MHz, acetone-d_6, 23 °C): δ 166.75 (CO), 71.34 (cage C), 53.00 (CH_2 of cation), 49.54, 31.15, 30.07, 25.14, 18.15 (broad signal, BCH_2CH_2), 7.69 (CH_3 of cation).

HRMS (ESI): *m/z* Calcd. for $[C_{37}H_{82}B_{11}O_2]^-$, 677.7411; found, 677.7452.

Preparation of palladium complex 1c-Pd

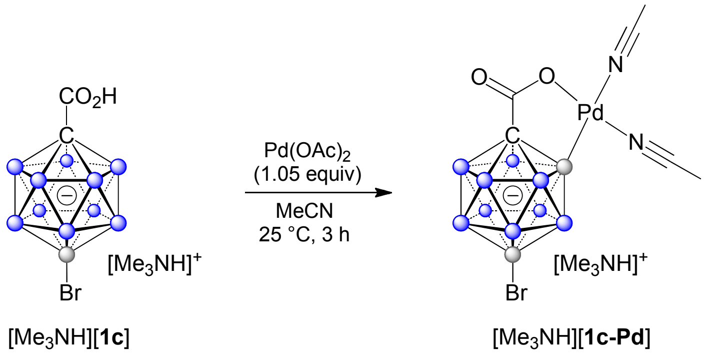

In a glovebox, $Pd(OAc)_2$ (103 mg, 0.458 mmol) was added to a stirred solution of $[Me_3NH][1\text{-}COOH\text{-}CB_{11}H_{10}\text{-}12\text{-}Br]$ (142 mg, 0.437 mmol) in dry acetonitrile (0.50 mL) in a 2 mL glass vial. The resulting mixture was stirred at 25 °C for 5 h. The pale precipitate that formed was collected by vacuum filtration through a fine glass frit, washed with hexane and dried in a vacuum. The filtrate was left to evaporate slowly at room temperature (evaporation of *ca.* 60% of the solvent) to yield colorless crystals, which were collected by filtration. The combined solids were dried at 25 °C in a vacuum to give palladium complex **1c-Pd** (182 mg, 82%).

$^{1}H\{^{11}B\}$ NMR (500 MHz, acetonitrile-d_3, 23 °C): δ 2.73 (s, 9H, CH_3 of cation), 1.96 (s, 6H, CH_3CN), 1.90-1.25 (broad overlapping signals, 9H, BH). MeCN ligand exchange with the solvent leads to the signal at 1.96 ppm, which corresponds to free MeCN.

$^{11}B\{^{1}H\}$ NMR (160 MHz, acetonitrile-d_3, 23 °C): δ -3.46 (s, 1B, B-Br), ca. -10.50 to -17.50 (overlapping signals with peaks at -12.64, -13.39 and 14.91, 9B), -21.92 (s, 1B, B-Pd).

$^{13}C\{^{1}H\}$ NMR (126 MHz, acetonitrile-d_3, 23 °C): δ 179.51 (CO), 67.71 (cage C), 45.55 (CH_3 of cation).

Reaction of 1c-Pd with fluorostyrene in the presence of AgOAc

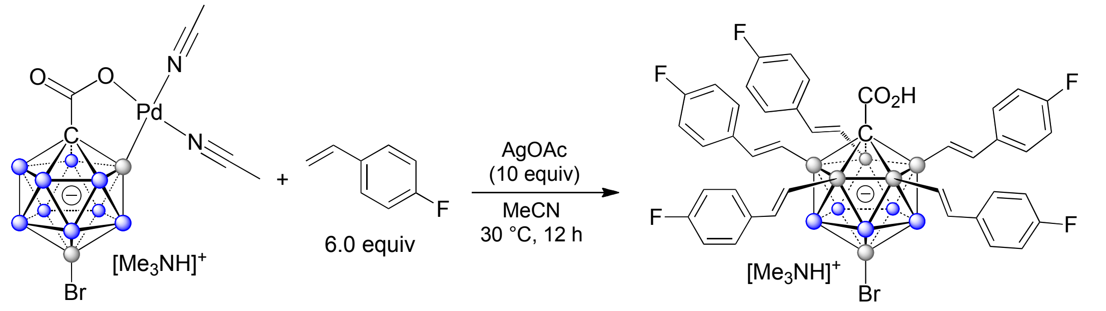

1c-Pd **3p**

Under nitrogen protection, **1c-Pd** (10 mg, 19 µmol), 4-fluorostyrene (6.0 equiv) and AgOAc (10 equiv) were combined in dry MeCN (0.6 mL) in a 4 mL glass vial. The vial was heated to 30 °C, and the reaction was monitored by ESI mass spectrometry. During the transformation, mono-, di-, tri-, tetra- and penta-substituted products were observed, but no other intermediates. The mass spectrum of the reaction mixture after 12 h is displayed in Figure S1. It shows clean formation of the penta-alkenylated product **3p**.

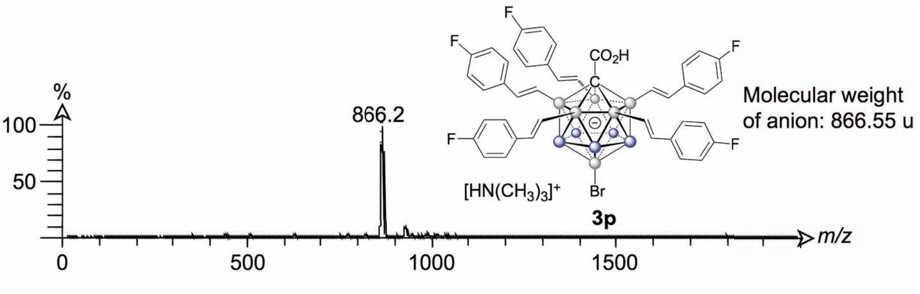

Figure S1. Full-range (–)-ESI mass spectrum of the reaction mixture **1c-Pd** + 4-fluorostyrene (6.0 equiv) + AgOAc (10 equiv) in MeCN after 12 h at 30 °C.

Reaction of 1c-Pd with fluorostyrene in the absence of AgOAc

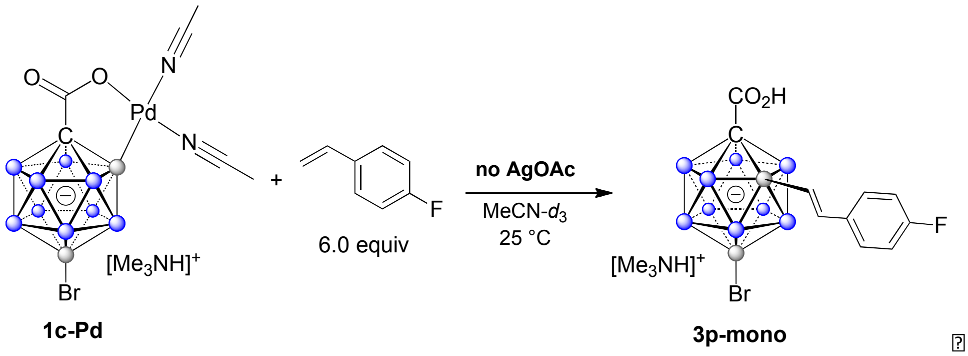

Under nitrogen protection, **1c-Pd** (10 mg, 19 μmol) and 4-fluorostyrene (6.0 equiv) were combined in dry MeCN-d_3 (0.6 mL) in a 4 mL glass vial. The mixture was stirred at 25 °C, and the reaction was monitored by ESI mass spectrometry and NMR spectroscopy. After 1 h, 3 h, 5 h and 12 h, clean formation of the mono-alkenylated product **3p-mono** was observed, and no other intermediates were detected. The $^{1}H\{^{11}B\}$ and $^{11}B\{^{1}H\}$ NMR spectra of the reaction mixture after 3 h are displayed in Figures S2 and S3; the mass spectrum after 3 h is displayed in Figure S4.

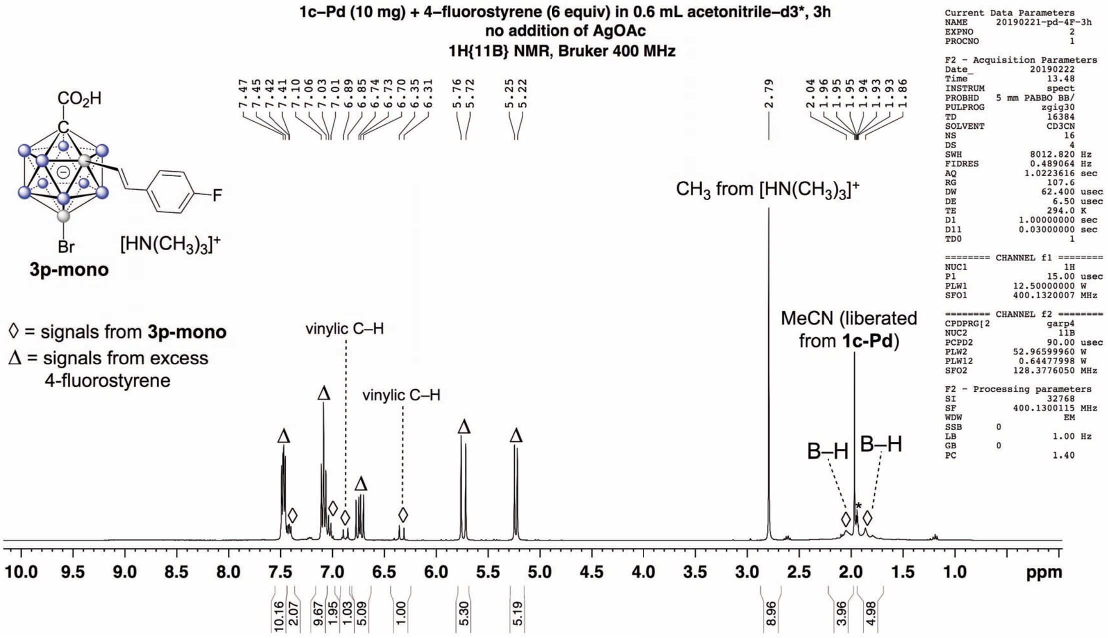

Figure S2. $^{1}H\{^{11}B\}$ NMR spectrum of the reaction mixture **1c-Pd** + 4-fluorostyrene (6.0 equiv) in MeCN-d_3 after 3 h at 25 °C (400 MHz, 23 °C).

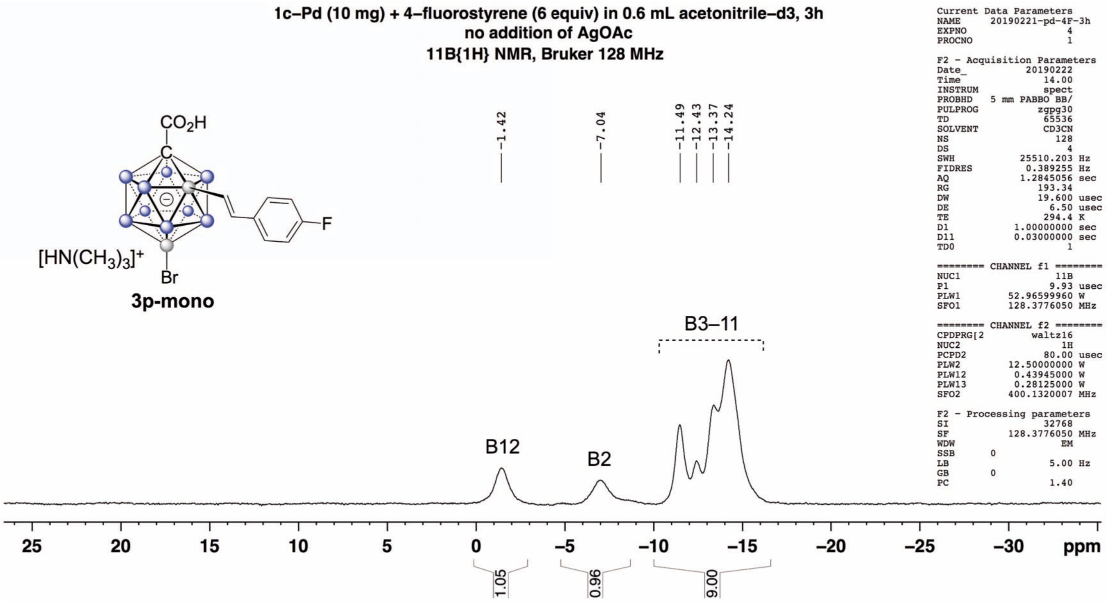

Figure S3. $^{11}B\{^{1}H\}$ NMR spectrum of the reaction mixture **1c-Pd** + 4-fluorostyrene (6.0 equiv) in MeCN-d_3 after 3 h at 25 °C (128 MHz for ^{11}B, 23 °C).

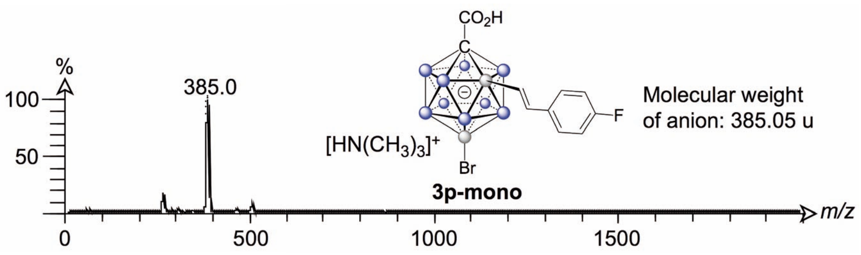

Figure S4. Full-range (–)-ESI mass spectrum of the reaction mixture **1c-Pd** + 4-fluorostyrene (6.0 equiv) in MeCN-d_3 after 3 h at 25 °C.

Proposed mechanism for the penta-alkenylation

A plausible mechanism for the penta-alkenylation is displayed in Scheme S1. Binding of the carboxylate group of **1** to Pd(II) affords the initial intermediate **IM-a**. Cyclometalation–deprotonation then gives palladacycle **1-Pd** with a direct B–Pd bond. Intermediates **IM-b**/**IM-c** are formed by alkene coordination/insertion, which is followed by β-hydride elimination to furnish Pd–H complex **IM-d**. Subsequent reaction with AgOAc leads to **IM-a'**, which contains one alkenyl substituent. Based on the above control experiment starting from **1c-Pd** and excess 4-fluorostyrene in the absence of AgOAc, it seems likely that the steps until formation of **IM-d** occur fast

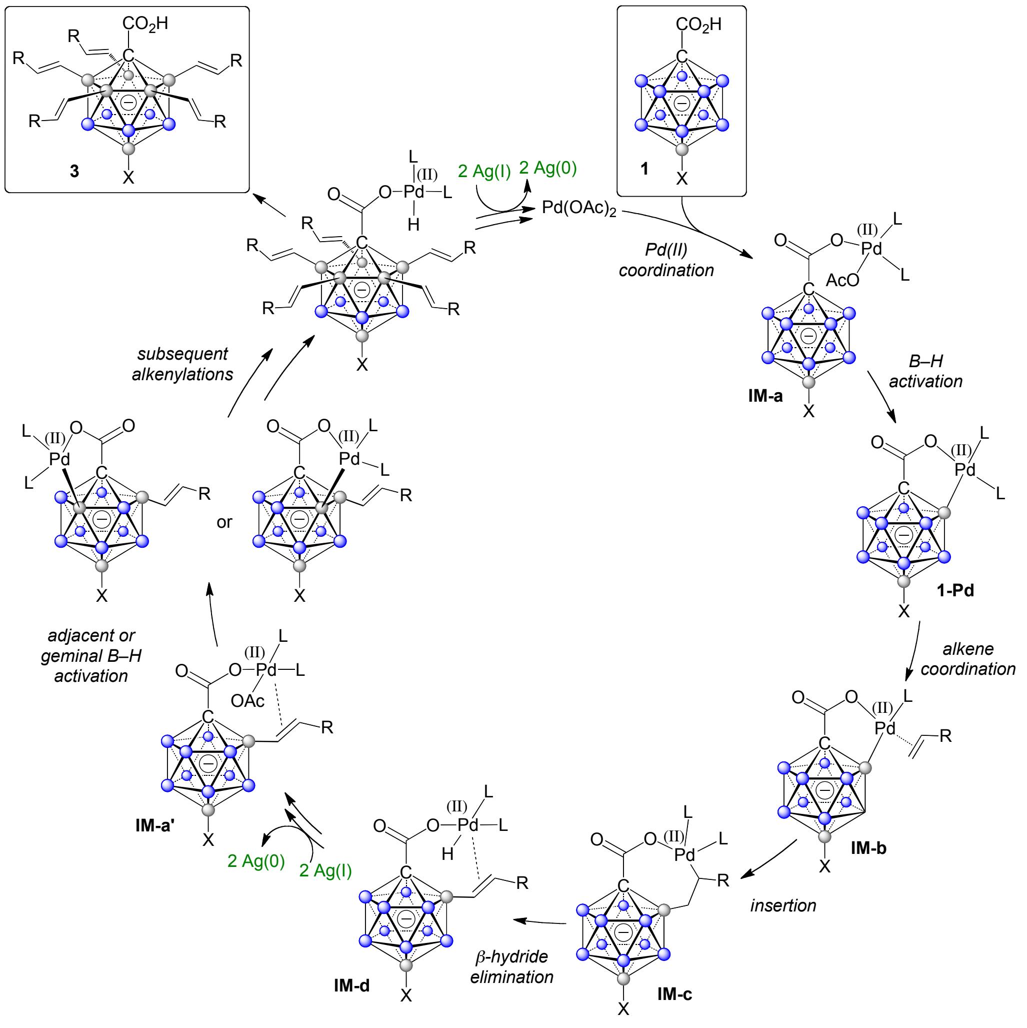

Scheme S1. Proposed mechanism for the five-fold B–H activation/B–C coupling cascade; L = solvent molecule or AcO^-.

and that AgOAc is necessary to promote subsequent coupling steps. Currently we believe that base-assisted elimination of H^+/L (L = AcO^- or solvent) from **IM-d** affords **IM-e** with a formal Pd(0) center (Scheme S2); formation of H_2 was not observed in any of the experiments of this study. Oxidation of **IM-e** by two equivalents of Ag(I) re-establishes the Pd(II) oxidation state, thereby enabling the system to undergo further B–H activation/alkenylation processes. They are assumed to occur in a similar manner until the final product **3** and Pd(II) are liberated.

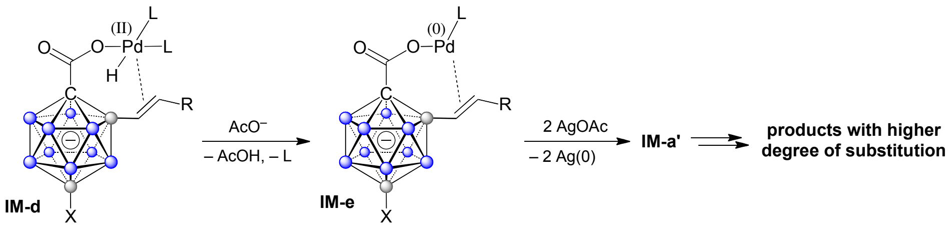

Scheme S2. Putative Pd(0) intermediate **IM-e** formed by deprotonation/ligand dissociation from **IM-d**, followed by reoxidation of the Pd center; L = solvent molecule or AcO^-.

III X-ray Crystallography

Crystal structure of 3b (CCDC 1886700)

Compound **3b** (15 mg, 0.018 mmol) was dissolved in acetone (0.5 mL) in a 1 mL glass vial. The resulting colorless solution was filtered into a 18 cm long NMR tube and layered with hexanes (1 mL). Colorless crystals of the composition [Et_4N][**3b**]·acetone suitable for X-ray diffraction grew within 3 d at room temperature.

```
Bond precision:   C-C = 0.0073 A          Wavelength=1.34139

Cell:             a=11.9981(3)     b=20.7041(4)      c=21.3976(4)
                  alpha=90         beta=92.910(1)    gamma=90
Temperature:      170 K

                 Calculated                    Reported
Volume           5308.5(2)                     5308.52(19)
Space group      P 21/n                        P 1 21/n 1
Hall group       -P 2yn                        -P 2yn
Moiety formula   C42 H42 B11 O2, C8 H20 N,     C42 H42 B11 O2, C3 H6 O,
                 C3 H6 O                       C8 H20 N
Sum formula      C53 H68 B11 N O3              C53 H68 B11 N O3
Mr               885.99                        885.99
Dx,g cm-3        1.109                         1.109
Z                4                             4
Mu (mm-1)        0.305                         0.308
F000             1888.0                        1888.0
F000'            1891.50
h,k,lmax         14,25,26                      14,25,26
Nref             10119                         10024
Tmin,Tmax        0.982,0.991                   0.638,0.751
Tmin'            0.976

Correction method= # Reported T Limits: Tmin=0.638 Tmax=0.751
AbsCorr = MULTI-SCAN

Data completeness= 0.991          Theta(max)= 54.945

R(reflections)= 0.0991( 5609)     wR2(reflections)= 0.3133( 10024)

S = 1.030                  Npar= 662
```

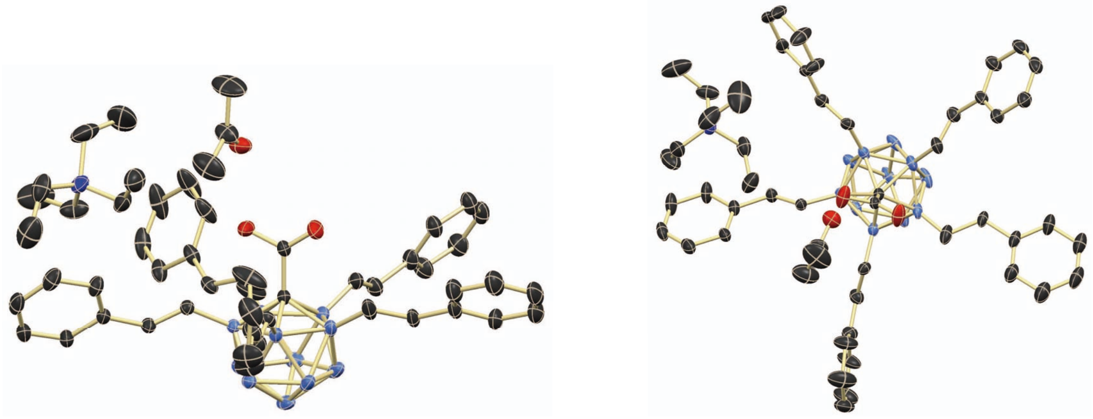

Figure S5. Crystal structure of [Et$_4$N][**3b**]·acetone (left: side view; right: view along C(cage)–B12 axis); hydrogen atoms are omitted for clarity; 30% displacement ellipsoids; one of the styrene substituents is disordered, and only one set of the disordered atoms is shown.

Crystal structure of 4b (CCDC 1886699)

Compound **4b** (10 mg, 0.013 mmol) was dissolved in acetone (0.4 mL) in a 1 mL glass vial. The resulting colorless solution was filtered into a 18 cm long NMR tube and layered with hexanes (1 mL). Colorless crystals of the composition [Et_4N][**4b**] suitable for X-ray diffraction grew within 3 d at room temperature.

Bond precision: C-C = 0.0066 A Wavelength=1.34139

Cell:	a=23.5746(11)	b=18.8529(6)	c=22.1333(6)
	alpha=90	beta=90	gamma=90
Temperature:	296 K		

	Calculated	Reported
Volume	9837.1(6)	9837.1(6)
Space group	I b a m	I b a m
Hall group	-I 2 2c	-I 2 2c
Moiety formula	C41 H42 B11, C8 H20 N [+ solvent]	C8 H20 N, C41 H42 B11
Sum formula	C49 H62 B11 N [+ solvent]	C49 H62 B11 N
Mr	783.91	783.90
Dx,g cm-3	1.059	1.059
Z	8	8
Mu (mm-1)	0.263	0.267
F000	3344.0	3344.0
F000'	3349.68	
h,k,lmax	28,23,27	28,23,27
Nref	4844	4797
Tmin,Tmax	0.962,0.987	0.563,0.751
Tmin'	0.948	

Correction method= # Reported T Limits: Tmin=0.563 Tmax=0.751
AbsCorr = MULTI-SCAN

Data completeness= 0.990 Theta(max)= 55.098

R(reflections)= 0.0839(3107) wR2(reflections)= 0.2591(4797)

S = 1.037 Npar= 337

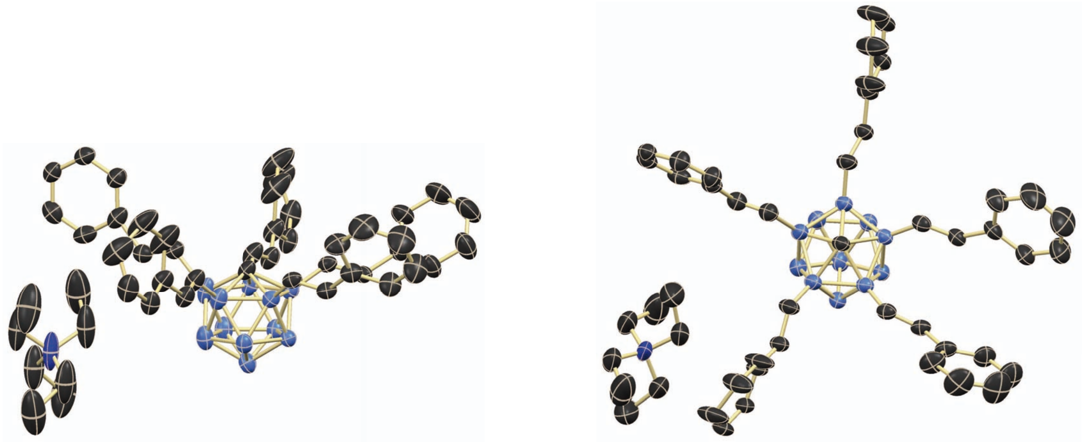

Figure S6. Crystal structure of [Et_4N][**4b**] (left: side view; right: view along C(cage)–B12 axis); hydrogen atoms and disordered parts are omitted for clarity; 30% displacement ellipsoids.

The Alert level B message for this structure indicates a short H···H contact and is the result of some disorder in this structure. While the disorder concerning one of the vinyl groups (C18/19) could be resolved successfully, the related disorder of the neighbouring phenyl group (C12–17) could not. The short contact is a consequence of that.

Crystal structure of 1c-Pd (CCDC 1886701)

Compound **1c-Pd** (15 mg, 0.029 mmol) was dissolved in acetonitrile (0.5 mL) in a 2 mL glass vial. The resulting clear solution was left to evaporate slowly at room temperature. Colorless crystals of the composition [Me_3NH][**1c-Pd**] suitable for X-ray diffraction grew within 4 d.

Bond precision: C-C = 0.0173 A   Wavelength=0.71073

Cell:	a=8.7468(9)	b=10.0535(7)	c=26.411(3)
	alpha=90	beta=95.913(8)	gamma=90
Temperature:	293 K		

	Calculated	Reported
Volume	2310.1(4)	2310.1(4)
Space group	P 21/c	P 1 21/c 1
Hall group	-P 2ybc	-P 2ybc
Moiety formula	C6 H15 B11 Br N2 O2 Pd, C3 H10 N	C6 H15 B11 Br N2 O2 Pd, C3 H10 N
Sum formula	C9 H25 B11 Br N3 O2 Pd	C9 H25 B11 Br N3 O2 Pd
Mr	512.53	512.54
Dx,g cm-3	1.474	1.474
Z	4	4
Mu (mm-1)	2.541	2.541
F000	1008.0	1008.0
F000'	1003.09	
h,k,lmax	10,12,31	10,12,31
Nref	4224	4195
Tmin,Tmax	0.430,0.776	0.442,1.000
Tmin'	0.341	

Correction method= # Reported T Limits: Tmin=0.442 Tmax=1.000
AbsCorr = MULTI-SCAN

Data completeness= 0.993   Theta(max)= 25.350

R(reflections)= 0.0974(2903)   wR2(reflections)= 0.2545(4195)

S = 1.054   Npar= 249

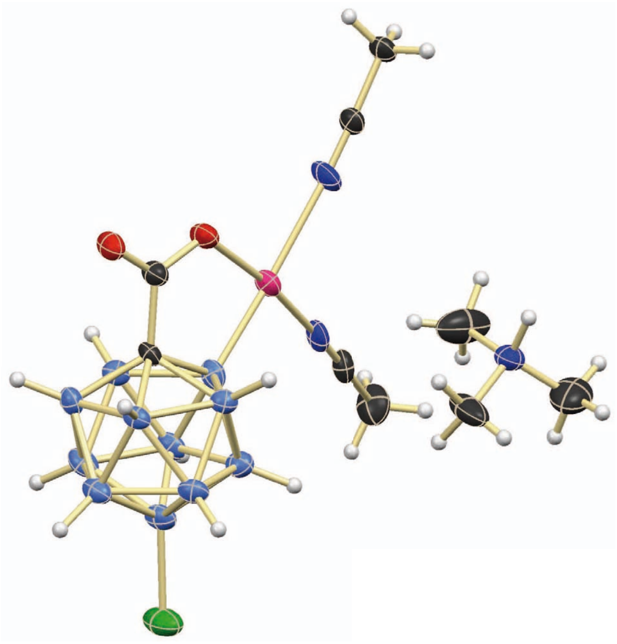

Figure S7. Crystal structure of [Me_3NH][**1c-Pd**]; 30% displacement ellipsoids.

IV References

[1] J. Plešek, T. Jelínek, E. Drdákova, S. Hermánek, B. Štibr, *Collect. Czech. Chem. Commun.*, **1984**, *49*, 1559–1562.

[2] C. A. Reed, *Acc. Chem. Res*. **2010**, *43*, 121–128.

[3] M. Valášek, J. Štursa, R. Pohl, J. Michl, *Inorg. Chem*. **2010**, *49*, 10247–10254.

[4] Y. Shen, Y. Pan, K. Zhang, X. Liang, J. Liu, B. Spingler, S. Duttwyler, *Dalton Trans.* **2017**, *46*, 3135–3140.

[5] T. Jelínek, P. Baldwin, W. R. Scheidt, C. A. Reed, *Inorg. Chem*. **1993**, *32*, 1982–1990.

[6] B. Grüner, Z. Janoušek, B. T. King, J. N. Woodford, C. H. Wang, V. Všetecka, J. Michl, *J. Am. Chem. Soc*. **1999**, *121*, 3122–3126.

[7] K. Zhang, Y. Shen, J. Liu, B. Spingler, S. Duttwyler, *Chem. Commun.* **2018**, *54*, 1698–1701.

[8] K. R. Prasad, S. P. Reddy, K. S. Babu, J. M. Rao, *Helv. Chim. Acta* **2015**, *98*, 143–147.

[9] J. R. Holmes, D. Kivelson, W. C. Drinkard, *J. Chem. Phys.* **1962**, *37*, 150–152; a more recent summary is available online from the Sigma-Aldrich company: https://www.sigmaaldrich.com/content/dam/sigma-aldrich/docs/Aldrich/General_Information/double_water_peaks.pdf

V NMR Spectra

Following on pp. S56–S201

VI Mass Spectra

Following on pp. S202–S236

1–COOH–12–Cl–CB11 product 30 mg in 0.6 ml acetone–d6 *
1H{11B} NMR, 500 MHz, 23 C

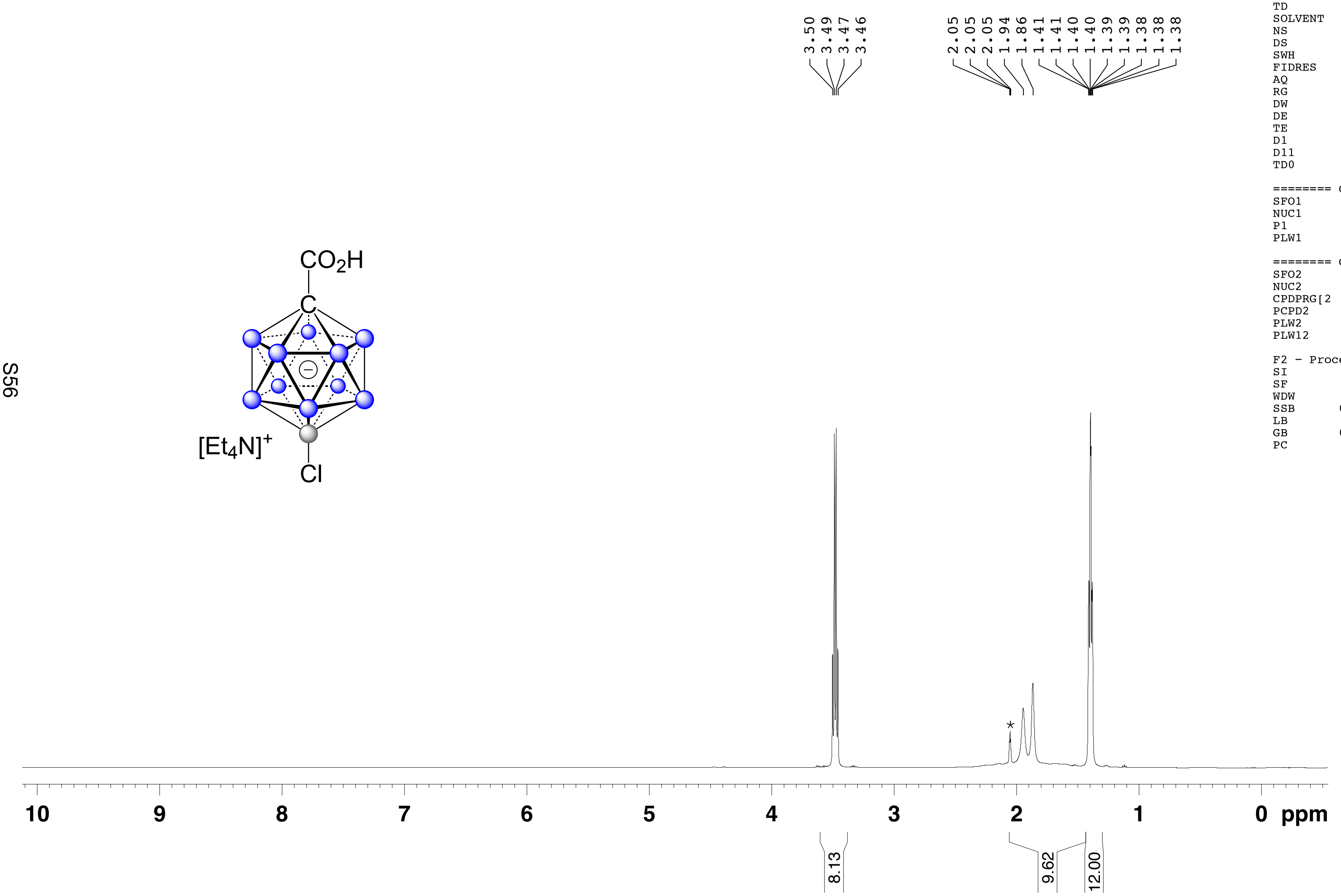


```
Current Data Parameters
NAME          12-Cl-acid
EXPNO                  1
PROCNO                 1

F2 - Acquisition Parameters
Date_           20180823
Time               19.39
INSTRUM            spect
PROBHD   5 mm PABBO BB-
PULPROG          zgig30
TD                 65536
SOLVENT          Acetone
NS                    16
DS                     0
SWH            12500.000 Hz
FIDRES          0.190735 Hz
AQ             2.6214399 sec
RG                    57
DW                40.000 usec
DE                  6.50 usec
TE                 297.2 K
D1            5.00000000 sec
D11           0.03000000 sec
TD0                    1

======== CHANNEL f1 ========
SFO1        500.1335009 MHz
NUC1                  1H
P1                 11.70 usec
PLW1         19.00000000 W

======== CHANNEL f2 ========
SFO2        160.4615690 MHz
NUC2                 11B
CPDPRG[2            garp
PCPD2             100.00 usec
PLW2         95.00000000 W
PLW12         1.63030005 W

F2 - Processing parameters
SI                 65536
SF          500.1300089 MHz
WDW                   EM
SSB      0
LB                  1.00 Hz
GB       0
PC                  1.00
```

1–COOH–12–Cl–CB11 product 30 mg in 0.6 ml acetone–d6
11B NMR, 160 MHz, 23 C

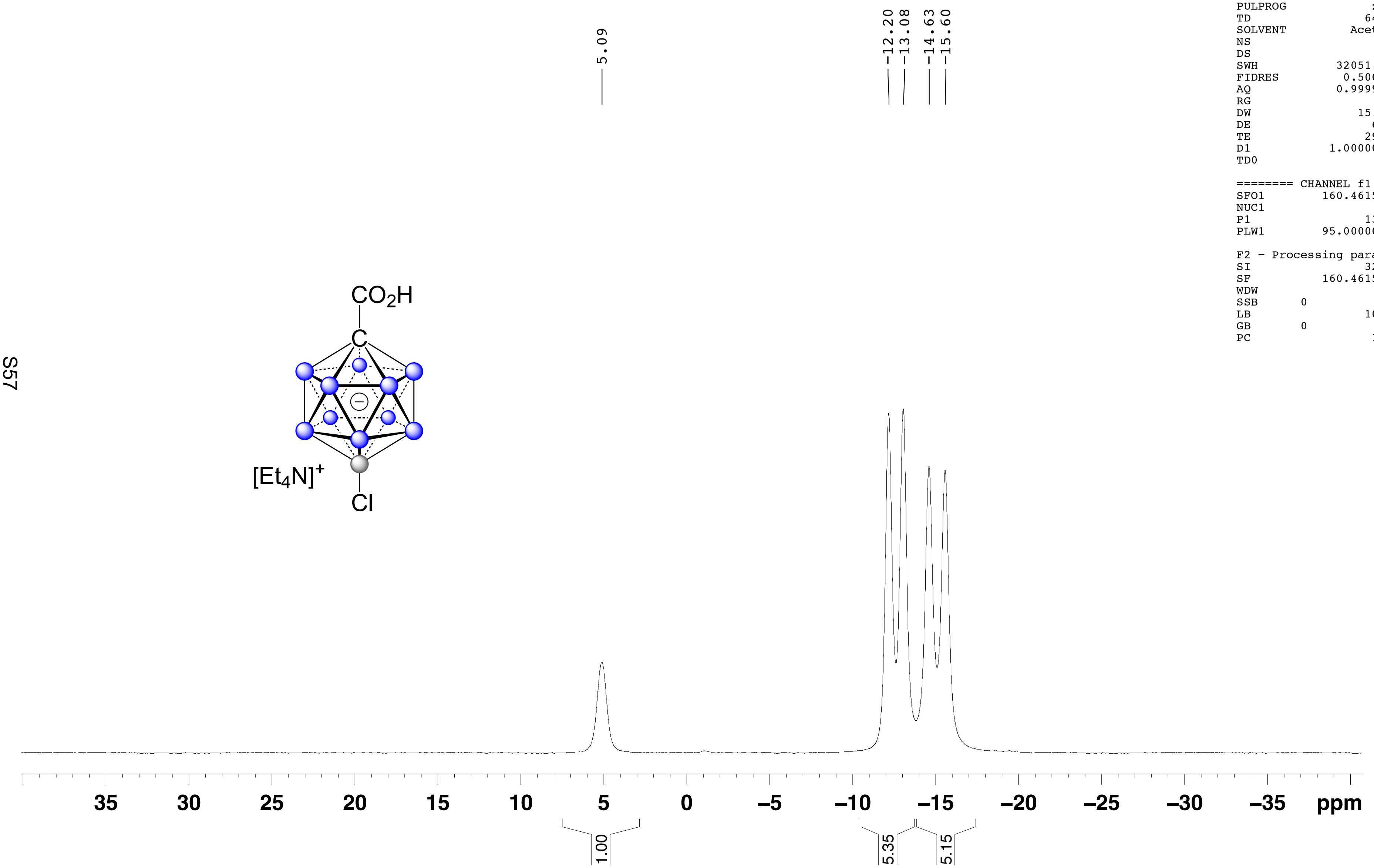


```
Current Data Parameters
NAME          12-Cl-acid
EXPNO                  2
PROCNO                 1

F2 - Acquisition Parameters
Date_           20180823
Time               19.41
INSTRUM            spect
PROBHD   5 mm PABBO BB-
PULPROG             zg30
TD                 64098
SOLVENT          Acetone
NS                    32
DS                     0
SWH            32051.281 Hz
FIDRES          0.500036 Hz
AQ             0.9999288 sec
RG                   203
DW                15.600 usec
DE                  6.50 usec
TE                 296.9 K
D1            1.00000000 sec
TD0                    1

======== CHANNEL f1 ========
SFO1         160.4615792 MHz
NUC1                 11B
P1                 13.10 usec
PLW1         95.00000000 W

F2 - Processing parameters
SI                 32768
SF           160.4615790 MHz
WDW                   EM
SSB      0
LB                 10.00 Hz
GB       0
PC                  1.40
```

1–COOH–12–Cl–CB11 product 30 mg in 0.6 ml acetone–d6
11B{1H} NMR, 160 MHz, 23 C

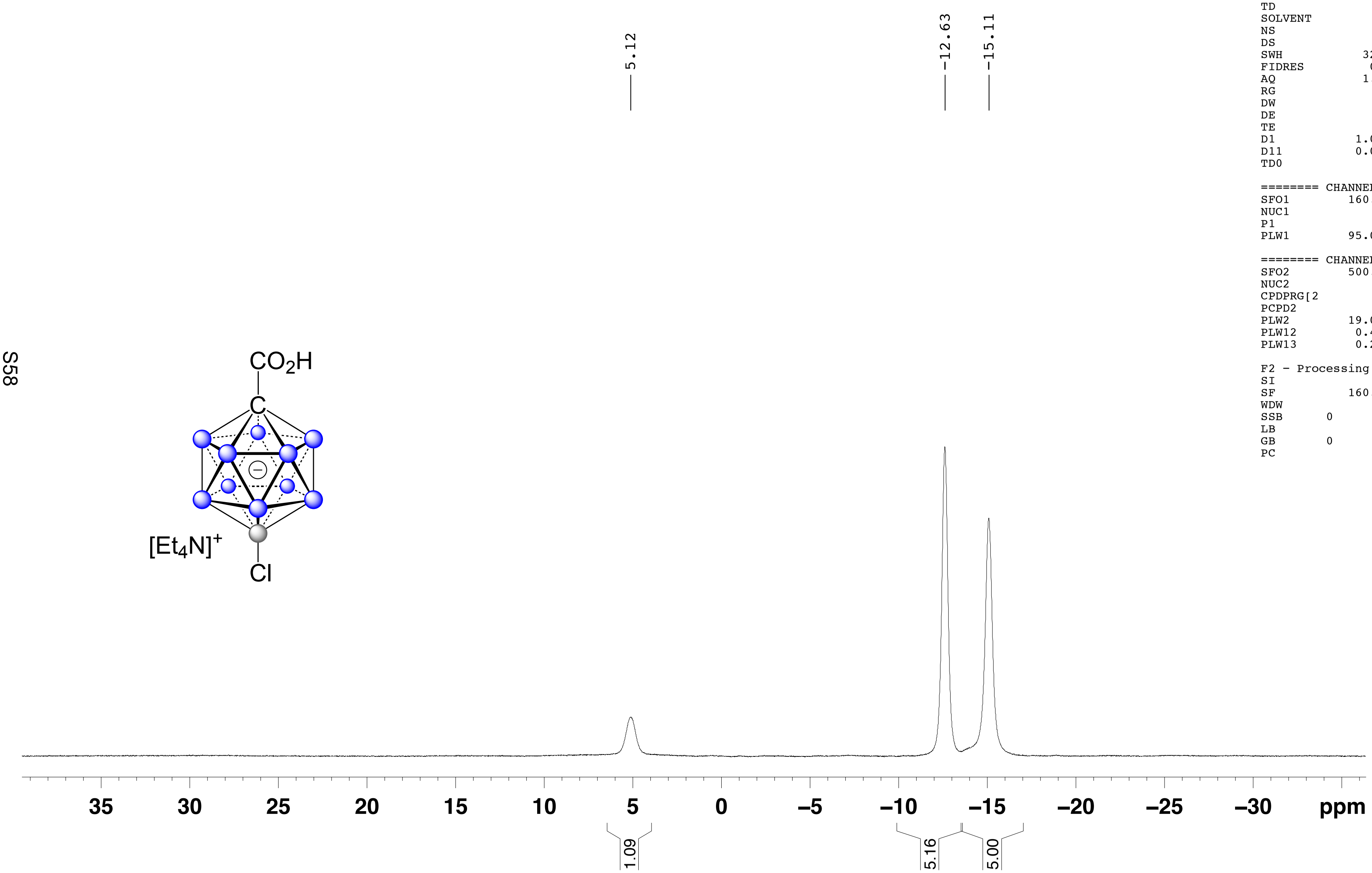


```
Current Data Parameters
NAME          12-Cl-acid
EXPNO                  3
PROCNO                 1

F2 - Acquisition Parameters
Date_           20180823
Time               19.43
INSTRUM            spect
PROBHD   5 mm PABBO BB-
PULPROG          zgpg30
TD                 65536
SOLVENT          Acetone
NS                    32
DS                     0
SWH            32051.281 Hz
FIDRES          0.489064 Hz
AQ             1.0223616 sec
RG                   203
DW                15.600 usec
DE                  6.50 usec
TE                 297.1 K
D1            1.00000000 sec
D11           0.03000000 sec
TD0                    1

======== CHANNEL f1 ========
SFO1         160.4615790 MHz
NUC1                 11B
P1                 13.10 usec
PLW1         95.00000000 W

======== CHANNEL f2 ========
SFO2         500.1325007 MHz
NUC2                  1H
CPDPRG[2         waltz16
PCPD2              80.00 usec
PLW2         19.00000000 W
PLW12         0.40639001 W
PLW13         0.26008999 W

F2 - Processing parameters
SI                 32768
SF           160.4615790 MHz
WDW                   EM
SSB      0
LB                  1.00 Hz
GB       0
PC                  1.40
```

1–COOH–12–Cl–CB11 product 30 mg in 0.6 ml acetone–d6 *
13C{1H} NMR, 125 MHz, 23 C

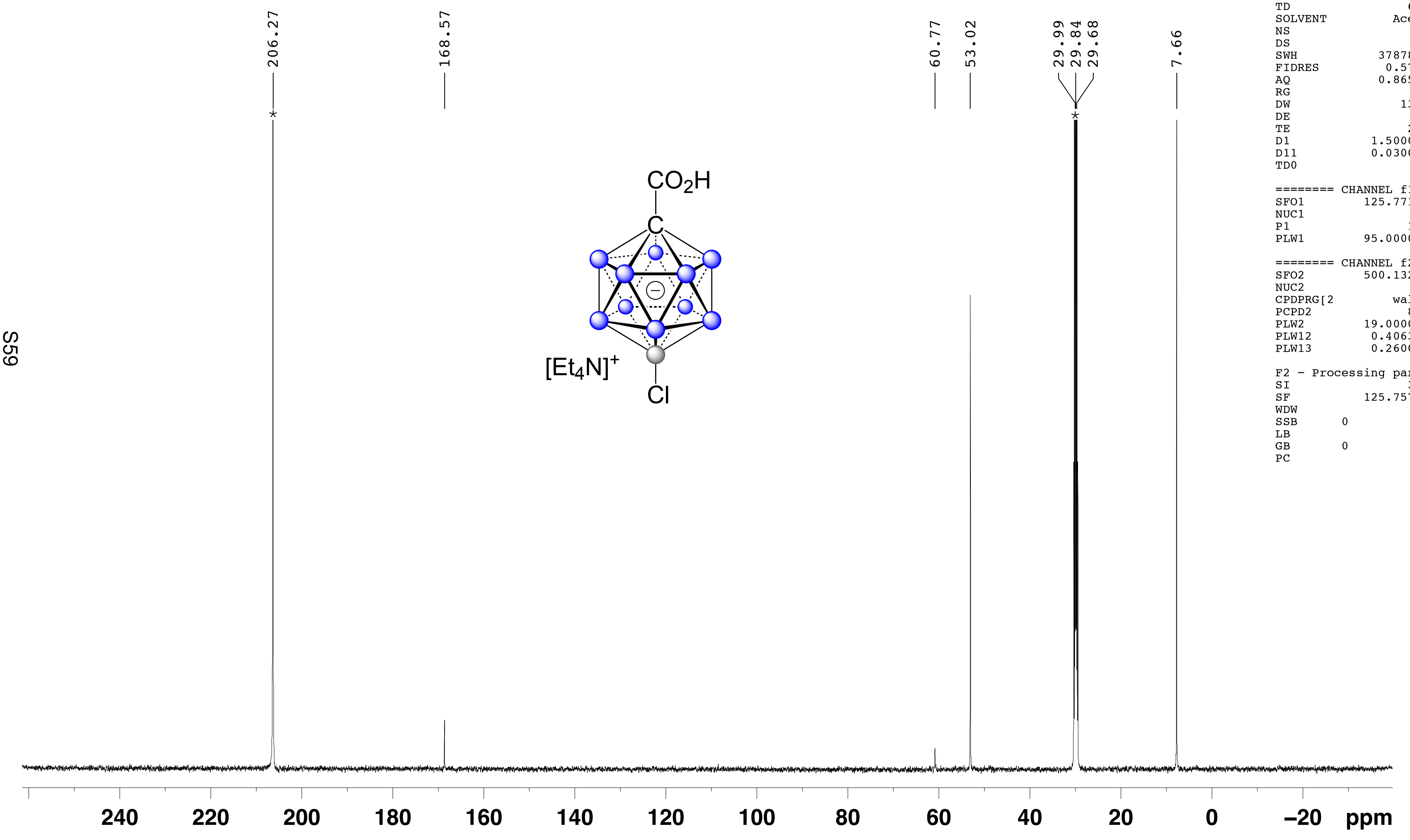


```
Current Data Parameters
NAME          12-Cl-acid
EXPNO                  4
PROCNO                 1

F2 - Acquisition Parameters
Date_           20180823
Time               20.26
INSTRUM            spect
PROBHD   5 mm PABBO BB-
PULPROG          zgpg30
TD                 65536
SOLVENT          Acetone
NS                  1024
DS                     4
SWH            37878.789 Hz
FIDRES          0.577984 Hz
AQ             0.8650752 sec
RG                   203
DW                13.200 usec
DE                  6.50 usec
TE                 297.1 K
D1            1.50000000 sec
D11           0.03000000 sec
TD0                    1

======== CHANNEL f1 ========
SFO1         125.7716224 MHz
NUC1                 13C
P1                 10.50 usec
PLW1         95.00000000 W

======== CHANNEL f2 ========
SFO2         500.1320005 MHz
NUC2                  1H
CPDPRG[2         waltz16
PCPD2              80.00 usec
PLW2         19.00000000 W
PLW12         0.40639001 W
PLW13         0.26008999 W

F2 - Processing parameters
SI                 32768
SF           125.7576833 MHz
WDW                   EM
SSB      0
LB                  3.00 Hz
GB       0
PC                  1.40
```

12−Br−1−COOH−Pd product 30 mg in 0.6 ml acetonitrile−d3 *
1H{11B} NMR, 500 MHz, 23 C

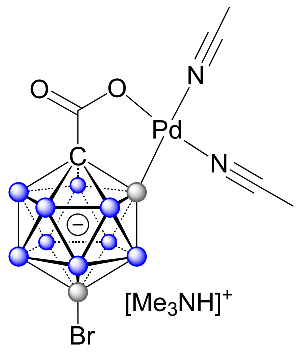

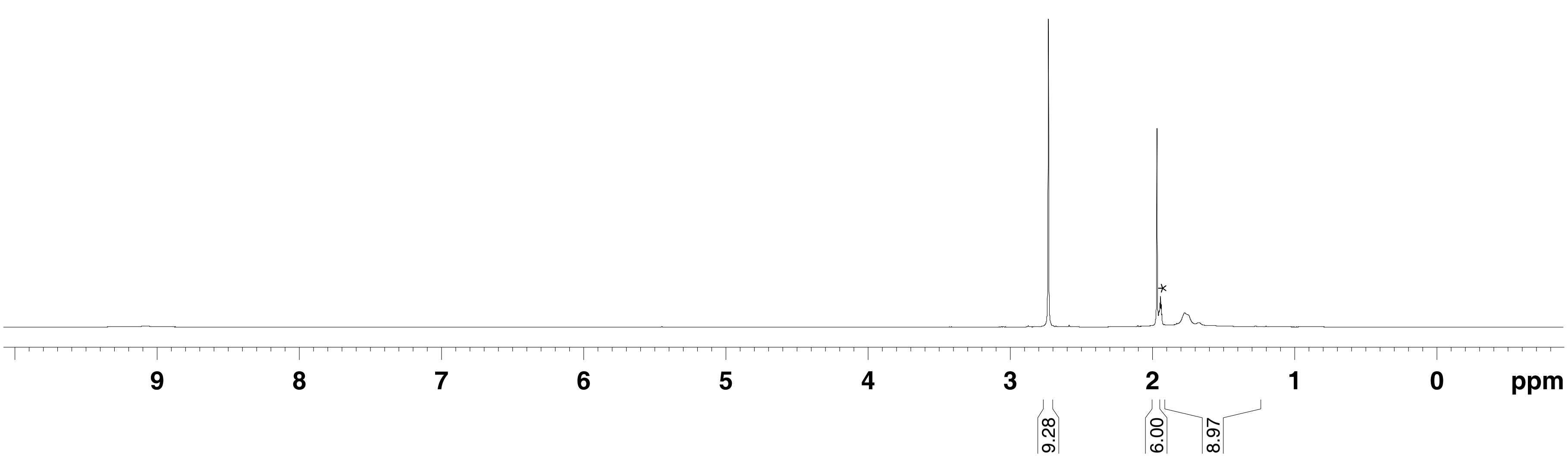


```
Current Data Parameters
NAME     12-Br-1-COOH-Pd-ACN
EXPNO                  1
PROCNO                 1

F2 - Acquisition Parameters
Date_           20170426
Time                2.20
INSTRUM            spect
PROBHD   5 mm PABBO BB-
PULPROG          zgig30
TD                65536
SOLVENT           CD3CN
NS                   16
DS                    0
SWH           12500.000 Hz
FIDRES         0.190735 Hz
AQ            2.6214399 sec
RG                  114
DW               40.000 usec
DE                 6.50 usec
TE                296.6 K
D1           5.00000000 sec
D11          0.03000000 sec

======== CHANNEL f1 ========
NUC1                 1H
P1                11.70 usec
PLW1        19.00000000 W
SFO1        500.1335009 MHz

======== CHANNEL f2 ========
CPDPRG[2           garp
NUC2                11B
PCPD2            100.00 usec
PLW2        95.00000000 W
PLW12        1.63030005 W
SFO2        160.4615690 MHz

F2 - Processing parameters
SI                65536
SF          500.1300157 MHz
WDW                  EM
SSB      0
LB                 1.00 Hz
GB       0
PC                 1.00
```

12–Br–1–COOH–Pd product 30 mg in 0.6 ml acetonitrile–d3
11B NMR, 160 MHz, 23 C

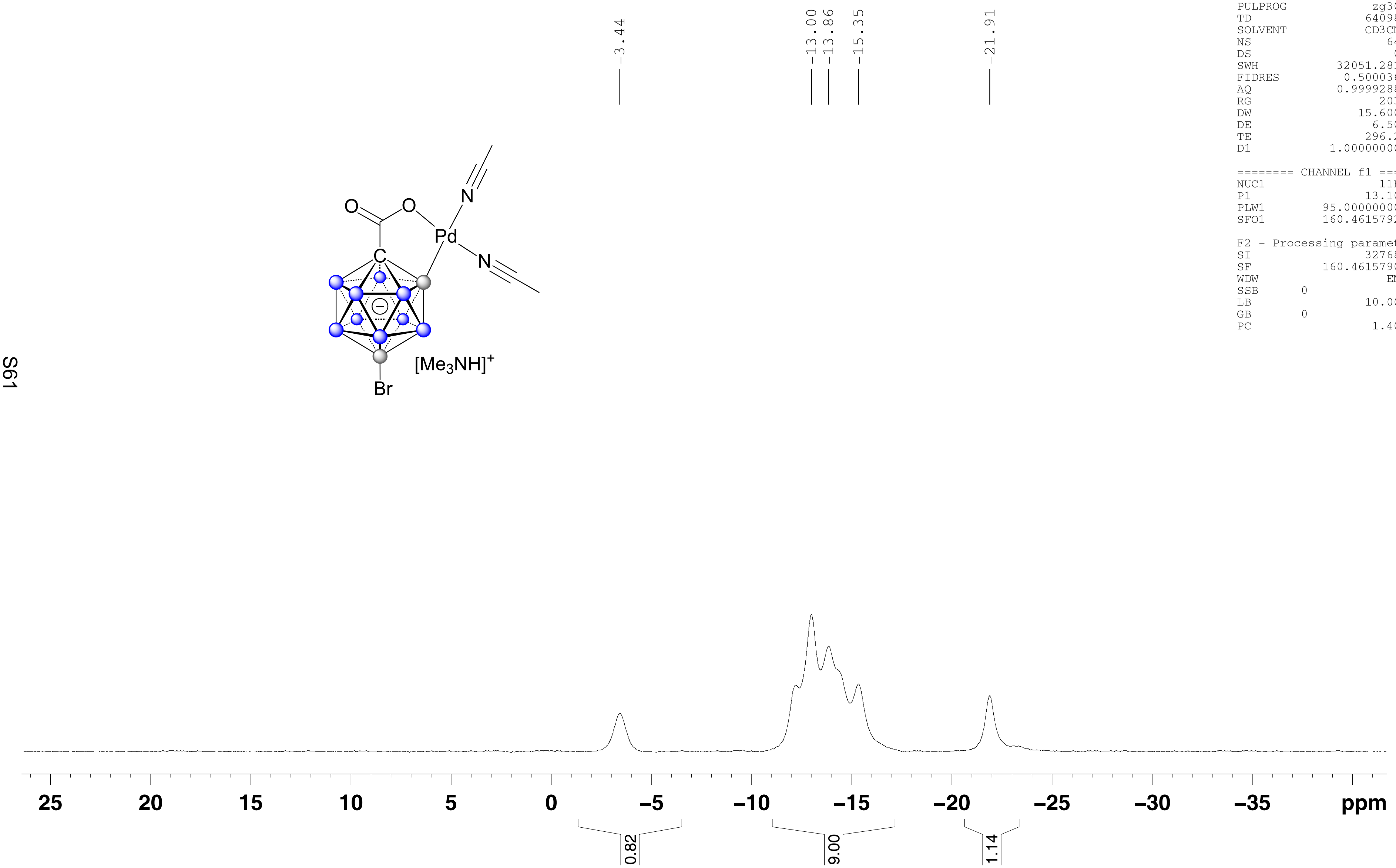


```
Current Data Parameters
NAME     12-Br-1-COOH-Pd-ACN
EXPNO                     2
PROCNO                    1

F2 - Acquisition Parameters
Date_           20170426
Time                2.23
INSTRUM            spect
PROBHD   5 mm PABBO BB-
PULPROG             zg30
TD                 64098
SOLVENT            CD3CN
NS                    64
DS                     0
SWH            32051.281 Hz
FIDRES          0.500036 Hz
AQ             0.9999288 sec
RG                   203
DW                15.600 usec
DE                  6.50 usec
TE                 296.2 K
D1            1.00000000 sec

======== CHANNEL f1 ========
NUC1                 11B
P1                 13.10 usec
PLW1         95.00000000 W
SFO1         160.4615792 MHz

F2 - Processing parameters
SI                 32768
SF           160.4615790 MHz
WDW                   EM
SSB      0
LB                 10.00 Hz
GB       0
PC                  1.40
```

12–Br–1–COOH–Pd product 30 mg in 0.6 ml acetonitrile–d3
11B{1H} NMR, 160 MHz, 23 C

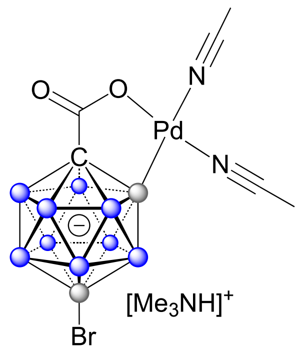

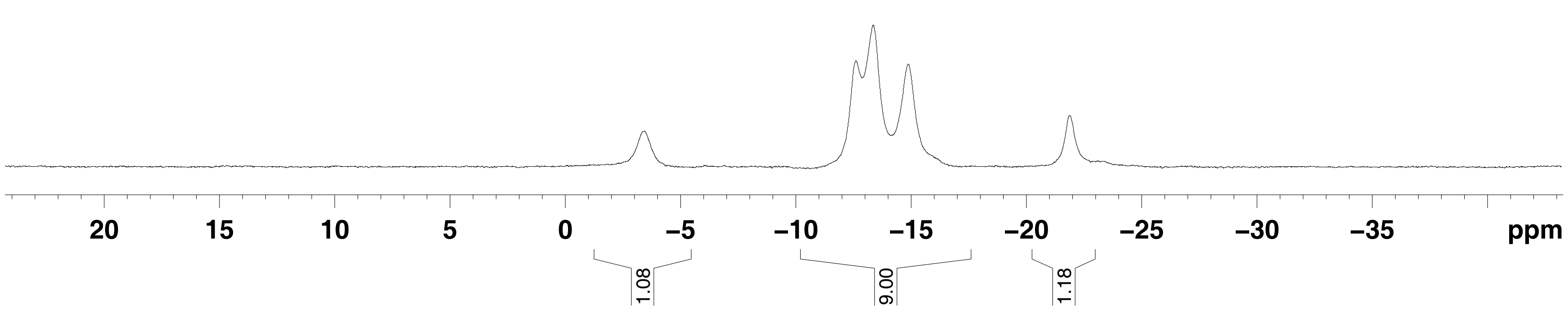


```
Current Data Parameters
NAME     12-Br-1-COOH-Pd-ACN
EXPNO                  3
PROCNO                 1

F2 - Acquisition Parameters
Date_          20170426
Time               2.26
INSTRUM           spect
PROBHD   5 mm PABBO BB-
PULPROG          zgpg30
TD                65536
SOLVENT           CD3CN
NS                   64
DS                    0
SWH           32051.281 Hz
FIDRES         0.489064 Hz
AQ            1.0223616 sec
RG                  203
DW               15.600 usec
DE                 6.50 usec
TE                296.4 K
D1           1.00000000 sec
D11          0.03000000 sec

======== CHANNEL f1 ========
NUC1                11B
P1                13.10 usec
PLW1        95.00000000 W
SFO1        160.4615790 MHz

======== CHANNEL f2 ========
CPDPRG[2        waltz16
NUC2                 1H
PCPD2             80.00 usec
PLW2        19.00000000 W
PLW12        0.40639001 W
PLW13        0.26008999 W
SFO2        500.1325007 MHz

F2 - Processing parameters
SI                32768
SF          160.4615790 MHz
WDW                  EM
SSB      0
LB                 5.00 Hz
GB       0
PC                 1.40
```

12−Br−1−COOH−Pd product 30 mg in 0.6 ml acetonitrile−d3*
13C{1H} NMR, 126 MHz, 23 C

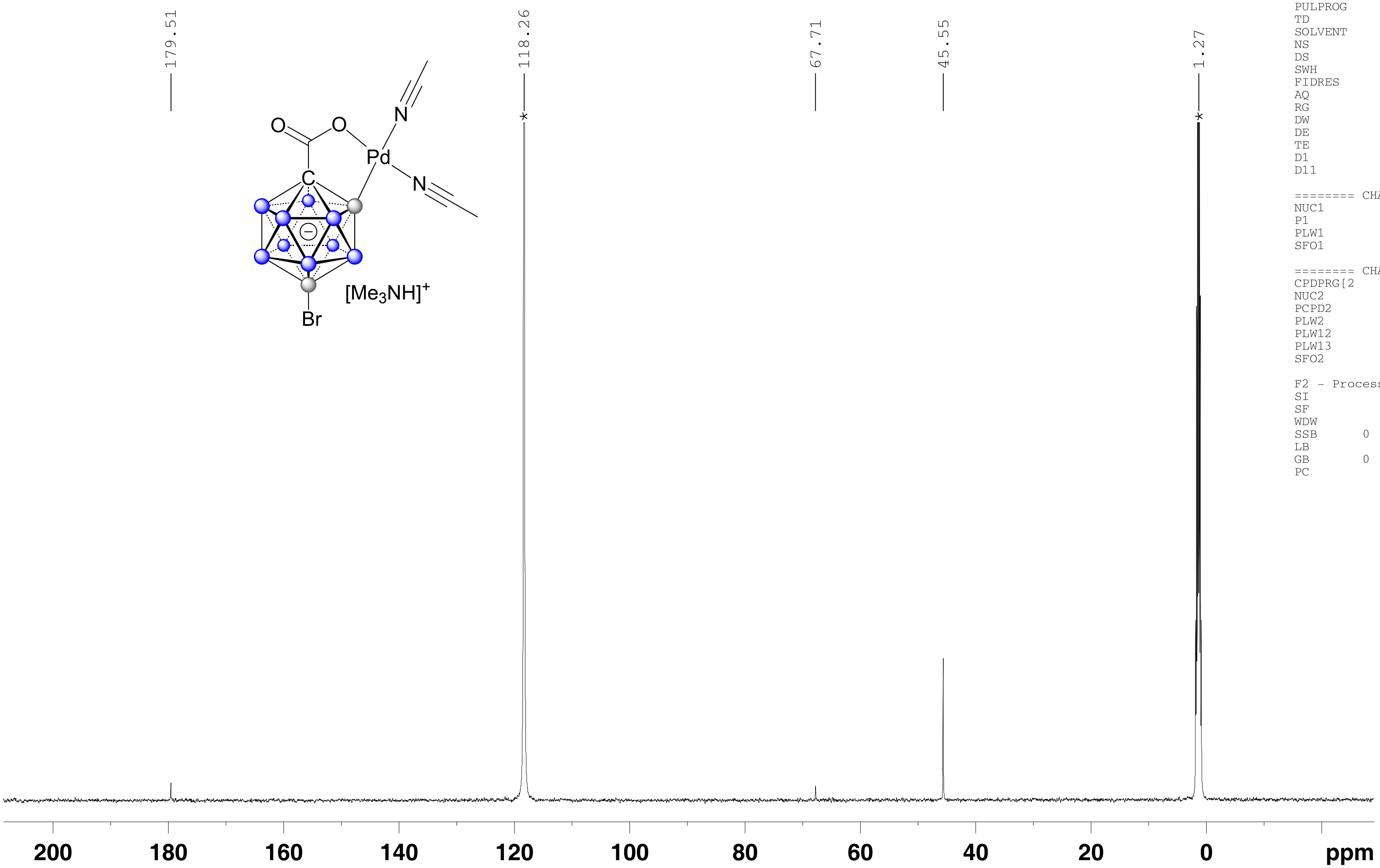


```
Current Data Parameters
NAME     12-Br-1-COOH-Pd-ACN
EXPNO                      4
PROCNO                     1

F2 - Acquisition Parameters
Date_             20170426
Time                  3.51
INSTRUM              spect
PROBHD   5 mm PABBO BB-
PULPROG            zgpg30
TD                  65536
SOLVENT             CD3CN
NS                   2048
DS                      4
SWH           37878.789 Hz
FIDRES         0.577984 Hz
AQ            0.8650752 sec
RG                    203
DW               13.200 usec
DE                 6.50 usec
TE                296.6 K
D1           1.50000000 sec
D11          0.03000000 sec

======== CHANNEL f1 ========
NUC1                  13C
P1                  10.50 usec
PLW1          95.00000000 W
SFO1          125.7716224 MHz

======== CHANNEL f2 ========
CPDPRG[2          waltz16
NUC2                   1H
PCPD2               80.00 usec
PLW2          19.00000000 W
PLW12          0.40639001 W
PLW13          0.26008999 W
SFO2          500.1320005 MHz

F2 - Processing parameters
SI                  32768
SF            125.7576724 MHz
WDW                    EM
SSB      0
LB                   6.00 Hz
GB       0
PC                   1.40
```

1–COOH–12–Me–CB11 product 30 mg in 0.6 ml acetone–d6 *
1H{11B}NMR, 500 MHz, 23 C

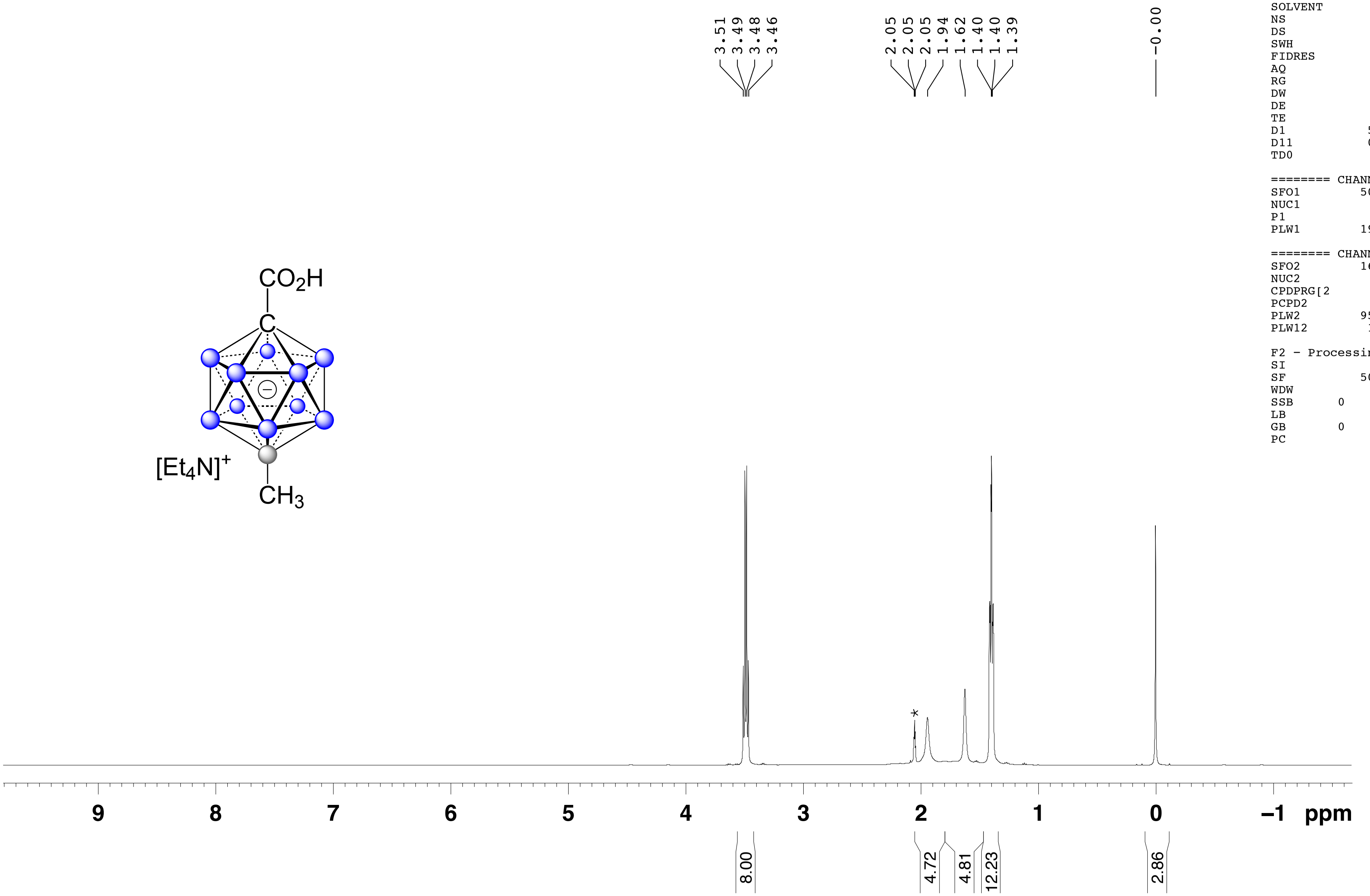


```
Current Data Parameters
NAME          12-Me-acid
EXPNO                  1
PROCNO                 1

F2 - Acquisition Parameters
Date_           20180824
Time                6.21
INSTRUM            spect
PROBHD   5 mm PABBO BB-
PULPROG          zgig30
TD                 65536
SOLVENT          Acetone
NS                    16
DS                     0
SWH            12500.000 Hz
FIDRES          0.190735 Hz
AQ             2.6214399 sec
RG                    64
DW                40.000 usec
DE                  6.50 usec
TE                 296.0 K
D1            5.00000000 sec
D11           0.03000000 sec
TD0                    1

======== CHANNEL f1 ========
SFO1         500.1335009 MHz
NUC1                  1H
P1                 11.70 usec
PLW1         19.00000000 W

======== CHANNEL f2 ========
SFO2         160.4615690 MHz
NUC2                 11B
CPDPRG[2            garp
PCPD2             100.00 usec
PLW2         95.00000000 W
PLW12         1.63030005 W

F2 - Processing parameters
SI                 65536
SF           500.1300087 MHz
WDW                   EM
SSB      0
LB                  1.00 Hz
GB       0
PC                  1.00
```

1–COOH–12–Me–CB11 product 30 mg in 0.6 ml acetone–d6
11B NMR, 160 MHz, 23 C

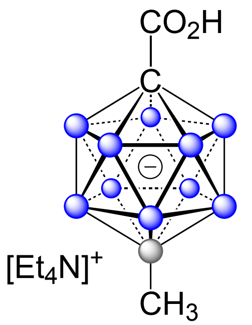

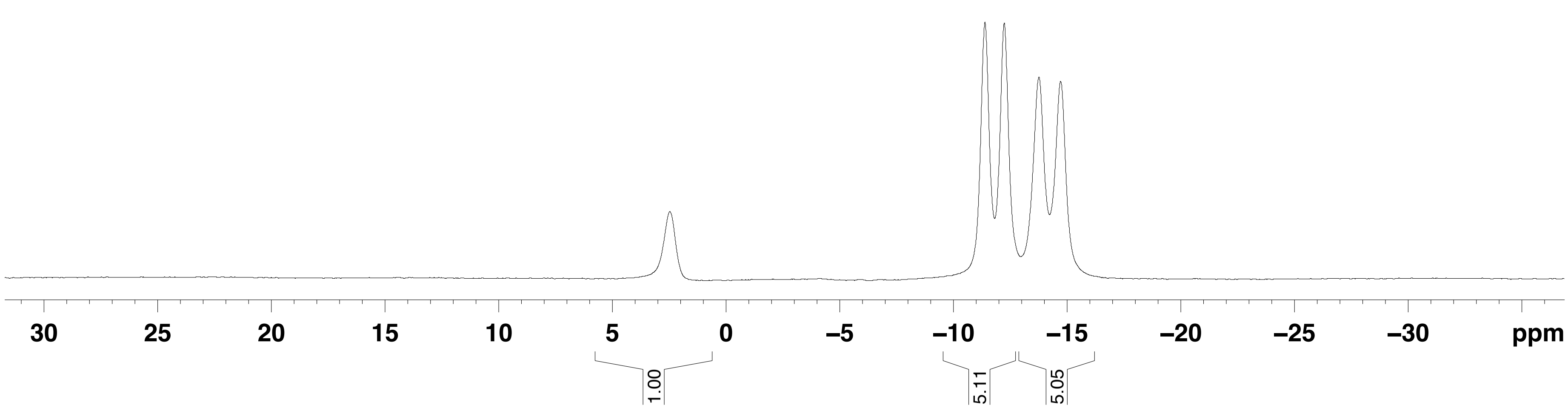

Current Data Parameters	
NAME	12-Me-acid
EXPNO	2
PROCNO	1
F2 - Acquisition Parameters	
Date_	20180824
Time	6.23
INSTRUM	spect
PROBHD	5 mm PABBO BB-
PULPROG	zg30
TD	64098
SOLVENT	Acetone
NS	32
DS	0
SWH	32051.281 Hz
FIDRES	0.500036 Hz
AQ	0.9999288 sec
RG	203
DW	15.600 usec
DE	6.50 usec
TE	295.9 K
D1	1.00000000 sec
TD0	1
======== CHANNEL f1 ========	
SFO1	160.4615792 MHz
NUC1	11B
P1	13.10 usec
PLW1	95.00000000 W
F2 - Processing parameters	
SI	32768
SF	160.4615790 MHz
WDW	EM
SSB	0
LB	10.00 Hz
GB	0
PC	1.40

1–COOH–12–Me–CB11 product 30 mg in 0.6 ml acetone–d6
11B{1H} NMR, 160 MHz, 23 C

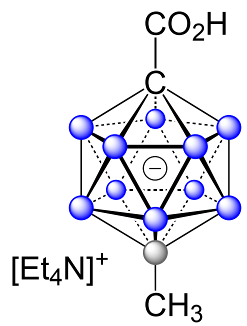

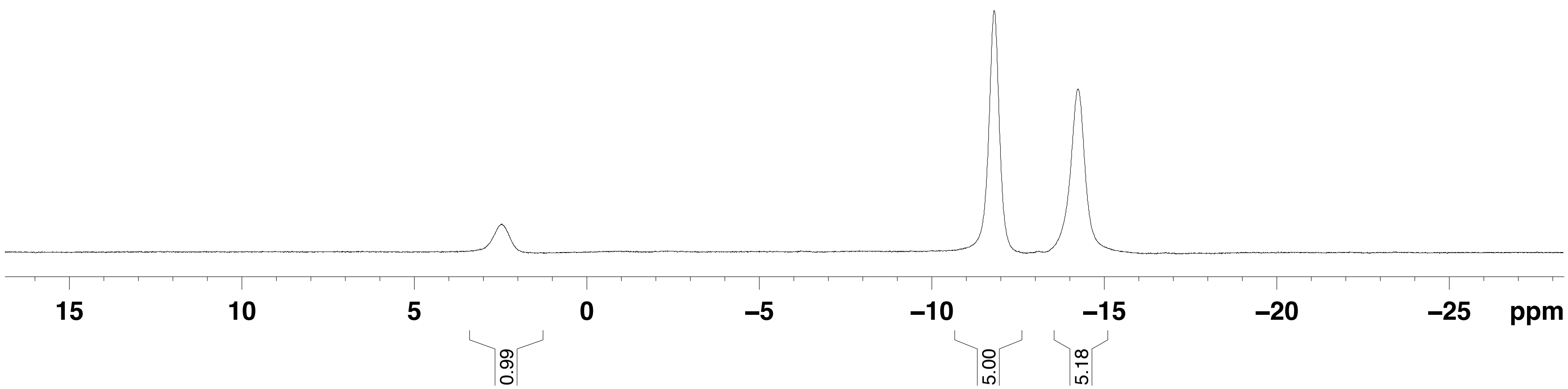


```
Current Data Parameters
NAME          12-Me-acid
EXPNO                  3
PROCNO                 1

F2 - Acquisition Parameters
Date_           20180824
Time                6.25
INSTRUM            spect
PROBHD   5 mm PABBO BB-
PULPROG           zgpg30
TD                 65536
SOLVENT          Acetone
NS                    32
DS                     0
SWH            32051.281 Hz
FIDRES          0.489064 Hz
AQ             1.0223616 sec
RG                   203
DW                15.600 usec
DE                  6.50 usec
TE                 296.3 K
D1            1.00000000 sec
D11           0.03000000 sec
TD0                    1

======== CHANNEL f1 ========
SFO1         160.4615790 MHz
NUC1                 11B
P1                 13.10 usec
PLW1         95.00000000 W

======== CHANNEL f2 ========
SFO2         500.1325007 MHz
NUC2                  1H
CPDPRG[2         waltz16
PCPD2              80.00 usec
PLW2         19.00000000 W
PLW12         0.40639001 W
PLW13         0.26008999 W

F2 - Processing parameters
SI                 32768
SF           160.4615790 MHz
WDW                   EM
SSB      0
LB                  1.00 Hz
GB       0
PC                  1.40
```

1–COOH–12–Me–CB11 product 30 mg in 0.6 ml acetone–d6 *
13C{1H} NMR, 125 MHz, 23 C

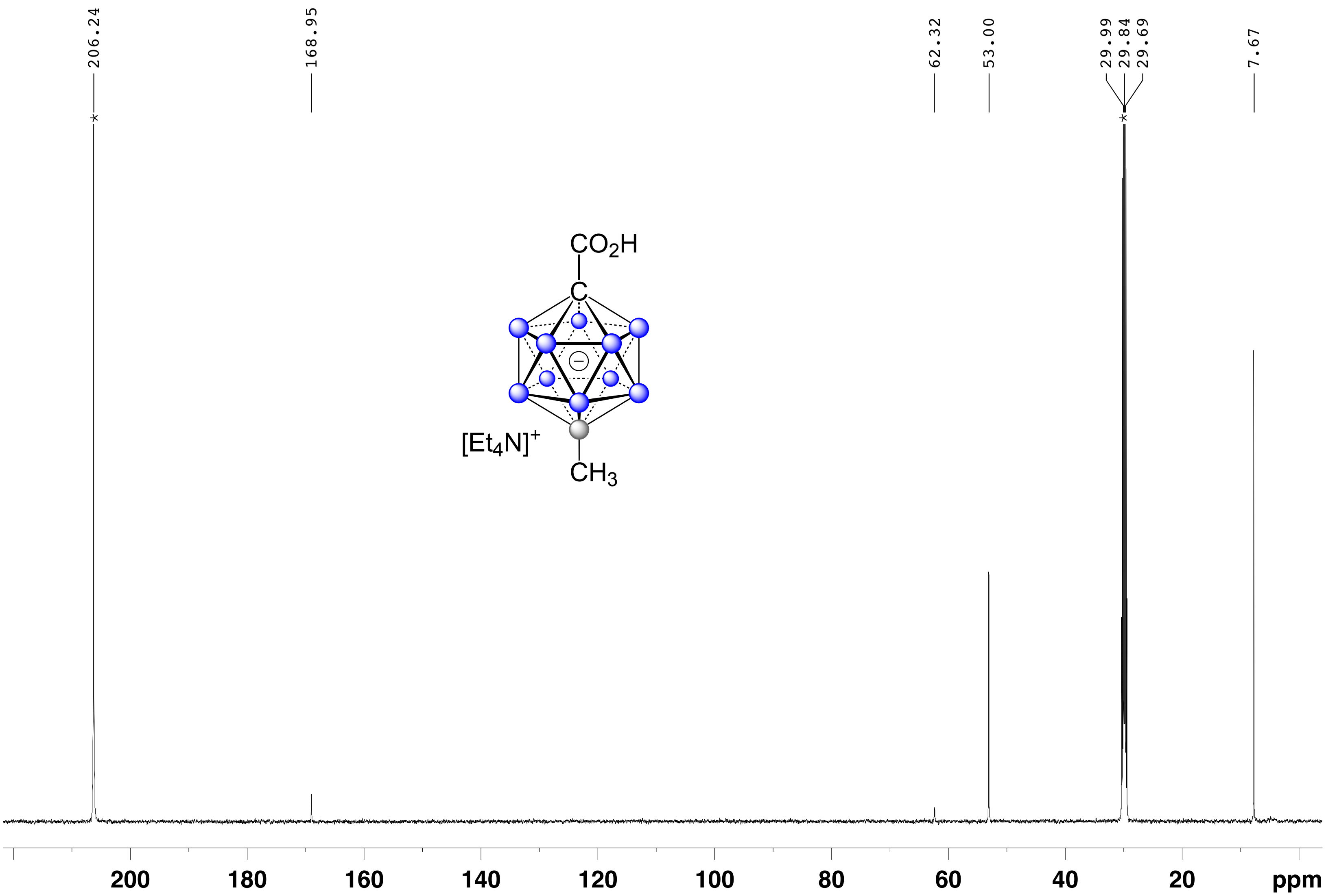


```
Current Data Parameters
NAME          12-Me-acid
EXPNO                  4
PROCNO                 1

F2 - Acquisition Parameters
Date_           20180824
Time                7.08
INSTRUM            spect
PROBHD   5 mm PABBO BB-
PULPROG          zgpg30
TD                 65536
SOLVENT          Acetone
NS                  1024
DS                     4
SWH            37878.789 Hz
FIDRES          0.577984 Hz
AQ             0.8650752 sec
RG                   203
DW                13.200 usec
DE                  6.50 usec
TE                 296.1 K
D1            1.50000000 sec
D11           0.03000000 sec
TD0                    1

======== CHANNEL f1 ========
SFO1         125.7716224 MHz
NUC1                 13C
P1                 10.50 usec
PLW1         95.00000000 W

======== CHANNEL f2 ========
SFO2         500.1320005 MHz
NUC2                  1H
CPDPRG[2         waltz16
PCPD2              80.00 usec
PLW2         19.00000000 W
PLW12         0.40639001 W
PLW13         0.26008999 W

F2 - Processing parameters
SI                 32768
SF           125.7576811 MHz
WDW                   EM
SSB      0
LB                  3.00 Hz
GB       0
PC                  1.40
```

1–COOH–12–Ph–CB11 product 30 mg in 0.6 ml acetone–d6 *
1H{11B}NMR, 400 MHz, 23 C

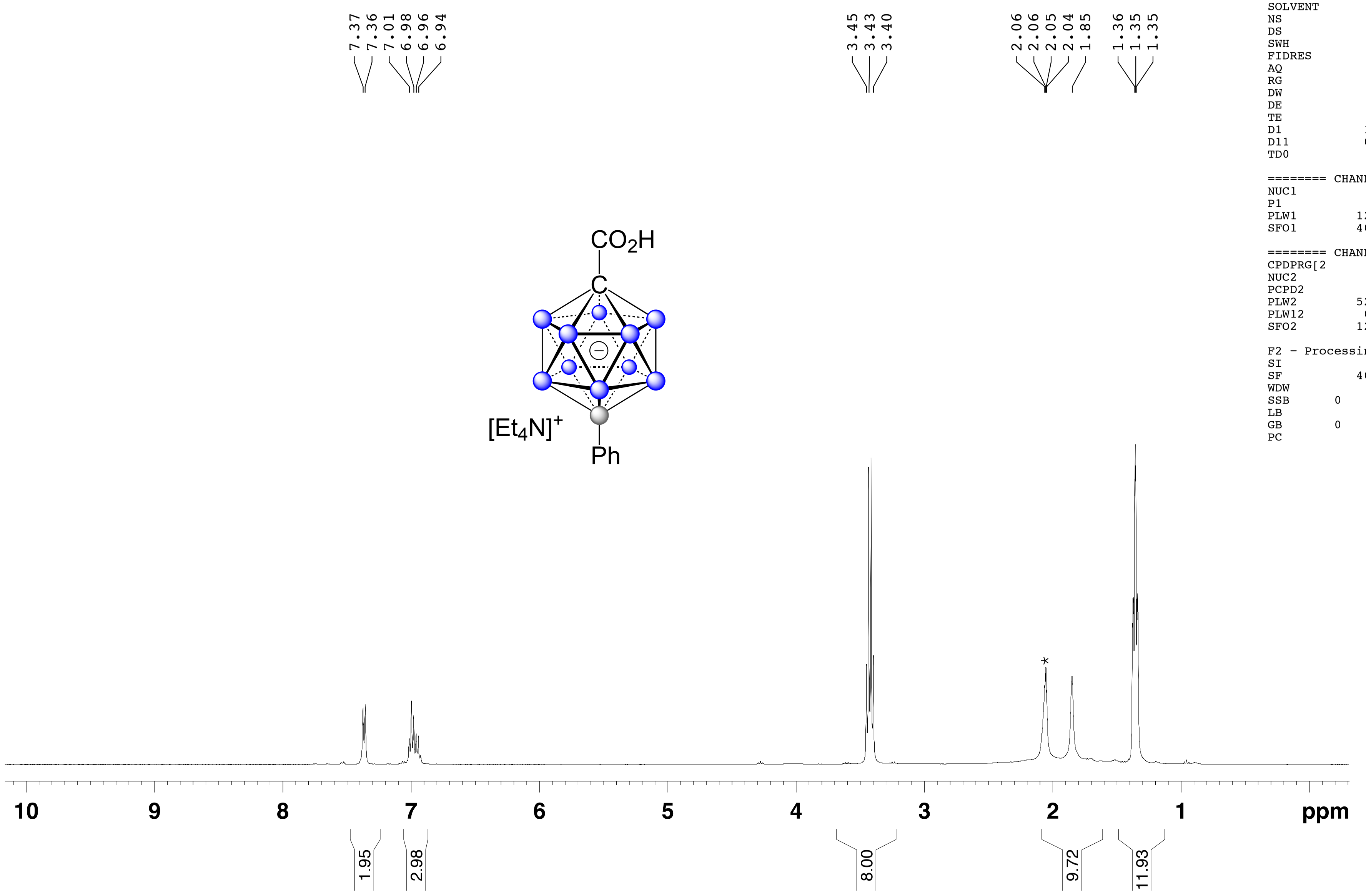


```
Current Data Parameters
NAME           12-Ph-acid
EXPNO                   1
PROCNO                  1

F2 - Acquisition Parameters
Date_            20180801
Time                14.05
INSTRUM             spect
PROBHD   5 mm PABBO BB/
PULPROG            zgig30
TD                  16384
SOLVENT           Acetone
NS                     16
DS                      4
SWH              8012.820 Hz
FIDRES           0.489064 Hz
AQ              1.0223616 sec
RG                  107.6
DW                 62.400 usec
DE                   6.50 usec
TE                  295.3 K
D1             1.00000000 sec
D11            0.03000000 sec
TD0                     1

======== CHANNEL f1 ========
NUC1                   1H
P1                  15.00 usec
PLW1          12.50000000 W
SFO1          400.1320007 MHz

======== CHANNEL f2 ========
CPDPRG[2            garp4
NUC2                  11B
PCPD2               90.00 usec
PLW2          52.96599960 W
PLW12          0.64477998 W
SFO2          128.3776050 MHz

F2 - Processing parameters
SI                  32768
SF            400.1300073 MHz
WDW                    EM
SSB      0
LB                   1.00 Hz
GB       0
PC                   1.40
```

1–COOH–12–Ph–CB11 product 30 mg in 0.6 ml acetone–d6
11B NMR, 128 MHz, 23 C

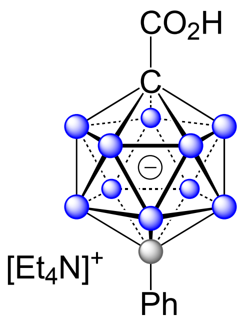

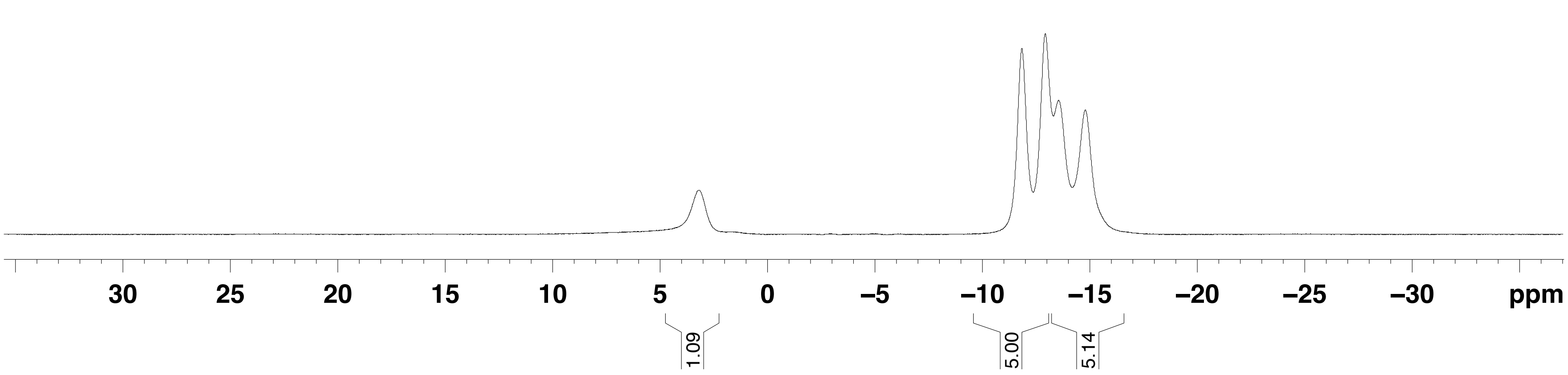

Current Data Parameters		
NAME	12-Ph-acid	
EXPNO	2	
PROCNO	1	
F2 - Acquisition Parameters		
Date_	20180801	
Time	14.11	
INSTRUM	spect	
PROBHD	5 mm PABBO BB/	
PULPROG	zg	
TD	65536	
SOLVENT	Acetone	
NS	128	
DS	4	
SWH	25510.203	Hz
FIDRES	0.389255	Hz
AQ	1.2845056	sec
RG	193.34	
DW	19.600	usec
DE	6.50	usec
TE	294.9	K
D1	1.00000000	sec
TD0	1	
======== CHANNEL f1 ========		
NUC1	11B	
P1	9.93	usec
PLW1	52.96599960	W
SFO1	128.3776052	MHz
F2 - Processing parameters		
SI	32768	
SF	128.3776050	MHz
WDW	EM	
SSB	0	
LB	1.00	Hz
GB	0	
PC	1.40	

1–COOH–12–Ph–CB11 product 30 mg in 0.6 ml acetone–d6
11B{1H} NMR, 128 MHz, 23 C

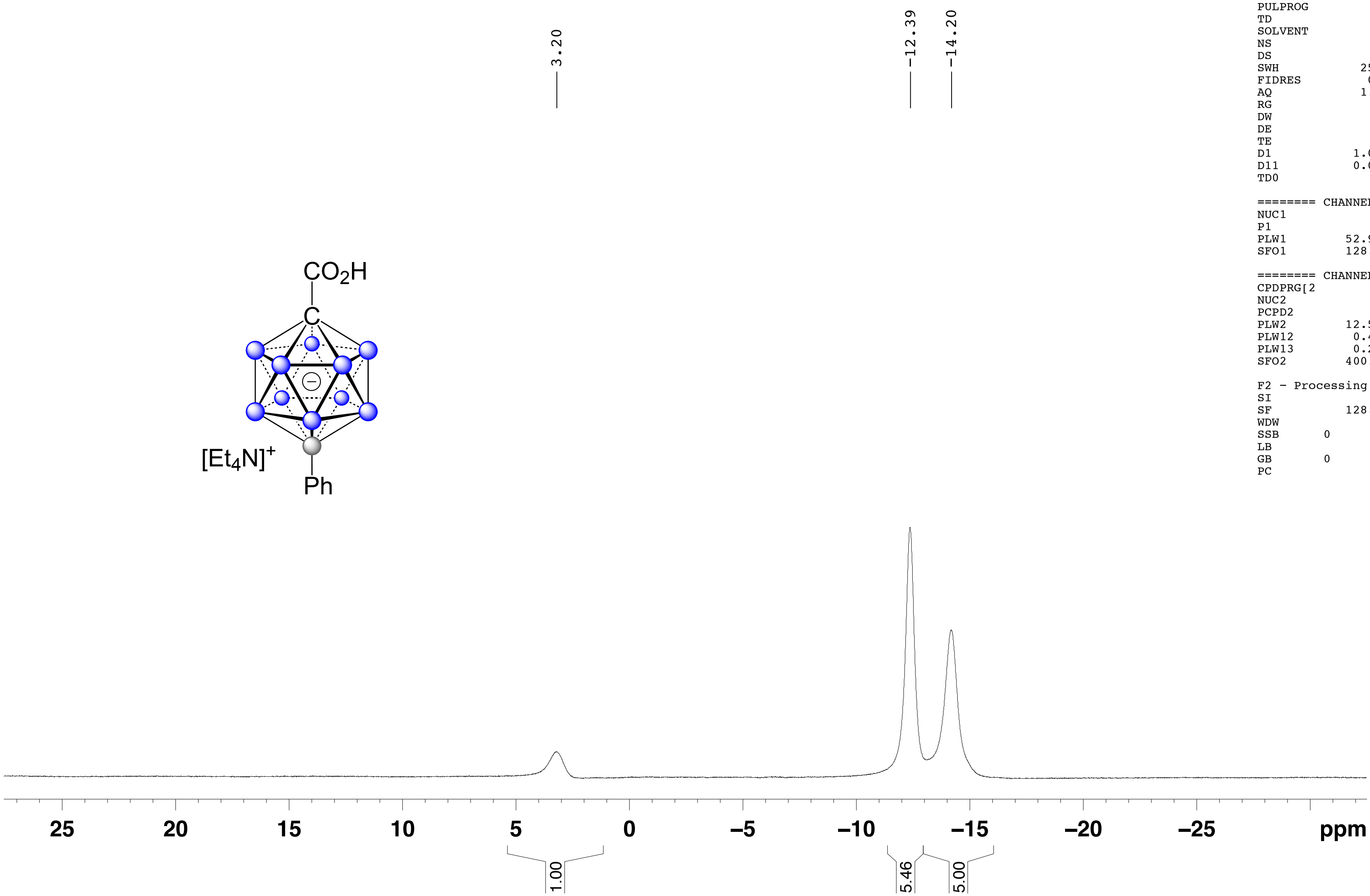


```
Current Data Parameters
NAME          12-Ph-acid
EXPNO                  3
PROCNO                 1

F2 - Acquisition Parameters
Date_           20180801
Time               14.17
INSTRUM            spect
PROBHD   5 mm PABBO BB/
PULPROG           zgpg30
TD                 65536
SOLVENT          Acetone
NS                   128
DS                     4
SWH            25510.203 Hz
FIDRES          0.389255 Hz
AQ             1.2845056 sec
RG                193.34
DW                19.600 usec
DE                  6.50 usec
TE                 295.5 K
D1            1.00000000 sec
D11           0.03000000 sec
TD0                    1

======== CHANNEL f1 ========
NUC1                 11B
P1                  9.93 usec
PLW1         52.96599960 W
SFO1         128.3776050 MHz

======== CHANNEL f2 ========
CPDPRG[2         waltz16
NUC2                  1H
PCPD2              80.00 usec
PLW2         12.50000000 W
PLW12         0.43945000 W
PLW13         0.28125000 W
SFO2         400.1320007 MHz

F2 - Processing parameters
SI                 32768
SF           128.3776050 MHz
WDW                   EM
SSB      0
LB                  1.00 Hz
GB       0
PC                  1.40
```

1–COOH–12–Ph–CB11 product 30 mg in 0.6 ml acetone–d6*
13C{1H} NMR, 101 MHz, 23 C

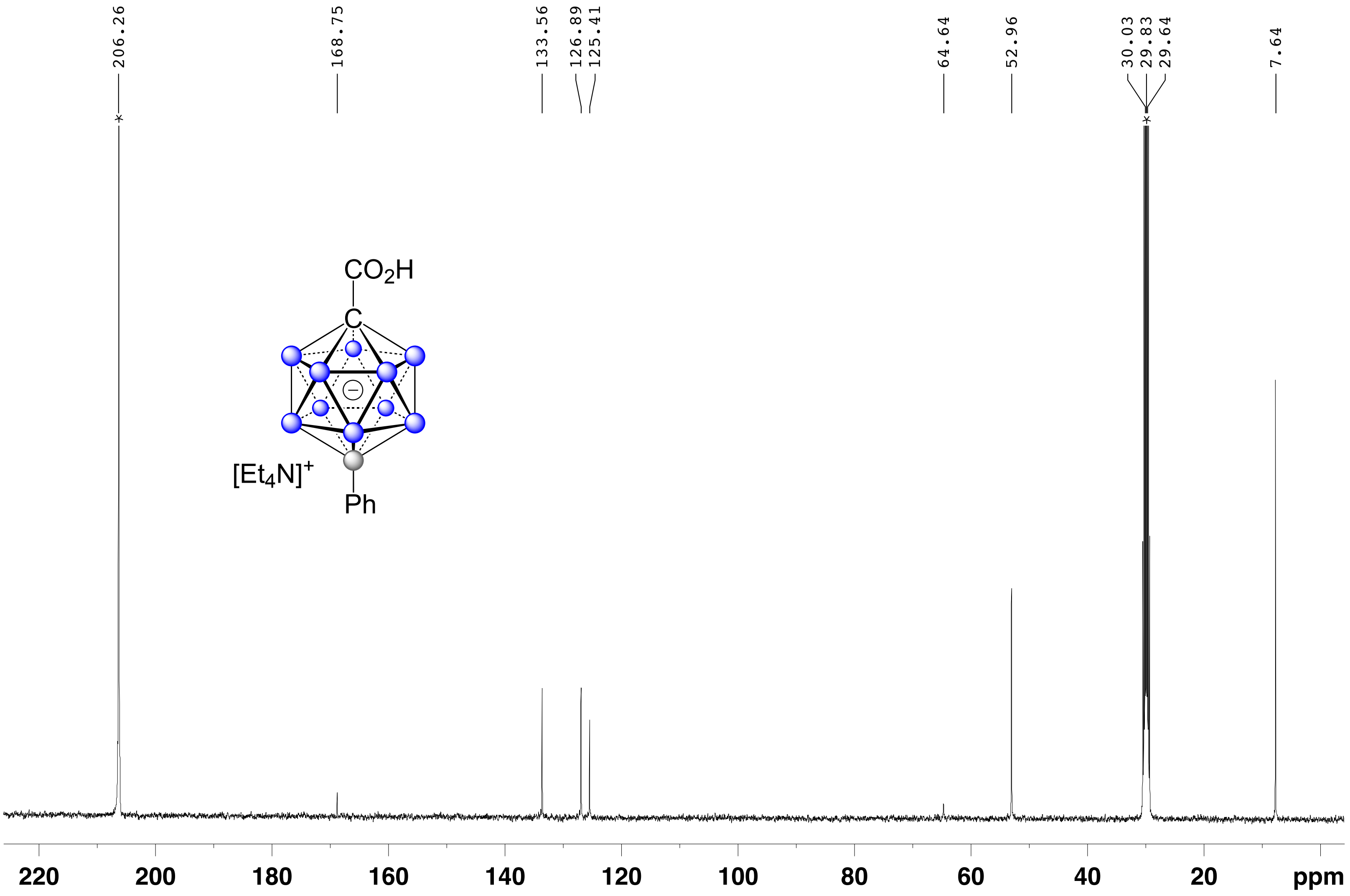


```
Current Data Parameters
NAME       12-Ph-acid-13C
EXPNO                   1
PROCNO                  1

F2 - Acquisition Parameters
Date_            20180802
Time                13.43
INSTRUM             spect
PROBHD   5 mm PABBO BB/
PULPROG           zgpg30
TD                 65536
SOLVENT          Acetone
NS                  1024
DS                     4
SWH            29761.904 Hz
FIDRES          0.454131 Hz
AQ             1.1010048 sec
RG                193.34
DW                16.800 usec
DE                  6.50 usec
TE                 295.5 K
D1            1.50000000 sec
D11           0.03000000 sec
TD0                    1

======== CHANNEL f1 ========
NUC1                 13C
P1                 10.00 usec
PLW1         53.00000000 W
SFO1         100.6228293 MHz

======== CHANNEL f2 ========
CPDPRG[2         waltz16
NUC2                  1H
PCPD2              80.00 usec
PLW2         12.50000000 W
PLW12         0.43945000 W
PLW13         0.28125000 W
SFO2         400.1316005 MHz

F2 - Processing parameters
SI                 32768
SF           100.6126835 MHz
WDW                   EM
SSB        0
LB                  3.00 Hz
GB         0
PC                  1.40
```

Penta-4–F–styrene product 80 mg in 0.6 ml acetone–d6*
1H{11B} NMR, 400 MHz, 23 C

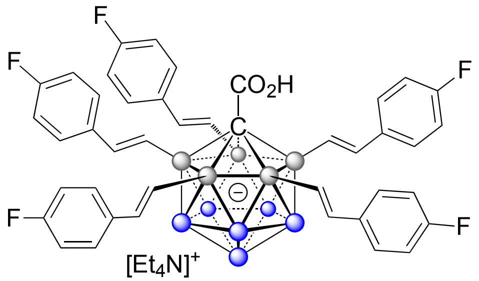

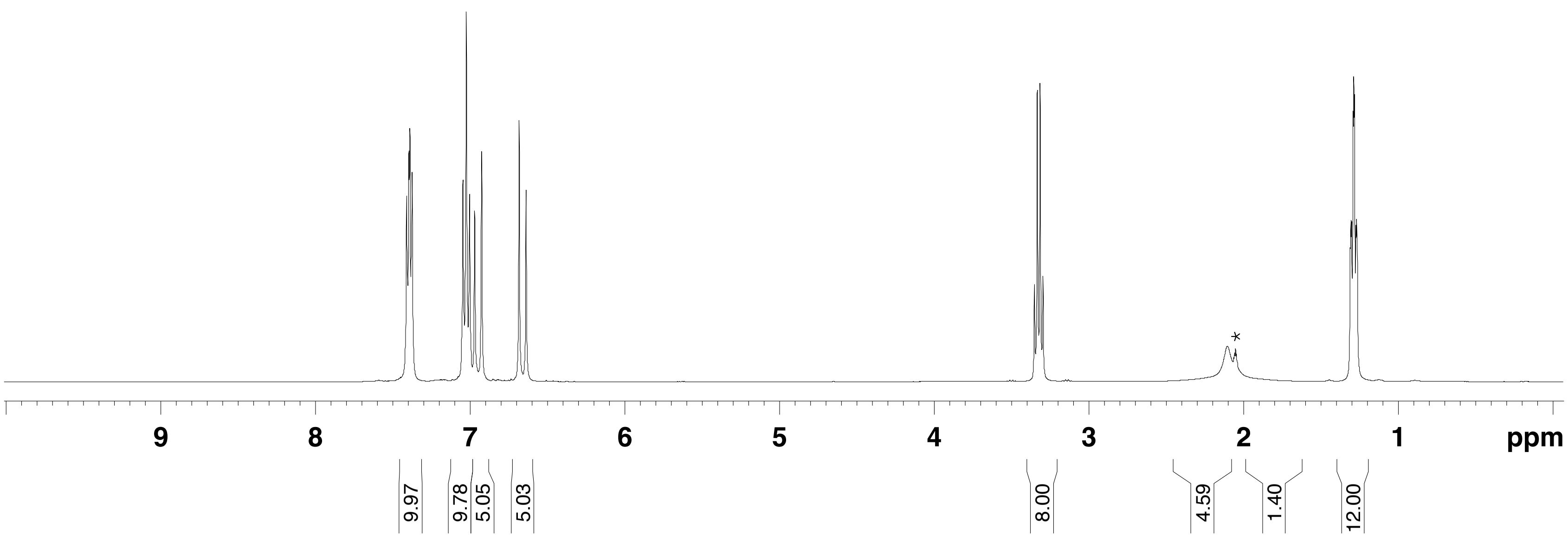


```
Current Data Parameters
NAME     penta-4-F-styrene
EXPNO                    1
PROCNO                   1

F2 - Acquisition Parameters
Date_             20180529
Time                 18.17
INSTRUM              spect
PROBHD   5 mm PABBO BB/
PULPROG             zgig30
TD                   16384
SOLVENT            Acetone
NS                      16
DS                       4
SWH               8012.820 Hz
FIDRES            0.489064 Hz
AQ               1.0223616 sec
RG                   23.04
DW                  62.400 usec
DE                    6.50 usec
TE                   294.3 K
D1              1.00000000 sec
D11             0.03000000 sec
TD0                      1

======== CHANNEL f1 ========
NUC1                    1H
P1                   15.00 usec
PLW1           12.50000000 W
SFO1           400.1320007 MHz

======== CHANNEL f2 ========
CPDPRG[2             garp4
NUC2                   11B
PCPD2                90.00 usec
PLW2           52.96599960 W
PLW12           0.64477998 W
SFO2           128.3776050 MHz

F2 - Processing parameters
SI                   32768
SF             400.1300073 MHz
WDW                     EM
SSB      0
LB                    1.00 Hz
GB       0
PC                    1.40
```

Penta-4–F–styrene product 80 mg in 0.6 ml acetone–d6
11B NMR, 128 MHz, 23 C

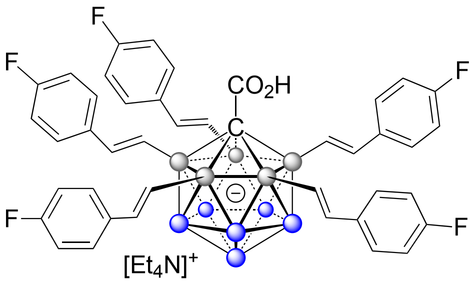

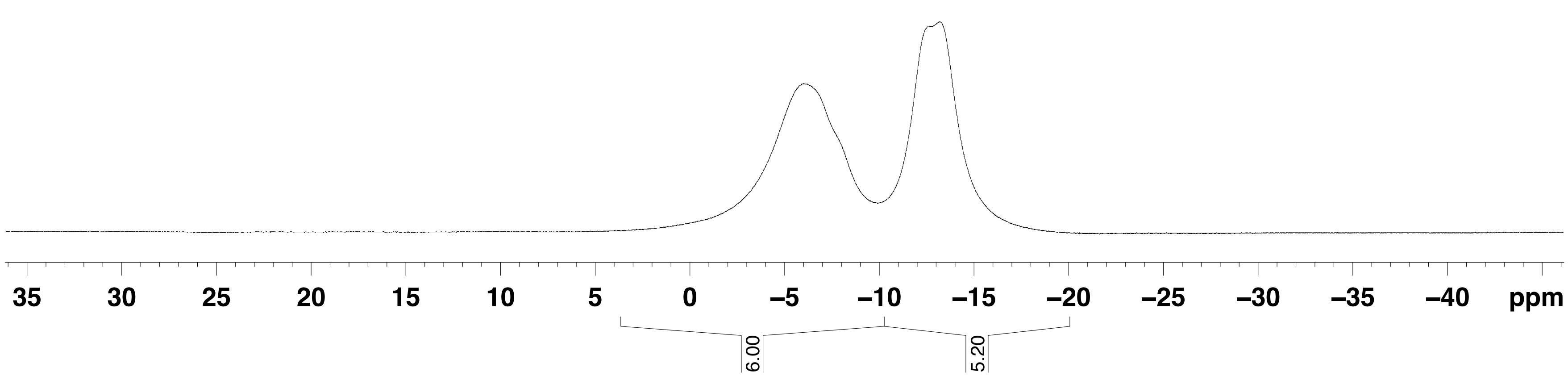


```
Current Data Parameters
NAME      penta-4-F-styrene
EXPNO                     2
PROCNO                    1

F2 - Acquisition Parameters
Date_              20180529
Time                  18.22
INSTRUM               spect
PROBHD   5 mm PABBO BB/
PULPROG                  zg
TD                    65536
SOLVENT             Acetone
NS                      128
DS                        4
SWH               25510.203 Hz
FIDRES             0.389255 Hz
AQ                1.2845056 sec
RG                   193.34
DW                   19.600 usec
DE                     6.50 usec
TE                    294.2 K
D1               1.00000000 sec
TD0                       1

======== CHANNEL f1 ========
NUC1                    11B
P1                     9.93 usec
PLW1            52.96599960 W
SFO1            128.3776052 MHz

F2 - Processing parameters
SI                    32768
SF              128.3776050 MHz
WDW                      EM
SSB      0
LB                     1.00 Hz
GB       0
PC                     1.40
```

Penta–4-F–styrine product 80 mg in 0.6 ml acetone–d6
11B{1H} NMR, 128 MHz, 23 C

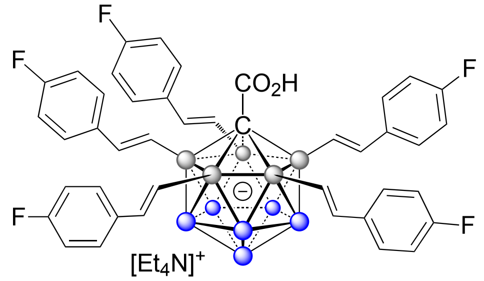

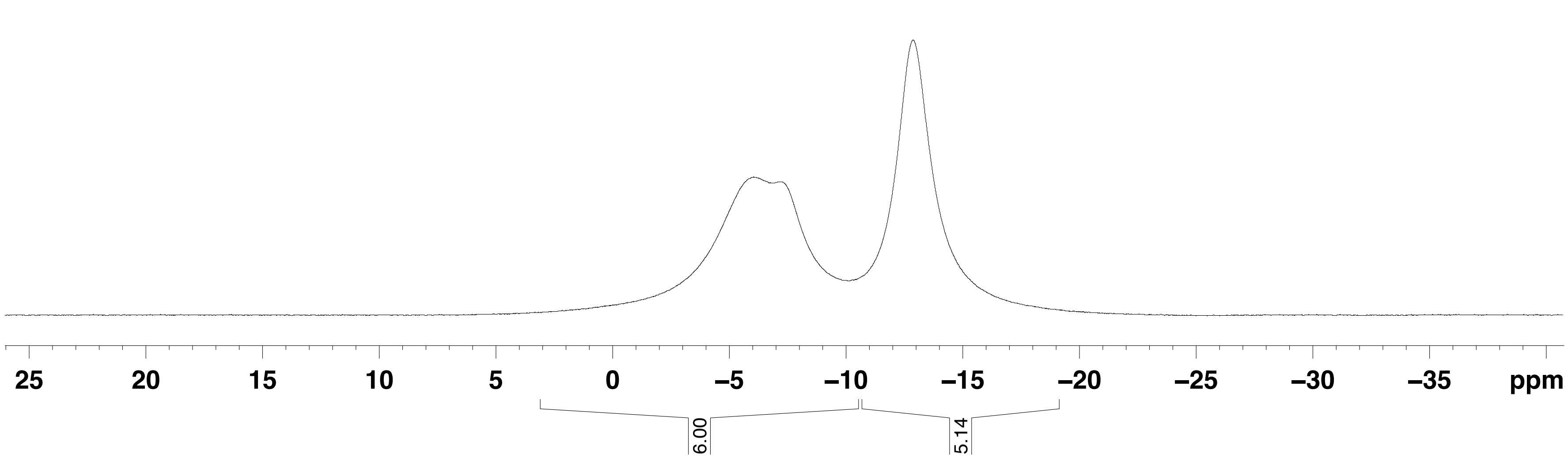


```
Current Data Parameters
NAME     penta-4-F-styrene
EXPNO                    3
PROCNO                   1

F2 - Acquisition Parameters
Date_          20180529
Time              18.28
INSTRUM           spect
PROBHD   5 mm PABBO BB/
PULPROG          zgpg30
TD                65536
SOLVENT         Acetone
NS                  128
DS                    4
SWH           25510.203 Hz
FIDRES         0.389255 Hz
AQ            1.2845056 sec
RG               193.34
DW               19.600 usec
DE                 6.50 usec
TE                293.9 K
D1           1.00000000 sec
D11          0.03000000 sec
TD0                   1

======== CHANNEL f1 ========
NUC1                 11B
P1                  9.93 usec
PLW1         52.96599960 W
SFO1        128.3776050 MHz

======== CHANNEL f2 ========
CPDPRG[2         waltz16
NUC2                  1H
PCPD2              80.00 usec
PLW2         12.50000000 W
PLW12         0.43945000 W
PLW13         0.28125000 W
SFO2        400.1320007 MHz

F2 - Processing parameters
SI                 32768
SF           128.3776050 MHz
WDW                   EM
SSB      0
LB                  3.00 Hz
GB       0
PC                  1.40
```

Penta-4–F–styrene product 80 mg in 0.6 ml acetone–d6*
13C{1H} NMR, 101 MHz, 23 C

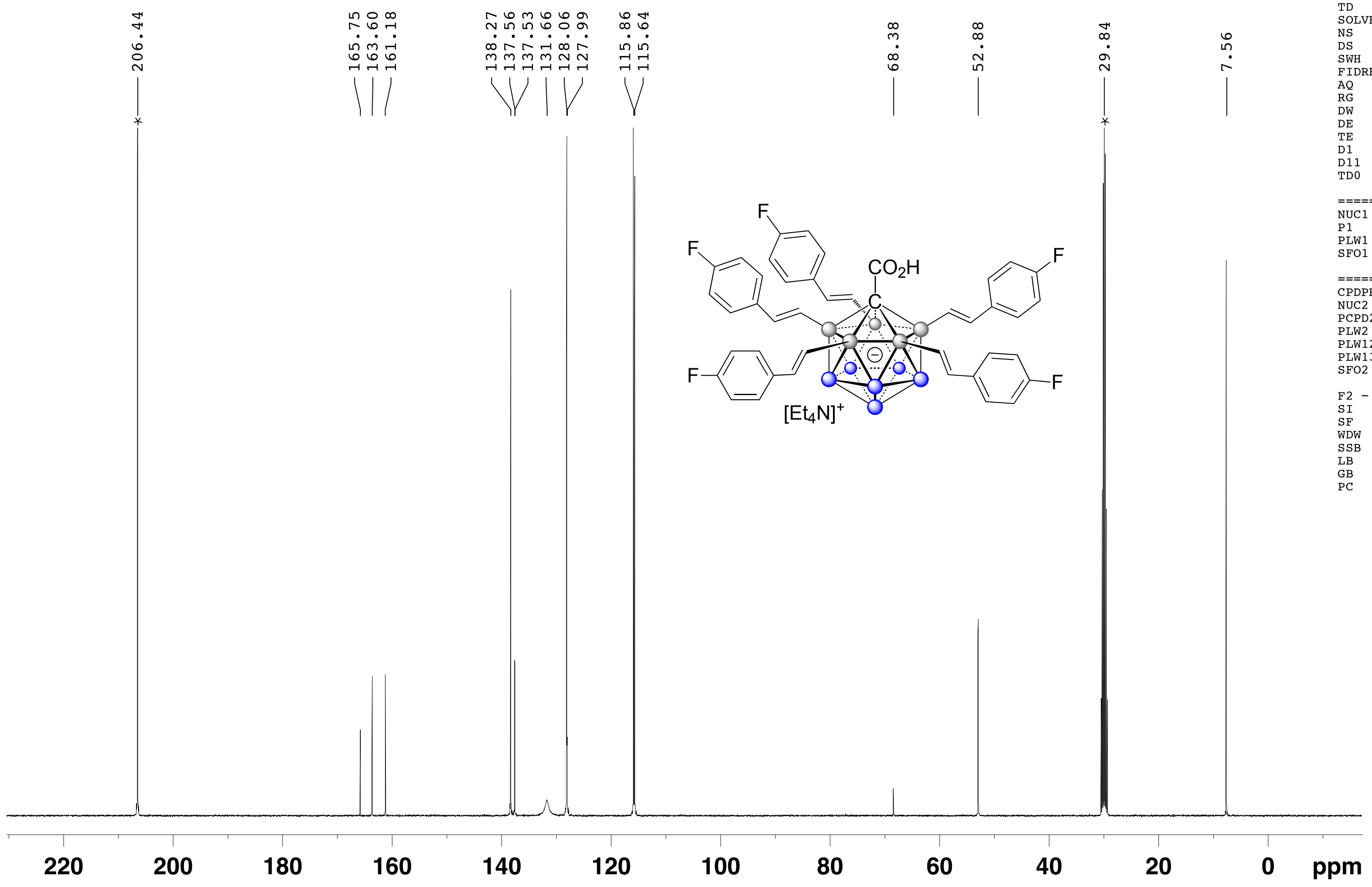


```
Current Data Parameters
NAME     penta-4-F-styrene
EXPNO                   4
PROCNO                  1

F2 - Acquisition Parameters
Date_          20180529
Time              20.01
INSTRUM           spect
PROBHD   5 mm PABBO BB/
PULPROG          zgpg30
TD                65536
SOLVENT         Acetone
NS                 2048
DS                    4
SWH          29761.904 Hz
FIDRES        0.454131 Hz
AQ           1.1010048 sec
RG               193.34
DW               16.800 usec
DE                 6.50 usec
TE                294.2 K
D1           1.50000000 sec
D11          0.03000000 sec
TD0                   1

======== CHANNEL f1 ========
NUC1                13C
P1                10.00 usec
PLW1        53.00000000 W
SFO1        100.6228293 MHz

======== CHANNEL f2 ========
CPDPRG[2        waltz16
NUC2                 1H
PCPD2             80.00 usec
PLW2        12.50000000 W
PLW12        0.43945000 W
PLW13        0.28125000 W
SFO2        400.1316005 MHz

F2 - Processing parameters
SI                32768
SF          100.6126935 MHz
WDW                  EM
SSB      0
LB                 1.00 Hz
GB       0
PC                 1.40
```

Penta–styrene product 50 mg in 0.6 ml acetone–d6*
1H{11B} NMR, 500 MHz, 23 C

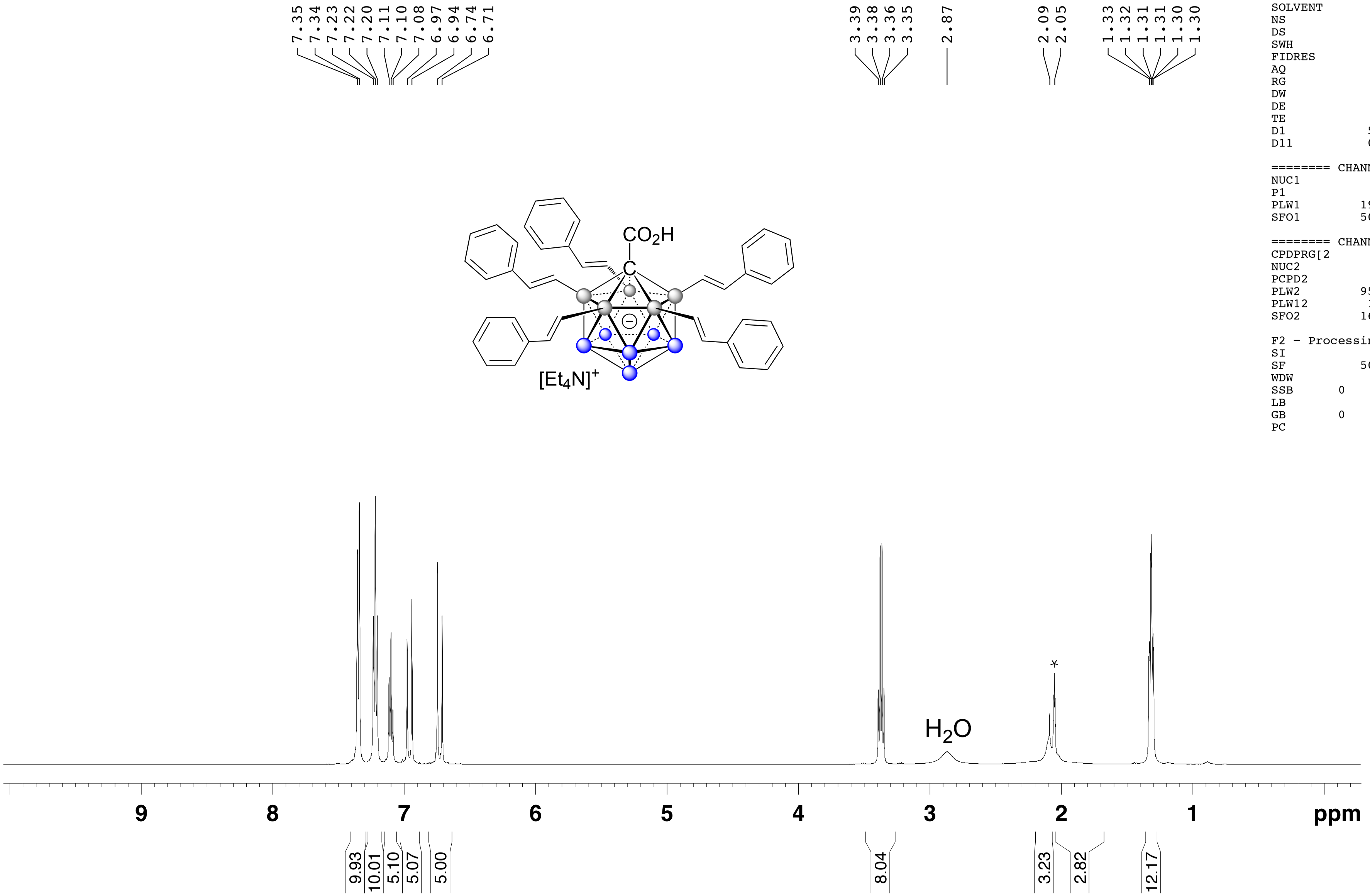


```
Current Data Parameters
NAME       penta-styrene
EXPNO                  1
PROCNO                 1

F2 - Acquisition Parameters
Date_           20180509
Time                2.08
INSTRUM            spect
PROBHD   5 mm PABBO BB-
PULPROG           zgig30
TD                 65536
SOLVENT          Acetone
NS                    16
DS                     0
SWH            12500.000 Hz
FIDRES          0.190735 Hz
AQ             2.6214399 sec
RG                  71.8
DW                40.000 usec
DE                  6.50 usec
TE                 296.3 K
D1            5.00000000 sec
D11           0.03000000 sec

======== CHANNEL f1 ========
NUC1                  1H
P1                 11.70 usec
PLW1         19.00000000 W
SFO1         500.1335009 MHz

======== CHANNEL f2 ========
CPDPRG[2            garp
NUC2                 11B
PCPD2             100.00 usec
PLW2         95.00000000 W
PLW12         1.63030005 W
SFO2         160.4615690 MHz

F2 - Processing parameters
SI                 65536
SF           500.1300101 MHz
WDW                   EM
SSB      0
LB                  1.00 Hz
GB       0
PC                  1.00
```

Penta–styrene product 50 mg in 0.6 ml acetone–d6
11B NMR, 160 MHz, 23 C

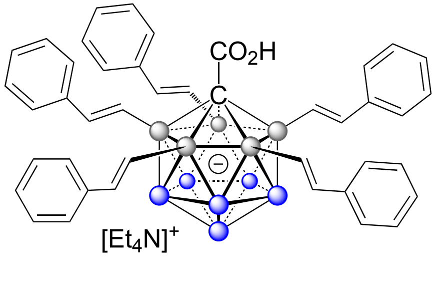

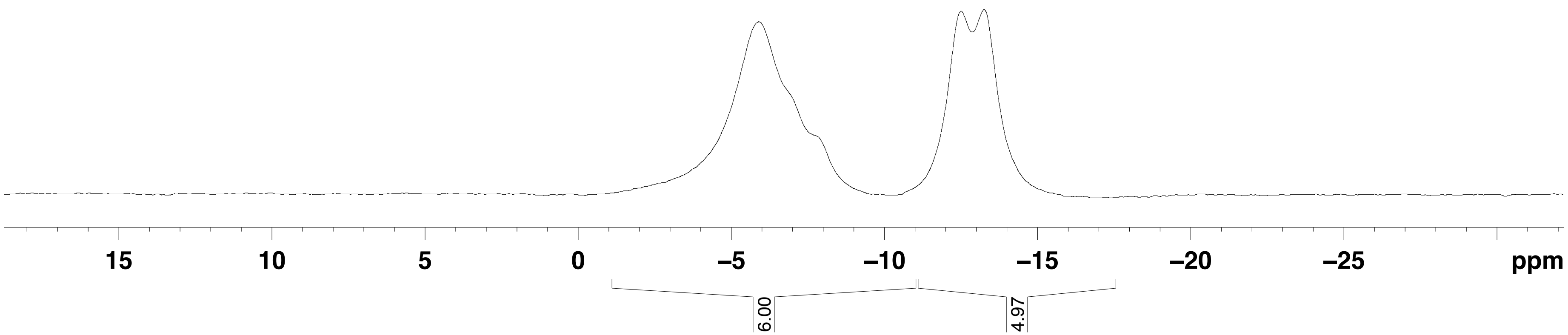


```
Current Data Parameters
NAME      penta-styrene
EXPNO                 2
PROCNO                1

F2 - Acquisition Parameters
Date_          20180509
Time               2.10
INSTRUM           spect
PROBHD   5 mm PABBO BB-
PULPROG            zg30
TD                64098
SOLVENT         Acetone
NS                   64
DS                    0
SWH           32051.281 Hz
FIDRES         0.500036 Hz
AQ            0.9999288 sec
RG                  203
DW               15.600 usec
DE                 6.50 usec
TE                295.8 K
D1           1.00000000 sec

======== CHANNEL f1 ========
NUC1                11B
P1                13.10 usec
PLW1        95.00000000 W
SFO1        160.4615792 MHz

F2 - Processing parameters
SI                32768
SF          160.4615790 MHz
WDW                  EM
SSB      0
LB                20.00 Hz
GB       0
PC                 1.40
```

Penta–styrene product 50 mg in 0.6 ml acetone–d6
11B{1H} NMR, 160 MHz, 23 C

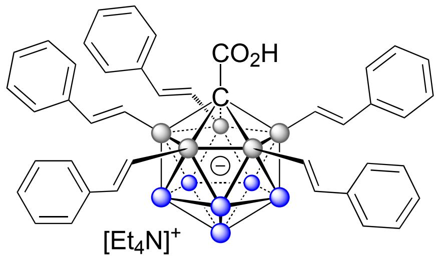

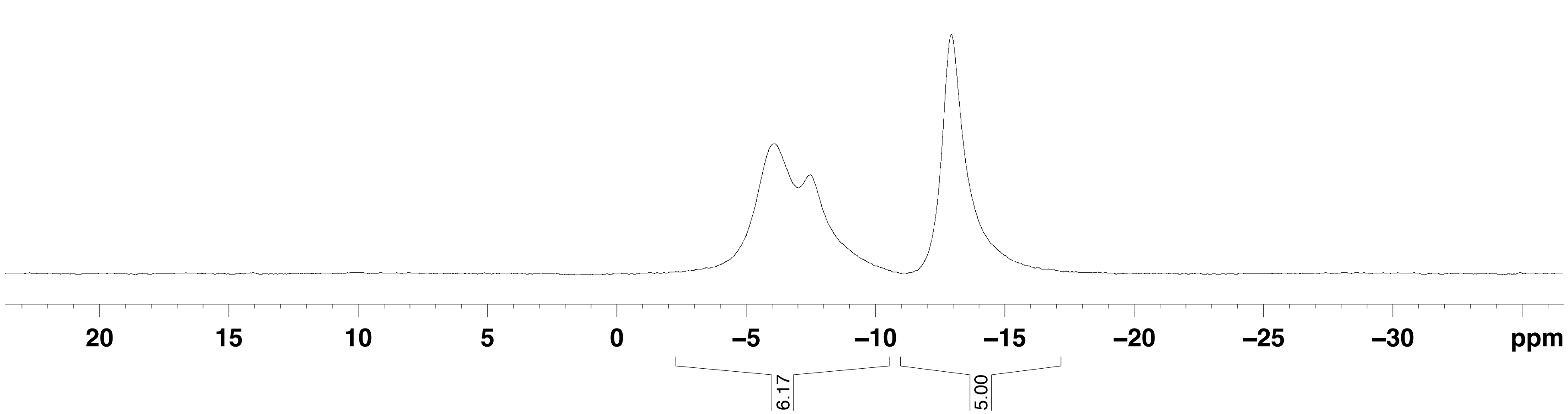


```
Current Data Parameters
NAME      penta-styrene
EXPNO                 3
PROCNO                1

F2 - Acquisition Parameters
Date_          20180509
Time               2.14
INSTRUM           spect
PROBHD   5 mm PABBO BB-
PULPROG          zgpg30
TD                65536
SOLVENT         Acetone
NS                   64
DS                    0
SWH           32051.281 Hz
FIDRES         0.489064 Hz
AQ            1.0223616 sec
RG                  203
DW               15.600 usec
DE                 6.50 usec
TE                295.8 K
D1           1.00000000 sec
D11          0.03000000 sec

======== CHANNEL f1 ========
NUC1                11B
P1                13.10 usec
PLW1        95.00000000 W
SFO1        160.4615790 MHz

======== CHANNEL f2 ========
CPDPRG[2        waltz16
NUC2                 1H
PCPD2             80.00 usec
PLW2        19.00000000 W
PLW12        0.40639001 W
PLW13        0.26008999 W
SFO2        500.1325007 MHz

F2 - Processing parameters
SI                32768
SF          160.4615790 MHz
WDW                  EM
SSB      0
LB                15.00 Hz
GB       0
PC                 1.40
```

Penta–styrene product 50 mg in 0.6 ml acetone–d6
13C{1H} NMR, 126 MHz, 23 C

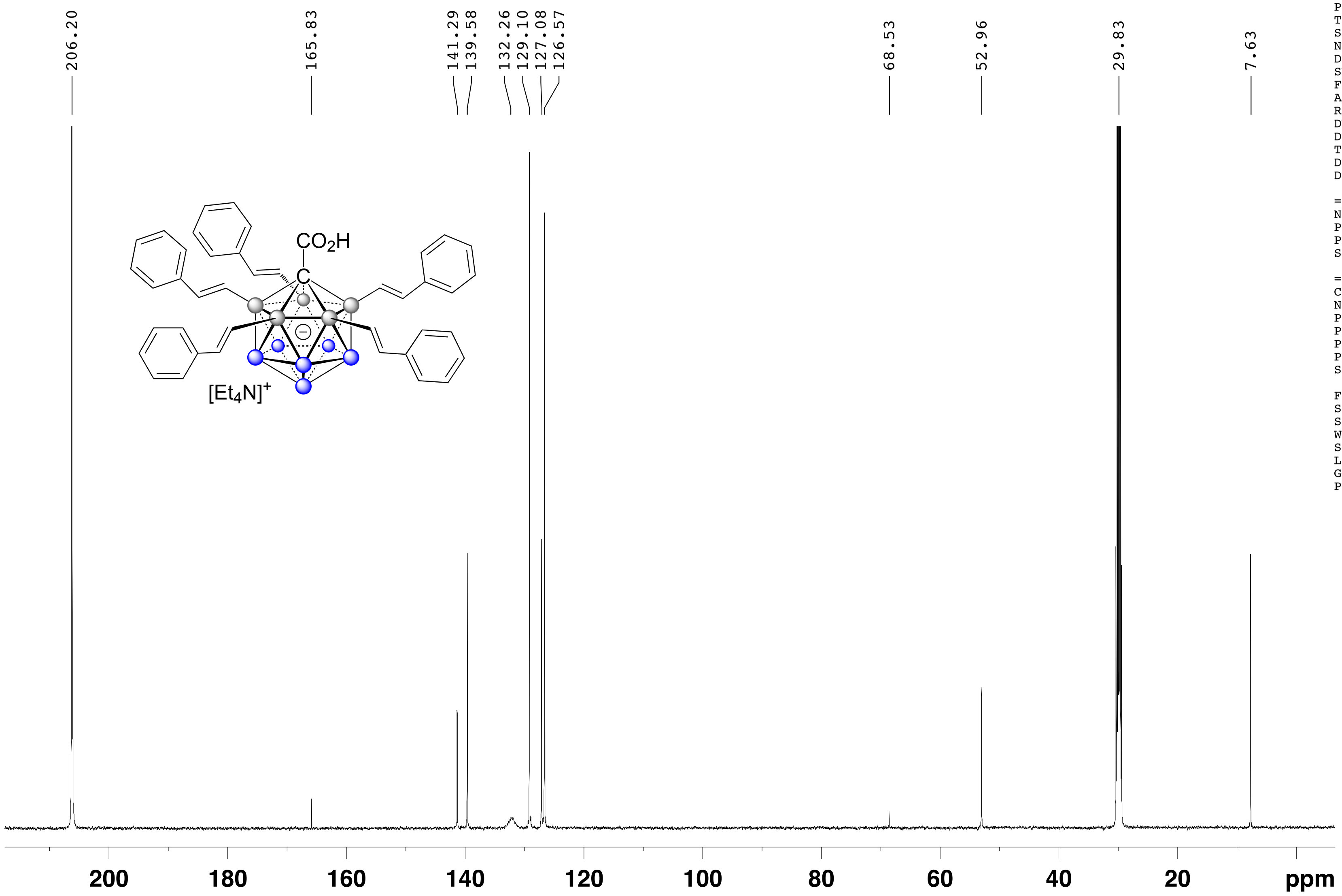


```
Current Data Parameters
NAME        penta-styrene
EXPNO                   4
PROCNO                  1

F2 - Acquisition Parameters
Date_            20180509
Time                 4.20
INSTRUM             spect
PROBHD   5 mm PABBO BB-
PULPROG           zgpg30
TD                 65536
SOLVENT          Acetone
NS                  3100
DS                     4
SWH           37878.789 Hz
FIDRES         0.577984 Hz
AQ            0.8650752 sec
RG                   203
DW                13.200 usec
DE                  6.50 usec
TE                 296.1 K
D1            1.50000000 sec
D11           0.03000000 sec

======== CHANNEL f1 ========
NUC1                  13C
P1                  10.50 usec
PLW1          95.00000000 W
SFO1          125.7716224 MHz

======== CHANNEL f2 ========
CPDPRG[2          waltz16
NUC2                   1H
PCPD2               80.00 usec
PLW2          19.00000000 W
PLW12          0.40639001 W
PLW13          0.26008999 W
SFO2          500.1320005 MHz

F2 - Processing parameters
SI                  32768
SF            125.7576810 MHz
WDW                    EM
SSB      0
LB                   3.00 Hz
GB       0
PC                   1.40
```

Penta–CF3–styrene–acid chloride product 30 mg in 0.6 ml acetonitrile–d3*
1H{11B} NMR, 500 MHz, 23 C

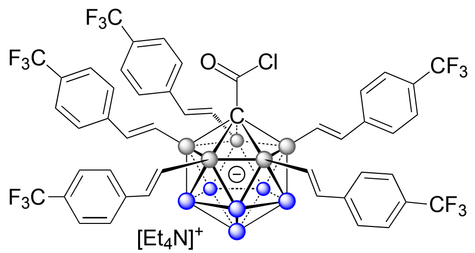

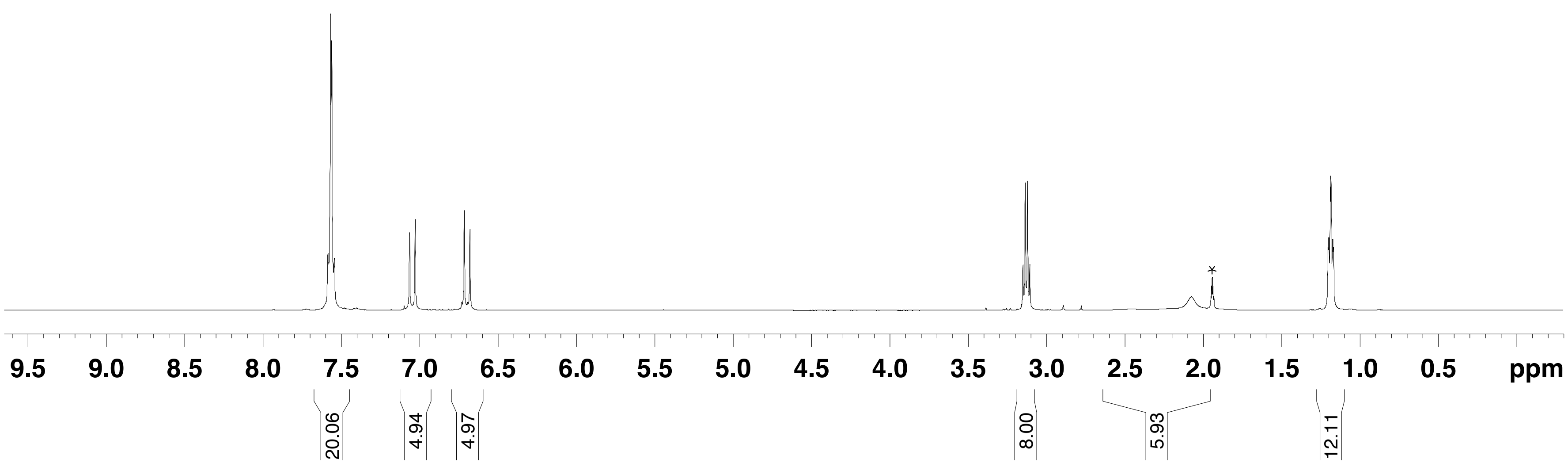


```
Current Data Parameters
NAME      Penta-CF3-styrine-acid chl
EXPNO                     1
PROCNO                    1

F2 - Acquisition Parameters
Date_              20181219
Time                  23.32
INSTRUM               spect
PROBHD   5 mm PABBO BB-
PULPROG              zgig30
TD                    65536
SOLVENT               CD3CN
NS                       16
DS                        0
SWH               12500.000 Hz
FIDRES             0.190735 Hz
AQ                2.6214399 sec
RG                      114
DW                   40.000 usec
DE                     6.50 usec
TE                    296.9 K
D1               5.00000000 sec
D11              0.03000000 sec

======== CHANNEL f1 ========
NUC1                     1H
P1                    11.70 usec
PLW1            19.00000000 W
SFO1            500.1335009 MHz

======== CHANNEL f2 ========
CPDPRG[2               garp
NUC2                    11B
PCPD2                100.00 usec
PLW2            95.00000000 W
PLW12            1.63030005 W
SFO2            160.4615690 MHz

F2 - Processing parameters
SI                    65536
SF              500.1300156 MHz
WDW                      EM
SSB      0
LB                     1.00 Hz
GB       0
PC                     1.00
```

Penta–CF3–styrene–acid chloride product 30 mg in 0.6 ml acetonitrile–d3
11B NMR, 160 MHz, 23 C

```
Current Data Parameters
NAME     Penta-CF3-styrine-acid chl
EXPNO                  2
PROCNO                 1

F2 - Acquisition Parameters
Date_           20181208
Time                2.18
INSTRUM            spect
PROBHD   5 mm PABBO BB-
PULPROG             zg30
TD                 64098
SOLVENT            CD3CN
NS                   128
DS                     0
SWH            32051.281 Hz
FIDRES          0.500036 Hz
AQ             0.9999288 sec
RG                   203
DW                15.600 usec
DE                  6.50 usec
TE                 296.9 K
D1            1.00000000 sec

======== CHANNEL f1 ========
NUC1                 11B
P1                 13.10 usec
PLW1         95.00000000 W
SFO1         160.4615792 MHz

F2 - Processing parameters
SI                 32768
SF           160.4615790 MHz
WDW                   EM
SSB      0
LB                 10.00 Hz
GB       0
PC                  1.40
```

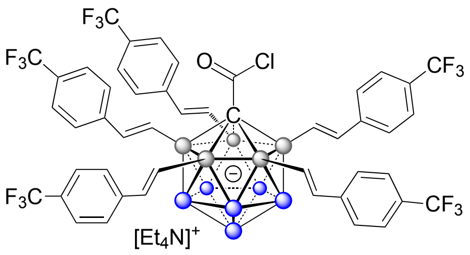

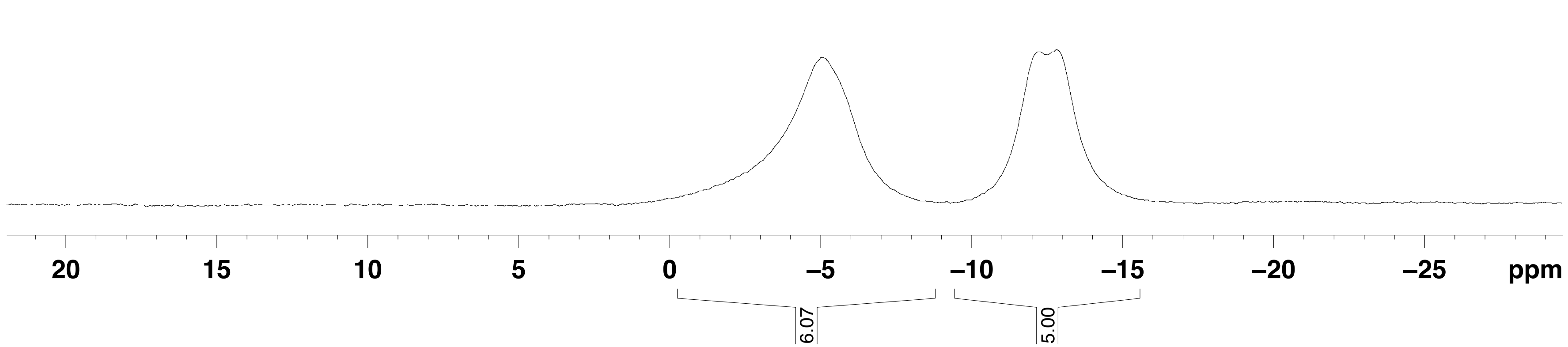

Penta–CF3–styrene–acid chloride product 30 mg in 0.6 ml acetonitrile–d3
11B{1H} NMR, 160 MHz, 23 C

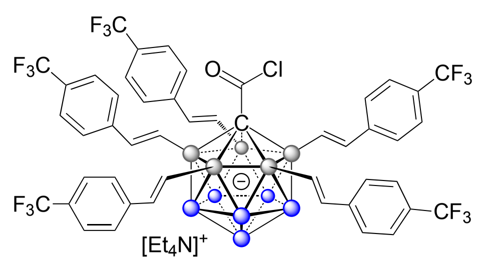

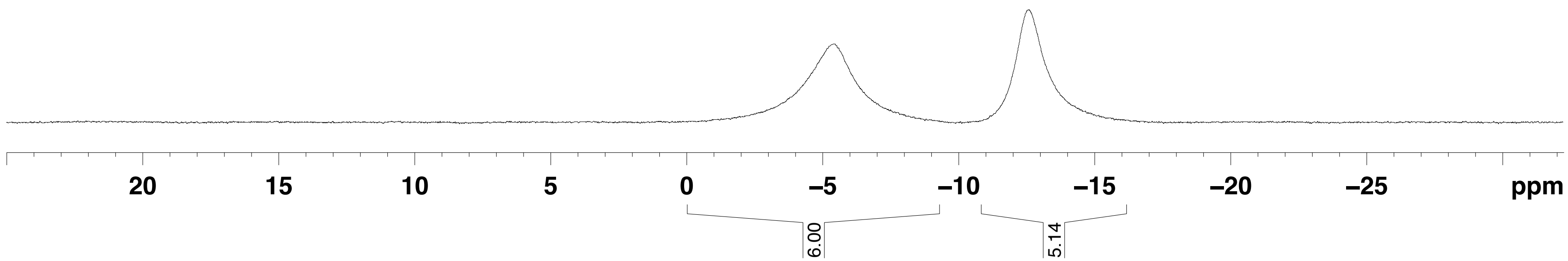


```
Current Data Parameters
NAME     Penta-CF3-styrine-acid chl
EXPNO                     3
PROCNO                    1

F2 - Acquisition Parameters
Date_              20181208
Time                   2.24
INSTRUM               spect
PROBHD   5 mm PABBO BB-
PULPROG             zgpg30
TD                    65536
SOLVENT               CD3CN
NS                      128
DS                        0
SWH           32051.281 Hz
FIDRES         0.489064 Hz
AQ            1.0223616 sec
RG                      203
DW               15.600 usec
DE                 6.50 usec
TE                296.9 K
D1           1.00000000 sec
D11          0.03000000 sec

======== CHANNEL f1 ========
NUC1                    11B
P1                 13.10 usec
PLW1        95.00000000 W
SFO1        160.4615790 MHz

======== CHANNEL f2 ========
CPDPRG[2            waltz16
NUC2                     1H
PCPD2              80.00 usec
PLW2        19.00000000 W
PLW12        0.40639001 W
PLW13        0.26008999 W
SFO2        500.1325007 MHz

F2 - Processing parameters
SI                    32768
SF          160.4615790 MHz
WDW                      EM
SSB      0
LB                 3.00 Hz
GB       0
PC                     1.40
```

Penta–CF3–styrene–acid chloride product 30 mg in 0.6 ml acetonitrile–d3*
13C{1H} NMR, 126 MHz, 23 C

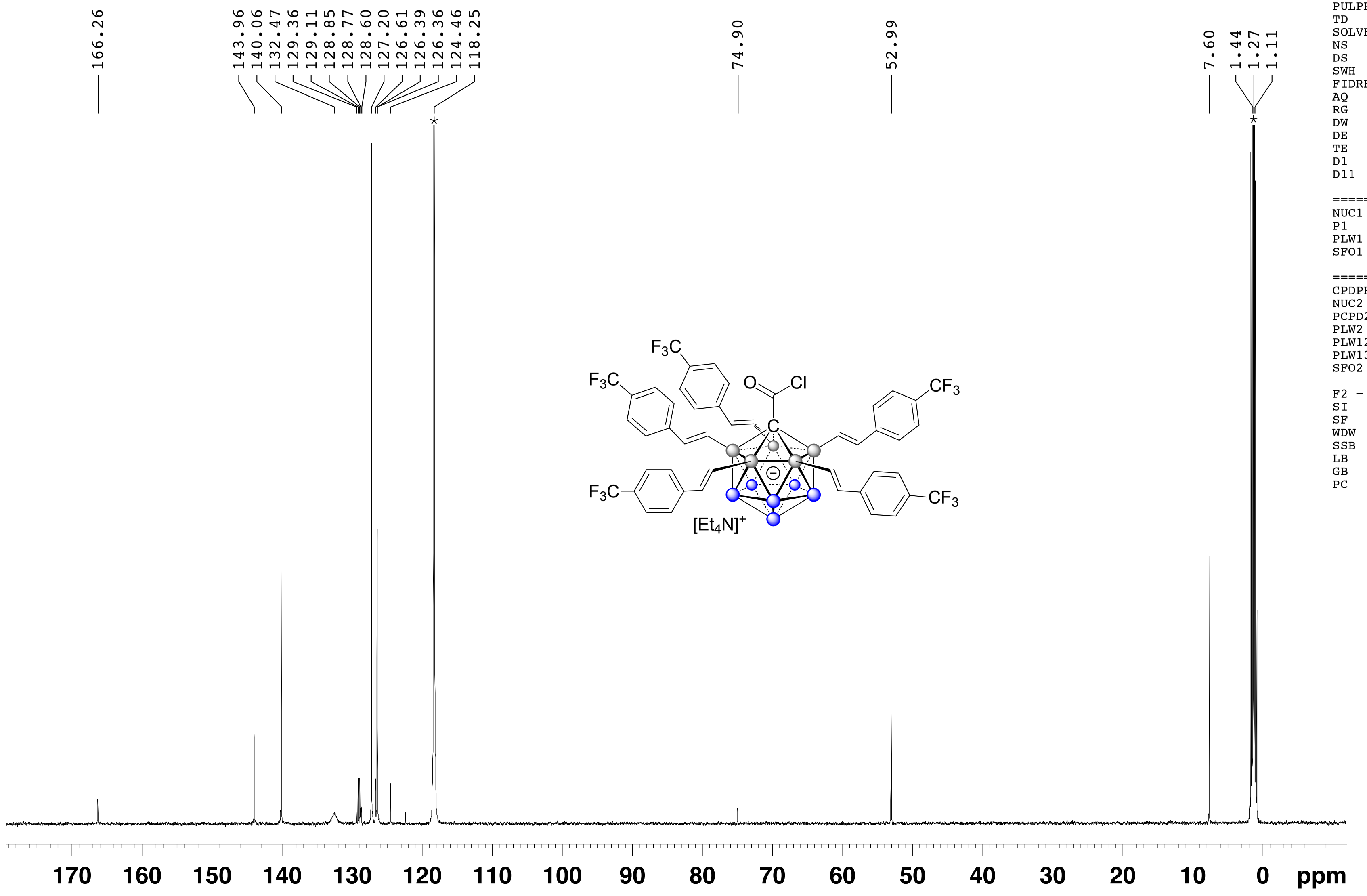


```
Current Data Parameters
NAME     Penta-CF3-styrine-acid chl
EXPNO                 4
PROCNO                1

F2 - Acquisition Parameters
Date_          20181208
Time               4.31
INSTRUM           spect
PROBHD   5 mm PABBO BB-
PULPROG         zgpg30
TD               65536
SOLVENT           CD3CN
NS                3100
DS                   4
SWH          37878.789 Hz
FIDRES        0.577984 Hz
AQ           0.8650752 sec
RG                 203
DW              13.200 usec
DE                6.50 usec
TE               295.4 K
D1          1.50000000 sec
D11         0.03000000 sec

======== CHANNEL f1 ========
NUC1               13C
P1               10.50 usec
PLW1       95.00000000 W
SFO1       125.7716224 MHz

======== CHANNEL f2 ========
CPDPRG[2        waltz16
NUC2                1H
PCPD2            80.00 usec
PLW2       19.00000000 W
PLW12       0.40639001 W
PLW13       0.26008999 W
SFO2       500.1320005 MHz

F2 - Processing parameters
SI               32768
SF         125.7576712 MHz
WDW                 EM
SSB      0
LB                2.00 Hz
GB       0
PC                1.40
```

Penta–4–CF3–styrene product 50 mg in 0.6 ml acetone–d6*
1H{11B} NMR, 500 MHz, 23 C

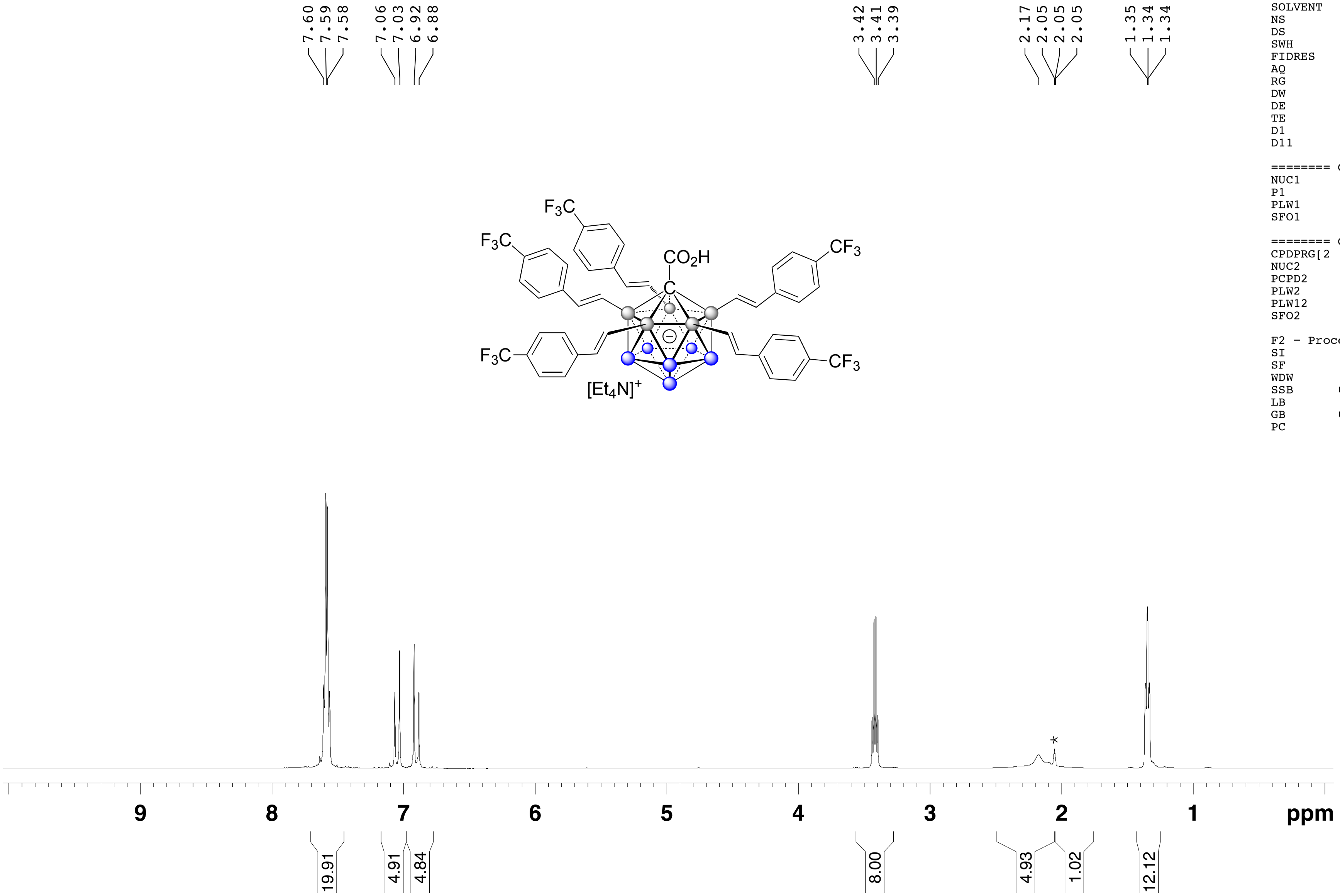


```
Current Data Parameters
NAME     penta-4-CF3-styrene
EXPNO                    1
PROCNO                   1

F2 - Acquisition Parameters
Date_          20180529
Time              22.50
INSTRUM           spect
PROBHD   5 mm PABBO BB-
PULPROG          zgig30
TD                65536
SOLVENT         Acetone
NS                   16
DS                    0
SWH           12500.000 Hz
FIDRES         0.190735 Hz
AQ            2.6214399 sec
RG                 22.6
DW               40.000 usec
DE                 6.50 usec
TE                296.4 K
D1           5.00000000 sec
D11          0.03000000 sec

======== CHANNEL f1 ========
NUC1                 1H
P1                11.70 usec
PLW1        19.00000000 W
SFO1        500.1335009 MHz

======== CHANNEL f2 ========
CPDPRG[2           garp
NUC2                11B
PCPD2            100.00 usec
PLW2        95.00000000 W
PLW12        1.63030005 W
SFO2        160.4615690 MHz

F2 - Processing parameters
SI                65536
SF          500.1300100 MHz
WDW                  EM
SSB      0
LB                 1.00 Hz
GB       0
PC                 1.00
```

Penta–CF3–styrene product 50 mg in 0.6 ml acetone–d6
11B NMR, 160 MHz, 23 C

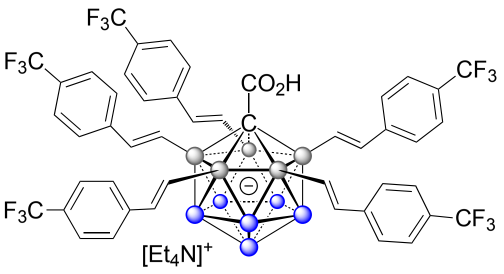

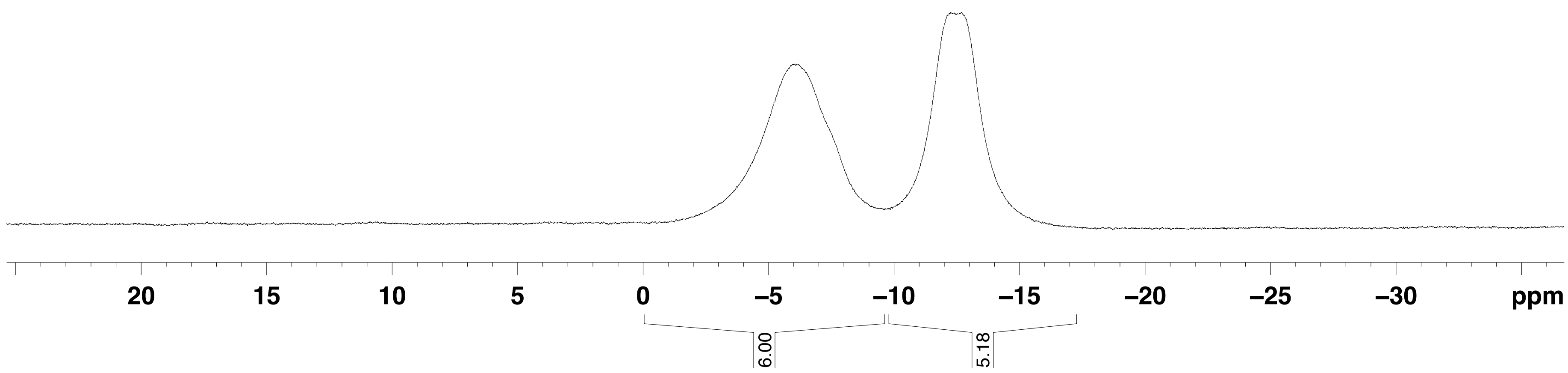


```
Current Data Parameters
NAME     penta-4-CF3-styrene
EXPNO                     2
PROCNO                    1

F2 - Acquisition Parameters
Date_            20180529
Time                22.53
INSTRUM             spect
PROBHD   5 mm PABBO BB-
PULPROG              zg30
TD                  64098
SOLVENT           Acetone
NS                     64
DS                      0
SWH             32051.281 Hz
FIDRES           0.500036 Hz
AQ              0.9999288 sec
RG                    203
DW                 15.600 usec
DE                   6.50 usec
TE                  295.9 K
D1             1.00000000 sec

======== CHANNEL f1 ========
NUC1                  11B
P1                  13.10 usec
PLW1          95.00000000 W
SFO1          160.4615792 MHz

F2 - Processing parameters
SI                  32768
SF            160.4615790 MHz
WDW                    EM
SSB      0
LB                   3.00 Hz
GB       0
PC                   1.40
```

Penta–4–CF3–styrene product 50 mg in 0.6 ml acetone–d6
11B{1H} NMR, 160 MHz, 23 C

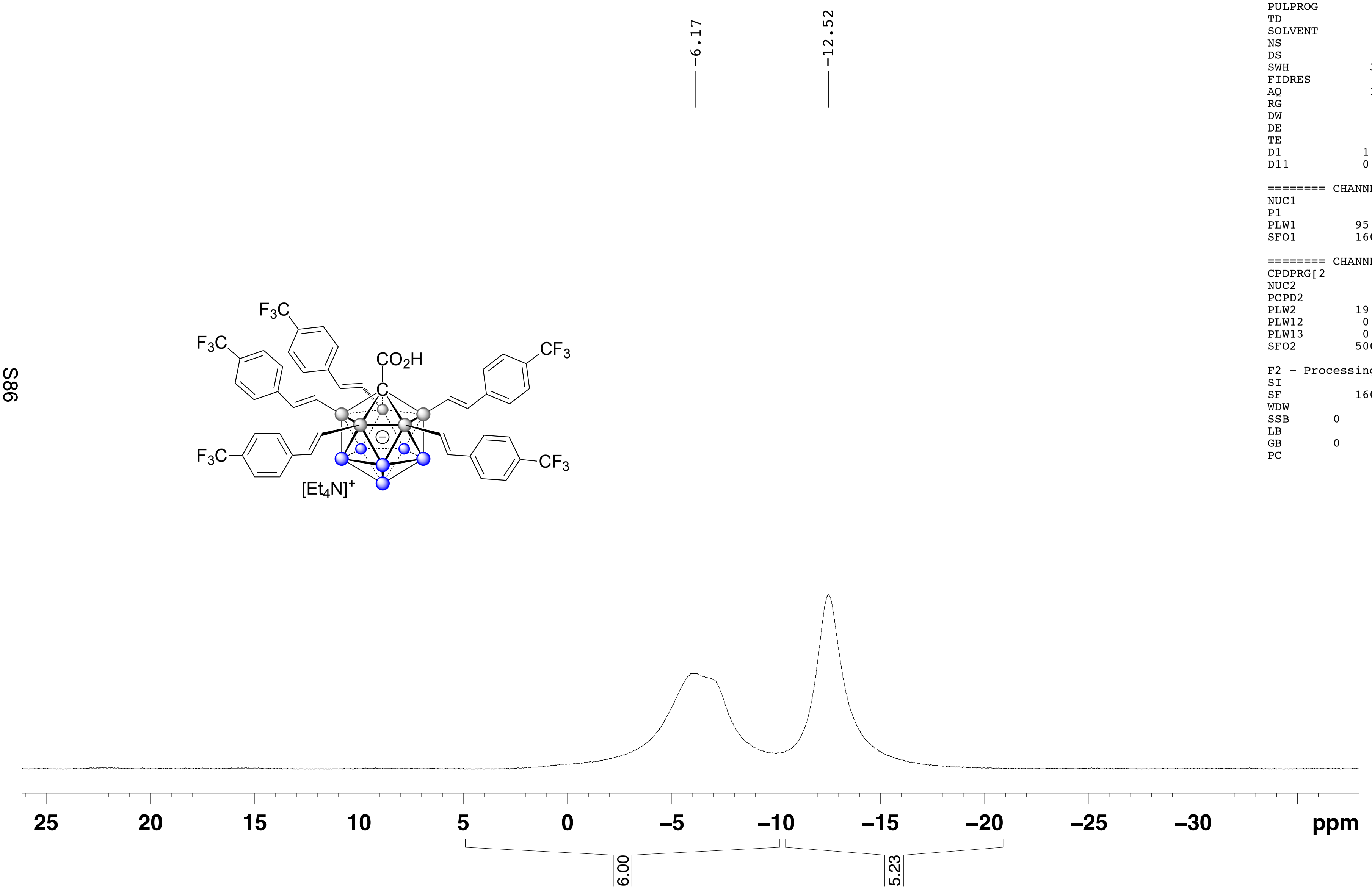


```
Current Data Parameters
NAME     penta-4-CF3-styrene
EXPNO                  3
PROCNO                 1

F2 - Acquisition Parameters
Date_           20180529
Time               22.56
INSTRUM            spect
PROBHD   5 mm PABBO BB-
PULPROG           zgpg30
TD                 65536
SOLVENT          Acetone
NS                    64
DS                     0
SWH            32051.281 Hz
FIDRES          0.489064 Hz
AQ             1.0223616 sec
RG                   203
DW                15.600 usec
DE                  6.50 usec
TE                 296.1 K
D1            1.00000000 sec
D11           0.03000000 sec

======== CHANNEL f1 ========
NUC1                 11B
P1                 13.10 usec
PLW1         95.00000000 W
SFO1         160.4615790 MHz

======== CHANNEL f2 ========
CPDPRG[2         waltz16
NUC2                  1H
PCPD2              80.00 usec
PLW2         19.00000000 W
PLW12         0.40639001 W
PLW13         0.26008999 W
SFO2         500.1325007 MHz

F2 - Processing parameters
SI                 32768
SF           160.4615790 MHz
WDW                   EM
SSB      0
LB                  4.00 Hz
GB       0
PC                  1.40
```

Penta–4–CF3–styrene product 50 mg in 0.6 ml acetone–d6*
13C{1H} NMR, 125 MHz, 23 C

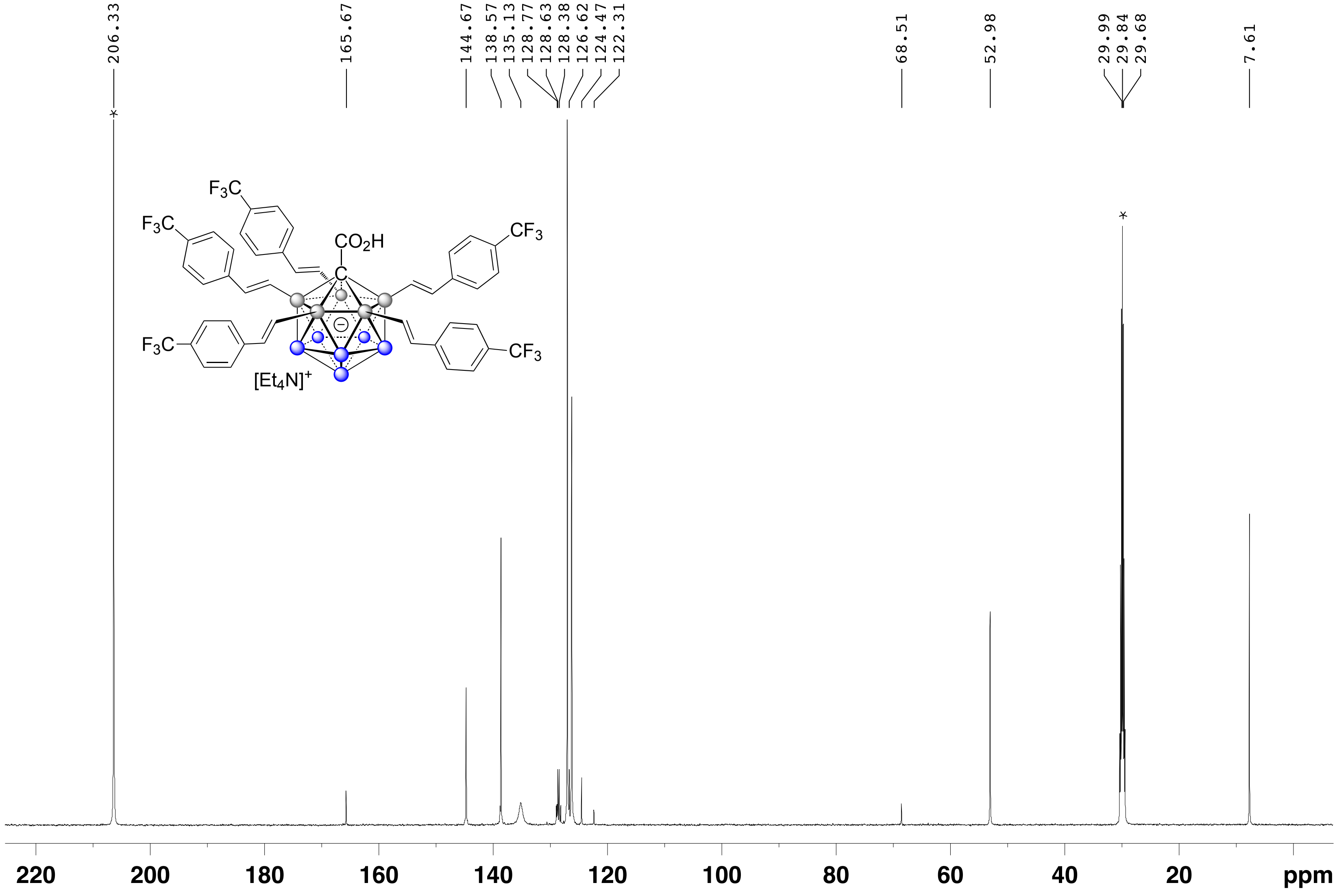


```
Current Data Parameters
NAME      penta-4-CF3-styrene
EXPNO                    4
PROCNO                   1

F2 - Acquisition Parameters
Date_           20180530
Time                0.20
INSTRUM            spect
PROBHD   5 mm PABBO BB-
PULPROG           zgpg30
TD                 65536
SOLVENT          Acetone
NS                  2050
DS                     4
SWH           37878.789 Hz
FIDRES         0.577984 Hz
AQ            0.8650752 sec
RG                   203
DW                13.200 usec
DE                  6.50 usec
TE                 296.6 K
D1            1.50000000 sec
D11           0.03000000 sec

======== CHANNEL f1 ========
NUC1                 13C
P1                 10.50 usec
PLW1         95.00000000 W
SFO1         125.7716224 MHz

======== CHANNEL f2 ========
CPDPRG[2         waltz16
NUC2                  1H
PCPD2              80.00 usec
PLW2         19.00000000 W
PLW12         0.40639001 W
PLW13         0.26008999 W
SFO2         500.1320005 MHz

F2 - Processing parameters
SI                 32768
SF           125.7576822 MHz
WDW                   EM
SSB      0
LB                  5.00 Hz
GB       0
PC                  1.40
```

Penta–4–CN–styrene product 40 mg in 0.6 ml acetone–d6*
1H{11B} NMR, 400 MHz, 23 C

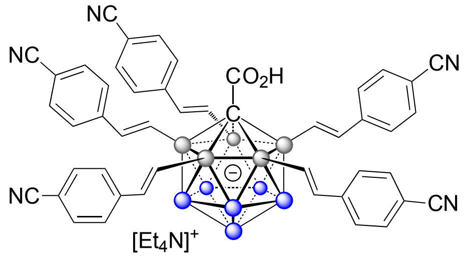

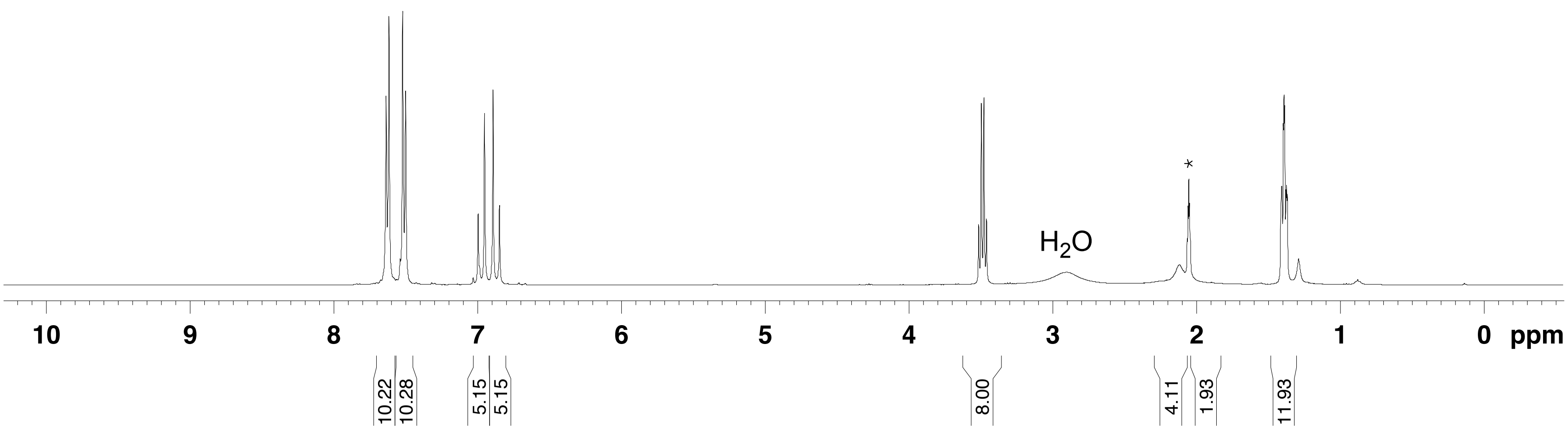


```
Current Data Parameters
NAME     penta-4-cn-styrene
EXPNO                     1
PROCNO                    1

F2 - Acquisition Parameters
Date_          20180702
Time               0.55
INSTRUM           spect
PROBHD   5 mm PABBO BB/
PULPROG          zgig30
TD                16384
SOLVENT         Acetone
NS                   16
DS                    4
SWH            8012.820 Hz
FIDRES         0.489064 Hz
AQ            1.0223616 sec
RG                107.6
DW               62.400 usec
DE                 6.50 usec
TE                410.1 K
D1           1.00000000 sec
D11          0.03000000 sec
TD0                   1

======== CHANNEL f1 ========
NUC1                 1H
P1                15.00 usec
PLW1        12.50000000 W
SFO1        400.1320007 MHz

======== CHANNEL f2 ========
CPDPRG[2          garp4
NUC2                11B
PCPD2             90.00 usec
PLW2        52.96599960 W
PLW12        0.64477998 W
SFO2        128.3776050 MHz

F2 - Processing parameters
SI                32768
SF          400.1300073 MHz
WDW                  EM
SSB      0
LB                 1.00 Hz
GB       0
PC                 1.40
```

Penta–CN–styrene product 40 mg in 0.6 ml acetone–d6
11B NMR, 128 MHz, 23 C

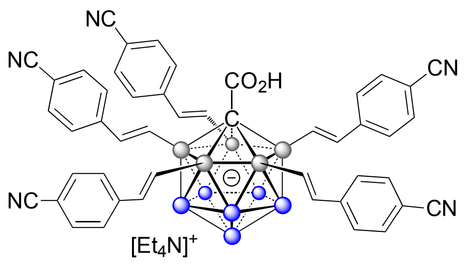

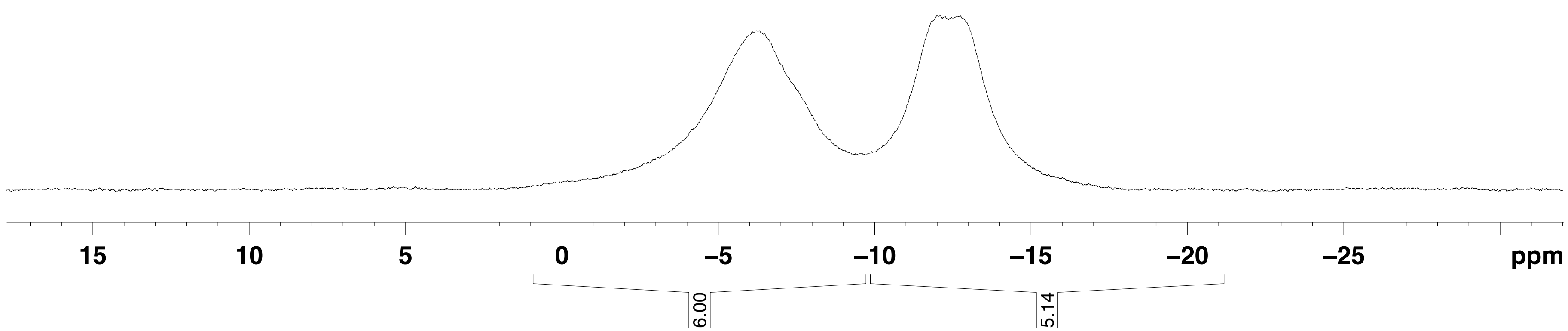


```
Current Data Parameters
NAME      penta-4-cn-styrene
EXPNO                  2
PROCNO                 1

F2 - Acquisition Parameters
Date_           20180702
Time                1.00
INSTRUM            spect
PROBHD   5 mm PABBO BB/
PULPROG               zg
TD                 65536
SOLVENT          Acetone
NS                   128
DS                     4
SWH            25510.203 Hz
FIDRES          0.389255 Hz
AQ             1.2845056 sec
RG                193.34
DW                19.600 usec
DE                  6.50 usec
TE                 410.1 K
D1            1.00000000 sec
TD0                    1

======== CHANNEL f1 ========
NUC1                 11B
P1                  9.93 usec
PLW1         52.96599960 W
SFO1         128.3776052 MHz

F2 - Processing parameters
SI                 32768
SF           128.3776050 MHz
WDW                   EM
SSB      0
LB                  4.00 Hz
GB       0
PC                  1.40
```

Penta–4–CN–styrene product 40 mg in 0.6 ml acetone–d6
11B{1H} NMR, 128 MHz, 23 C

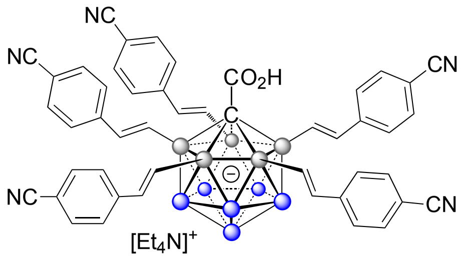

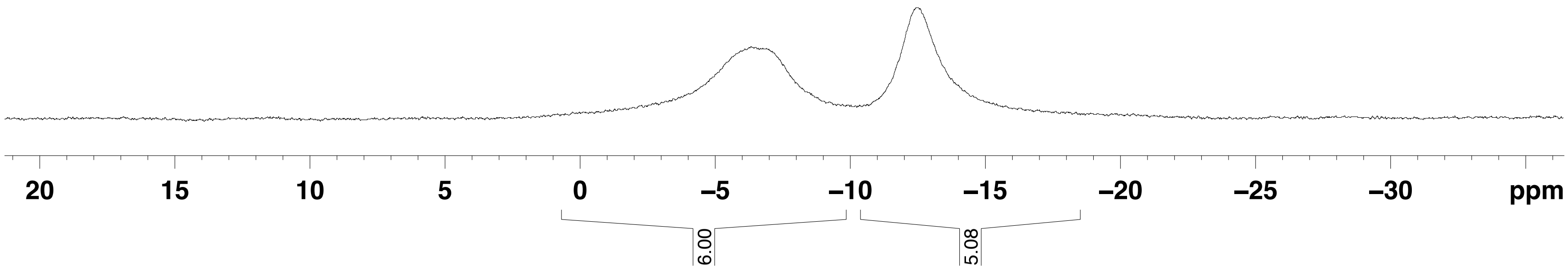


```
Current Data Parameters
NAME     penta-4-cn-styrene
EXPNO                     3
PROCNO                    1

F2 - Acquisition Parameters
Date_            20180702
Time                 1.07
INSTRUM             spect
PROBHD   5 mm PABBO BB/
PULPROG           zgpg30
TD                 65536
SOLVENT          Acetone
NS                   128
DS                     4
SWH            25510.203 Hz
FIDRES          0.389255 Hz
AQ             1.2845056 sec
RG                193.34
DW                19.600 usec
DE                  6.50 usec
TE                 417.0 K
D1            1.00000000 sec
D11           0.03000000 sec
TD0                    1

======== CHANNEL f1 ========
NUC1                  11B
P1                   9.93 usec
PLW1          52.96599960 W
SFO1          128.3776050 MHz

======== CHANNEL f2 ========
CPDPRG[2         waltz16
NUC2                  1H
PCPD2               80.00 usec
PLW2          12.50000000 W
PLW12          0.43945000 W
PLW13          0.28125000 W
SFO2          400.1320007 MHz

F2 - Processing parameters
SI                  32768
SF            128.3776050 MHz
WDW                    EM
SSB      0
LB                   4.00 Hz
GB       0
PC                   1.40
```

Penta–4–CN–styrene product 40 mg in 0.6 ml acetone–d6*
13C{1H} NMR, 128 MHz, 23 C

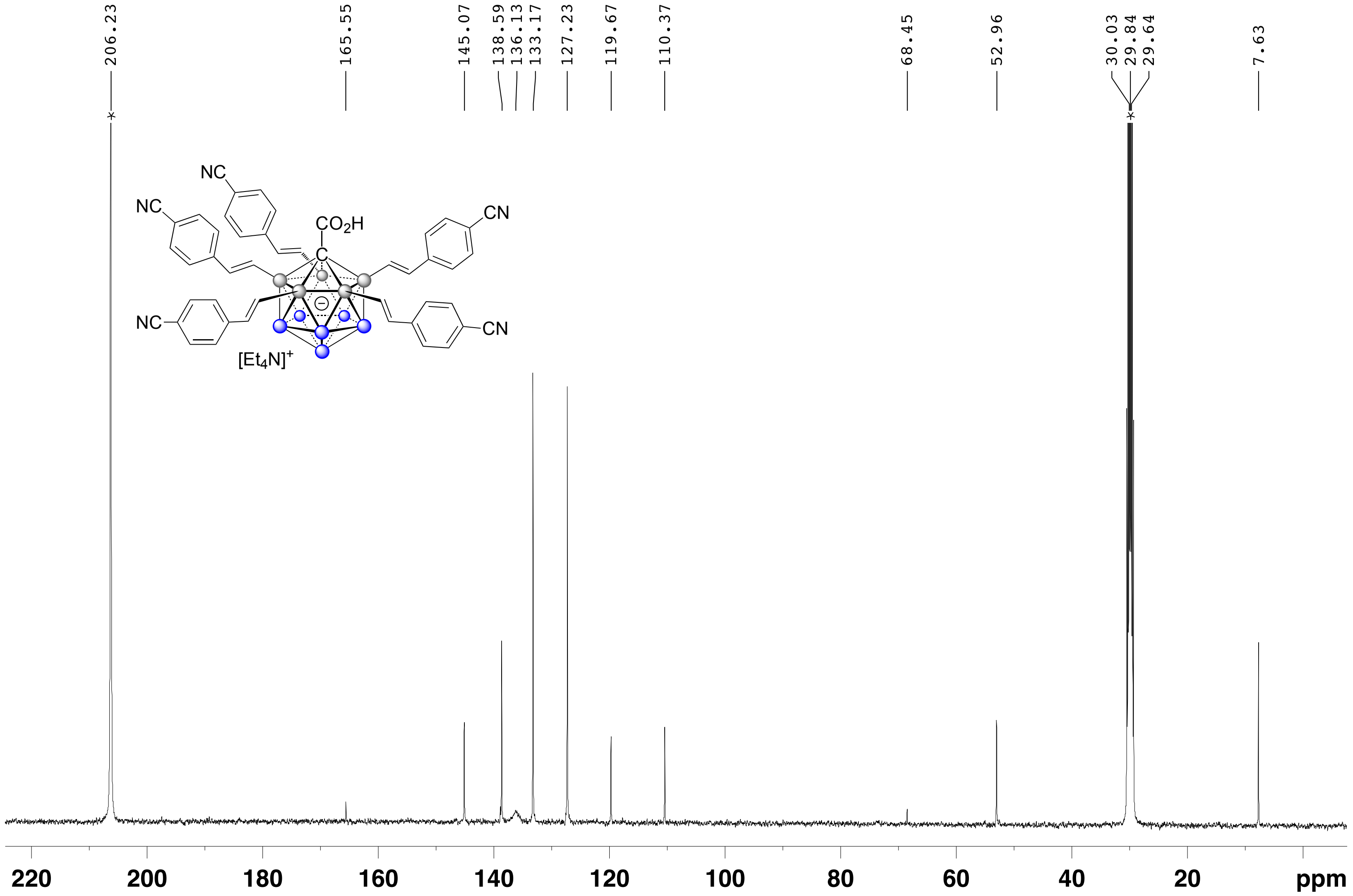


```
Current Data Parameters
NAME      penta-4-cn-styrene-13C
EXPNO                    1
PROCNO                   1

F2 - Acquisition Parameters
Date_             20180702
Time                 10.00
INSTRUM              spect
PROBHD   5 mm PABBO BB/
PULPROG             zgpg30
TD                   65536
SOLVENT            Acetone
NS                    3072
DS                       4
SWH              29761.904 Hz
FIDRES            0.454131 Hz
AQ               1.1010048 sec
RG                  193.34
DW                  16.800 usec
DE                    6.50 usec
TE                   415.0 K
D1              1.50000000 sec
D11             0.03000000 sec
TD0                      1

======== CHANNEL f1 ========
NUC1                   13C
P1                   10.00 usec
PLW1           53.00000000 W
SFO1          100.6228293 MHz

======== CHANNEL f2 ========
CPDPRG[2           waltz16
NUC2                    1H
PCPD2                80.00 usec
PLW2           12.50000000 W
PLW12           0.43945000 W
PLW13           0.28125000 W
SFO2          400.1316005 MHz

F2 - Processing parameters
SI                   32768
SF            100.6126819 MHz
WDW                     EM
SSB      0
LB                    4.00 Hz
GB       0
PC                    1.40
```

Penta–C6F5 product 40 mg in 0.6 ml acetone–d6 *
1H{11B} NMR, 500 MHz, 23 C

```
Current Data Parameters
NAME          penta-C6F5
EXPNO                  1
PROCNO                 1

F2 - Acquisition Parameters
Date_           20181001
Time                5.47
INSTRUM            spect
PROBHD   5 mm PABBO BB-
PULPROG           zgig30
TD                 65536
SOLVENT          Acetone
NS                    16
DS                     0
SWH            12500.000 Hz
FIDRES          0.190735 Hz
AQ             2.6214399 sec
RG                   114
DW                40.000 usec
DE                  6.50 usec
TE                 296.2 K
D1            5.00000000 sec
D11           0.03000000 sec
TD0                    1

======== CHANNEL f1 ========
SFO1        500.1335009 MHz
NUC1                 1H
P1                12.00 usec
PLW1        19.00000000 W

======== CHANNEL f2 ========
SFO2        160.4615690 MHz
NUC2                 11B
CPDPRG[2            garp
PCPD2             100.00 usec
PLW2        95.00000000 W
PLW12        1.63030005 W

F2 - Processing parameters
SI                 65536
SF           500.1300088 MHz
WDW                   EM
SSB      0
LB                  1.00 Hz
GB       0
PC                  1.00
```

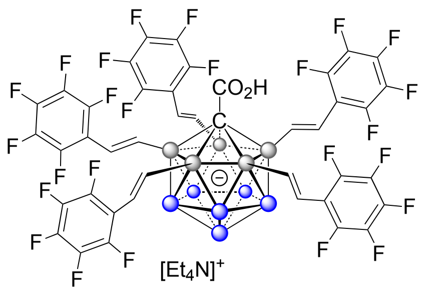

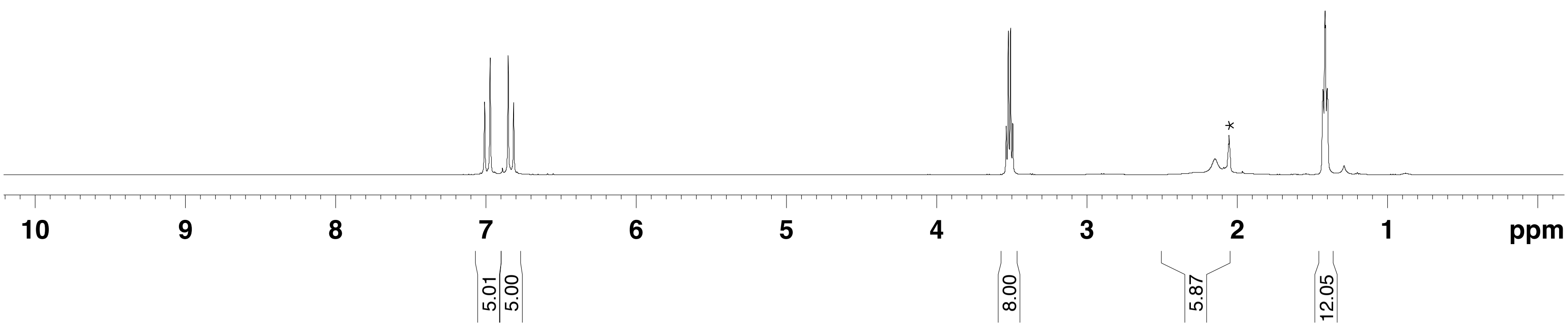

Penta–C6F5 product 40 mg in 0.6 ml acetone–d6
11B NMR, 160 MHz, 23 C

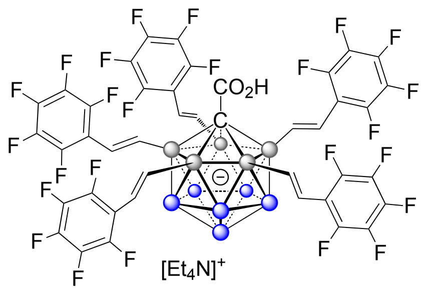

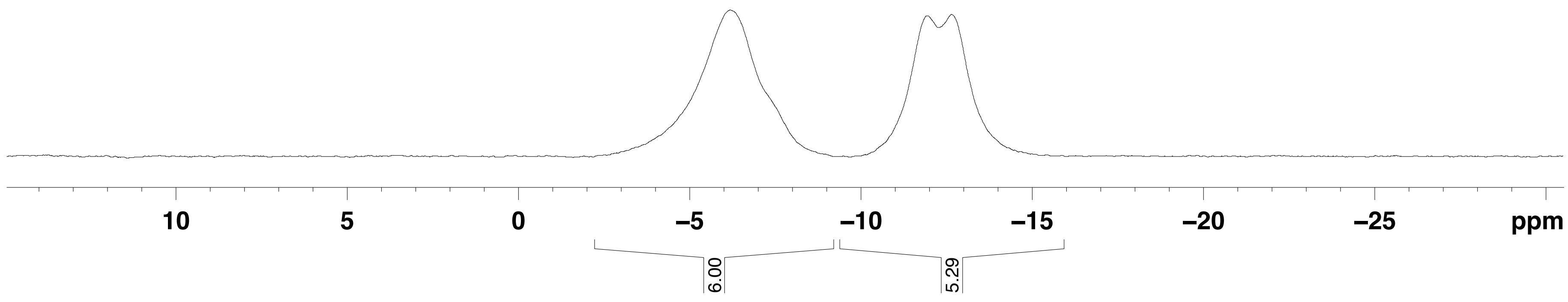


```
Current Data Parameters
NAME          penta-C6F5
EXPNO                  2
PROCNO                 1

F2 - Acquisition Parameters
Date_           20181001
Time                5.50
INSTRUM            spect
PROBHD   5 mm PABBO BB-
PULPROG             zg30
TD                 64098
SOLVENT          Acetone
NS                    64
DS                     0
SWH            32051.281 Hz
FIDRES          0.500036 Hz
AQ             0.9999288 sec
RG                   203
DW                15.600 usec
DE                  6.50 usec
TE                 296.2 K
D1            1.00000000 sec
TD0                    1

======== CHANNEL f1 ========
SFO1         160.4615792 MHz
NUC1                 11B
P1                 13.10 usec
PLW1         95.00000000 W

F2 - Processing parameters
SI                 32768
SF           160.4615790 MHz
WDW                   EM
SSB      0
LB                 10.00 Hz
GB       0
PC                  1.40
```

Penta–C6F5 product 40 mg in 0.6 ml acetone–d6
11B{1H} NMR, 160 MHz, 23 C

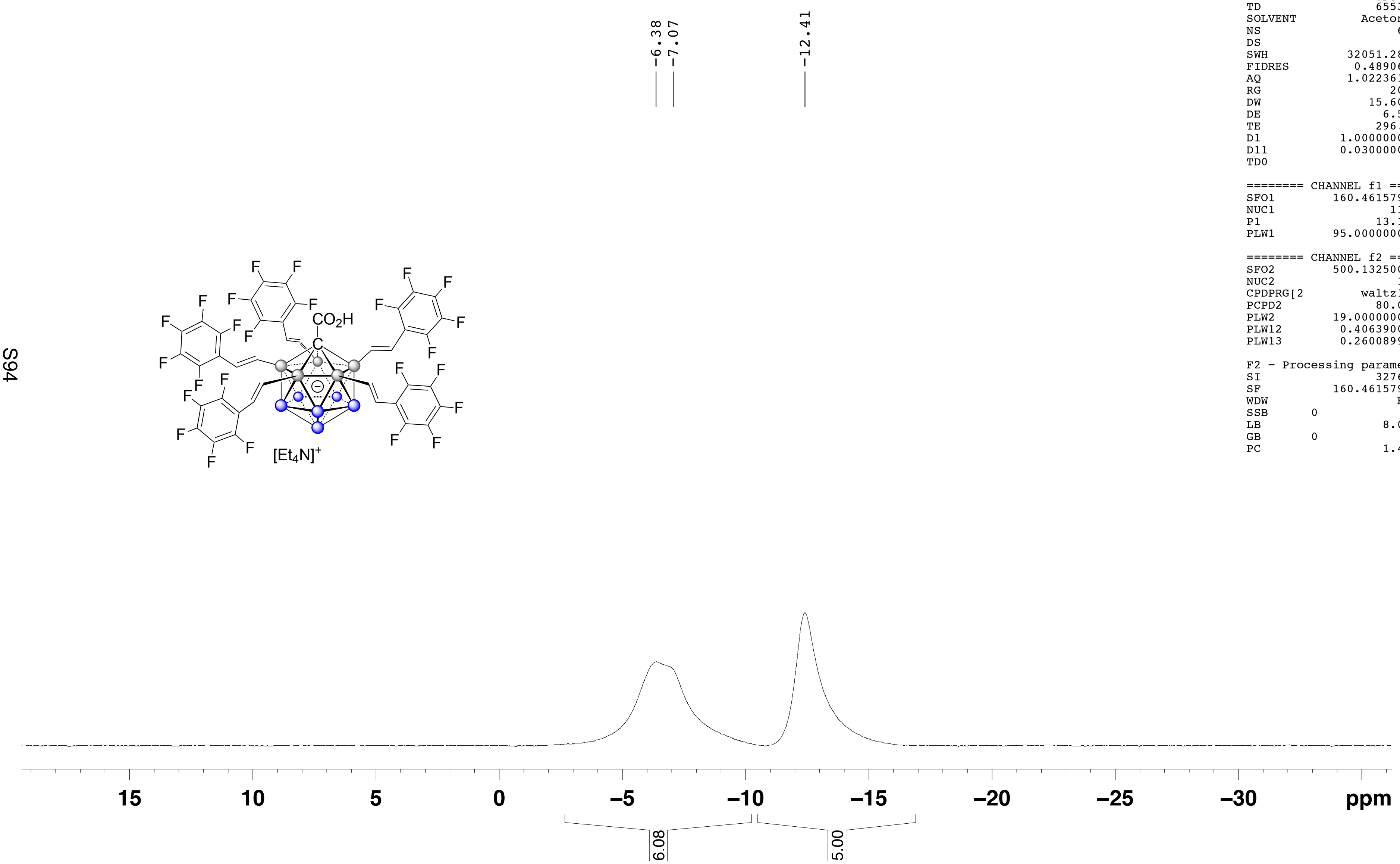


```
Current Data Parameters
NAME          penta-C6F5
EXPNO                  3
PROCNO                 1

F2 - Acquisition Parameters
Date_           20181001
Time                5.53
INSTRUM            spect
PROBHD   5 mm PABBO BB-
PULPROG           zgpg30
TD                 65536
SOLVENT          Acetone
NS                    64
DS                     0
SWH            32051.281 Hz
FIDRES          0.489064 Hz
AQ             1.0223616 sec
RG                   203
DW                15.600 usec
DE                  6.50 usec
TE                 296.3 K
D1            1.00000000 sec
D11           0.03000000 sec
TD0                    1

======== CHANNEL f1 ========
SFO1         160.4615790 MHz
NUC1                 11B
P1                 13.10 usec
PLW1         95.00000000 W

======== CHANNEL f2 ========
SFO2         500.1325007 MHz
NUC2                  1H
CPDPRG[2         waltz16
PCPD2              80.00 usec
PLW2         19.00000000 W
PLW12         0.40639001 W
PLW13         0.26008999 W

F2 - Processing parameters
SI                 32768
SF           160.4615790 MHz
WDW                   EM
SSB      0
LB                  8.00 Hz
GB       0
PC                  1.40
```

Penta–C6F5 product 40 mg in 0.6 ml acetone–d6*
13C{1H} NMR, 126 MHz, 23 C

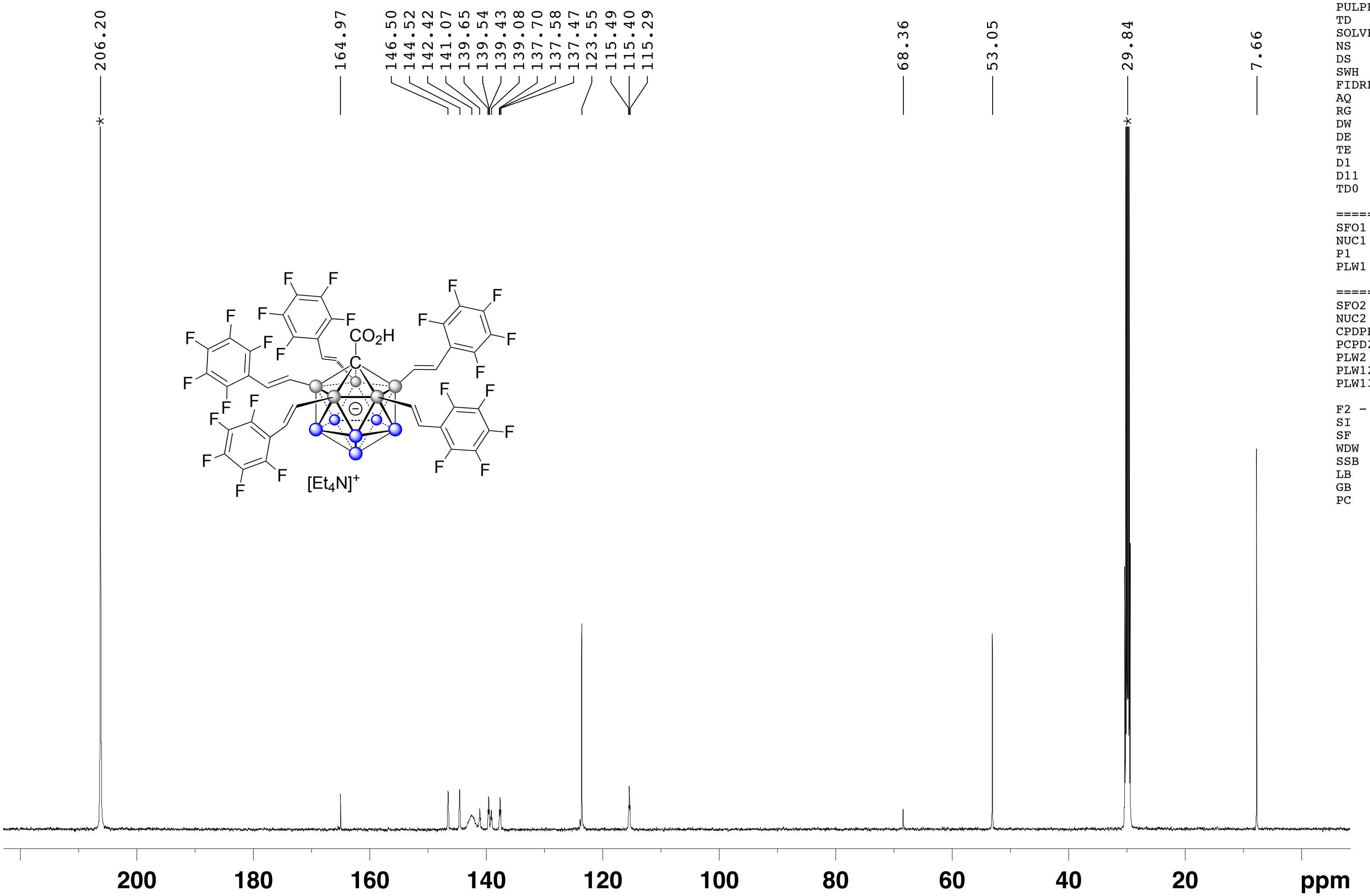


```
Current Data Parameters
NAME            penta-C6F5
EXPNO                    4
PROCNO                   1

F2 - Acquisition Parameters
Date_             20181001
Time                  8.00
INSTRUM              spect
PROBHD    5 mm PABBO BB-
PULPROG            zgpg30
TD                   65536
SOLVENT            Acetone
NS                    3100
DS                       4
SWH             37878.789 Hz
FIDRES           0.577984 Hz
AQ              0.8650752 sec
RG                     203
DW                  13.200 usec
DE                    6.50 usec
TE                   296.3 K
D1              1.50000000 sec
D11             0.03000000 sec
TD0                      1

======== CHANNEL f1 ========
SFO1          125.7716224 MHz
NUC1                   13C
P1                   10.70 usec
PLW1           95.00000000 W

======== CHANNEL f2 ========
SFO2          500.1320005 MHz
NUC2                    1H
CPDPRG[2           waltz16
PCPD2                80.00 usec
PLW2           19.00000000 W
PLW12           0.40639001 W
PLW13           0.26008999 W

F2 - Processing parameters
SI                   32768
SF            125.7576741 MHz
WDW                     EM
SSB      0
LB                    3.00 Hz
GB       0
PC                    1.40
```

penta–CH2Ph product 40 mg in 0.6 ml acetone–d6*
1H{1B} NMR, 400 MHz, 23 C

7.24 7.23 7.22 7.21 7.13 7.12 7.12 6.04 6.02 5.99 5.94 5.90 5.86 3.37 3.35 3.34 3.33 2.90 2.05 1.99 1.97 1.75 1.30 1.30 1.29

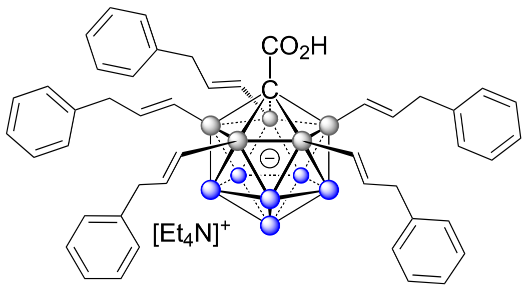

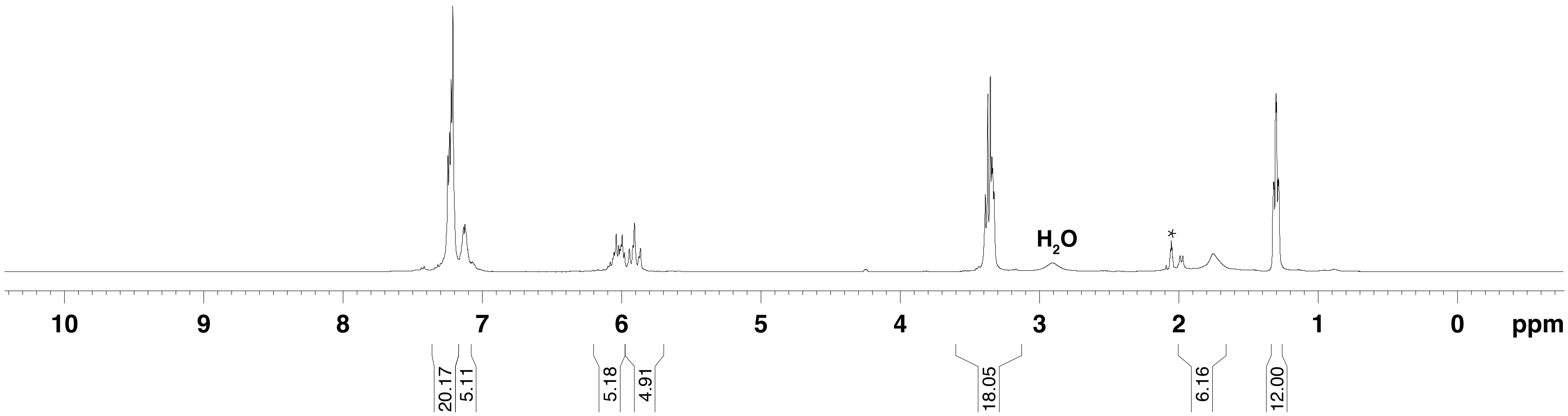


```
Current Data Parameters
NAME      penta-CH2Ph-1H
EXPNO                  1
PROCNO                 1

F2 - Acquisition Parameters
Date_           20180611
Time               15.46
INSTRUM            spect
PROBHD   5 mm PABBO BB/
PULPROG           zgig30
TD                 16384
SOLVENT          Acetone
NS                    16
DS                     4
SWH             8012.820 Hz
FIDRES          0.489064 Hz
AQ             1.0223616 sec
RG                 64.43
DW                62.400 usec
DE                  6.50 usec
TE                 294.4 K
D1            1.00000000 sec
D11           0.03000000 sec
TD0                    1

======== CHANNEL f1 ========
NUC1                  1H
P1                 15.00 usec
PLW1         12.50000000 W
SFO1         400.1320007 MHz

======== CHANNEL f2 ========
CPDPRG[2           garp4
NUC2                 11B
PCPD2              90.00 usec
PLW2         52.96599960 W
PLW12         0.64477998 W
SFO2         128.3776050 MHz

F2 - Processing parameters
SI                 32768
SF           400.1300073 MHz
WDW                   EM
SSB      0
LB                  1.00 Hz
GB       0
PC                  1.40
```

penta–CH2Ph product 40 mg in 0.6 ml acetone–d6
1B NMR, 160 MHz, 23 C

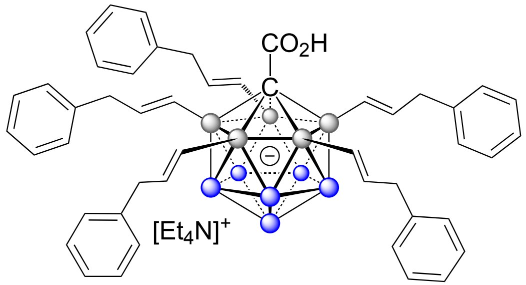

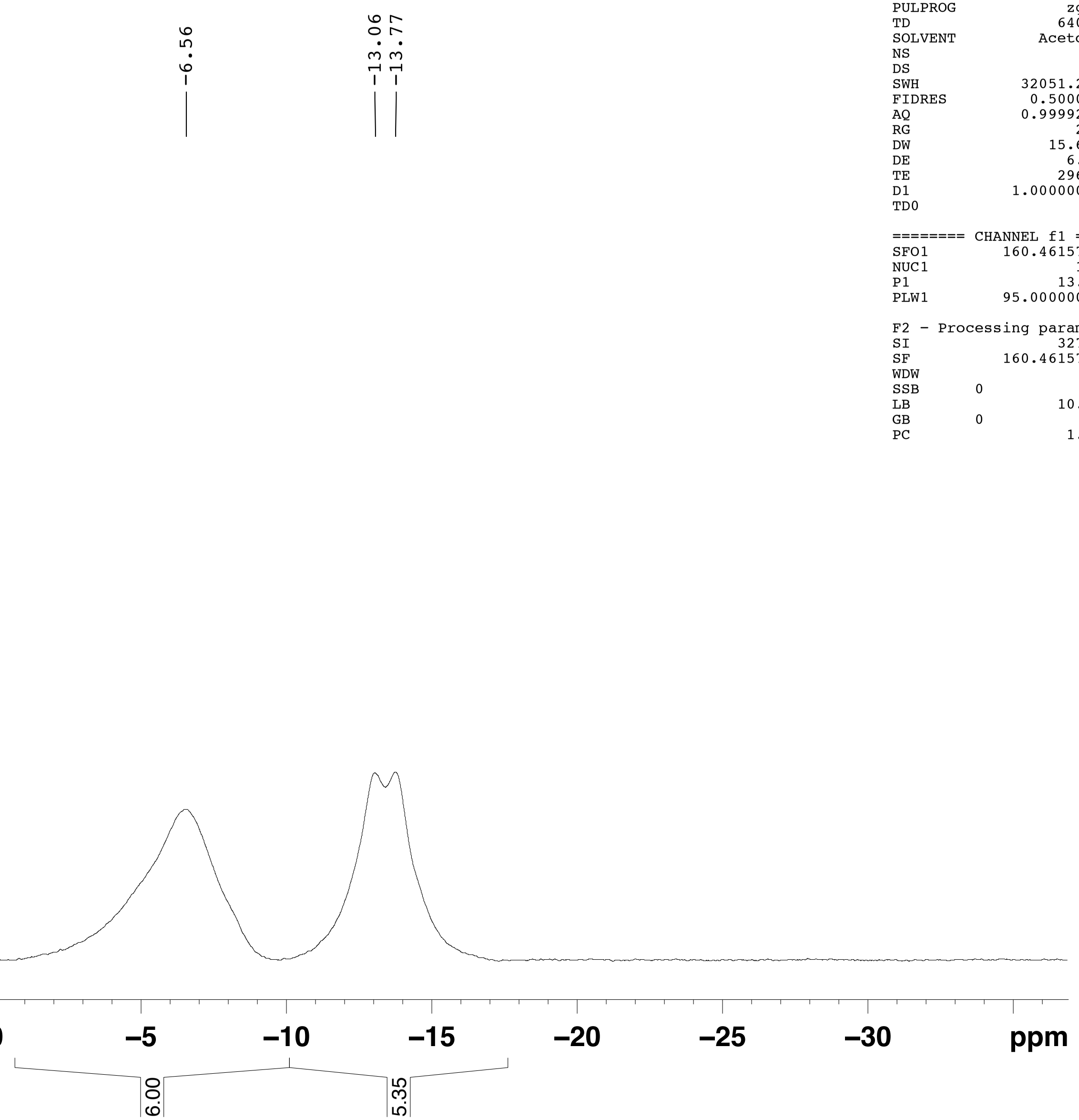


```
Current Data Parameters
NAME        penta-CH2Ph
EXPNO                 3
PROCNO                1

F2 - Acquisition Parameters
Date_          20180824
Time               6.13
INSTRUM           spect
PROBHD   5 mm PABBO BB-
PULPROG            zg30
TD                64098
SOLVENT         Acetone
NS                   64
DS                    0
SWH           32051.281 Hz
FIDRES         0.500036 Hz
AQ            0.9999288 sec
RG                  203
DW               15.600 usec
DE                 6.50 usec
TE                296.0 K
D1           1.00000000 sec
TD0                   1

======== CHANNEL f1 ========
SFO1        160.4615792 MHz
NUC1                11B
P1                13.10 usec
PLW1        95.00000000 W

F2 - Processing parameters
SI                32768
SF          160.4615790 MHz
WDW                  EM
SSB      0
LB                10.00 Hz
GB       0
PC                 1.40
```

penta−CH2Ph product 40 mg in 0.6 ml acetone−d6
11B{1H} NMR, 160 MHz, 23 C

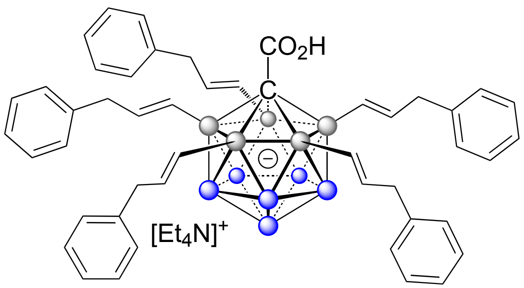

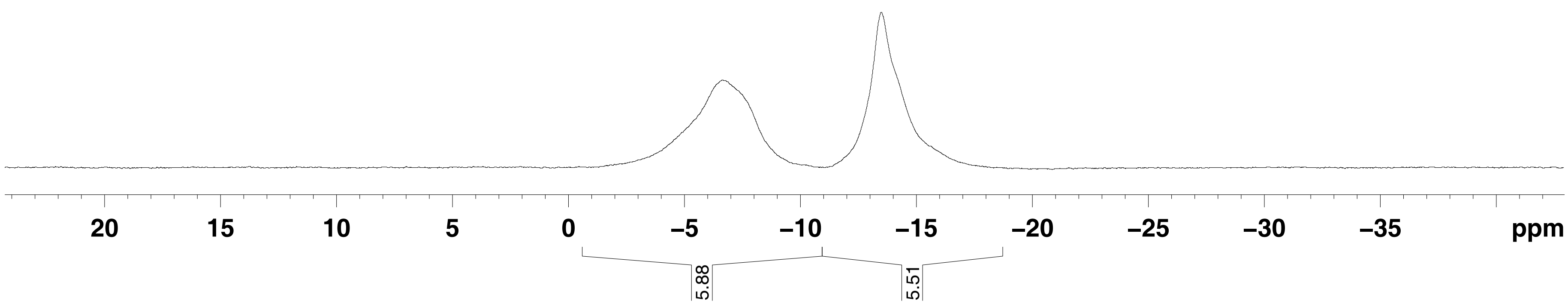


```
Current Data Parameters
NAME          penta-CH2Ph
EXPNO                   4
PROCNO                  1

F2 - Acquisition Parameters
Date_            20180824
Time                 6.16
INSTRUM             spect
PROBHD    5 mm PABBO BB-
PULPROG           zgpg30
TD                  65536
SOLVENT           Acetone
NS                     64
DS                      0
SWH             32051.281 Hz
FIDRES           0.489064 Hz
AQ              1.0223616 sec
RG                    203
DW                 15.600 usec
DE                   6.50 usec
TE                  296.5 K
D1             1.00000000 sec
D11            0.03000000 sec
TD0                     1

======== CHANNEL f1 ========
SFO1          160.4615790 MHz
NUC1                  11B
P1                  13.10 usec
PLW1          95.00000000 W

======== CHANNEL f2 ========
SFO2          500.1325007 MHz
NUC2                   1H
CPDPRG[2          waltz16
PCPD2               80.00 usec
PLW2          19.00000000 W
PLW12          0.40639001 W
PLW13          0.26008999 W

F2 - Processing parameters
SI                  32768
SF            160.4615790 MHz
WDW                    EM
SSB      0
LB                   7.00 Hz
GB       0
PC                   1.40
```

penta−CH2Ph product 40 mg in 0.6 ml acetone−d6*
13C{1H} NMR, 126 MHz, 23 C

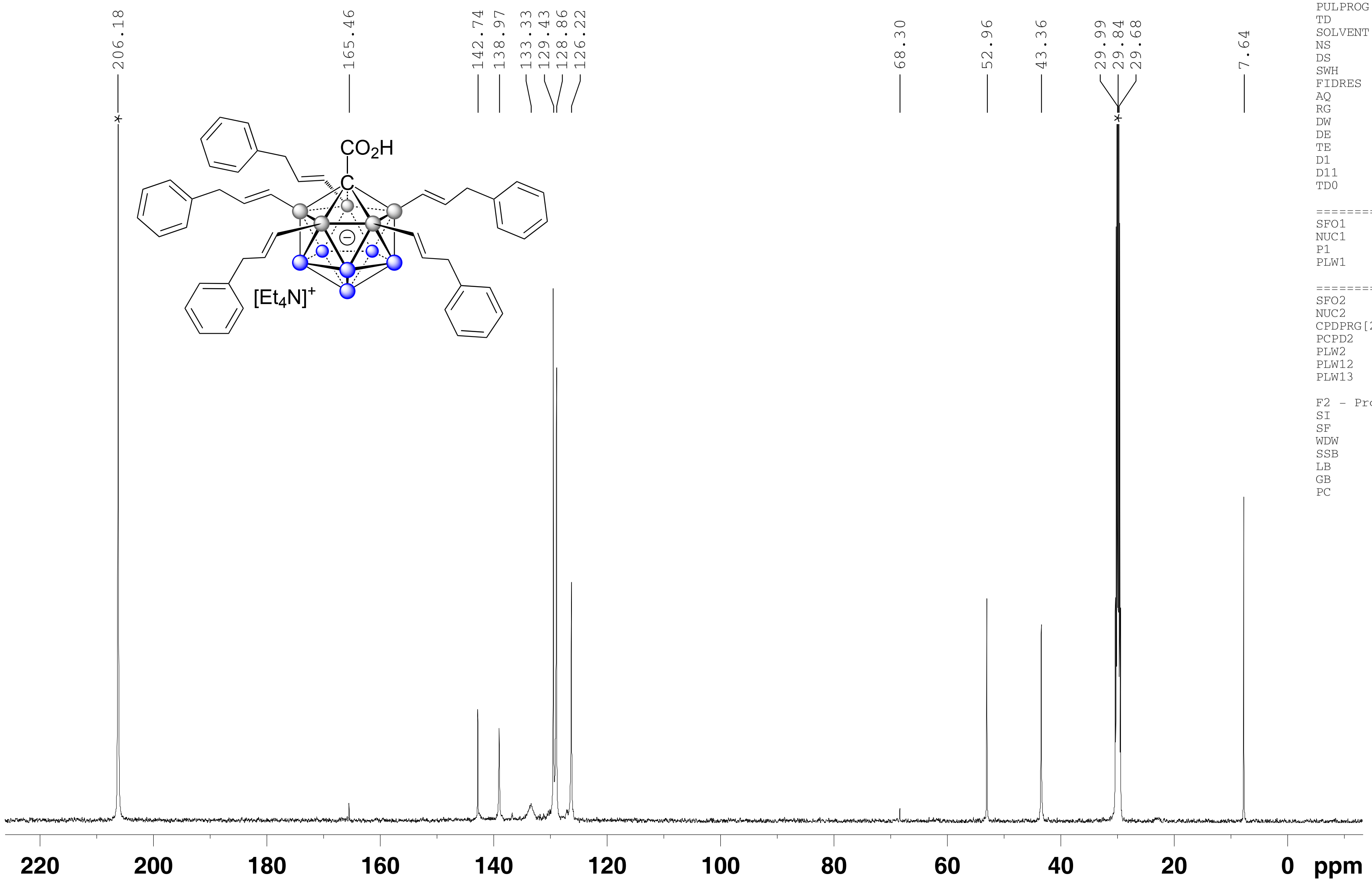


```
Current Data Parameters
NAME          penta-CH2Ph
EXPNO                   2
PROCNO                  1

F2 - Acquisition Parameters
Date_            20180823
Time                17.23
INSTRUM             spect
PROBHD   5 mm PABBO BB-
PULPROG           zgpg30
TD                  65536
SOLVENT           Acetone
NS                   1024
DS                      4
SWH             37878.789 Hz
FIDRES           0.577984 Hz
AQ              0.8650752 sec
RG                    203
DW                 13.200 usec
DE                   6.50 usec
TE                  297.3 K
D1             1.50000000 sec
D11            0.03000000 sec
TD0                     1

======== CHANNEL f1 ========
SFO1          125.7716224 MHz
NUC1                  13C
P1                  10.50 usec
PLW1          95.00000000 W

======== CHANNEL f2 ========
SFO2          500.1320005 MHz
NUC2                   1H
CPDPRG[2          waltz16
PCPD2               80.00 usec
PLW2          19.00000000 W
PLW12          0.40639001 W
PLW13          0.26008999 W

F2 - Processing parameters
SI                  32768
SF            125.7576835 MHz
WDW                    EM
SSB      0
LB                   5.00 Hz
GB       0
PC                   1.40
```

Penta–CH2–C6F5 product 50 mg in 0.6 ml acetone–d6 *
1H{11B} NMR, 400 MHz, 23 C

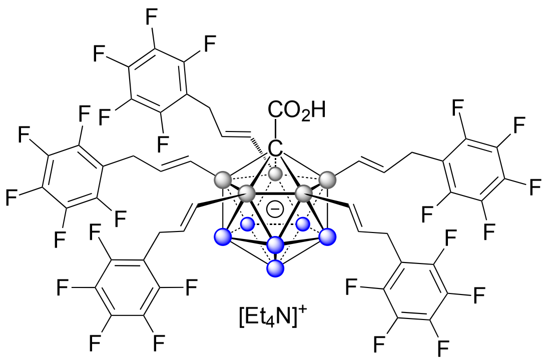


```
Current Data Parameters
NAME        penta-CH2C6F5
EXPNO                   1
PROCNO                  1

F2 - Acquisition Parameters
Date_            20180527
Time                21.31
INSTRUM             spect
PROBHD   5 mm PABBO BB/
PULPROG            zgig30
TD                  16384
SOLVENT           Acetone
NS                     16
DS                      4
SWH              8012.820 Hz
FIDRES           0.489064 Hz
AQ              1.0223616 sec
RG                  78.69
DW                 62.400 usec
DE                   6.50 usec
TE                  294.3 K
D1             1.00000000 sec
D11            0.03000000 sec
TD0                     1

======== CHANNEL f1 ========
NUC1                   1H
P1                  15.00 usec
PLW1          12.50000000 W
SFO1          400.1320007 MHz

======== CHANNEL f2 ========
CPDPRG[2            garp4
NUC2                  11B
PCPD2               90.00 usec
PLW2          52.96599960 W
PLW12          0.64477998 W
SFO2          128.3776050 MHz

F2 - Processing parameters
SI                  32768
SF            400.1300072 MHz
WDW                    EM
SSB      0
LB                   1.00 Hz
GB       0
PC                   1.40
```

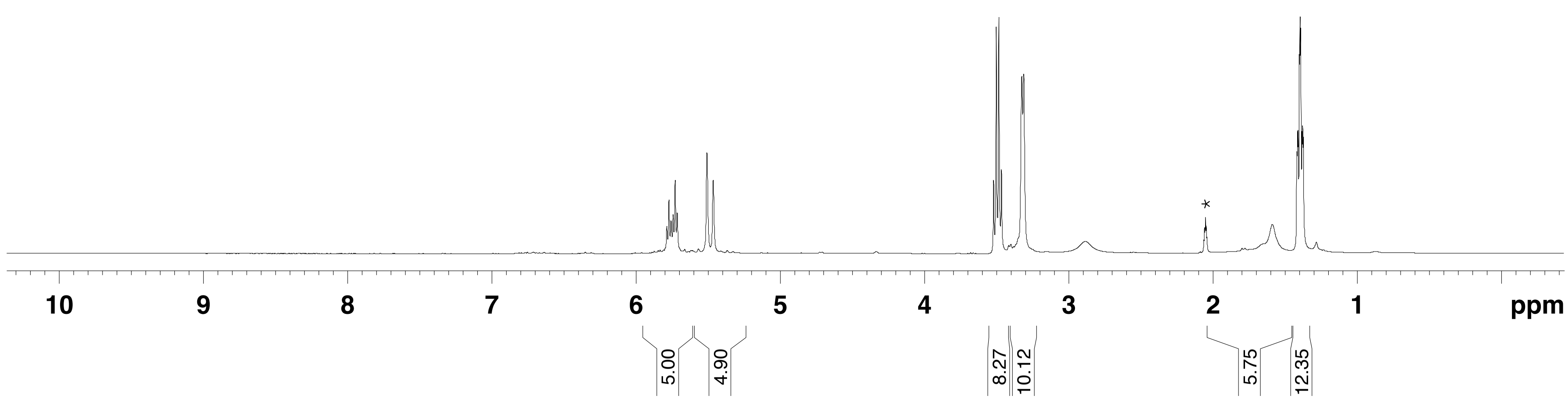

Penta–CH2–C6F5 product 50 mg in 0.6 ml acetone–d6
11B NMR, 128 MHz, 23 C

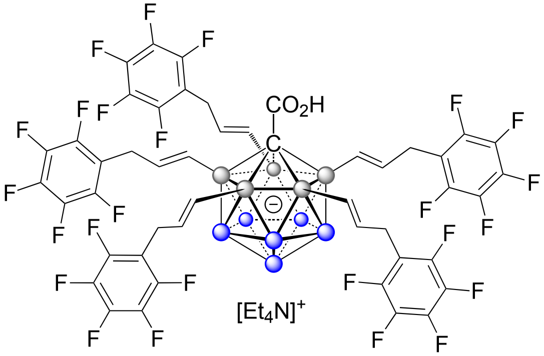

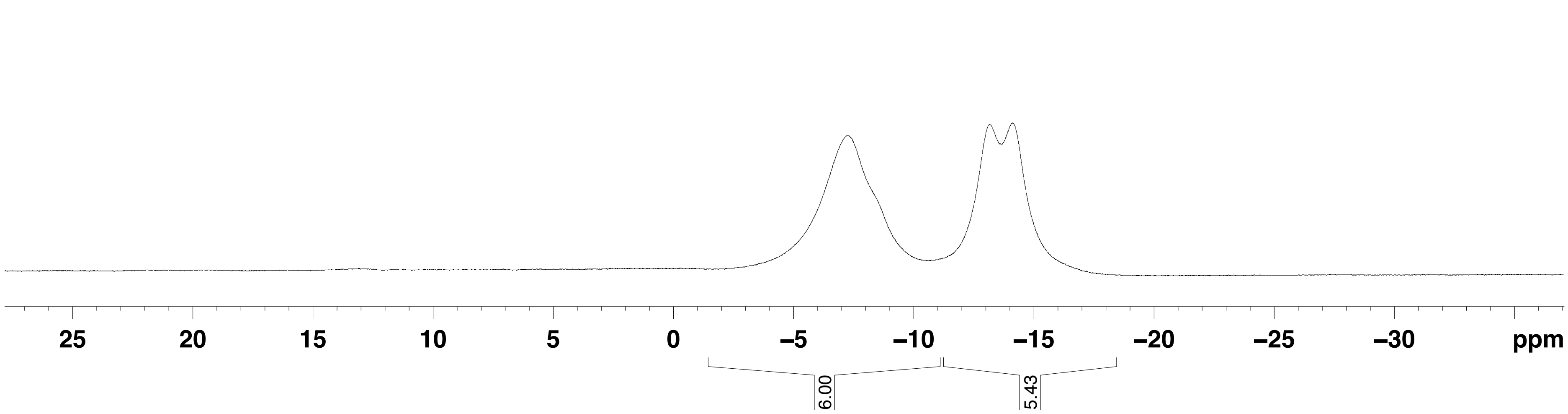


```
Current Data Parameters
NAME      penta-CH2C6F5
EXPNO                 2
PROCNO                1

F2 - Acquisition Parameters
Date_          20180527
Time              21.37
INSTRUM           spect
PROBHD   5 mm PABBO BB/
PULPROG              zg
TD                65536
SOLVENT         Acetone
NS                  128
DS                    4
SWH           25510.203 Hz
FIDRES         0.389255 Hz
AQ            1.2845056 sec
RG               193.34
DW               19.600 usec
DE                 6.50 usec
TE                294.3 K
D1           1.00000000 sec
TD0                   1

======== CHANNEL f1 ========
NUC1                11B
P1                 9.93 usec
PLW1        52.96599960 W
SFO1        128.3776052 MHz

F2 - Processing parameters
SI                32768
SF          128.3776050 MHz
WDW                  EM
SSB      0
LB                 2.00 Hz
GB       0
PC                 1.40
```

Penta–CH2–C6F5 product 50 mg in 0.6 ml acetone–d6
11B{1H} NMR, 128 MHz, 23 C

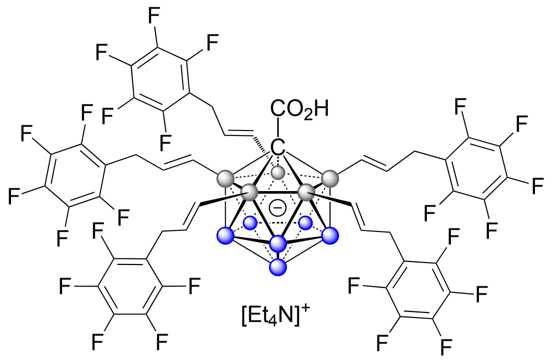

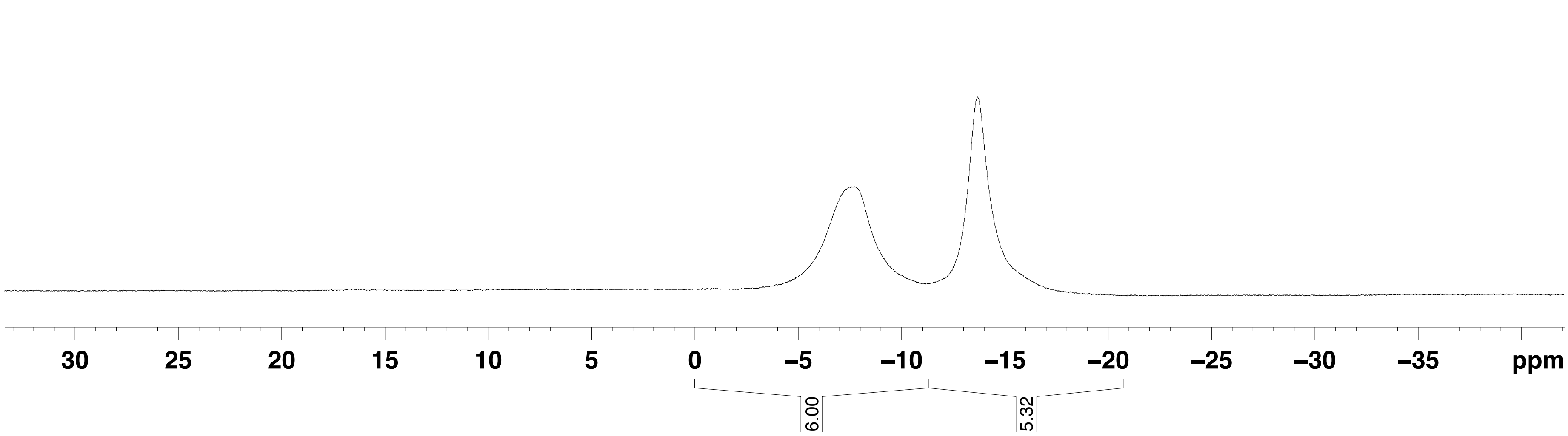


```
Current Data Parameters
NAME       penta-CH2C6F5
EXPNO                  3
PROCNO                 1

F2 - Acquisition Parameters
Date_           20180527
Time               21.43
INSTRUM            spect
PROBHD   5 mm PABBO BB/
PULPROG          zgpg30
TD                 65536
SOLVENT          Acetone
NS                   128
DS                     4
SWH            25510.203 Hz
FIDRES          0.389255 Hz
AQ             1.2845056 sec
RG                193.34
DW                19.600 usec
DE                  6.50 usec
TE                 294.1 K
D1            1.00000000 sec
D11           0.03000000 sec
TD0                    1

======== CHANNEL f1 ========
NUC1                 11B
P1                  9.93 usec
PLW1         52.96599960 W
SFO1         128.3776050 MHz

======== CHANNEL f2 ========
CPDPRG[2         waltz16
NUC2                  1H
PCPD2              80.00 usec
PLW2         12.50000000 W
PLW12         0.43945000 W
PLW13         0.28125000 W
SFO2         400.1320007 MHz

F2 - Processing parameters
SI                 32768
SF           128.3776050 MHz
WDW                   EM
SSB       0
LB                  3.00 Hz
GB        0
PC                  1.40
```

Penta–CH2–C6F5 product 50 mg in 0.6 ml acetone–d6*
13C{1H} NMR, 101 MHz, 23 C

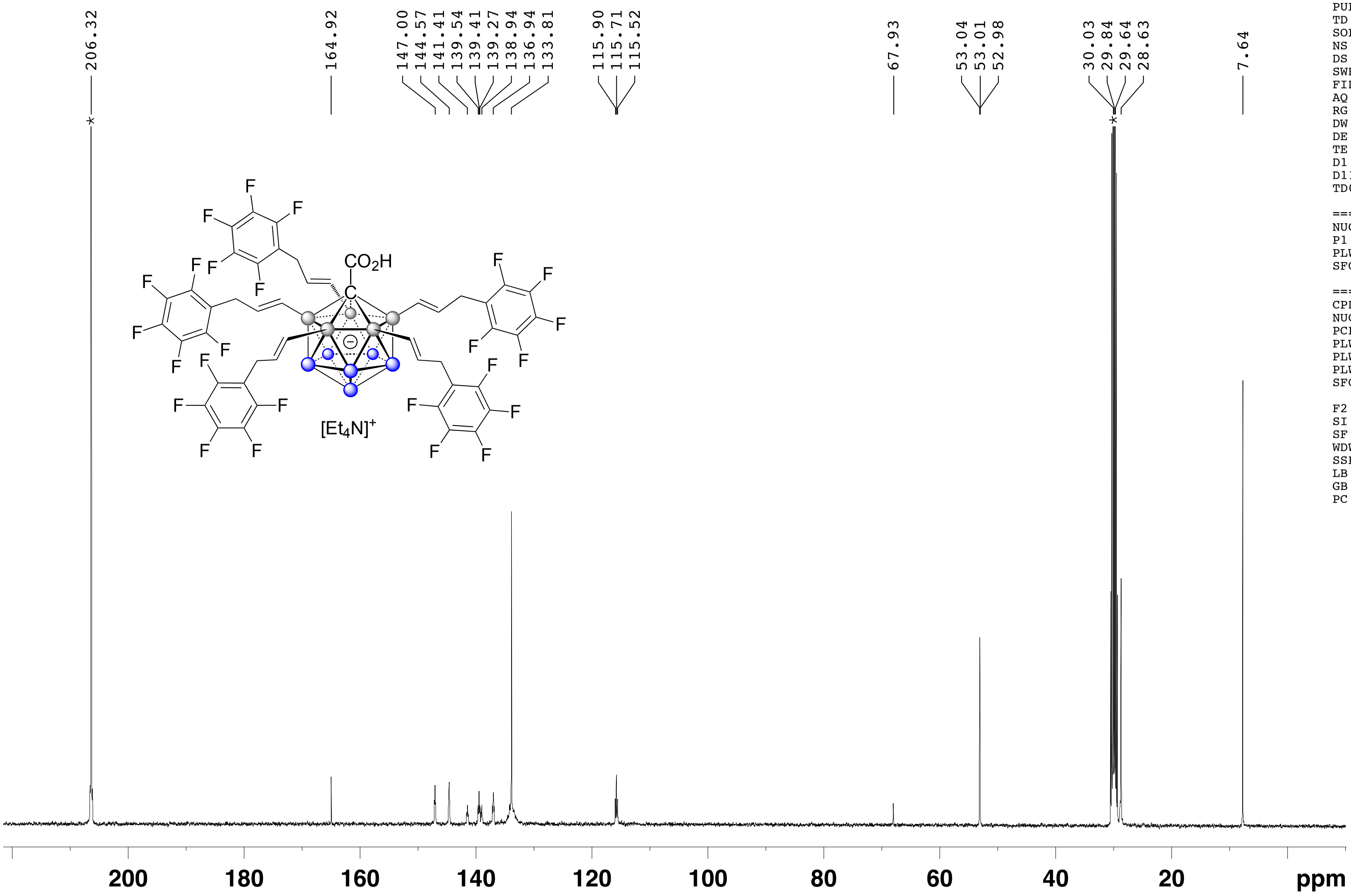


```
Current Data Parameters
NAME       penta-CH2C6F5
EXPNO                  4
PROCNO                 1

F2 - Acquisition Parameters
Date_           20180527
Time               23.15
INSTRUM            spect
PROBHD   5 mm PABBO BB/
PULPROG          zgpg30
TD                65536
SOLVENT          Acetone
NS                 2048
DS                    4
SWH          29761.904 Hz
FIDRES        0.454131 Hz
AQ           1.1010048 sec
RG              193.34
DW              16.800 usec
DE                6.50 usec
TE               294.5 K
D1          1.50000000 sec
D11         0.03000000 sec
TD0                   1

======== CHANNEL f1 ========
NUC1                13C
P1                10.00 usec
PLW1        53.00000000 W
SFO1        100.6228293 MHz

======== CHANNEL f2 ========
CPDPRG[2         waltz16
NUC2                 1H
PCPD2             80.00 usec
PLW2        12.50000000 W
PLW12        0.43945000 W
PLW13        0.28125000 W
SFO2        400.1316005 MHz

F2 - Processing parameters
SI                32768
SF          100.6126791 MHz
WDW                  EM
SSB      0
LB                 2.00 Hz
GB       0
PC                 1.40
```

Penta–hexene product 40 mg in 0.6 ml acetone–d6 *
1H{11B} NMR, 500 MHz, 23 C

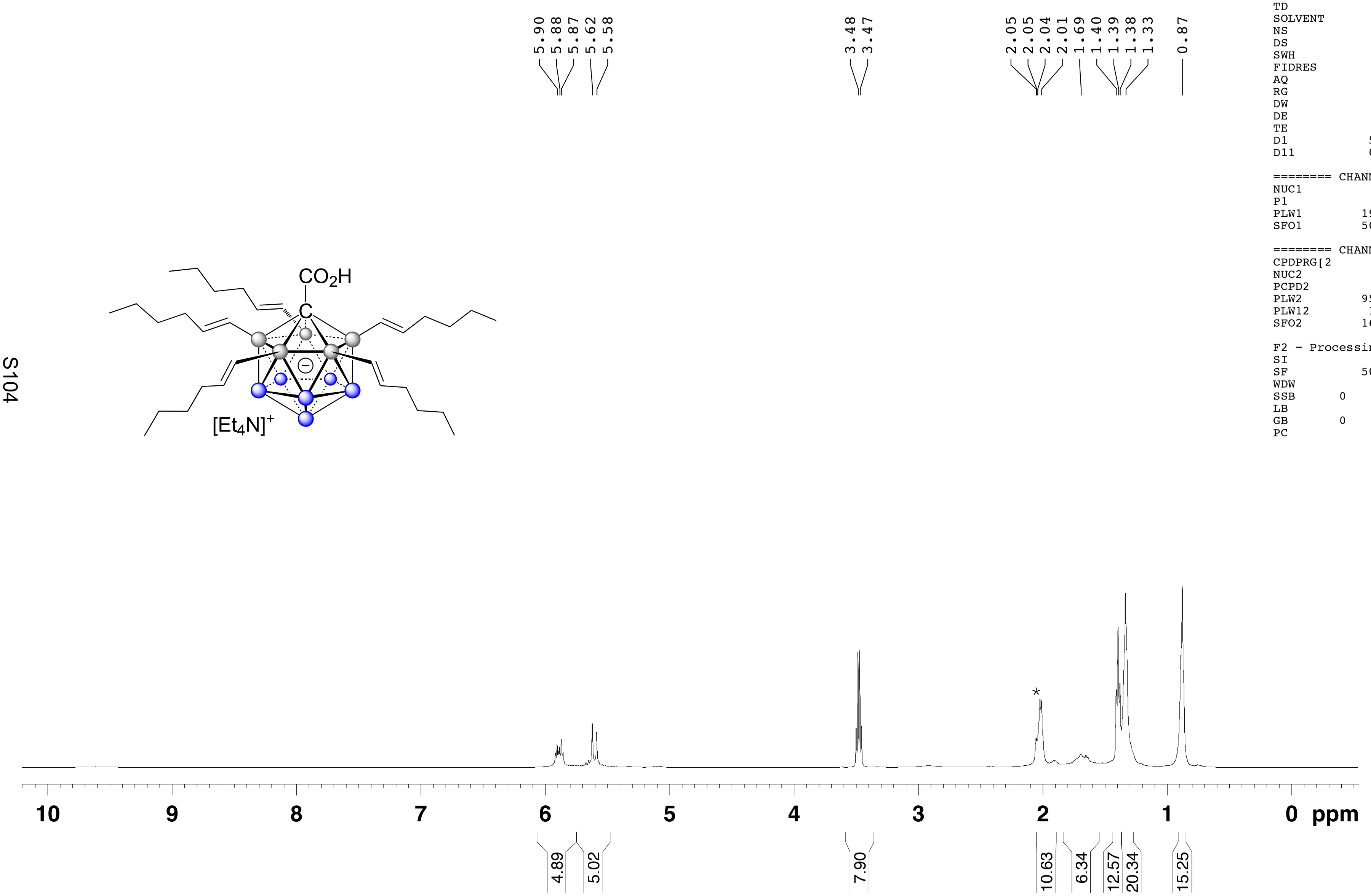


```
Current Data Parameters
NAME        penta-hexene
EXPNO                  1
PROCNO                 1

F2 - Acquisition Parameters
Date_           20170509
Time                0.55
INSTRUM            spect
PROBHD   5 mm PABBO BB-
PULPROG          zgig30
TD                65536
SOLVENT          Acetone
NS                   16
DS                    0
SWH           12500.000 Hz
FIDRES         0.190735 Hz
AQ            2.6214399 sec
RG                   18
DW               40.000 usec
DE                 6.50 usec
TE                295.9 K
D1           5.00000000 sec
D11          0.03000000 sec

======== CHANNEL f1 ========
NUC1                 1H
P1                11.70 usec
PLW1        19.00000000 W
SFO1        500.1335009 MHz

======== CHANNEL f2 ========
CPDPRG[2           garp
NUC2                11B
PCPD2            100.00 usec
PLW2        95.00000000 W
PLW12        1.63030005 W
SFO2        160.4615690 MHz

F2 - Processing parameters
SI                65536
SF          500.1300101 MHz
WDW                  EM
SSB      0
LB                 1.00 Hz
GB       0
PC                 1.00
```

Penta–hexene product 40 mg in 0.6 ml acetone–d6
11B NMR, 160 MHz, 23 C

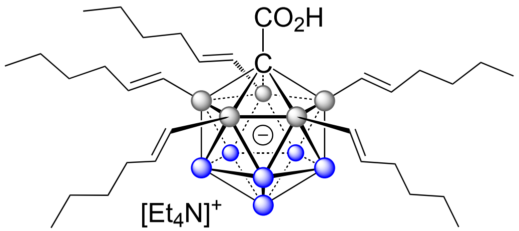

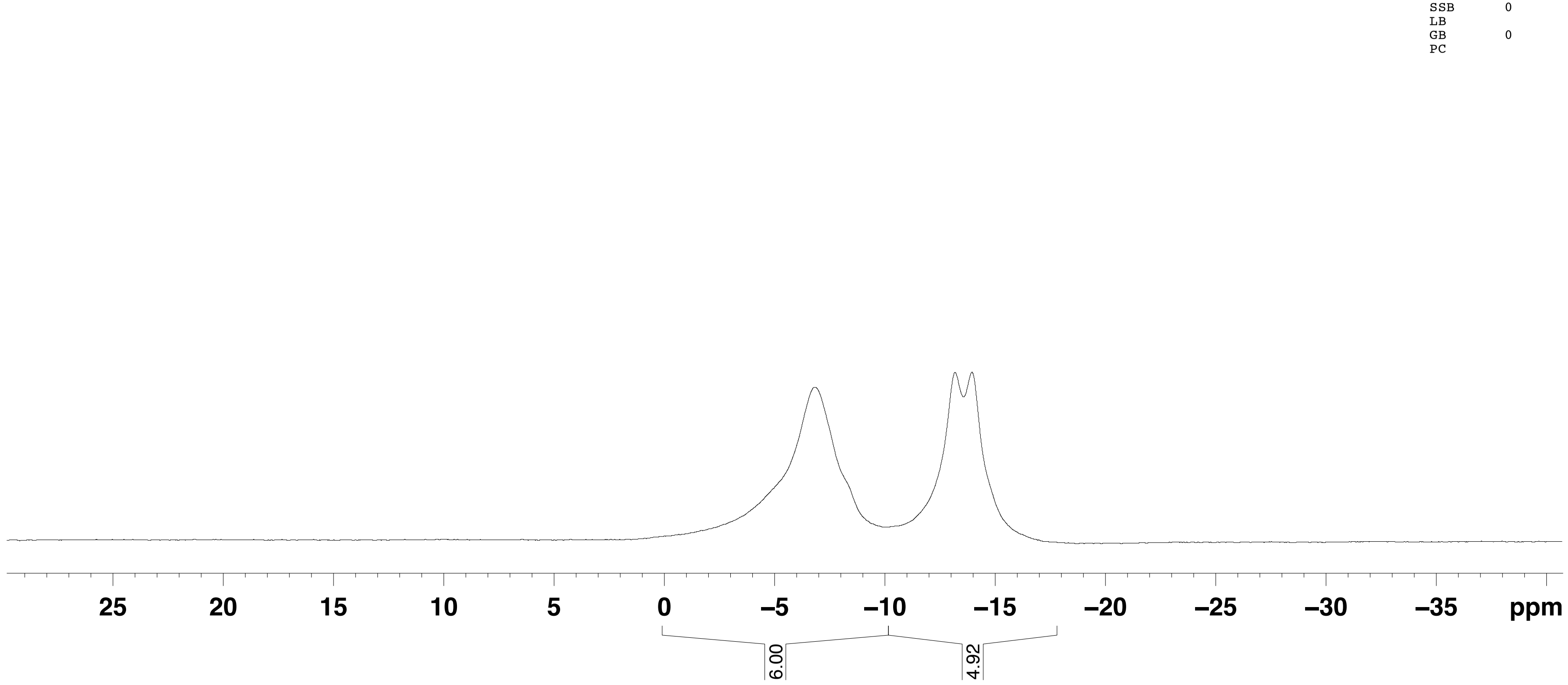


```
Current Data Parameters
NAME          penta-hexene
EXPNO                    2
PROCNO                   1

F2 - Acquisition Parameters
Date_             20170509
Time                  0.58
INSTRUM              spect
PROBHD   5 mm PABBO BB-
PULPROG               zg30
TD                   64098
SOLVENT            Acetone
NS                      64
DS                       0
SWH              32051.281 Hz
FIDRES            0.500036 Hz
AQ               0.9999288 sec
RG                     203
DW                  15.600 usec
DE                    6.50 usec
TE                   295.7 K
D1              1.00000000 sec

======== CHANNEL f1 ========
NUC1                   11B
P1                   13.10 usec
PLW1           95.00000000 W
SFO1           160.4615792 MHz

F2 - Processing parameters
SI                   32768
SF             160.4615790 MHz
WDW                     EM
SSB      0
LB                   10.00 Hz
GB       0
PC                    1.40
```

Penta–hexene product 40 mg in 0.6 ml acetone–d6
11B{1H} 160 MHz, 23 C

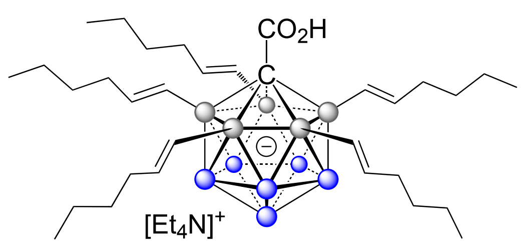

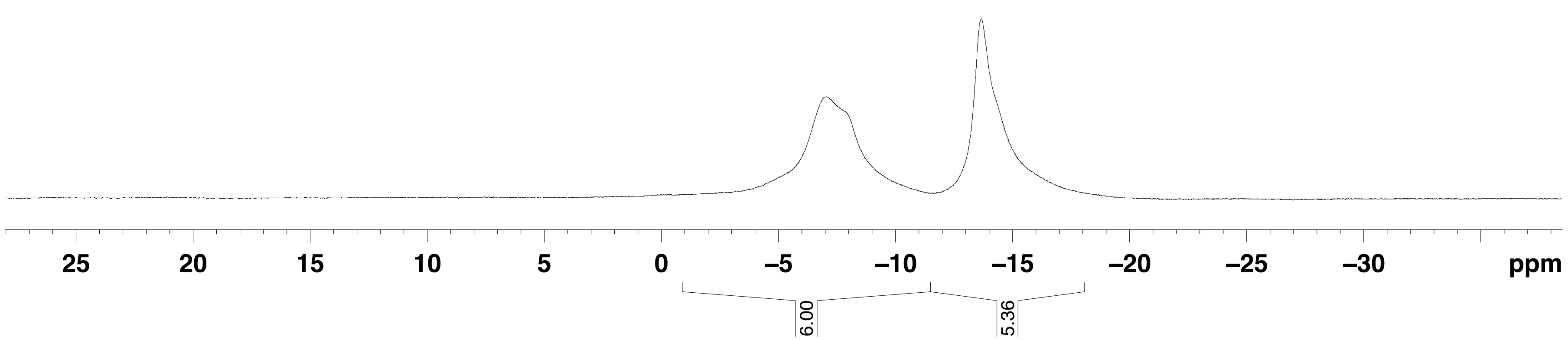


```
Current Data Parameters
NAME        penta-hexene
EXPNO                  3
PROCNO                 1

F2 - Acquisition Parameters
Date_           20170509
Time                1.01
INSTRUM            spect
PROBHD   5 mm PABBO BB-
PULPROG          zgpg30
TD                65536
SOLVENT          Acetone
NS                    64
DS                     0
SWH            32051.281 Hz
FIDRES          0.489064 Hz
AQ             1.0223616 sec
RG                   203
DW                15.600 usec
DE                  6.50 usec
TE                 296.2 K
D1            1.00000000 sec
D11           0.03000000 sec

======== CHANNEL f1 ========
NUC1                 11B
P1                 13.10 usec
PLW1         95.00000000 W
SFO1         160.4615790 MHz

======== CHANNEL f2 ========
CPDPRG[2         waltz16
NUC2                  1H
PCPD2              80.00 usec
PLW2         19.00000000 W
PLW12         0.40639001 W
PLW13         0.26008999 W
SFO2         500.1325007 MHz

F2 - Processing parameters
SI                 32768
SF           160.4615790 MHz
WDW                   EM
SSB      0
LB                  4.00 Hz
GB       0
PC                  1.40
```

Penta–hexene product 40 mg in 0.6 ml acetone–d6 *
13C{1H} NMR, 126 MHz, 23 C

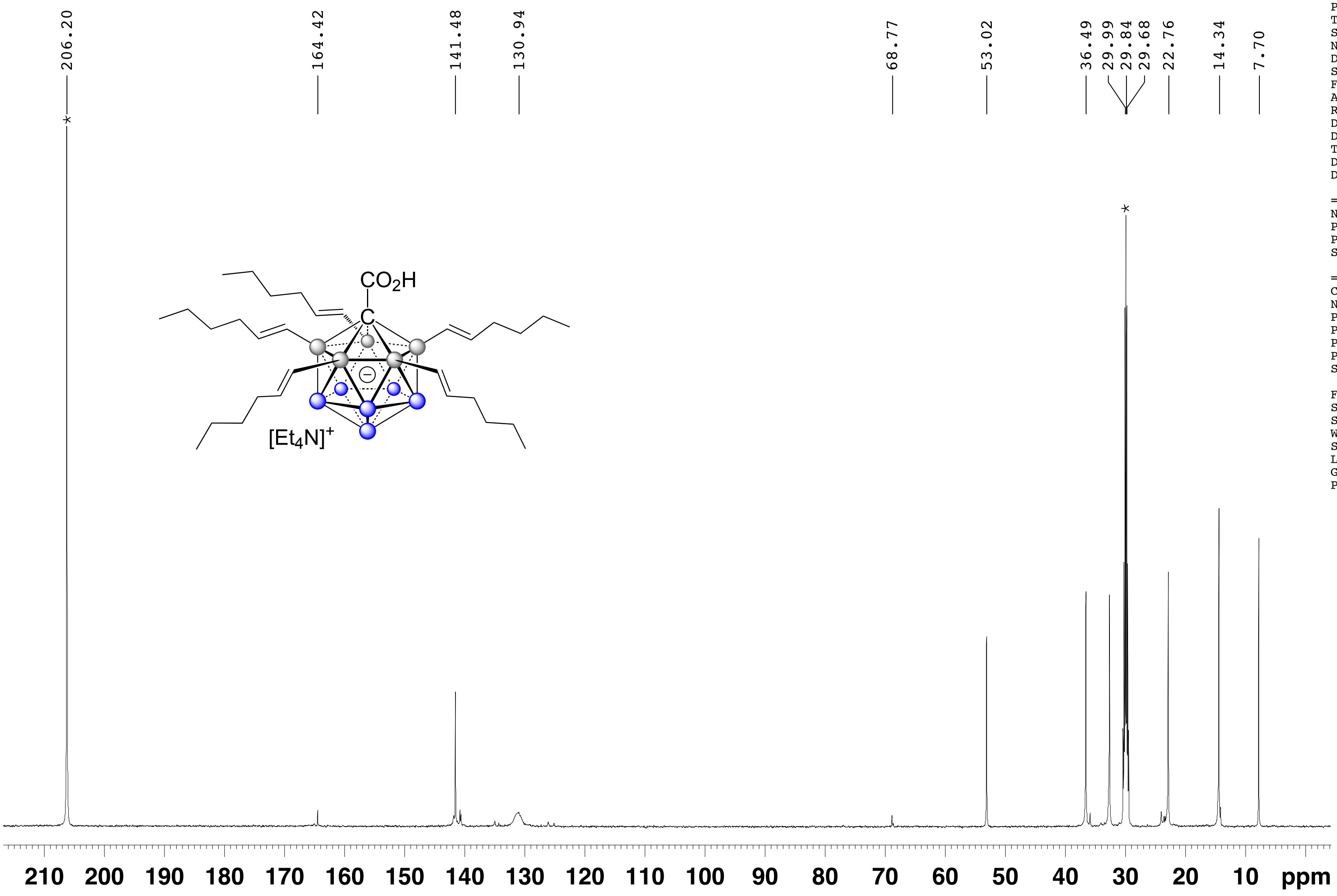


```
Current Data Parameters
NAME        penta-hexene
EXPNO                  4
PROCNO                 1

F2 - Acquisition Parameters
Date_           20170509
Time                2.25
INSTRUM            spect
PROBHD   5 mm PABBO BB-
PULPROG          zgpg30
TD                65536
SOLVENT          Acetone
NS                 2048
DS                    4
SWH           37878.789 Hz
FIDRES         0.577984 Hz
AQ            0.8650752 sec
RG                  203
DW               13.200 usec
DE                 6.50 usec
TE                296.4 K
D1           1.50000000 sec
D11          0.03000000 sec

======== CHANNEL f1 ========
NUC1                13C
P1                10.50 usec
PLW1        95.00000000 W
SFO1       125.7716224 MHz

======== CHANNEL f2 ========
CPDPRG[2          waltz16
NUC2                  1H
PCPD2              80.00 usec
PLW2        19.00000000 W
PLW12        0.40639001 W
PLW13        0.26008999 W
SFO2       500.1320005 MHz

F2 - Processing parameters
SI                32768
SF          125.7576857 MHz
WDW                  EM
SSB      0
LB                 5.00 Hz
GB       0
PC                 1.40
```

penta–4–methyl–1–pentene product 40 mg in 0.6 ml acetone–d6 *
1H{11B} NMR, 500 MHz, 23 C

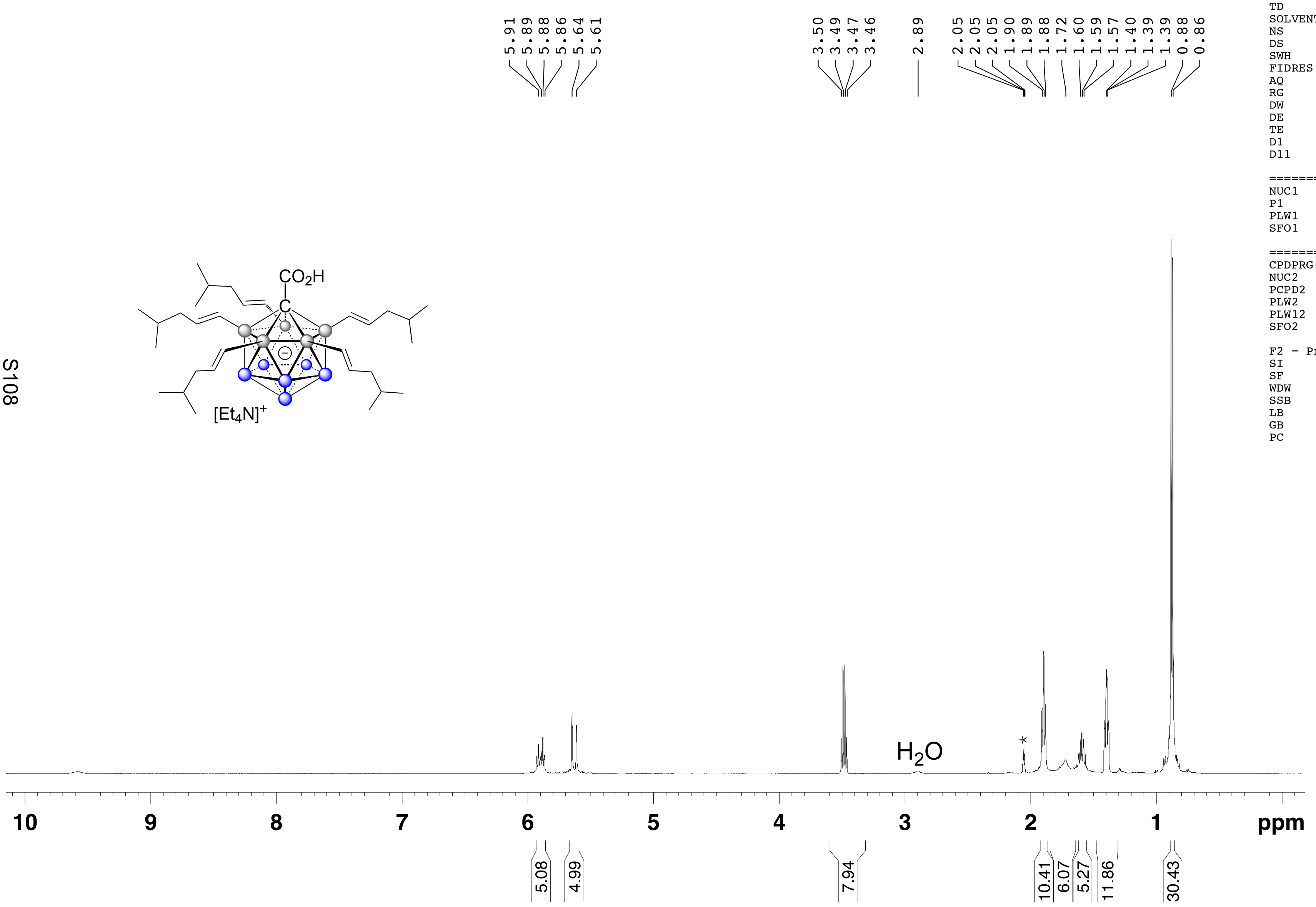


```
Current Data Parameters
NAME     penta-4-methyl-1-pentene
EXPNO                   1
PROCNO                  1

F2 - Acquisition Parameters
Date_            20170414
Time                16.04
INSTRUM             spect
PROBHD   5 mm PABBO BB-
PULPROG           zgig30
TD                 65536
SOLVENT          Acetone
NS                    16
DS                     0
SWH           12500.000 Hz
FIDRES         0.190735 Hz
AQ            2.6214399 sec
RG                    36
DW               40.000 usec
DE                 6.50 usec
TE                296.0 K
D1           5.00000000 sec
D11          0.03000000 sec

======== CHANNEL f1 ========
NUC1                  1H
P1                 11.50 usec
PLW1         19.00000000 W
SFO1        500.1335009 MHz

======== CHANNEL f2 ========
CPDPRG[2            garp
NUC2                 11B
PCPD2             100.00 usec
PLW2         95.00000000 W
PLW12         1.63030005 W
SFO2        160.4615690 MHz

F2 - Processing parameters
SI                 65536
SF          500.1300103 MHz
WDW                   no
SSB      0
LB       0 Hz
GB       0
PC                  1.00
```

penta–4–methyl–1–pentene product 40 mg in 0.6 ml acetone–d6
11B NMR, 500 MHz, 23 C

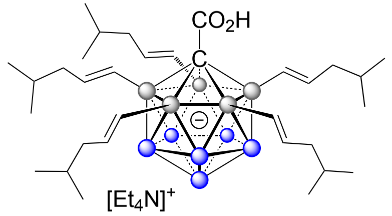

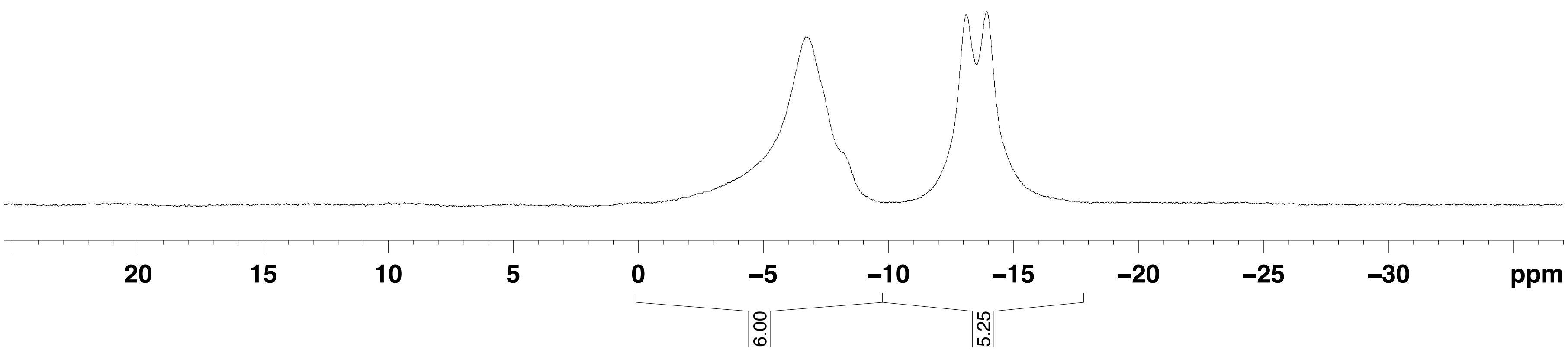


```
Current Data Parameters
NAME     penta-4-methyl-1-pentene
EXPNO                   2
PROCNO                  1

F2 - Acquisition Parameters
Date_          20170414
Time              16.13
INSTRUM           spect
PROBHD   5 mm PABBO BB-
PULPROG            zg30
TD                65536
SOLVENT         Acetone
NS                   32
DS                    2
SWH           32051.281 Hz
FIDRES         0.489064 Hz
AQ            1.0223616 sec
RG                  203
DW               15.600 usec
DE                 6.50 usec
TE                295.5 K
D1           2.00000000 sec

======== CHANNEL f1 ========
NUC1                11B
P1                13.10 usec
PLW1        95.00000000 W
SFO1        160.4615792 MHz

F2 - Processing parameters
SI                32768
SF          160.4615790 MHz
WDW                  EM
SSB      0
LB                 5.00 Hz
GB       0
PC                 1.40
```

penta–4–methyl–1–pentene product 40 mg in 0.6 ml acetone–d6
11B{1H} NMR, 160 MHz, 23 C

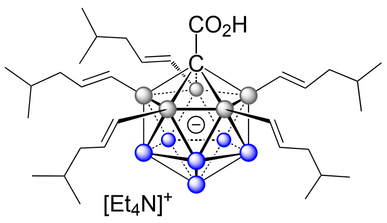

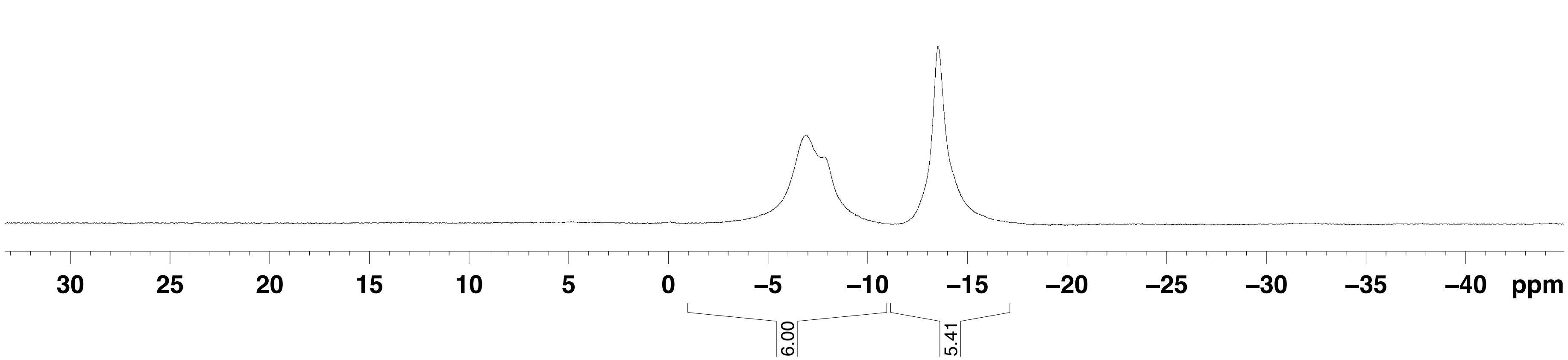


```
Current Data Parameters
NAME     penta-4-methyl-1-pentene
EXPNO                       3
PROCNO                      1

F2 - Acquisition Parameters
Date_          20170414
Time              16.14
INSTRUM           spect
PROBHD   5 mm PABBO BB-
PULPROG          zgpg30
TD                65536
SOLVENT         Acetone
NS                   32
DS                    0
SWH           32051.281 Hz
FIDRES         0.489064 Hz
AQ            1.0223616 sec
RG                  203
DW               15.600 usec
DE                 6.50 usec
TE                295.7 K
D1           1.00000000 sec
D11          0.03000000 sec

======== CHANNEL f1 ========
NUC1                 11B
P1                 13.10 usec
PLW1         95.00000000 W
SFO1         160.4615790 MHz

======== CHANNEL f2 ========
CPDPRG[2         waltz16
NUC2                  1H
PCPD2              80.00 usec
PLW2         19.00000000 W
PLW12         0.39262000 W
PLW13         0.25127000 W
SFO2         500.1325007 MHz

F2 - Processing parameters
SI                 32768
SF           160.4615790 MHz
WDW                   EM
SSB      0
LB                  5.00 Hz
GB       0
PC                  1.40
```

penta–4–methyl–1–pentene product 40 mg in 0.6 ml acetone–d6*
13C{1H} NMR, 101 MHz, 23 C

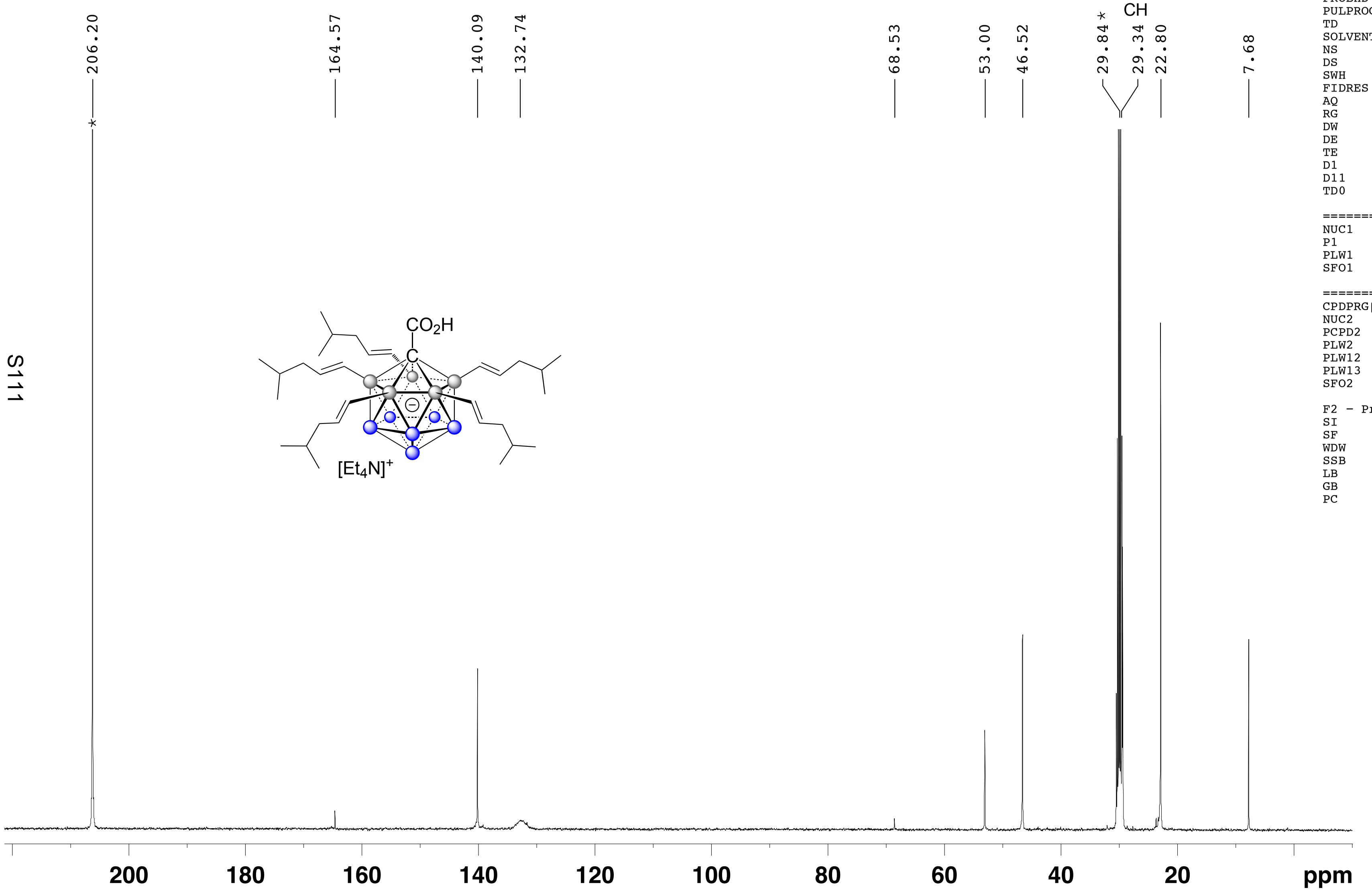


```
Current Data Parameters
NAME      penta-4-methyl-1-pentene-1
EXPNO                1
PROCNO               1

F2 - Acquisition Parameters
Date_          20170415
Time              17.42
INSTRUM           spect
PROBHD   5 mm PABBO BB/
PULPROG         zgpg30
TD               65536
SOLVENT         Acetone
NS                2048
DS                   4
SWH          29761.904 Hz
FIDRES        0.454131 Hz
AQ           1.1010048 sec
RG              193.34
DW              16.800 usec
DE                6.50 usec
TE               295.4 K
D1          1.50000000 sec
D11         0.03000000 sec
TD0                  1

======== CHANNEL f1 ========
NUC1               13C
P1               10.00 usec
PLW1       53.00000000 W
SFO1       100.6228293 MHz

======== CHANNEL f2 ========
CPDPRG[2        waltz16
NUC2                1H
PCPD2            80.00 usec
PLW2       12.50000000 W
PLW12       0.43945000 W
PLW13       0.28125000 W
SFO2       400.1316005 MHz

F2 - Processing parameters
SI               32768
SF         100.6126854 MHz
WDW                 EM
SSB      0
LB                3.00 Hz
GB       0
PC                1.40
```

penta–4–methyl–1–hexene product 40 mg in 0.6 ml acetone–d6 *
1H{11B} NMR, 500 MHz, 23 C

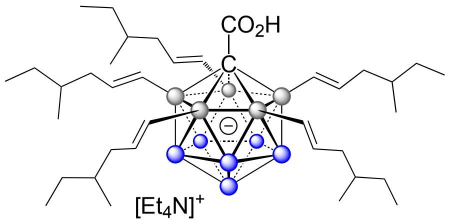

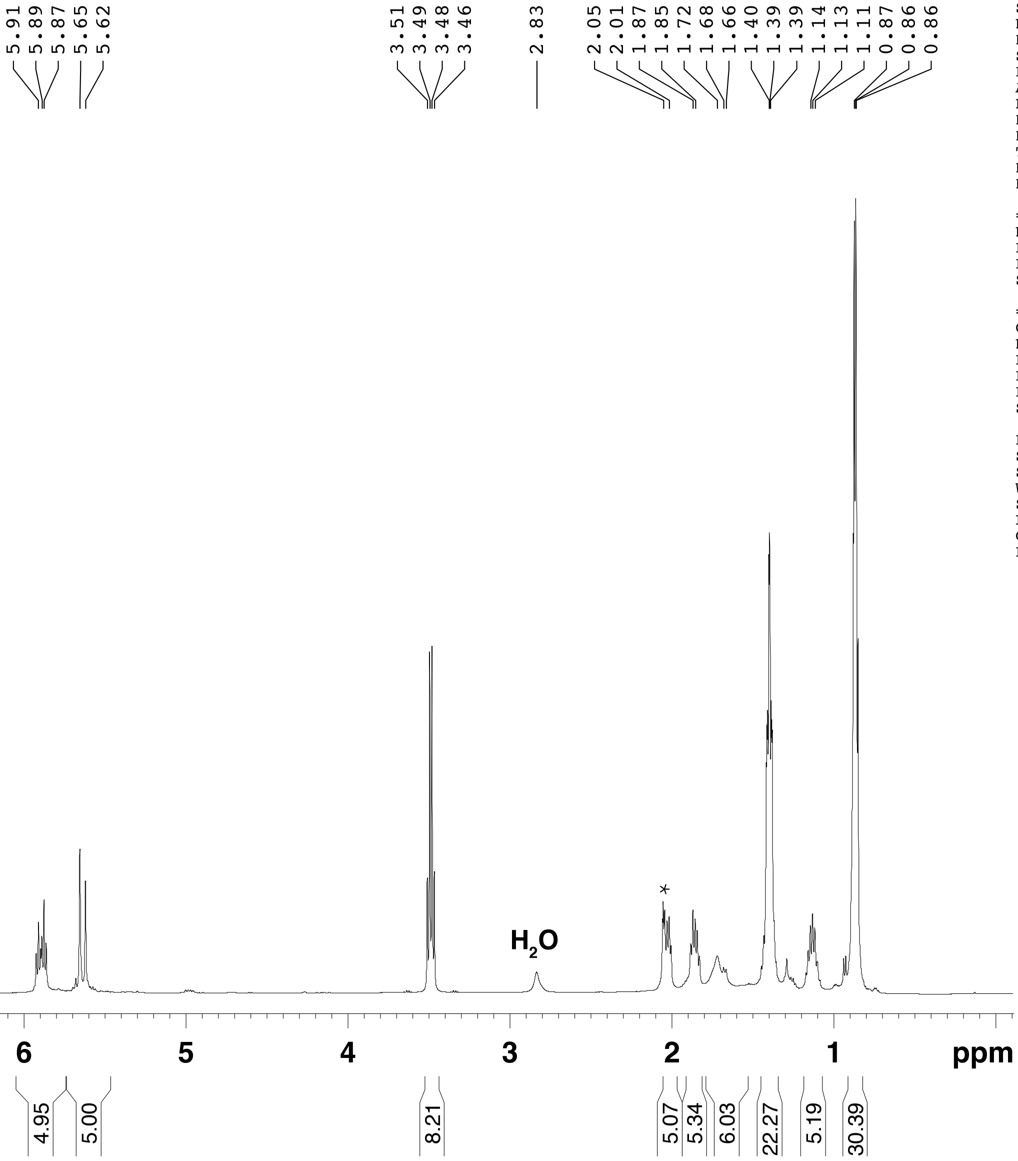


```
Current Data Parameters
NAME     penta-4-methyl-1-hexene
EXPNO                 1
PROCNO                1

F2 - Acquisition Parameters
Date_          20180403
Time               6.21
INSTRUM           spect
PROBHD   5 mm PABBO BB-
PULPROG          zgig30
TD                65536
SOLVENT            DMSO
NS                   16
DS                    0
SWH           12500.000 Hz
FIDRES         0.190735 Hz
AQ            2.6214399 sec
RG                 22.6
DW               40.000 usec
DE                 6.50 usec
TE                296.4 K
D1           5.00000000 sec
D11          0.03000000 sec

======== CHANNEL f1 ========
NUC1                 1H
P1                11.70 usec
PLW1        19.00000000 W
SFO1        500.1335009 MHz

======== CHANNEL f2 ========
CPDPRG[2           garp
NUC2                11B
PCPD2            100.00 usec
PLW2        95.00000000 W
PLW12        1.63030005 W
SFO2        160.4615690 MHz

F2 - Processing parameters
SI                65536
SF          500.1302351 MHz
WDW                  EM
SSB      0
LB                 1.00 Hz
GB       0
PC                 1.00
```

penta–4–methyl–1–hexene product 40 mg in 0.6 ml acetone–d6
11B NMR, 500 MHz, 23 C

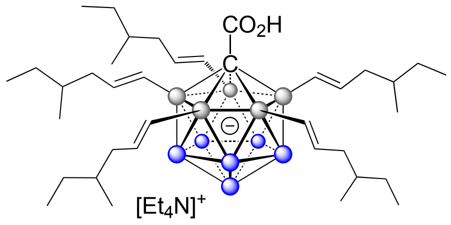

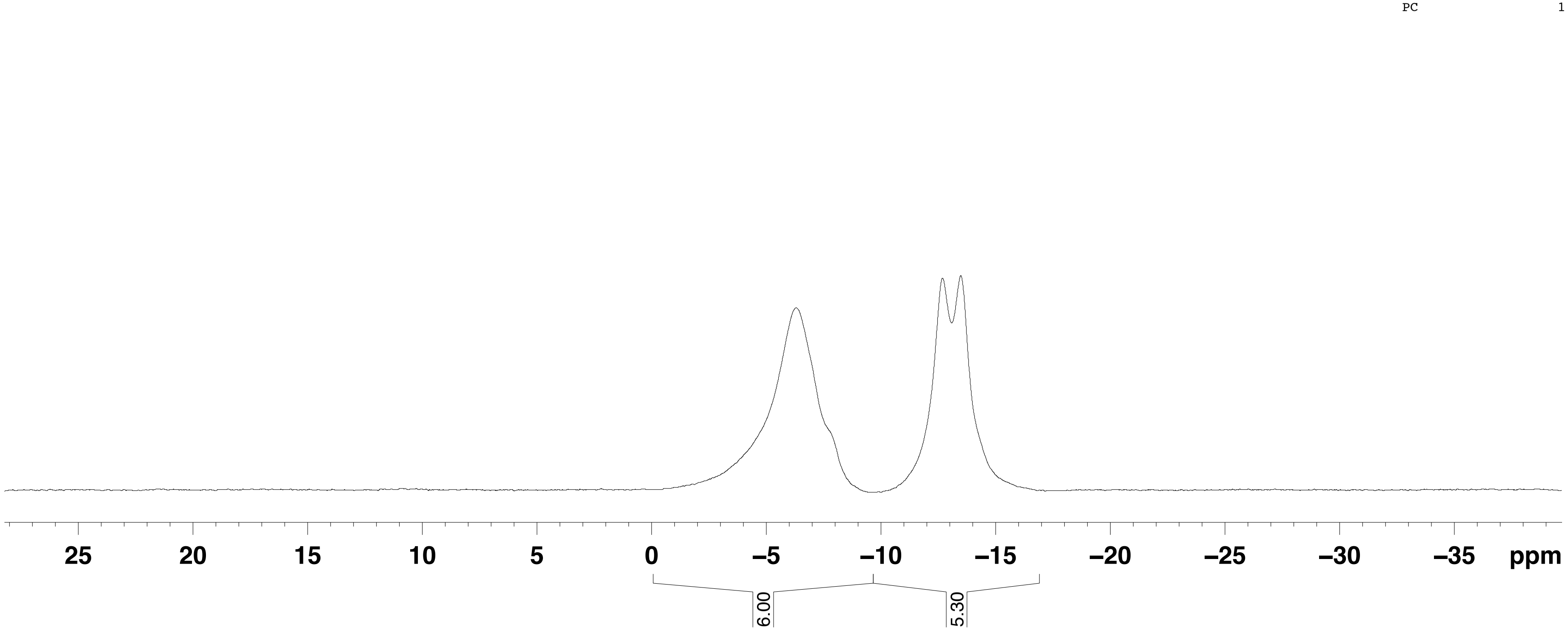


```
Current Data Parameters
NAME     penta-4-methyl-1-hexene
EXPNO                 2
PROCNO                1

F2 - Acquisition Parameters
Date_          20180403
Time               6.24
INSTRUM           spect
PROBHD   5 mm PABBO BB-
PULPROG            zg30
TD                64098
SOLVENT            DMSO
NS                   64
DS                    0
SWH           32051.281 Hz
FIDRES         0.500036 Hz
AQ            0.9999288 sec
RG                  203
DW               15.600 usec
DE                 6.50 usec
TE                296.0 K
D1           1.00000000 sec

======== CHANNEL f1 ========
NUC1                11B
P1                13.10 usec
PLW1        95.00000000 W
SFO1        160.4615792 MHz

F2 - Processing parameters
SI                32768
SF          160.4615790 MHz
WDW                  EM
SSB      0
LB                10.00 Hz
GB       0
PC                 1.40
```

penta-4-methyl-1-hexene product 40 mg in 0.6 ml acetone-d6
11B{1H} NMR, 160 MHz, 23 C

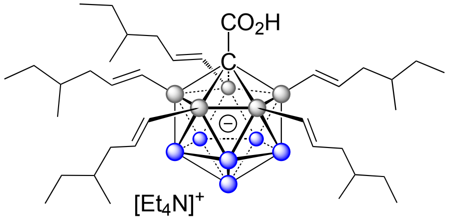

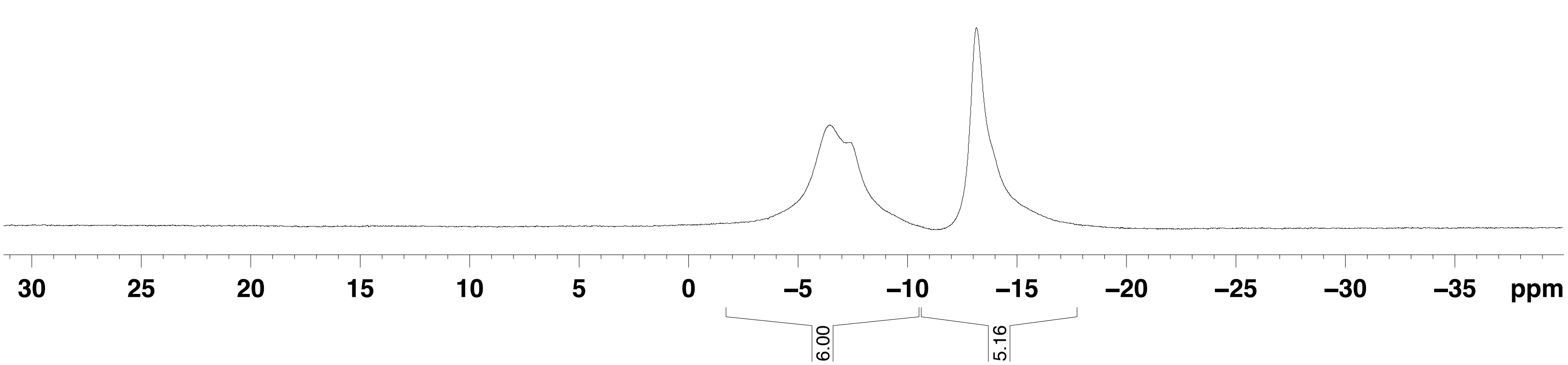


```
Current Data Parameters
NAME     penta-4-methyl-1-hexene
EXPNO                  3
PROCNO                 1

F2 - Acquisition Parameters
Date_          20180403
Time               6.27
INSTRUM           spect
PROBHD   5 mm PABBO BB-
PULPROG         zgpg30
TD               65536
SOLVENT           DMSO
NS                  64
DS                   0
SWH          32051.281 Hz
FIDRES        0.489064 Hz
AQ           1.0223616 sec
RG                 203
DW              15.600 usec
DE                6.50 usec
TE               296.1 K
D1          1.00000000 sec
D11         0.03000000 sec

======== CHANNEL f1 ========
NUC1               11B
P1               13.10 usec
PLW1       95.00000000 W
SFO1       160.4615790 MHz

======== CHANNEL f2 ========
CPDPRG[2       waltz16
NUC2                1H
PCPD2            80.00 usec
PLW2       19.00000000 W
PLW12       0.40639001 W
PLW13       0.26008999 W
SFO2       500.1325007 MHz

F2 - Processing parameters
SI               32768
SF         160.4615790 MHz
WDW                 EM
SSB      0
LB                5.00 Hz
GB       0
PC                1.40
```

penta–4–methyl–1–hexene product 40 mg in 0.6 ml acetone–d6*
13C{1H} NMR, 126 MHz, 23 C

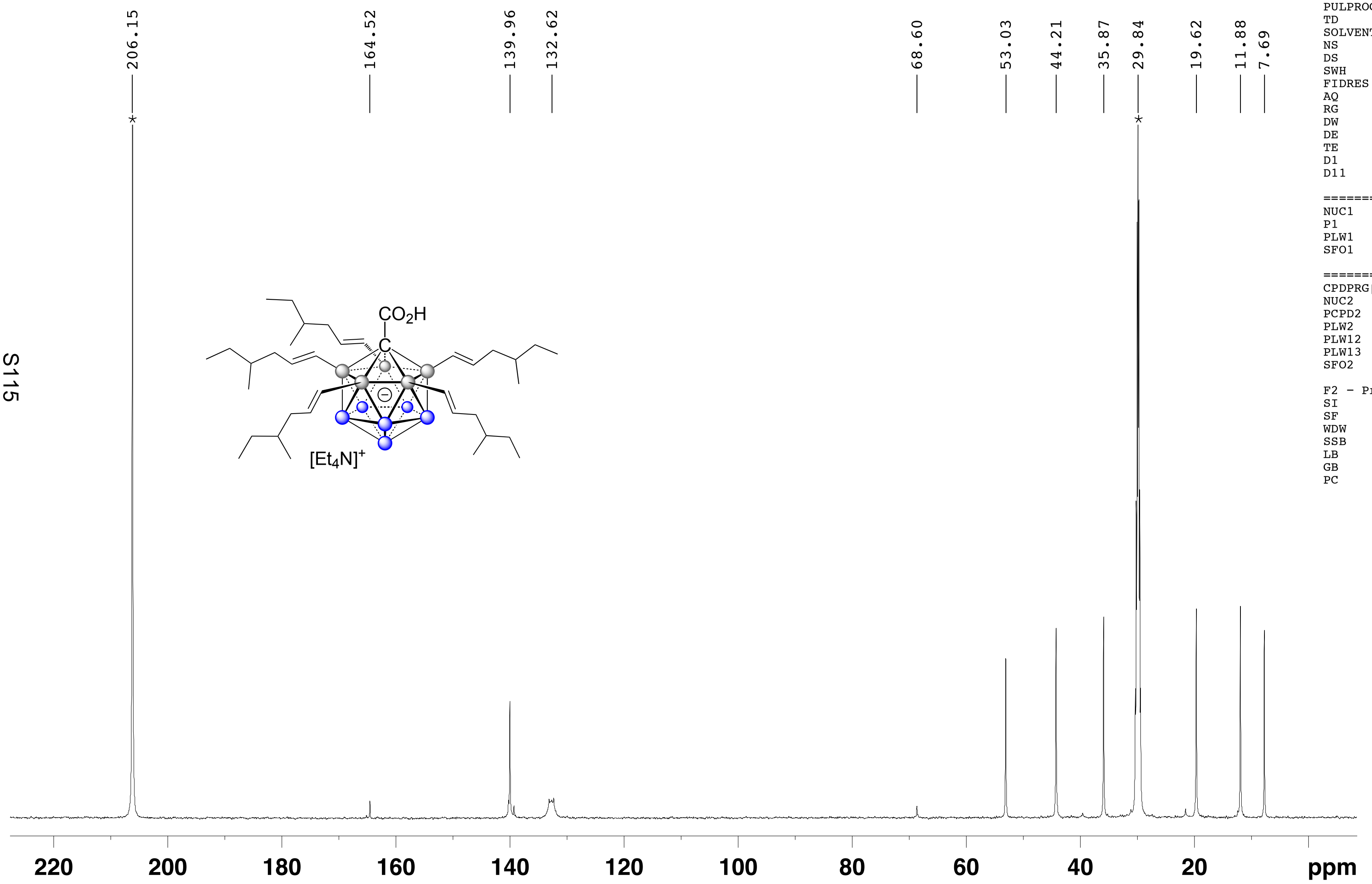


```
Current Data Parameters
NAME      penta-4-methyl-1-hexene
EXPNO                   4
PROCNO                  1

F2 - Acquisition Parameters
Date_            20180403
Time                 7.54
INSTRUM             spect
PROBHD   5 mm PABBO BB-
PULPROG           zgpg30
TD                 65536
SOLVENT             DMSO
NS                  2100
DS                     4
SWH           37878.789 Hz
FIDRES         0.577984 Hz
AQ            0.8650752 sec
RG                   203
DW               13.200 usec
DE                 6.50 usec
TE                296.6 K
D1           1.50000000 sec
D11          0.03000000 sec

======== CHANNEL f1 ========
NUC1                 13C
P1                 10.50 usec
PLW1         95.00000000 W
SFO1         125.7716224 MHz

======== CHANNEL f2 ========
CPDPRG[2          waltz16
NUC2                  1H
PCPD2              80.00 usec
PLW2         19.00000000 W
PLW12         0.40639001 W
PLW13         0.26008999 W
SFO2         500.1320005 MHz

F2 - Processing parameters
SI                 32768
SF           125.7577380 MHz
WDW                   EM
SSB      0
LB                 10.00 Hz
GB       0
PC                  1.40
```

penta–4,4–dimethyl–1–pentene product 50 mg in 0.6 ml acetone–d6*
1H{11B} NMR, 400 MHz, 23 C

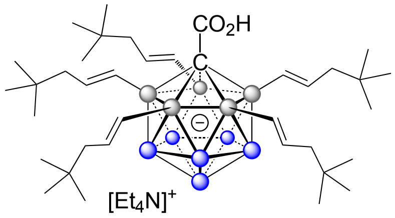

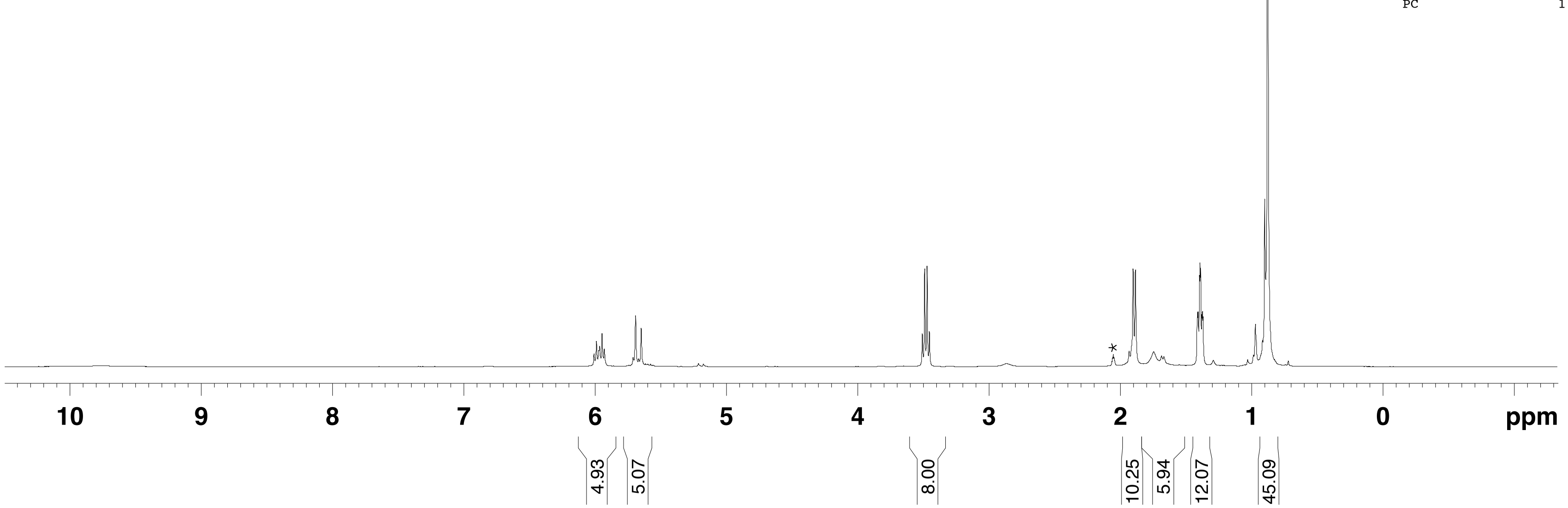


```
Current Data Parameters
NAME     penta-44-dimethyl-1-penter
EXPNO                 1
PROCNO                1

F2 - Acquisition Parameters
Date_          20180506
Time               7.42
INSTRUM           spect
PROBHD   5 mm PABBO BB/
PULPROG          zgig30
TD                16384
SOLVENT         Acetone
NS                   16
DS                    4
SWH            8012.820 Hz
FIDRES         0.489064 Hz
AQ            1.0223616 sec
RG                36.87
DW               62.400 usec
DE                 6.50 usec
TE                294.8 K
D1           1.00000000 sec
D11          0.03000000 sec
TD0                   1

======== CHANNEL f1 ========
NUC1                 1H
P1                15.00 usec
PLW1        12.50000000 W
SFO1        400.1320007 MHz

======== CHANNEL f2 ========
CPDPRG[2          garp4
NUC2                11B
PCPD2             90.00 usec
PLW2        52.96599960 W
PLW12        0.64477998 W
SFO2        128.3776050 MHz

F2 - Processing parameters
SI                32768
SF          400.1300074 MHz
WDW                  EM
SSB      0
LB                 1.00 Hz
GB       0
PC                 1.40
```

penta–44–dimethyl–1–pentene product 50 mg in 0.6 ml acetone–d6
11B NMR, 128 MHz, 23 C

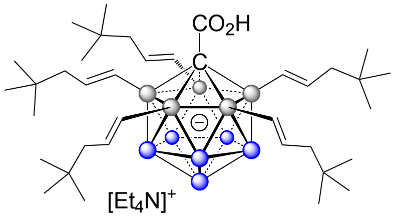

−6.69
−8.19
−12.98
−13.98

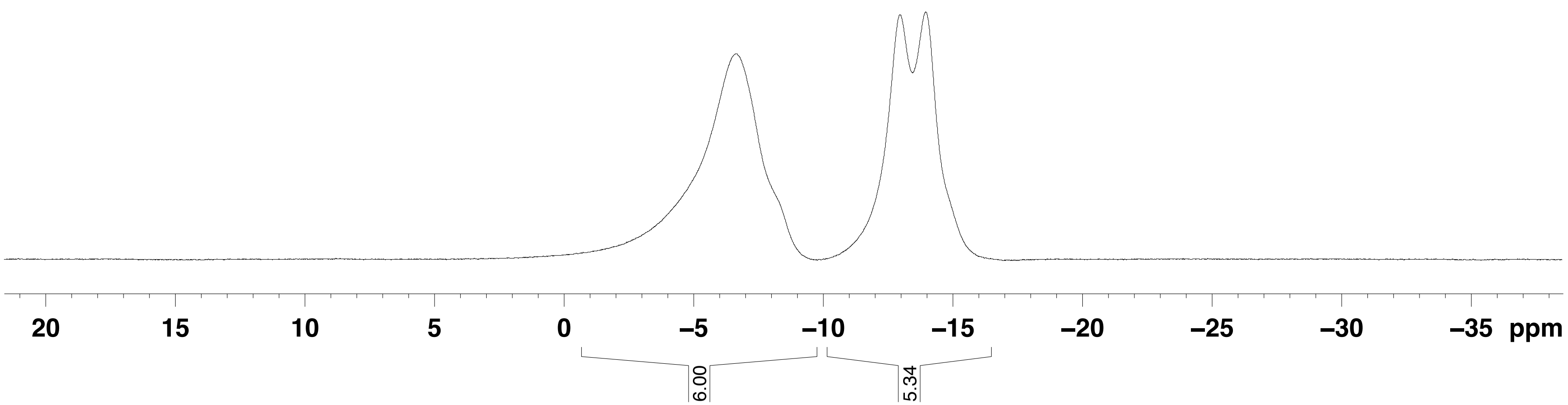


```
Current Data Parameters
NAME     penta-44-dimethyl-1-penter
EXPNO                     2
PROCNO                    1

F2 - Acquisition Parameters
Date_             20180506
Time                  7.48
INSTRUM              spect
PROBHD   5 mm PABBO BB/
PULPROG                 zg
TD                   65536
SOLVENT            Acetone
NS                     128
DS                       4
SWH              25510.203 Hz
FIDRES            0.389255 Hz
AQ               1.2845056 sec
RG                  193.34
DW                  19.600 usec
DE                    6.50 usec
TE                   294.7 K
D1              1.00000000 sec
TD0                      1

======== CHANNEL f1 ========
NUC1                   11B
P1                    9.93 usec
PLW1           52.96599960 W
SFO1           128.3776052 MHz

F2 - Processing parameters
SI                   32768
SF             128.3776050 MHz
WDW                     EM
SSB      0
LB                    2.00 Hz
GB       0
PC                    1.40
```

penta-4,4-dimethyl-1-pentene product 50 mg in 0.6 ml acetone-d6
11B{1H} NMR, 128 MHz, 23 C

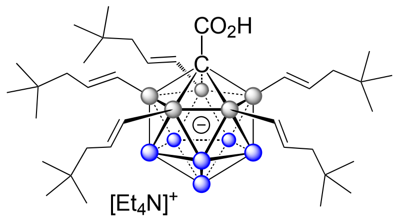

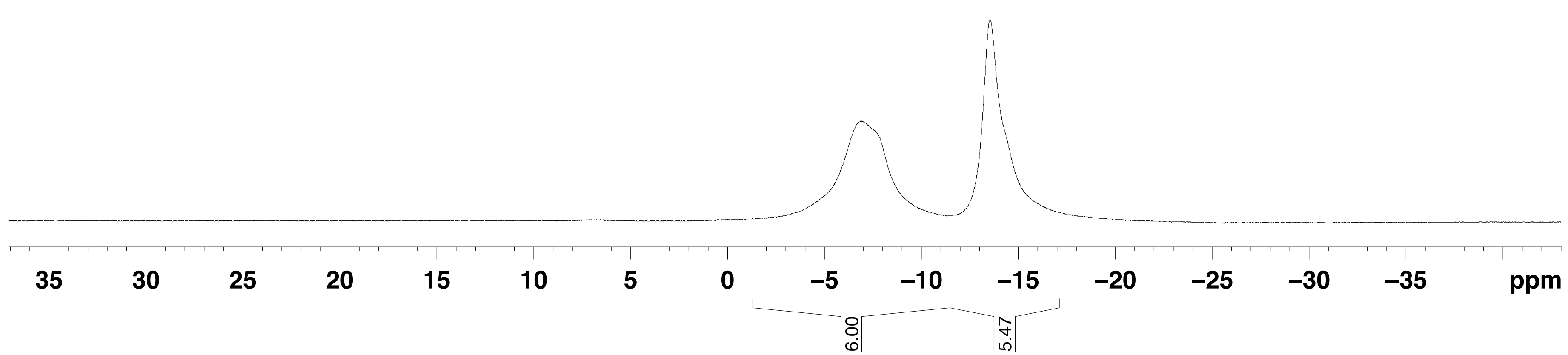


```
Current Data Parameters
NAME      penta-44-dimethyl-1-penter
EXPNO                    3
PROCNO                   1

F2 - Acquisition Parameters
Date_             20180506
Time                  7.54
INSTRUM              spect
PROBHD   5 mm PABBO BB/
PULPROG            zgpg30
TD                  65536
SOLVENT            Acetone
NS                     128
DS                       4
SWH              25510.203 Hz
FIDRES            0.389255 Hz
AQ               1.2845056 sec
RG                  193.34
DW                  19.600 usec
DE                    6.50 usec
TE                   295.7 K
D1              1.00000000 sec
D11             0.03000000 sec
TD0                      1

======== CHANNEL f1 ========
NUC1                   11B
P1                    9.93 usec
PLW1           52.96599960 W
SFO1          128.3776050 MHz

======== CHANNEL f2 ========
CPDPRG[2           waltz16
NUC2                    1H
PCPD2                80.00 usec
PLW2           12.50000000 W
PLW12           0.43945000 W
PLW13           0.28125000 W
SFO2          400.1320007 MHz

F2 - Processing parameters
SI                   32768
SF            128.3776050 MHz
WDW                     EM
SSB      0
LB                    3.00 Hz
GB       0
PC                    1.40
```

penta–4,4–dimethyl–1–pentene product 50 mg in 0.6 ml acetone–d6*
13C{1H} NMR, 126 MHz, 23 C

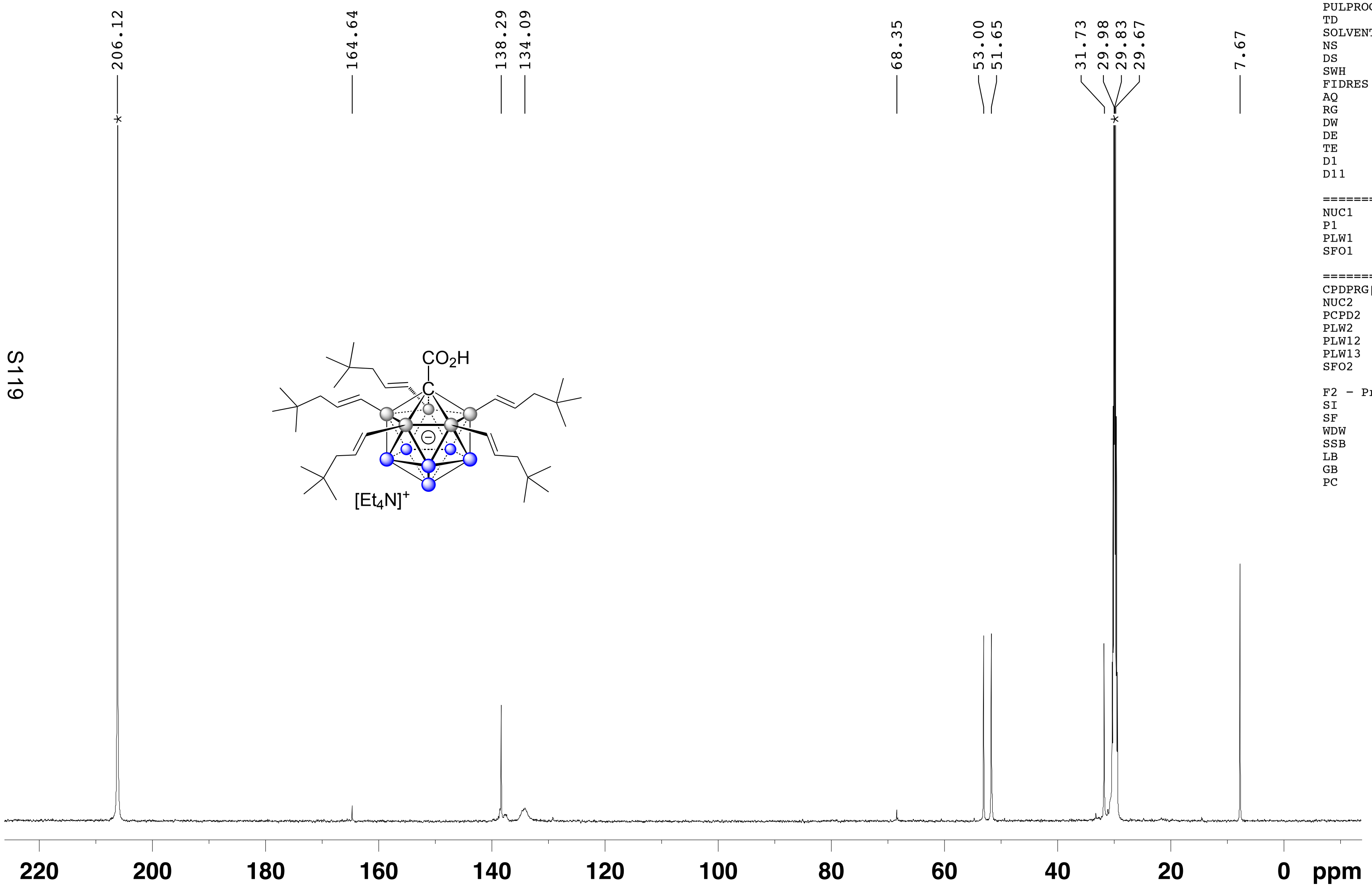


```
Current Data Parameters
NAME     penta-44-dimethyl-1-penter
EXPNO                 4
PROCNO                1

F2 - Acquisition Parameters
Date_          20180413
Time_              5.31
INSTRUM           spect
PROBHD   5 mm PABBO BB-
PULPROG         zgpg30
TD               65536
SOLVENT         Acetone
NS                2048
DS                   4
SWH          37878.789 Hz
FIDRES        0.577984 Hz
AQ           0.8650752 sec
RG                 203
DW              13.200 usec
DE                6.50 usec
TE               296.9 K
D1          1.50000000 sec
D11         0.03000000 sec

======== CHANNEL f1 ========
NUC1                13C
P1                10.50 usec
PLW1        95.00000000 W
SFO1        125.7716224 MHz

======== CHANNEL f2 ========
CPDPRG[2         waltz16
NUC2                 1H
PCPD2             80.00 usec
PLW2        19.00000000 W
PLW12        0.40639001 W
PLW13        0.26008999 W
SFO2        500.1320005 MHz

F2 - Processing parameters
SI                32768
SF          125.7576846 MHz
WDW                  EM
SSB      0
LB                 6.00 Hz
GB       0
PC                 1.40
```

Penta–CH2CH2–Ph product 50 mg in 0.6 ml acetone–d6*
1H{11B} NMR, 400 MHz, 23 C

```
Current Data Parameters
NAME      penta-CH2CH2Ph
EXPNO                  1
PROCNO                 1

F2 - Acquisition Parameters
Date_           20180503
Time               16.51
INSTRUM            spect
PROBHD   5 mm PABBO BB/
PULPROG           zgig30
TD                 16384
SOLVENT          Acetone
NS                    16
DS                     4
SWH             8012.820 Hz
FIDRES          0.489064 Hz
AQ             1.0223616 sec
RG                 20.46
DW                62.400 usec
DE                  6.50 usec
TE                 294.7 K
D1            1.00000000 sec
D11           0.03000000 sec
TD0                    1

======== CHANNEL f1 ========
NUC1                  1H
P1                 15.00 usec
PLW1         12.50000000 W
SFO1         400.1320007 MHz

======== CHANNEL f2 ========
CPDPRG[2           garp4
NUC2                 11B
PCPD2              90.00 usec
PLW2         52.96599960 W
PLW12         0.64477998 W
SFO2         128.3776050 MHz

F2 - Processing parameters
SI                 32768
SF           400.1300073 MHz
WDW                   EM
SSB      0
LB                  1.00 Hz
GB       0
PC                  1.40
```

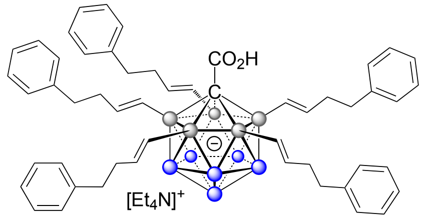

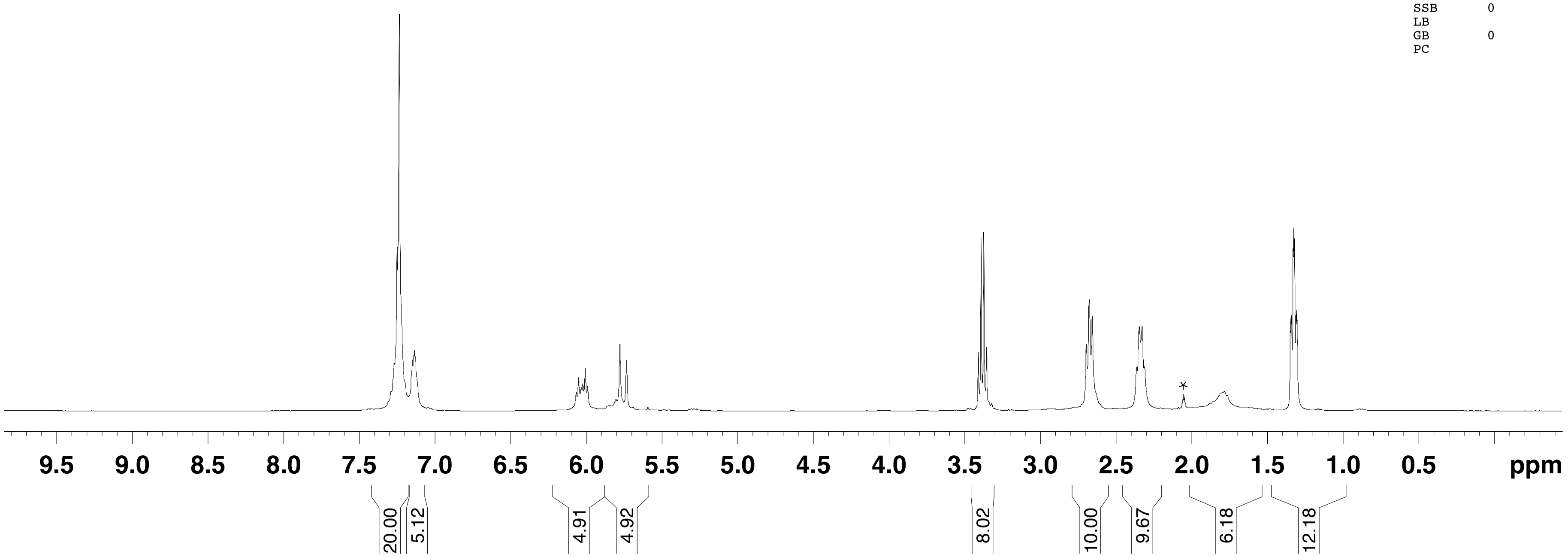

Penta–CH2CH2–Ph product 50 mg in 0.6 ml acetone–d6
11B NMR, 128 MHz, 23 C

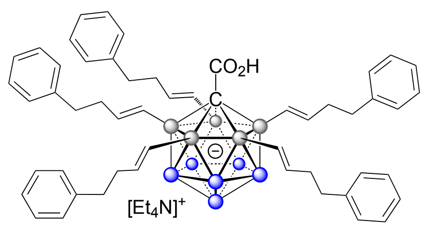

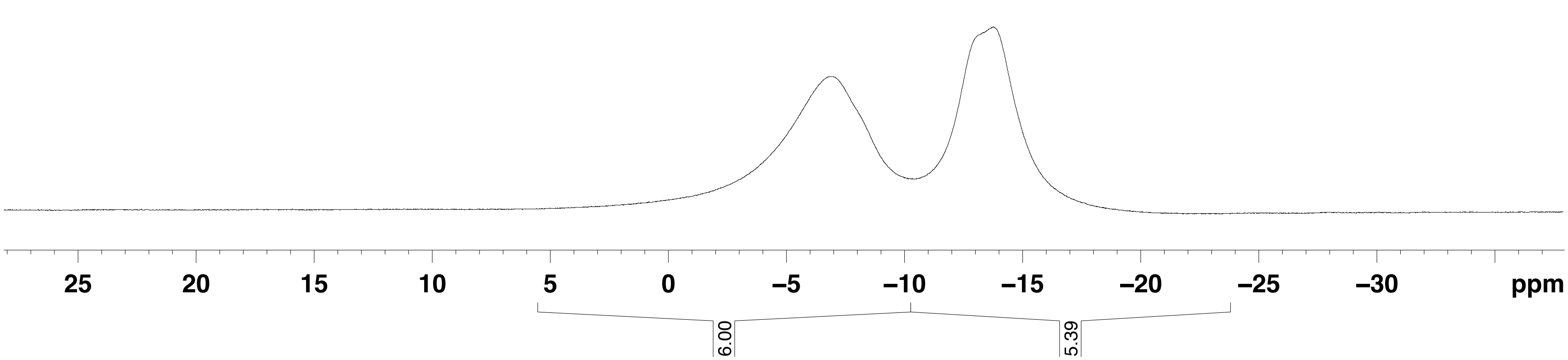


```
Current Data Parameters
NAME        penta-CH2CH2Ph
EXPNO                    2
PROCNO                   1

F2 - Acquisition Parameters
Date_             20180503
Time                 16.57
INSTRUM              spect
PROBHD    5 mm PABBO BB/
PULPROG                 zg
TD                   65536
SOLVENT            Acetone
NS                     128
DS                       4
SWH              25510.203 Hz
FIDRES            0.389255 Hz
AQ               1.2845056 sec
RG                  193.34
DW                  19.600 usec
DE                    6.50 usec
TE                   294.7 K
D1              1.00000000 sec
TD0                      1

======== CHANNEL f1 ========
NUC1                   11B
P1                    9.93 usec
PLW1           52.96599960 W
SFO1           128.3776052 MHz

F2 - Processing parameters
SI                   32768
SF             128.3776050 MHz
WDW                     EM
SSB      0
LB                    2.00 Hz
GB       0
PC                    1.40
```

Penta–CH2CH2–Ph product 50 mg in 0.6 ml acetone–d6
11B{1H} NMR, 128 MHz, 23 C

```
Current Data Parameters
NAME     penta-CH2CH2Ph
EXPNO                 3
PROCNO                1

F2 - Acquisition Parameters
Date_          20180503
Time              17.03
INSTRUM           spect
PROBHD   5 mm PABBO BB/
PULPROG          zgpg30
TD                65536
SOLVENT         Acetone
NS                  128
DS                    4
SWH           25510.203 Hz
FIDRES         0.389255 Hz
AQ            1.2845056 sec
RG               193.34
DW               19.600 usec
DE                 6.50 usec
TE                295.7 K
D1           1.00000000 sec
D11          0.03000000 sec
TD0                   1

======== CHANNEL f1 ========
NUC1                11B
P1                 9.93 usec
PLW1        52.96599960 W
SFO1       128.3776050 MHz

======== CHANNEL f2 ========
CPDPRG[2        waltz16
NUC2                 1H
PCPD2             80.00 usec
PLW2        12.50000000 W
PLW12        0.43945000 W
PLW13        0.28125000 W
SFO2       400.1320007 MHz

F2 - Processing parameters
SI                32768
SF         128.3776050 MHz
WDW                  EM
SSB      0
LB                 3.00 Hz
GB       0
PC                 1.40
```

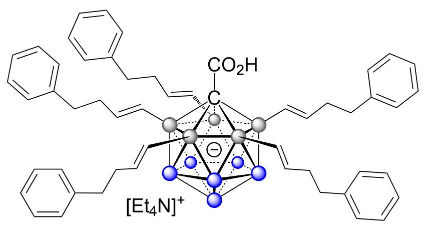

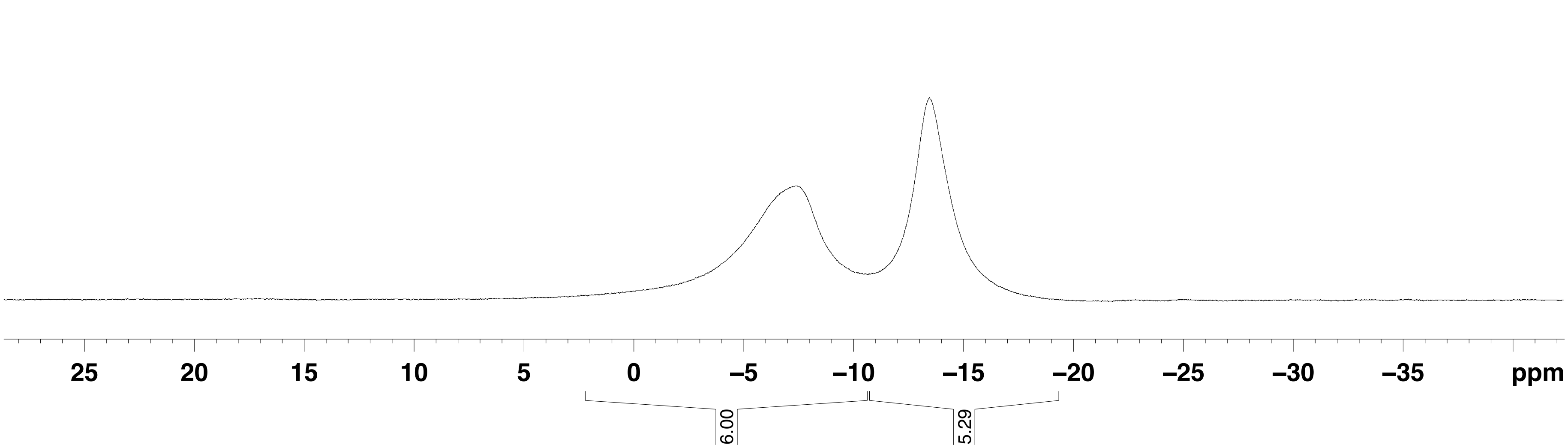

Penta–CH2CH2–Ph product 50 mg in 0.6 ml acetone–d6 *
13C{1H} NMR, 101 MHz, 23 C

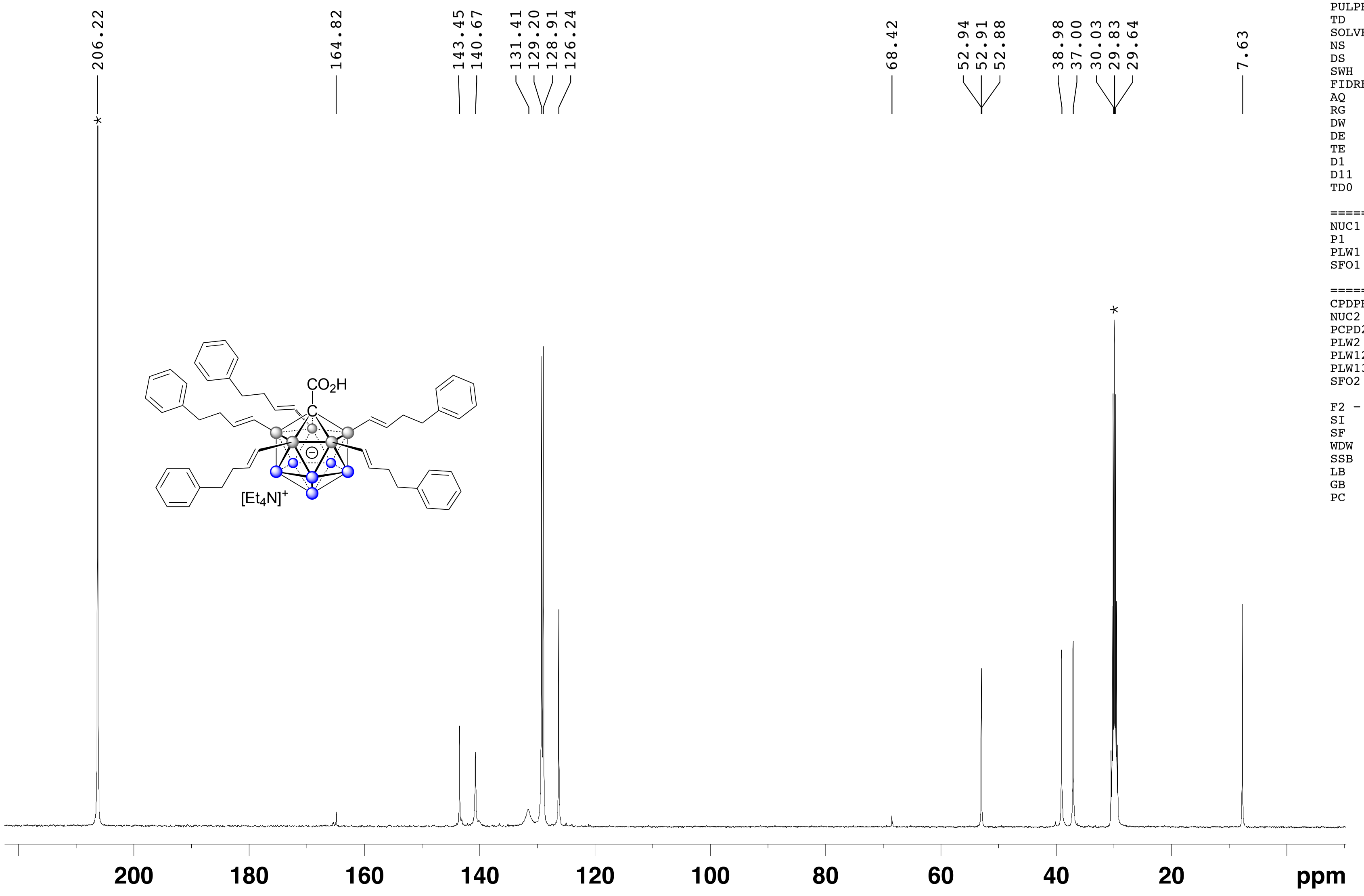


```
Current Data Parameters
NAME       penta-CH2CH2Ph
EXPNO                   4
PROCNO                  1

F2 - Acquisition Parameters
Date_            20180503
Time                18.36
INSTRUM             spect
PROBHD   5 mm PABBO BB/
PULPROG            zgpg30
TD                  65536
SOLVENT           Acetone
NS                   2048
DS                      4
SWH            29761.904 Hz
FIDRES          0.454131 Hz
AQ             1.1010048 sec
RG                 193.34
DW                 16.800 usec
DE                   6.50 usec
TE                  296.1 K
D1            1.50000000 sec
D11           0.03000000 sec
TD0                     1

======== CHANNEL f1 ========
NUC1                  13C
P1                  10.00 usec
PLW1          53.00000000 W
SFO1          100.6228293 MHz

======== CHANNEL f2 ========
CPDPRG[2          waltz16
NUC2                   1H
PCPD2               80.00 usec
PLW2          12.50000000 W
PLW12          0.43945000 W
PLW13          0.28125000 W
SFO2          400.1316005 MHz

F2 - Processing parameters
SI                  32768
SF            100.6126926 MHz
WDW                    EM
SSB       0
LB                   6.00 Hz
GB        0
PC                   1.40
```

penta–CH2CH2COOEt product 15 mg in 0.6 ml acetone–d6 *
1H{1B} NMR, 500 MHz, 23 C

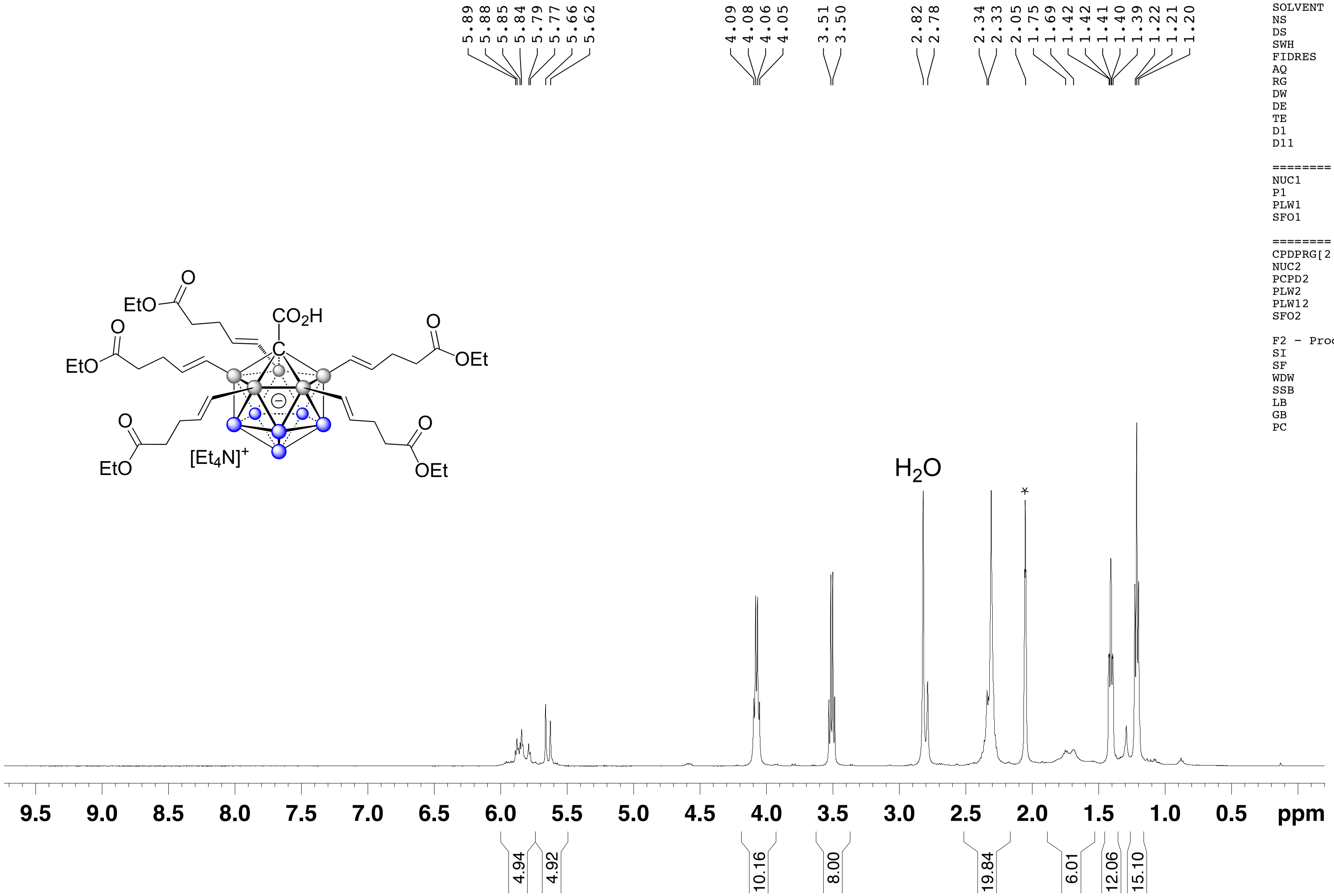


```
Current Data Parameters
NAME      penta-CH2CH2COOEt-1H
EXPNO                    1
PROCNO                   1

F2 - Acquisition Parameters
Date_             20180413
Time                 18.44
INSTRUM              spect
PROBHD   5 mm PABBO BB-
PULPROG             zgig30
TD                   65536
SOLVENT            Acetone
NS                      32
DS                       0
SWH              12500.000 Hz
FIDRES            0.190735 Hz
AQ               2.6214399 sec
RG                     114
DW                  40.000 usec
DE                    6.50 usec
TE                   296.9 K
D1              5.00000000 sec
D11             0.03000000 sec

======== CHANNEL f1 ========
NUC1                    1H
P1                   11.70 usec
PLW1           19.00000000 W
SFO1          500.1335009 MHz

======== CHANNEL f2 ========
CPDPRG[2              garp
NUC2                   11B
PCPD2               100.00 usec
PLW2           95.00000000 W
PLW12           1.63030005 W
SFO2          160.4615690 MHz

F2 - Processing parameters
SI                   65536
SF             500.1300101 MHz
WDW                     EM
SSB      0
LB                    1.00 Hz
GB       0
PC                    1.00
```

penta–CH2CH2COOEt product 40 mg in 0.6 ml acetone–d6
1B NMR, 160 MHz, 23 C

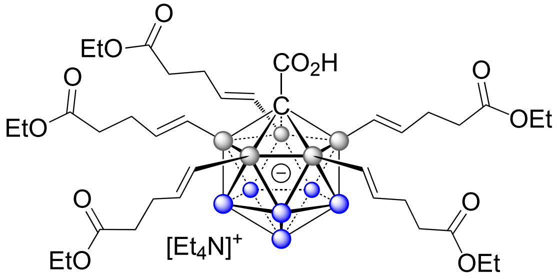

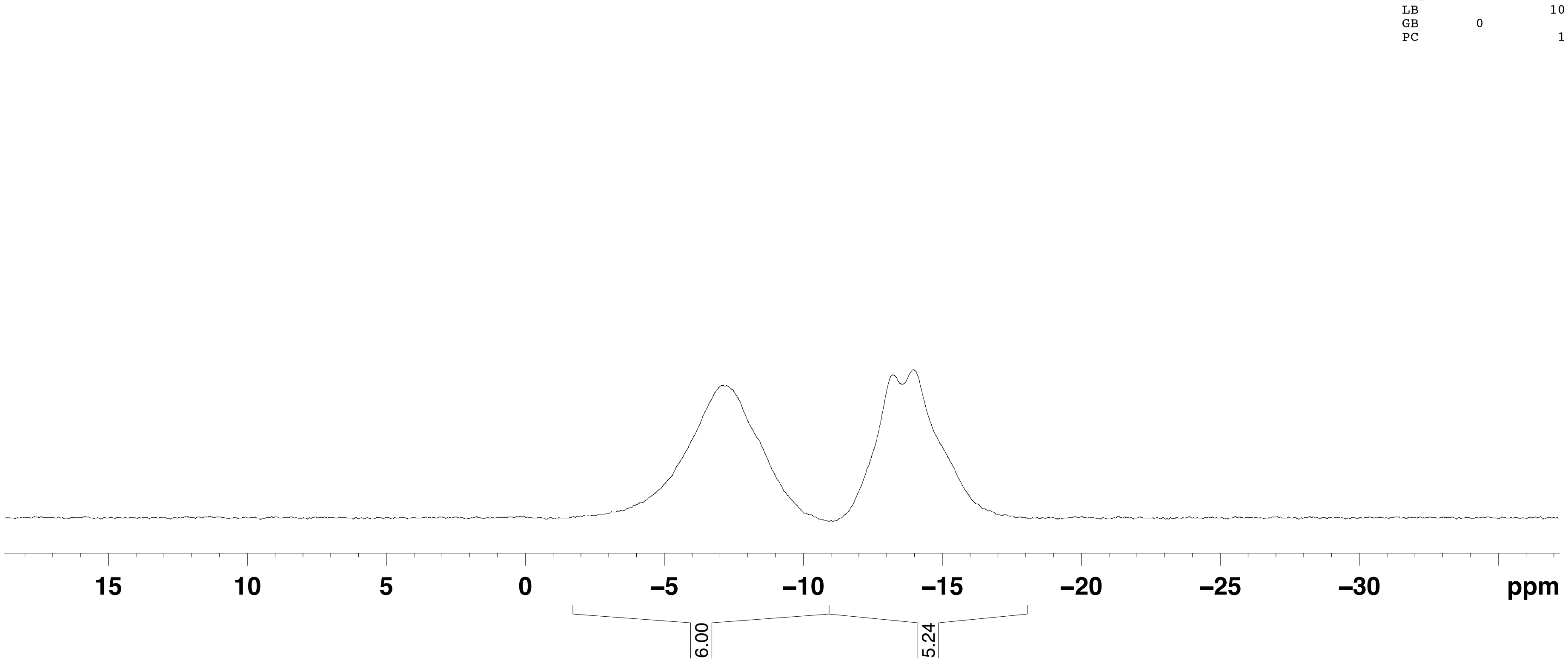


```
Current Data Parameters
NAME     penta-CH2CH2COOEt
EXPNO                   2
PROCNO                  1

F2 - Acquisition Parameters
Date_            20180412
Time                 7.12
INSTRUM             spect
PROBHD   5 mm PABBO BB-
PULPROG              zg30
TD                  64098
SOLVENT           Acetone
NS                     64
DS                      0
SWH             32051.281 Hz
FIDRES           0.500036 Hz
AQ              0.9999288 sec
RG                    203
DW                 15.600 usec
DE                   6.50 usec
TE                  295.5 K
D1             1.00000000 sec

======== CHANNEL f1 ========
NUC1                  11B
P1                  13.10 usec
PLW1          95.00000000 W
SFO1          160.4615792 MHz

F2 - Processing parameters
SI                  32768
SF            160.4615790 MHz
WDW                    EM
SSB      0
LB                  10.00 Hz
GB       0
PC                   1.40
```

penta–CH2CH2COOEt product 40 mg in 0.6 ml acetone–d6
1B{1H} NMR, 160 MHz, 23 C

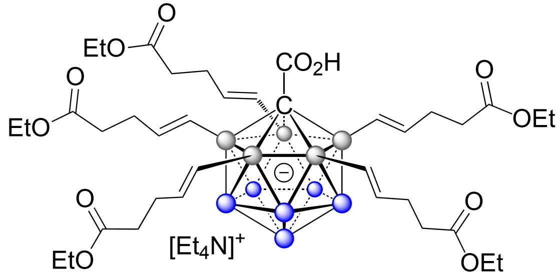

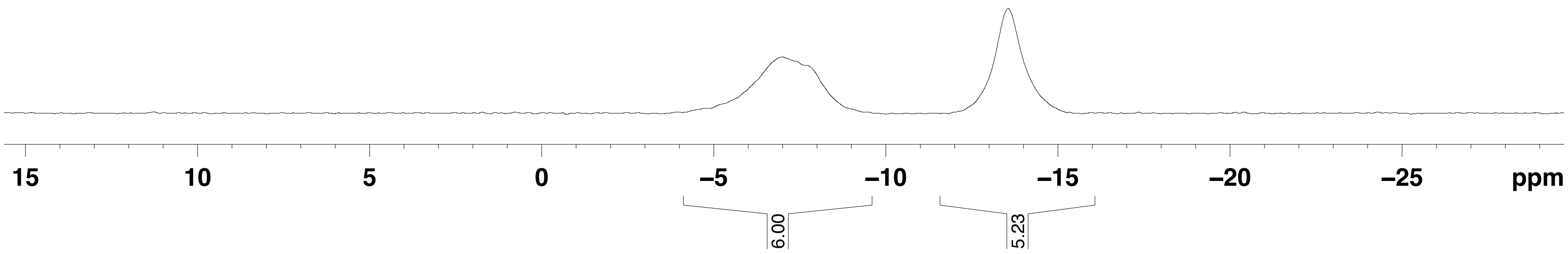


```
Current Data Parameters
NAME       penta-CH2CH2COOEt-13C
EXPNO                  3
PROCNO                 1

F2 - Acquisition Parameters
Date_           20181018
Time                7.06
INSTRUM            spect
PROBHD    5 mm PABBO BB-
PULPROG           zgpg30
TD                 65536
SOLVENT          Acetone
NS                    64
DS                     0
SWH            32051.281 Hz
FIDRES          0.489064 Hz
AQ             1.0223616 sec
RG                   203
DW                15.600 usec
DE                  6.50 usec
TE                 296.6 K
D1            1.00000000 sec
D11           0.03000000 sec
TD0                    1

======== CHANNEL f1 ========
SFO1         160.4615790 MHz
NUC1                 11B
P1                 13.10 usec
PLW1         95.00000000 W

======== CHANNEL f2 ========
SFO2         500.1325007 MHz
NUC2                  1H
CPDPRG[2         waltz16
PCPD2              80.00 usec
PLW2         19.00000000 W
PLW12         0.40639001 W
PLW13         0.26008999 W

F2 - Processing parameters
SI                 32768
SF           160.4615790 MHz
WDW                   EM
SSB      0
LB                 20.00 Hz
GB       0
PC                  1.40
```

penta–CH2CH2COOEt product 40 mg in 0.6 ml acetone–d6
13C{1H} NMR, 126 MHz, 23 C

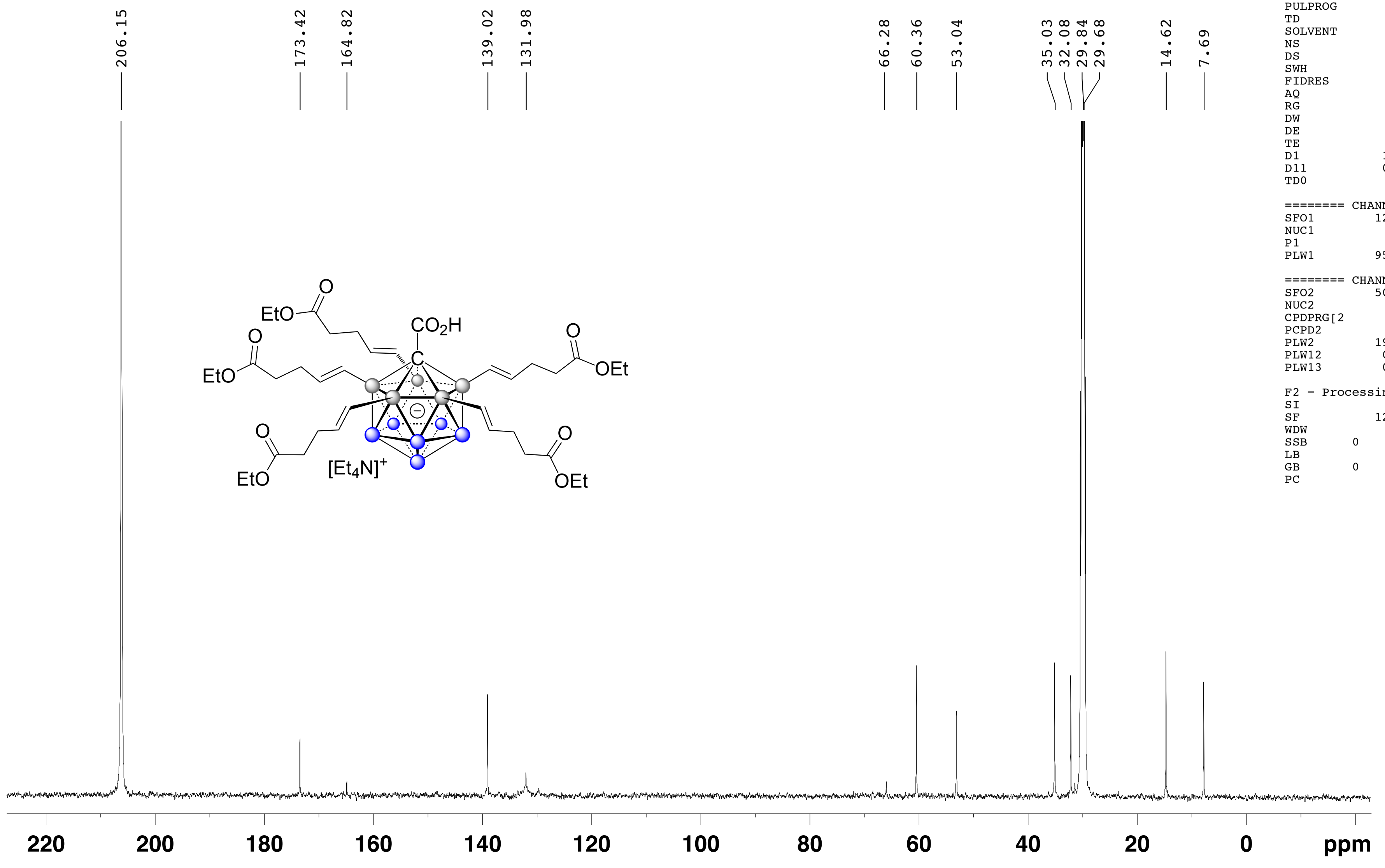


```
Current Data Parameters
NAME      penta-CH2CH2COOEt-13C
EXPNO                  4
PROCNO                 1

F2 - Acquisition Parameters
Date_           20181018
Time                8.34
INSTRUM            spect
PROBHD    5 mm PABBO BB-
PULPROG          zgpg30
TD                65536
SOLVENT          Acetone
NS                 2148
DS                    4
SWH          37878.789 Hz
FIDRES        0.577984 Hz
AQ           0.8650752 sec
RG                  203
DW               13.200 usec
DE                 6.50 usec
TE                297.0 K
D1           1.50000000 sec
D11          0.03000000 sec
TD0                   1

======== CHANNEL f1 ========
SFO1        125.7716224 MHz
NUC1                13C
P1                10.70 usec
PLW1        95.00000000 W

======== CHANNEL f2 ========
SFO2        500.1320005 MHz
NUC2                 1H
CPDPRG[2        waltz16
PCPD2             80.00 usec
PLW2        19.00000000 W
PLW12        0.40639001 W
PLW13        0.26008999 W

F2 - Processing parameters
SI                32768
SF          125.7576765 MHz
WDW                  EM
SSB      0
LB                 7.00 Hz
GB       0
PC                 1.40
```

Penta–CH2OPh product 40 mg in 0.6 ml acetone–d6 *
1H{11B} NMR, 500 MHz, 23 C

```
Current Data Parameters
NAME        penta-CH2OPh
EXPNO                  1
PROCNO                 1

F2 - Acquisition Parameters
Date_           20180119
Time                4.48
INSTRUM            spect
PROBHD   5 mm PABBO BB-
PULPROG          zgig30
TD                 65536
SOLVENT          Acetone
NS                    16
DS                     0
SWH            12500.000 Hz
FIDRES          0.190735 Hz
AQ             2.6214399 sec
RG                    57
DW                40.000 usec
DE                  6.50 usec
TE                 296.0 K
D1            5.00000000 sec
D11           0.03000000 sec

======== CHANNEL f1 ========
NUC1                  1H
P1                 11.70 usec
PLW1         19.00000000 W
SFO1         500.1335009 MHz

======== CHANNEL f2 ========
CPDPRG[2            garp
NUC2                 11B
PCPD2             100.00 usec
PLW2         95.00000000 W
PLW12         1.63030005 W
SFO2         160.4615690 MHz

F2 - Processing parameters
SI                 65536
SF           500.1300101 MHz
WDW                   EM
SSB      0
LB                  1.00 Hz
GB       0
PC                  1.00
```

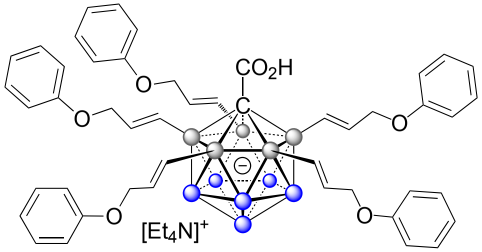

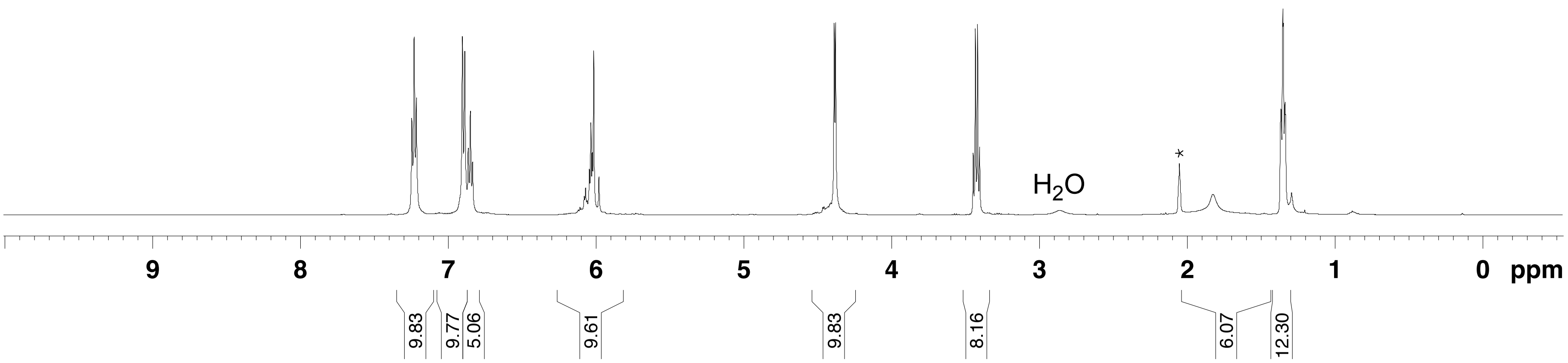

Penta–CH2OPh product 40 mg in 0.6 ml acetone–d6
11B NMR, 160 MHz, 23 C

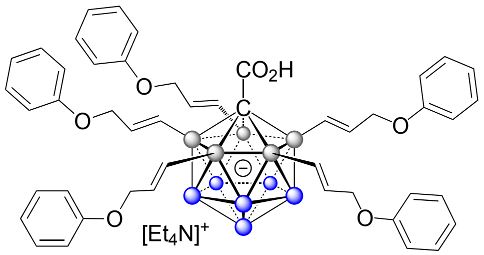

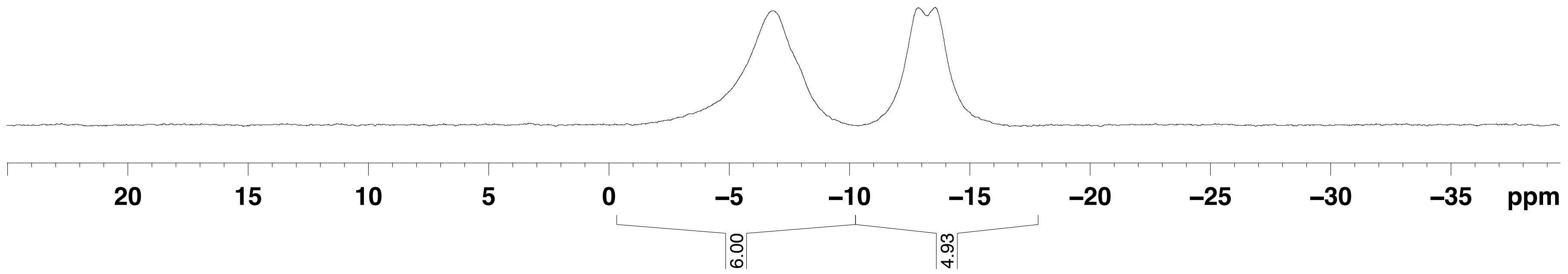


```
Current Data Parameters
NAME        penta-CH2OPh
EXPNO                  2
PROCNO                 1

F2 - Acquisition Parameters
Date_           20180119
Time                4.51
INSTRUM            spect
PROBHD   5 mm PABBO BB-
PULPROG             zg30
TD                 64098
SOLVENT          Acetone
NS                    64
DS                     0
SWH          32051.281 Hz
FIDRES        0.500036 Hz
AQ           0.9999288 sec
RG                   203
DW              15.600 usec
DE                6.50 usec
TE               295.7 K
D1        1.00000000 sec

======== CHANNEL f1 ========
NUC1                 11B
P1                 13.10 usec
PLW1         95.00000000 W
SFO1        160.4615792 MHz

F2 - Processing parameters
SI                 32768
SF          160.4615790 MHz
WDW                   EM
SSB      0
LB                 10.00 Hz
GB       0
PC                  1.40
```

Penta–CH2OPh product 40 mg in 0.6 ml acetone–d6
11B{1H} NMR, 160 MHz, 23 C

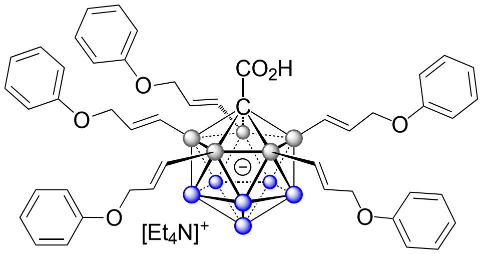

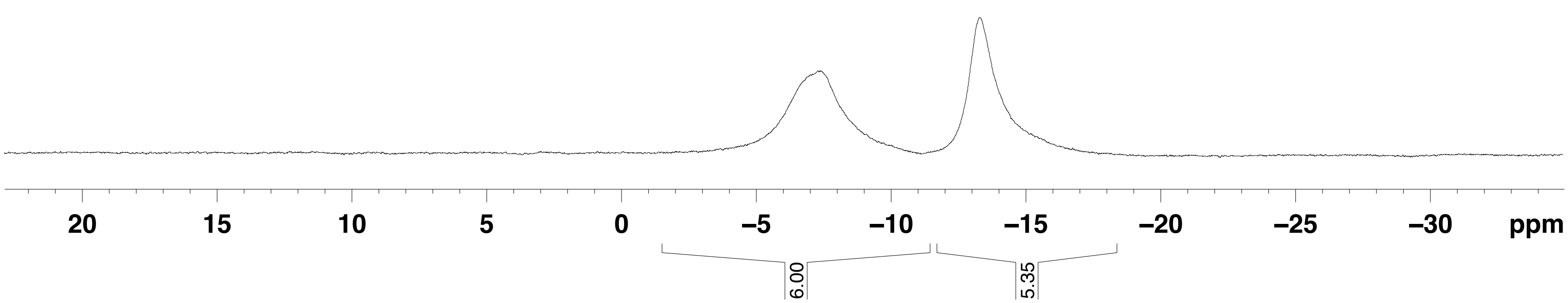


```
Current Data Parameters
NAME          penta-CH2OPh
EXPNO                    3
PROCNO                   1

F2 - Acquisition Parameters
Date_             20180119
Time                  4.54
INSTRUM              spect
PROBHD   5 mm PABBO BB-
PULPROG             zgpg30
TD                   65536
SOLVENT            Acetone
NS                      64
DS                       0
SWH              32051.281 Hz
FIDRES            0.489064 Hz
AQ               1.0223616 sec
RG                     203
DW                  15.600 usec
DE                    6.50 usec
TE                   296.1 K
D1              1.00000000 sec
D11             0.03000000 sec

======== CHANNEL f1 ========
NUC1                   11B
P1                   13.10 usec
PLW1           95.00000000 W
SFO1           160.4615790 MHz

======== CHANNEL f2 ========
CPDPRG[2           waltz16
NUC2                    1H
PCPD2                80.00 usec
PLW2           19.00000000 W
PLW12           0.40639001 W
PLW13           0.26008999 W
SFO2           500.1325007 MHz

F2 - Processing parameters
SI                   32768
SF             160.4615790 MHz
WDW                     EM
SSB      0
LB                    4.00 Hz
GB       0
PC                    1.40
```

Penta-CH2OPh product 40 mg in 0.6 ml acetone-d6 *
13C{1H} NMR, 126 MHz, 23 C

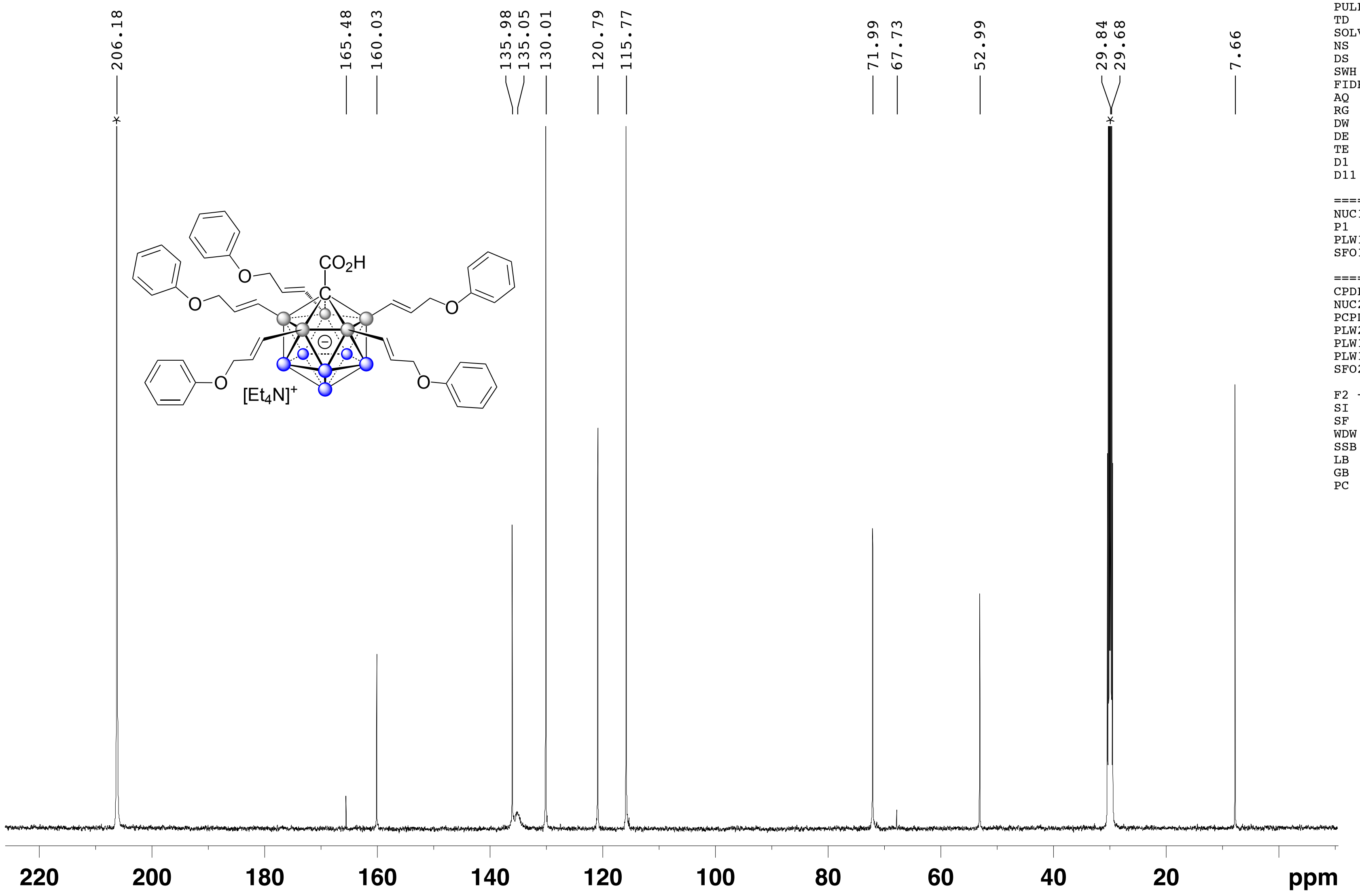


```
Current Data Parameters
NAME        penta-CH2OPh
EXPNO                  4
PROCNO                 1

F2 - Acquisition Parameters
Date_           20180119
Time                6.20
INSTRUM            spect
PROBHD   5 mm PABBO BB-
PULPROG          zgpg30
TD                 65536
SOLVENT          Acetone
NS                  2100
DS                     4
SWH           37878.789 Hz
FIDRES         0.577984 Hz
AQ            0.8650752 sec
RG                   203
DW               13.200 usec
DE                 6.50 usec
TE                296.4 K
D1           1.50000000 sec
D11          0.03000000 sec

======== CHANNEL f1 ========
NUC1                 13C
P1                 10.50 usec
PLW1         95.00000000 W
SFO1         125.7716224 MHz

======== CHANNEL f2 ========
CPDPRG[2         waltz16
NUC2                  1H
PCPD2              80.00 usec
PLW2         19.00000000 W
PLW12         0.40639001 W
PLW13         0.26008999 W
SFO2         500.1320005 MHz

F2 - Processing parameters
SI                 32768
SF           125.7576822 MHz
WDW                   EM
SSB      0
LB                  3.00 Hz
GB       0
PC                  1.40
```

12–Cl–Penta–styrine product 50 mg in 0.6 ml acetone–d6 *
1H{11B} NMR, 500 MHz, 23 C

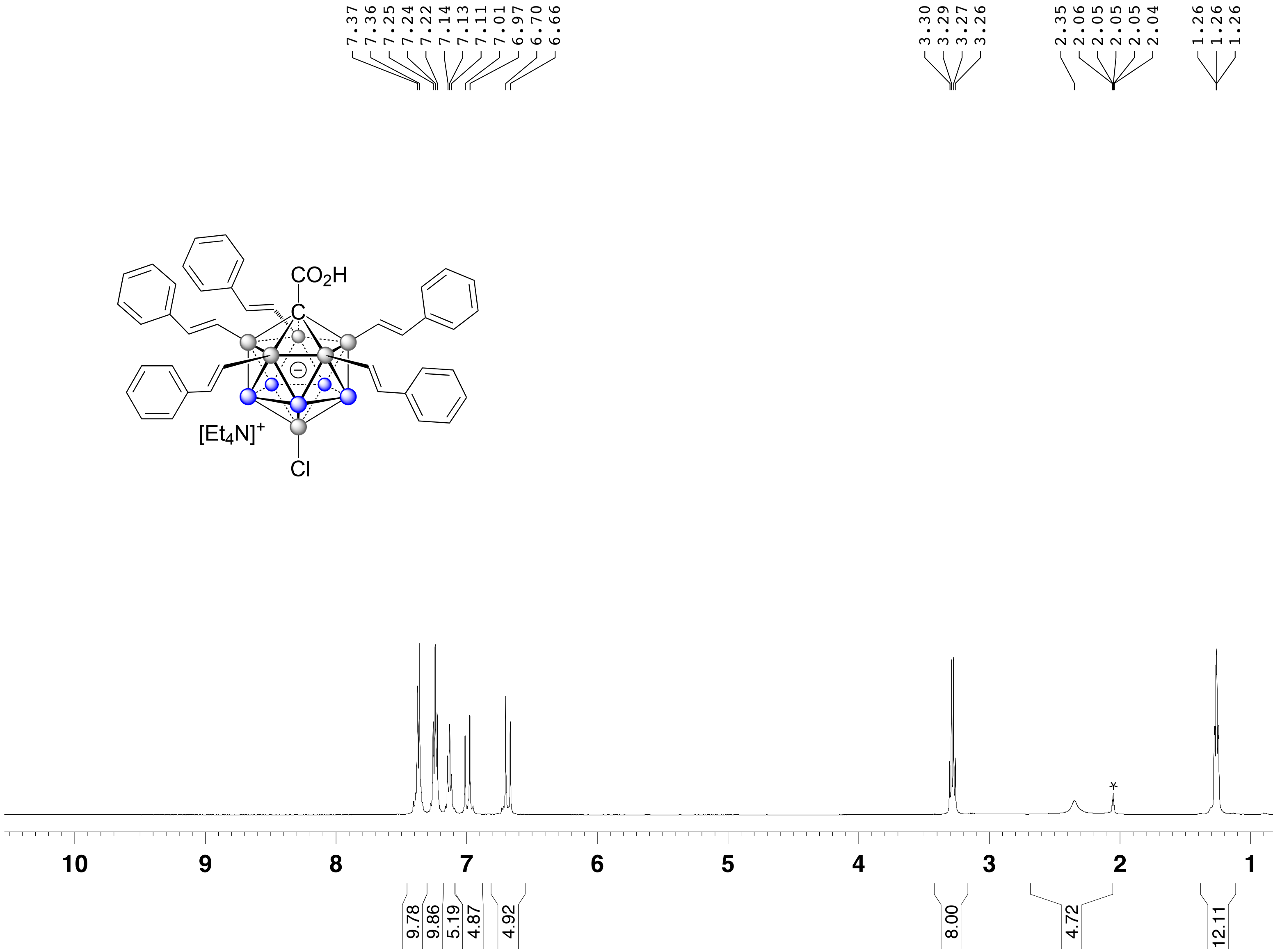


```
Current Data Parameters
NAME      12-Cl-penta-styrene
EXPNO                   1
PROCNO                  1

F2 - Acquisition Parameters
Date_            20180612
Time                 2.31
INSTRUM             spect
PROBHD   5 mm PABBO BB-
PULPROG            zgig30
TD                  65536
SOLVENT           Acetone
NS                     16
DS                      0
SWH             12500.000 Hz
FIDRES           0.190735 Hz
AQ              2.6214399 sec
RG                   40.3
DW                 40.000 usec
DE                   6.50 usec
TE                  296.0 K
D1             5.00000000 sec
D11            0.03000000 sec

======== CHANNEL f1 ========
NUC1                   1H
P1                  11.70 usec
PLW1          19.00000000 W
SFO1         500.1335009 MHz

======== CHANNEL f2 ========
CPDPRG[2             garp
NUC2                  11B
PCPD2              100.00 usec
PLW2          95.00000000 W
PLW12          1.63030005 W
SFO2         160.4615690 MHz

F2 - Processing parameters
SI                  65536
SF           500.1300100 MHz
WDW                    EM
SSB      0
LB                   1.00 Hz
GB       0
PC                   1.00
```

12–Cl–Penta–styrine product 50 mg in 0.6 ml acetone–d6
11B NMR, 160 MHz, 23 C

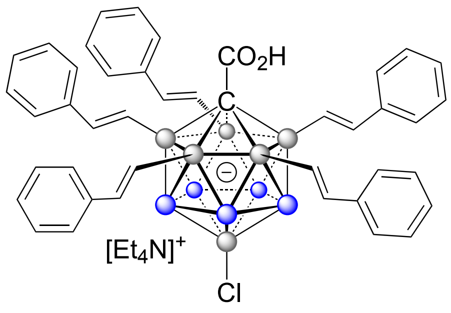

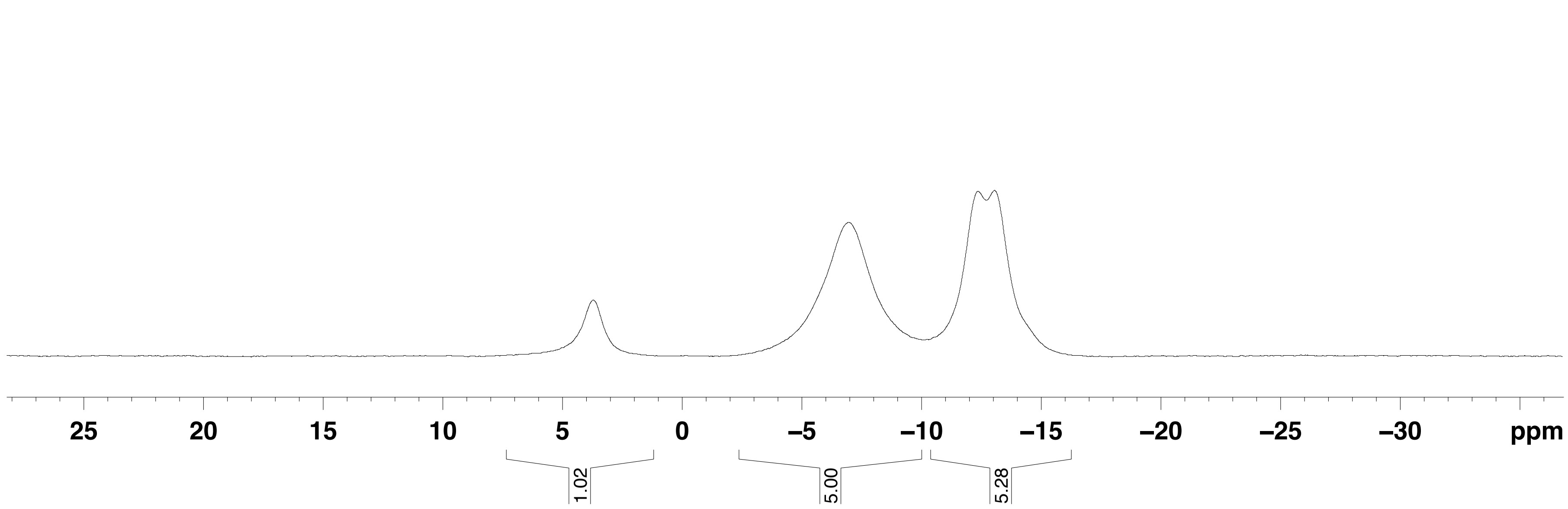


```
Current Data Parameters
NAME     12-Cl-penta-styrene
EXPNO                  2
PROCNO                 1

F2 - Acquisition Parameters
Date_           20180612
Time                2.34
INSTRUM            spect
PROBHD   5 mm PABBO BB-
PULPROG             zg30
TD                 64098
SOLVENT          Acetone
NS                    64
DS                     0
SWH          32051.281 Hz
FIDRES        0.500036 Hz
AQ           0.9999288 sec
RG                   203
DW              15.600 usec
DE                6.50 usec
TE               295.8 K
D1          1.00000000 sec

======== CHANNEL f1 ========
NUC1                 11B
P1                 13.10 usec
PLW1         95.00000000 W
SFO1        160.4615792 MHz

F2 - Processing parameters
SI                 32768
SF          160.4615790 MHz
WDW                   EM
SSB      0
LB                 10.00 Hz
GB       0
PC                  1.40
```

12–Cl–Penta–styrine product 50 mg in 0.6 ml acetone–d6
11B{1H} NMR, 160 MHz, 23 C

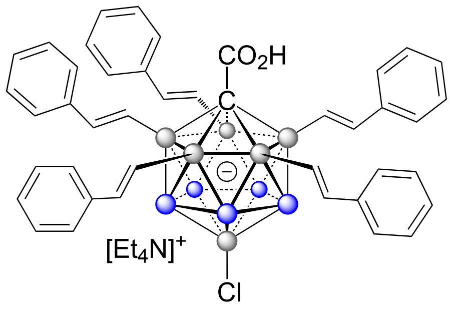

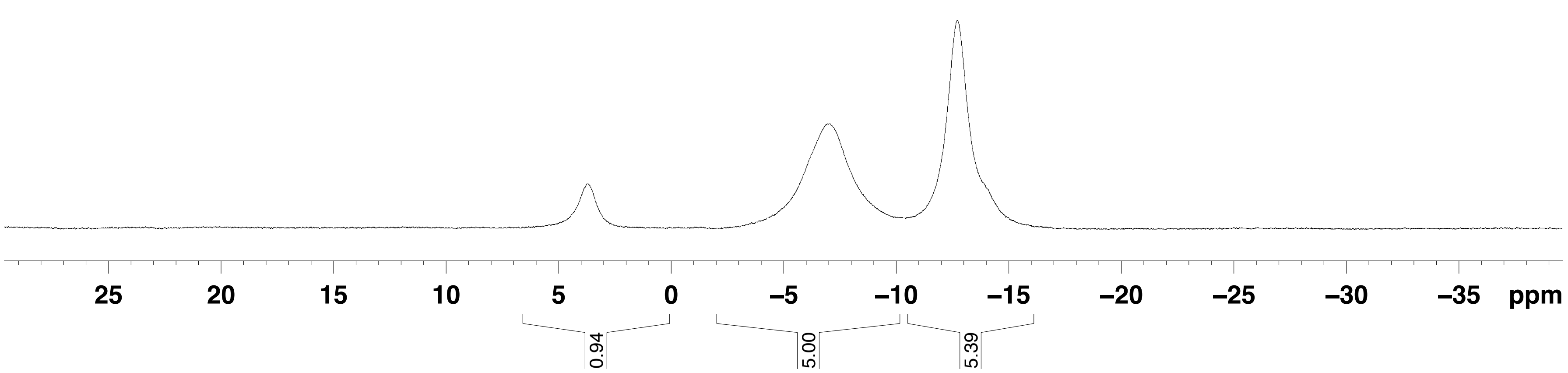


```
Current Data Parameters
NAME     12-Cl-penta-styrene
EXPNO                  3
PROCNO                 1

F2 - Acquisition Parameters
Date_           20180612
Time                2.37
INSTRUM            spect
PROBHD   5 mm PABBO BB-
PULPROG          zgpg30
TD                 65536
SOLVENT          Acetone
NS                    64
DS                     0
SWH          32051.281 Hz
FIDRES        0.489064 Hz
AQ           1.0223616 sec
RG                   203
DW                15.600 usec
DE                  6.50 usec
TE                 296.4 K
D1            1.00000000 sec
D11           0.03000000 sec

======== CHANNEL f1 ========
NUC1                 11B
P1                 13.10 usec
PLW1         95.00000000 W
SFO1         160.4615790 MHz

======== CHANNEL f2 ========
CPDPRG[2         waltz16
NUC2                  1H
PCPD2              80.00 usec
PLW2         19.00000000 W
PLW12         0.40639001 W
PLW13         0.26008999 W
SFO2         500.1325007 MHz

F2 - Processing parameters
SI                 32768
SF           160.4615790 MHz
WDW                   EM
SSB      0
LB                  3.00 Hz
GB       0
PC                  1.40
```

12–Cl–Penta–styrine product 50 mg in 0.6 ml acetone–d6*
13C{1H} NMR, 126 MHz, 23 C

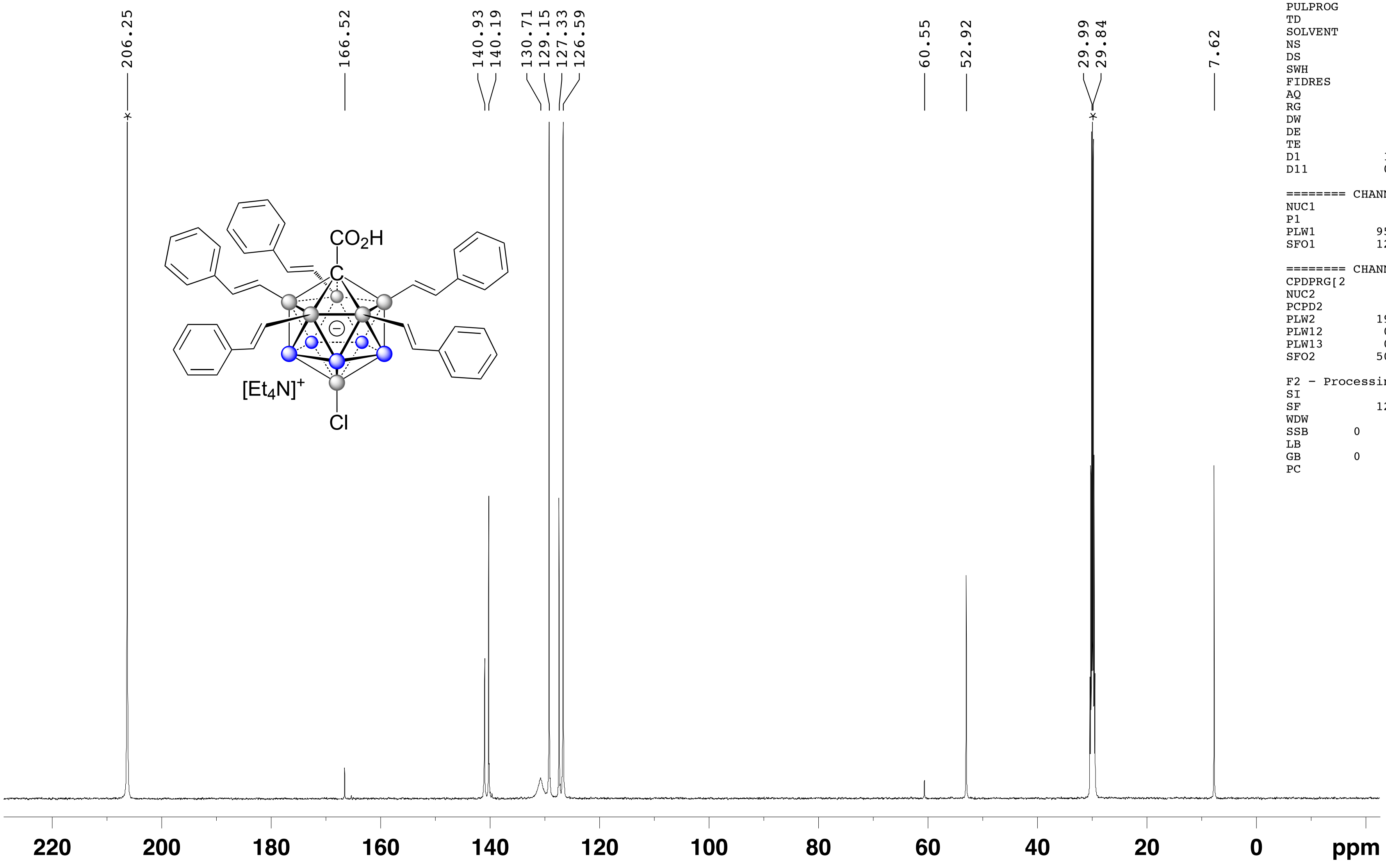


```
Current Data Parameters
NAME      12-Cl-penta-styrene
EXPNO                   4
PROCNO                  1

F2 - Acquisition Parameters
Date_            20180612
Time                 4.03
INSTRUM             spect
PROBHD   5 mm PABBO BB-
PULPROG           zgpg30
TD                 65536
SOLVENT          Acetone
NS                  2100
DS                     4
SWH           37878.789 Hz
FIDRES         0.577984 Hz
AQ            0.8650752 sec
RG                   203
DW                13.200 usec
DE                  6.50 usec
TE                 296.6 K
D1           1.50000000 sec
D11          0.03000000 sec

======== CHANNEL f1 ========
NUC1                 13C
P1                 10.50 usec
PLW1         95.00000000 W
SFO1        125.7716224 MHz

======== CHANNEL f2 ========
CPDPRG[2         waltz16
NUC2                  1H
PCPD2              80.00 usec
PLW2         19.00000000 W
PLW12         0.40639001 W
PLW13         0.26008999 W
SFO2        500.1320005 MHz

F2 - Processing parameters
SI                 32768
SF          125.7576880 MHz
WDW                   EM
SSB      0
LB                  1.00 Hz
GB       0
PC                  1.40
```

12–Br–Penta–F–styrene product 50 mg in 0.6 ml acetone–d6*
1H{11B} NMR, 500 MHz, 23 C

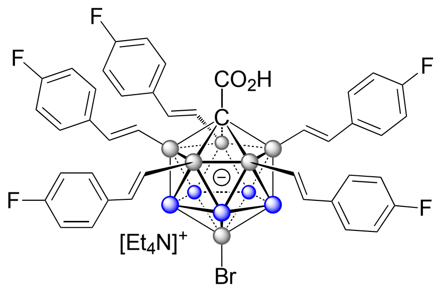

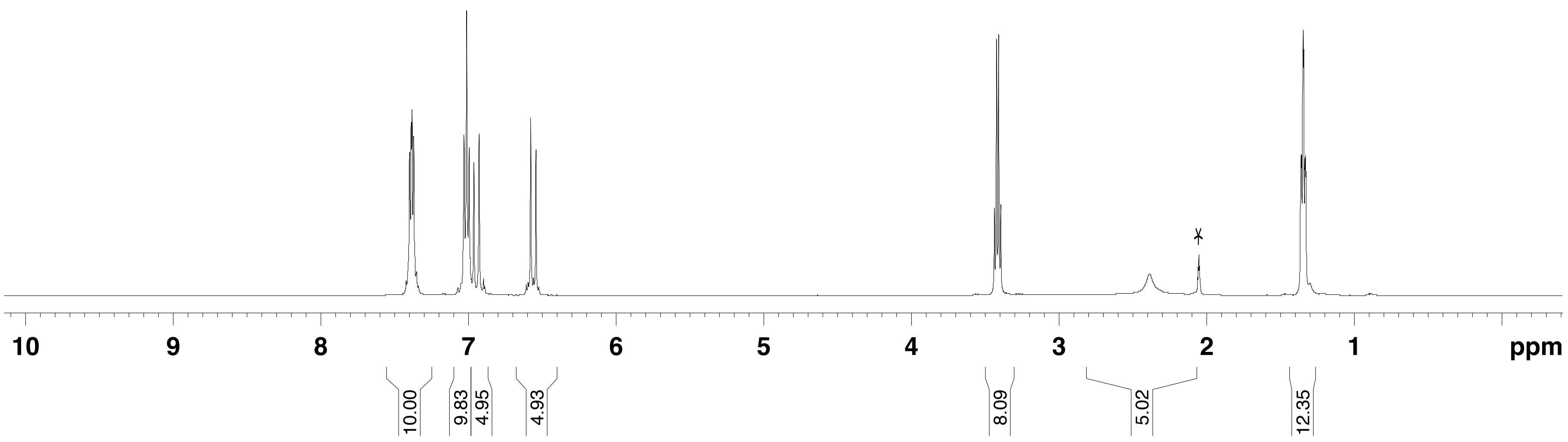

Current Data Parameters		
NAME	12-Br-penta-4-F-styrene	
EXPNO	1	
PROCNO	1	
F2 - Acquisition Parameters		
Date_	20180507	
Time	23.12	
INSTRUM	spect	
PROBHD	5 mm PABBO BB-	
PULPROG	zgig30	
TD	65536	
SOLVENT	Acetone	
NS	16	
DS	0	
SWH	12500.000	Hz
FIDRES	0.190735	Hz
AQ	2.6214399	sec
RG	45.2	
DW	40.000	usec
DE	6.50	usec
TE	296.2	K
D1	5.00000000	sec
D11	0.03000000	sec
======== CHANNEL f1 ========		
NUC1	1H	
P1	11.70	usec
PLW1	19.00000000	W
SFO1	500.1335009	MHz
======== CHANNEL f2 ========		
CPDPRG[2	garp	
NUC2	11B	
PCPD2	100.00	usec
PLW2	95.00000000	W
PLW12	1.63030005	W
SFO2	160.4615690	MHz
F2 - Processing parameters		
SI	65536	
SF	500.1300101	MHz
WDW	EM	
SSB	0	
LB	1.00	Hz
GB	0	
PC	1.00	

12–Br–Penta–F–styrene product 50 mg in 0.6 ml acetone–d6
11B NMR, 160 MHz, 23 C

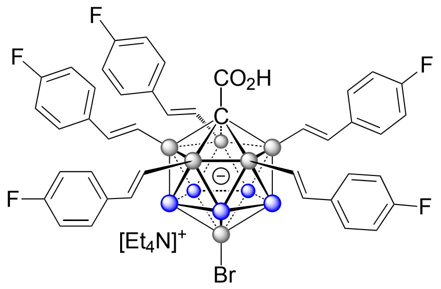

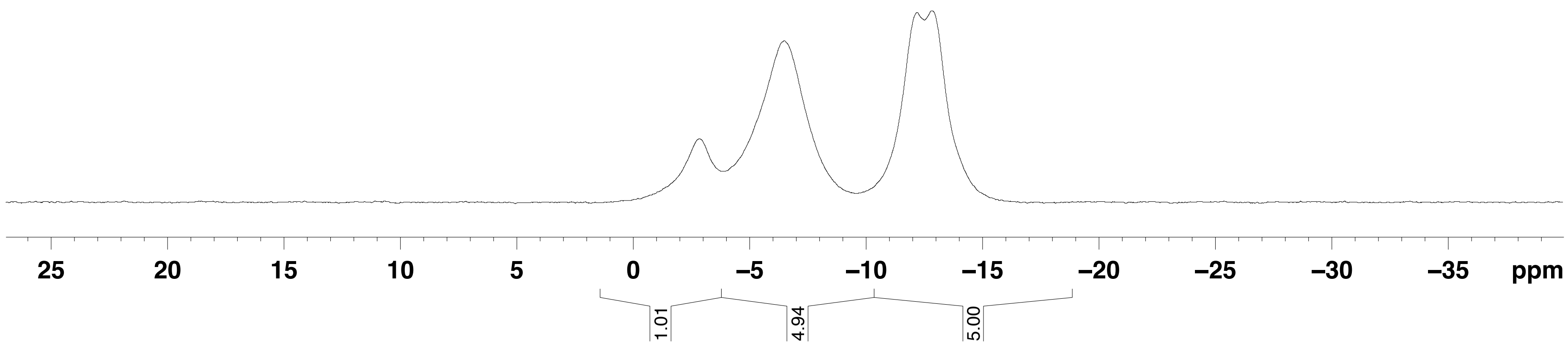


```
Current Data Parameters
NAME     12-Br-penta-4-F-styrene
EXPNO                    2
PROCNO                   1

F2 - Acquisition Parameters
Date_          20180507
Time              23.15
INSTRUM           spect
PROBHD   5 mm PABBO BB-
PULPROG            zg30
TD                64098
SOLVENT         Acetone
NS                   64
DS                    0
SWH           32051.281 Hz
FIDRES         0.500036 Hz
AQ            0.9999288 sec
RG                  203
DW               15.600 usec
DE                 6.50 usec
TE                295.7 K
D1           1.00000000 sec

======== CHANNEL f1 ========
NUC1                11B
P1                13.10 usec
PLW1        95.00000000 W
SFO1        160.4615792 MHz

F2 - Processing parameters
SI                32768
SF          160.4615790 MHz
WDW                  EM
SSB      0
LB                10.00 Hz
GB       0
PC                 1.40
```

12–Br–Penta–F–styrene product 50 mg in 0.6 ml acetone–d6
11B{1H} NMR, 160 MHz, 23 C

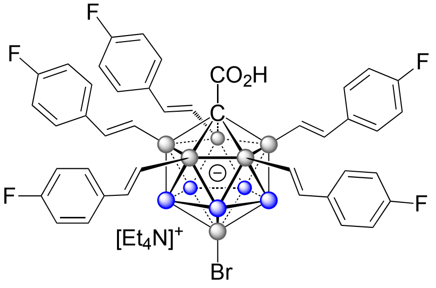

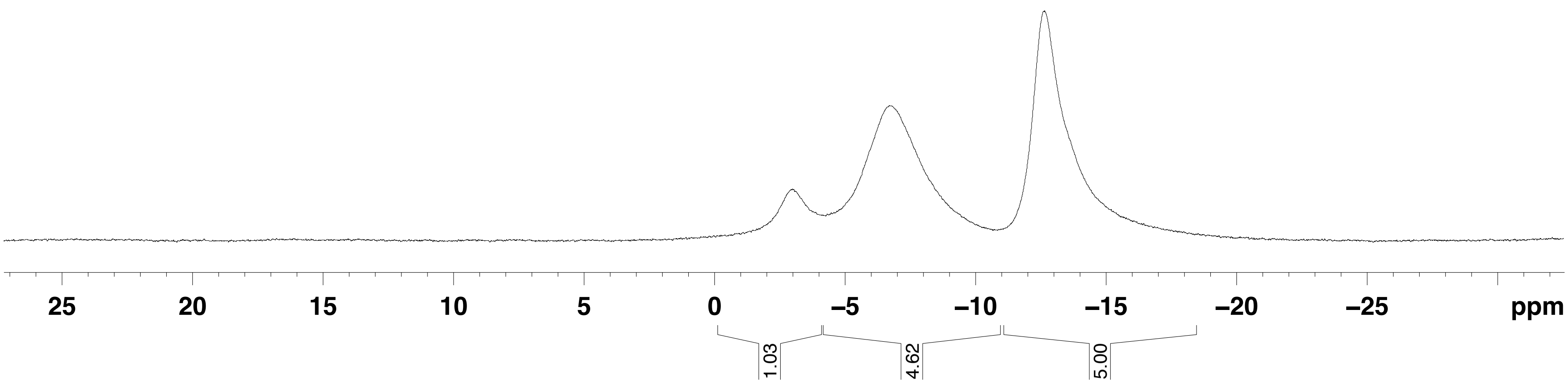


```
Current Data Parameters
NAME      12-Br-penta-4-F-styrene
EXPNO                    3
PROCNO                   1

F2 - Acquisition Parameters
Date_           20180507
Time               23.19
INSTRUM            spect
PROBHD   5 mm PABBO BB-
PULPROG          zgpg30
TD                65536
SOLVENT          Acetone
NS                   64
DS                    0
SWH          32051.281 Hz
FIDRES        0.489064 Hz
AQ           1.0223616 sec
RG                  203
DW               15.600 usec
DE                 6.50 usec
TE                295.8 K
D1           1.00000000 sec
D11          0.03000000 sec

======== CHANNEL f1 ========
NUC1                11B
P1                13.10 usec
PLW1        95.00000000 W
SFO1        160.4615790 MHz

======== CHANNEL f2 ========
CPDPRG[2         waltz16
NUC2                 1H
PCPD2             80.00 usec
PLW2        19.00000000 W
PLW12        0.40639001 W
PLW13        0.26008999 W
SFO2        500.1325007 MHz

F2 - Processing parameters
SI                32768
SF          160.4615790 MHz
WDW                  EM
SSB      0
LB                 3.00 Hz
GB       0
PC                 1.40
```

12–Br–Penta–F–styrene product 50 mg in 0.6 ml acetone–d6*
13C{1H} NMR, 126 MHz, 23 C

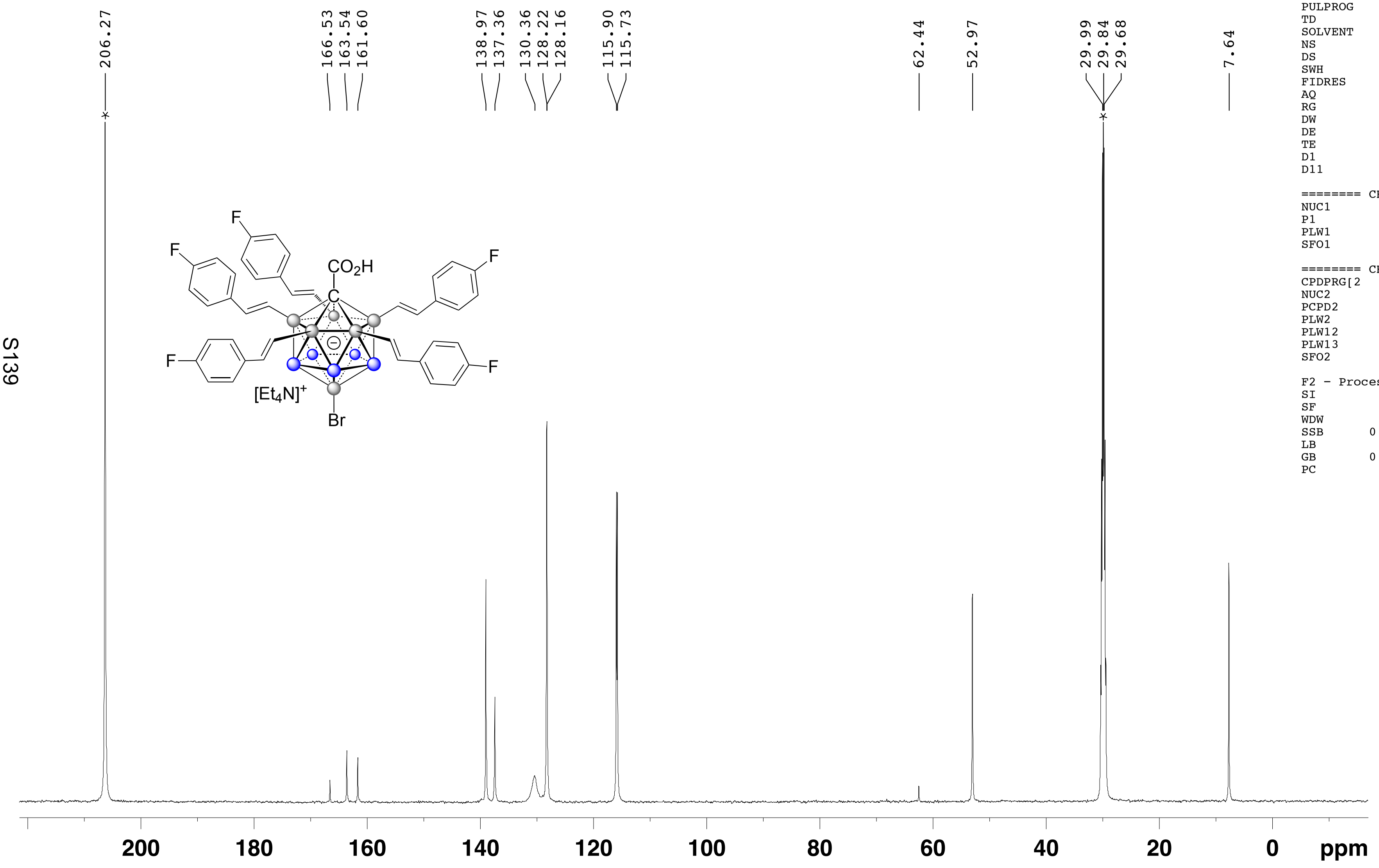


```
Current Data Parameters
NAME     12-Br-penta-4-F-styrene
EXPNO                  4
PROCNO                 1

F2 - Acquisition Parameters
Date_           20180508
Time                0.45
INSTRUM            spect
PROBHD   5 mm PABBO BB-
PULPROG          zgpg30
TD                65536
SOLVENT          Acetone
NS                 2100
DS                    4
SWH          37878.789 Hz
FIDRES        0.577984 Hz
AQ           0.8650752 sec
RG                  203
DW               13.200 usec
DE                 6.50 usec
TE                296.5 K
D1           1.50000000 sec
D11          0.03000000 sec

======== CHANNEL f1 ========
NUC1                13C
P1                10.50 usec
PLW1        95.00000000 W
SFO1        125.7716224 MHz

======== CHANNEL f2 ========
CPDPRG[2         waltz16
NUC2                 1H
PCPD2             80.00 usec
PLW2        19.00000000 W
PLW12        0.40639001 W
PLW13        0.26008999 W
SFO2        500.1320005 MHz

F2 - Processing parameters
SI                32768
SF          125.7576845 MHz
WDW                  EM
SSB      0
LB                10.00 Hz
GB       0
PC                 1.40
```

12–Me–Penta–F–styrine product 50 mg in 0.6 ml acetone–d6 *
1H{11B} NMR, 400 MHz, 23 C

```
Current Data Parameters
NAME      12-Me-penta-4-F-styrene
EXPNO                   1
PROCNO                  1

F2 - Acquisition Parameters
Date_           20180604
Time               17.03
INSTRUM            spect
PROBHD   5 mm PABBO BB/
PULPROG          zgig30
TD                16384
SOLVENT         Acetone
NS                   16
DS                    4
SWH            8012.820 Hz
FIDRES         0.489064 Hz
AQ            1.0223616 sec
RG                29.52
DW               62.400 usec
DE                 6.50 usec
TE                294.7 K
D1           1.00000000 sec
D11          0.03000000 sec
TD0                   1

======== CHANNEL f1 ========
NUC1                 1H
P1                15.00 usec
PLW1        12.50000000 W
SFO1        400.1320007 MHz

======== CHANNEL f2 ========
CPDPRG[2          garp4
NUC2                11B
PCPD2             90.00 usec
PLW2        52.96599960 W
PLW12        0.64477998 W
SFO2        128.3776050 MHz

F2 - Processing parameters
SI                32768
SF          400.1300073 MHz
WDW                  EM
SSB      0
LB                 1.00 Hz
GB       0
PC                 1.40
```

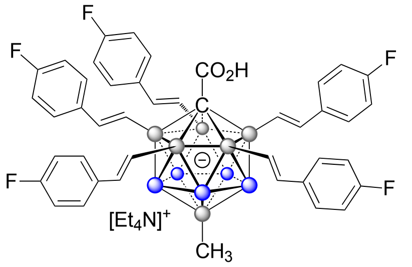

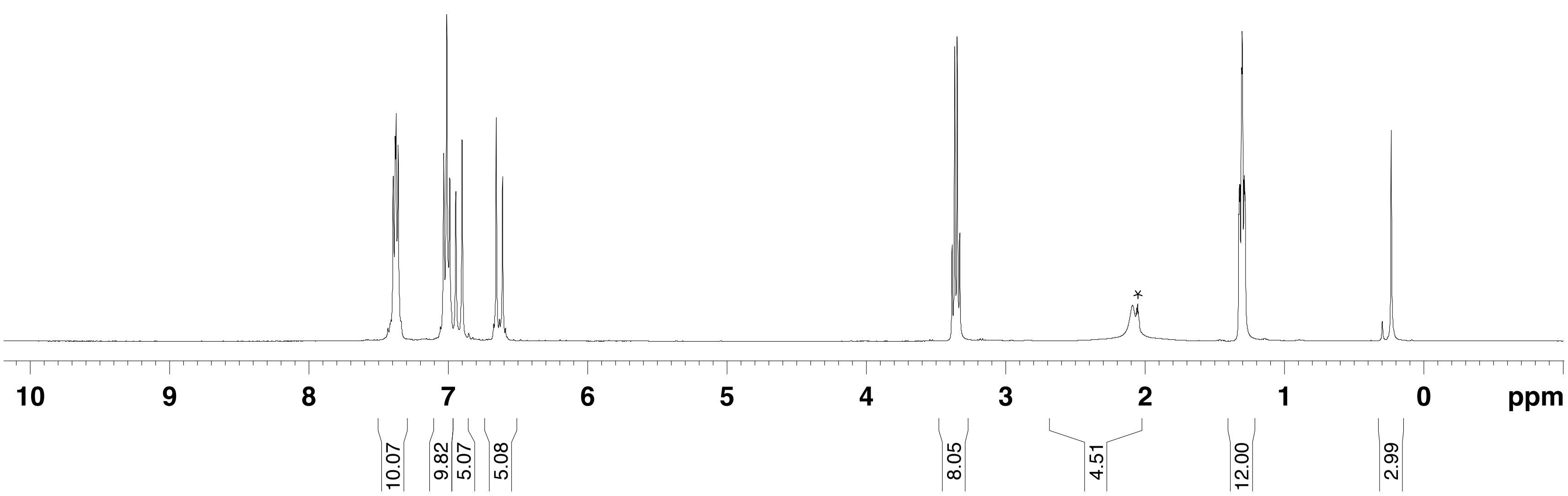

12–Me–Penta–F–styrine product 50 mg in 0.6 ml acetone–d6
11B NMR, 128 MHz, 23 C

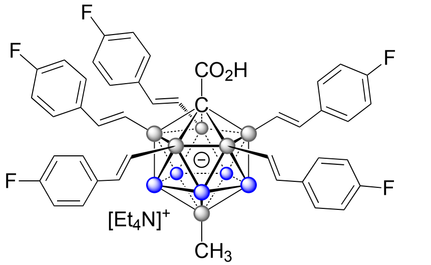

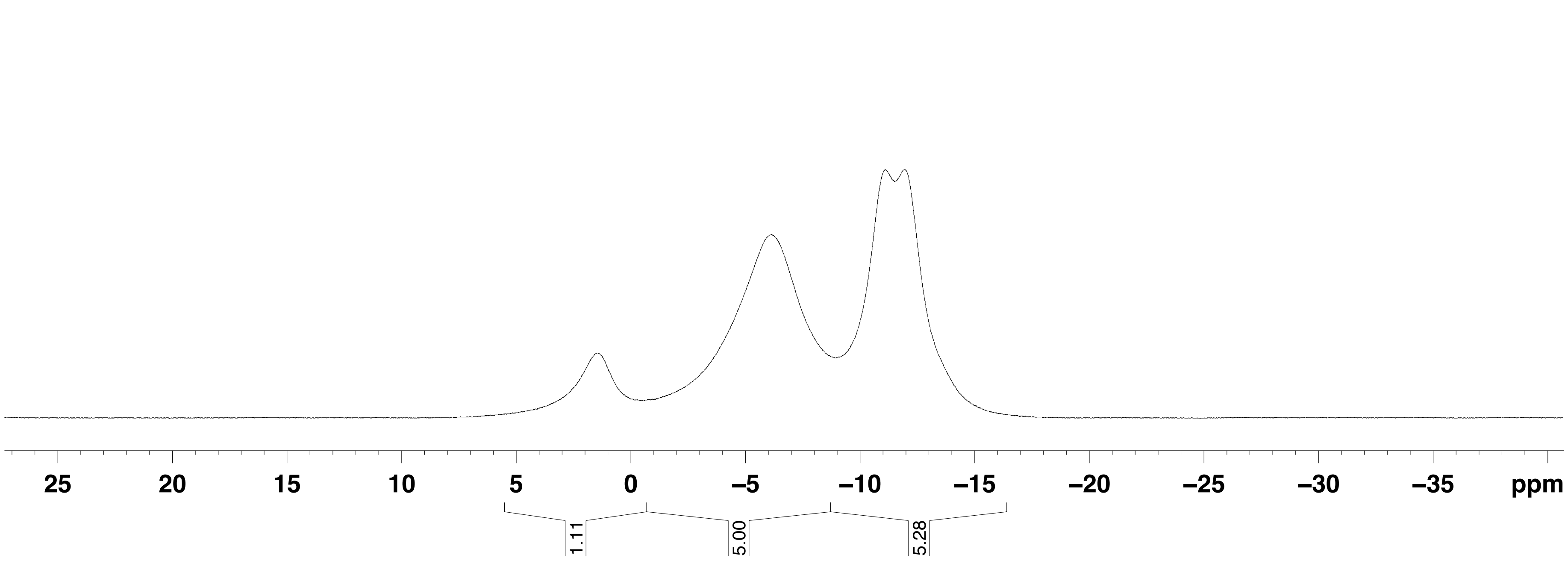


```
Current Data Parameters
NAME     12-Me-penta-4-F-styrene
EXPNO                  2
PROCNO                 1

F2 - Acquisition Parameters
Date_           20180604
Time               17.09
INSTRUM            spect
PROBHD   5 mm PABBO BB/
PULPROG               zg
TD                 65536
SOLVENT          Acetone
NS                   128
DS                     4
SWH            25510.203 Hz
FIDRES          0.389255 Hz
AQ             1.2845056 sec
RG                193.34
DW                19.600 usec
DE                  6.50 usec
TE                 293.5 K
D1            1.00000000 sec
TD0                    1

======== CHANNEL f1 ========
NUC1                 11B
P1                  9.93 usec
PLW1         52.96599960 W
SFO1        128.3776052 MHz

F2 - Processing parameters
SI                 32768
SF          128.3776050 MHz
WDW                   EM
SSB      0
LB                  2.00 Hz
GB       0
PC                  1.40
```

12–Me–Penta–F–styrine product 50 mg in 0.6 ml acetone–d6
11B{1H} NMR, 128 MHz, 23 C

```
Current Data Parameters
NAME       12-Me-penta-4-F-styrene
EXPNO                    3
PROCNO                   1

F2 - Acquisition Parameters
Date_             20180604
Time                 17.15
INSTRUM              spect
PROBHD     5 mm PABBO BB/
PULPROG             zgpg30
TD                   65536
SOLVENT            Acetone
NS                     128
DS                       4
SWH              25510.203 Hz
FIDRES            0.389255 Hz
AQ               1.2845056 sec
RG                  193.34
DW                  19.600 usec
DE                    6.50 usec
TE                   294.6 K
D1              1.00000000 sec
D11             0.03000000 sec
TD0                      1

======== CHANNEL f1 ========
NUC1                   11B
P1                    9.93 usec
PLW1           52.96599960 W
SFO1           128.3776050 MHz

======== CHANNEL f2 ========
CPDPRG[2           waltz16
NUC2                    1H
PCPD2                80.00 usec
PLW2           12.50000000 W
PLW12           0.43945000 W
PLW13           0.28125000 W
SFO2           400.1320007 MHz

F2 - Processing parameters
SI                   32768
SF             128.3776050 MHz
WDW                     EM
SSB      0
LB                    3.00 Hz
GB       0
PC                    1.40
```

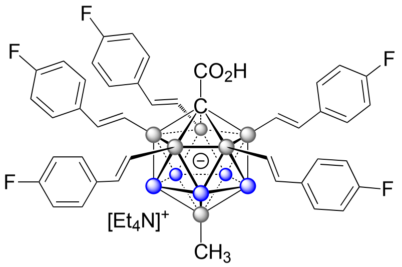

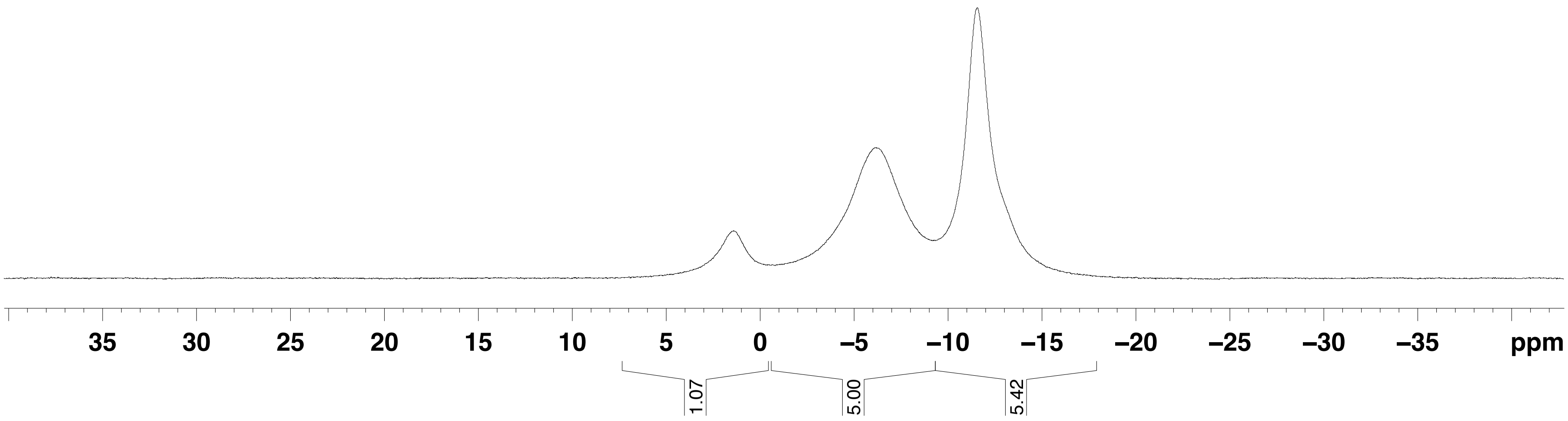

12–Me–Penta–F–styrine product 50 mg in 0.6 ml acetone–d6
13C{1H} NMR, 101 MHz, 23 C

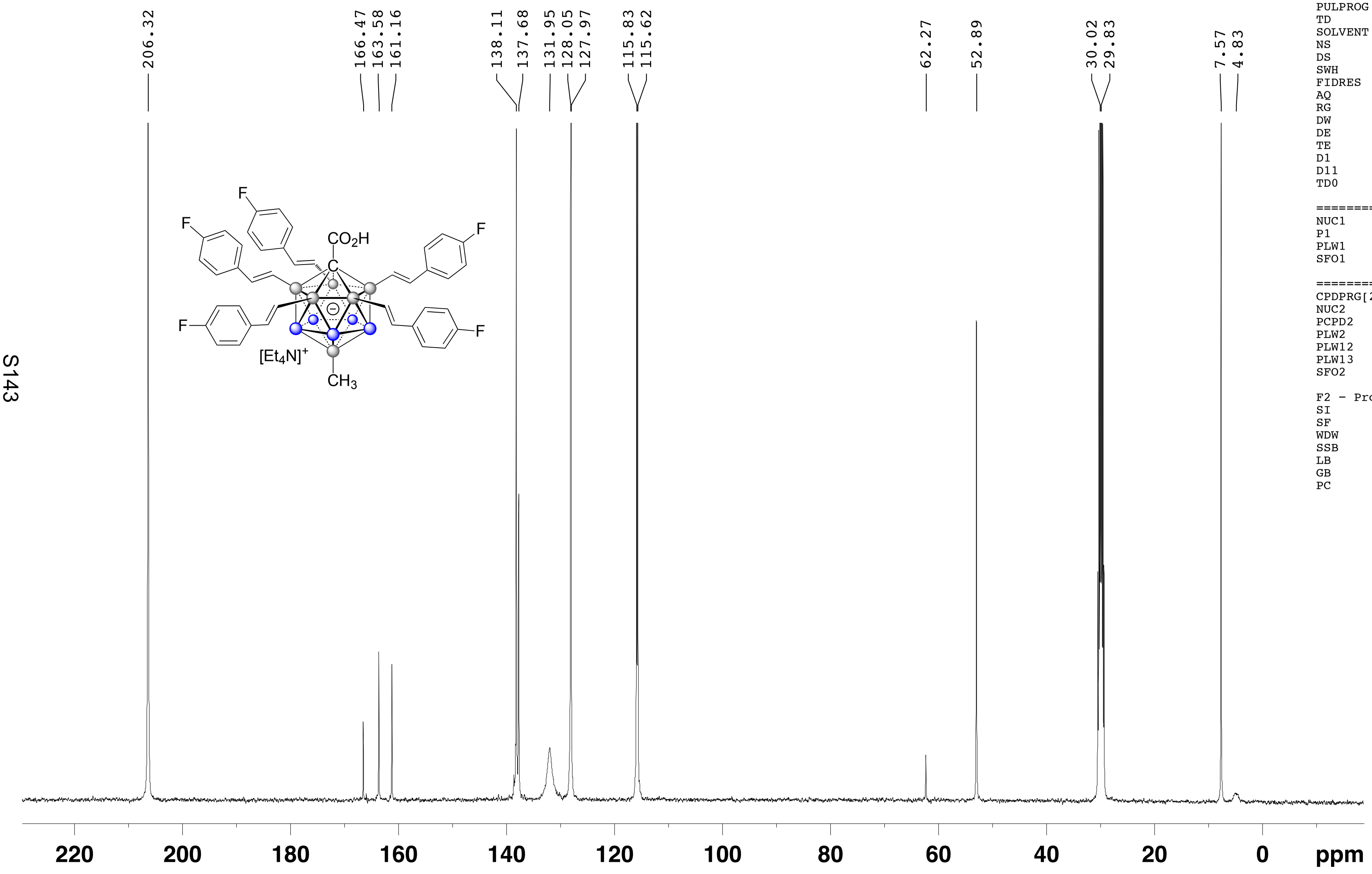


```
Current Data Parameters
NAME     12-Me-penta-4-F-styrene
EXPNO                  4
PROCNO                 1

F2 - Acquisition Parameters
Date_           20180604
Time               18.47
INSTRUM            spect
PROBHD   5 mm PABBO BB/
PULPROG           zgpg30
TD                 65536
SOLVENT          Acetone
NS                  2048
DS                     4
SWH            29761.904 Hz
FIDRES          0.454131 Hz
AQ             1.1010048 sec
RG                193.34
DW                16.800 usec
DE                  6.50 usec
TE                 294.9 K
D1            1.50000000 sec
D11           0.03000000 sec
TD0                    1

======== CHANNEL f1 ========
NUC1                 13C
P1                 10.00 usec
PLW1         53.00000000 W
SFO1        100.6228293 MHz

======== CHANNEL f2 ========
CPDPRG[2         waltz16
NUC2                  1H
PCPD2              80.00 usec
PLW2         12.50000000 W
PLW12         0.43945000 W
PLW13         0.28125000 W
SFO2        400.1316005 MHz

F2 - Processing parameters
SI                 32768
SF          100.6126899 MHz
WDW                   EM
SSB      0
LB                  5.00 Hz
GB       0
PC                  1.40
```

12–Ph–Penta–styrine product 50 mg in 0.6 ml acetone–d6*
1H{11B} NMR, 400 MHz, 23 C

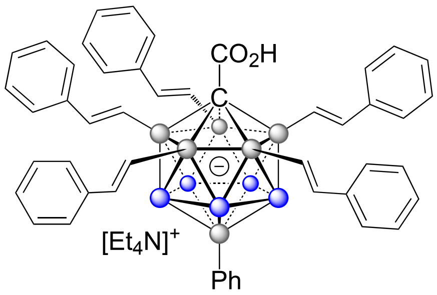

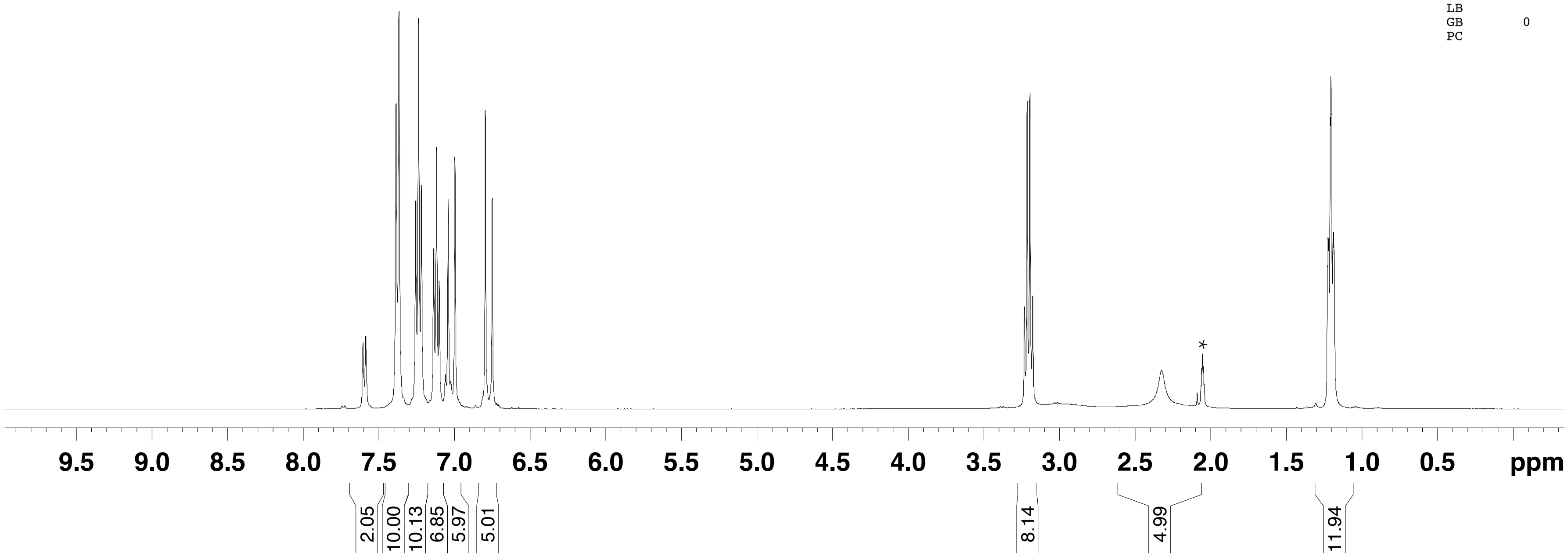


```
Current Data Parameters
NAME      12-Ph-penta-styrene
EXPNO                    1
PROCNO                   1

F2 - Acquisition Parameters
Date_           20180603
Time               14.13
INSTRUM            spect
PROBHD    5 mm PABBO BB/
PULPROG          zgig30
TD                16384
SOLVENT          Acetone
NS                   16
DS                    4
SWH            8012.820 Hz
FIDRES         0.489064 Hz
AQ            1.0223616 sec
RG                55.74
DW               62.400 usec
DE                 6.50 usec
TE                293.7 K
D1           1.00000000 sec
D11          0.03000000 sec
TD0                   1

======== CHANNEL f1 ========
NUC1                 1H
P1                15.00 usec
PLW1        12.50000000 W
SFO1        400.1320007 MHz

======== CHANNEL f2 ========
CPDPRG[2          garp4
NUC2                11B
PCPD2             90.00 usec
PLW2        52.96599960 W
PLW12        0.64477998 W
SFO2        128.3776050 MHz

F2 - Processing parameters
SI                32768
SF          400.1300072 MHz
WDW                  EM
SSB      0
LB                 1.00 Hz
GB       0
PC                 1.40
```

12–Ph–Penta–styrine product 50 mg in 0.6 ml acetone–d6
11B NMR, 128 MHz, 23 C

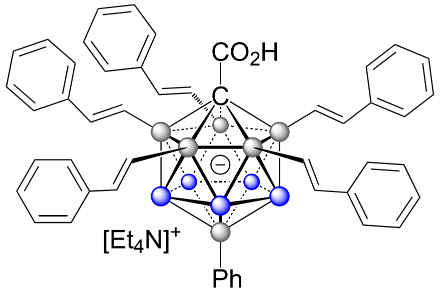

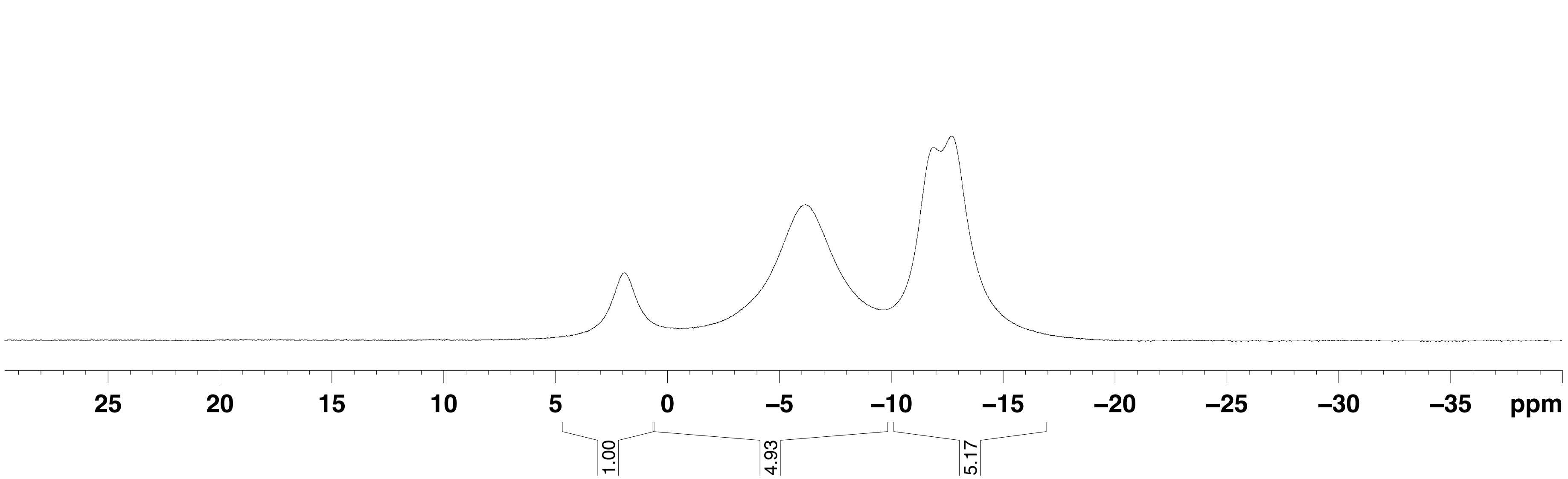


```
Current Data Parameters
NAME     12-Ph-penta-styrene
EXPNO                  2
PROCNO                 1

F2 - Acquisition Parameters
Date_           20180603
Time               14.19
INSTRUM            spect
PROBHD   5 mm PABBO BB/
PULPROG               zg
TD                 65536
SOLVENT          Acetone
NS                   128
DS                     4
SWH            25510.203 Hz
FIDRES          0.389255 Hz
AQ             1.2845056 sec
RG                193.34
DW                19.600 usec
DE                  6.50 usec
TE                 293.6 K
D1            1.00000000 sec
TD0                    1

======== CHANNEL f1 ========
NUC1                 11B
P1                  9.93 usec
PLW1         52.96599960 W
SFO1         128.3776052 MHz

F2 - Processing parameters
SI                 32768
SF           128.3776050 MHz
WDW                   EM
SSB      0
LB                  3.00 Hz
GB       0
PC                  1.40
```

12–Ph–Penta–styrine product 50 mg in 0.6 ml acetone–d6
11B{1H} NMR, 128 MHz, 23 C

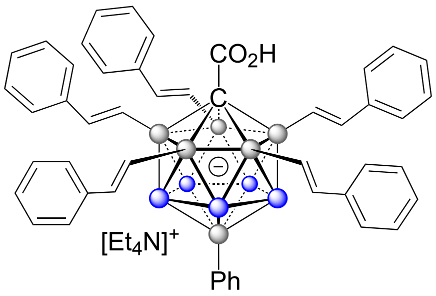

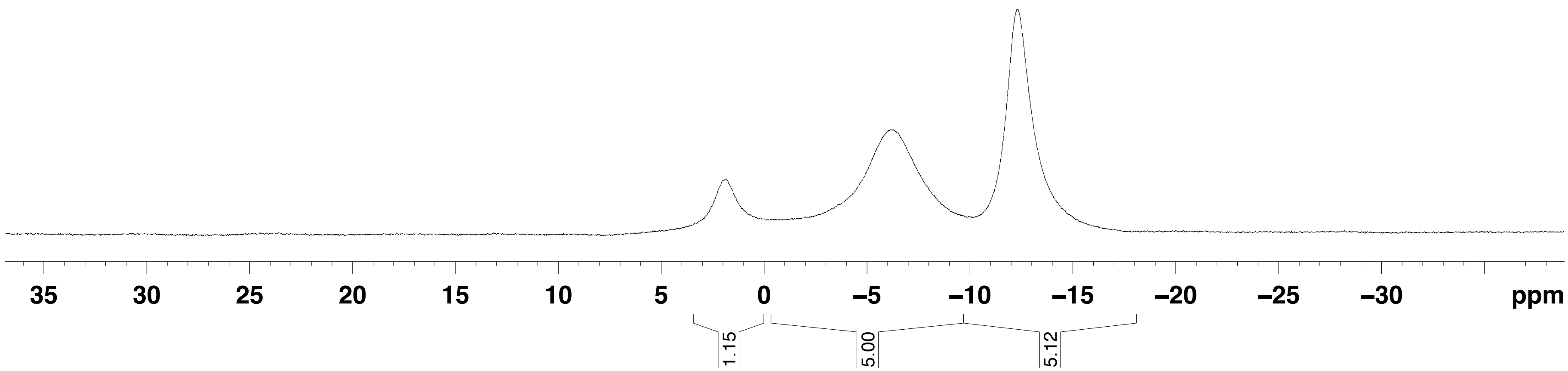


```
Current Data Parameters
NAME     12-Ph-penta-styrene
EXPNO                  3
PROCNO                 1

F2 - Acquisition Parameters
Date_           20180603
Time               14.25
INSTRUM            spect
PROBHD   5 mm PABBO BB/
PULPROG          zgpg30
TD                 65536
SOLVENT          Acetone
NS                   128
DS                     4
SWH            25510.203 Hz
FIDRES          0.389255 Hz
AQ             1.2845056 sec
RG                193.34
DW                19.600 usec
DE                  6.50 usec
TE                 294.8 K
D1            1.00000000 sec
D11           0.03000000 sec
TD0                    1

======== CHANNEL f1 ========
NUC1                 11B
P1                  9.93 usec
PLW1         52.96599960 W
SFO1        128.3776050 MHz

======== CHANNEL f2 ========
CPDPRG[2         waltz16
NUC2                  1H
PCPD2              80.00 usec
PLW2         12.50000000 W
PLW12         0.43945000 W
PLW13         0.28125000 W
SFO2        400.1320007 MHz

F2 - Processing parameters
SI                 32768
SF          128.3776050 MHz
WDW                   EM
SSB      0
LB                  3.00 Hz
GB       0
PC                  1.40
```

12–Ph–Penta–styrine product 50 mg in 0.6 ml acetone–d6*
13C{1H} NMR, 101 MHz, 23

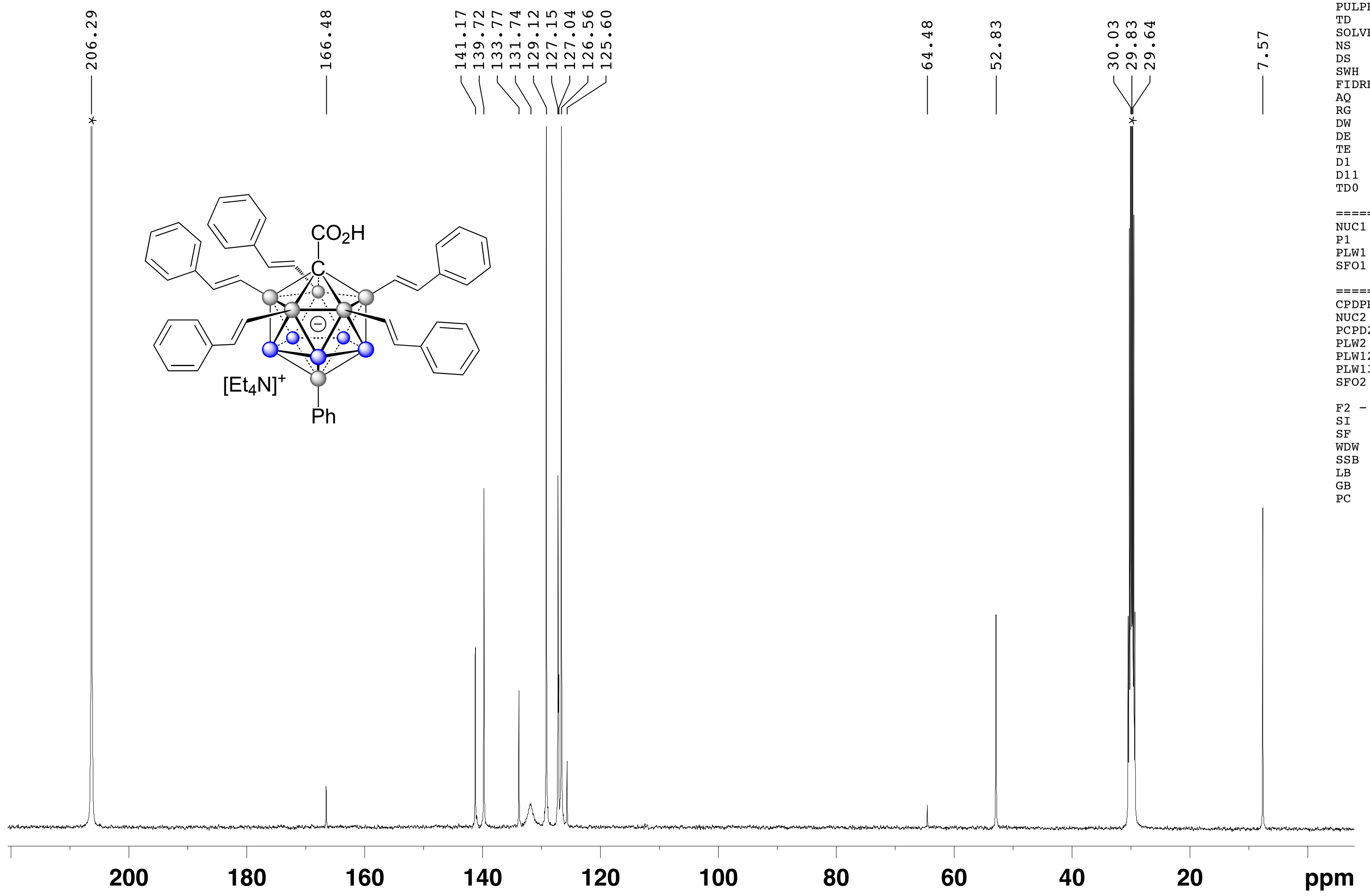


```
Current Data Parameters
NAME     12-Ph-penta-styrene
EXPNO                  4
PROCNO                 1

F2 - Acquisition Parameters
Date_           20180603
Time               15.58
INSTRUM            spect
PROBHD   5 mm PABBO BB/
PULPROG          zgpg30
TD                65536
SOLVENT          Acetone
NS                 2048
DS                    4
SWH          29761.904 Hz
FIDRES        0.454131 Hz
AQ           1.1010048 sec
RG               193.34
DW               16.800 usec
DE                 6.50 usec
TE                294.9 K
D1          1.50000000 sec
D11         0.03000000 sec
TD0                   1

======== CHANNEL f1 ========
NUC1                 13C
P1                 10.00 usec
PLW1         53.00000000 W
SFO1         100.6228293 MHz

======== CHANNEL f2 ========
CPDPRG[2         waltz16
NUC2                  1H
PCPD2              80.00 usec
PLW2         12.50000000 W
PLW12         0.43945000 W
PLW13         0.28125000 W
SFO2         400.1316005 MHz

F2 - Processing parameters
SI                 32768
SF           100.6126872 MHz
WDW                   EM
SSB      0
LB                  5.00 Hz
GB       0
PC                  1.40
```

12–Ph–Penta–F–styrene product 50 mg in 0.6 ml acetone–d6*
1H{11B} NMR, 400 MHz, 23 C

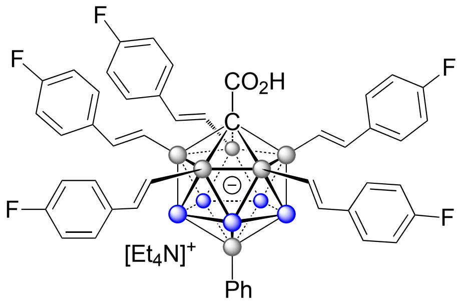

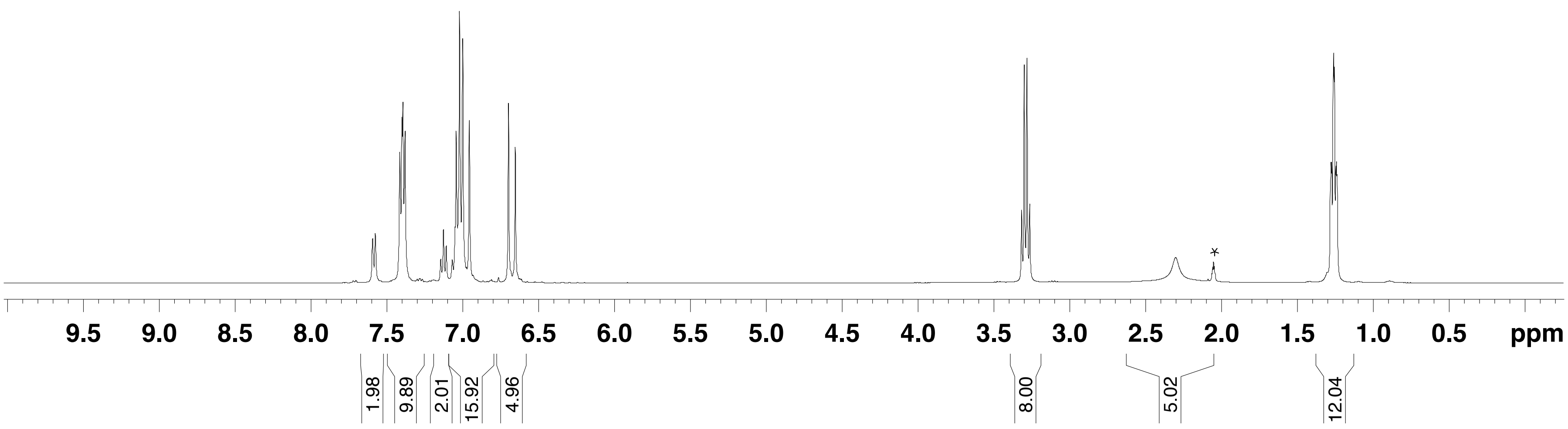


```
Current Data Parameters
NAME     12-Ph-penta-4-F-styrene
EXPNO                 1
PROCNO                1

F2 - Acquisition Parameters
Date_          20180610
Time               5.28
INSTRUM           spect
PROBHD   5 mm PABBO BB/
PULPROG          zgig30
TD                16384
SOLVENT         Acetone
NS                   16
DS                    4
SWH            8012.820 Hz
FIDRES         0.489064 Hz
AQ            1.0223616 sec
RG                52.99
DW               62.400 usec
DE                 6.50 usec
TE                293.7 K
D1           1.00000000 sec
D11          0.03000000 sec
TD0                   1

======== CHANNEL f1 ========
NUC1                 1H
P1                15.00 usec
PLW1        12.50000000 W
SFO1        400.1320007 MHz

======== CHANNEL f2 ========
CPDPRG[2          garp4
NUC2                11B
PCPD2             90.00 usec
PLW2        52.96599960 W
PLW12        0.64477998 W
SFO2        128.3776050 MHz

F2 - Processing parameters
SI                32768
SF          400.1300071 MHz
WDW                  EM
SSB      0
LB                 1.00 Hz
GB       0
PC                 1.40
```

12–Ph–Penta–F–styrene product 50 mg in 0.6 ml acetone–d6
11B NMR, 128 MHz, 23 C

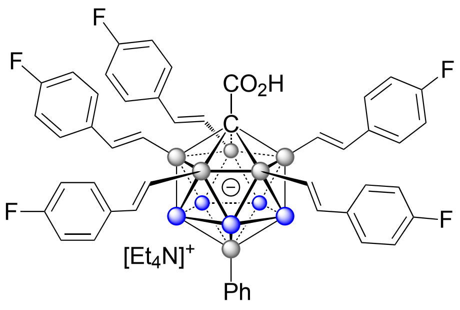

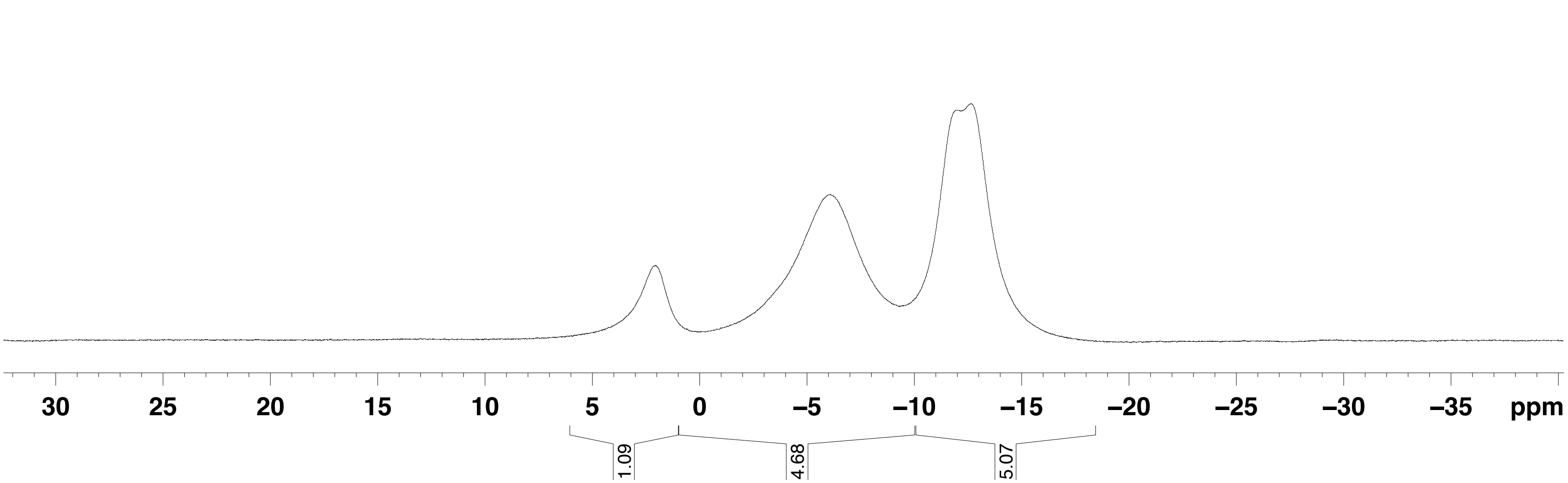


```
Current Data Parameters
NAME     12-Ph-penta-4-F-styrene
EXPNO                  2
PROCNO                 1

F2 - Acquisition Parameters
Date_           20180610
Time                5.34
INSTRUM            spect
PROBHD   5 mm PABBO BB/
PULPROG               zg
TD                 65536
SOLVENT          Acetone
NS                   128
DS                     4
SWH            25510.203 Hz
FIDRES          0.389255 Hz
AQ             1.2845056 sec
RG                193.34
DW                19.600 usec
DE                  6.50 usec
TE                 293.4 K
D1            1.00000000 sec
TD0                    1

======== CHANNEL f1 ========
NUC1                 11B
P1                  9.93 usec
PLW1         52.96599960 W
SFO1         128.3776052 MHz

F2 - Processing parameters
SI                 32768
SF           128.3776050 MHz
WDW                   EM
SSB      0
LB                  2.00 Hz
GB       0
PC                  1.40
```

12–Ph–Penta–F–styrene product 50 mg in 0.6 ml acetone–d6
11B{1H} NMR, 128 MHz, 23 C

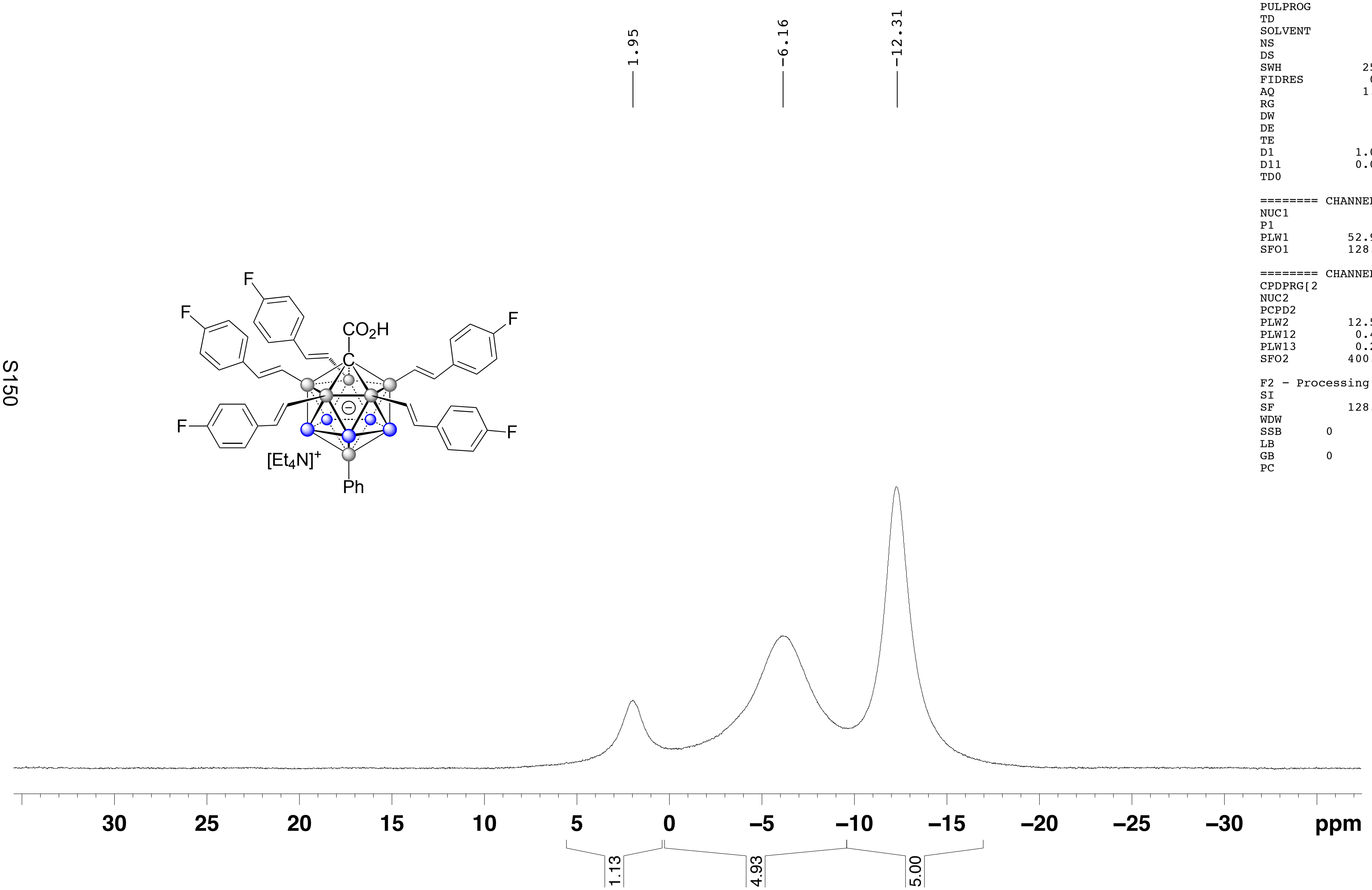


```
Current Data Parameters
NAME     12-Ph-penta-4-F-styrene
EXPNO                   3
PROCNO                  1

F2 - Acquisition Parameters
Date_           20180610
Time                5.40
INSTRUM            spect
PROBHD   5 mm PABBO BB/
PULPROG           zgpg30
TD                 65536
SOLVENT          Acetone
NS                   128
DS                     4
SWH            25510.203 Hz
FIDRES          0.389255 Hz
AQ             1.2845056 sec
RG                193.34
DW                19.600 usec
DE                  6.50 usec
TE                 294.6 K
D1            1.00000000 sec
D11           0.03000000 sec
TD0                    1

======== CHANNEL f1 ========
NUC1                 11B
P1                  9.93 usec
PLW1         52.96599960 W
SFO1         128.3776050 MHz

======== CHANNEL f2 ========
CPDPRG[2         waltz16
NUC2                  1H
PCPD2              80.00 usec
PLW2         12.50000000 W
PLW12         0.43945000 W
PLW13         0.28125000 W
SFO2         400.1320007 MHz

F2 - Processing parameters
SI                 32768
SF           128.3776050 MHz
WDW                   EM
SSB      0
LB                  3.00 Hz
GB       0
PC                  1.40
```

12–Ph–Penta–F–styrene product 50 mg in 0.6 ml acetone–d6*
13C{1H} NMR, 101 MHz, 23 C

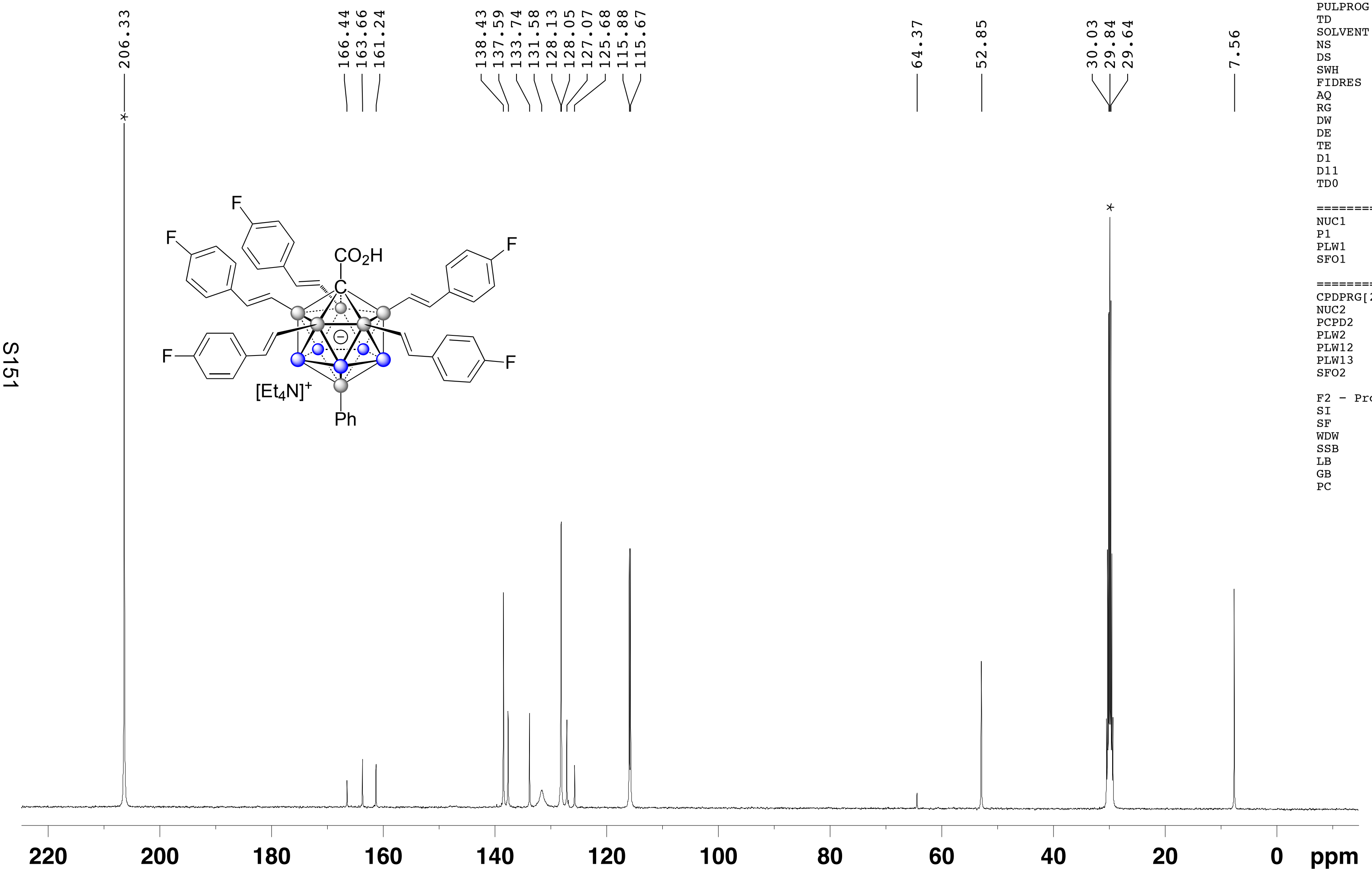


```
Current Data Parameters
NAME     12-Ph-penta-4-F-styrene
EXPNO                  4
PROCNO                 1

F2 - Acquisition Parameters
Date_           20180610
Time                7.12
INSTRUM            spect
PROBHD   5 mm PABBO BB/
PULPROG          zgpg30
TD                 65536
SOLVENT          Acetone
NS                  2048
DS                     4
SWH           29761.904 Hz
FIDRES         0.454131 Hz
AQ            1.1010048 sec
RG                193.34
DW                16.800 usec
DE                  6.50 usec
TE                 294.1 K
D1           1.50000000 sec
D11          0.03000000 sec
TD0                    1

======== CHANNEL f1 ========
NUC1                 13C
P1                 10.00 usec
PLW1         53.00000000 W
SFO1        100.6228293 MHz

======== CHANNEL f2 ========
CPDPRG[2         waltz16
NUC2                  1H
PCPD2              80.00 usec
PLW2         12.50000000 W
PLW12         0.43945000 W
PLW13         0.28125000 W
SFO2        400.1316005 MHz

F2 - Processing parameters
SI                 32768
SF          100.6126881 MHz
WDW                   EM
SSB      0
LB                  5.00 Hz
GB       0
PC                  1.40
```

12–CN–Penta–F–styrene product 40 mg in 0.6 ml acetone–d6*
1H{11B} NMR, 400 MHz, 23 C

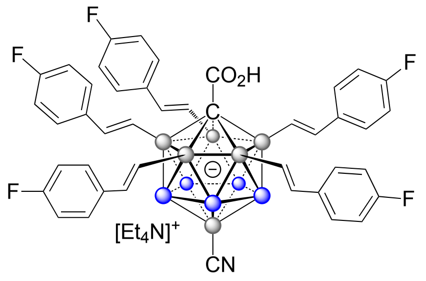

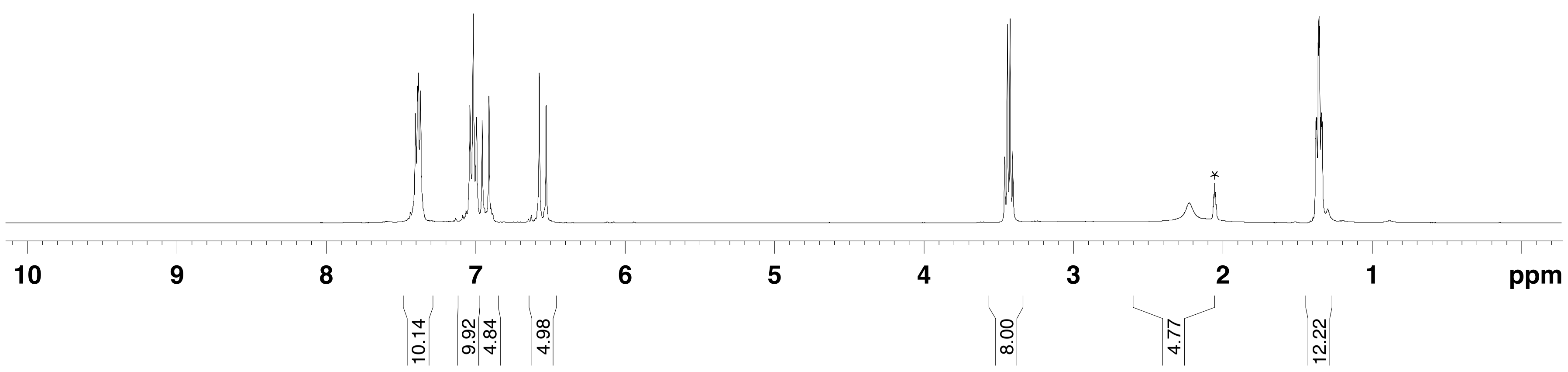


```
Current Data Parameters
NAME        12-CN-penta-4-F-styrene
EXPNO                     1
PROCNO                    1

F2 - Acquisition Parameters
Date_              20180528
Time                  14.40
INSTRUM               spect
PROBHD   5 mm PABBO BB/
PULPROG             zgig30
TD                    16384
SOLVENT             Acetone
NS                       16
DS                        4
SWH                8012.820 Hz
FIDRES             0.489064 Hz
AQ                1.0223616 sec
RG                    78.69
DW                   62.400 usec
DE                     6.50 usec
TE                    294.5 K
D1               1.00000000 sec
D11              0.03000000 sec
TD0                       1

======== CHANNEL f1 ========
NUC1                     1H
P1                    15.00 usec
PLW1            12.50000000 W
SFO1            400.1320007 MHz

======== CHANNEL f2 ========
CPDPRG[2              garp4
NUC2                    11B
PCPD2                 90.00 usec
PLW2            52.96599960 W
PLW12            0.64477998 W
SFO2            128.3776050 MHz

F2 - Processing parameters
SI                    32768
SF              400.1300072 MHz
WDW                      EM
SSB      0
LB                     1.00 Hz
GB       0
PC                     1.40
```

12–CN–Penta–F–styrene product 30 mg in 0.6 ml acetone–d6
11B NMR, 160 MHz, 23 C

```
Current Data Parameters
NAME     12-CN-Penta-F-styrine-new
EXPNO                    2
PROCNO                   1

F2 - Acquisition Parameters
Date_          20181224
Time              17.18
INSTRUM           spect
PROBHD   5 mm PABBO BB-
PULPROG              zg
TD                32048
SOLVENT         Acetone
NS                   64
DS                    0
SWH           32051.281 Hz
FIDRES         1.000102 Hz
AQ            0.4999488 sec
RG                  203
DW               15.600 usec
DE                16.00 usec
TE                294.9 K
D1           0.50000000 sec

======== CHANNEL f1 ========
NUC1                 11B
P1                 10.00 usec
PLW1         75.00000000 W
SFO1        160.4615792 MHz

F2 - Processing parameters
SI                32768
SF          160.4615993 MHz
WDW                  EM
SSB      0
LB                10.00 Hz
GB       0
PC                 1.40
```

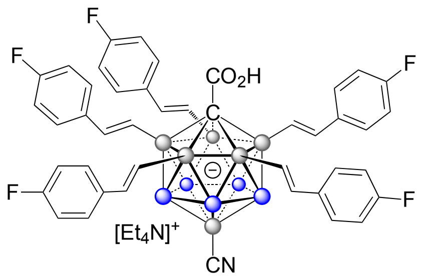

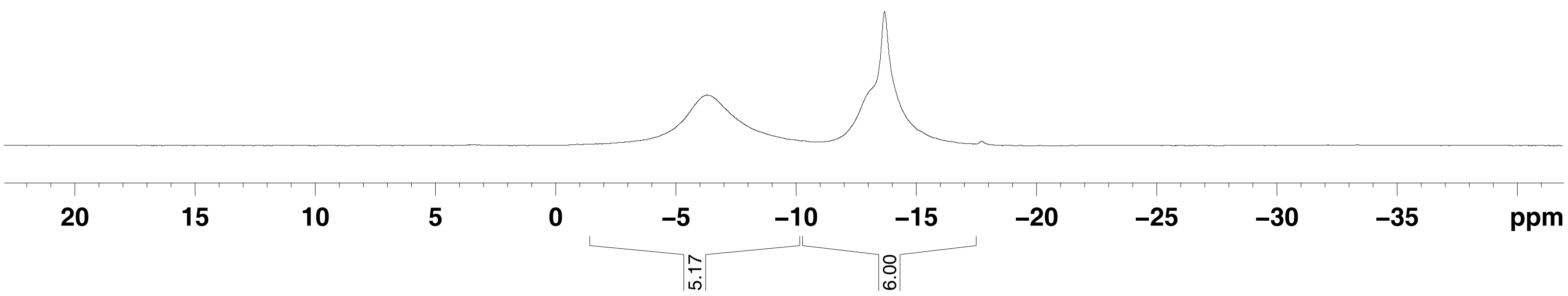

12–CN–Penta–F–styrene product 30 mg in 0.6 ml acetone–d6
11B{1H} NMR, 160 MHz, 23 C

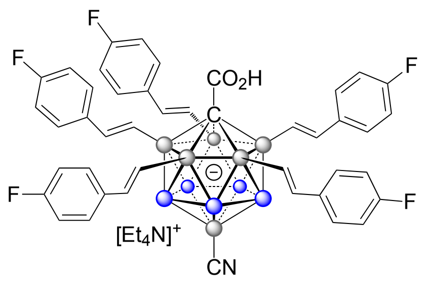

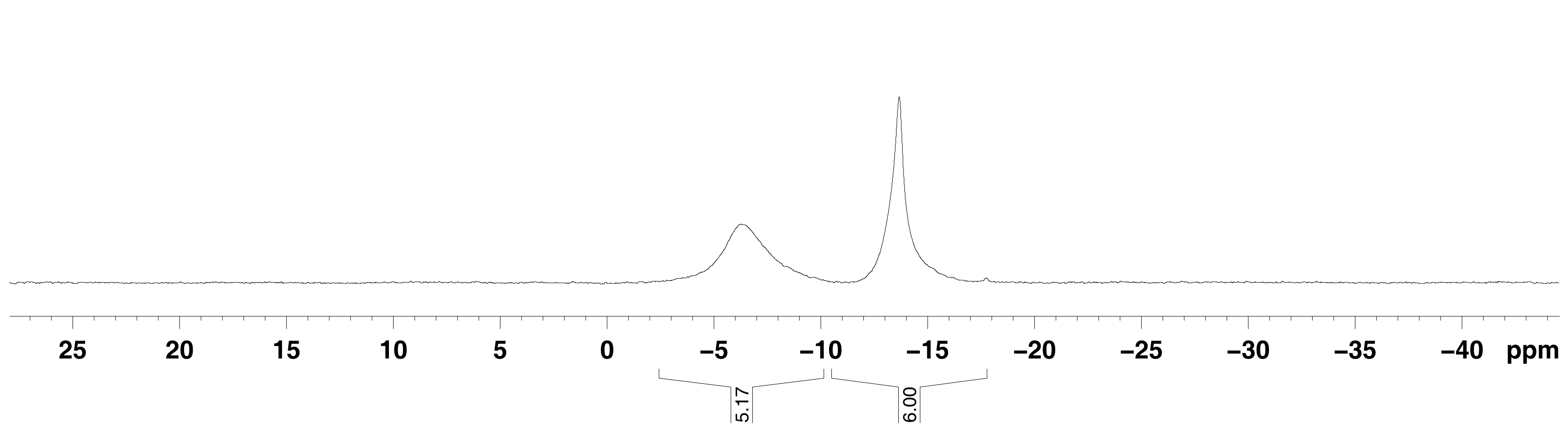

Current Data Parameters

NAME	12-CN-Penta-F-styrine-new
EXPNO	3
PROCNO	1

F2 - Acquisition Parameters

Date_	20181224
Time	17.20
INSTRUM	spect
PROBHD	5 mm PABBO BB-
PULPROG	zgpg30
TD	32048
SOLVENT	Acetone
NS	64
DS	0
SWH	32051.281 Hz
FIDRES	1.000102 Hz
AQ	0.4999488 sec
RG	203
DW	15.600 usec
DE	6.50 usec
TE	295.3 K
D1	0.50000000 sec
D11	0.03000000 sec

======== CHANNEL f1 ========

NUC1	11B
P1	10.00 usec
PLW1	75.00000000 W
SFO1	160.4615792 MHz

======== CHANNEL f2 ========

CPDPRG[2	waltz16
NUC2	1H
PCPD2	80.00 usec
PLW2	19.00000000 W
PLW12	0.42750001 W
PLW13	0.27360001 W
SFO2	500.1330885 MHz

F2 - Processing parameters

SI	32768
SF	160.4615993 MHz
WDW	EM
SSB	0
LB	10.00 Hz
GB	0
PC	1.40

12–CN–Penta–F–styrene product 40 mg in 0.6 ml acetone–d6*
13C{1H} NMR, 101 MHz, 23 C

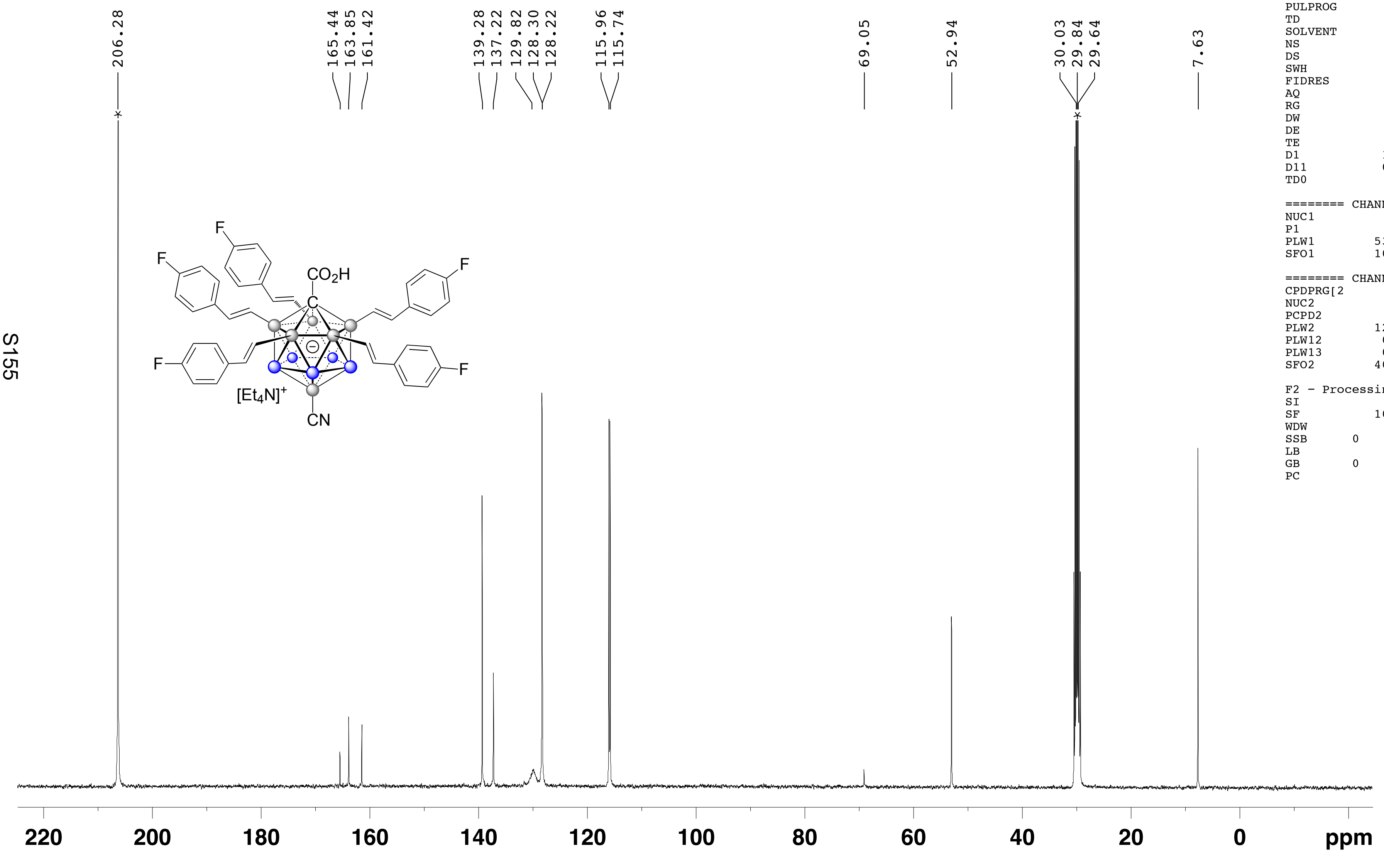


```
Current Data Parameters
NAME     12-CN-penta-4-F-styrene
EXPNO                    4
PROCNO                   1

F2 - Acquisition Parameters
Date_             20180528
Time                 16.25
INSTRUM              spect
PROBHD   5 mm PABBO BB/
PULPROG             zgpg30
TD                   65536
SOLVENT            Acetone
NS                    2048
DS                       4
SWH              29761.904 Hz
FIDRES            0.454131 Hz
AQ               1.1010048 sec
RG                  193.34
DW                  16.800 usec
DE                    6.50 usec
TE                   294.2 K
D1              1.50000000 sec
D11             0.03000000 sec
TD0                      1

======== CHANNEL f1 ========
NUC1                   13C
P1                   10.00 usec
PLW1           53.00000000 W
SFO1          100.6228293 MHz

======== CHANNEL f2 ========
CPDPRG[2           waltz16
NUC2                    1H
PCPD2                80.00 usec
PLW2           12.50000000 W
PLW12           0.43945000 W
PLW13           0.28125000 W
SFO2          400.1316005 MHz

F2 - Processing parameters
SI                   32768
SF            100.6126846 MHz
WDW                     EM
SSB      0
LB                    3.00 Hz
GB       0
PC                    1.40
```

Penta–R–ether product 60 mg in 0.6 ml acetone–d6*
1H{11B} NMR, 400 MHz, 23 C

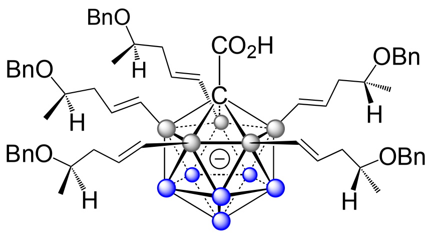

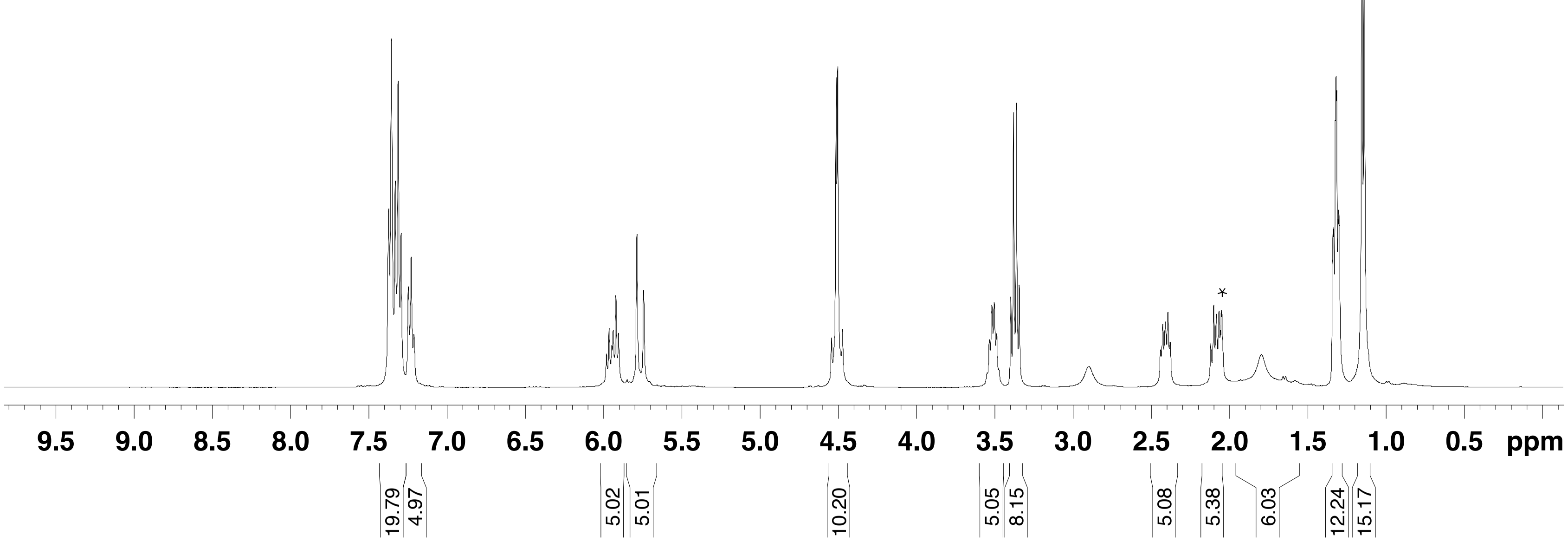


```
Current Data Parameters
NAME      penta-R-penten-ol
EXPNO                    1
PROCNO                   1

F2 - Acquisition Parameters
Date_             20180628
Time                 15.53
INSTRUM              spect
PROBHD    5 mm PABBO BB/
PULPROG            zgig30
TD                  16384
SOLVENT           Acetone
NS                     16
DS                      4
SWH              8012.820 Hz
FIDRES           0.489064 Hz
AQ              1.0223616 sec
RG                  29.52
DW                 62.400 usec
DE                   6.50 usec
TE                  413.7 K
D1             1.00000000 sec
D11            0.03000000 sec
TD0                     1

======== CHANNEL f1 ========
NUC1                   1H
P1                  15.00 usec
PLW1          12.50000000 W
SFO1          400.1320007 MHz

======== CHANNEL f2 ========
CPDPRG[2            garp4
NUC2                  11B
PCPD2               90.00 usec
PLW2          52.96599960 W
PLW12          0.64477998 W
SFO2          128.3776050 MHz

F2 - Processing parameters
SI                  32768
SF            400.1300071 MHz
WDW                    EM
SSB      0
LB                   1.00 Hz
GB       0
PC                   1.40
```

Penta–R–ether product 60 mg in 0.6 ml acetone–d6
11B NMR, 128 MHz, 23 C

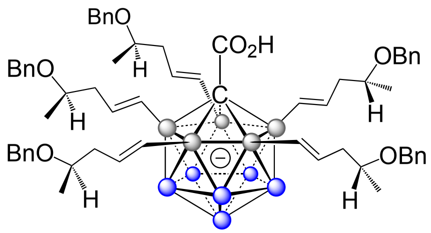

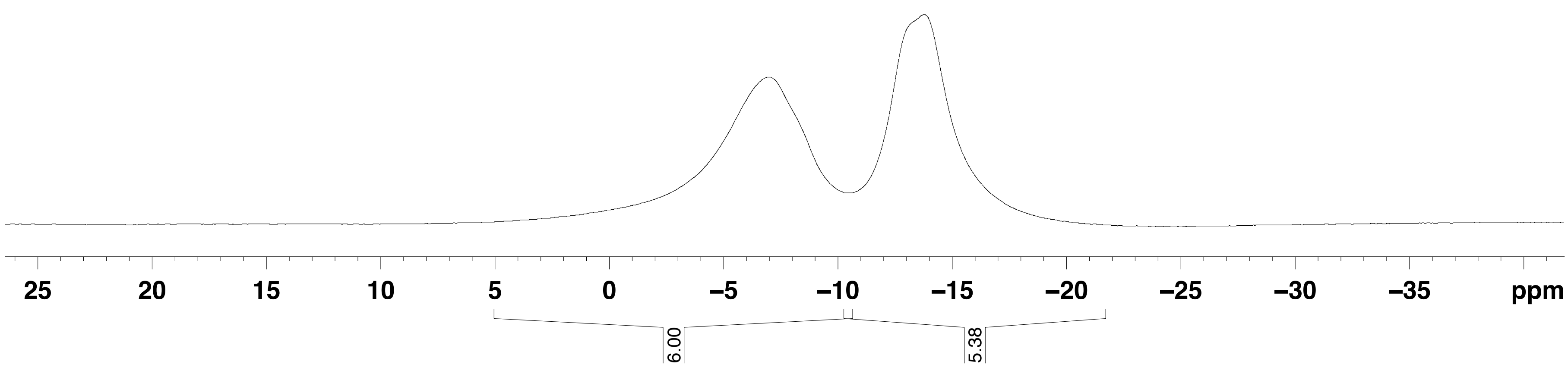


```
Current Data Parameters
NAME      penta-R-penten-ol
EXPNO                     2
PROCNO                    1

F2 - Acquisition Parameters
Date_              20180628
Time                  15.58
INSTRUM               spect
PROBHD    5 mm PABBO BB/
PULPROG                  zg
TD                    65536
SOLVENT             Acetone
NS                      128
DS                        4
SWH               25510.203 Hz
FIDRES             0.389255 Hz
AQ                1.2845056 sec
RG                   193.34
DW                   19.600 usec
DE                     6.50 usec
TE                    416.6 K
D1               1.00000000 sec
TD0                       1

======== CHANNEL f1 ========
NUC1                    11B
P1                     9.93 usec
PLW1            52.96599960 W
SFO1            128.3776052 MHz

F2 - Processing parameters
SI                    32768
SF              128.3776050 MHz
WDW                      EM
SSB       0
LB                    15.00 Hz
GB        0
PC                     1.40
```

Penta–R–ether product 60 mg in 0.6 ml acetone–d6
11B{1H} NMR, 128 MHz, 23 C

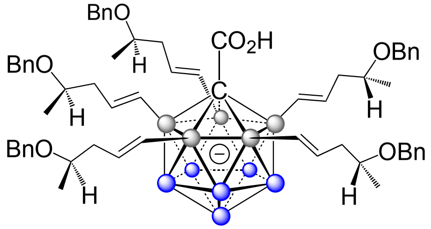

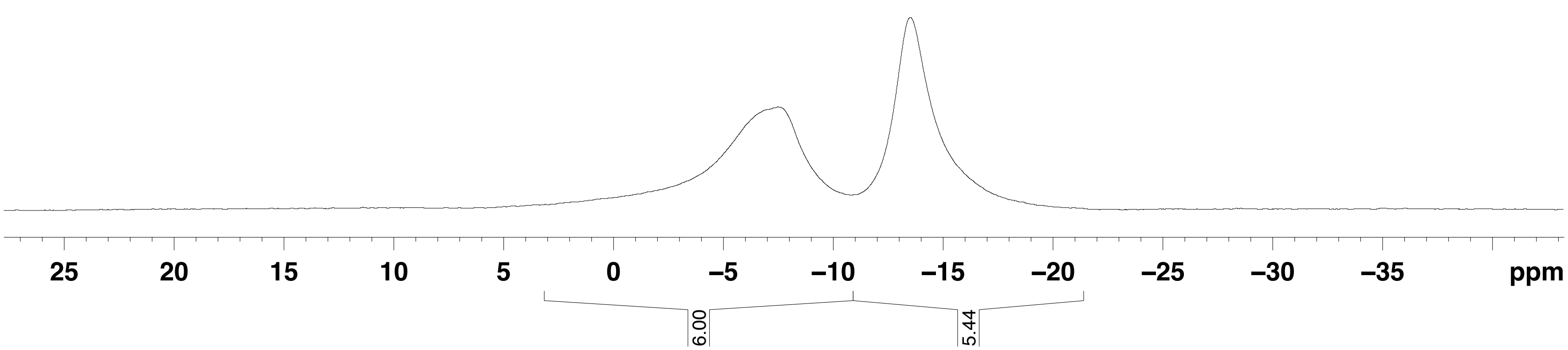


```
Current Data Parameters
NAME     penta-R-penten-ol
EXPNO                    3
PROCNO                   1

F2 - Acquisition Parameters
Date_             20180628
Time                 16.05
INSTRUM              spect
PROBHD   5 mm PABBO BB/
PULPROG            zgpg30
TD                   65536
SOLVENT            Acetone
NS                     128
DS                       4
SWH              25510.203 Hz
FIDRES            0.389255 Hz
AQ               1.2845056 sec
RG                  193.34
DW                  19.600 usec
DE                    6.50 usec
TE                   417.8 K
D1              1.00000000 sec
D11             0.03000000 sec
TD0                      1

======== CHANNEL f1 ========
NUC1                   11B
P1                    9.93 usec
PLW1           52.96599960 W
SFO1           128.3776050 MHz

======== CHANNEL f2 ========
CPDPRG[2           waltz16
NUC2                    1H
PCPD2                80.00 usec
PLW2           12.50000000 W
PLW12           0.43945000 W
PLW13           0.28125000 W
SFO2           400.1320007 MHz

F2 - Processing parameters
SI                   32768
SF             128.3776050 MHz
WDW                     EM
SSB      0
LB                   15.00 Hz
GB       0
PC                    1.40
```

Penta–R–ether product 60 mg in 0.6 ml acetone–d6*
13C{1H} NMR, 101 MHz, 23 C

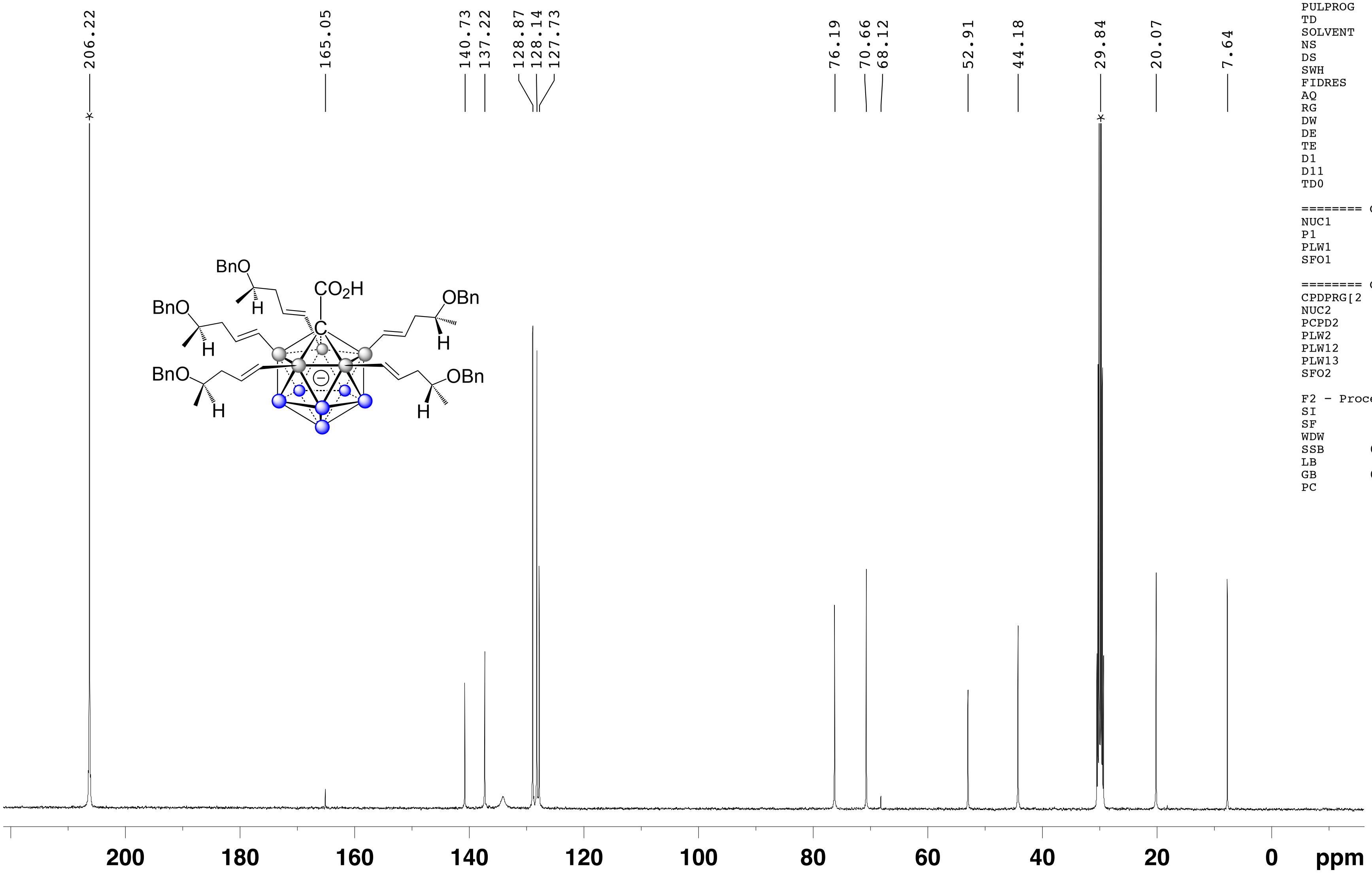


```
Current Data Parameters
NAME      penta-R-penten-ol
EXPNO                     4
PROCNO                    1

F2 - Acquisition Parameters
Date_              20180628
Time                  17.37
INSTRUM               spect
PROBHD    5 mm PABBO BB/
PULPROG              zgpg30
TD                    65536
SOLVENT             Acetone
NS                     2048
DS                        4
SWH               29761.904 Hz
FIDRES             0.454131 Hz
AQ                1.1010048 sec
RG                   193.34
DW                   16.800 usec
DE                     6.50 usec
TE                    416.8 K
D1               1.50000000 sec
D11              0.03000000 sec
TD0                       1

======== CHANNEL f1 ========
NUC1                    13C
P1                    10.00 usec
PLW1            53.00000000 W
SFO1           100.6228293 MHz

======== CHANNEL f2 ========
CPDPRG[2            waltz16
NUC2                     1H
PCPD2                 80.00 usec
PLW2            12.50000000 W
PLW12            0.43945000 W
PLW13            0.28125000 W
SFO2           400.1316005 MHz

F2 - Processing parameters
SI                    32768
SF             100.6126881 MHz
WDW                      EM
SSB       0
LB                     3.00 Hz
GB        0
PC                     1.40
```

Penta–S–penten–ol product 40 mg in 0.6 ml acetone–d6 *
1H{11B} NMR, 500 MHz, 23 C

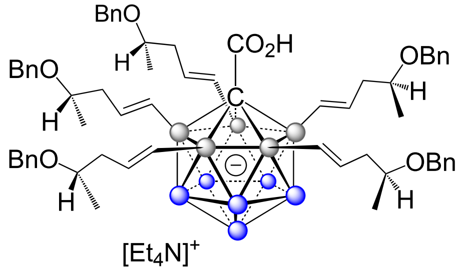

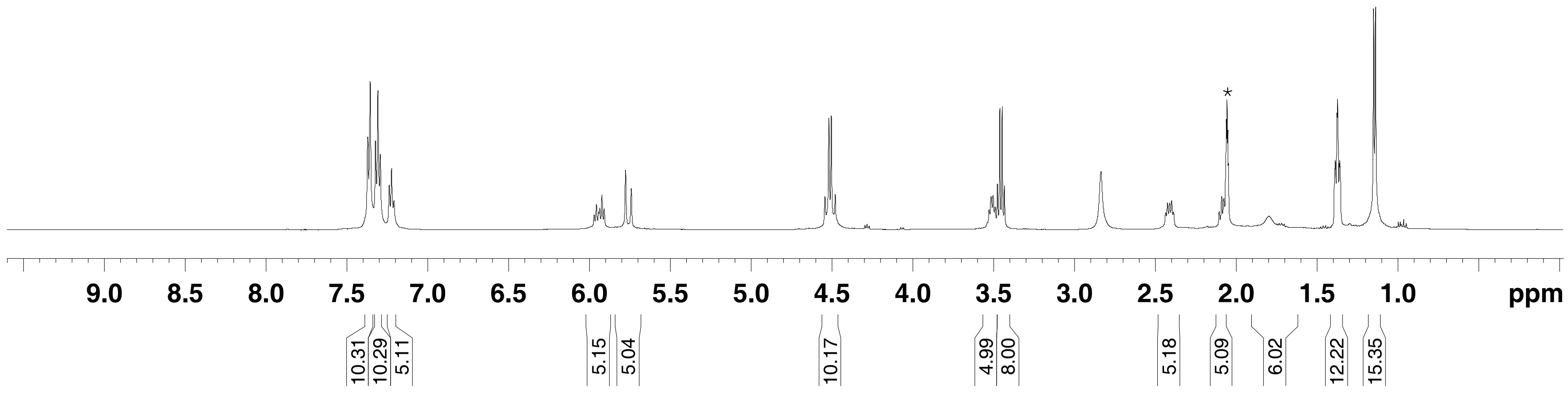


```
Current Data Parameters
NAME       penta-S-penten-ol
EXPNO                      2
PROCNO                     1

F2 - Acquisition Parameters
Date_               20180824
Time                    2.25
INSTRUM                spect
PROBHD     5 mm PABBO BB-
PULPROG              zgig30
TD                     65536
SOLVENT              Acetone
NS                        16
DS                         0
SWH             12500.000 Hz
FIDRES           0.190735 Hz
AQ              2.6214399 sec
RG                       114
DW                40.000 usec
DE                  6.50 usec
TE                   296.5 K
D1            5.00000000 sec
D11           0.03000000 sec
TD0                        1

======== CHANNEL f1 ========
SFO1        500.1335009 MHz
NUC1                      1H
P1                11.70 usec
PLW1          19.00000000 W

======== CHANNEL f2 ========
SFO2        160.4615690 MHz
NUC2                     11B
CPDPRG[2                garp
PCPD2            100.00 usec
PLW2          95.00000000 W
PLW12          1.63030005 W

F2 - Processing parameters
SI                     65536
SF          500.1300065 MHz
WDW                       EM
SSB       0
LB                   1.00 Hz
GB        0
PC                      1.00
```

Penta-S-penten-ol product 40 mg in 0.6 ml acetone-d6
11B NMR, 128 MHz, 23 C

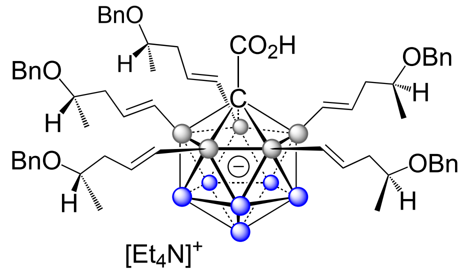

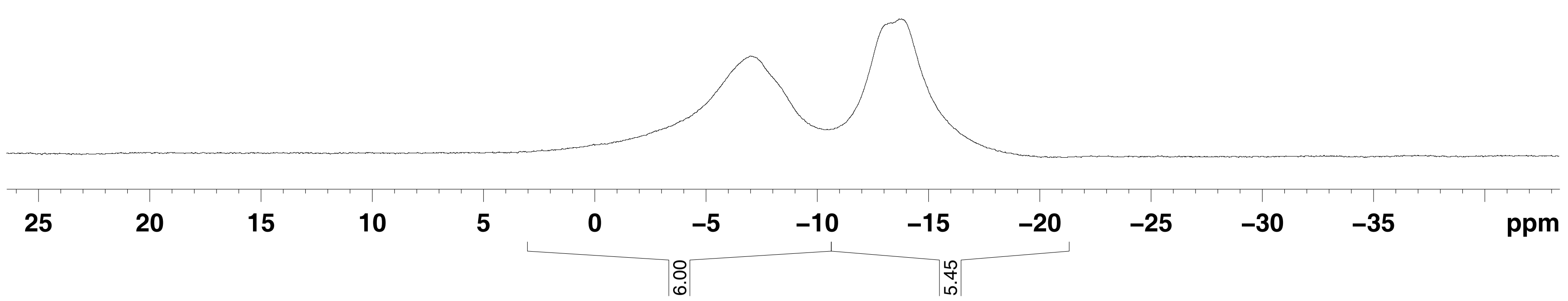


```
Current Data Parameters
NAME     penta-S-penten-ol
EXPNO                   3
PROCNO                  1

F2 - Acquisition Parameters
Date_          20180731
Time               3.45
INSTRUM           spect
PROBHD   5 mm PABBO BB/
PULPROG              zg
TD                65536
SOLVENT         Acetone
NS                  128
DS                    4
SWH           25510.203 Hz
FIDRES         0.389255 Hz
AQ            1.2845056 sec
RG               193.34
DW               19.600 usec
DE                 6.50 usec
TE                295.2 K
D1           1.00000000 sec
TD0                   1

======== CHANNEL f1 ========
NUC1                11B
P1                 9.93 usec
PLW1        52.96599960 W
SFO1        128.3776052 MHz

F2 - Processing parameters
SI                32768
SF          128.3776050 MHz
WDW                  EM
SSB      0
LB                 5.00 Hz
GB       0
PC                 1.40
```

Penta-S-penten-ol product 40 mg in 0.6 ml acetone-d6
11B{1H} NMR, 128 MHz, 23 C

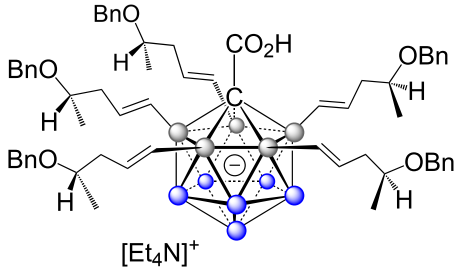

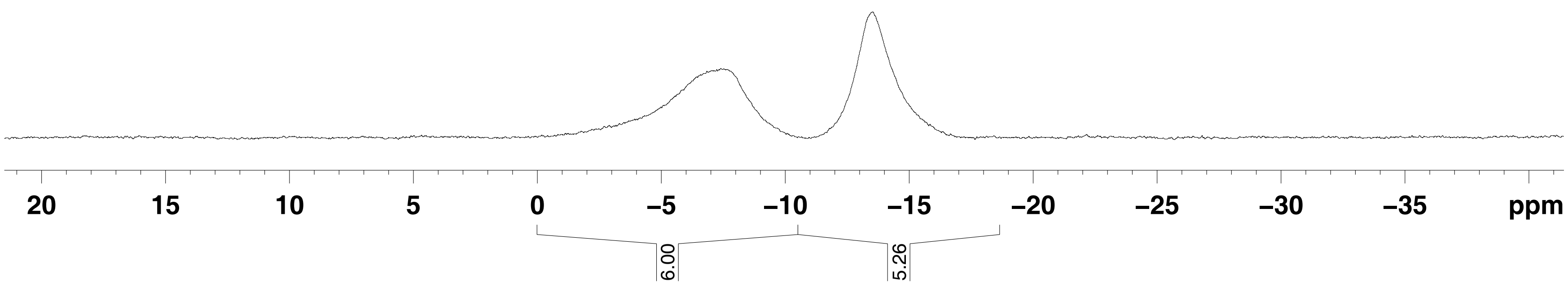


```
Current Data Parameters
NAME      penta-S-penten-ol
EXPNO                     4
PROCNO                    1

F2 - Acquisition Parameters
Date_              20180731
Time                   3.52
INSTRUM               spect
PROBHD   5 mm PABBO BB/
PULPROG              zgpg30
TD                    65536
SOLVENT             Acetone
NS                      128
DS                        4
SWH               25510.203 Hz
FIDRES             0.389255 Hz
AQ                1.2845056 sec
RG                   193.34
DW                   19.600 usec
DE                     6.50 usec
TE                    295.7 K
D1               1.00000000 sec
D11              0.03000000 sec
TD0                       1

======== CHANNEL f1 ========
NUC1                    11B
P1                     9.93 usec
PLW1            52.96599960 W
SFO1            128.3776050 MHz

======== CHANNEL f2 ========
CPDPRG[2            waltz16
NUC2                     1H
PCPD2                 80.00 usec
PLW2            12.50000000 W
PLW12            0.43945000 W
PLW13            0.28125000 W
SFO2            400.1320007 MHz

F2 - Processing parameters
SI                    32768
SF              128.3776050 MHz
WDW                      EM
SSB       0
LB                     5.00 Hz
GB        0
PC                     1.40
```

Penta–S–penten–ol product 40 mg in 0.6 ml acetone–d6*
13C{1H} NMR, 126 MHz, 23 C

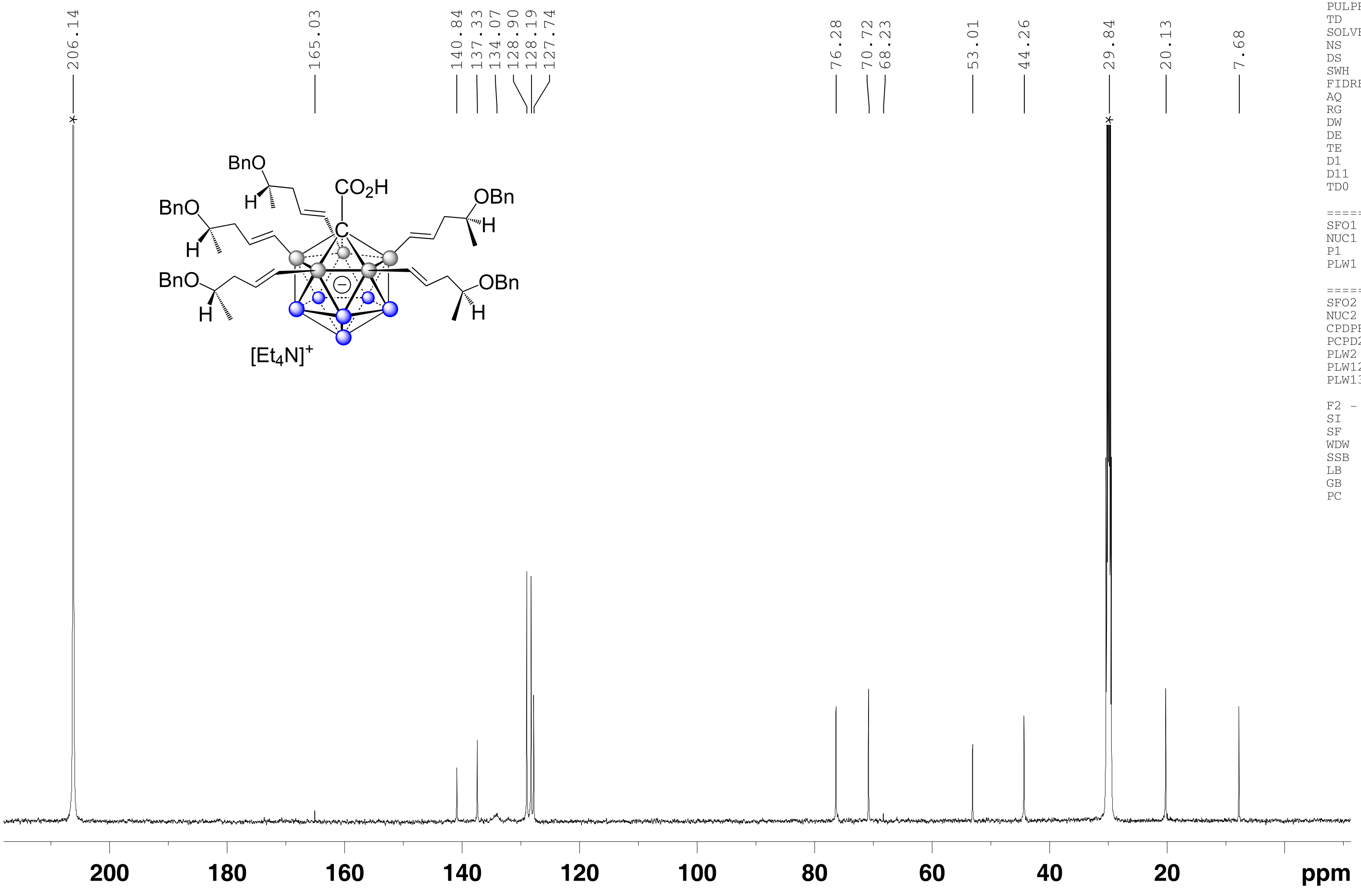


```
Current Data Parameters
NAME      penta-S-penten-ol
EXPNO                     5
PROCNO                    1

F2 - Acquisition Parameters
Date_              20180824
Time                   4.38
INSTRUM               spect
PROBHD    5 mm PABBO BB-
PULPROG             zgpg30
TD                   65536
SOLVENT             Acetone
NS                    3100
DS                       4
SWH              37878.789 Hz
FIDRES            0.577984 Hz
AQ               0.8650752 sec
RG                     203
DW                  13.200 usec
DE                    6.50 usec
TE                   296.5 K
D1              1.50000000 sec
D11             0.03000000 sec
TD0                      1

======== CHANNEL f1 ========
SFO1           125.7716224 MHz
NUC1                   13C
P1                   10.50 usec
PLW1           95.00000000 W

======== CHANNEL f2 ========
SFO2           500.1320005 MHz
NUC2                    1H
CPDPRG[2           waltz16
PCPD2                80.00 usec
PLW2           19.00000000 W
PLW12           0.40639001 W
PLW13           0.26008999 W

F2 - Processing parameters
SI                   32768
SF             125.7576776 MHz
WDW                     EM
SSB      0
LB                    5.00 Hz
GB       0
PC                    1.40
```

Penta–F–styrene–de product, 40 mg in 0.6 ml acetone–d6*
1H{11B} NMR, 400 MHz, 23 C

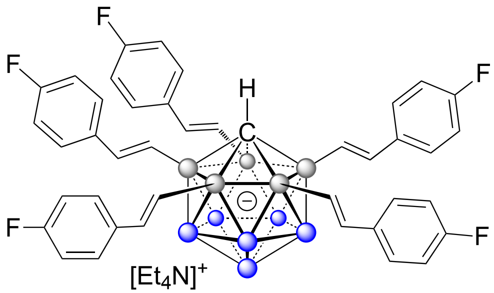

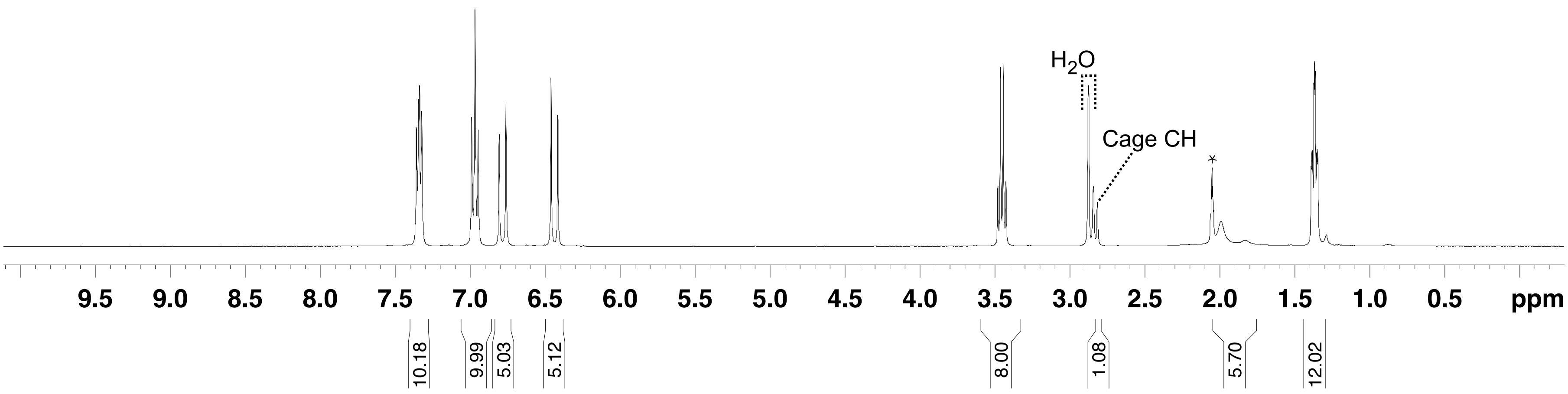


```
Current Data Parameters
NAME       penta-4-F-styrene-de
EXPNO                    1
PROCNO                   1

F2 - Acquisition Parameters
Date_             20180601
Time                 18.03
INSTRUM              spect
PROBHD    5 mm PABBO BB/
PULPROG             zgig30
TD                   16384
SOLVENT            Acetone
NS                      16
DS                       4
SWH               8012.820 Hz
FIDRES            0.489064 Hz
AQ               1.0223616 sec
RG                   107.6
DW                  62.400 usec
DE                    6.50 usec
TE                   294.3 K
D1              1.00000000 sec
D11             0.03000000 sec
TD0                      1

======== CHANNEL f1 ========
NUC1                    1H
P1                   15.00 usec
PLW1           12.50000000 W
SFO1           400.1320007 MHz

======== CHANNEL f2 ========
CPDPRG[2             garp4
NUC2                   11B
PCPD2                90.00 usec
PLW2           52.96599960 W
PLW12           0.64477998 W
SFO2           128.3776050 MHz

F2 - Processing parameters
SI                   32768
SF             400.1300072 MHz
WDW                     EM
SSB       0
LB                    1.00 Hz
GB        0
PC                    1.40
```

Penta–F–styrene–de product, 40 mg in 0.6 ml acetone–d6
11B NMR, 128 MHz, 23 C

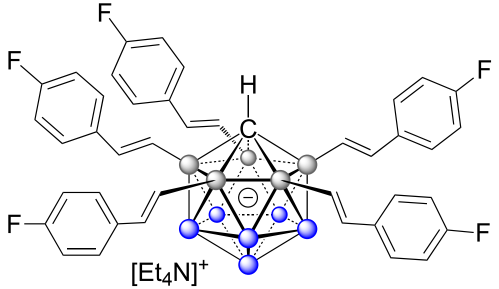

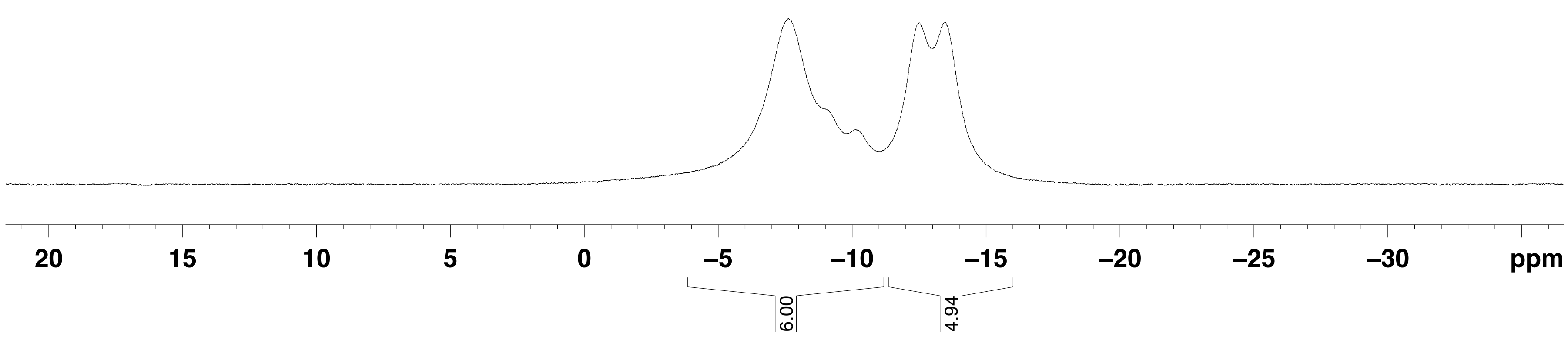


```
Current Data Parameters
NAME      penta-4-F-styrene-de
EXPNO                    2
PROCNO                   1

F2 - Acquisition Parameters
Date_             20180601
Time                 18.08
INSTRUM              spect
PROBHD    5 mm PABBO BB/
PULPROG                 zg
TD                   65536
SOLVENT            Acetone
NS                     128
DS                       4
SWH              25510.203 Hz
FIDRES            0.389255 Hz
AQ               1.2845056 sec
RG                  193.34
DW                  19.600 usec
DE                    6.50 usec
TE                   294.0 K
D1              1.00000000 sec
TD0                      1

======== CHANNEL f1 ========
NUC1                   11B
P1                    9.93 usec
PLW1           52.96599960 W
SFO1           128.3776052 MHz

F2 - Processing parameters
SI                   32768
SF             128.3776050 MHz
WDW                     EM
SSB      0
LB                    3.00 Hz
GB       0
PC                    1.40
```

Penta−F−styrene−de product, 40 mg in 0.6 ml acetone−d6
11B{1H} NMR, 128 MHz, 23 C

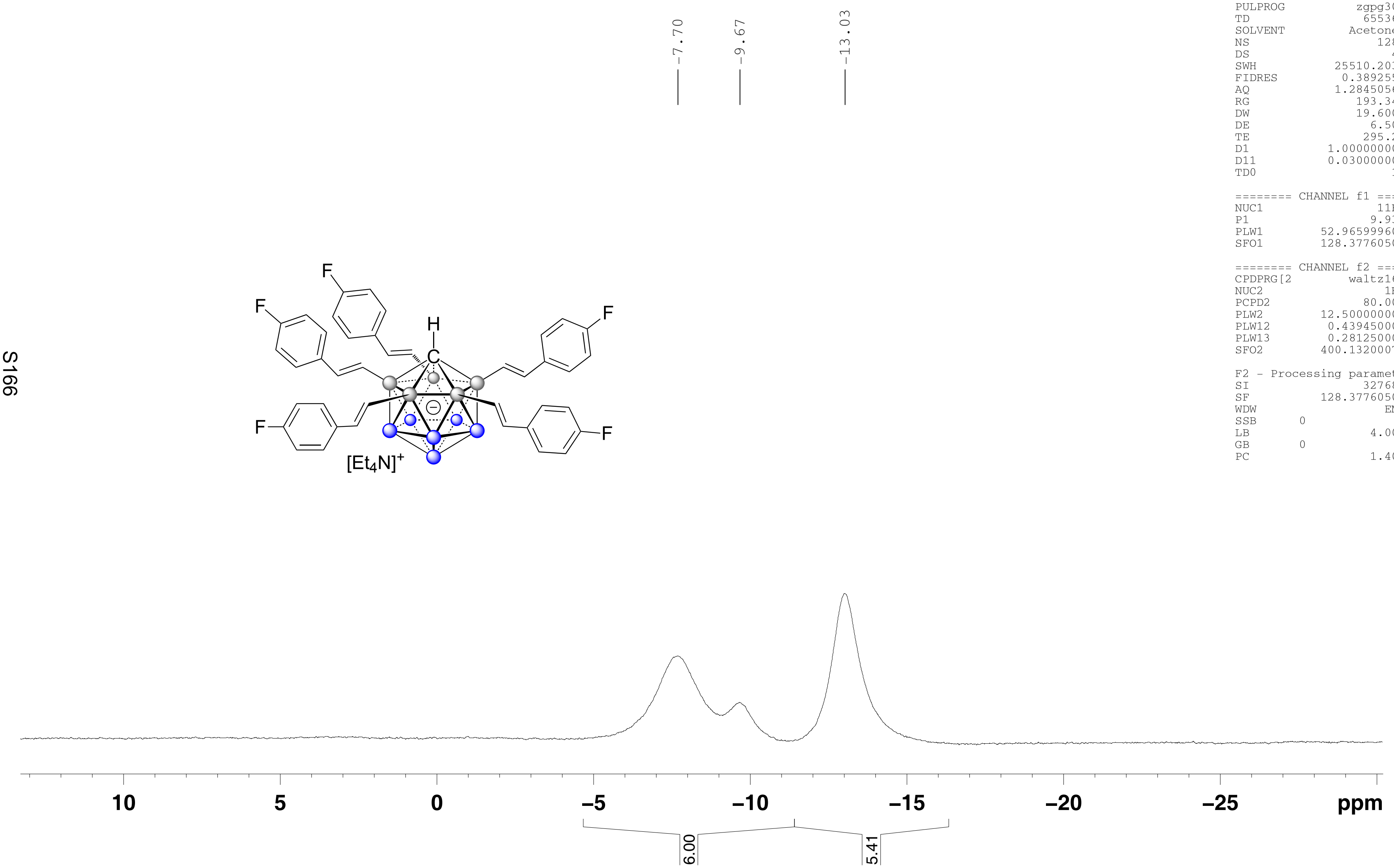


```
Current Data Parameters
NAME       penta-4-F-styrene-de
EXPNO                         3
PROCNO                        1

F2 - Acquisition Parameters
Date_               20180601
Time                   18.14
INSTRUM                spect
PROBHD    5 mm PABBO BB/
PULPROG               zgpg30
TD                     65536
SOLVENT              Acetone
NS                       128
DS                         4
SWH                25510.203 Hz
FIDRES              0.389255 Hz
AQ                 1.2845056 sec
RG                    193.34
DW                    19.600 usec
DE                      6.50 usec
TE                     295.2 K
D1                1.00000000 sec
D11               0.03000000 sec
TD0                        1

======== CHANNEL f1 ========
NUC1                     11B
P1                      9.93 usec
PLW1             52.96599960 W
SFO1             128.3776050 MHz

======== CHANNEL f2 ========
CPDPRG[2             waltz16
NUC2                      1H
PCPD2                  80.00 usec
PLW2             12.50000000 W
PLW12             0.43945000 W
PLW13             0.28125000 W
SFO2             400.1320007 MHz

F2 - Processing parameters
SI                     32768
SF               128.3776050 MHz
WDW                       EM
SSB      0
LB                      4.00 Hz
GB       0
PC                      1.40
```

Penta-F-styrene-de product, 40 mg in 0.6 ml acetone-d6 *
13C{1H} NMR, 100 MHz, 23 C

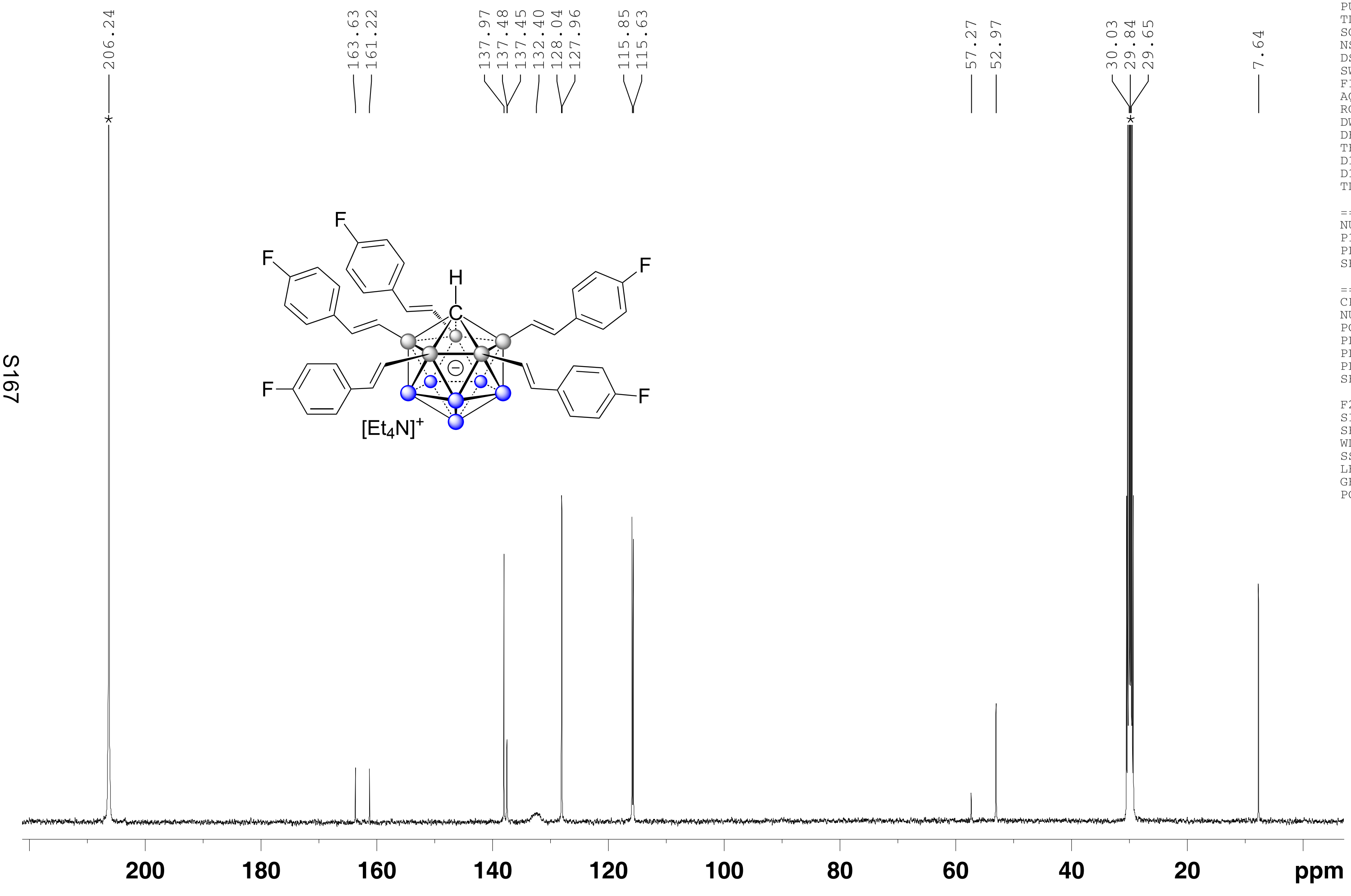


```
Current Data Parameters
NAME     penta-4-F-styrene-de
EXPNO                   4
PROCNO                  1

F2 - Acquisition Parameters
Date_          20180601
Time              19.47
INSTRUM           spect
PROBHD   5 mm PABBO BB/
PULPROG          zgpg30
TD                65536
SOLVENT         Acetone
NS                 2048
DS                    4
SWH           29761.904 Hz
FIDRES         0.454131 Hz
AQ            1.1010048 sec
RG               193.34
DW               16.800 usec
DE                 6.50 usec
TE                294.4 K
D1           1.50000000 sec
D11          0.03000000 sec
TD0                   1

======== CHANNEL f1 ========
NUC1                13C
P1                10.00 usec
PLW1        53.00000000 W
SFO1        100.6228293 MHz

======== CHANNEL f2 ========
CPDPRG[2        waltz16
NUC2                 1H
PCPD2             80.00 usec
PLW2        12.50000000 W
PLW12        0.43945000 W
PLW13        0.28125000 W
SFO2        400.1316005 MHz

F2 - Processing parameters
SI                32768
SF          100.6126818 MHz
WDW                  EM
SSB      0
LB                 3.00 Hz
GB       0
PC                 1.40
```

Penta–styrene–de product, 40 mg in 0.6 ml acetone–d6*
1H{11B} NMR, 400 MHz, 23 C

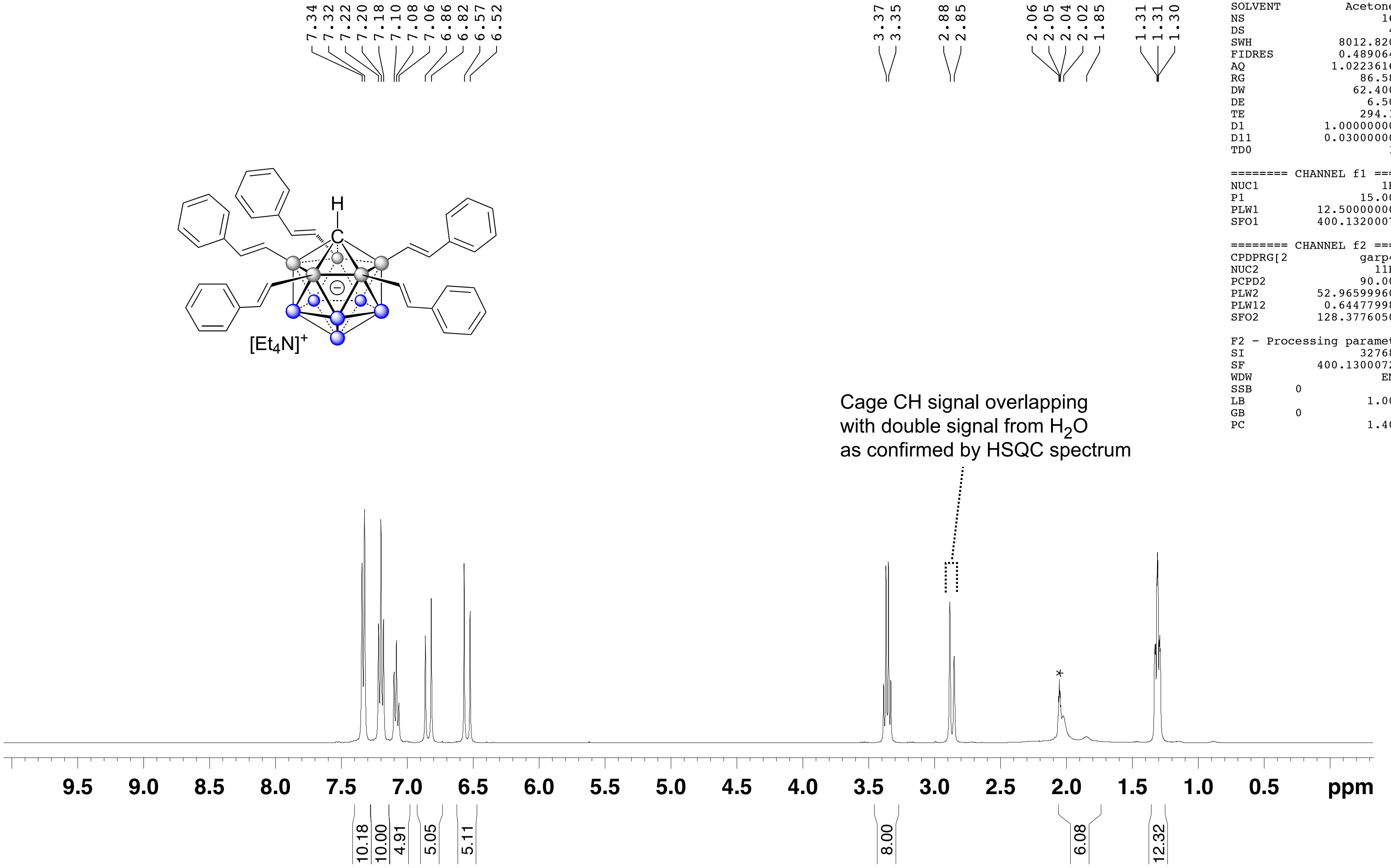


```
Current Data Parameters
NAME     penta-styrene-de
EXPNO                   1
PROCNO                  1

F2 - Acquisition Parameters
Date_            20180531
Time                17.43
INSTRUM             spect
PROBHD   5 mm PABBO BB/
PULPROG            zgig30
TD                  16384
SOLVENT           Acetone
NS                     16
DS                      4
SWH              8012.820 Hz
FIDRES           0.489064 Hz
AQ              1.0223616 sec
RG                  86.58
DW                 62.400 usec
DE                   6.50 usec
TE                  294.1 K
D1             1.00000000 sec
D11            0.03000000 sec
TD0                     1

======== CHANNEL f1 ========
NUC1                   1H
P1                  15.00 usec
PLW1          12.50000000 W
SFO1          400.1320007 MHz

======== CHANNEL f2 ========
CPDPRG[2            garp4
NUC2                  11B
PCPD2               90.00 usec
PLW2          52.96599960 W
PLW12          0.64477998 W
SFO2          128.3776050 MHz

F2 - Processing parameters
SI                  32768
SF            400.1300072 MHz
WDW                    EM
SSB      0
LB                   1.00 Hz
GB       0
PC                   1.40
```

Penta-styrene-de product, 40 mg in 0.6 ml acetone-d6
11B NMR, 128 MHz, 23 C

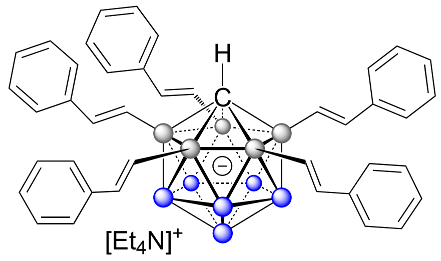

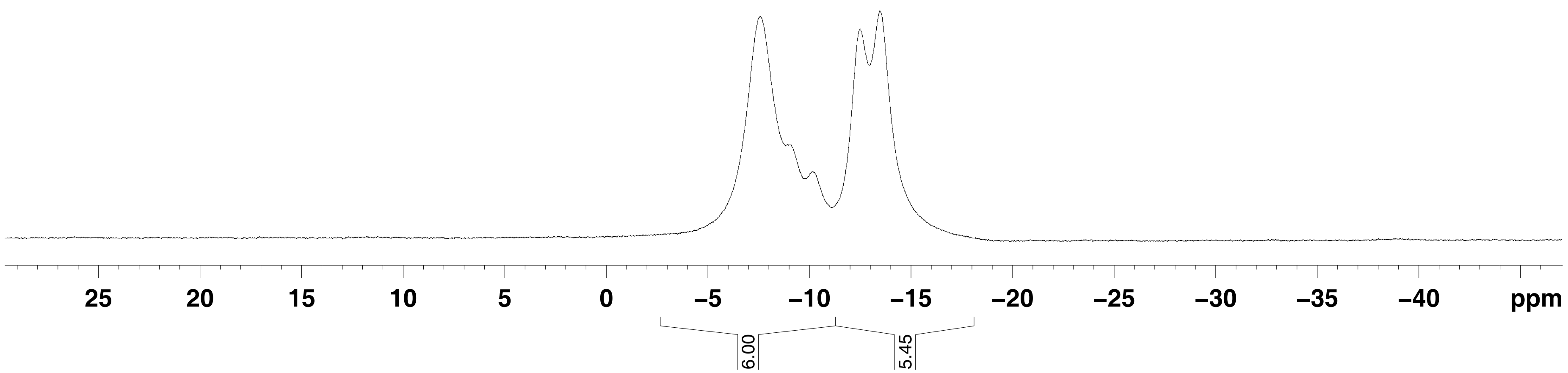


```
Current Data Parameters
NAME        penta-styrene-de
EXPNO                      2
PROCNO                     1

F2 - Acquisition Parameters
Date_               20180531
Time                   17.49
INSTRUM                spect
PROBHD     5 mm PABBO BB/
PULPROG                   zg
TD                     65536
SOLVENT              Acetone
NS                       128
DS                         4
SWH                25510.203 Hz
FIDRES              0.389255 Hz
AQ                 1.2845056 sec
RG                    193.34
DW                    19.600 usec
DE                      6.50 usec
TE                     293.5 K
D1                1.00000000 sec
TD0                        1

======== CHANNEL f1 ========
NUC1                     11B
P1                      9.93 usec
PLW1             52.96599960 W
SFO1             128.3776052 MHz

F2 - Processing parameters
SI                     32768
SF               128.3776050 MHz
WDW                       EM
SSB      0
LB                      3.00 Hz
GB       0
PC                      1.40
```

Penta-styrene-de product, 40 mg in 0.6 ml acetone-d6
11B{1H} NMR, 128 MHz, 23 C

```
Current Data Parameters
NAME      penta-styrene-de
EXPNO                    3
PROCNO                   1

F2 - Acquisition Parameters
Date_             20180531
Time                 17.55
INSTRUM              spect
PROBHD   5 mm PABBO BB/
PULPROG            zgpg30
TD                  65536
SOLVENT           Acetone
NS                    128
DS                      4
SWH             25510.203 Hz
FIDRES           0.389255 Hz
AQ              1.2845056 sec
RG                 193.34
DW                 19.600 usec
DE                   6.50 usec
TE                  294.8 K
D1             1.00000000 sec
D11            0.03000000 sec
TD0                     1

======== CHANNEL f1 ========
NUC1                  11B
P1                   9.93 usec
PLW1          52.96599960 W
SFO1          128.3776050 MHz

======== CHANNEL f2 ========
CPDPRG[2          waltz16
NUC2                   1H
PCPD2               80.00 usec
PLW2          12.50000000 W
PLW12          0.43945000 W
PLW13          0.28125000 W
SFO2          400.1320007 MHz

F2 - Processing parameters
SI                  32768
SF            128.3776050 MHz
WDW                    EM
SSB      0
LB                   4.00 Hz
GB       0
PC                   1.40
```

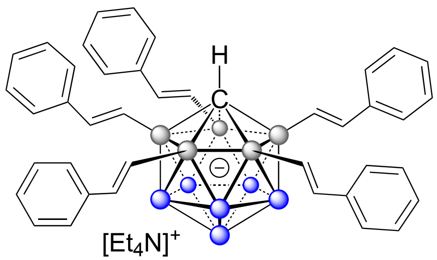

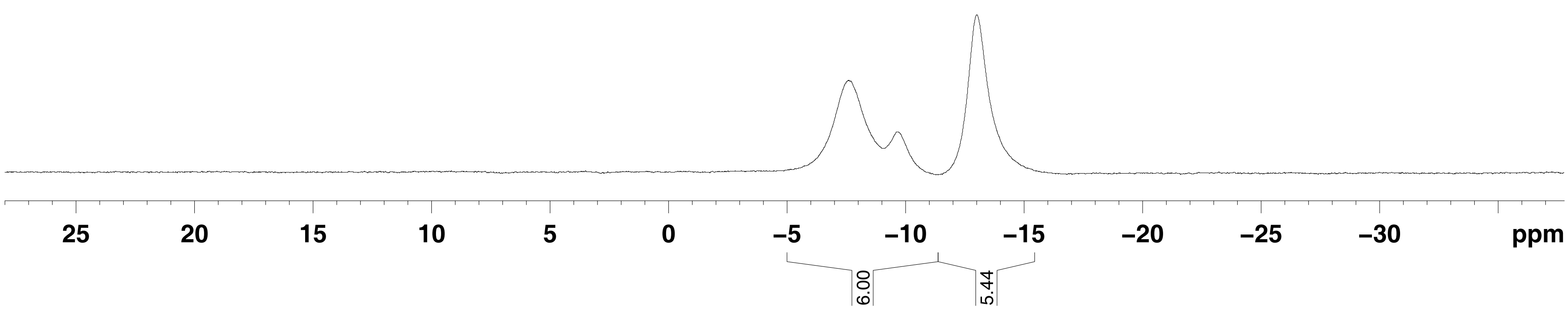

Penta–styrene–de product, 40 mg in 0.6 ml acetone–d6 *
13C{1H} NMR, 101 MHz, 23 C

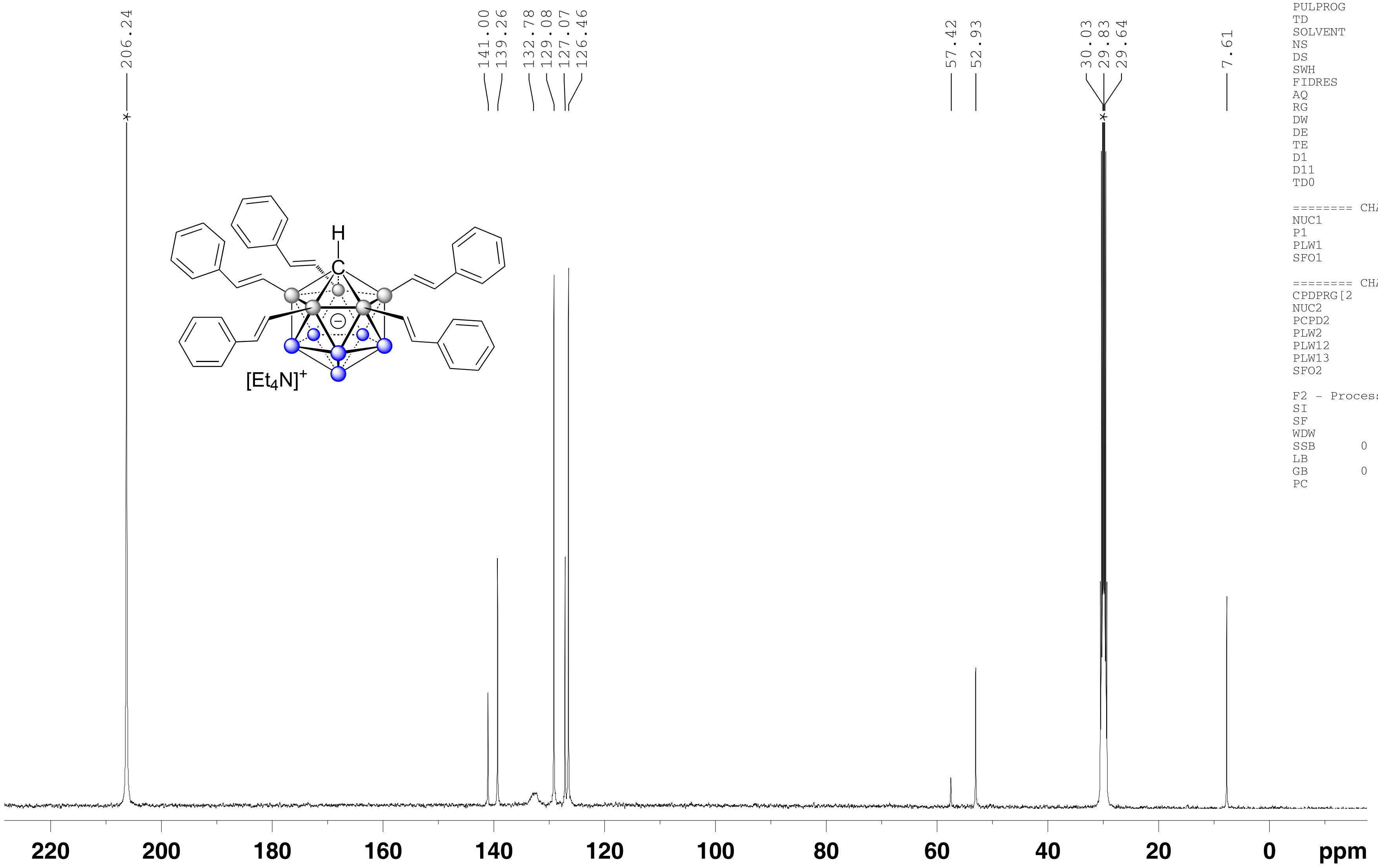


```
Current Data Parameters
NAME       penta-styrene-de
EXPNO                     4
PROCNO                    1

F2 - Acquisition Parameters
Date_              20180531
Time                  19.28
INSTRUM               spect
PROBHD     5 mm PABBO BB/
PULPROG             zgpg30
TD                    65536
SOLVENT             Acetone
NS                     2048
DS                        4
SWH              29761.904 Hz
FIDRES            0.454131 Hz
AQ               1.1010048 sec
RG                   193.34
DW                   16.800 usec
DE                     6.50 usec
TE                    294.8 K
D1               1.50000000 sec
D11              0.03000000 sec
TD0                       1

======== CHANNEL f1 ========
NUC1                    13C
P1                    10.00 usec
PLW1            53.00000000 W
SFO1            100.6228293 MHz

======== CHANNEL f2 ========
CPDPRG[2            waltz16
NUC2                     1H
PCPD2                 80.00 usec
PLW2            12.50000000 W
PLW12            0.43945000 W
PLW13            0.28125000 W
SFO2            400.1316005 MHz

F2 - Processing parameters
SI                    32768
SF              100.6126842 MHz
WDW                      EM
SSB       0
LB                     5.00 Hz
GB        0
PC                     1.40
```

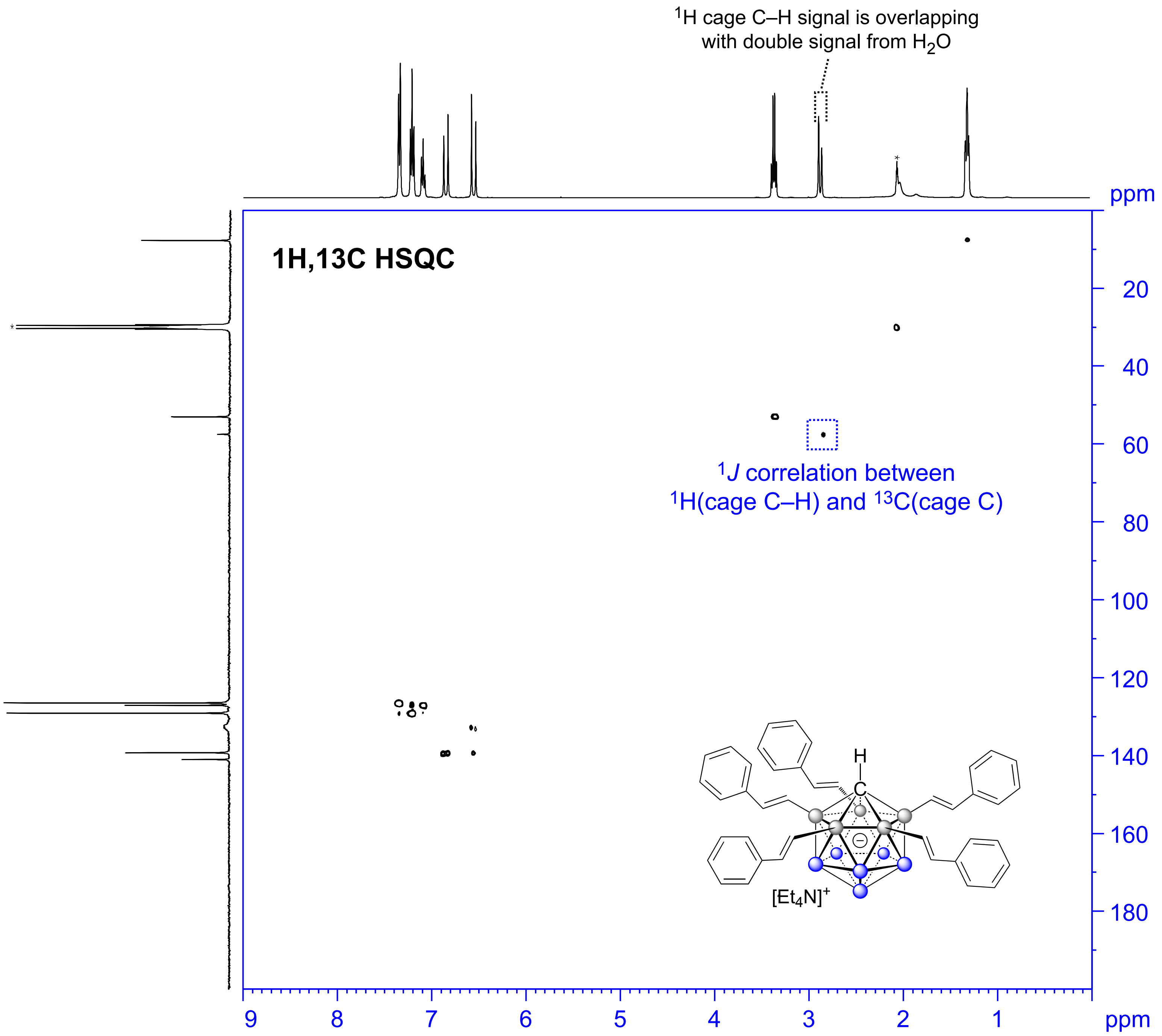


```
Current Data Parameters
NAME       20180804-phde-hsqc
EXPNO                       3
PROCNO                      1

F2 - Acquisition Parameters
Date_               20180805
Time                   20.08
INSTRUM                spect
PROBHD   5 mm PABBO BB/
PULPROG        hsqcetgpsi2
TD                      1024
SOLVENT              Acetone
NS                         2
DS                        16
SWH                 6009.615 Hz
FIDRES              5.868765 Hz
AQ                 0.0851968 sec
RG                    193.34
DW                    83.200 usec
DE                      6.50 usec
TE                     295.0 K
CNST2            145.0000000
D0                0.00000300 sec
D1                1.50000000 sec
D4                0.00172414 sec
D11               0.03000000 sec
D16               0.00020000 sec
D24               0.00086207 sec
IN0               0.00001990 sec
ZGOPTNS

======== CHANNEL f1 ========
NUC1                      1H
P1                     15.00 usec
P2                     30.00 usec
P28                  1000.00 usec
PLW1             12.50000000 W
SFO1             400.1328009 MHz

======== CHANNEL f2 ========
CPDPRG[2                garp
NUC2                     13C
P3                     10.00 usec
P4                     20.00 usec
PCPD2                  70.00 usec
PLW2             53.00000000 W
PLW12             1.08159995 W
SFO2             100.6238364 MHz

====== GRADIENT CHANNEL =====
GPNAM[1]         SMSQ10.100
GPNAM[2]         SMSQ10.100
GPNAM[3]         SMSQ10.100
GPNAM[4]         SMSQ10.100
GPZ1                   80.00 %
GPZ2                   20.10 %
GPZ3                   11.00 %
GPZ4                   -5.00 %
P16                  1000.00 usec
P19                   600.00 usec

F1 - Acquisition parameters
TD                       256
SFO1                100.6238 MHz
FIDRES            196.524048 Hz
SW                   249.991 ppm
FnMODE          Echo-Antiecho

F2 - Processing parameters
SI                      1024
SF               400.1299985 MHz
WDW                    QSINE
SSB                        2
LB                      0 Hz
GB                         0
PC                      1.40

F1 - Processing parameters
SI                      1024
MC2             echo-antiecho
SF               100.6126624 MHz
WDW                    QSINE
SSB                        2
LB                      0 Hz
GB                         0
```

**12–Me–Penta–F–styrine–de product 40 mg in 0.6 ml acetone–d6*
1H{11B} NMR, 400 MHz, 23 C**

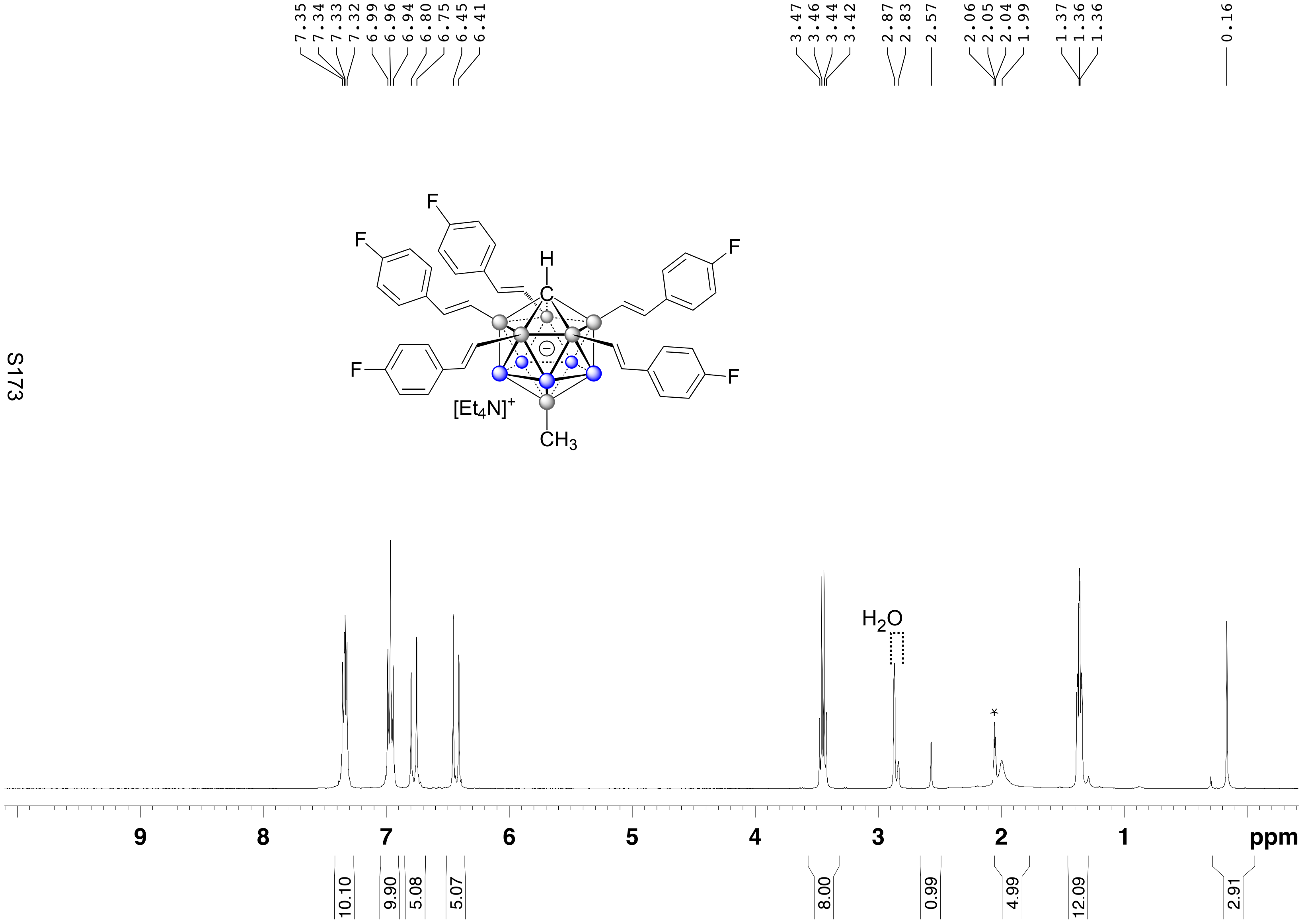


```
Current Data Parameters
NAME     12-Me-penta-4-F-styrene-de
EXPNO                     1
PROCNO                    1

F2 - Acquisition Parameters
Date_              20180610
Time                  13.51
INSTRUM               spect
PROBHD   5 mm PABBO BB/
PULPROG              zgig30
TD                    16384
SOLVENT             Acetone
NS                       16
DS                        4
SWH                8012.820 Hz
FIDRES             0.489064 Hz
AQ                1.0223616 sec
RG                    107.6
DW                   62.400 usec
DE                     6.50 usec
TE                    293.7 K
D1               1.00000000 sec
D11              0.03000000 sec
TD0                       1

======== CHANNEL f1 ========
NUC1                     1H
P1                    15.00 usec
PLW1            12.50000000 W
SFO1            400.1320007 MHz

======== CHANNEL f2 ========
CPDPRG[2              garp4
NUC2                    11B
PCPD2                 90.00 usec
PLW2            52.96599960 W
PLW12            0.64477998 W
SFO2            128.3776050 MHz

F2 - Processing parameters
SI                    32768
SF              400.1300072 MHz
WDW                      EM
SSB      0
LB                     1.00 Hz
GB       0
PC                     1.40
```

12–Me–Penta–F–styrine–de product 40 mg in 0.6 ml acetone–d6
11B NMR, 128 MHz, 23 C

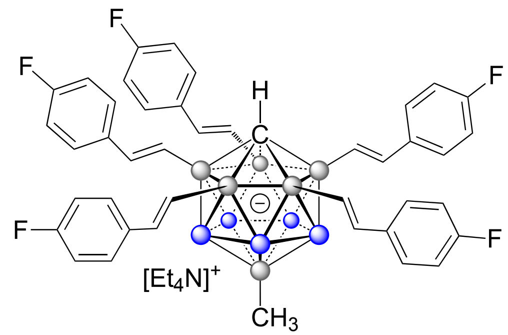

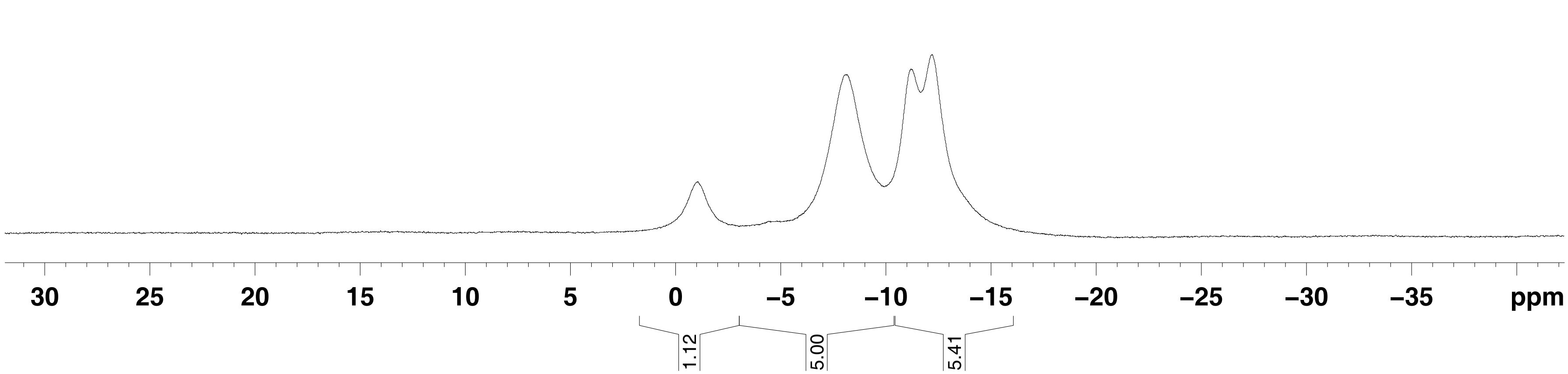


```
Current Data Parameters
NAME     12-Me-penta-4-F-styrene-de
EXPNO                   2
PROCNO                  1

F2 - Acquisition Parameters
Date_          20180610
Time              13.56
INSTRUM           spect
PROBHD   5 mm PABBO BB/
PULPROG              zg
TD                65536
SOLVENT         Acetone
NS                  128
DS                    4
SWH           25510.203 Hz
FIDRES         0.389255 Hz
AQ            1.2845056 sec
RG               193.34
DW               19.600 usec
DE                 6.50 usec
TE                293.8 K
D1           1.00000000 sec
TD0                   1

======== CHANNEL f1 ========
NUC1                11B
P1                 9.93 usec
PLW1        52.96599960 W
SFO1        128.3776052 MHz

F2 - Processing parameters
SI                32768
SF          128.3776050 MHz
WDW                  EM
SSB      0
LB                 3.00 Hz
GB       0
PC                 1.40
```

12–Me–Penta–F–styrine–de product 40 mg in 0.6 ml acetone–d6
11B{1H} NMR, 128 MHz, 23 C

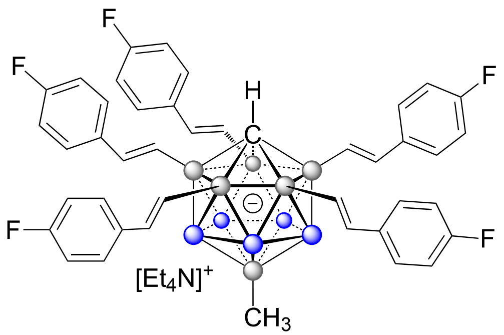

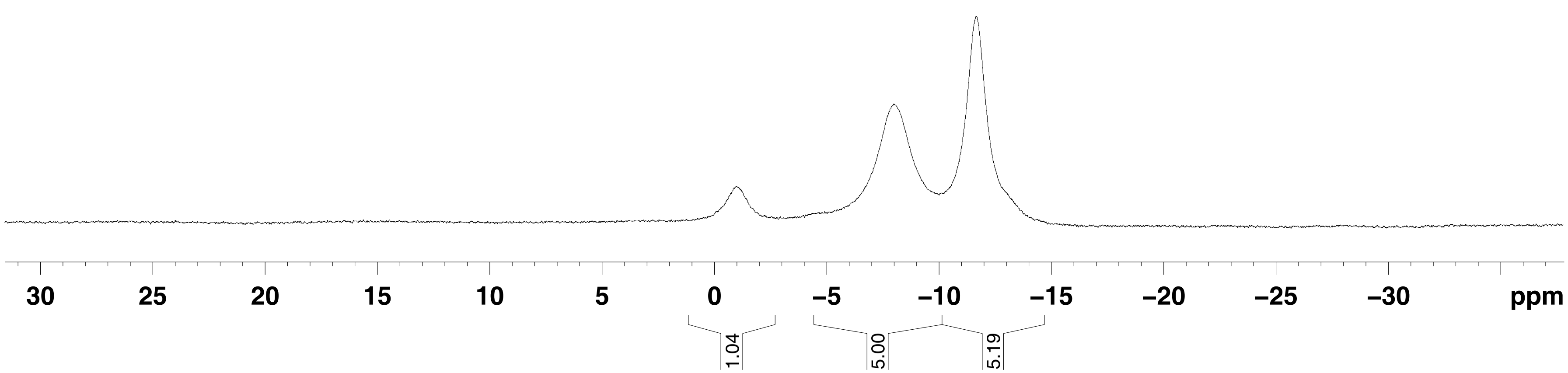


```
Current Data Parameters
NAME     12-Me-penta-4-F-styrene-de
EXPNO                    3
PROCNO                   1

F2 - Acquisition Parameters
Date_           20180610
Time               14.03
INSTRUM            spect
PROBHD   5 mm PABBO BB/
PULPROG           zgpg30
TD                 65536
SOLVENT          Acetone
NS                   128
DS                     4
SWH            25510.203 Hz
FIDRES          0.389255 Hz
AQ             1.2845056 sec
RG                193.34
DW                19.600 usec
DE                  6.50 usec
TE                 293.7 K
D1            1.00000000 sec
D11           0.03000000 sec
TD0                    1

======== CHANNEL f1 ========
NUC1                 11B
P1                  9.93 usec
PLW1         52.96599960 W
SFO1         128.3776050 MHz

======== CHANNEL f2 ========
CPDPRG[2         waltz16
NUC2                  1H
PCPD2              80.00 usec
PLW2         12.50000000 W
PLW12         0.43945000 W
PLW13         0.28125000 W
SFO2         400.1320007 MHz

F2 - Processing parameters
SI                 32768
SF           128.3776050 MHz
WDW                   EM
SSB      0
LB                  3.00 Hz
GB       0
PC                  1.40
```

12−Me−Penta−F−styrine−de product 40 mg in 0.6 ml acetone−d6*
13C{1H} NMR, 101 MHz, 23 C

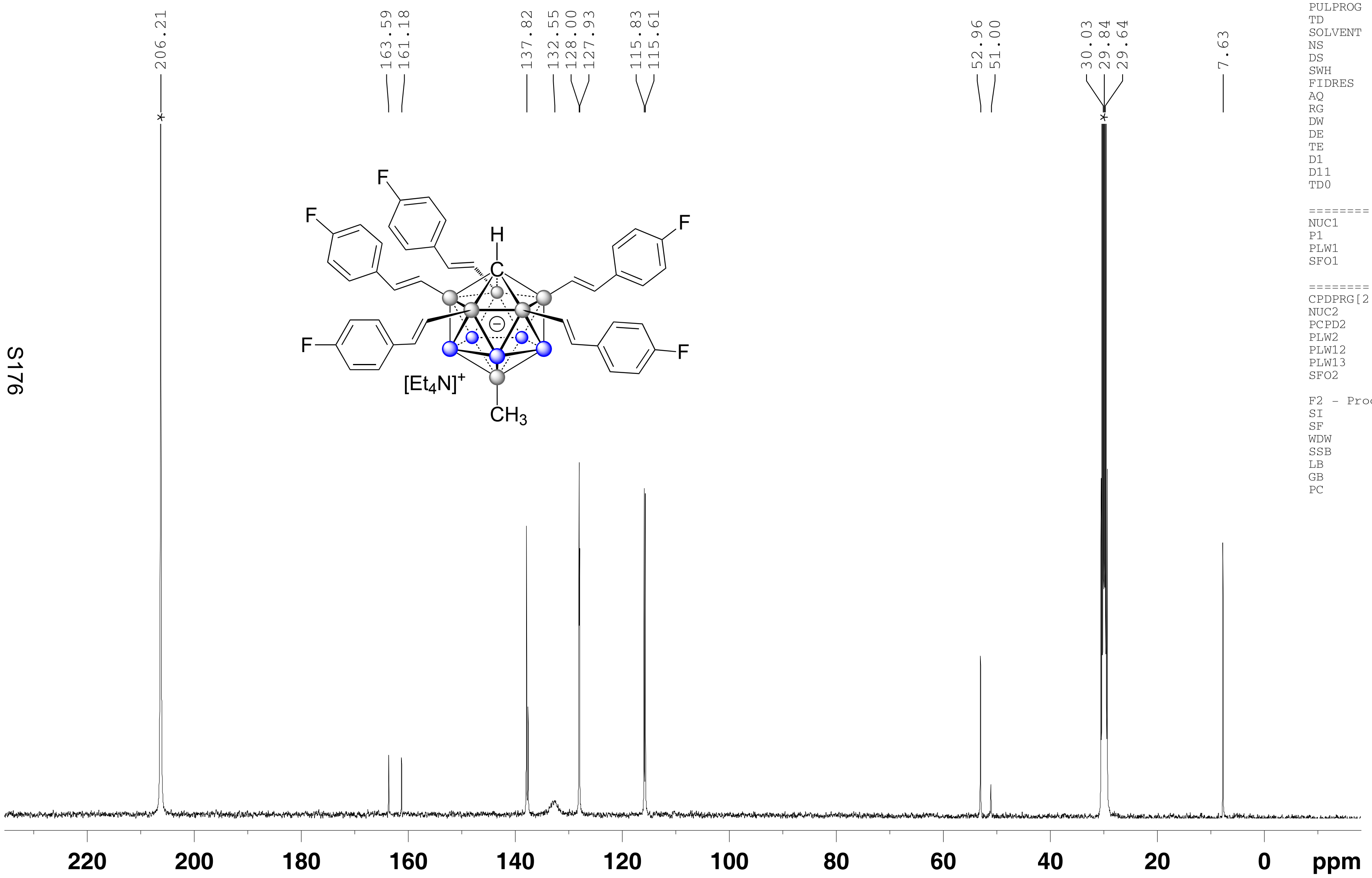

Current Data Parameters	
NAME	12-Me-penta-4-F-styrene-de
EXPNO	4
PROCNO	1
F2 - Acquisition Parameters	
Date_	20180610
Time	15.35
INSTRUM	spect
PROBHD	5 mm PABBO BB/
PULPROG	zgpg30
TD	65536
SOLVENT	Acetone
NS	2048
DS	4
SWH	29761.904 Hz
FIDRES	0.454131 Hz
AQ	1.1010048 sec
RG	193.34
DW	16.800 usec
DE	6.50 usec
TE	294.9 K
D1	1.50000000 sec
D11	0.03000000 sec
TD0	1
======== CHANNEL f1 ========	
NUC1	13C
P1	10.00 usec
PLW1	53.00000000 W
SFO1	100.6228293 MHz
======== CHANNEL f2 ========	
CPDPRG[2	waltz16
NUC2	1H
PCPD2	80.00 usec
PLW2	12.50000000 W
PLW12	0.43945000 W
PLW13	0.28125000 W
SFO2	400.1316005 MHz
F2 - Processing parameters	
SI	32768
SF	100.6126823 MHz
WDW	EM
SSB	0
LB	4.00 Hz
GB	0
PC	1.40

12–Ph–Penta–styrene–de product 40 mg in 0.6 ml acetone–d6*
1H{11B} NMR, 500 MHz, 23 C

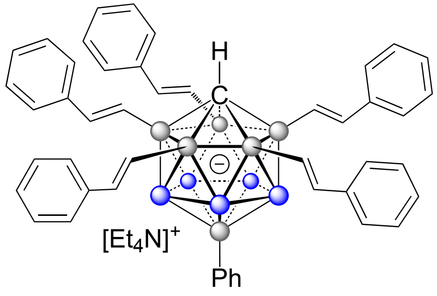

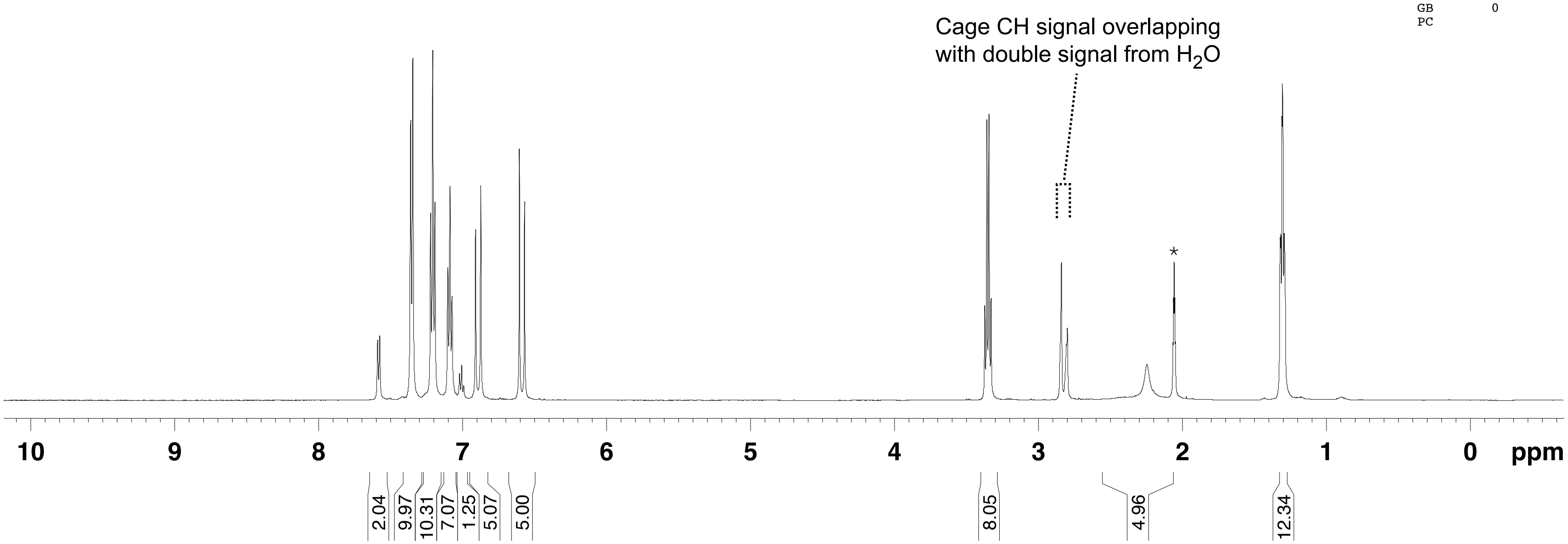


```
Current Data Parameters
NAME      12-Ph-penta-styrene-de
EXPNO                  1
PROCNO                 1

F2 - Acquisition Parameters
Date_           20180614
Time               18.07
INSTRUM            spect
PROBHD   5 mm PABBO BB-
PULPROG           zgig30
TD                 65536
SOLVENT          Acetone
NS                    16
DS                     0
SWH            12500.000 Hz
FIDRES          0.190735 Hz
AQ             2.6214399 sec
RG                   114
DW                40.000 usec
DE                  6.50 usec
TE                 295.9 K
D1            5.00000000 sec
D11           0.03000000 sec

======== CHANNEL f1 ========
NUC1                  1H
P1                 11.70 usec
PLW1         19.00000000 W
SFO1         500.1335009 MHz

======== CHANNEL f2 ========
CPDPRG[2            garp
NUC2                 11B
PCPD2             100.00 usec
PLW2         95.00000000 W
PLW12         1.63030005 W
SFO2         160.4615690 MHz

F2 - Processing parameters
SI                 65536
SF           500.1300101 MHz
WDW                   EM
SSB      0
LB                  1.00 Hz
GB       0
PC                  1.00
```

12–Ph–Penta–styrene–de product 40 mg in 0.6 ml acetone–d6
11B NMR, 160 MHz, 23 C

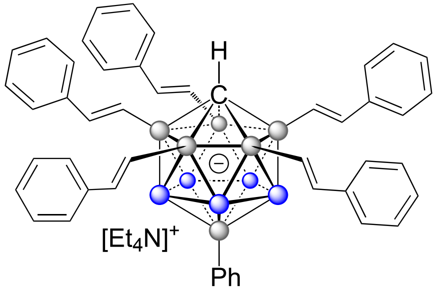

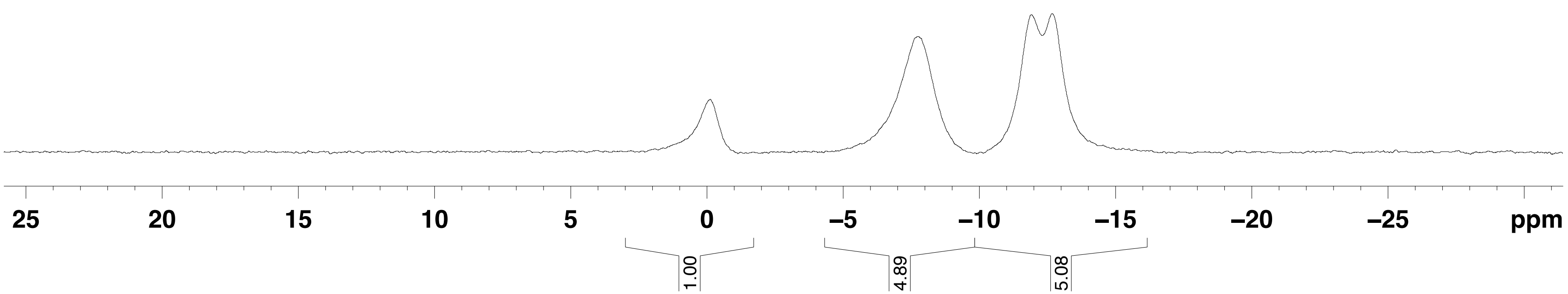


```
Current Data Parameters
NAME     12-Ph-penta-styrene-de
EXPNO                    2
PROCNO                   1

F2 - Acquisition Parameters
Date_          20180614
Time              18.10
INSTRUM           spect
PROBHD   5 mm PABBO BB-
PULPROG            zg30
TD                64098
SOLVENT         Acetone
NS                   64
DS                    0
SWH           32051.281 Hz
FIDRES         0.500036 Hz
AQ            0.9999288 sec
RG                  203
DW               15.600 usec
DE                 6.50 usec
TE                295.4 K
D1           1.00000000 sec

======== CHANNEL f1 ========
NUC1                11B
P1                13.10 usec
PLW1        95.00000000 W
SFO1        160.4615792 MHz

F2 - Processing parameters
SI                32768
SF          160.4615790 MHz
WDW                  EM
SSB      0
LB                10.00 Hz
GB       0
PC                 1.40
```

12–Ph–Penta–styrene–de product 40 mg in 0.6 ml acetone–d6
11B{1H} NMR, 160 MHz, 23 C

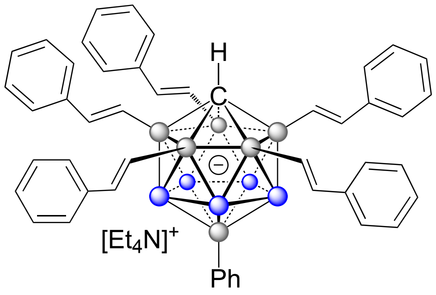

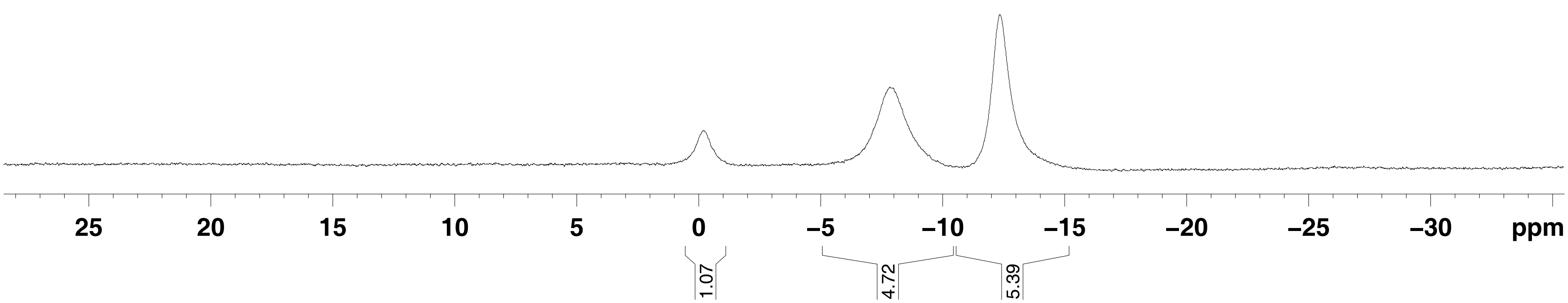


```
Current Data Parameters
NAME      12-Ph-penta-styrene-de
EXPNO                   3
PROCNO                  1

F2 - Acquisition Parameters
Date_            20180614
Time                18.13
INSTRUM             spect
PROBHD   5 mm PABBO BB-
PULPROG            zgpg30
TD                  65536
SOLVENT           Acetone
NS                     64
DS                      0
SWH             32051.281 Hz
FIDRES           0.489064 Hz
AQ              1.0223616 sec
RG                    203
DW                 15.600 usec
DE                   6.50 usec
TE                  296.1 K
D1             1.00000000 sec
D11            0.03000000 sec

======== CHANNEL f1 ========
NUC1                  11B
P1                  13.10 usec
PLW1          95.00000000 W
SFO1          160.4615790 MHz

======== CHANNEL f2 ========
CPDPRG[2          waltz16
NUC2                   1H
PCPD2               80.00 usec
PLW2          19.00000000 W
PLW12          0.40639001 W
PLW13          0.26008999 W
SFO2          500.1325007 MHz

F2 - Processing parameters
SI                  32768
SF            160.4615790 MHz
WDW                    EM
SSB      0
LB                   4.00 Hz
GB       0
PC                   1.40
```

12–Ph–Penta–styrene–de product 40 mg in 0.6 ml acetone–d6*
13C{1H} NMR, 126 MHz, 23 C

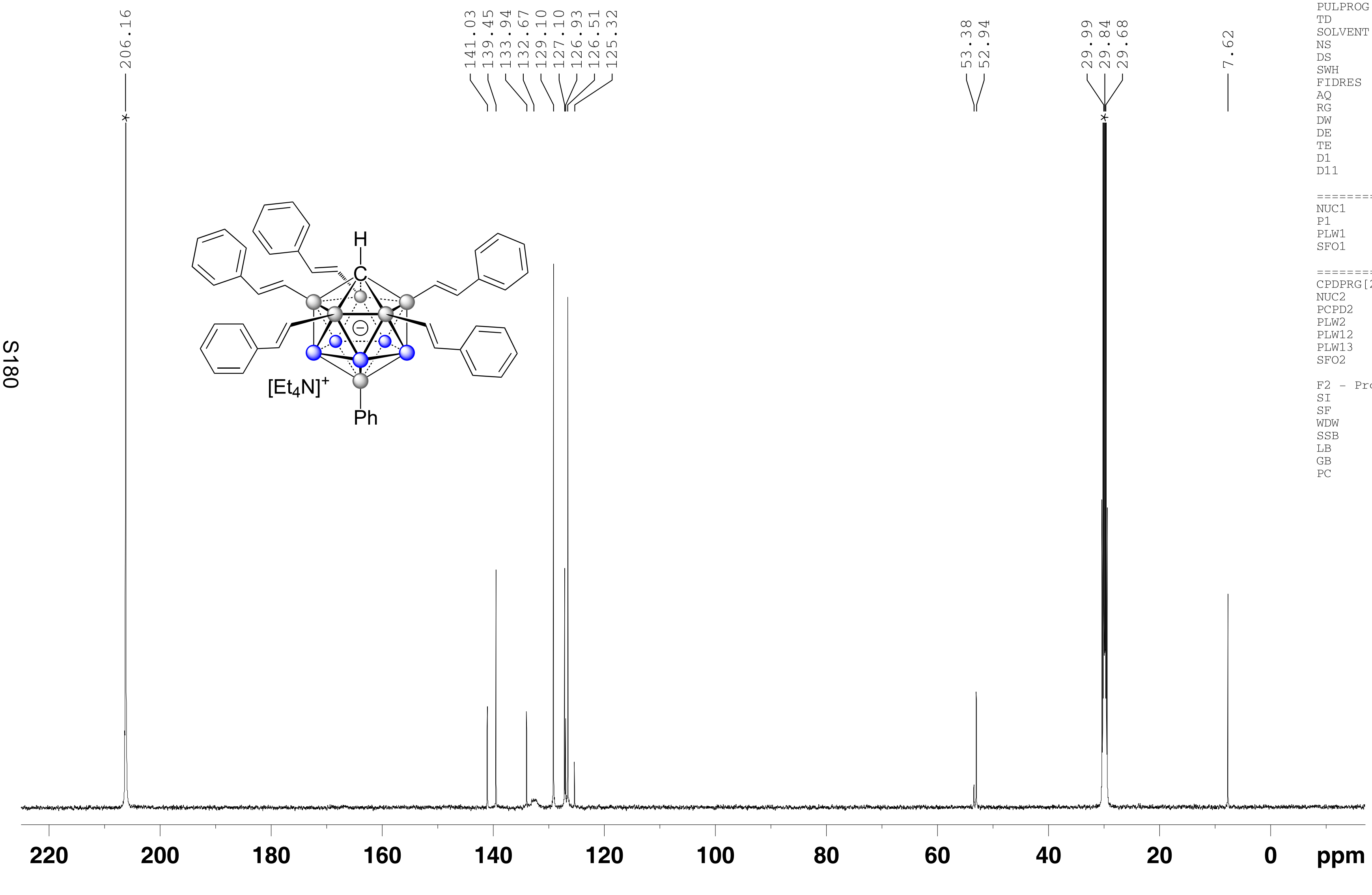


```
Current Data Parameters
NAME     12-Ph-penta-styrene-de
EXPNO                 4
PROCNO                1

F2 - Acquisition Parameters
Date_          20180614
Time              19.16
INSTRUM           spect
PROBHD   5 mm PABBO BB-
PULPROG         zgpg30
TD               65536
SOLVENT         Acetone
NS                2000
DS                   4
SWH          37878.789 Hz
FIDRES        0.577984 Hz
AQ           0.8650752 sec
RG                 203
DW              13.200 usec
DE                6.50 usec
TE               296.3 K
D1          1.50000000 sec
D11         0.03000000 sec

======== CHANNEL f1 ========
NUC1                13C
P1                10.50 usec
PLW1        95.00000000 W
SFO1        125.7716224 MHz

======== CHANNEL f2 ========
CPDPRG[2         waltz16
NUC2                 1H
PCPD2             80.00 usec
PLW2        19.00000000 W
PLW12        0.40639001 W
PLW13        0.26008999 W
SFO2        500.1320005 MHz

F2 - Processing parameters
SI                32768
SF          125.7576798 MHz
WDW                  EM
SSB      0
LB                 3.00 Hz
GB       0
PC                 1.40
```

12–CN–Penta–F–styrine–de product 40 mg in 0.6 ml acetone–d6*
1H{11B} NMR, 400 MHz, 23 C

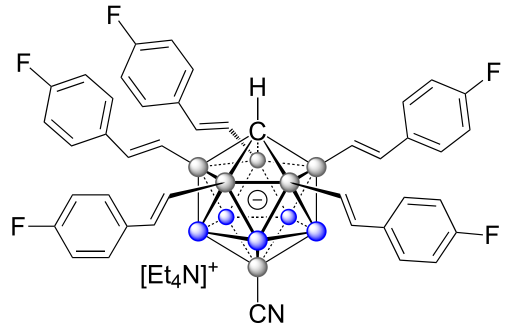

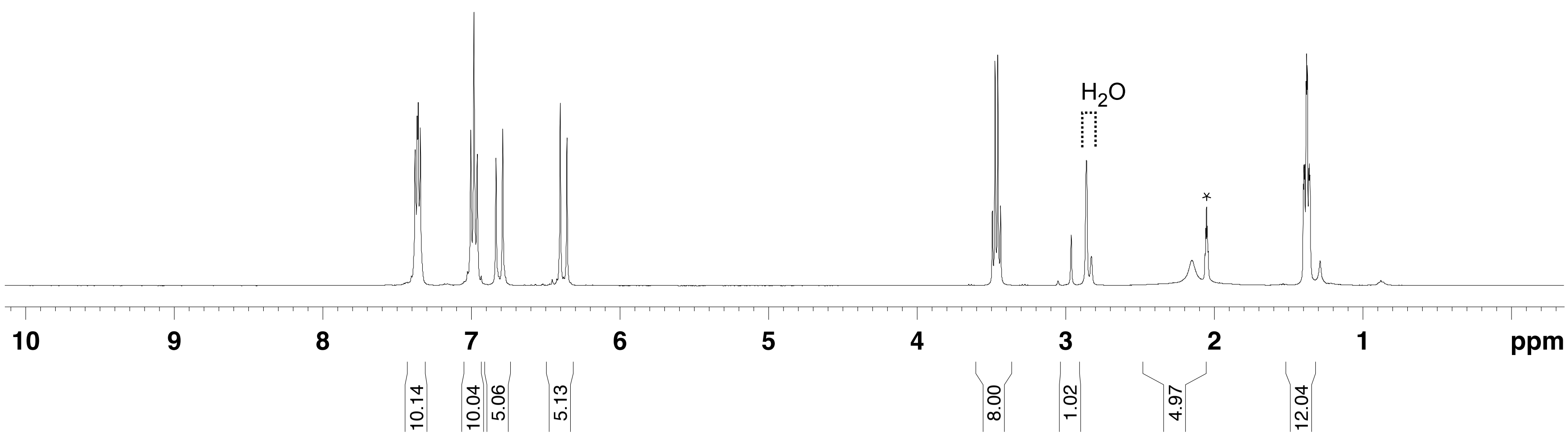


```
Current Data Parameters
NAME     12-CN-penta-4-F-styrene-de
EXPNO                    1
PROCNO                   1

F2 - Acquisition Parameters
Date_            20180611
Time                 8.28
INSTRUM             spect
PROBHD   5 mm PABBO BB/
PULPROG            zgig30
TD                  16384
SOLVENT           Acetone
NS                     16
DS                      4
SWH              8012.820 Hz
FIDRES           0.489064 Hz
AQ              1.0223616 sec
RG                  107.6
DW                 62.400 usec
DE                   6.50 usec
TE                  294.1 K
D1             1.00000000 sec
D11            0.03000000 sec
TD0                     1

======== CHANNEL f1 ========
NUC1                   1H
P1                  15.00 usec
PLW1          12.50000000 W
SFO1          400.1320007 MHz

======== CHANNEL f2 ========
CPDPRG[2            garp4
NUC2                  11B
PCPD2               90.00 usec
PLW2          52.96599960 W
PLW12          0.64477998 W
SFO2          128.3776050 MHz

F2 - Processing parameters
SI                  32768
SF            400.1300072 MHz
WDW                    EM
SSB      0
LB                   1.00 Hz
GB       0
PC                   1.40
```

12-CN-Penta-F-styrine-de product 40 mg in 0.6 ml acetone-d6
11B NMR, 128 MHz, 23 C

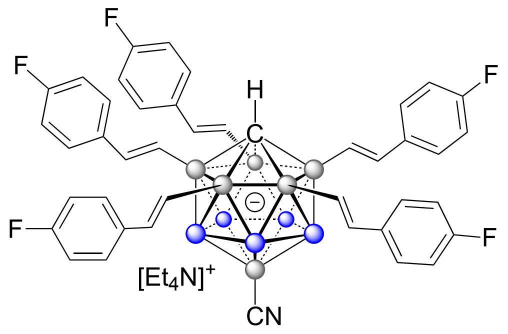

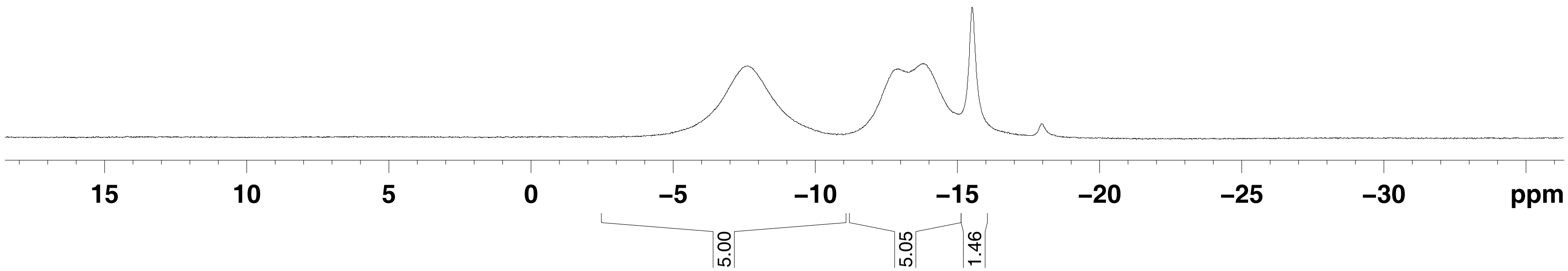


```
Current Data Parameters
NAME     12-CN-penta-4-F-styrene-de
EXPNO                     2
PROCNO                    1

F2 - Acquisition Parameters
Date_          20180611
Time               8.34
INSTRUM           spect
PROBHD   5 mm PABBO BB/
PULPROG              zg
TD                65536
SOLVENT         Acetone
NS                  128
DS                    4
SWH           25510.203 Hz
FIDRES         0.389255 Hz
AQ            1.2845056 sec
RG               193.34
DW               19.600 usec
DE                 6.50 usec
TE                293.0 K
D1           1.00000000 sec
TD0                   1

======== CHANNEL f1 ========
NUC1                11B
P1                 9.93 usec
PLW1        52.96599960 W
SFO1        128.3776052 MHz

F2 - Processing parameters
SI                32768
SF          128.3776050 MHz
WDW                  EM
SSB      0
LB                 2.00 Hz
GB       0
PC                 1.40
```

12–CN–Penta–F–styrine–de product 40 mg in 0.6 ml acetone–d6
11B{1H} NMR, 128 MHz, 23 C

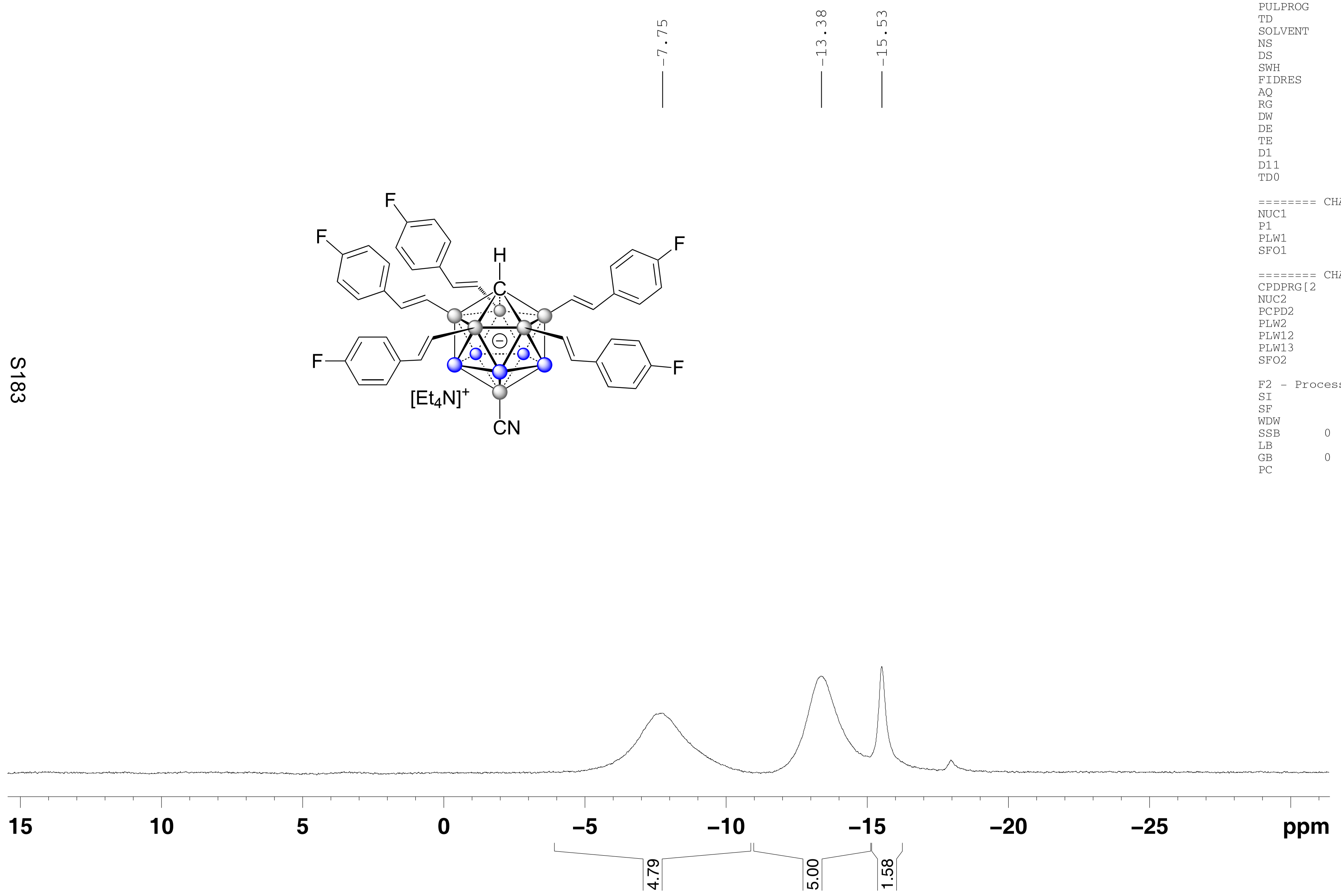


```
Current Data Parameters
NAME      12-CN-penta-4-F-styrene-de
EXPNO                    3
PROCNO                   1

F2 - Acquisition Parameters
Date_          20180611
Time               8.40
INSTRUM           spect
PROBHD   5 mm PABBO BB/
PULPROG          zgpg30
TD                65536
SOLVENT         Acetone
NS                  128
DS                    4
SWH           25510.203 Hz
FIDRES         0.389255 Hz
AQ            1.2845056 sec
RG               193.34
DW               19.600 usec
DE                 6.50 usec
TE                294.4 K
D1           1.00000000 sec
D11          0.03000000 sec
TD0                   1

======== CHANNEL f1 ========
NUC1                11B
P1                 9.93 usec
PLW1        52.96599960 W
SFO1        128.3776050 MHz

======== CHANNEL f2 ========
CPDPRG[2        waltz16
NUC2                 1H
PCPD2             80.00 usec
PLW2        12.50000000 W
PLW12        0.43945000 W
PLW13        0.28125000 W
SFO2        400.1320007 MHz

F2 - Processing parameters
SI                32768
SF          128.3776050 MHz
WDW                  EM
SSB      0
LB                 3.00 Hz
GB       0
PC                 1.40
```

12–CN–Penta–F–styrine–de product 40 mg in 0.6 ml acetone–d6*
13C{1H} NMR, 101 MHz, 23 C

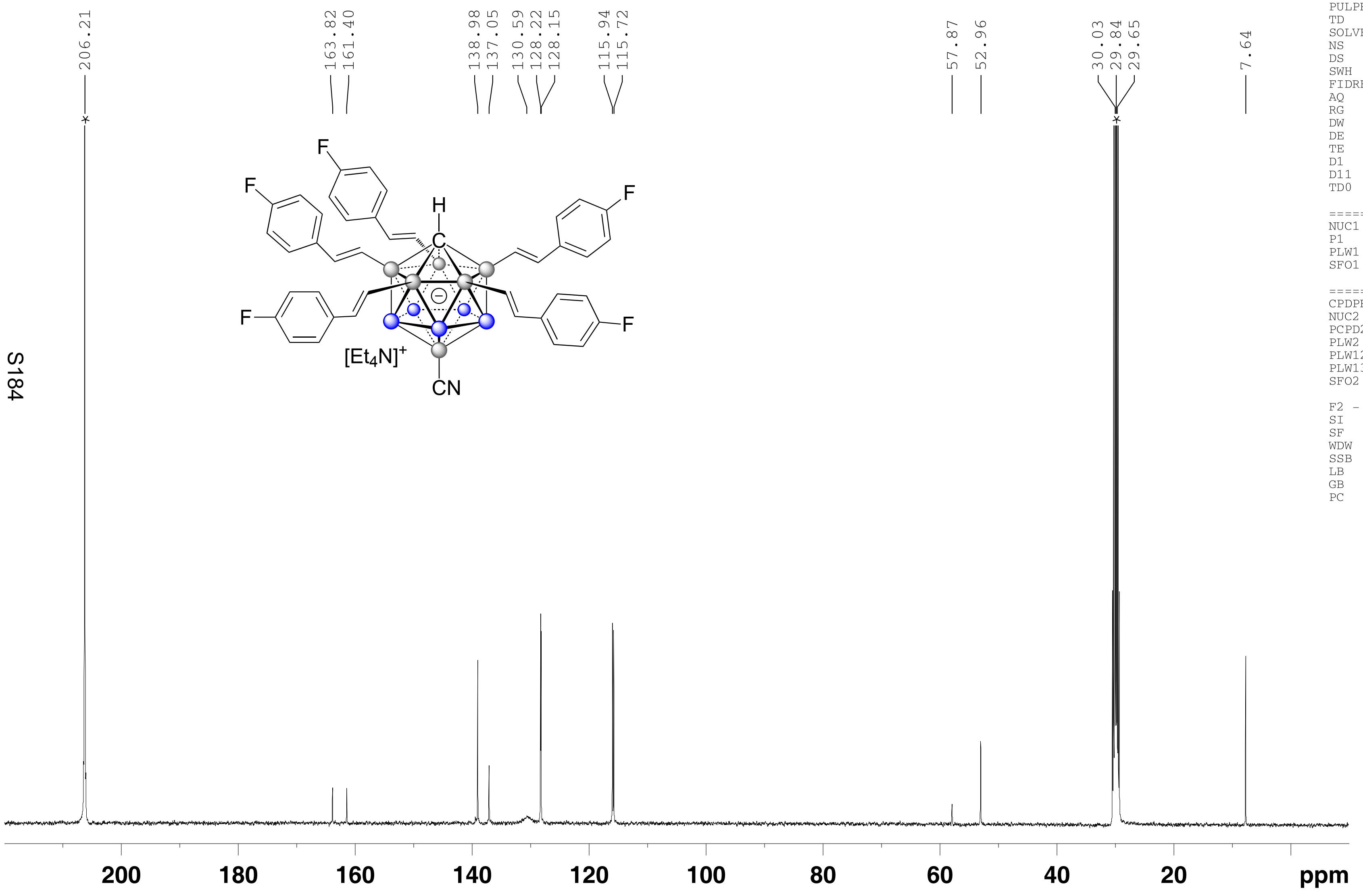


```
Current Data Parameters
NAME     12-CN-penta-4-F-styrene-de
EXPNO                 4
PROCNO                1

F2 - Acquisition Parameters
Date_          20180611
Time              10.12
INSTRUM           spect
PROBHD   5 mm PABBO BB/
PULPROG         zgpg30
TD                65536
SOLVENT         Acetone
NS                 2048
DS                    4
SWH          29761.904 Hz
FIDRES        0.454131 Hz
AQ           1.1010048 sec
RG               193.34
DW               16.800 usec
DE                 6.50 usec
TE                293.9 K
D1           1.50000000 sec
D11          0.03000000 sec
TD0                   1

======== CHANNEL f1 ========
NUC1                13C
P1                10.00 usec
PLW1        53.00000000 W
SFO1        100.6228293 MHz

======== CHANNEL f2 ========
CPDPRG[2        waltz16
NUC2                 1H
PCPD2             80.00 usec
PLW2        12.50000000 W
PLW12        0.43945000 W
PLW13        0.28125000 W
SFO2        400.1316005 MHz

F2 - Processing parameters
SI                32768
SF          100.6126819 MHz
WDW                  EM
SSB      0
LB                 3.00 Hz
GB       0
PC                 1.40
```

penta–R–penten–ol–de product, 30 mg in 0.6 ml acetone–d6*
1H{11B} NMR, 500 MHz, 23 C

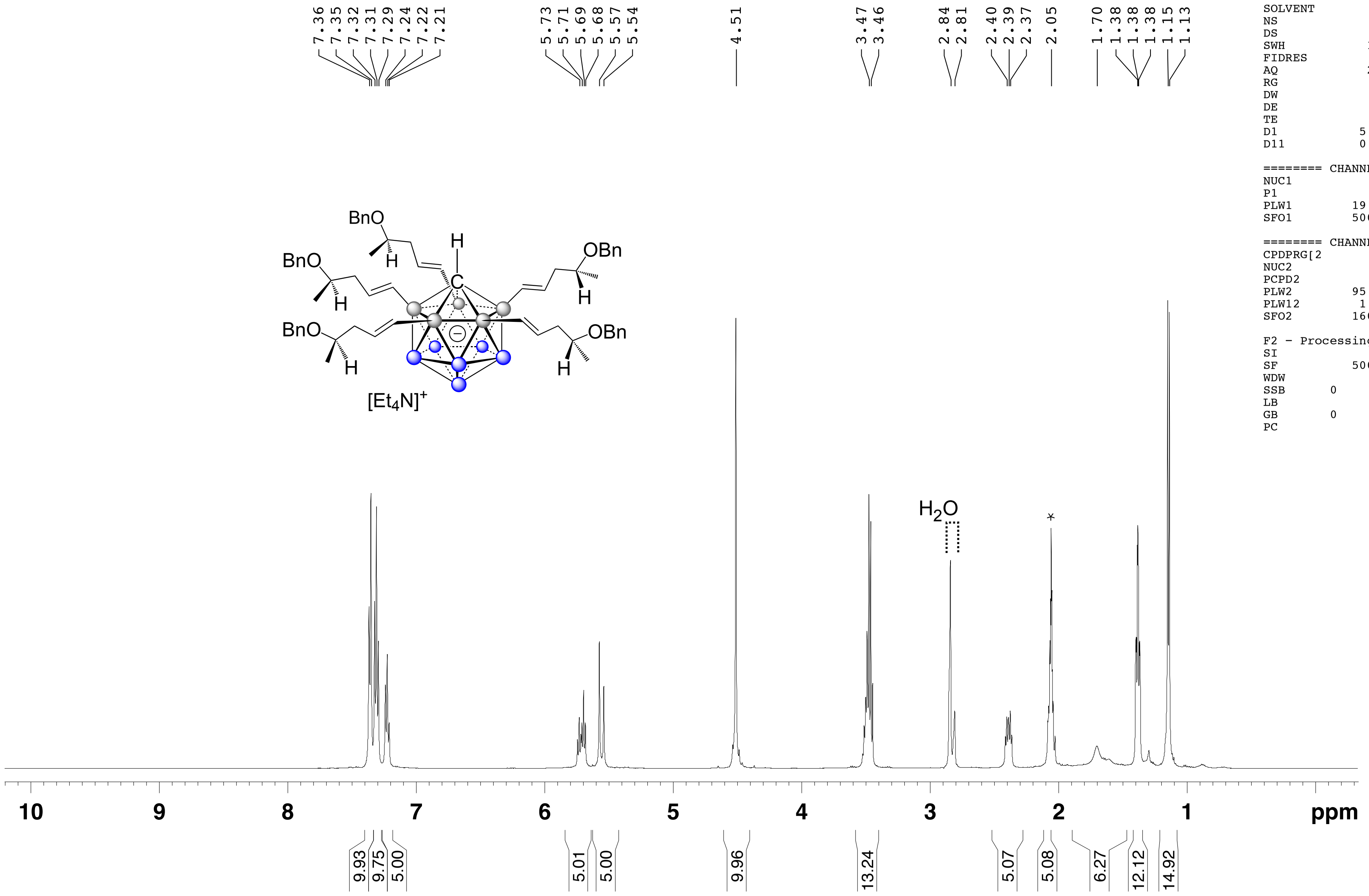


```
Current Data Parameters
NAME      penta-R-penten-ol-de
EXPNO                    6
PROCNO                   1

F2 - Acquisition Parameters
Date_           20180704
Time               20.03
INSTRUM            spect
PROBHD   5 mm PABBO BB-
PULPROG           zgig30
TD                 65536
SOLVENT          Acetone
NS                    16
DS                     0
SWH            12500.000 Hz
FIDRES          0.190735 Hz
AQ             2.6214399 sec
RG                   114
DW                40.000 usec
DE                  6.50 usec
TE                 295.7 K
D1            5.00000000 sec
D11           0.03000000 sec

======== CHANNEL f1 ========
NUC1                  1H
P1                 11.70 usec
PLW1         19.00000000 W
SFO1         500.1335009 MHz

======== CHANNEL f2 ========
CPDPRG[2            garp
NUC2                 11B
PCPD2             100.00 usec
PLW2         95.00000000 W
PLW12         1.63030005 W
SFO2         160.4615690 MHz

F2 - Processing parameters
SI                 65536
SF           500.1300080 MHz
WDW                   EM
SSB      0
LB                  1.00 Hz
GB       0
PC                  1.00
```

penta–R–penten–ol–de product, 30 mg in 0.6 ml acetone–d6
11B NMR, 160 MHz, 23 C

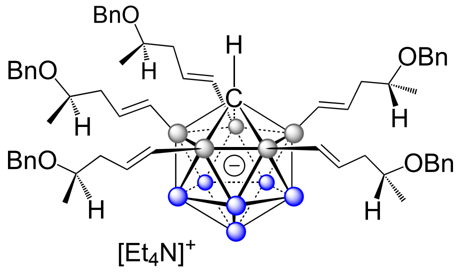

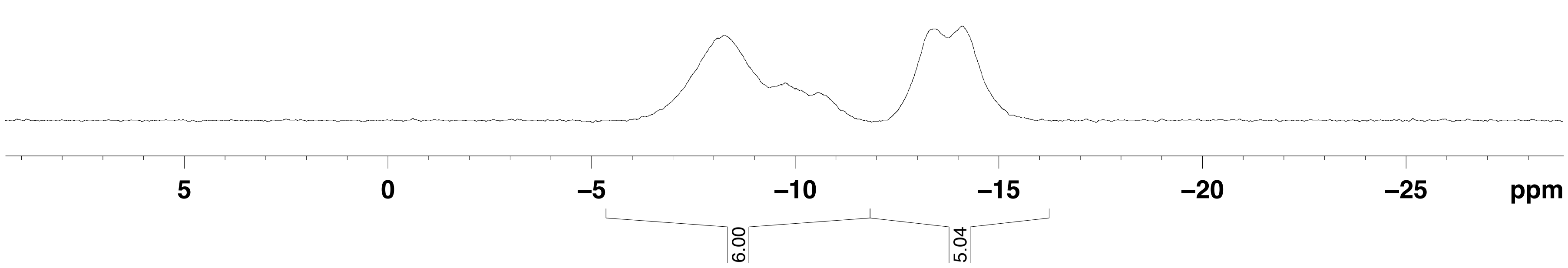


```
Current Data Parameters
NAME     penta-R-penten-ol-de
EXPNO                       2
PROCNO                      1

F2 - Acquisition Parameters
Date_           20180704
Time               13.14
INSTRUM            spect
PROBHD   5 mm PABBO BB-
PULPROG             zg30
TD                 64098
SOLVENT          Acetone
NS                   128
DS                     0
SWH            32051.281 Hz
FIDRES          0.500036 Hz
AQ             0.9999288 sec
RG                   203
DW                15.600 usec
DE                  6.50 usec
TE                 295.5 K
D1            1.00000000 sec

======== CHANNEL f1 ========
NUC1                 11B
P1                 13.10 usec
PLW1         95.00000000 W
SFO1         160.4615792 MHz

F2 - Processing parameters
SI                 32768
SF           160.4615790 MHz
WDW                   EM
SSB      0
LB                 10.00 Hz
GB       0
PC                  1.40
```

penta-R-penten-ol-de product, 30 mg in 0.6 ml acetone-d6
11B{1H} NMR, 160 MHz, 23 C

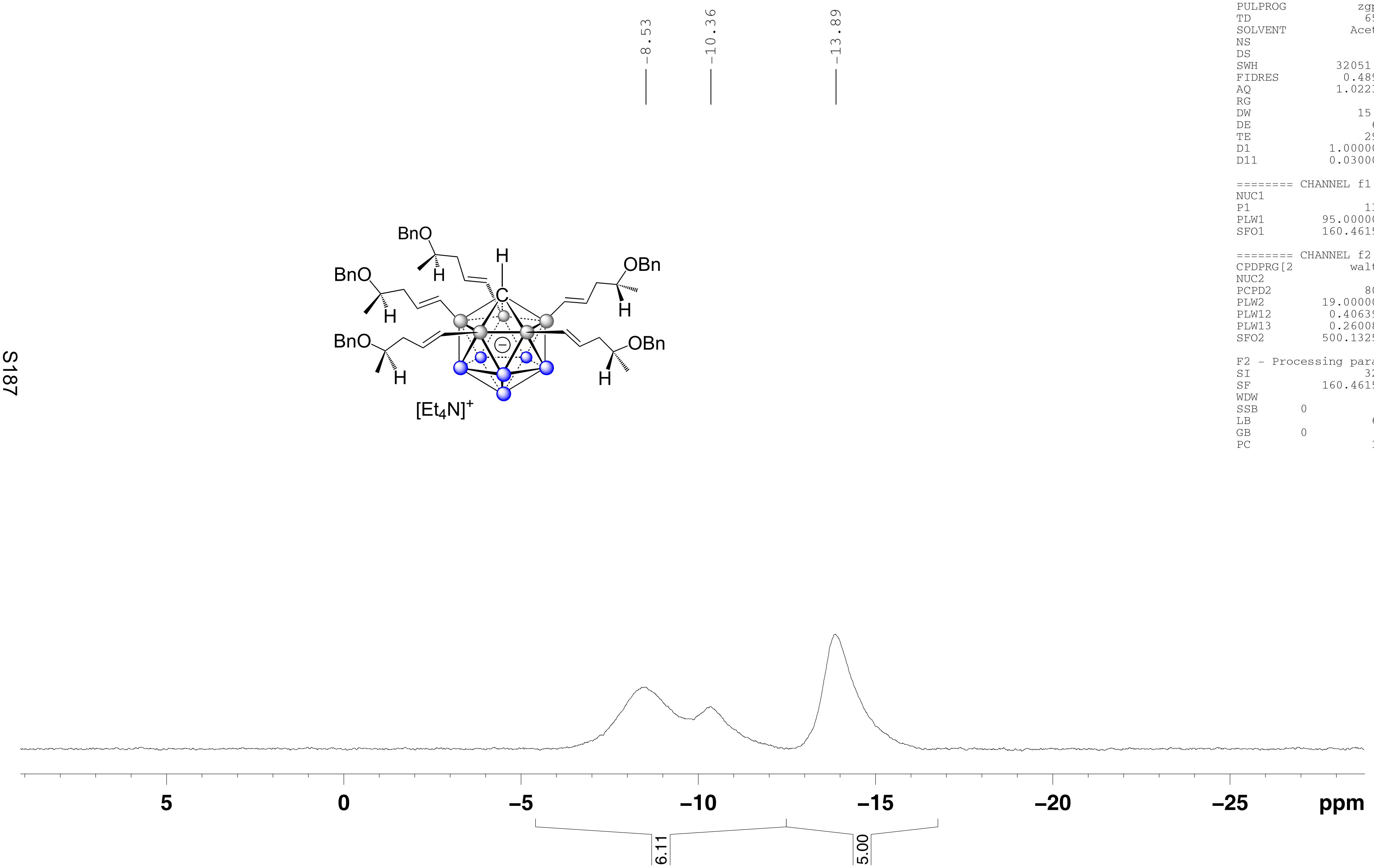


```
Current Data Parameters
NAME     penta-R-penten-ol-de
EXPNO                       3
PROCNO                      1

F2 - Acquisition Parameters
Date_           20180704
Time               13.23
INSTRUM            spect
PROBHD   5 mm PABBO BB-
PULPROG           zgpg30
TD                 65536
SOLVENT          Acetone
NS                   128
DS                     0
SWH            32051.281 Hz
FIDRES          0.489064 Hz
AQ             1.0223616 sec
RG                   203
DW                15.600 usec
DE                  6.50 usec
TE                 295.8 K
D1            1.00000000 sec
D11           0.03000000 sec

======== CHANNEL f1 ========
NUC1                 11B
P1                 13.10 usec
PLW1         95.00000000 W
SFO1         160.4615790 MHz

======== CHANNEL f2 ========
CPDPRG[2         waltz16
NUC2                  1H
PCPD2              80.00 usec
PLW2         19.00000000 W
PLW12         0.40639001 W
PLW13         0.26008999 W
SFO2         500.1325007 MHz

F2 - Processing parameters
SI                 32768
SF           160.4615790 MHz
WDW                   EM
SSB      0
LB                  6.00 Hz
GB       0
PC                  1.40
```

penta-R-penten-ol-de product, 30 mg in 0.6 ml acetone-d6 *
13C{1H} NMR, 126 MHz, 23 C

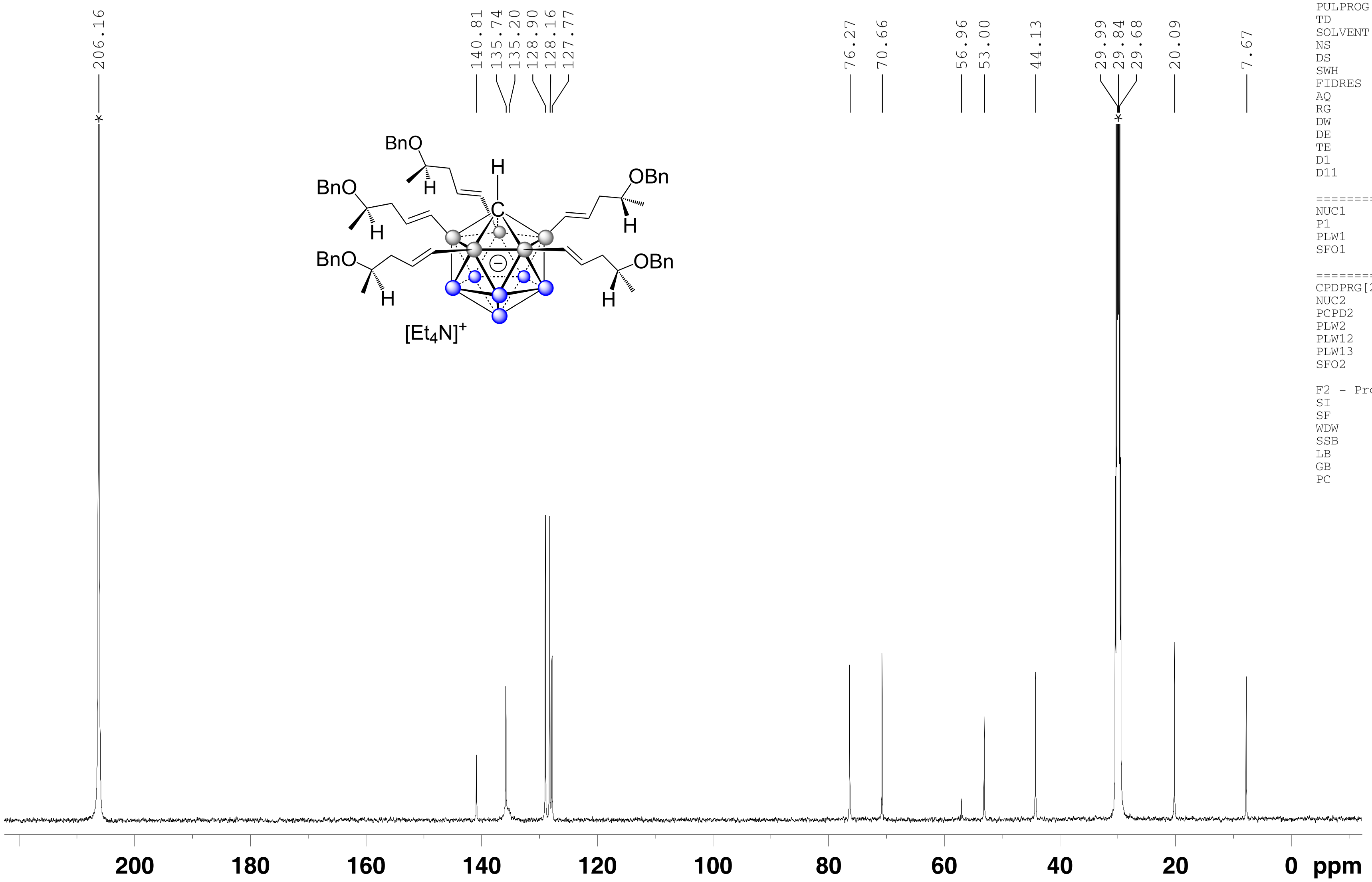


```
Current Data Parameters
NAME       penta-R-penten-ol-de
EXPNO                         4
PROCNO                        1

F2 - Acquisition Parameters
Date_          20180704
Time              15.22
INSTRUM           spect
PROBHD   5 mm PABBO BB-
PULPROG         zgpg30
TD               65536
SOLVENT         Acetone
NS                3000
DS                   4
SWH         37878.789 Hz
FIDRES       0.577984 Hz
AQ          0.8650752 sec
RG                 203
DW              13.200 usec
DE                6.50 usec
TE               296.5 K
D1          1.50000000 sec
D11         0.03000000 sec

======== CHANNEL f1 ========
NUC1                13C
P1                10.50 usec
PLW1        95.00000000 W
SFO1        125.7716224 MHz

======== CHANNEL f2 ========
CPDPRG[2         waltz16
NUC2                 1H
PCPD2             80.00 usec
PLW2        19.00000000 W
PLW12        0.40639001 W
PLW13        0.26008999 W
SFO2        500.1320005 MHz

F2 - Processing parameters
SI                32768
SF          125.7576794 MHz
WDW                  EM
SSB      0
LB                 6.00 Hz
GB       0
PC                 1.40
```

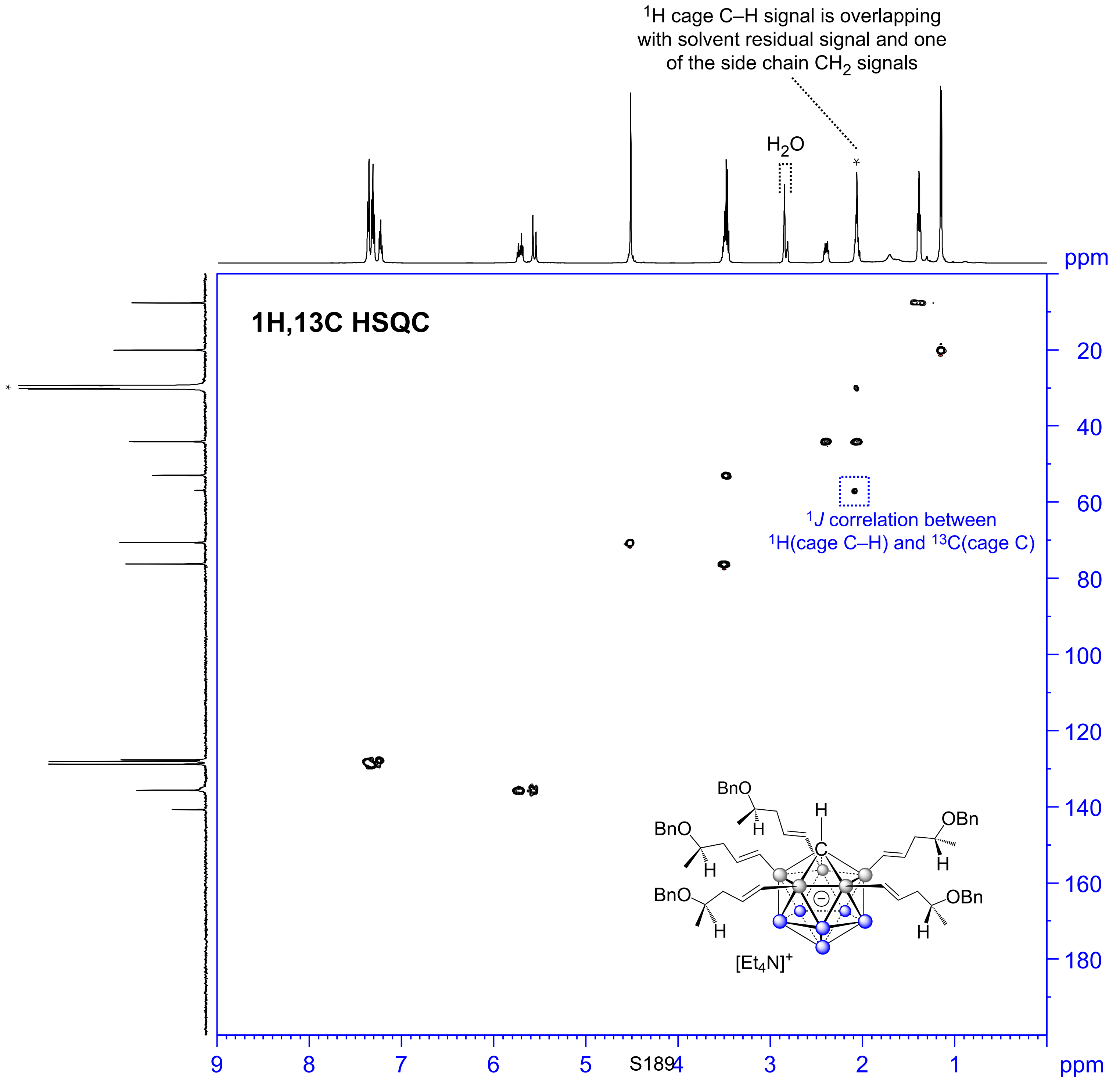

Current Data Parameters
NAME 20180730-chide-hsqc
EXPNO 4
PROCNO 1

F2 - Acquisition Parameters
Date_ 20180731
Time 7.17
INSTRUM spect
PROBHD 5 mm PABBO BB/
PULPROG hsqcetgpsi2
TD 1024
SOLVENT Acetone
NS 2
DS 16
SWH 6009.615 Hz
FIDRES 5.868765 Hz
AQ 0.0851968 sec
RG 193.34
DW 83.200 usec
DE 6.50 usec
TE 295.1 K
CNST2 145.0000000
D0 0.00000300 sec
D1 1.50000000 sec
D4 0.00172414 sec
D11 0.03000000 sec
D16 0.00020000 sec
D24 0.00086207 sec
IN0 0.00001990 sec
ZGOPTNS

======== CHANNEL f1 ========
NUC1 1H
P1 15.00 usec
P2 30.00 usec
P28 1000.00 usec
PLW1 12.50000000 W
SFO1 400.1328009 MHz

======== CHANNEL f2 ========
CPDPRG[2 garp
NUC2 13C
P3 10.00 usec
P4 20.00 usec
PCPD2 70.00 usec
PLW2 53.00000000 W
PLW12 1.08159995 W
SFO2 100.6238364 MHz

====== GRADIENT CHANNEL =====
GPNAM[1] SMSQ10.100
GPNAM[2] SMSQ10.100
GPNAM[3] SMSQ10.100
GPNAM[4] SMSQ10.100
GPZ1 80.00 %
GPZ2 20.10 %
GPZ3 11.00 %
GPZ4 -5.00 %
P16 1000.00 usec
P19 600.00 usec

F1 - Acquisition parameters
TD 256
SFO1 100.6238 MHz
FIDRES 196.524048 Hz
SW 249.991 ppm
FnMODE Echo-Antiecho

F2 - Processing parameters
SI 1024
SF 400.1300000 MHz
WDW QSINE
SSB 2
LB 0 Hz
GB 0
PC 1.40

F1 - Processing parameters
SI 1024
MC2 echo-antiecho
SF 100.6127690 MHz
WDW QSINE
SSB 2
LB 0 Hz
GB 0

penta–S–penten–ol–de product, 30 mg in 0.6 ml acetone–d6*
1H{11B} NMR, 500 MHz, 23 C

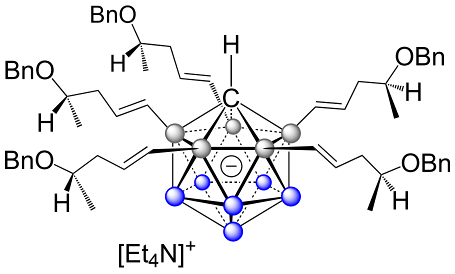

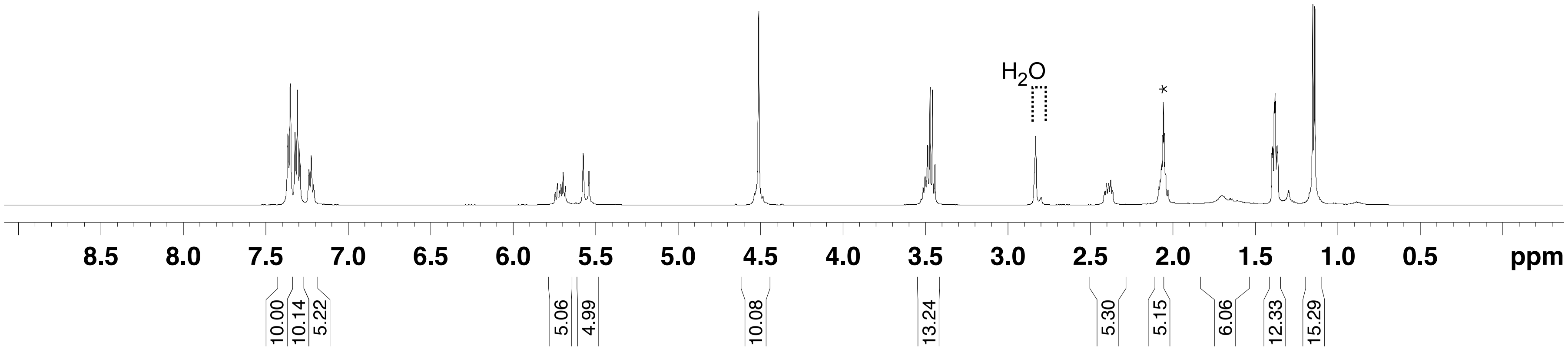


```
Current Data Parameters
NAME     penta-S-penten-ol-de
EXPNO                   1
PROCNO                  1

F2 - Acquisition Parameters
Date_          20181025
Time              10.22
INSTRUM           spect
PROBHD   5 mm PABBO BB-
PULPROG         zgig30
TD                65536
SOLVENT         Acetone
NS                   16
DS                    0
SWH          12500.000 Hz
FIDRES        0.190735 Hz
AQ           2.6214399 sec
RG                  114
DW              40.000 usec
DE                6.50 usec
TE               296.6 K
D1          5.00000000 sec
D11         0.03000000 sec

======== CHANNEL f1 ========
NUC1                 1H
P1                11.70 usec
PLW1        19.00000000 W
SFO1       500.1335009 MHz

======== CHANNEL f2 ========
CPDPRG[2           garp
NUC2                11B
PCPD2            100.00 usec
PLW2        95.00000000 W
PLW12        1.63030005 W
SFO2       160.4615690 MHz

F2 - Processing parameters
SI                65536
SF          500.1300079 MHz
WDW                  EM
SSB      0
LB                 1.00 Hz
GB       0
PC                 1.00
```

penta–S–penten–ol–de product, 30 mg in 0.6 ml acetone–d6
11B NMR, 160 MHz, 23 C

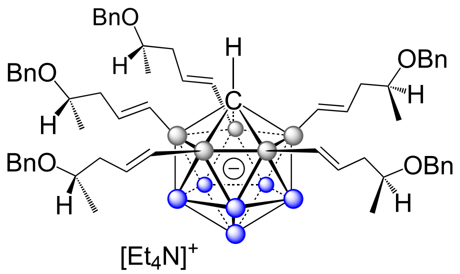

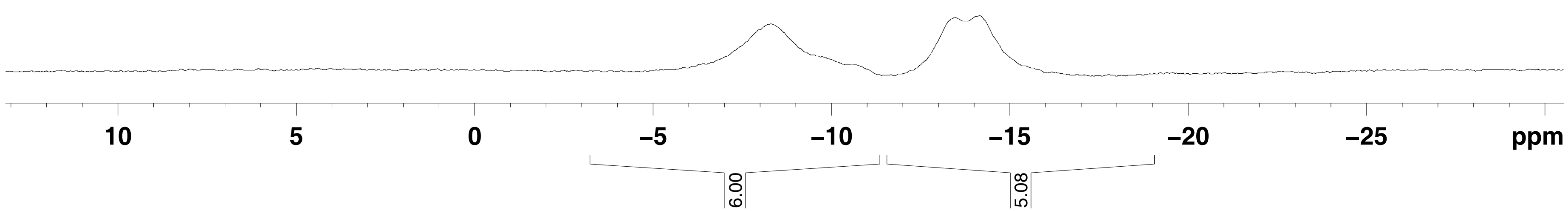


```
Current Data Parameters
NAME      penta-S-penten-ol-de
EXPNO                    2
PROCNO                   1

F2 - Acquisition Parameters
Date_             20181025
Time                 10.25
INSTRUM              spect
PROBHD   5 mm PABBO BB-
PULPROG               zg30
TD                   64098
SOLVENT            Acetone
NS                      64
DS                       0
SWH              32051.281 Hz
FIDRES            0.500036 Hz
AQ               0.9999288 sec
RG                     203
DW                  15.600 usec
DE                    6.50 usec
TE                   296.2 K
D1              1.00000000 sec

======== CHANNEL f1 ========
NUC1                   11B
P1                   13.10 usec
PLW1           95.00000000 W
SFO1           160.4615792 MHz

F2 - Processing parameters
SI                   32768
SF             160.4615790 MHz
WDW                     EM
SSB      0
LB                   10.00 Hz
GB       0
PC                    1.40
```

penta-S-penten-ol-de product, 30 mg in 0.6 ml acetone-d6
11B{1H} NMR, 160 MHz, 23 C

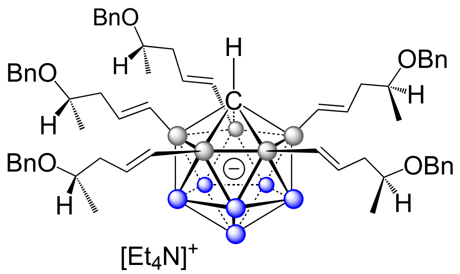

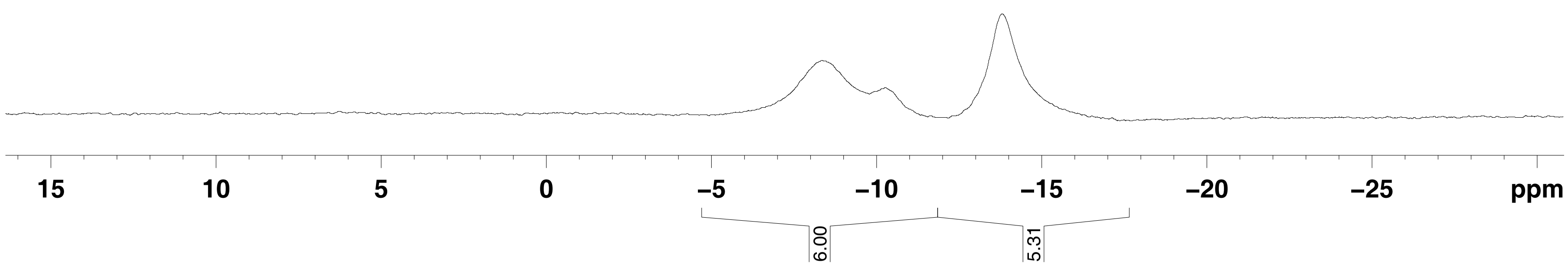


```
Current Data Parameters
NAME      penta-S-penten-ol-de
EXPNO                    3
PROCNO                   1

F2 - Acquisition Parameters
Date_           20181025
Time               10.29
INSTRUM            spect
PROBHD   5 mm PABBO BB-
PULPROG           zgpg30
TD                 65536
SOLVENT          Acetone
NS                    64
DS                     0
SWH            32051.281 Hz
FIDRES          0.489064 Hz
AQ             1.0223616 sec
RG                   203
DW                15.600 usec
DE                  6.50 usec
TE                 296.2 K
D1            1.00000000 sec
D11           0.03000000 sec

======== CHANNEL f1 ========
NUC1                 11B
P1                 13.10 usec
PLW1         95.00000000 W
SFO1        160.4615790 MHz

======== CHANNEL f2 ========
CPDPRG[2         waltz16
NUC2                  1H
PCPD2              80.00 usec
PLW2         19.00000000 W
PLW12         0.40639001 W
PLW13         0.26008999 W
SFO2        500.1325007 MHz

F2 - Processing parameters
SI                 32768
SF          160.4615790 MHz
WDW                   EM
SSB      0
LB                 10.00 Hz
GB       0
PC                  1.40
```

penta-S-penten-ol-de product, 30 mg in 0.6 ml acetone-d6*
13C{1H} NMR, 126 MHz, 23 C

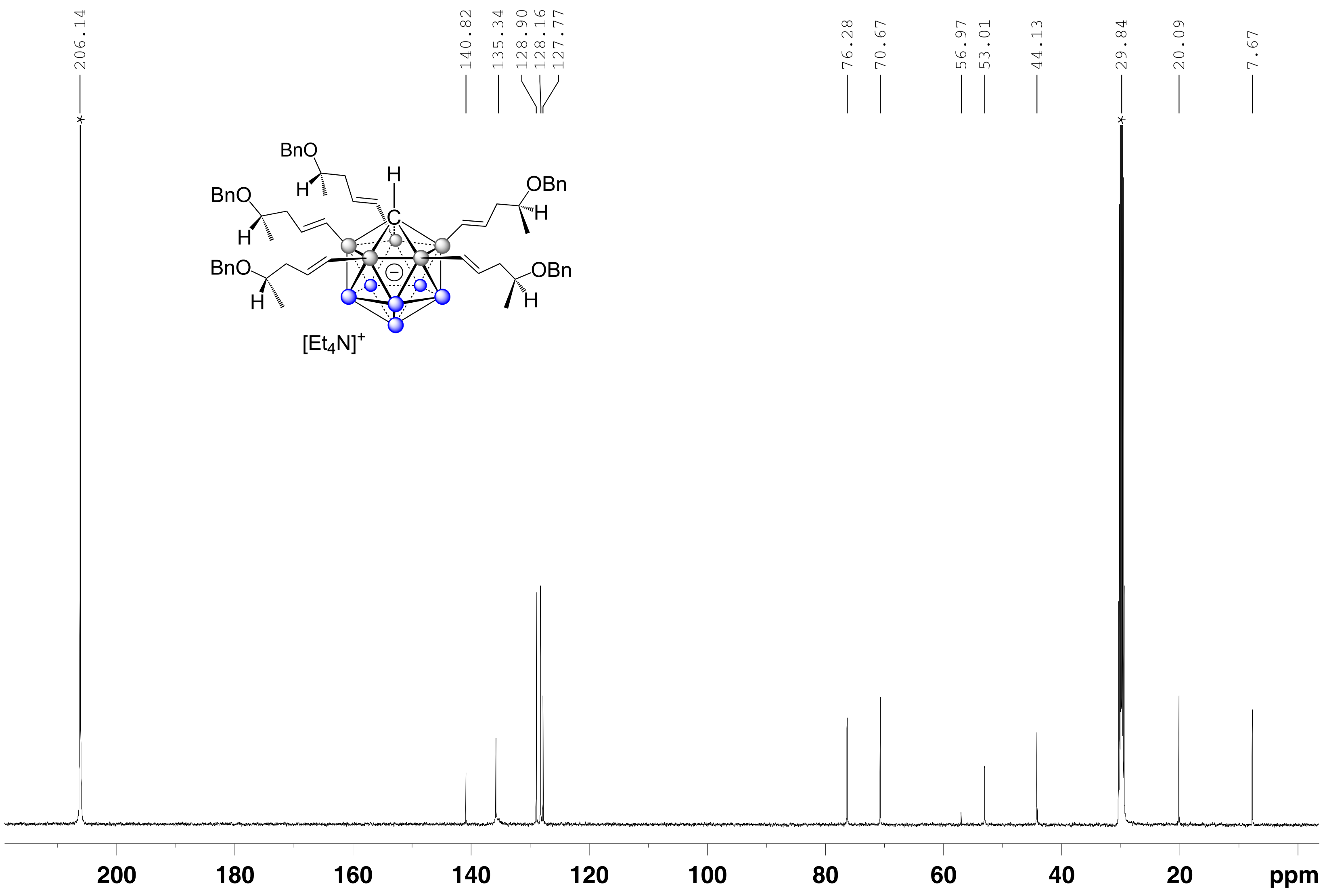


```
Current Data Parameters
NAME     penta-S-penten-ol-de
EXPNO                    4
PROCNO                   1

F2 - Acquisition Parameters
Date_          20181025
Time              11.53
INSTRUM           spect
PROBHD   5 mm PABBO BB-
PULPROG          zgpg30
TD                65536
SOLVENT         Acetone
NS                 2048
DS                    4
SWH          37878.789 Hz
FIDRES        0.577984 Hz
AQ           0.8650752 sec
RG                  203
DW               13.200 usec
DE                 6.50 usec
TE                296.3 K
D1           1.50000000 sec
D11          0.03000000 sec

======== CHANNEL f1 ========
NUC1                13C
P1                10.50 usec
PLW1        95.00000000 W
SFO1        125.7716224 MHz

======== CHANNEL f2 ========
CPDPRG[2        waltz16
NUC2                 1H
PCPD2             80.00 usec
PLW2        19.00000000 W
PLW12        0.40639001 W
PLW13        0.26008999 W
SFO2        500.1320005 MHz

F2 - Processing parameters
SI                32768
SF          125.7576787 MHz
WDW                  EM
SSB      0
LB                 3.00 Hz
GB       0
PC                 1.40
```

penta−4−F−styrene−H product, 50 mg in 0.6 ml acetone−d6*
1H{11B} NMR, 400 MHz, 23 C

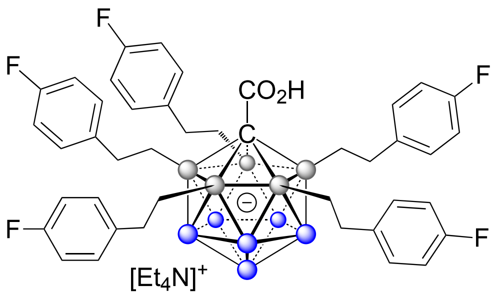

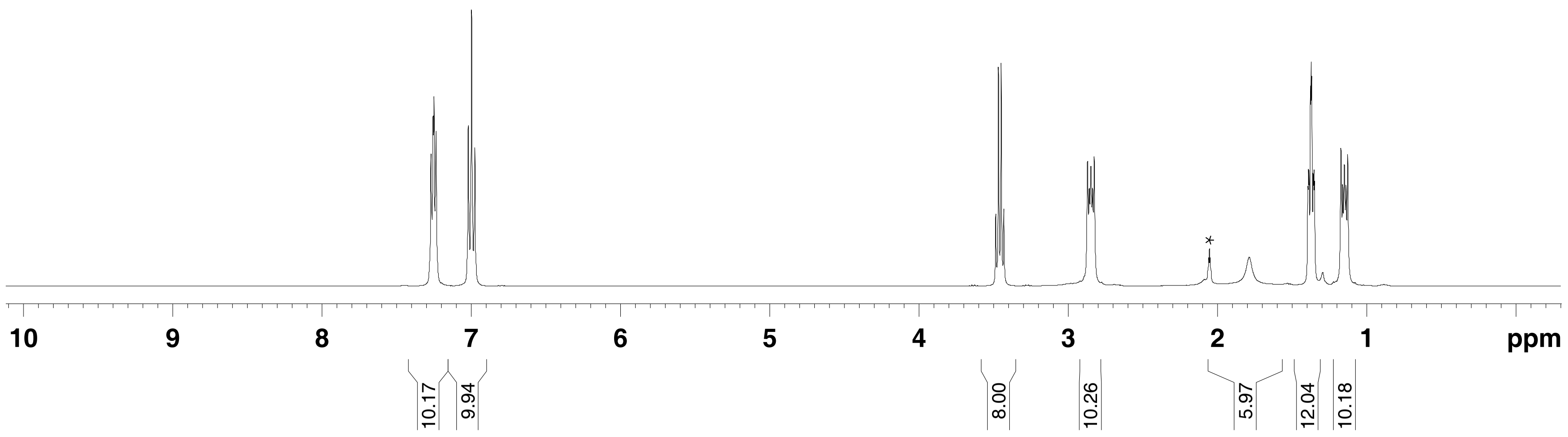


```
Current Data Parameters
NAME     penta-4-F-styrene-H
EXPNO                      2
PROCNO                     1

F2 - Acquisition Parameters
Date_           20180804
Time               16.57
INSTRUM             spect
PROBHD   5 mm PABBO BB/
PULPROG           zgig30
TD                 16384
SOLVENT          Acetone
NS                    16
DS                     4
SWH             8012.820 Hz
FIDRES          0.489064 Hz
AQ             1.0223616 sec
RG                 71.39
DW                62.400 usec
DE                  6.50 usec
TE                 295.2 K
D1            1.00000000 sec
D11           0.03000000 sec
TD0                    1

======== CHANNEL f1 ========
NUC1                  1H
P1                 15.00 usec
PLW1         12.50000000 W
SFO1         400.1320007 MHz

======== CHANNEL f2 ========
CPDPRG[2           garp4
NUC2                 11B
PCPD2              90.00 usec
PLW2         52.96599960 W
PLW12         0.64477998 W
SFO2         128.3776050 MHz

F2 - Processing parameters
SI                 32768
SF           400.1300072 MHz
WDW                   EM
SSB      0
LB                  1.00 Hz
GB       0
PC                  1.40
```

penta-4-F-styrene-H product, 50 mg in 0.6 ml acetone-d6
11B NMR, 128 MHz, 23 C

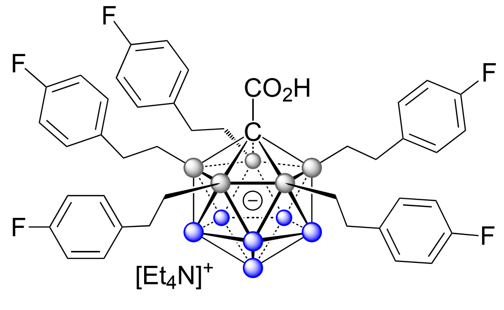


```
Current Data Parameters
NAME     penta-4-F-styrene-H
EXPNO                  3
PROCNO                 1

F2 - Acquisition Parameters
Date_          20180804
Time              17.03
INSTRUM           spect
PROBHD   5 mm PABBO BB/
PULPROG              zg
TD                65536
SOLVENT         Acetone
NS                  128
DS                    4
SWH           25510.203 Hz
FIDRES         0.389255 Hz
AQ            1.2845056 sec
RG               193.34
DW               19.600 usec
DE                 6.50 usec
TE                294.7 K
D1           1.00000000 sec
TD0                   1

======== CHANNEL f1 ========
NUC1                11B
P1                 9.93 usec
PLW1        52.96599960 W
SFO1        128.3776052 MHz

F2 - Processing parameters
SI                32768
SF          128.3776050 MHz
WDW                  EM
SSB      0
LB                 3.00 Hz
GB       0
PC                 1.40
```

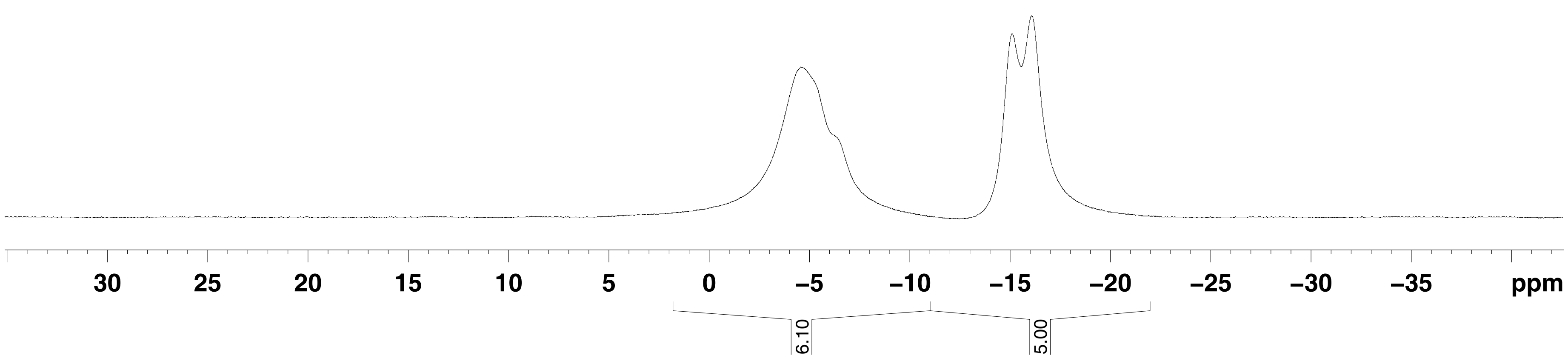

penta-4-F-styrene-H product, 50 mg in 0.6 ml acetone-d6
11B{1H} NMR, 128 MHz, 23 C

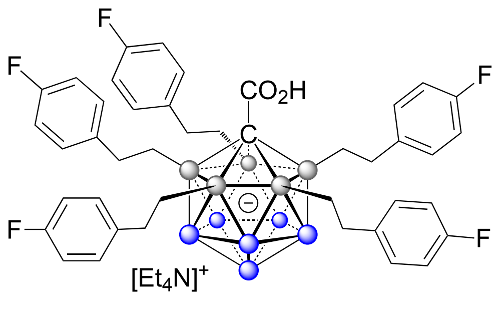

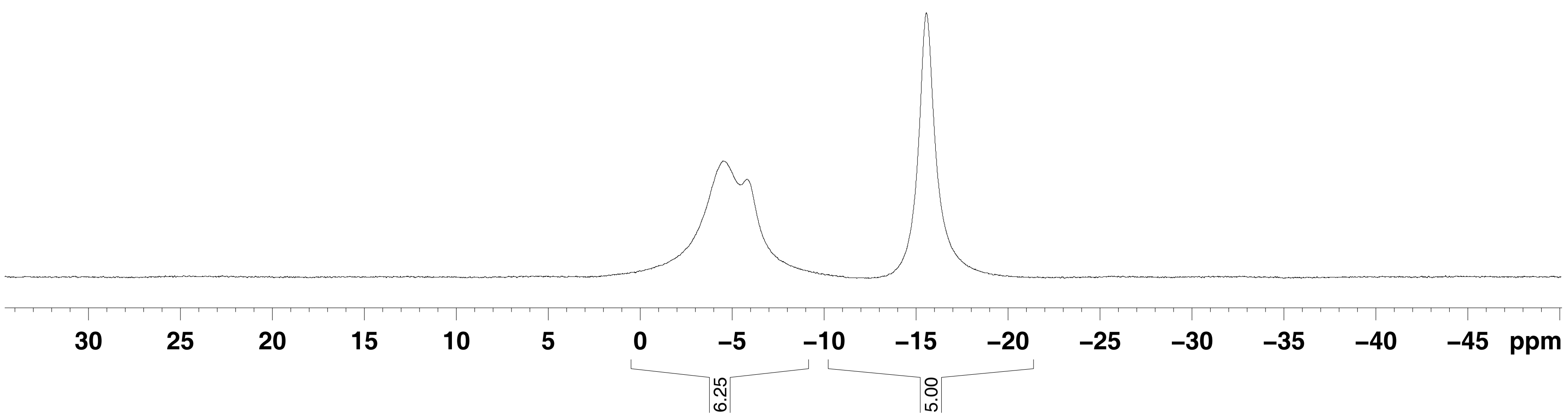


```
Current Data Parameters
NAME     penta-4-F-styrene-H
EXPNO                      4
PROCNO                     1

F2 - Acquisition Parameters
Date_           20180804
Time               17.09
INSTRUM            spect
PROBHD   5 mm PABBO BB/
PULPROG          zgpg30
TD                65536
SOLVENT          Acetone
NS                  128
DS                    4
SWH           25510.203 Hz
FIDRES         0.389255 Hz
AQ            1.2845056 sec
RG               193.34
DW               19.600 usec
DE                 6.50 usec
TE                295.6 K
D1           1.00000000 sec
D11          0.03000000 sec
TD0                   1

======== CHANNEL f1 ========
NUC1                 11B
P1                  9.93 usec
PLW1         52.96599960 W
SFO1        128.3776050 MHz

======== CHANNEL f2 ========
CPDPRG[2         waltz16
NUC2                  1H
PCPD2              80.00 usec
PLW2         12.50000000 W
PLW12         0.43945000 W
PLW13         0.28125000 W
SFO2        400.1320007 MHz

F2 - Processing parameters
SI                 32768
SF           128.3776050 MHz
WDW                   EM
SSB      0
LB                  4.00 Hz
GB       0
PC                  1.40
```

penta−4−F−styrene−H product, 50 mg in 0.6 ml acetone−d6*
13C{1H} NMR, 101 MHz, 23 C

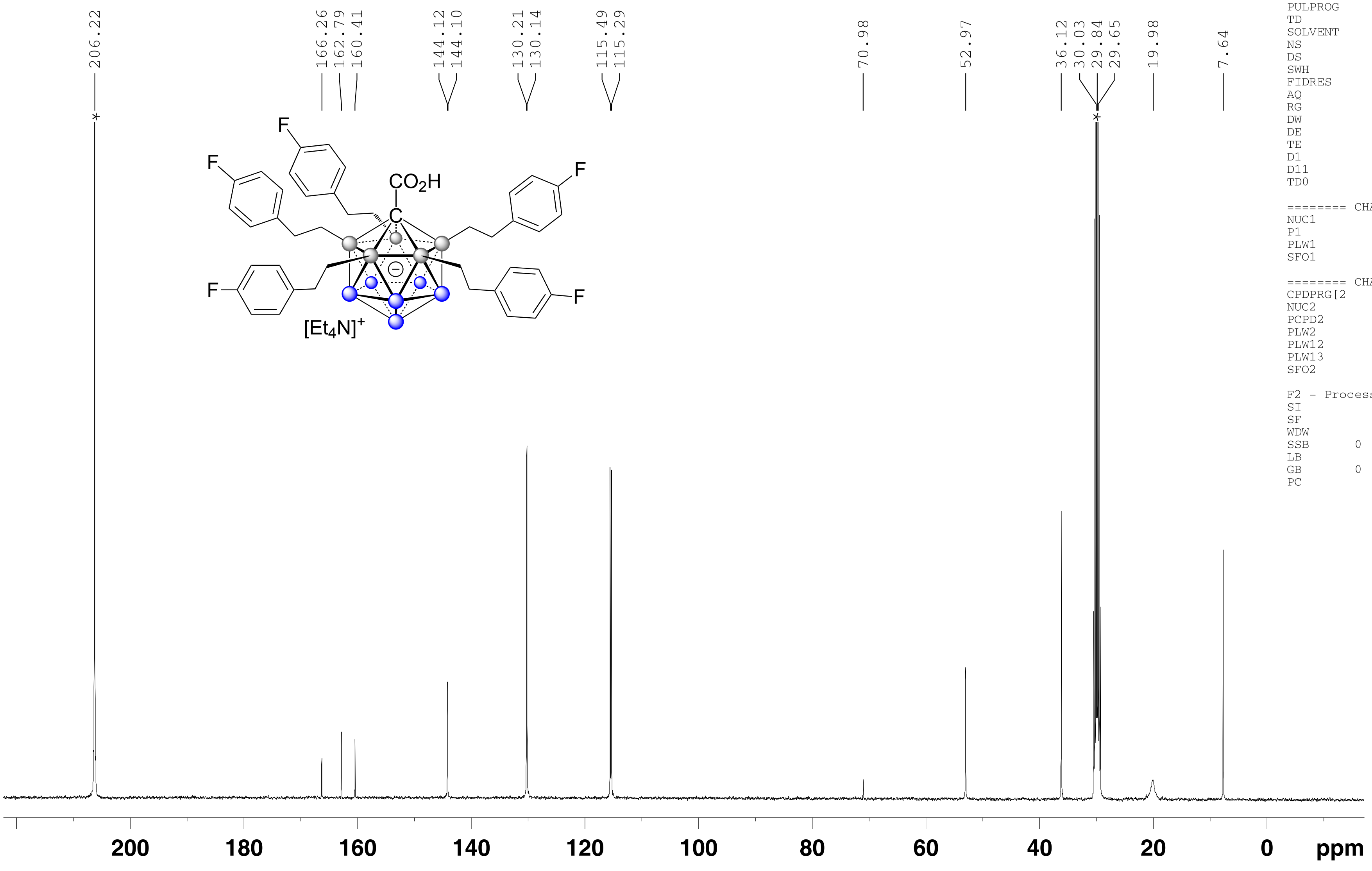


```
Current Data Parameters
NAME     penta-4-F-styrene-H
EXPNO                  5
PROCNO                 1

F2 - Acquisition Parameters
Date_          20180804
Time              18.41
INSTRUM           spect
PROBHD   5 mm PABBO BB/
PULPROG          zgpg30
TD                65536
SOLVENT         Acetone
NS                 2048
DS                    4
SWH          29761.904 Hz
FIDRES        0.454131 Hz
AQ           1.1010048 sec
RG               193.34
DW               16.800 usec
DE                 6.50 usec
TE                295.6 K
D1           1.50000000 sec
D11          0.03000000 sec
TD0                   1

======== CHANNEL f1 ========
NUC1                13C
P1                10.00 usec
PLW1        53.00000000 W
SFO1        100.6228293 MHz

======== CHANNEL f2 ========
CPDPRG[2        waltz16
NUC2                 1H
PCPD2             80.00 usec
PLW2        12.50000000 W
PLW12        0.43945000 W
PLW13        0.28125000 W
SFO2        400.1316005 MHz

F2 - Processing parameters
SI                32768
SF          100.6126829 MHz
WDW                  EM
SSB      0
LB                 3.00 Hz
GB       0
PC                 1.40
```

penta‒4,4‒Dimethylpent‒1‒en‒H product, 30 mg in 0.6 ml acetone‒d6*
1H{11B} NMR, 400 MHz, 23 C

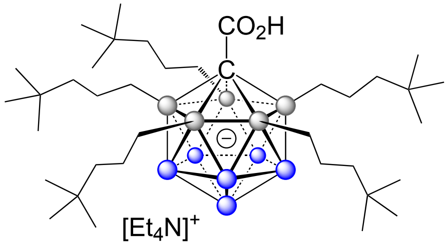

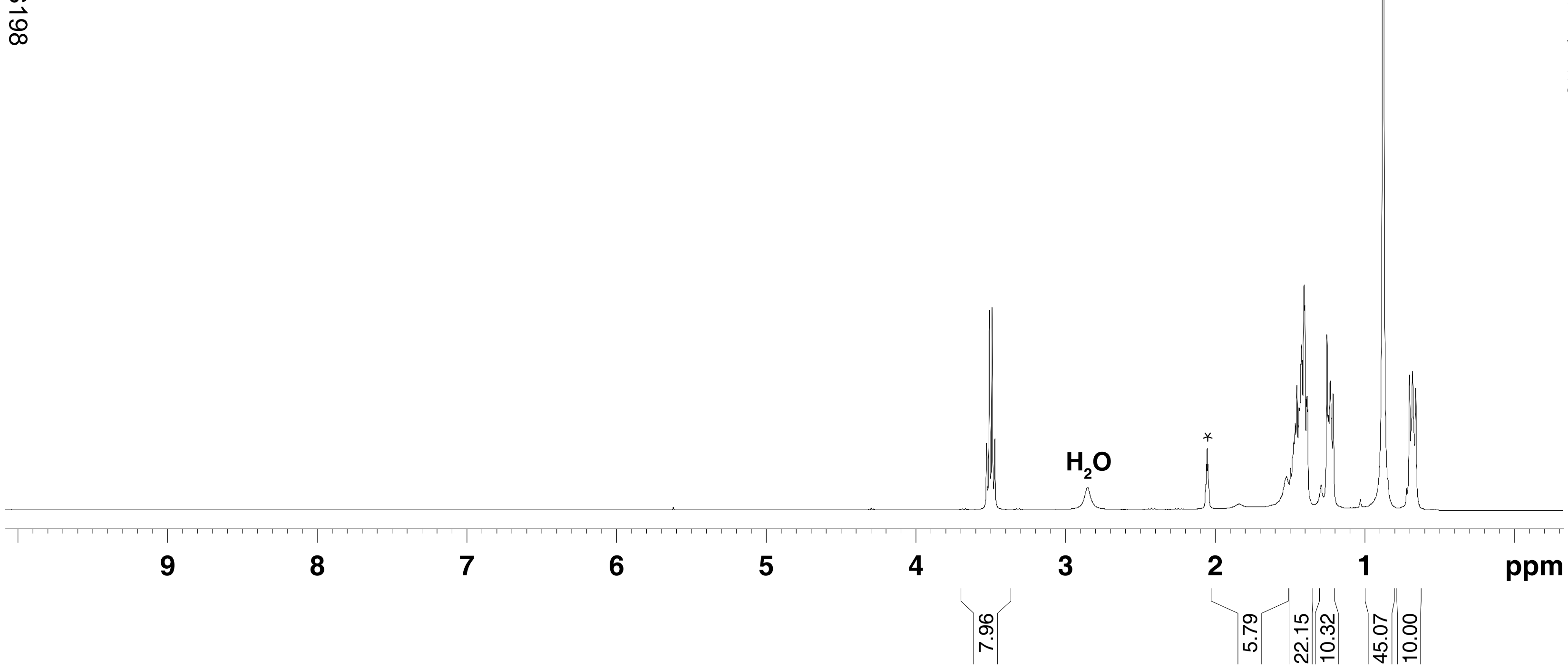


```
Current Data Parameters
NAME     penta-4,4-Dimethylpent-1-e
EXPNO                   2
PROCNO                  1

F2 - Acquisition Parameters
Date_            20180804
Time                14.20
INSTRUM             spect
PROBHD   5 mm PABBO BB/
PULPROG            zgig30
TD                  16384
SOLVENT           Acetone
NS                     16
DS                      4
SWH              8012.820 Hz
FIDRES           0.489064 Hz
AQ              1.0223616 sec
RG                  78.69
DW                 62.400 usec
DE                   6.50 usec
TE                  294.9 K
D1             1.00000000 sec
D11            0.03000000 sec
TD0                     1

======== CHANNEL f1 ========
NUC1                   1H
P1                  15.00 usec
PLW1          12.50000000 W
SFO1          400.1320007 MHz

======== CHANNEL f2 ========
CPDPRG[2            garp4
NUC2                  11B
PCPD2               90.00 usec
PLW2          52.96599960 W
PLW12          0.64477998 W
SFO2          128.3776050 MHz

F2 - Processing parameters
SI                  32768
SF            400.1300072 MHz
WDW                    EM
SSB      0
LB                   1.00 Hz
GB       0
PC                   1.40
```

penta-4,4-Dimethylpent-1-en-H product, 30 mg in 0.6 ml acetone-d6
11B NMR, 128 MHz, 23 C

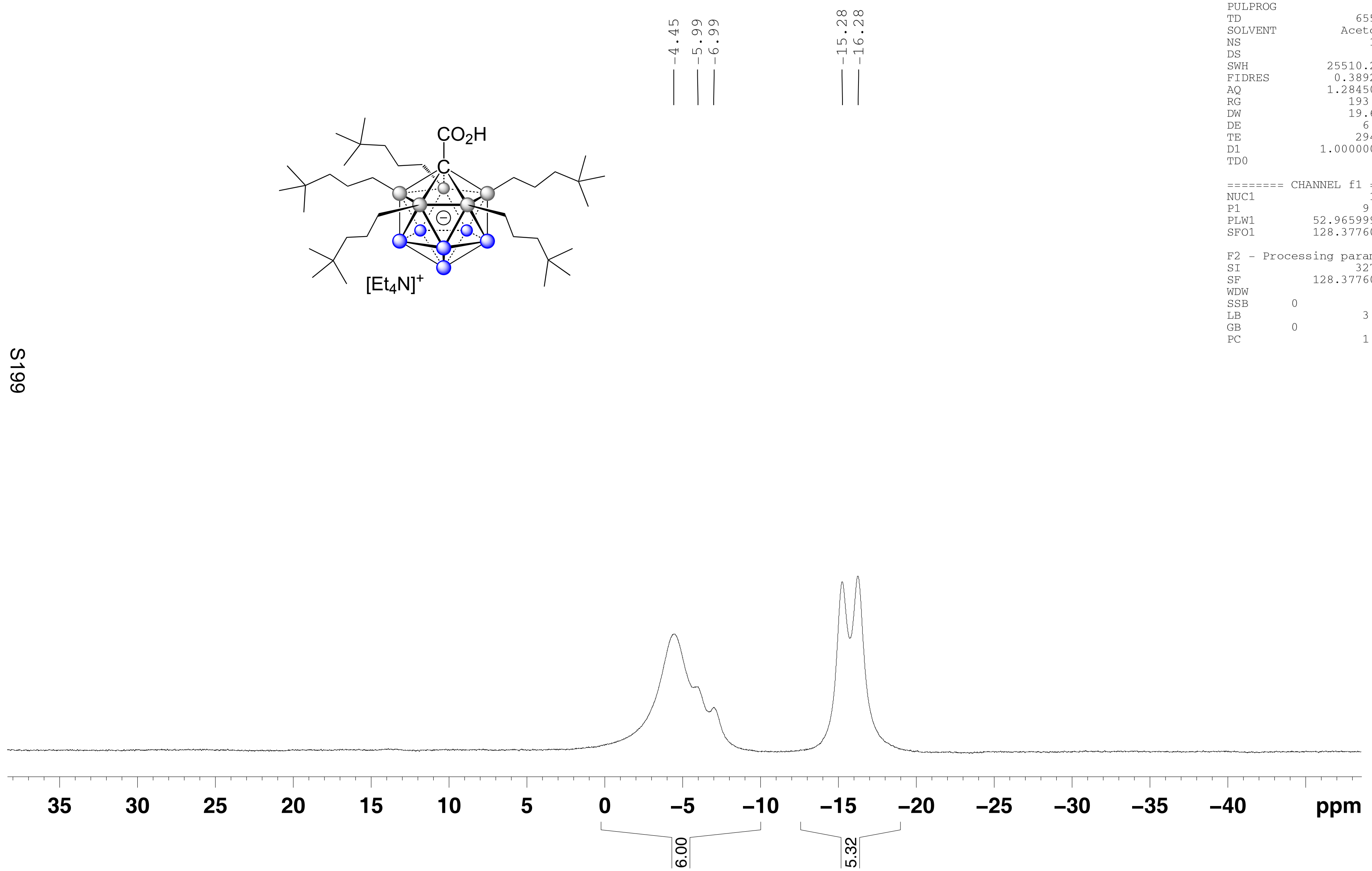


```
Current Data Parameters
NAME      penta-4,4-Dimethylpent-1-e
EXPNO                     3
PROCNO                    1

F2 - Acquisition Parameters
Date_              20180804
Time                  14.26
INSTRUM               spect
PROBHD   5 mm PABBO BB/
PULPROG                  zg
TD                    65536
SOLVENT             Acetone
NS                      128
DS                        4
SWH               25510.203 Hz
FIDRES             0.389255 Hz
AQ                1.2845056 sec
RG                   193.34
DW                   19.600 usec
DE                     6.50 usec
TE                    294.8 K
D1               1.00000000 sec
TD0                       1

======== CHANNEL f1 ========
NUC1                    11B
P1                     9.93 usec
PLW1            52.96599960 W
SFO1            128.3776052 MHz

F2 - Processing parameters
SI                    32768
SF              128.3776050 MHz
WDW                      EM
SSB      0
LB                     3.00 Hz
GB       0
PC                     1.40
```

penta-4,4-Dimethylpent-1-en-H product, 30 mg in 0.6 ml acetone-d6
11B{1H} NMR, 128 MHz, 23 C

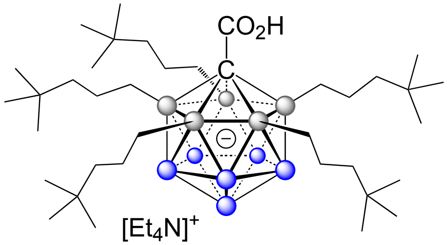

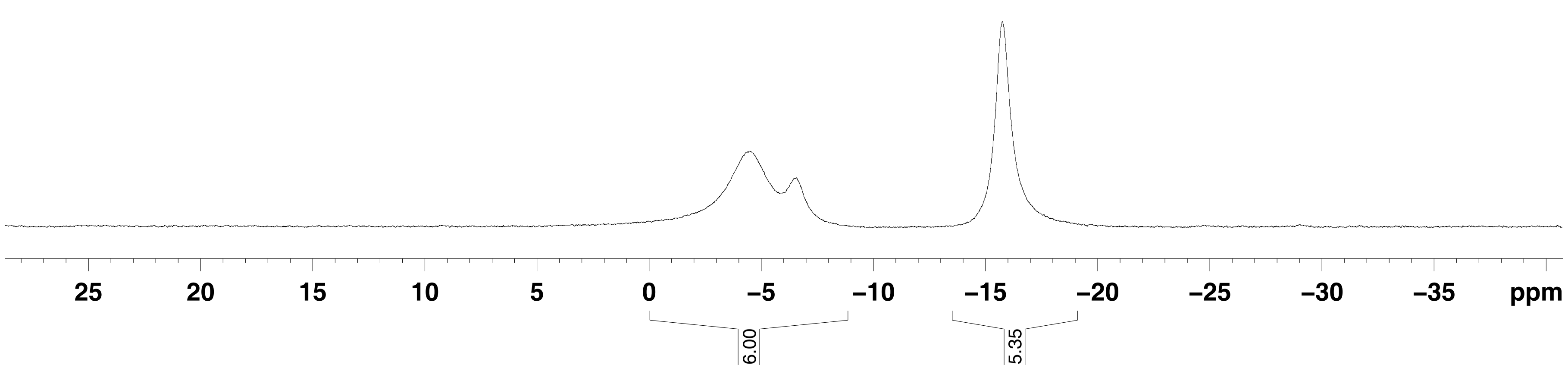


```
Current Data Parameters
NAME     penta-4,4-Dimethylpent-1-e
EXPNO                    4
PROCNO                   1

F2 - Acquisition Parameters
Date_           20180804
Time               14.32
INSTRUM            spect
PROBHD   5 mm PABBO BB/
PULPROG          zgpg30
TD                65536
SOLVENT         Acetone
NS                  128
DS                    4
SWH           25510.203 Hz
FIDRES         0.389255 Hz
AQ            1.2845056 sec
RG               193.34
DW               19.600 usec
DE                 6.50 usec
TE                295.5 K
D1           1.00000000 sec
D11          0.03000000 sec
TD0                   1

======== CHANNEL f1 ========
NUC1                11B
P1                 9.93 usec
PLW1        52.96599960 W
SFO1       128.3776050 MHz

======== CHANNEL f2 ========
CPDPRG[2        waltz16
NUC2                 1H
PCPD2             80.00 usec
PLW2        12.50000000 W
PLW12        0.43945000 W
PLW13        0.28125000 W
SFO2       400.1320007 MHz

F2 - Processing parameters
SI                32768
SF         128.3776050 MHz
WDW                  EM
SSB      0
LB                 4.00 Hz
GB       0
PC                 1.40
```

penta‑4,4‑Dimethylpent‑1‑en‑H product, 30 mg in 0.6 ml acetone‑d6 *
13C{1H} NMR, 101 MHz, 23 C

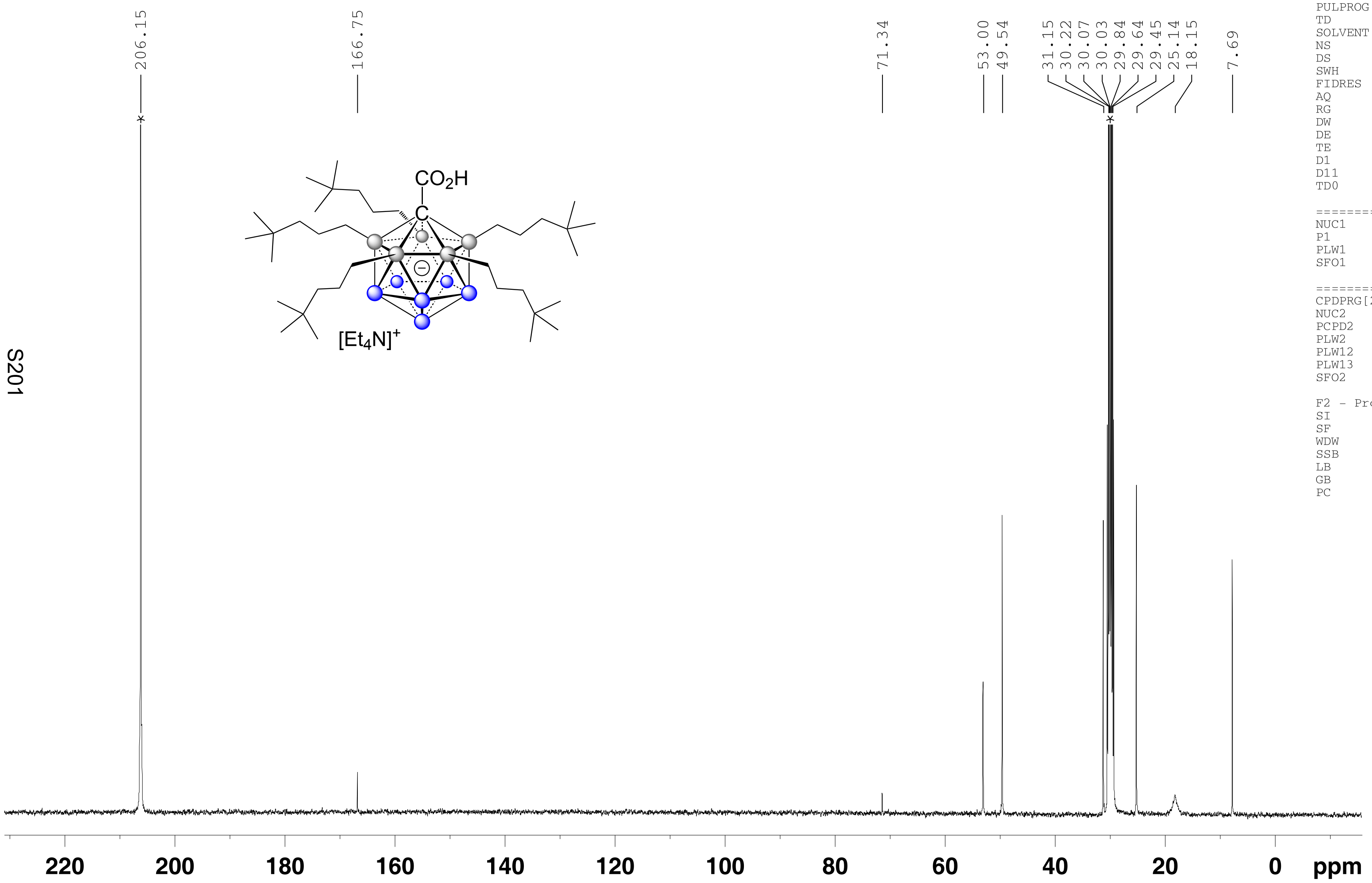


```
Current Data Parameters
NAME     penta-4,4-Dimethylpent-1-e
EXPNO                    5
PROCNO                   1

F2 - Acquisition Parameters
Date_             20180804
Time                 16.50
INSTRUM              spect
PROBHD   5 mm PABBO BB/
PULPROG             zgpg30
TD                   65536
SOLVENT            Acetone
NS                    3072
DS                       4
SWH              29761.904 Hz
FIDRES            0.454131 Hz
AQ               1.1010048 sec
RG                  193.34
DW                  16.800 usec
DE                    6.50 usec
TE                   295.6 K
D1              1.50000000 sec
D11             0.03000000 sec
TD0                      1

======== CHANNEL f1 ========
NUC1                   13C
P1                   10.00 usec
PLW1           53.00000000 W
SFO1          100.6228293 MHz

======== CHANNEL f2 ========
CPDPRG[2           waltz16
NUC2                    1H
PCPD2                80.00 usec
PLW2           12.50000000 W
PLW12           0.43945000 W
PLW13           0.28125000 W
SFO2          400.1316005 MHz

F2 - Processing parameters
SI                   32768
SF             100.6126817 MHz
WDW                     EM
SSB      0
LB                    3.00 Hz
GB       0
PC                    1.40
```

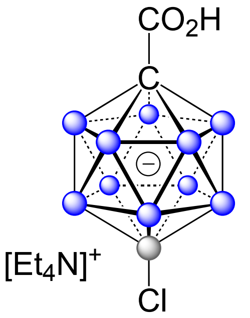

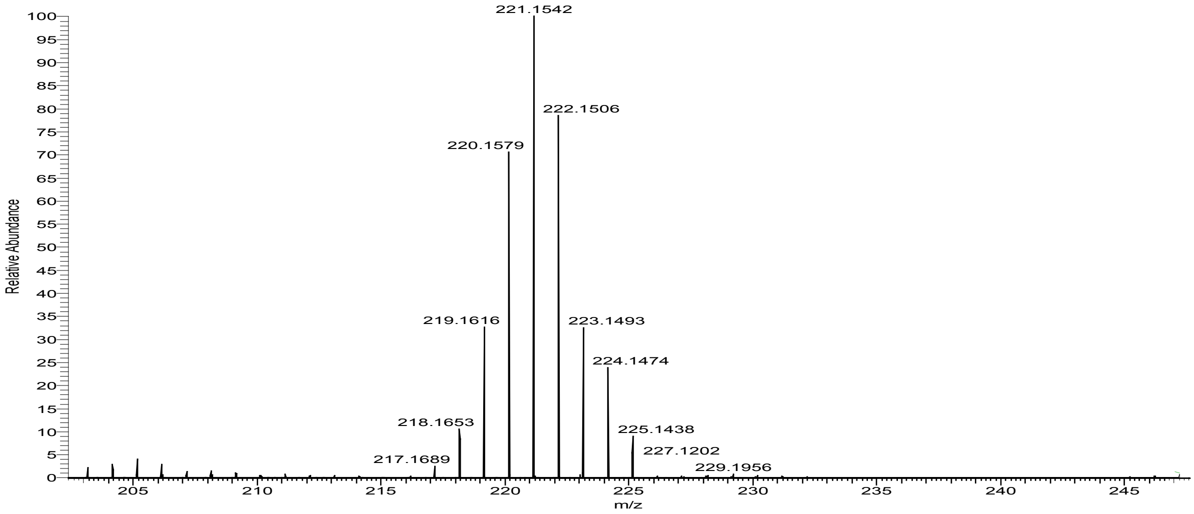

(–)-ESI-HRMS Shimadzu IT-TOF

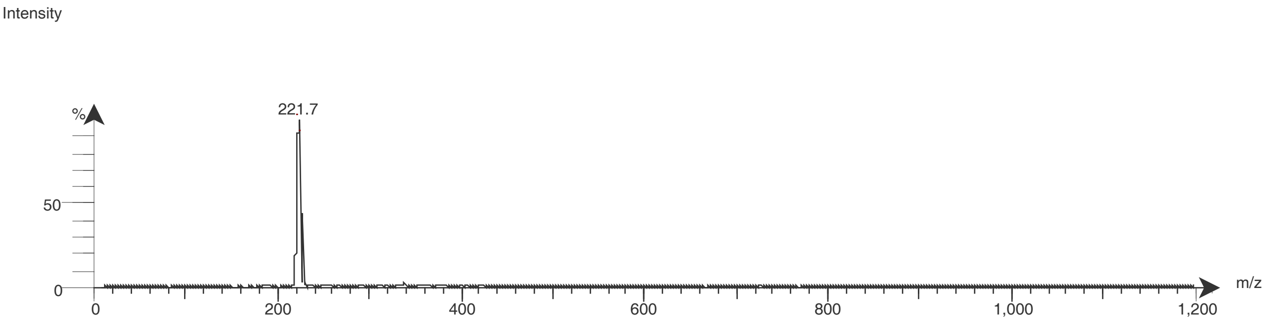

Full-range (–)-ESI-MS Expression CMS

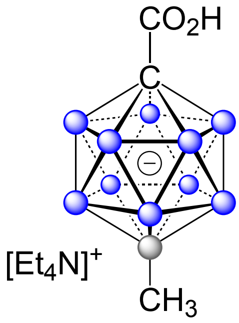

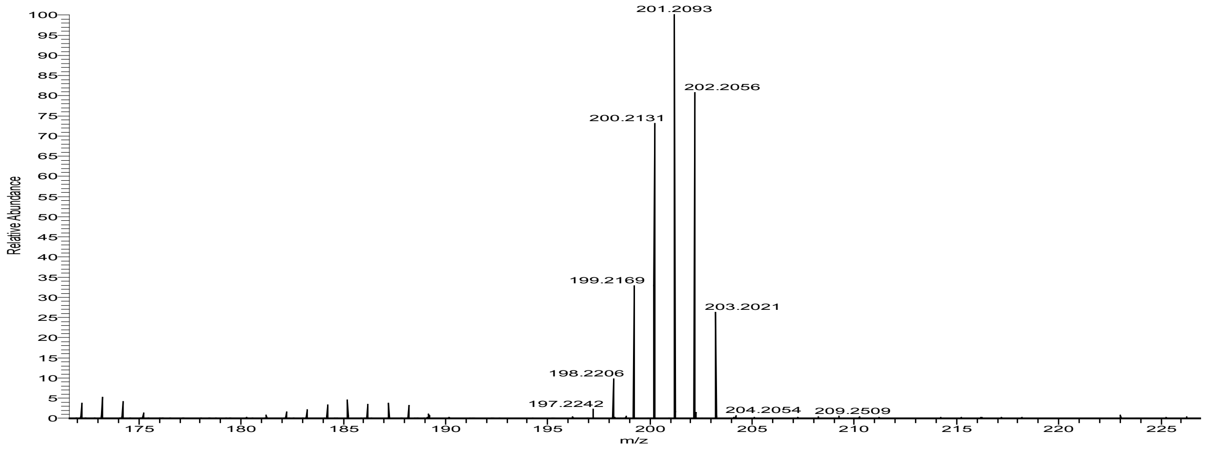

(–)-ESI-HRMS Shimadzu IT-TOF

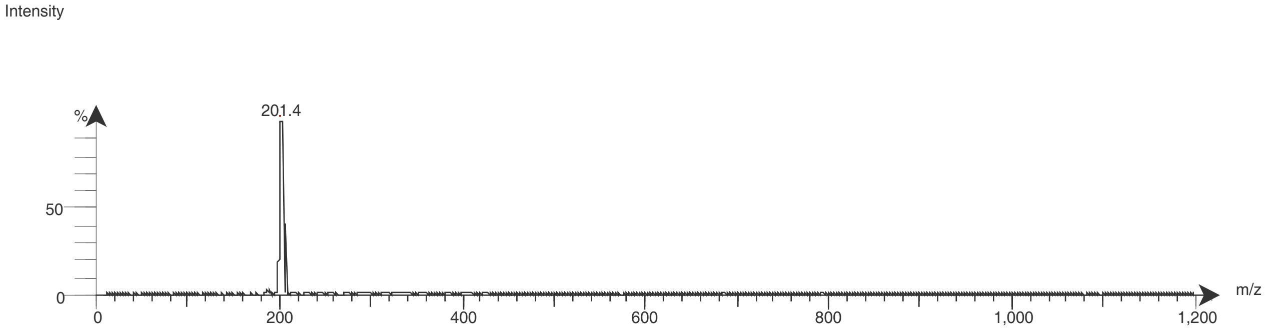

Full-range (–)-ESI-MS Expression CMS

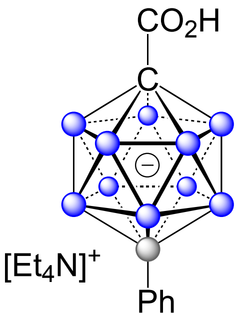

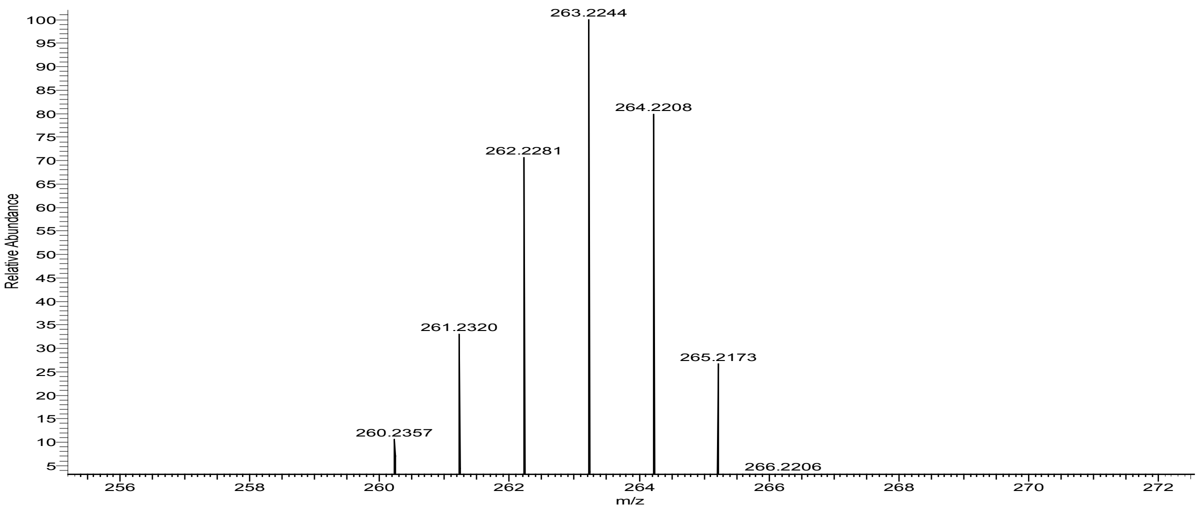

(–)-ESI-HRMS Shimadzu IT-TOF

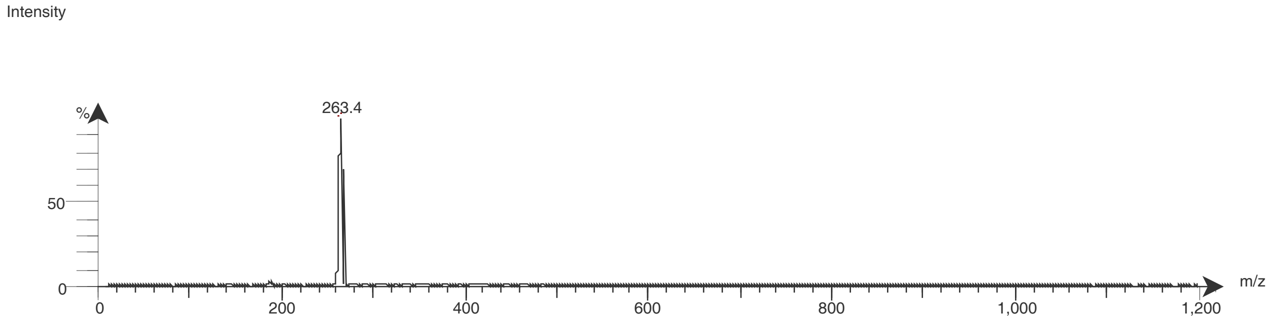

Full-range (–)-ESI-MS Expression CMS

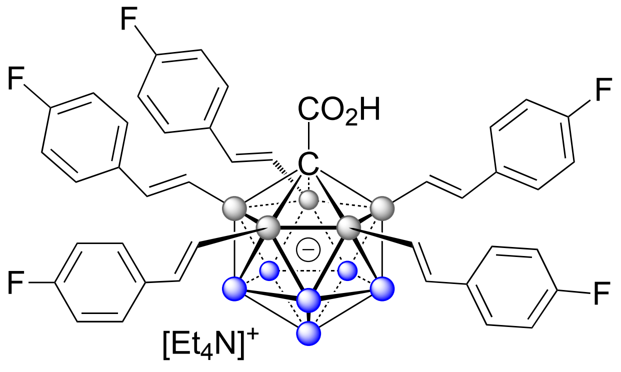

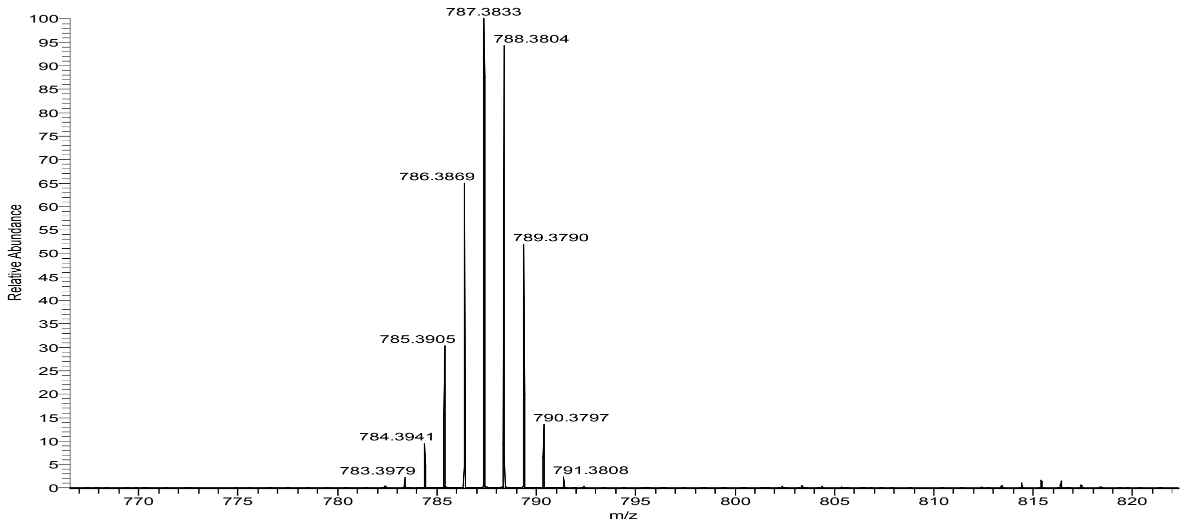

(–)-ESI-HRMS Shimadzu IT-TOF

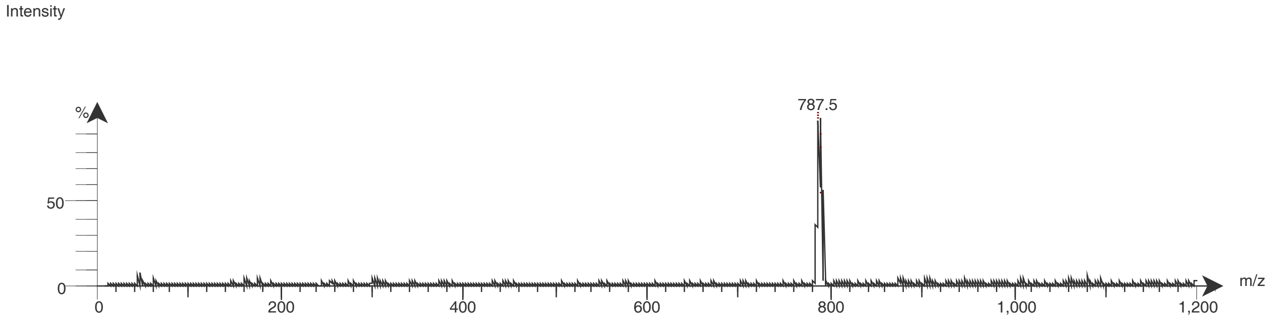

Full-range (–)-ESI-MS Expression CMS

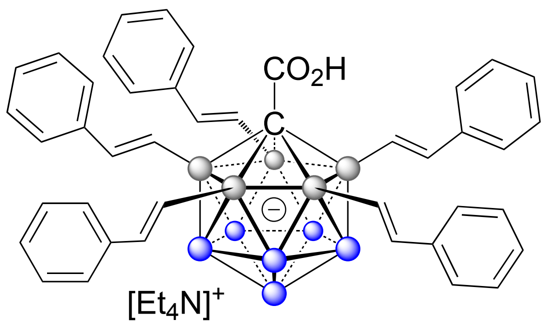

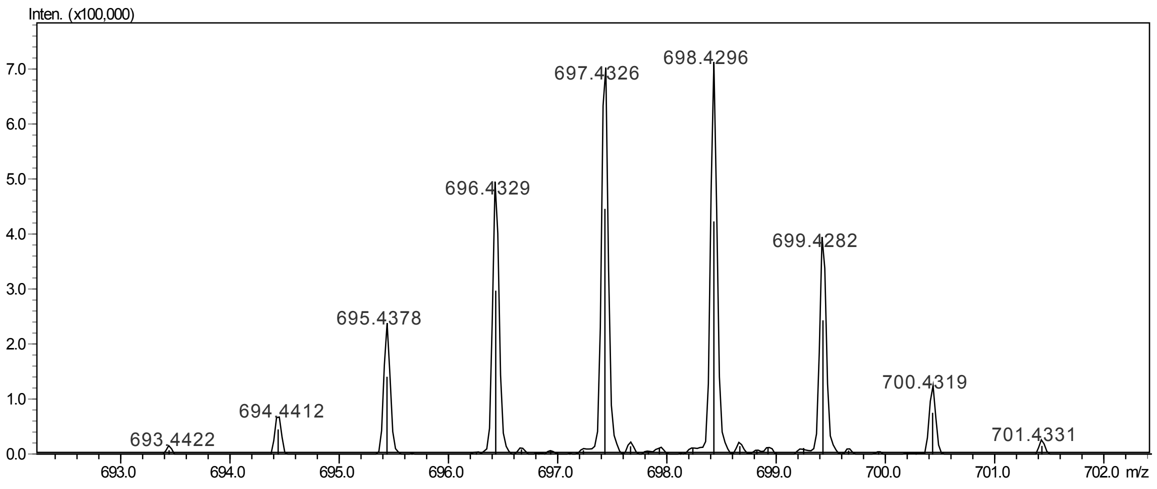

(–)-ESI-HRMS Shimadzu IT-TOF

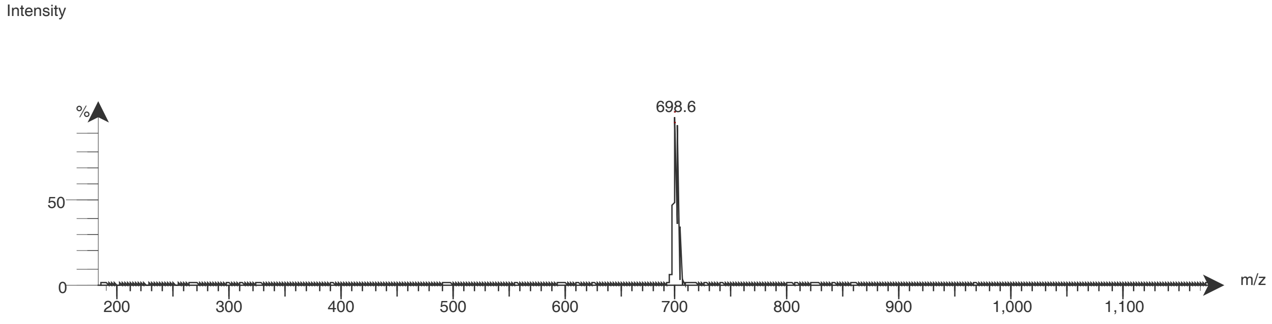

Full-range (–)-ESI-MS Expression CMS

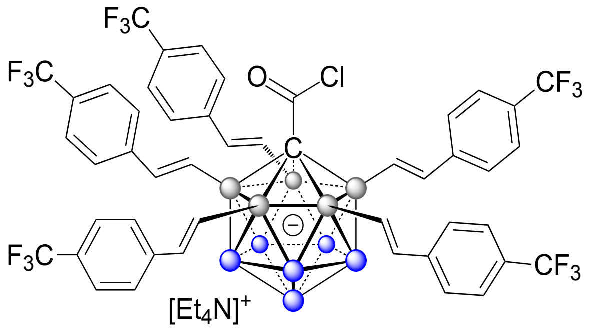

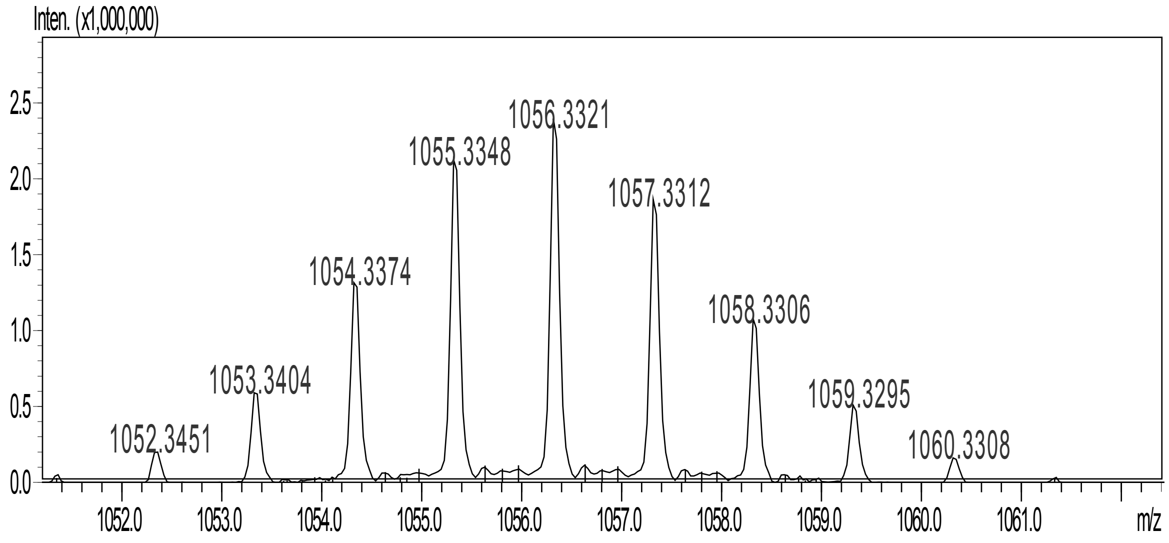

(–)-ESI-HRMS Shimadzu IT-TOF

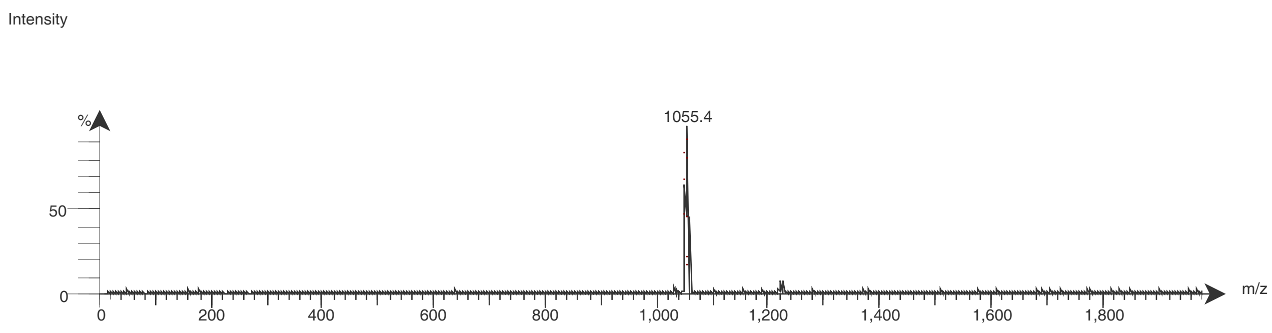

Full-range (–)-ESI-MS Expression CMS

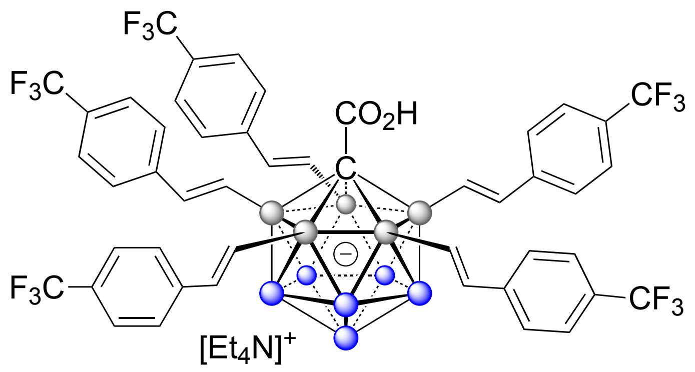

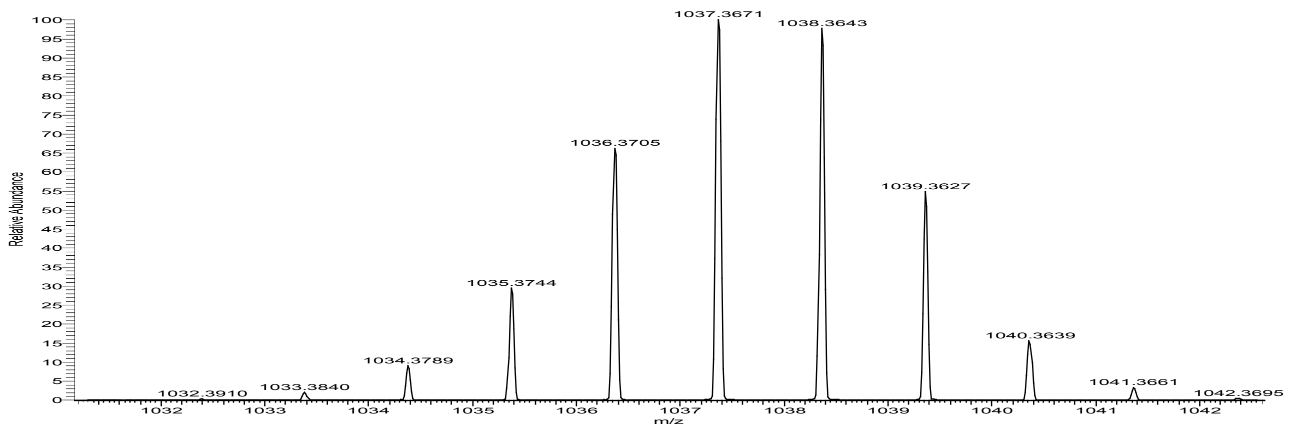

(–)-ESI-HRMS Shimadzu IT-TOF

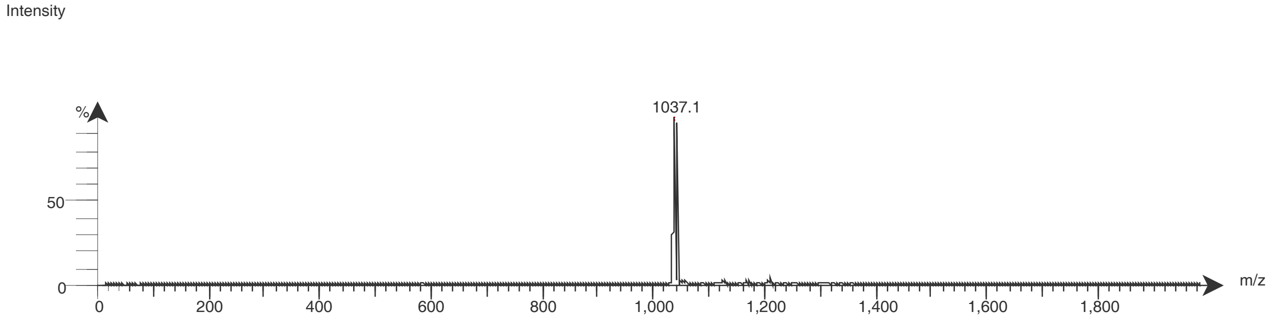

Full-range (–)-ESI-MS Expression CMS

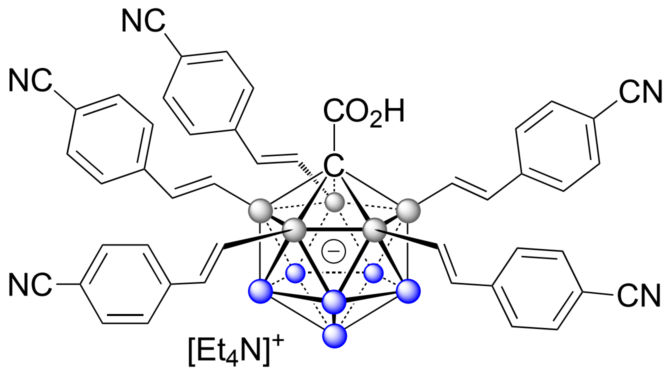

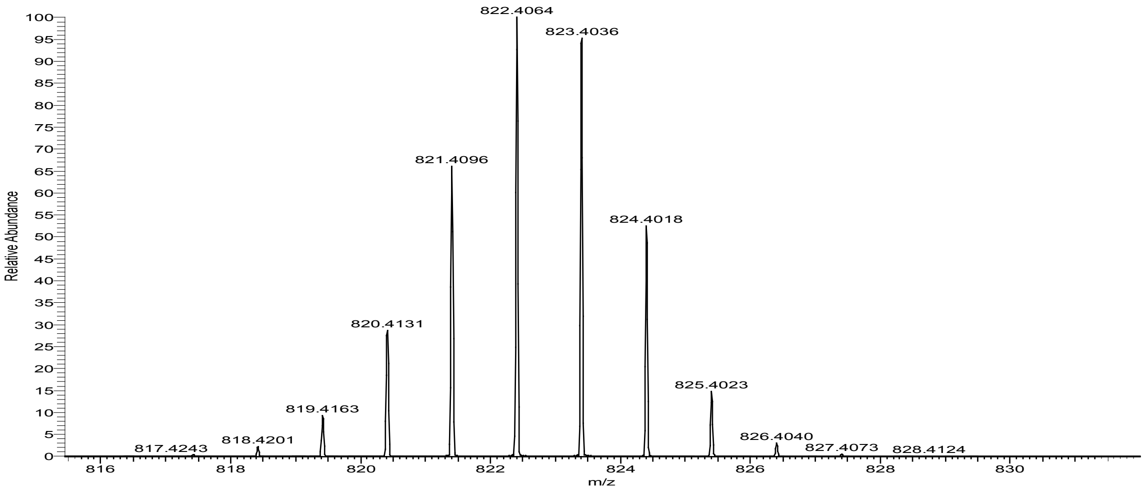

(–)-ESI-HRMS Shimadzu IT-TOF

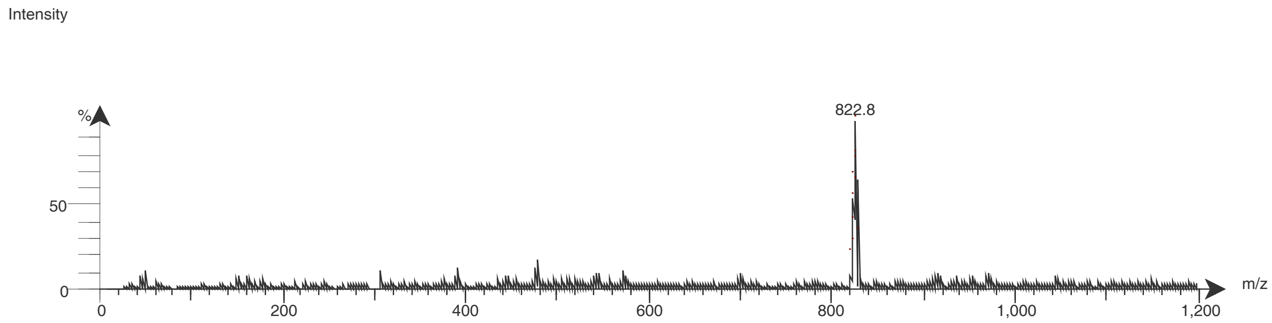

Full-range (–)-ESI-MS Expression CMS

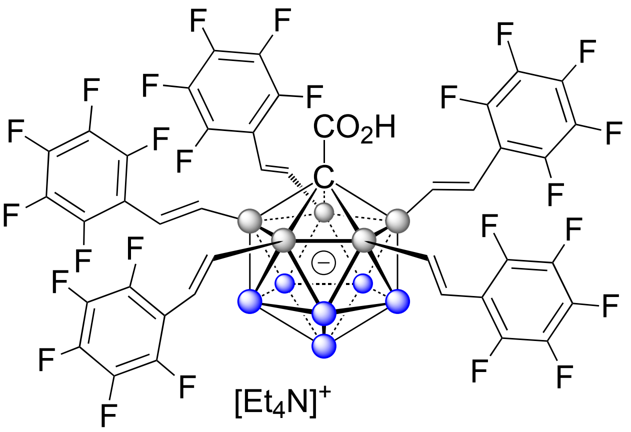

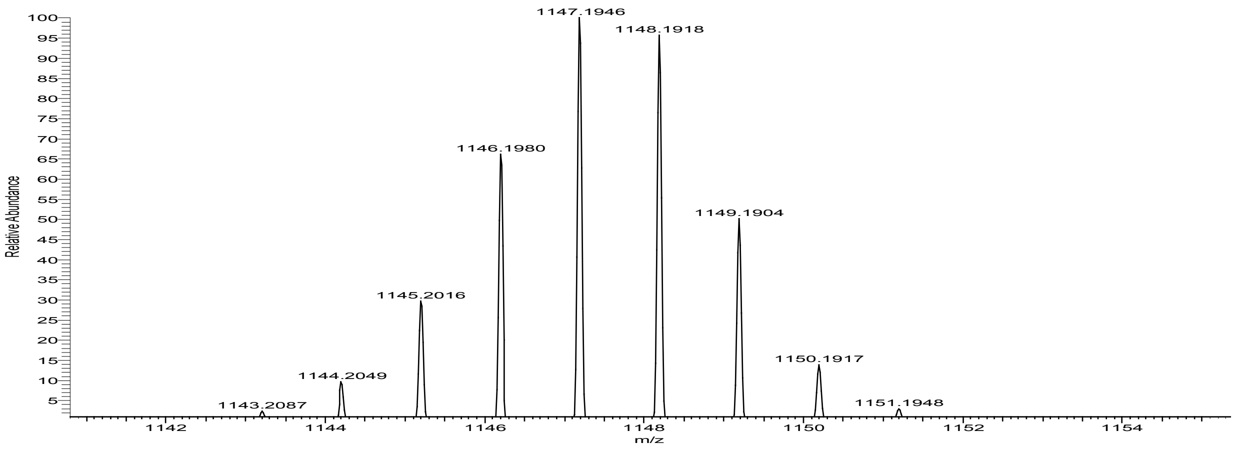

(–)-ESI-HRMS Shimadzu IT-TOF

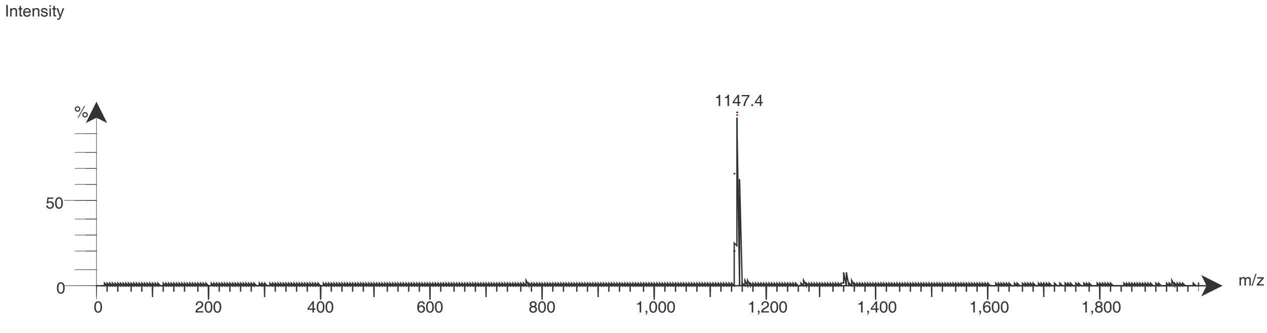

Full-range (–)-ESI-MS Expression CMS

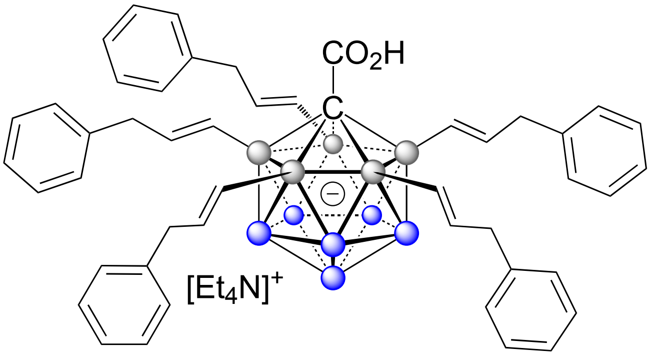

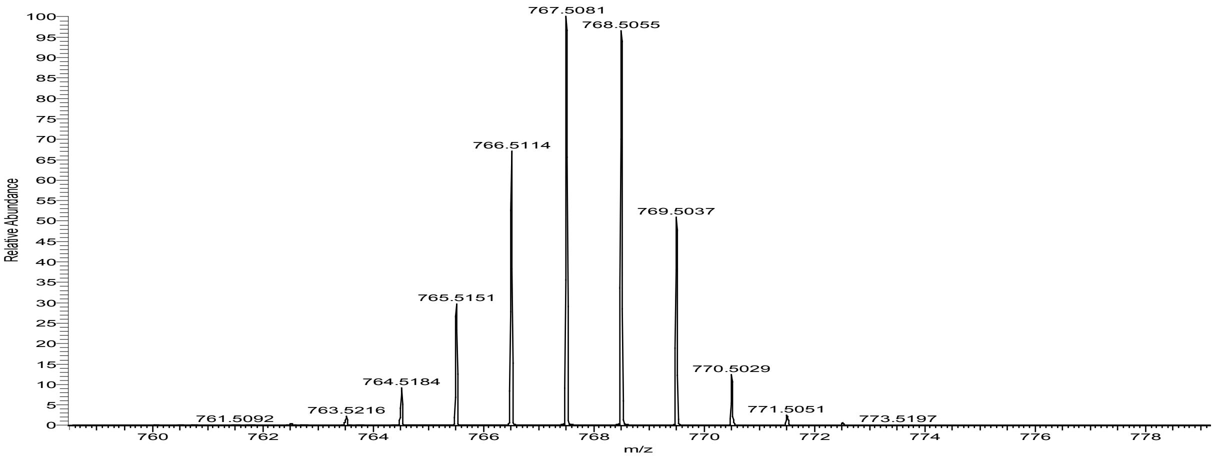

(–)-ESI-HRMS Shimadzu IT-TOF

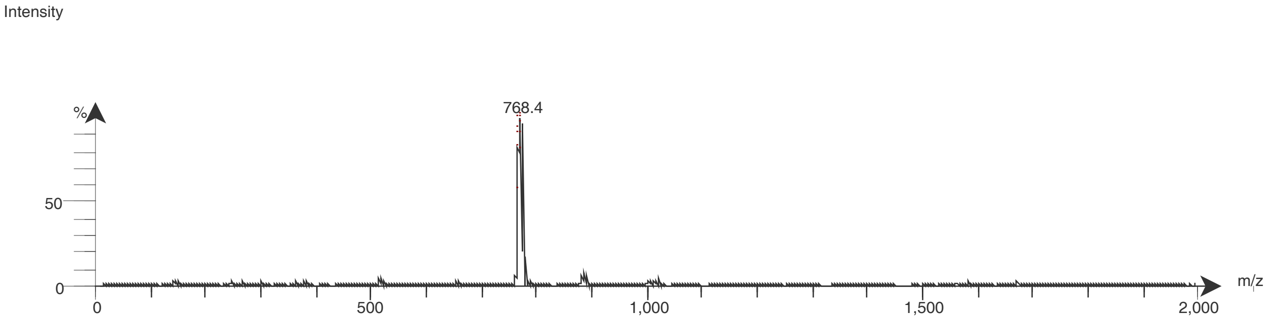

Full-range (–)-ESI-MS Expression CMS

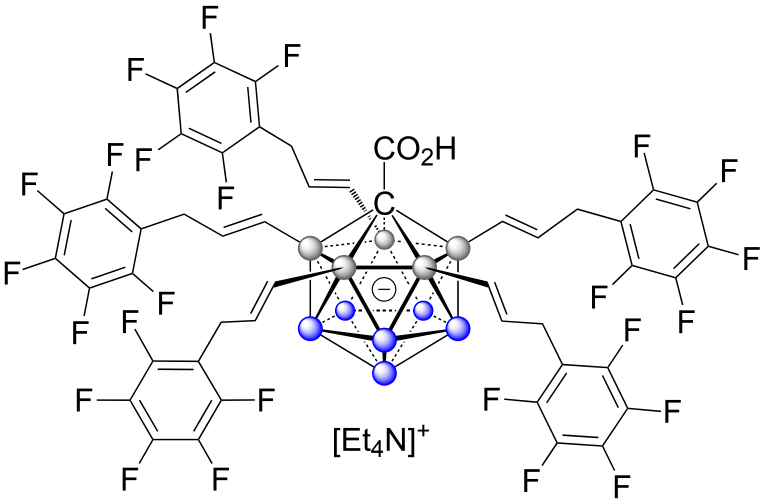

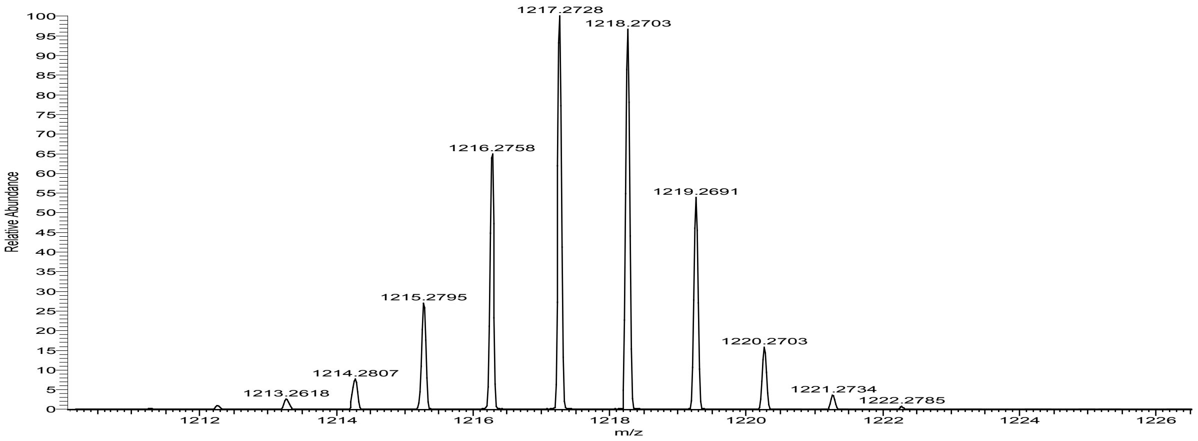

(–)-ESI-HRMS Shimadzu IT-TOF

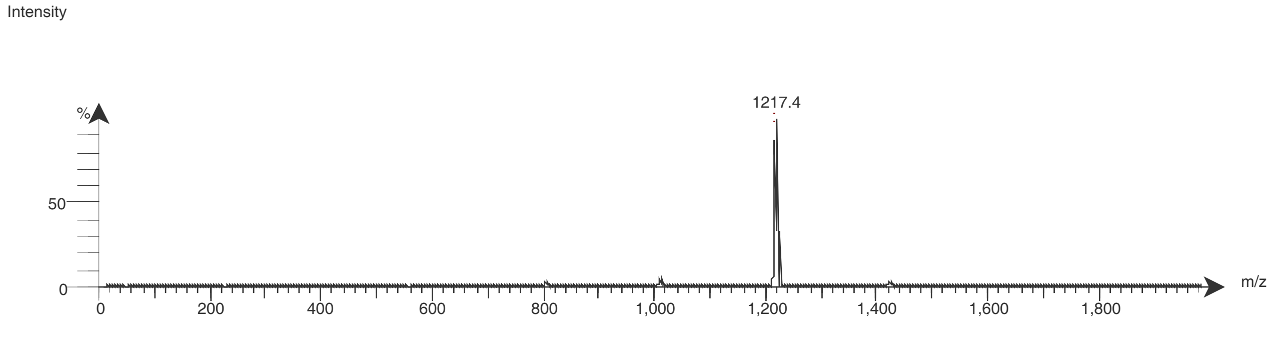

Full-range (–)-ESI-MS Expression CMS

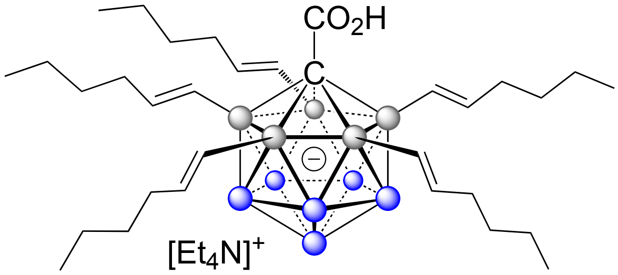

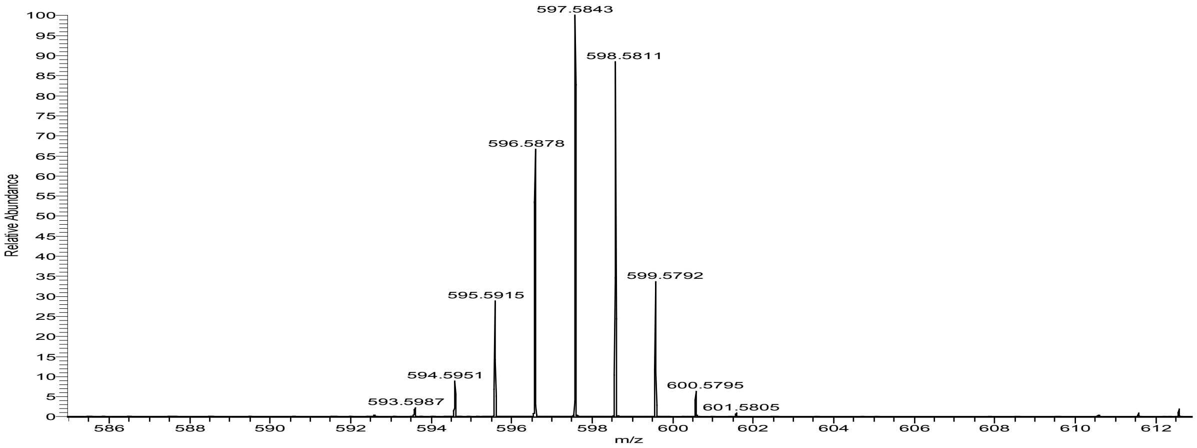

(–)-ESI-HRMS Shimadzu IT-TOF

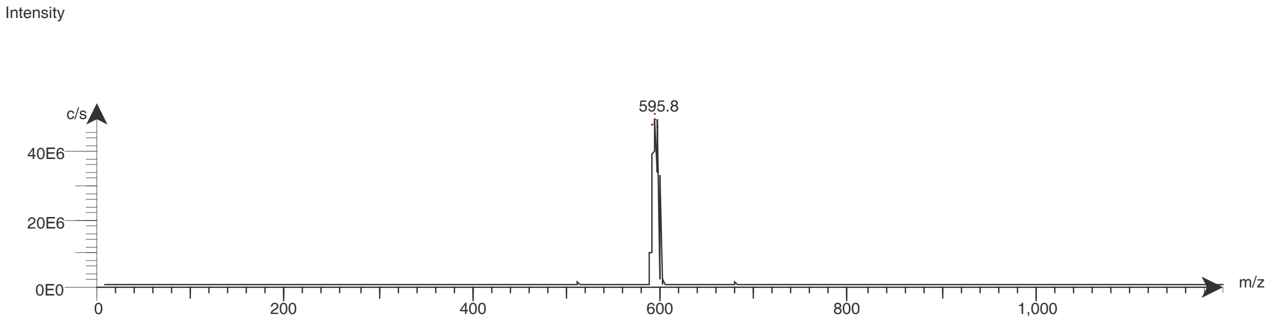

Full-range (–)-ESI-MS Expression CMS

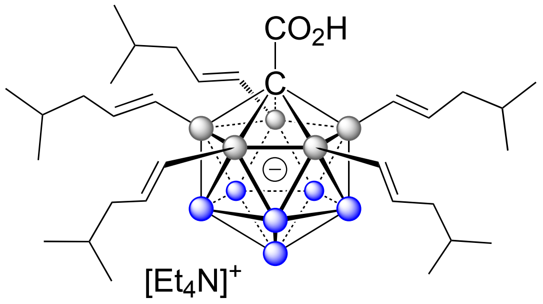

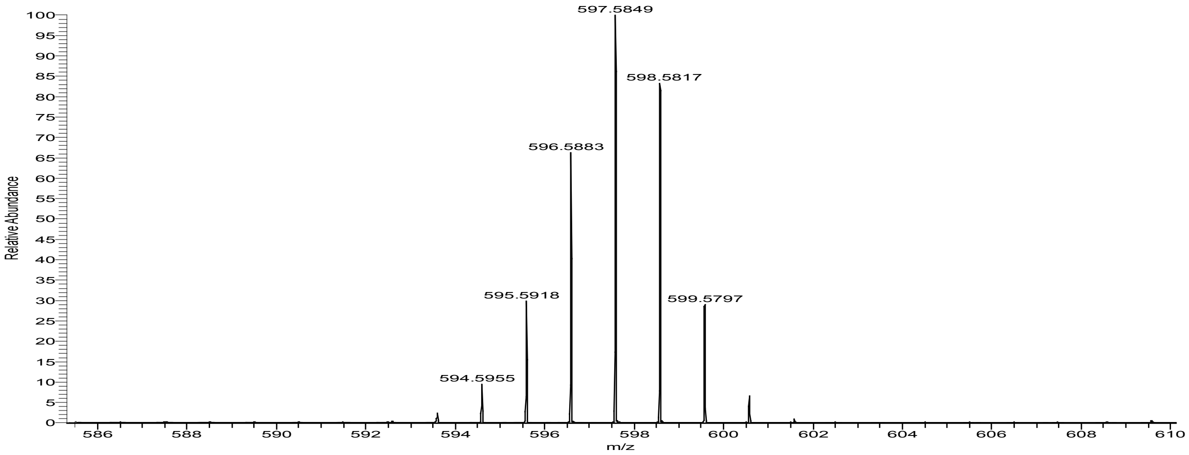

(–)-ESI-HRMS Shimadzu IT-TOF

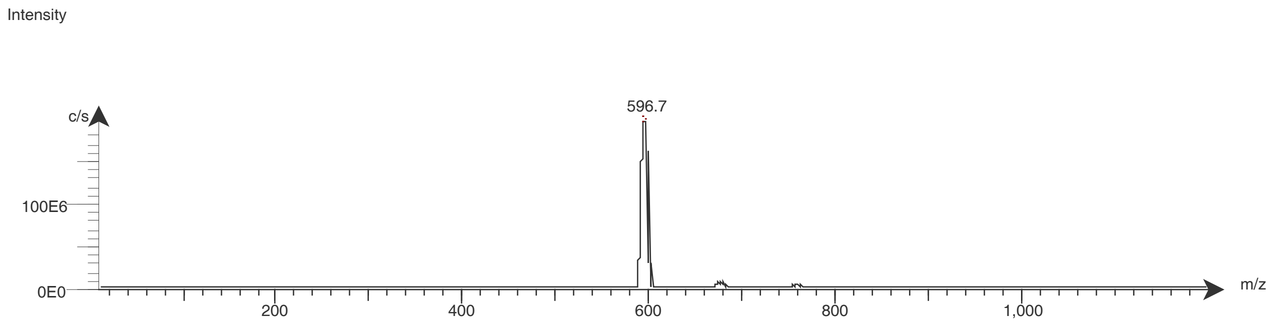

Full-range (–)-ESI-MS Expression CMS

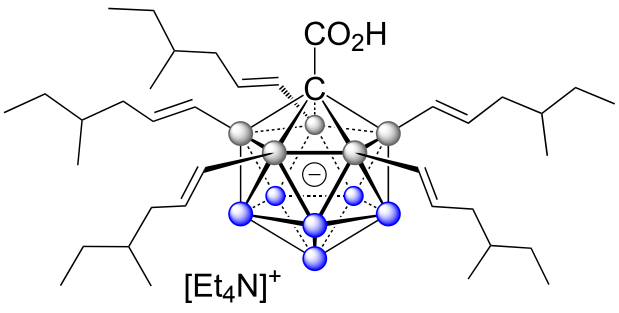

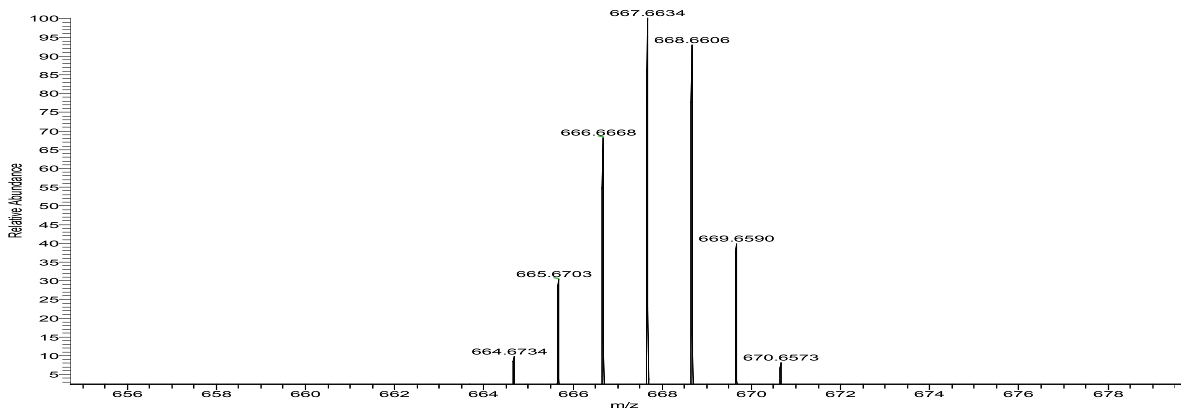

(–)-ESI-HRMS Shimadzu IT-TOF

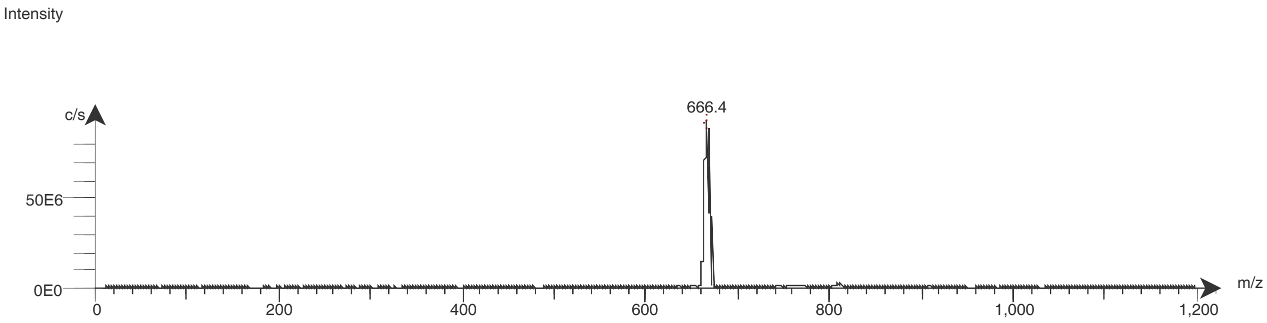

Full-range (–)-ESI-MS Expression CMS

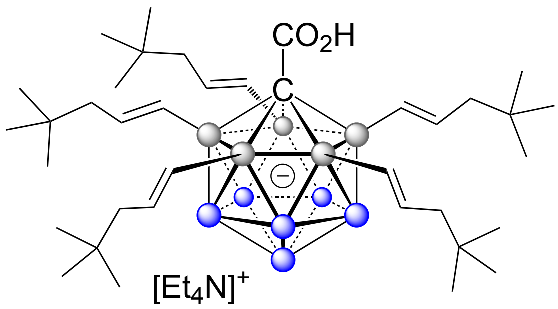

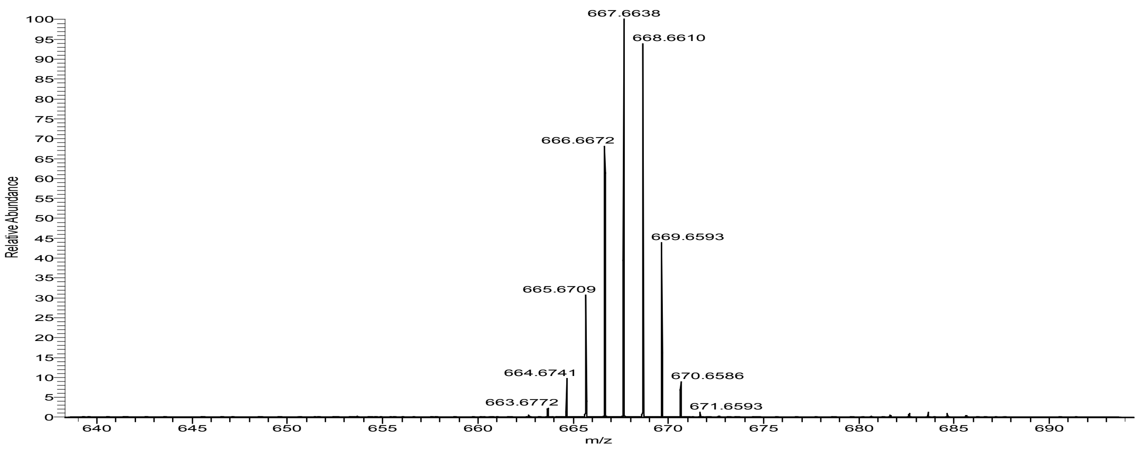

(–)-ESI-HRMS Shimadzu IT-TOF

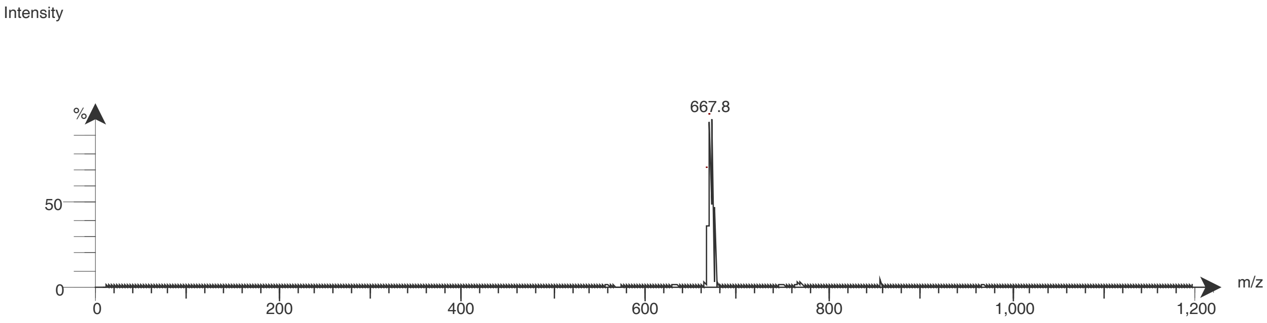

Full-range (–)-ESI-MS Expression CMS

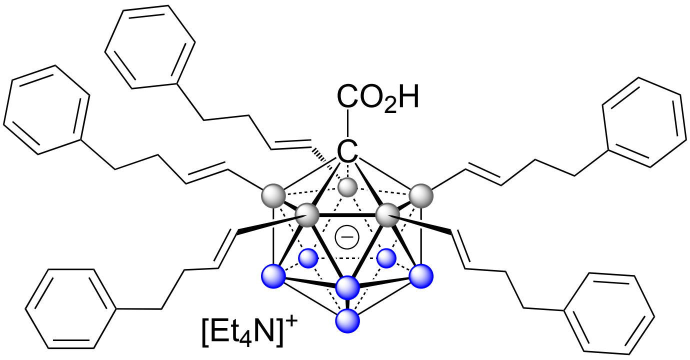

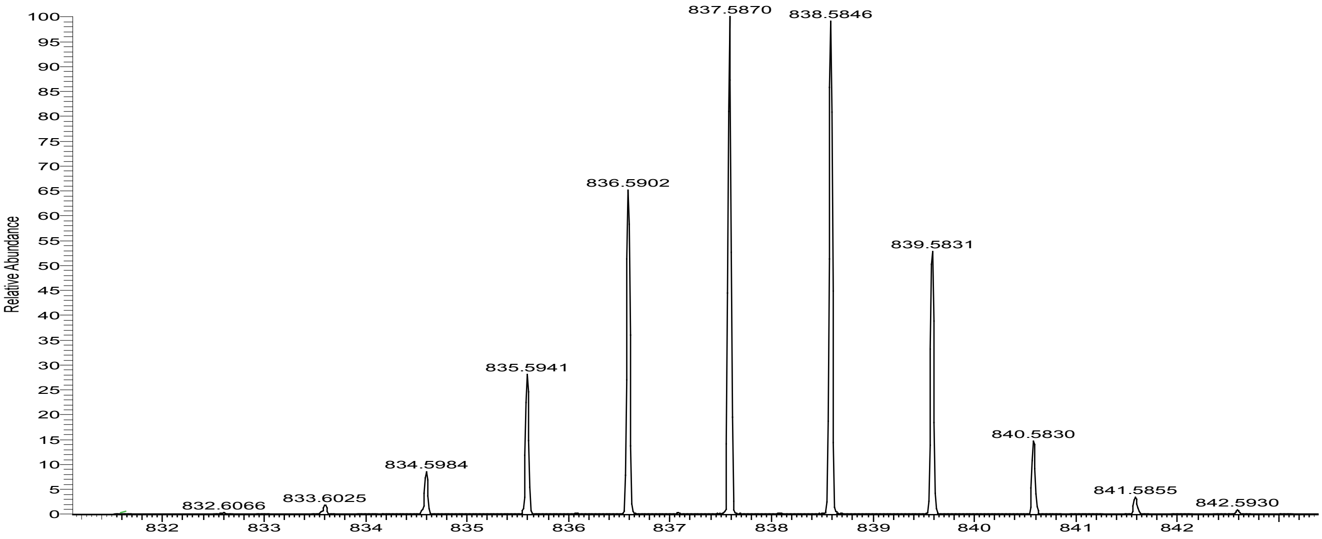

(–)-ESI-HRMS Shimadzu IT-TOF

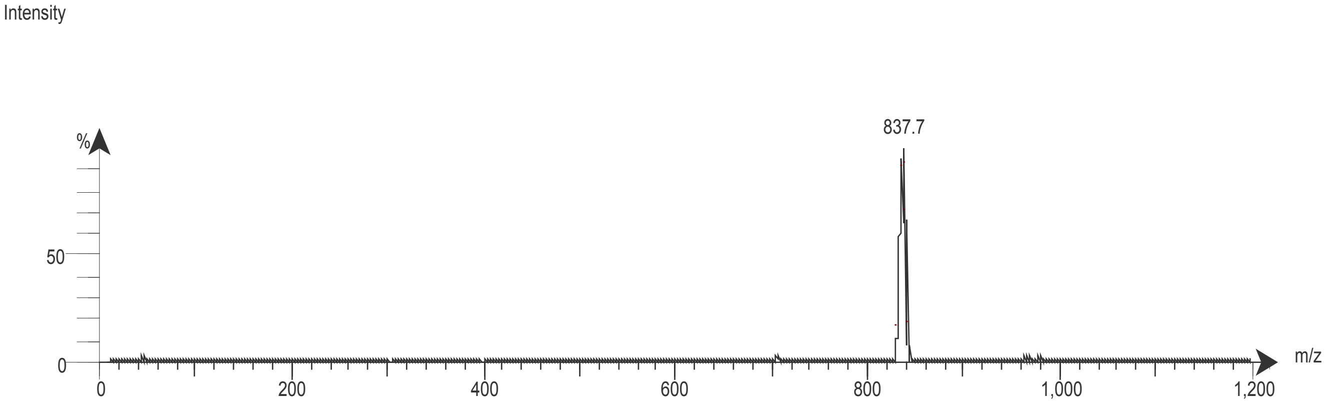

Full-range (–)-ESI-MS Expression CMS

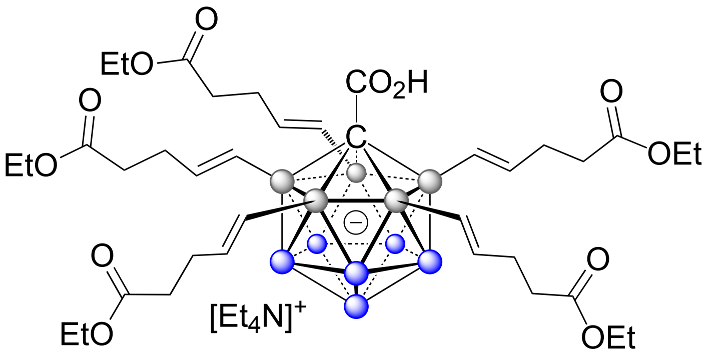

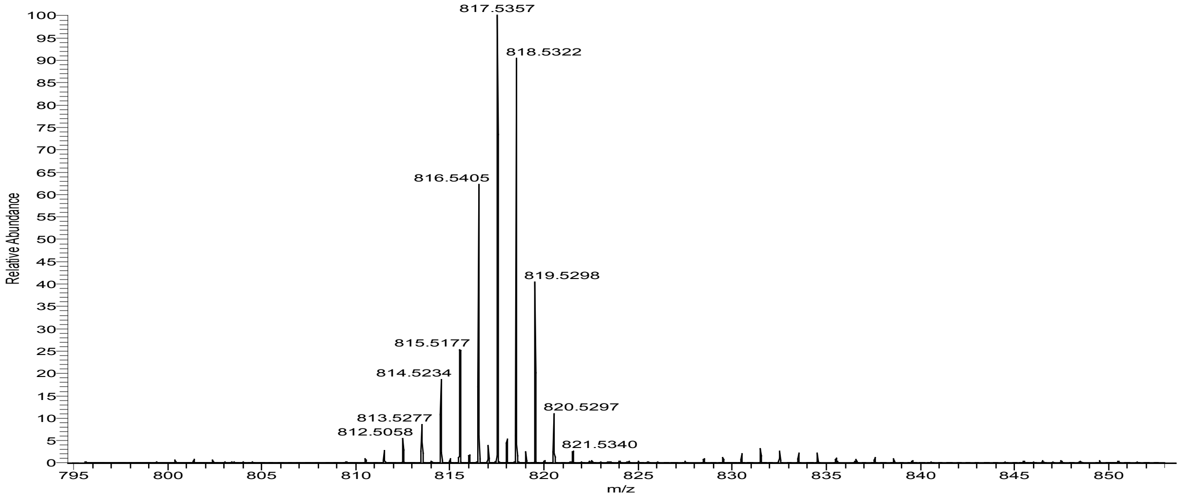

(–)-ESI-HRMS Shimadzu IT-TOF

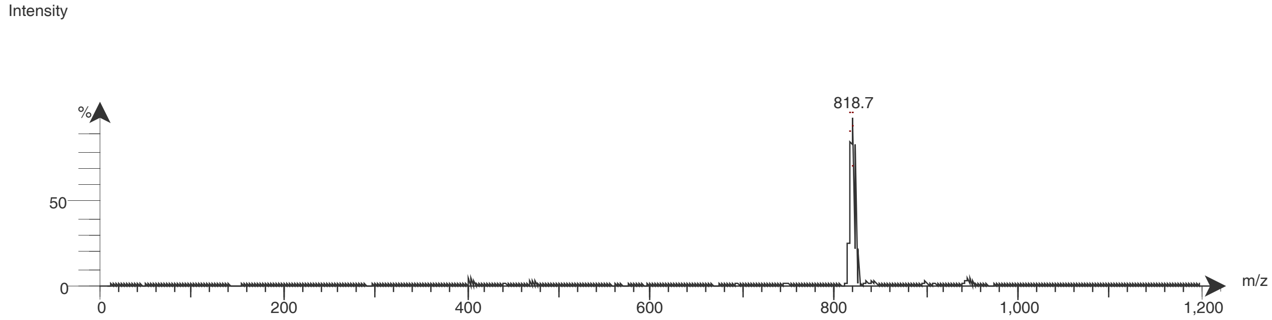

Full-range (–)-ESI-MS Expression CMS

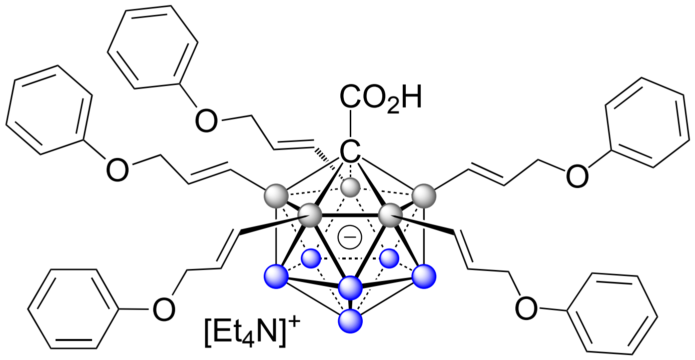

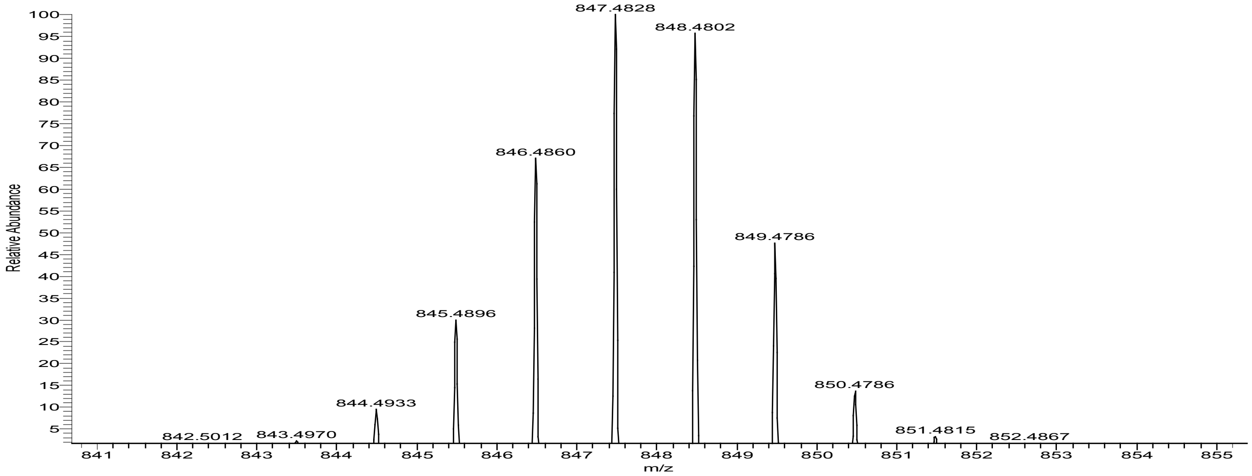

(–)-ESI-HRMS Shimadzu IT-TOF

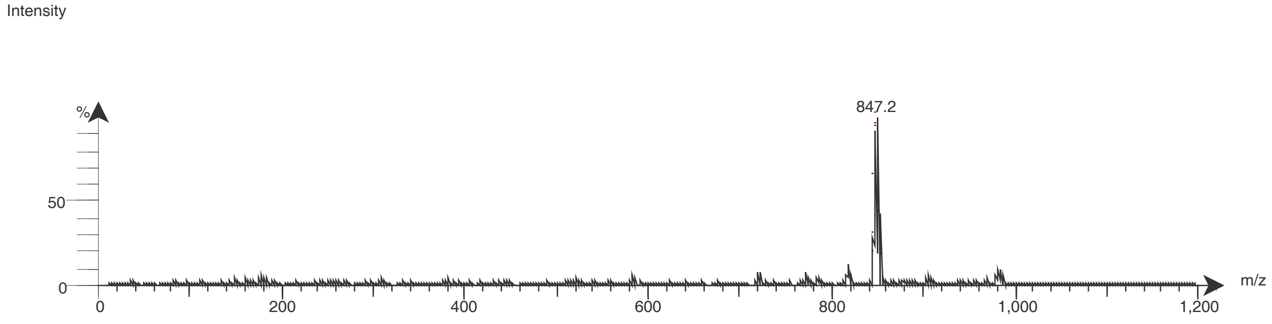

Full-range (–)-ESI-MS Expression CMS

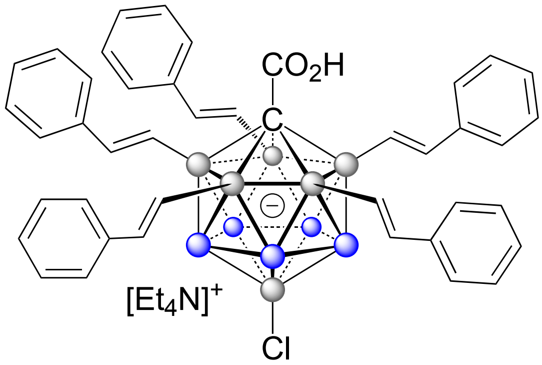

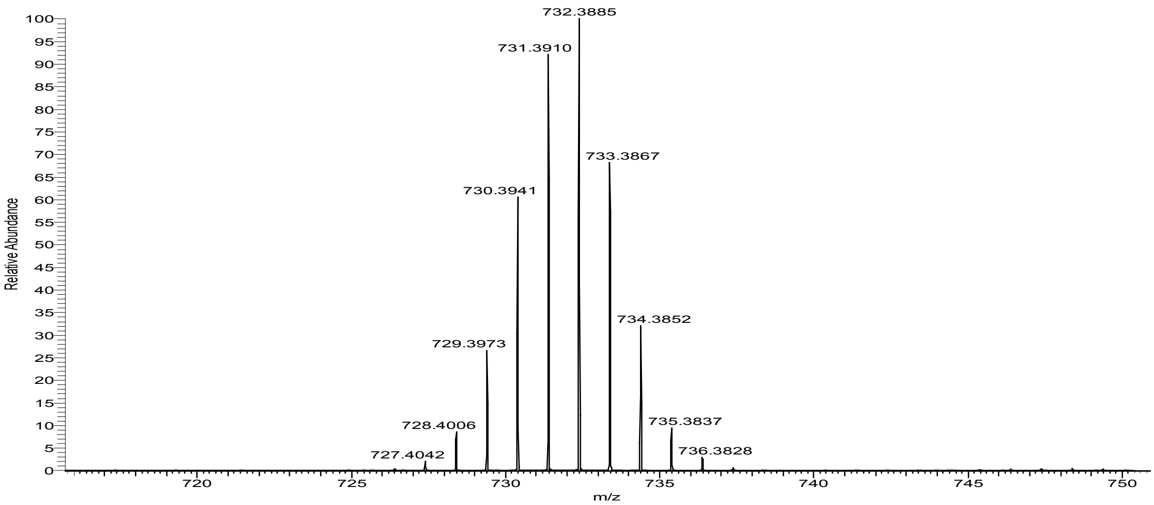

(–)-ESI-HRMS Shimadzu IT-TOF

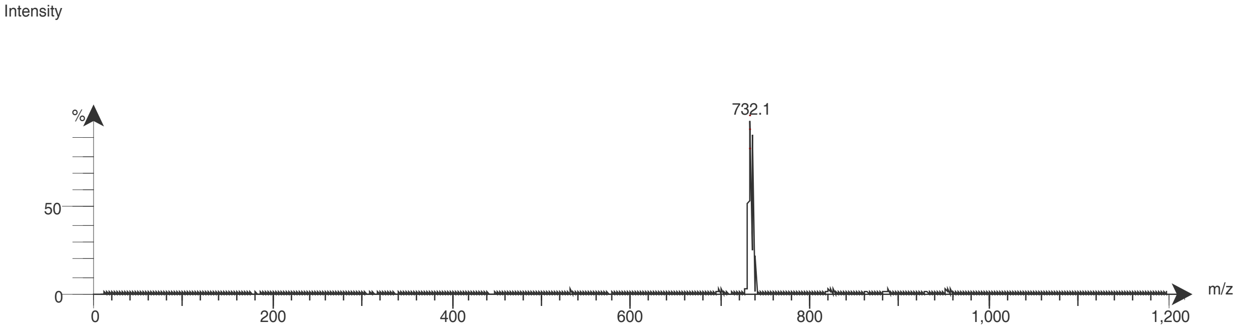

Full-range (–)-ESI-MS Expression CMS

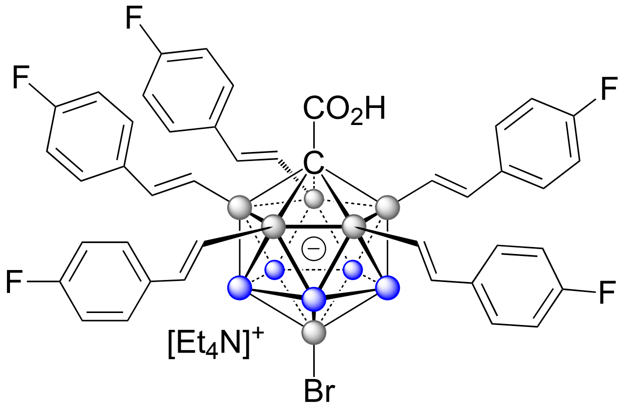

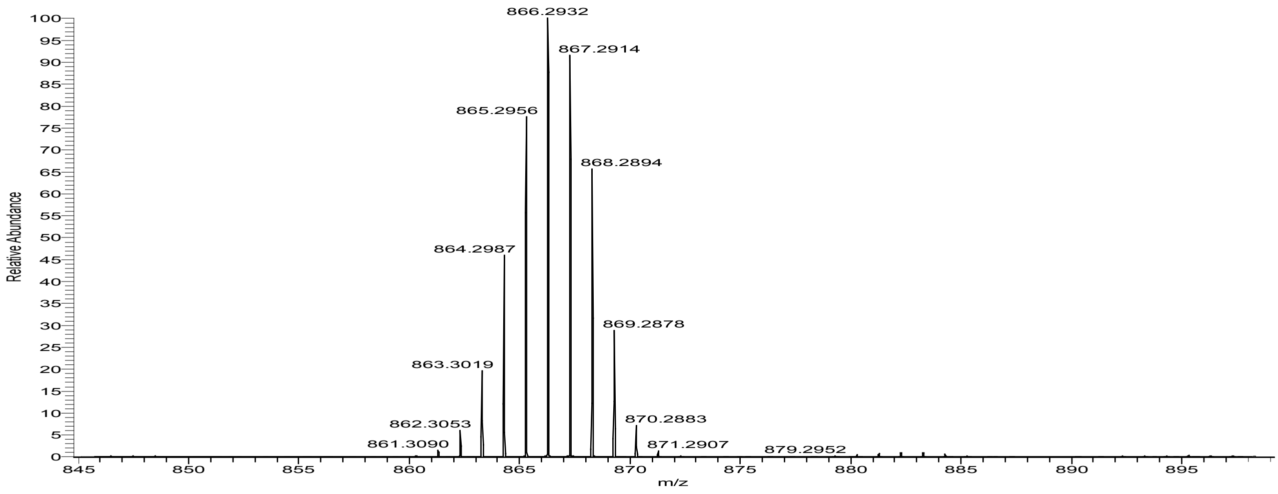

(–)-ESI-HRMS Shimadzu IT-TOF

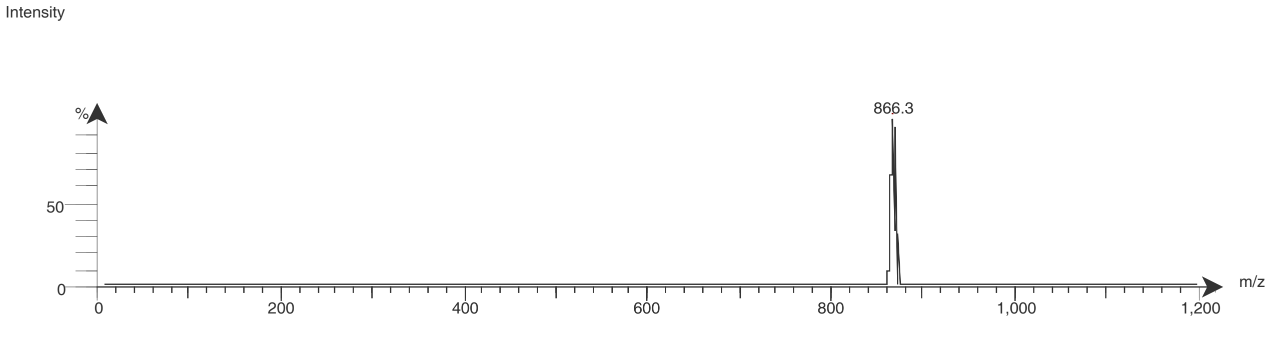

Full-range (–)-ESI-MS Expression CMS

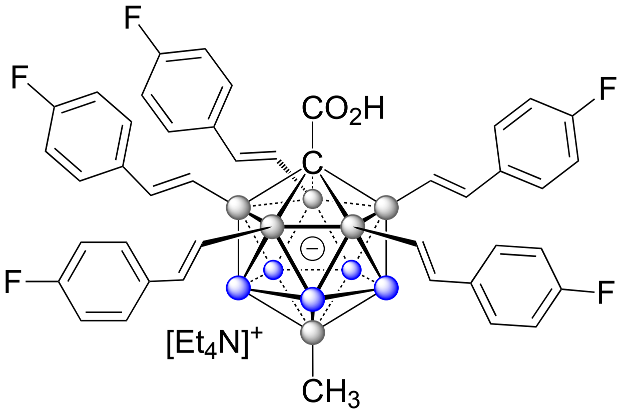

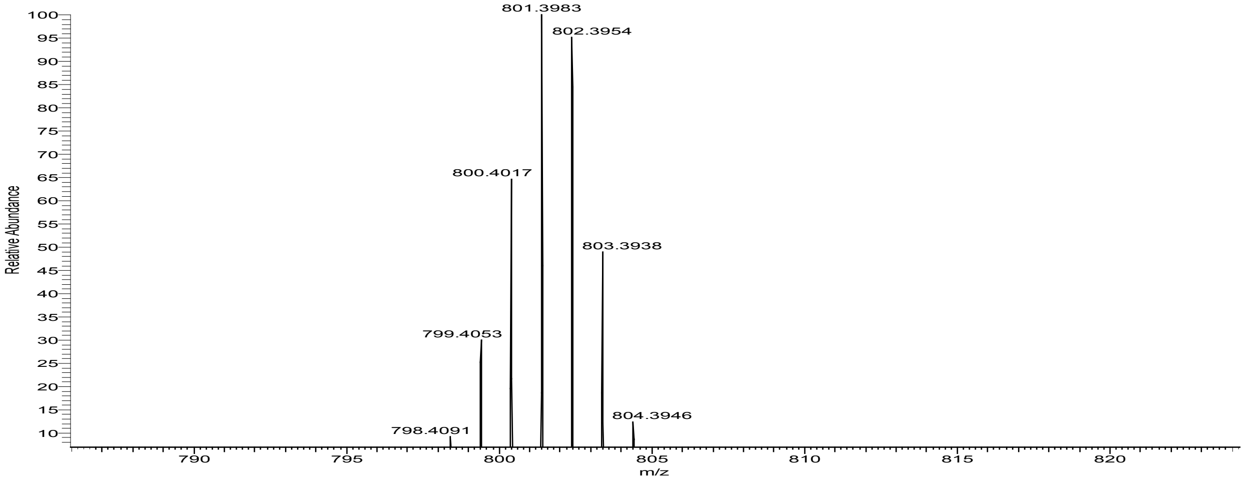

(–)-ESI-HRMS Shimadzu IT-TOF

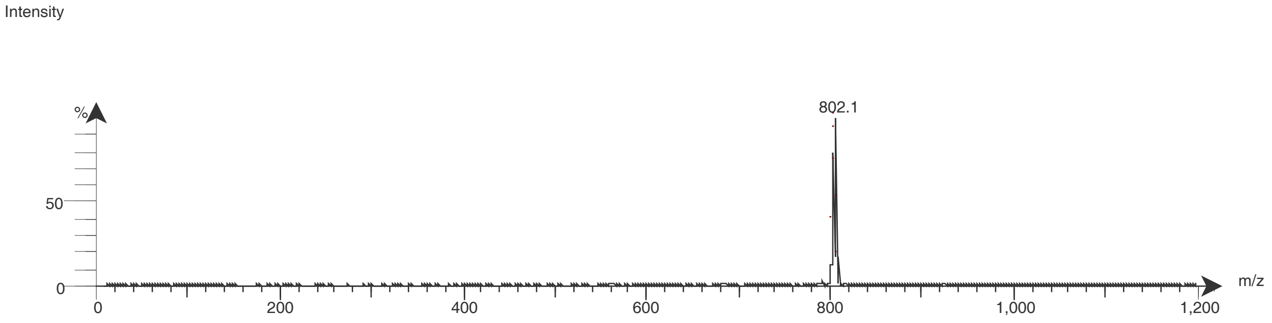

Full-range (–)-ESI-MS Expression CMS

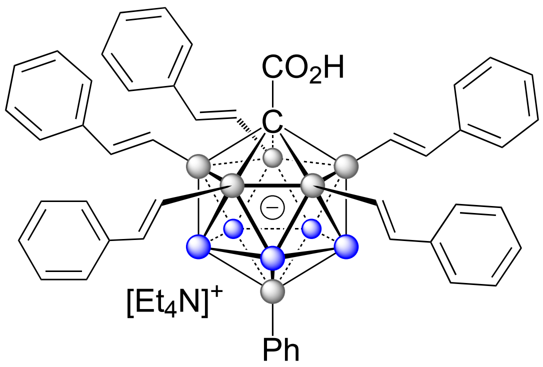

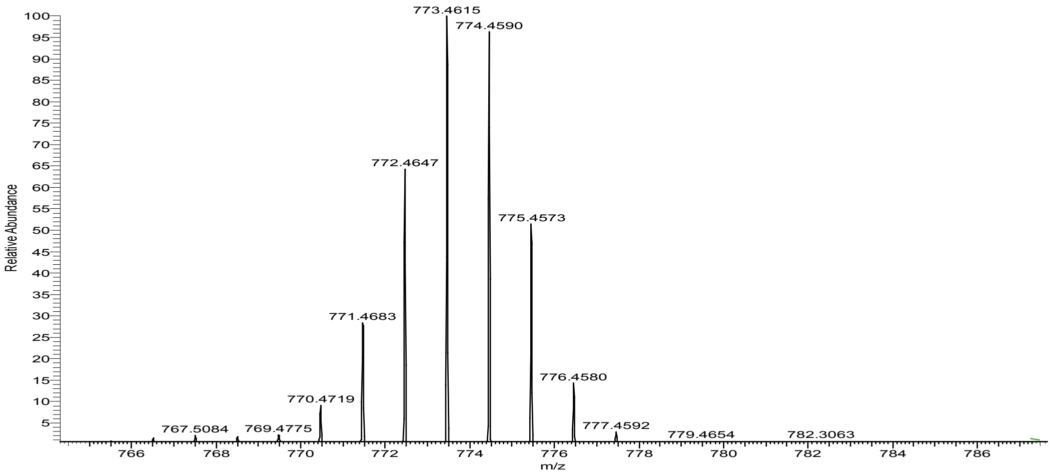

(–)-ESI-HRMS Shimadzu IT-TOF

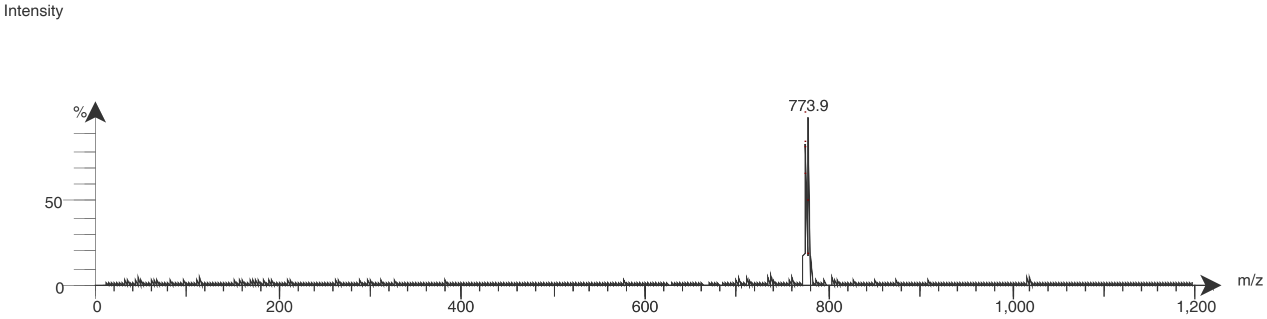

Full-range (–)-ESI-MS Expression CMS

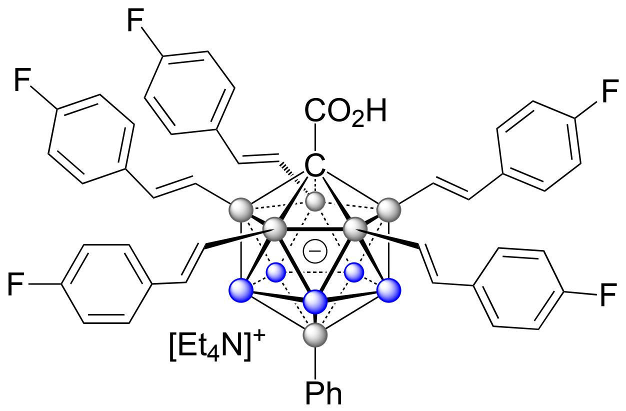

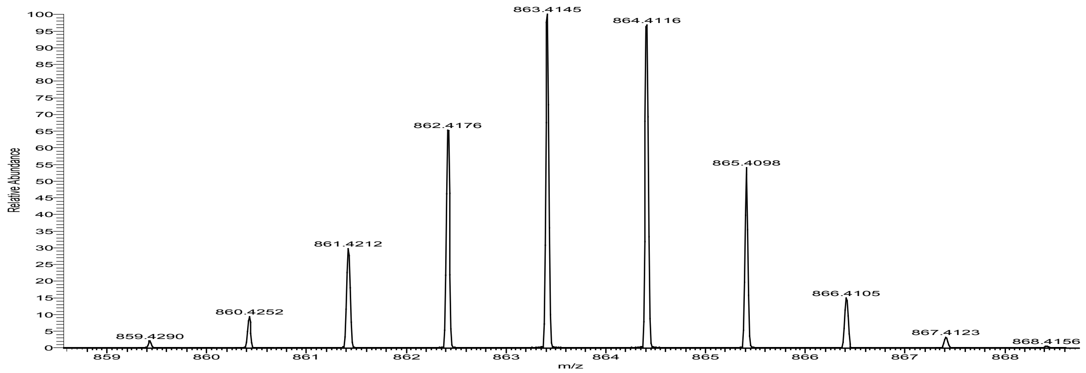

(–)-ESI-HRMS Shimadzu IT-TOF

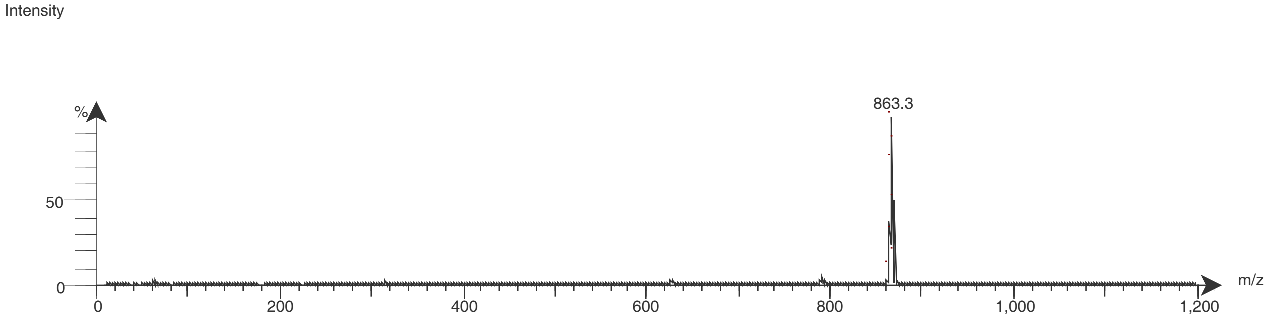

Full-range (–)-ESI-MS Expression CMS

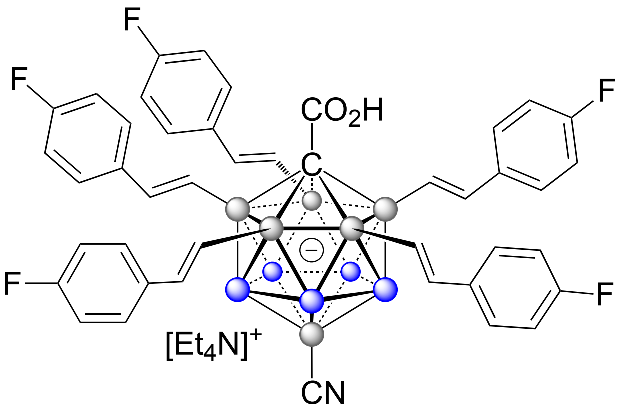

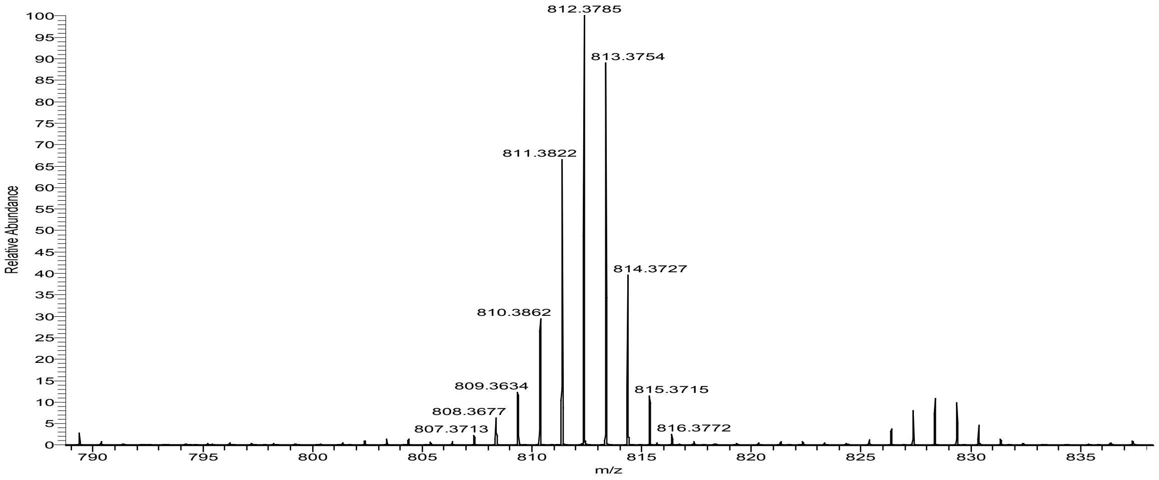

(–)-ESI-HRMS Shimadzu IT-TOF

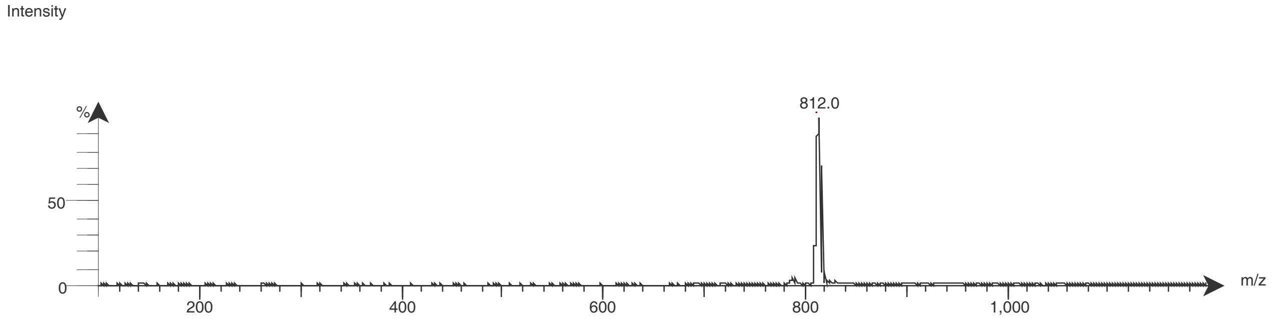

Full-range (–)-ESI-MS Expression CMS

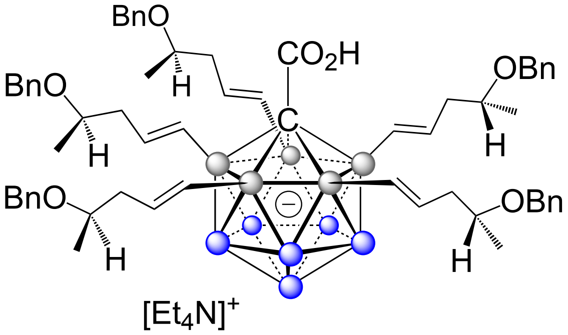

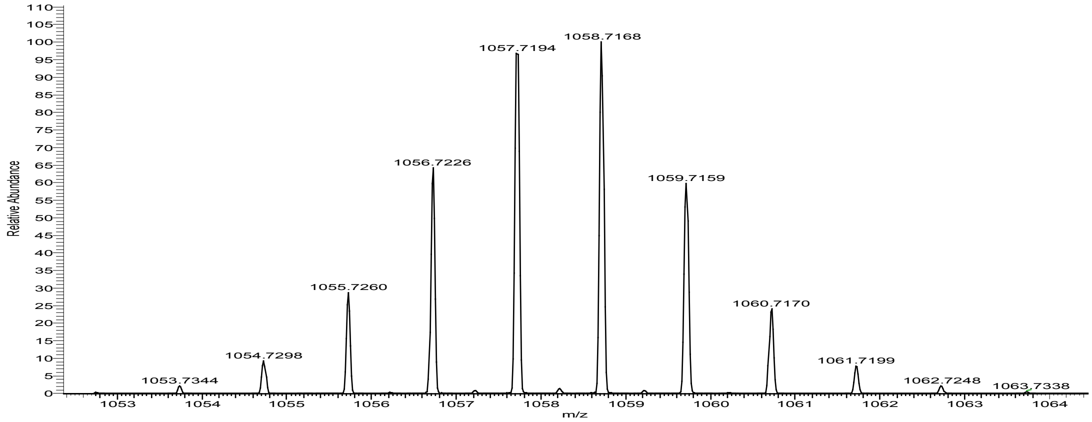

(–)-ESI-HRMS Shimadzu IT-TOF

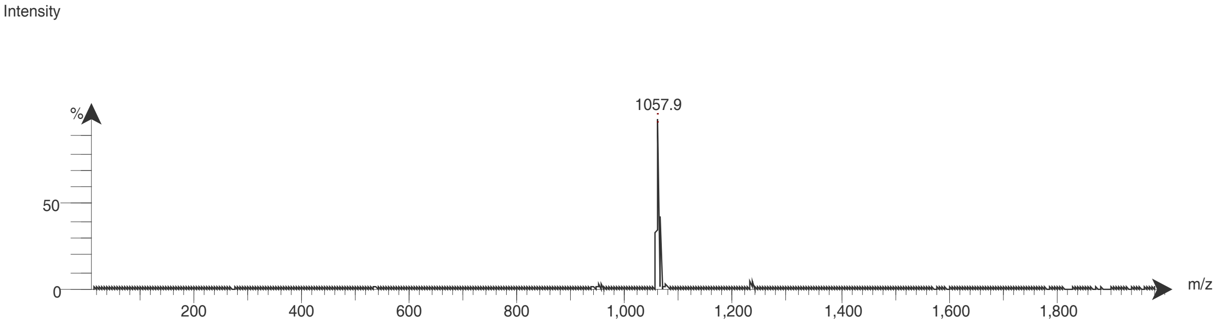

Full-range (–)-ESI-MS Expression CMS

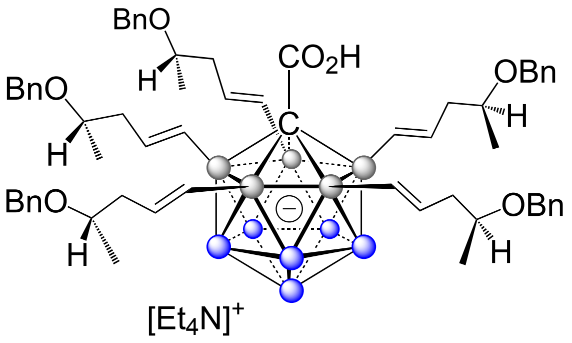

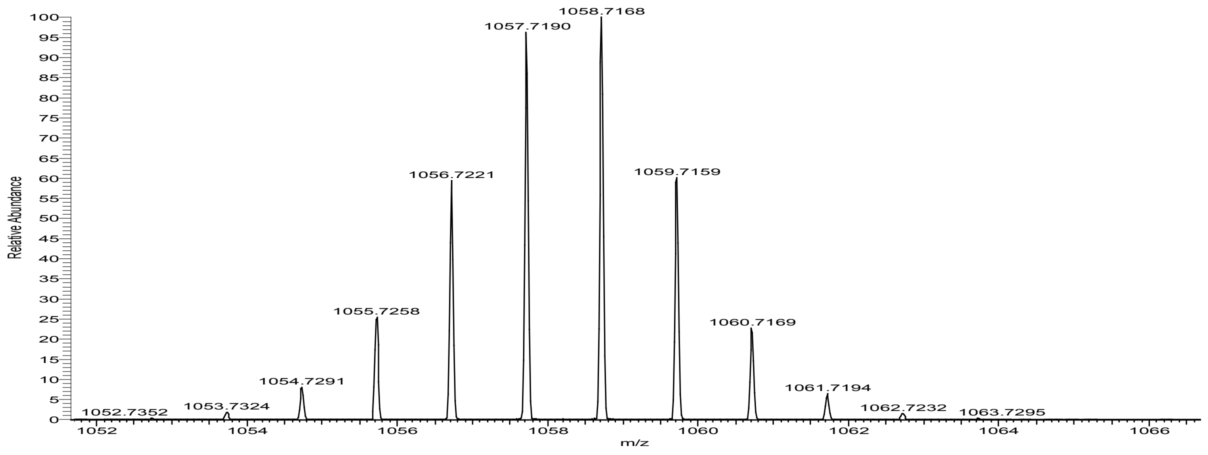

(–)-ESI-HRMS Shimadzu IT-TOF

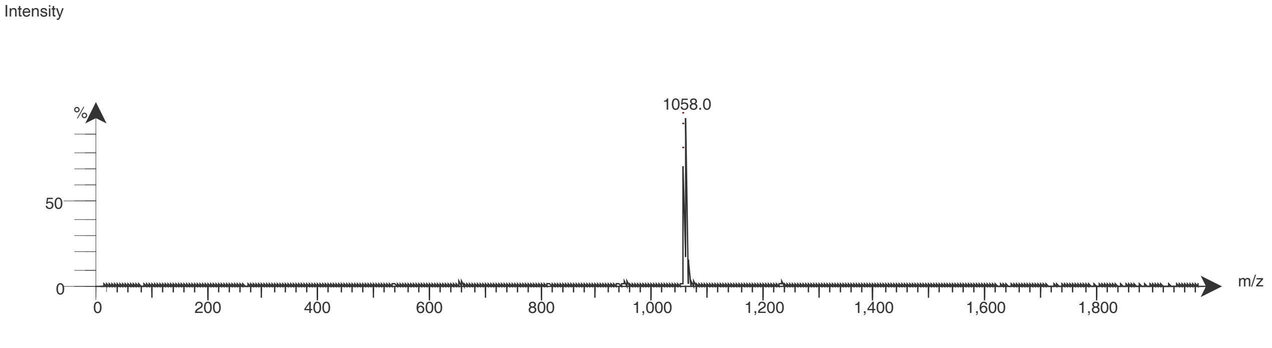

Full-range (–)-ESI-MS Expression CMS

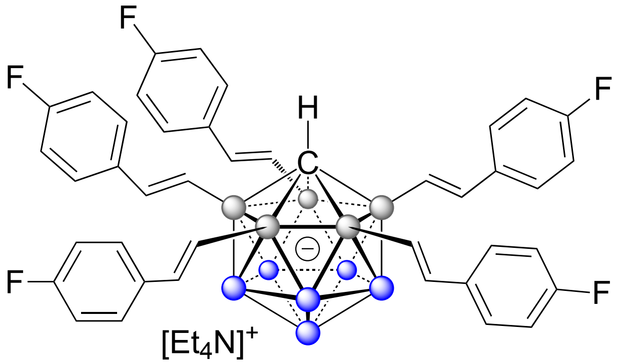

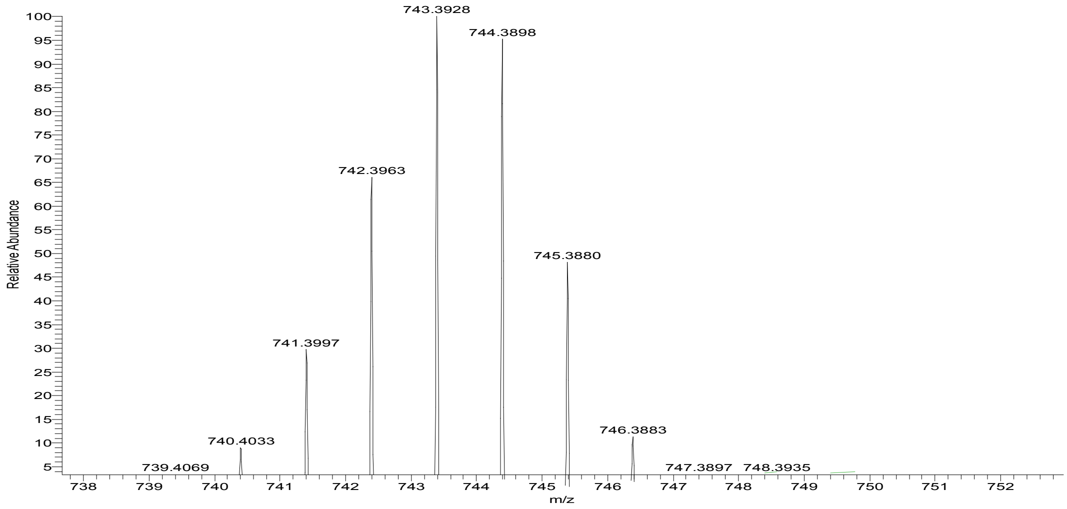

(–)-ESI-HRMS Shimadzu IT-TOF

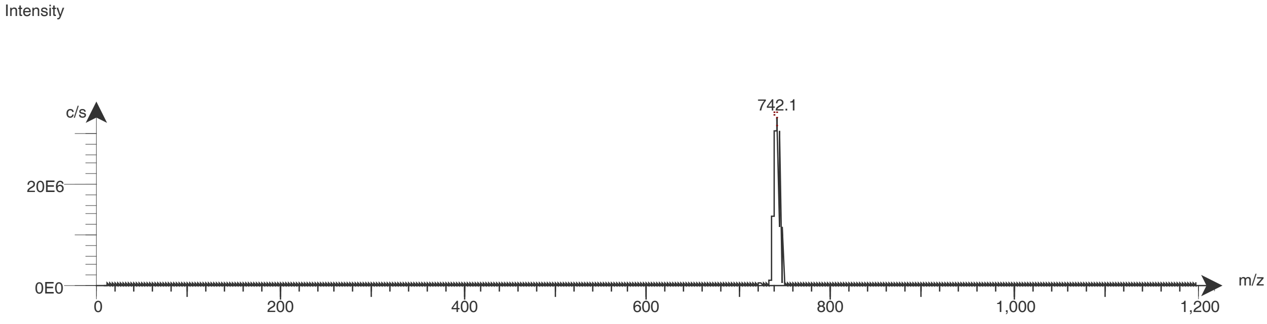

Full-range (–)-ESI-MS Expression CMS

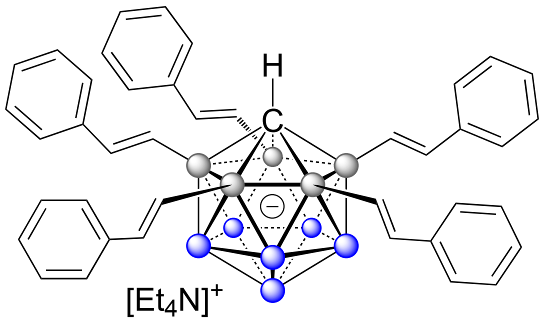

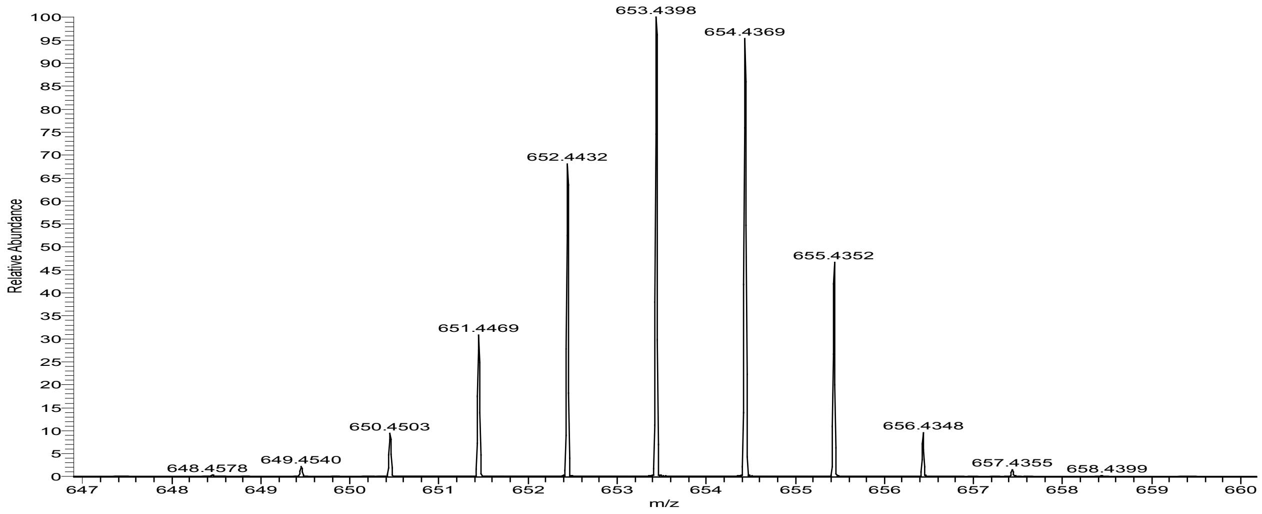

(–)-ESI-HRMS Shimadzu IT-TOF

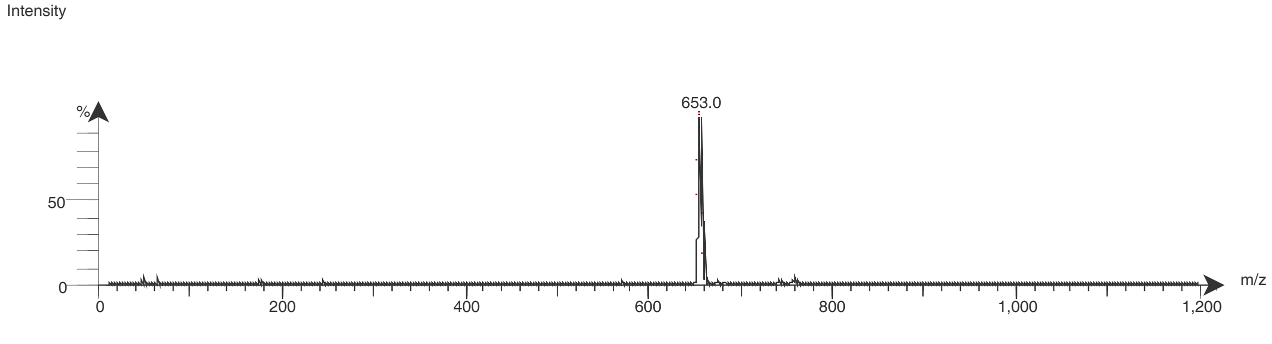

Full-range (–)-ESI-MS Expression CMS

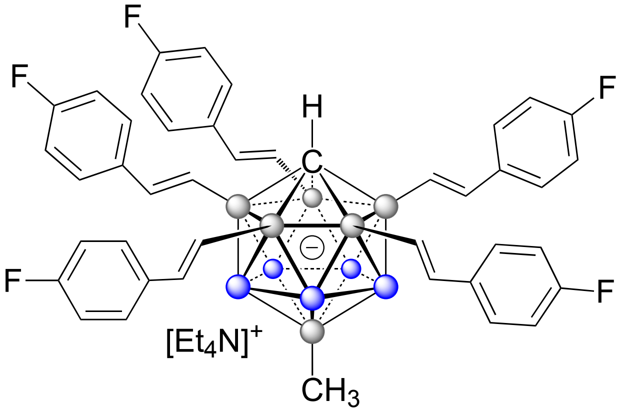

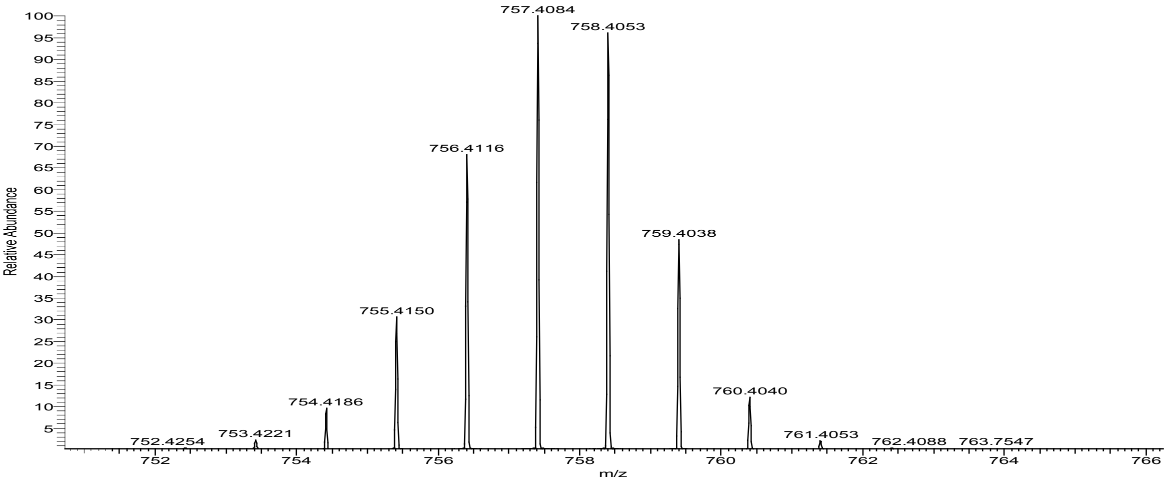

(–)-ESI-HRMS Shimadzu IT-TOF

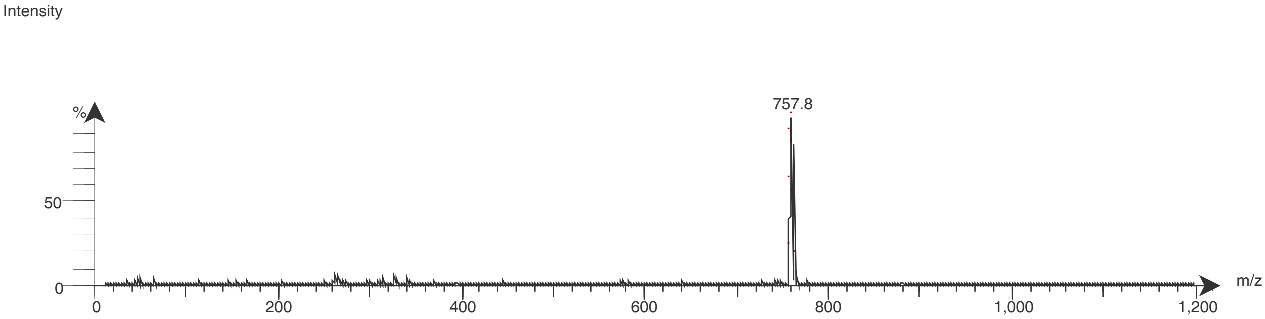

Full-range (–)-ESI-MS Expression CMS

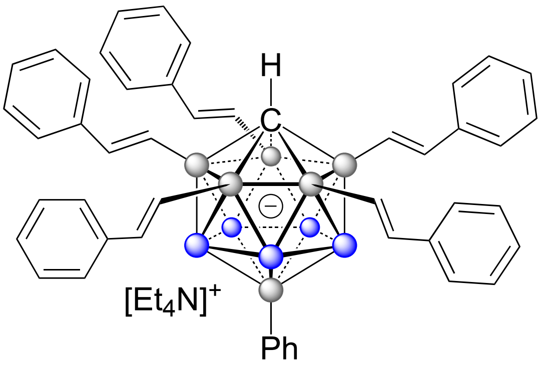

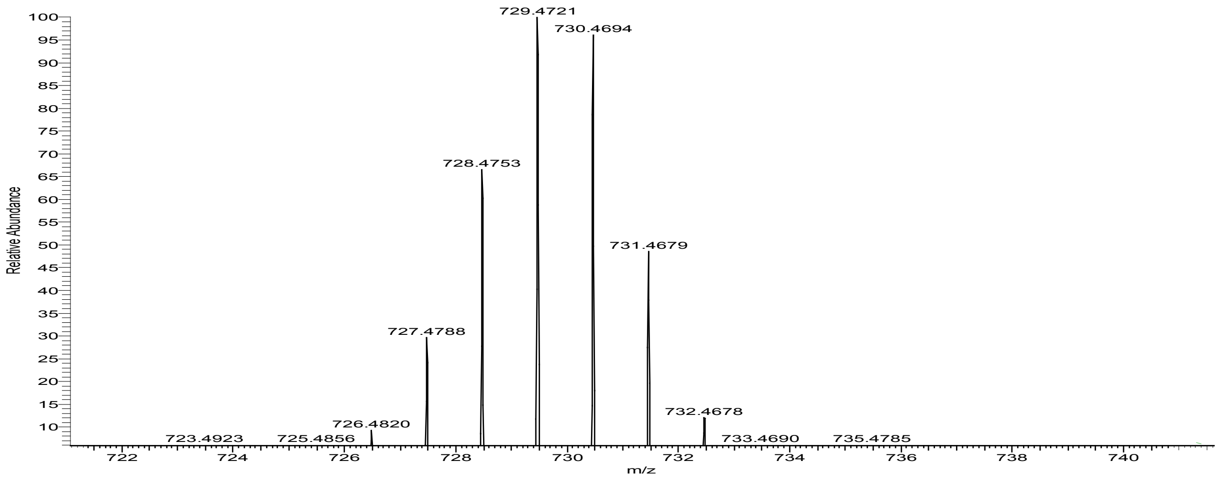

(–)-ESI-HRMS Shimadzu IT-TOF

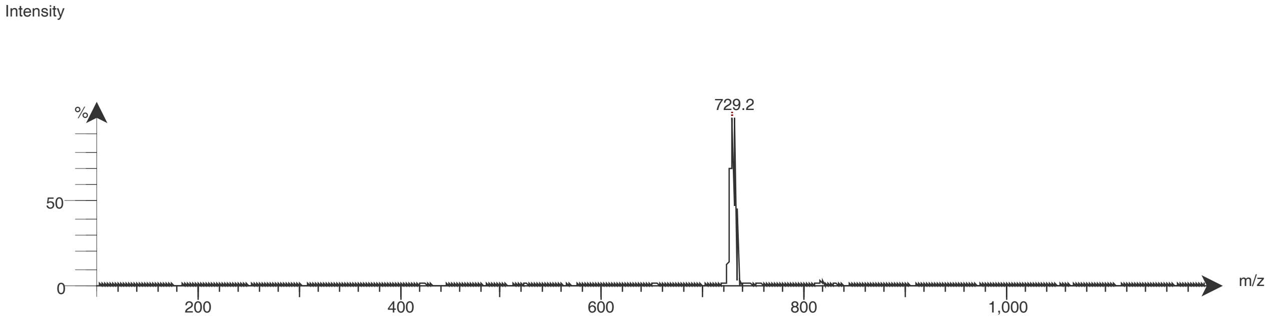

Full-range (–)-ESI-MS Expression CMS

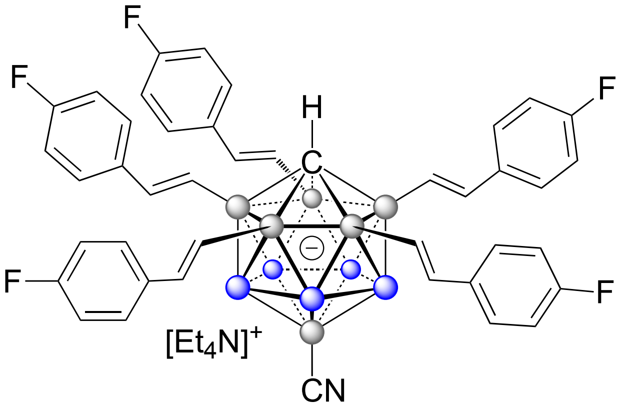

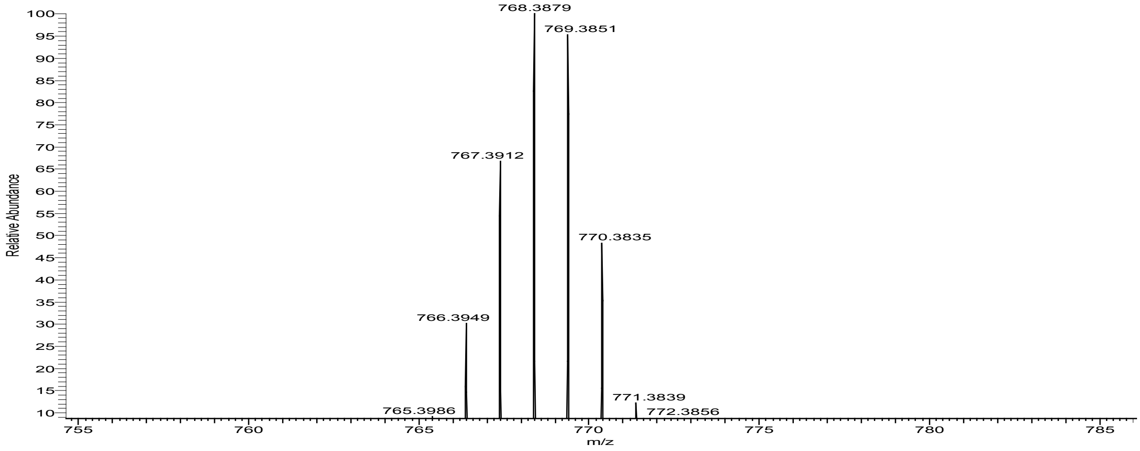

(–)-ESI-HRMS Shimadzu IT-TOF

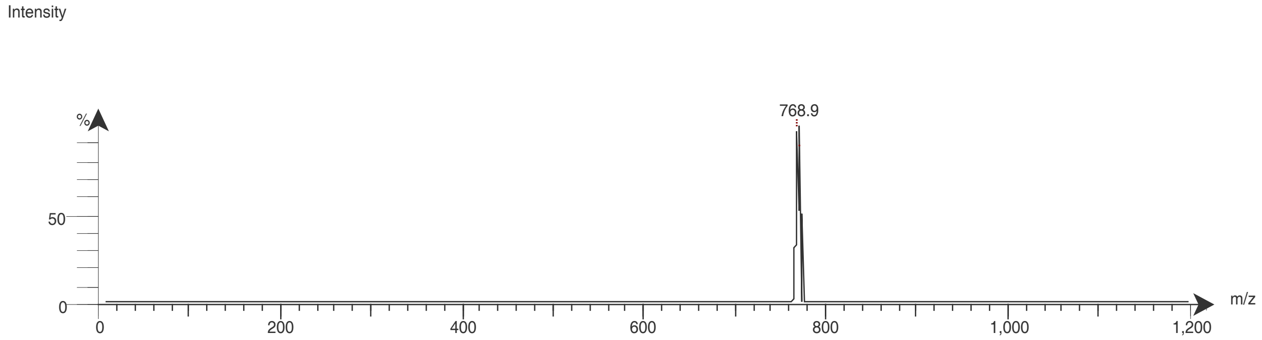

Full-range (–)-ESI-MS Expression CMS

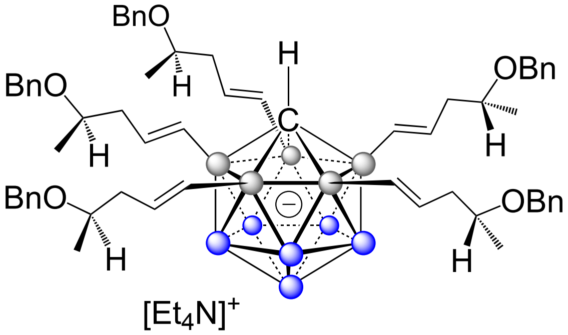

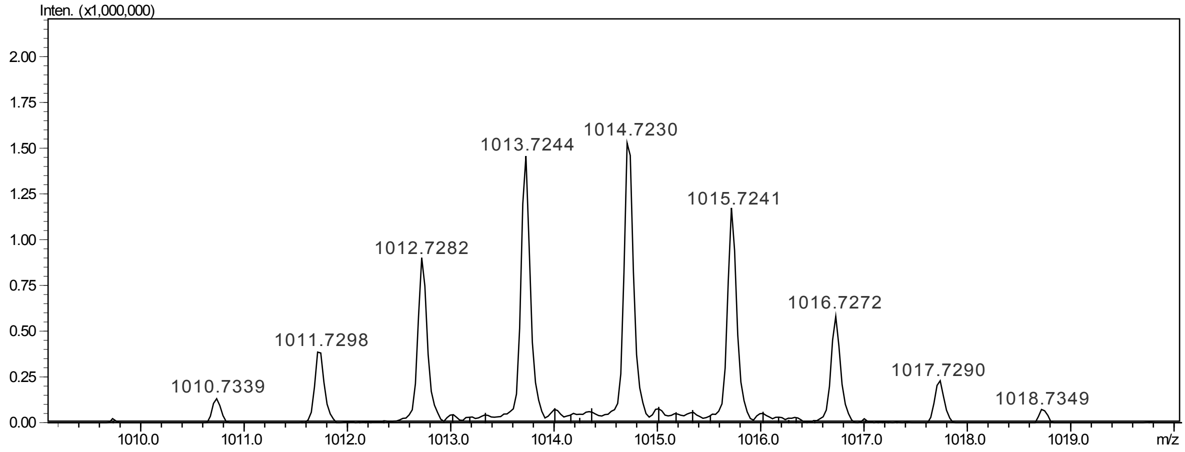

(–)-ESI-HRMS Shimadzu IT-TOF

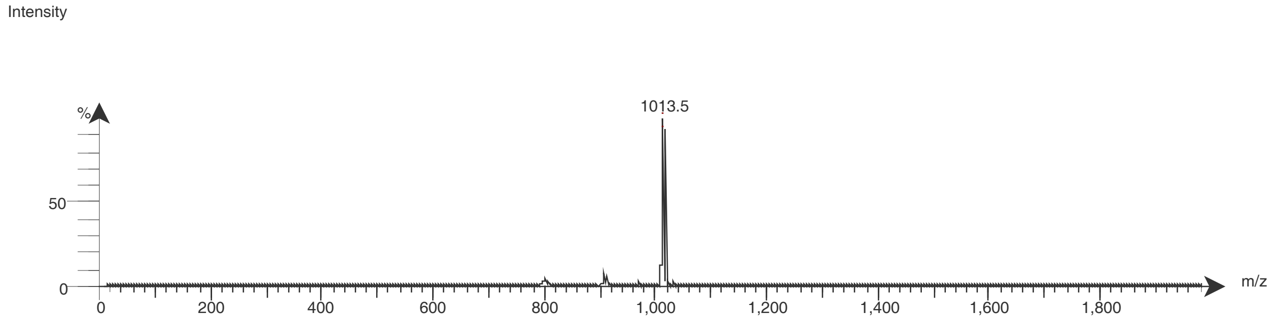

Full-range (–)-ESI-MS Expression CMS

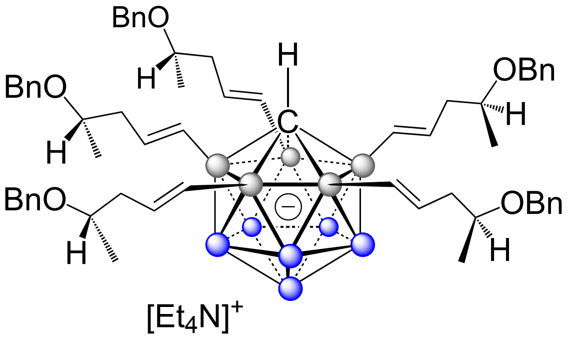

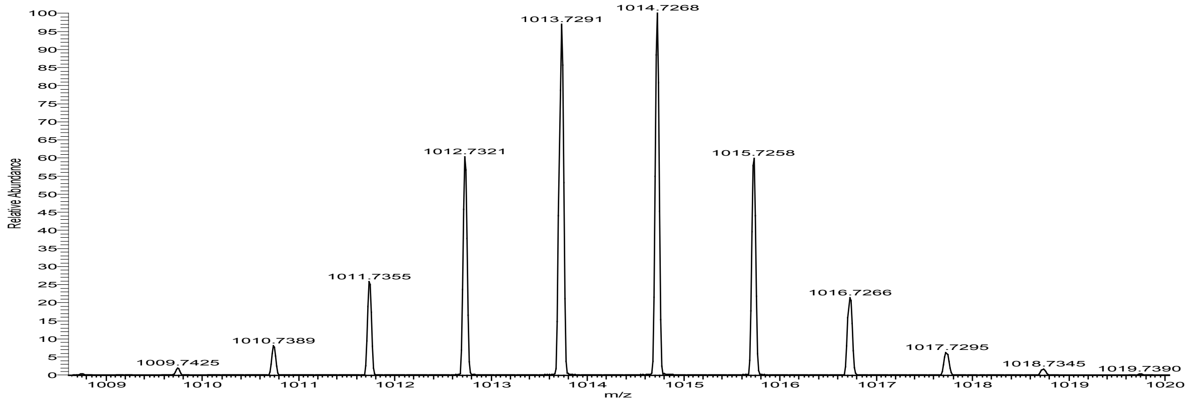

(–)-ESI-HRMS Shimadzu IT-TOF

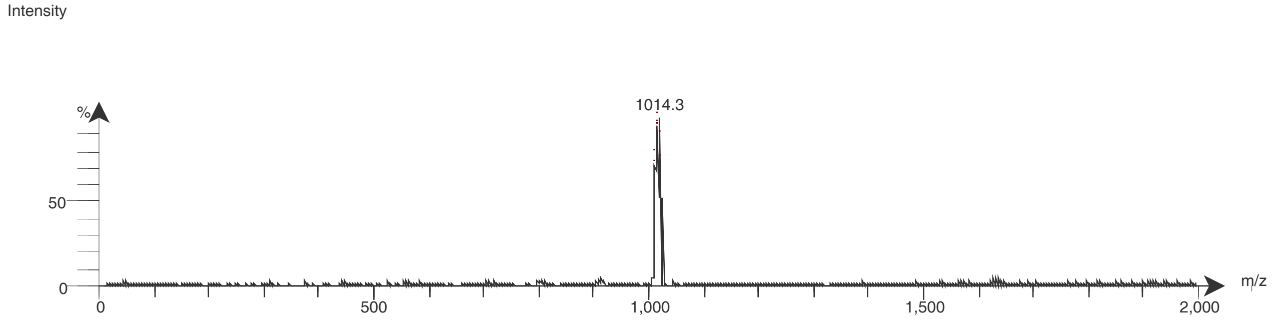

Full-range (–)-ESI-MS Expression CMS

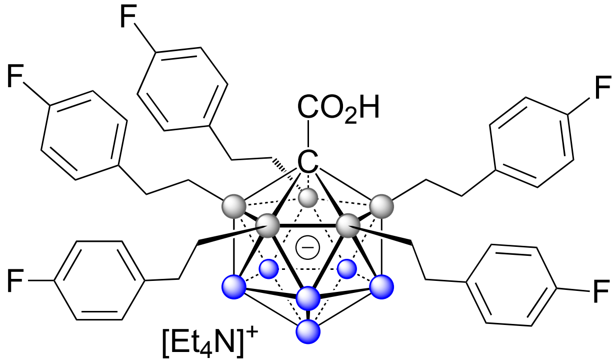

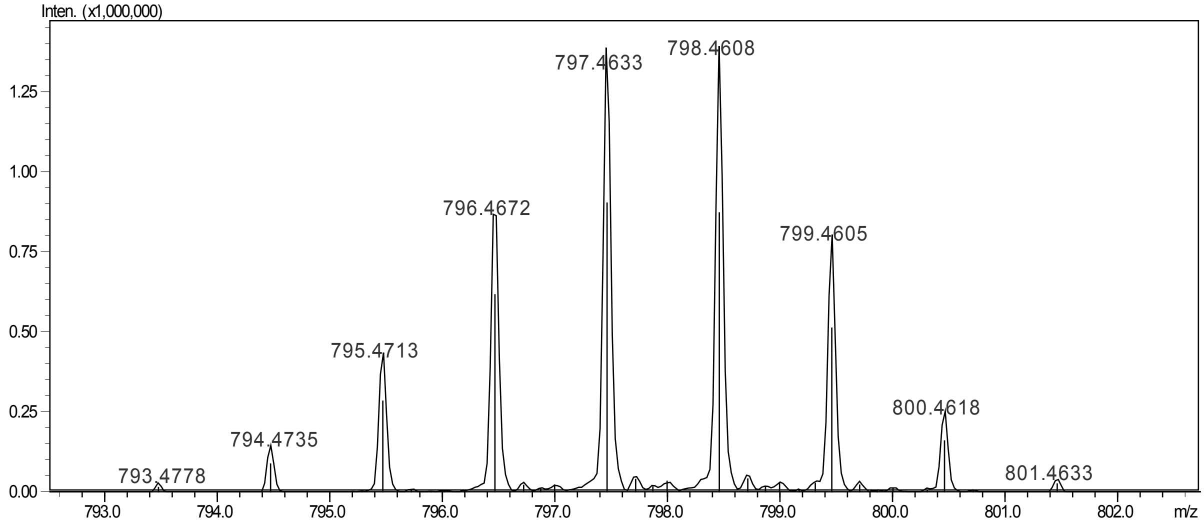

(–)-ESI-HRMS Shimadzu IT-TOF

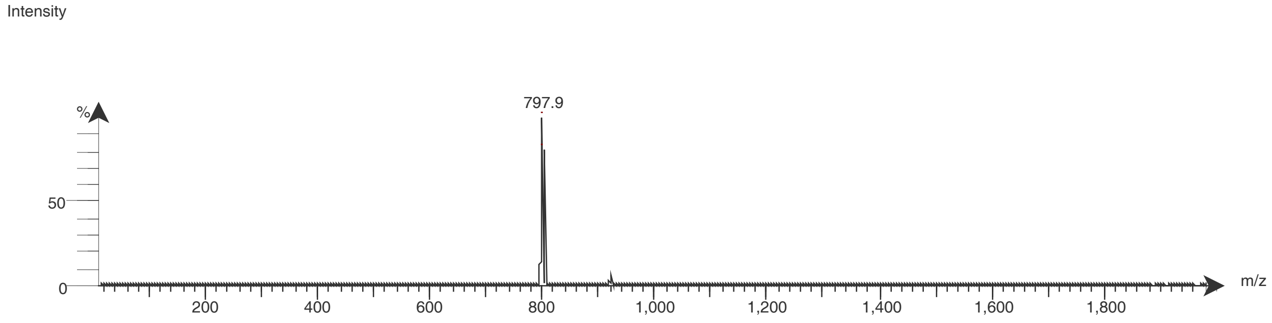

Full-range (–)-ESI-MS Expression CMS

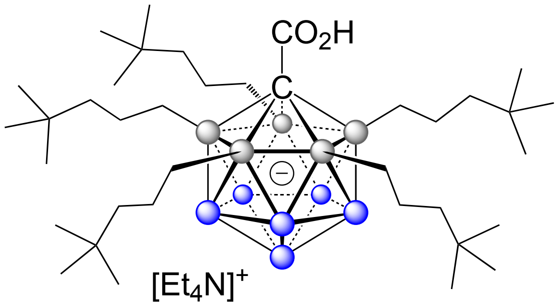

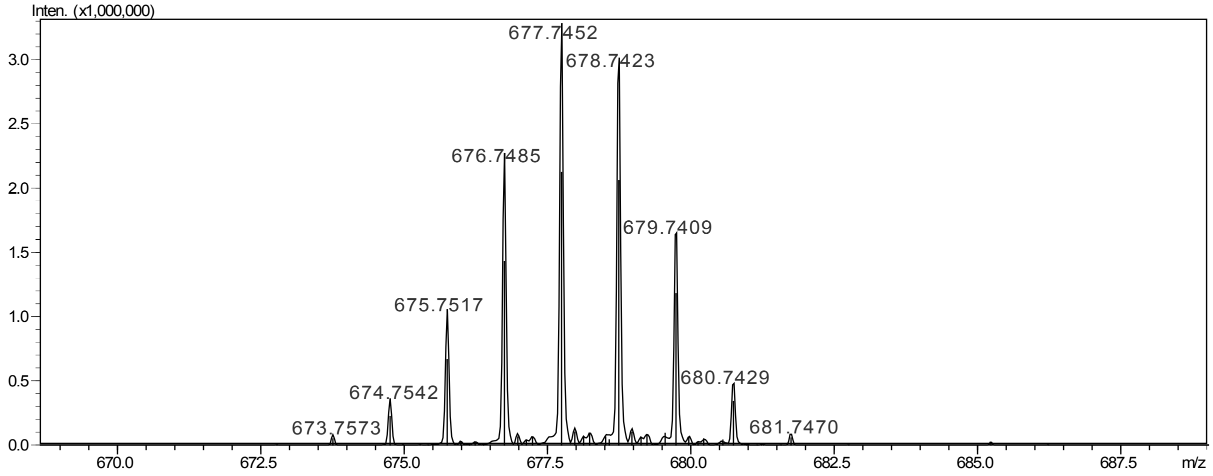

(–)-ESI-HRMS Shimadzu IT-TOF

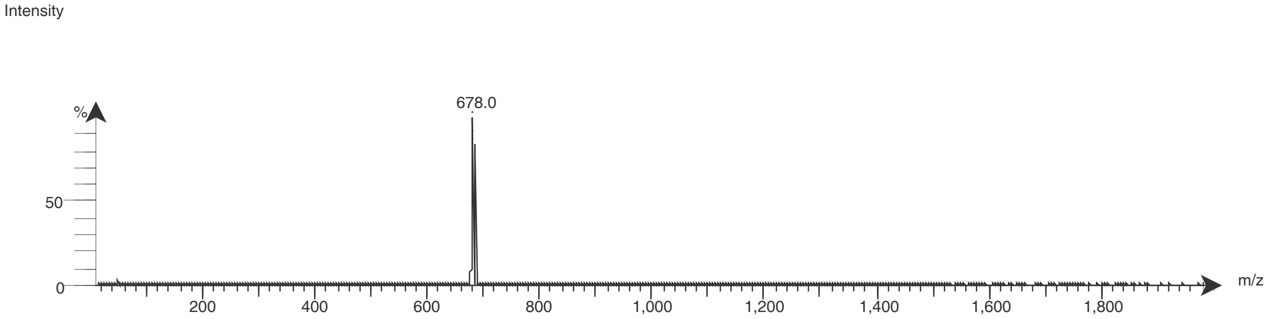

Full-range (–)-ESI-MS Expression CMS
